# Zipper polydefluorination-monoborylation of perfluoroalkyl chains

**DOI:** 10.1038/s41467-026-77437-9

**Published:** 2026-09-04

**Authors:** Sanne L. Albers, Johanna K. Harzendorf, Marie Deliaval, Johanne Köllmann, Jan Streuff

**Affiliations:** Department of Chemistry for Life Sciences, Uppsala University, Uppsala, Sweden

**Keywords:** Synthetic chemistry methodology, Pollution remediation, Organic chemistry

## Abstract

Selective defluorination of perfluoroalkyl chains remains challenging because of the strength of C–F bonds and the inertness of perfluoroalkyl groups. Here, the authors report a zipper reaction that completely defluorinates perfluoroalkylated alkenes into synthetically useful linear alkyl boranes while preserving the carbon backbone. The reaction is scalable and allows the recovery of fluoride in the form of LiF by simple filtration. It can be further applied to *gem*-difluoroalkenes, which are concluded to be intermediates of the underlying iterative fluoride removal process.

## Abstract (web summary)

Selective defluorination of perfluoroalkyl chains remains challenging because of the strength of C–F bonds and the inertness of perfluoroalkyl groups. Here, the authors report a zipper reaction that completely defluorinates perfluoroalkylated alkenes into synthetically useful linear alkyl boranes while preserving the carbon backbone.

## Introduction

**Fig. 1:**
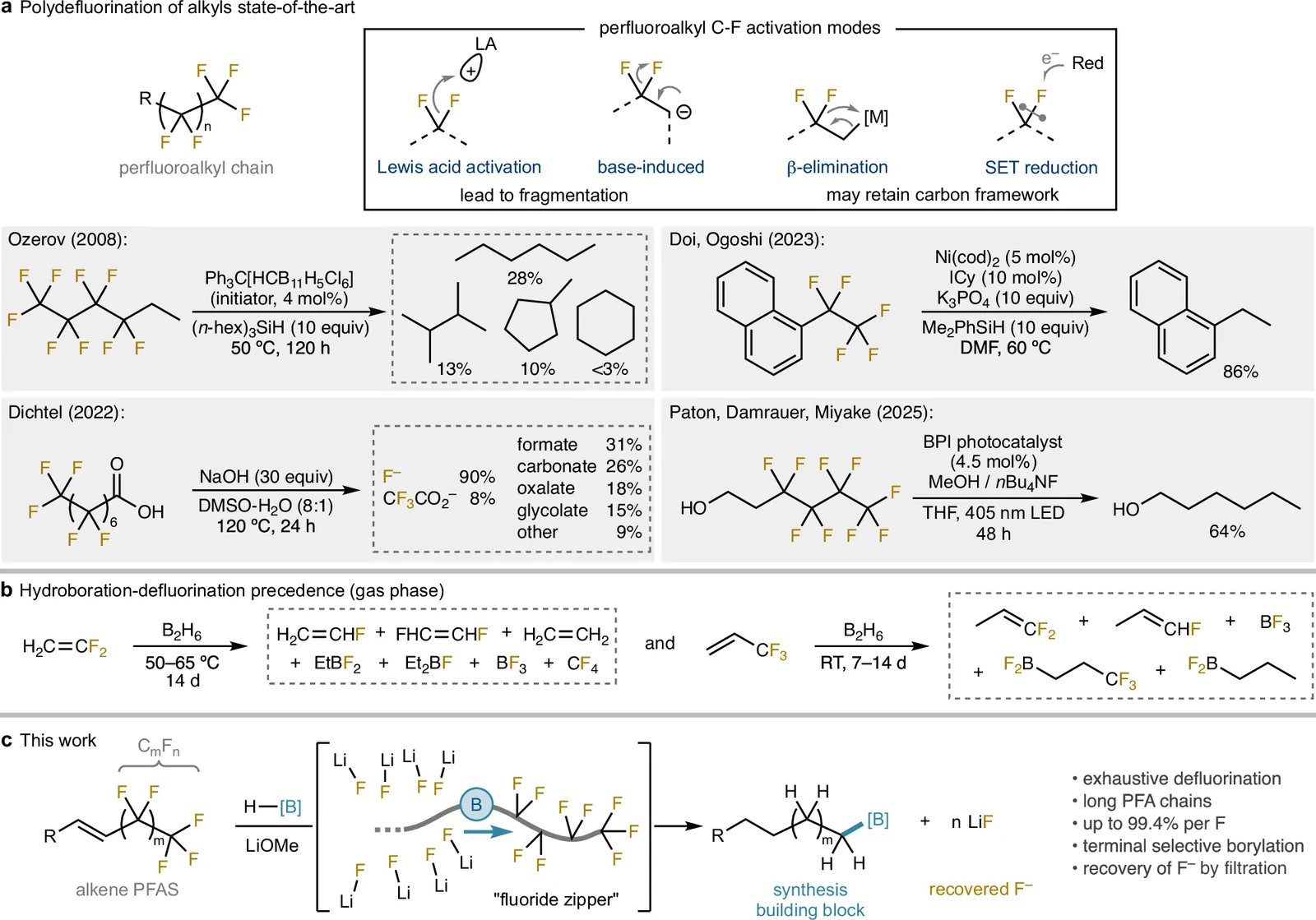
PFAS defluorination strategies. **a** C–F activation modes for the defluorination of perfluoroalkyl chains and corresponding state-of-the-art methods. BPI = benzo[ghi]perylene monoimide. **b** Precedence of defluorination in the presence of diborane in gas phase reactions. **c** The polydefluorination-borylation strategy explored in this work. Drawn hydrogen atoms indicate removed fluorides.

The removal and substitution of fluoride substituents in organic molecules is particularly challenging due to the strength of C–F bonds, and thus the development of C–F activation methods has been a major research topic during the past decades^1–7^. In particular, perfluorinated alkyl groups are chemically inert and highly apolar, which makes them notoriously difficult to defluorinate and renders perfluoroalkyl substances (PFAS) persistent and bioaccumulating with numerous associated negative environmental and health effects^8–11^. While most research on aliphatic C–F activation using molecular reagents and catalysts has focused on fluorinated benzylic, allylic, and α-carbonyl carbons that facilitate the cleavage through stabilization of ionic or radical intermediates^12,13^, the defluorination of longer perfluorinated alkyl chains is significantly more difficult and less explored. One main reason is that the oxidative addition of a PFAS C–F bond to a transition metal reagent or catalyst is thermodynamically and kinetically hindered. Nevertheless, the defluorination of perfluoroalkyls has been reported via several alternative activation modes shown in Fig. 1a together with state-of-the-art examples: i) Strong Lewis acids such as silylium cations can abstract a fluoride, which leads to reactive carbocations that are then prone to rearrange, as was shown by the Ozerov group for the defluorination of a nonafluorohexane, giving various hexane isomers^14^. ii) The base-induced (decarboxylative) generation of a carbanion followed by an E1cB fluoride elimination is the basis of several previously reported PFAS destruction and mineralization methods^15–20^. For example, perfluoroalkanoic acids undergo fragmentation in the presence of base at elevated temperatures to form a range of carboxylates and fluoride^17^. The recovery and reuse of fluoride is an important aspect of such approaches and recent examples featuring mechanochemical and reductive strategies that achieve high fluoride yield and purity^19–23^. Alternative pathways that avoid the destruction of the carbon backbone are iii) the β-F-elimination from metalated intermediates that have previously been explored in the form of titanocene- and zirconocene-promoted partial defluorinations^24–26^ and more recently as part of the shown nickel-catalyzed exhaustive defluorination of 1-(pentafluoroethyl)naphthalene^27^. iv) Single-electron transfer reagents or photoreductive conditions can be harnessed to defluorinate via a homolytic C–F cleavage. For example, radical defluorinations with stoichiometric zirconium reagents have been reported^28^, and more recently, the photocatalytic hydrodefluorination of various PFAS substances has been demonstrated^29^. While these are impressive examples, these methods typically suffer from either long reaction times, low conversion for extended perfluoroalkyl chains, or fragmentation of the carbon backbone. Moreover, the outcome is often reported as conversion determined by gas or ion chromatography of the reaction mixture and not as the yield of isolated products. Hence, there is a great need for operationally simple and quantitative defluorination methods that are scalable and allow the recovery of both fluoride *and* the organic molecular skeleton^30^. Even more desirable would be the conversion into synthetically useful building blocks through the introduction of a new functional group, which would lead to new directions in the field.

More than 60 years ago, it was reported that exposing polyfluoroethylenes or 3,3,3-trifluoropropylene in the gas phase to diborane (B_2_H_6_) leads to mixtures of defluorinated products as well as defluorination-borylation products (Fig. 1b)^31,32^. Although unselective and incomplete, these examples demonstrated intriguing defluorination reactivity of a borane. Defluoroborylations have further been reported by means of transition metal catalysis, but only for fluoroarenes and alkenes, and rarely for more than a single fluorine atom at a time^33–35^. Our group has been engaged for several years in the development of heterodefunctionalizations of alkenes and alkene-tethered alkyls using zirconium catalysis^36–38^. We now disclose a strikingly simple, exhaustive polydefluorination-monoborylation reaction that addresses all of the above-stated challenges and converts alkene-bound perfluorinated alkyls and *gem*-difluoroalkenes into alkyl pinacolboronates (Fig. 1c). Moreover, it allows the straightforward recovery of fluoride as LiF by filtration on a gram scale.

## Results and discussion

### Reaction Development

While investigating zirconium-catalyzed defluorinations in the presence of mild hydride transfer agents, we discovered that β-trifluoromethylstyrene **1a** underwent an exhaustive poly-defluorination with pinacol borane (HBpin) and LiOMe at 85 °C in the absence of a transition metal catalyst (Table 1, entry 1). A concomitant borylation took place, which resulted in an overall 95% conversion of the perfluoroalkylated alkene to linear alkylborane product **2a** as determined by ^1^H NMR. Reducing the amount of LiOMe or attempting the reaction in the absence of it led to lower conversion or no product formation, respectively (entries 2, 3). Furthermore, the lithium cation was important as sodium methoxide did not promote the reaction (entry 4), and other lithium alkoxides were inferior to LiOMe. While LiOEt still gave 85% conversion to the product, bulky LiO*t*-Bu reduced the amount to 7% (entries 5, 6). Lowering the reaction temperature to 60 °C or changing the solvent from the sustainable 2-MeTHF to THF slightly diminished the product formation to 72% and 86%, respectively (entries 7, 8). Moreover, 2.5 equiv of HBpin with respect to each fluorine and the final hydroboration were required to achieve full conversion to the desired product, as was demonstrated by lowering the overall amount to 8 equiv (entry 9). Increasing the concentration from *c* = 0.2 M to *c* = 0.4 M also led to a slight decrease in conversion to the product (entry 10). Importantly, borane **2a** was the only product received under the optimal conditions, besides a small amount (~5%) of the branched isomer (entry 1). A corresponding substrate without the alkene functionality did not undergo the defluorination-borylation reaction (see Supplementary Fig. 1).

**Table 1:** Variation of the reaction conditions


Entry	Change in conditions	Conversion to product^a^
1	none	95%
2	2 equiv LiOMe	87%
3	no LiOMe	0%
4	NaOMe instead of LiOMe	0%
5	LiOEt instead of LiOMe	85%
6	LiO*t*-Bu instead of LiOMe	7%
7	reaction at 60 °C	72%
8	THF as solvent, 60 °C	86%
9	8 equiv HBpin	72%
10	*c* = 0.4 M	84%

^a^ Determined by ^1^H NMR of the crude reaction mixture with benzyl benzoate as internal standard.

### Reaction scope and scalability

**Fig. 2:**
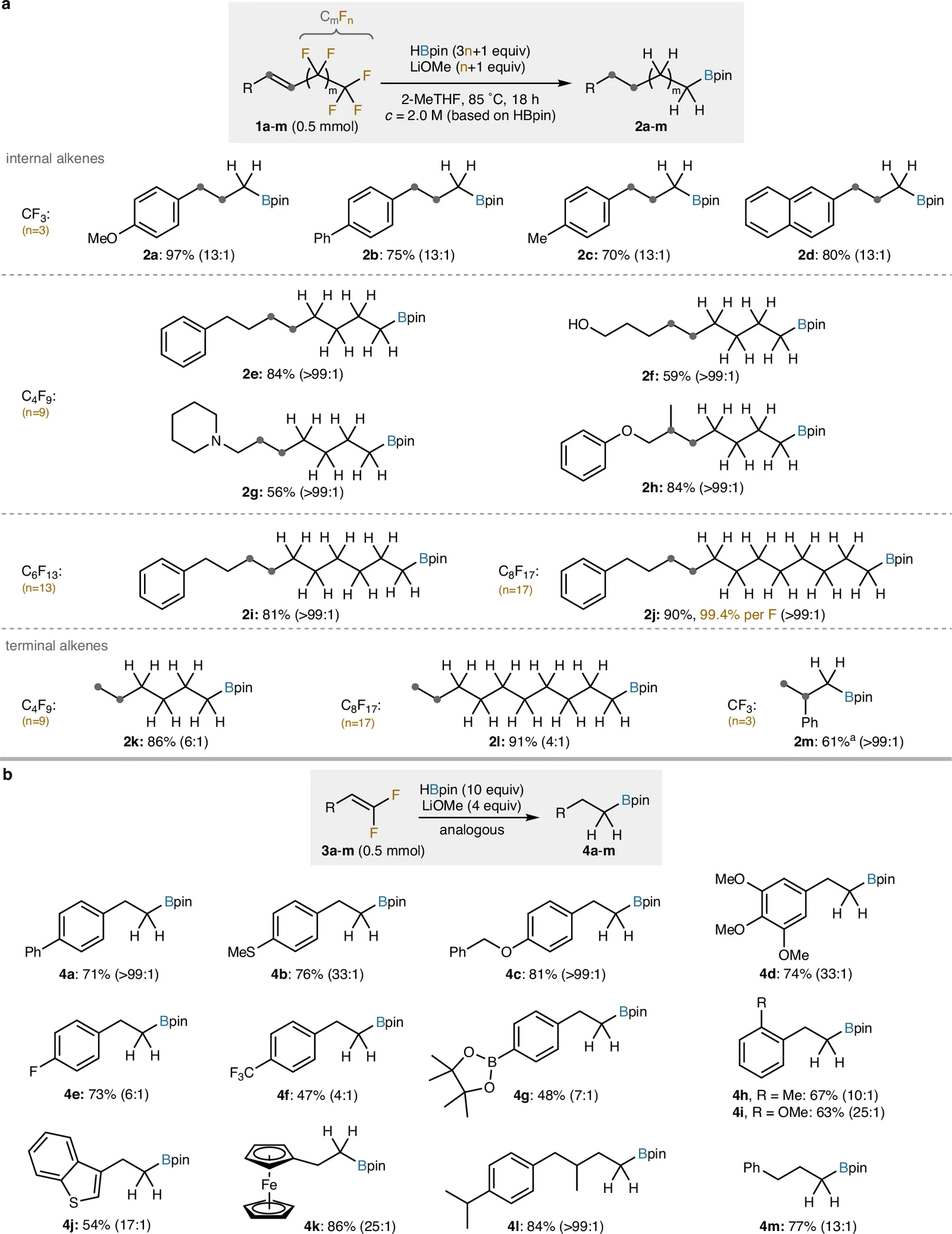
Scope of the polydefluoro-monoborylation. Drawn hydrogen atoms indicate removed fluorides, and brackets show linear-branched product ratios. All yields refer to isolated material. **a** Perfluoroalkylated alkenes. *n* = number of removed fluorides. Conditions: substrate **1** (0.5 mmol), HBpin (3*n* + 1 equiv), LiOMe (*n* + 1 equiv), 2-MeTHF (*c* = 2.0 M based on HBpin), 85 °C, 18 h. **b** *gem*-Difluoroalkenes. Conditions: substrate **3** (0.5 mmol), HBpin (10 equiv), LiOMe (4 equiv), 2-MeTHF (*c* = 2.0 M based on HBpin), 85 °C, 18 h. ^a^ (3,3,3-Trifluoro-2-phenylpropyl)pinacolborane was observed as a major byproduct.

We then explored the polydefluoro-monoborylation reaction on a range of perfluoroalkylated *E*-alkenes (<10% *Z*-isomer content) on a 0.5 mmol scale and determined the yield of the isolated borylation product (Fig. 2a). In all cases, the linear alkyl borane was received as the main product in high regioselectivity. Methoxystyrene **1a** gave an excellent 97% yield in **2a** after 18 h. Other β-trifluoromethyl styrene derivatives, as well as a corresponding naphth-2-yl alkene, gave good yields of 70–80% (**2b**–**d**). Here, substrates **1b** and **1d** had a higher 29% and 33% *Z*-isomer content, which did not seem to affect the yield. Gratifyingly, substrates with longer perfluoroalkyl chains attached to an alkene moiety also smoothly underwent the reaction, as is shown for *n-*perfluorobutyl alkene **1e**, which gave 84% of alkylborane **2e**. Even a primary alcohol function was tolerated, and exhaustive defluorination occurred, giving 59% yield in product **2f**. Moreover, a tertiary amine as well as a trisubstituted alkene were well-tolerated, giving products **2g** and **2h** in 56% and 84%. Even *n*-perfluorohexyl and *n*-perfluorooctyl groups were quantitatively defluorinated and gave the corresponding products **2i** and **2j** in 81% and 90% yield, respectively. In the case of the latter, this corresponded to an outstanding defluorination performance of 99.4% per fluorine. Importantly, terminal alkenes smoothly underwent the polydefluorination-monoborlyation as well. Primary alkenes with perfluoroalkyl chains, such as **1k** and **1l**, are common precursors of PFAS polymers, and both gave the desired borane in high yield. In addition, a 1,1-disubstituted alkene (α-trifluoromethylstyrene, **1m**) gave product **2m** in 61% yield with the linear hydroboration product (3,3,3-trifluoro-2-phenylpropyl)pinacolborane as a major byproduct. This successful polydefluorination-monoborylation of longer perfluoroalkyl chains (products **2e**–**2l**) also pointed towards an iterative mechanism (*vide infra*).

Since *gem*-difluoroalkenes are readily available and versatile synthesis building blocks^39,40^, we investigated the transfer of our method towards this compound class and tested the performance in the presence of various functional groups under the optimized conditions of Table 1, entry 1 (Fig. 2b). Besides biphenyl substrate **3a**, electron-donating thioether and ether functions as well as electron-withdrawing fluoride, trifluoromethyl, and pinacolboryl groups were well-tolerated, resulting in yields of 47–81% (**4a**–**4g**). Here, 0–5% of the branched product was observed, with the exception of products **4e**–**g**, which contained electron-withdrawing aryl substituents that affected the final hydroboration selectivity (*vide infra*) and raised the amount of the branched isomer to 13–20%. Product **4f** further showed that a chemoselective conversion occurred in the presence of a benzylic trifluoromethyl group. *ortho*-Methyl- and methoxy-substituted phenyls were tolerated as well, as is shown for products **4h**, **4i** that were obtained in good yields. Further examples included a benzothiophene (**4j**), a ferrocene (**4k**), and a branched aliphatic compound (**4l**) with yields of 54%, 86%, and 84%, respectively, all of which gave the linear product with high selectivity. Importantly, allylic *gem*-difluoroalkene **3m** was smoothly converted into **4m** (77% yield), indicating that such *gem*-difluoroalkenes are potential intermediates in the reaction of PFA substrates **1**.

It is noteworthy that substrates **1a–j** were synthesized in one or two steps from perfluoroalkyl iodides or TFA, respectively^41,42^, and substrates **3** were directly accessible from sodium chlorodifluoroacetate^43^, all of which are commonly used PFAS or precursors thereof.

**Fig. 3:**
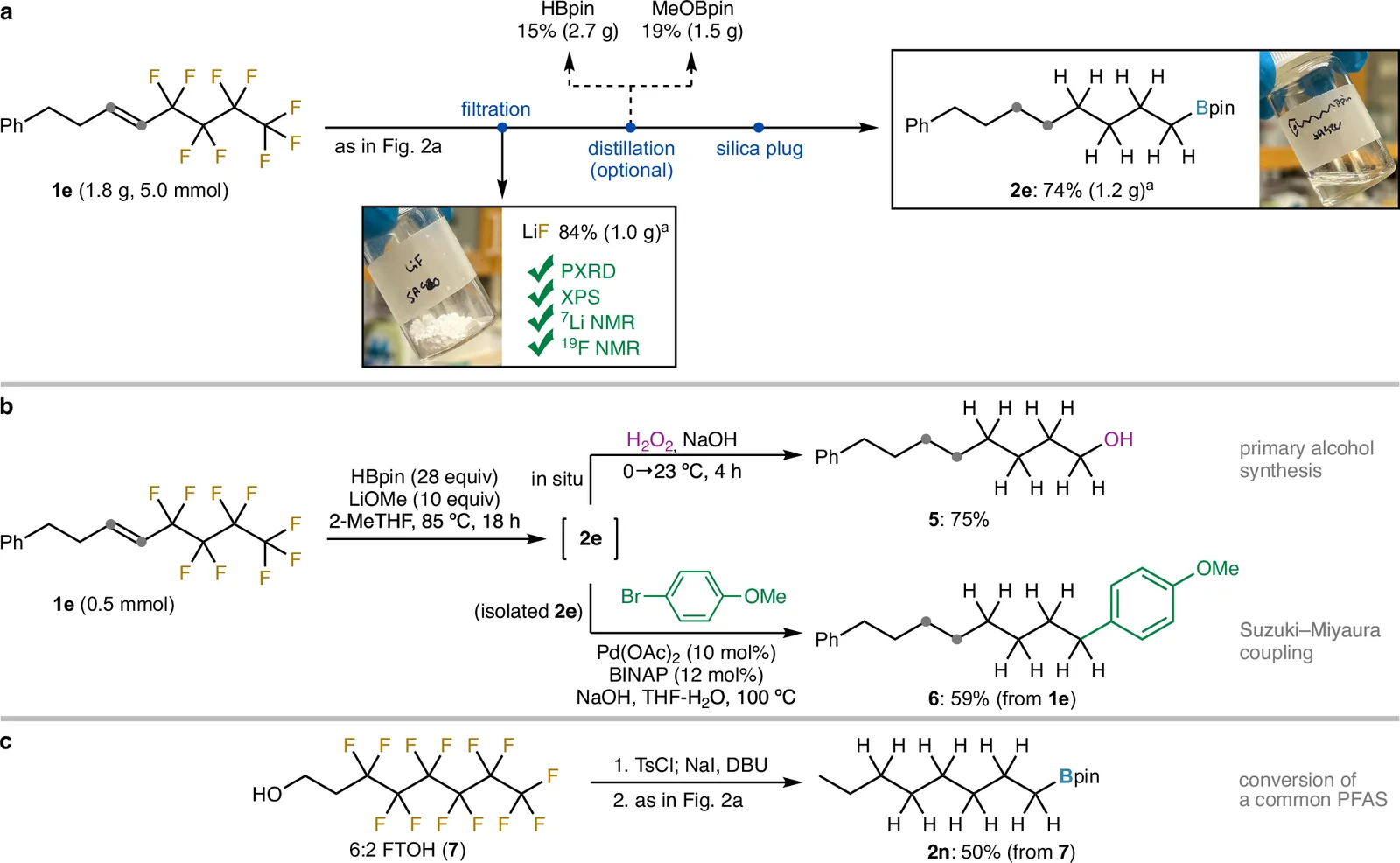
Scale-up with fluoride recovery and application examples. All yields refer to isolated material. **a** An experiment on a 5 mmol scale with isolation of LiF by filtration and optional isolation of HBpin and MeOBpin by distillation. **b** Defluorination-oxidation and sequential Suzuki coupling reactions. **c** Two-step conversion of a commercially used fluorotelomer alcohol into a primary borane. ^a^ An average of two experiments.

The scalability of the reaction and the recovery of fluoride were demonstrated by subjecting perfluoroalkene **1e** to the optimized conditions on a 5 mmol scale (Fig. 3a). For all examples in Fig. 2, it was observed that a white solid formed, which was now conveniently isolated by filtration followed by washing with methanol. It was identified as LiF by powder X-ray diffraction analysis (PXRD) and X-ray photoelectron spectroscopy (XPS). The purity of the LiF obtained from two experiments was further determined by XPS and was found to be at least equal to a commercial sample of 99.98% purity (trace metal basis). In addition, the ^7^Li and ^19^F NMR (in D_2_O) spectra matched a commercial sample, and organic and boron-containing material was absent as determined by ^1^H and ^11^B NMR, respectively. The average yield in LiF was 84% (755% based on **1e**). In one of the experiments, no MeOH was added in the workup, and a short path distillation of the mother liquor was carried out, which allowed the recovery of 2.7 g of HBpin (15%) and the isolation of 1.5 g of MeOBpin (19% based on LiOMe), a byproduct of the reaction. Purification of the remaining material gave **2e** in an average yield of 74%.

To further demonstrate the usefulness of our approach, we demonstrated the direct conversion of **1e** into primary alcohol **5** by changing to an oxidative workup (Fig. 3b). Separately, following the purification of **2e**, the Suzuki coupling with 4-bromoanisole gave **6** in an overall yield of 59% from **1e**. Finally, fluorotelomer alcohol **7**, an industrially used PFAS, was converted into borane **2k** by elimination of the alcohol followed by polydefluorination-borylation in an overall 50% yield.

### Elucidation of the reaction mechanism

We then carried out preliminary investigations to probe the reaction mechanism. The defluorination of 3,3,3-trifluoropropylene (see Fig. 1b) was first proposed to proceed via a *syn*-β-F-elimination after hydroboration^32^. But later, related 1,2-deborochlorination and 1,2-deborobromination reactions after hydroboration have been reported, for which an E2-type *anti*-elimination mechanism was established if a boron-ate complex-forming base, such as *n*-BuLi, or a Lewis acid was present^44,45^. We therefore suspected that the defluorination steps in our reaction also occurred via a similar *anti*-elimination mechanism and that the overall polydefluorination-monoborylation reaction resulted from iterative repetition of hydroboration-elimination events.

**Fig. 4:**
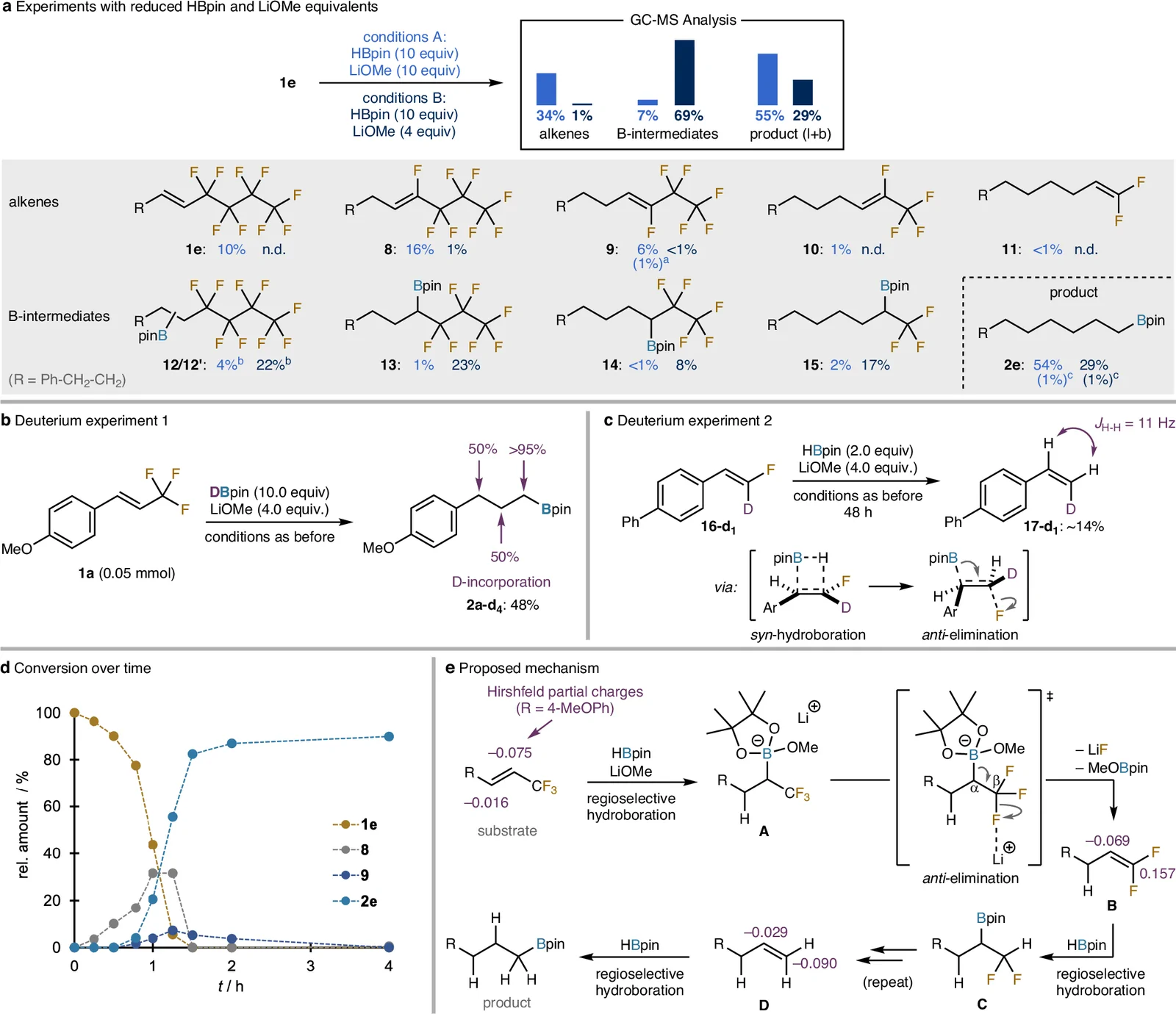
Elucidation of the reaction mechanism. **a** Experiments with reduced HBpin (conditions A) and reduced HBpin/LiOMe (conditions B) followed by GC-MS analysis of the reaction mixture. Alkenes **8**–**10** were assigned to be the shown *Z*-isomers based on their lower relative energy. **b** Defluoro-deuteroboration of **1a** with DBpin. **c** Experiment with a deuterated fluorostyrene and 2.0 equiv HBpin proving the *anti*-elimination. **d** Reaction time course plot from individual reactions stopped after a certain time, followed by GC-MS analysis. **e** Proposed mechanism featuring iterative hydroboration/*anti*-F-elimination steps. Calculated Hirshfeld partial charges indicate the expected hydroboration regioselectivity. ^a^ A small amount of the minor *E*-isomer (**9’**) was detected. ^b^ Combined amount of two regioisomers **12**/**12’** that could not be assigned. ^c^ Amount of the branched product regioisomer.

In order to probe this iterative mechanism, an experiment with **1e** and a reduced amount of borane (conditions A) was carried out and the reaction mixture was analyzed by GC-MS (Fig. 4a). In addition to still unreacted **1e** (10%), multiple partially defluorinated alkenes **8**–**10** (24%) and, in smaller quantity, borane (7%) species **12**–**15** were identified based on their mass and fragmentation pattern. Product **2e** was the main compound (54%), and 1% of the branched isomer was detected as well. The detailed composition of detected species is shown in the gray box in Fig. 4a. In a second experiment, both reagents, borane and lithium methoxide, were reduced (conditions B), which, in contrast, led to mainly borylated intermediates being present (69%) and only traces of fluorinated alkenes. Although not all expected intermediates could be detected, these two experiments support the iterative mechanism, and that lithium methoxide promotes the 1,2-deboro-defluorination.

In a separate experiment, trifluoromethyl styrene **1a** was reacted with deuterated pinacolborane DBpin. NMR analysis revealed 50% deuterium incorporation in the benzylic and homobenzylic position as well as full deuteration at the former trifluoromethyl carbon (Fig. 4b). This deuterium distribution matched the expected distribution from an iterative hydroboration-elimination mechanism (see Supplementary Fig. 18). A second deuterium study was then carried out using selectively *cis*-monodeuterated fluoroalkene **16**-**d**_**1**_ and a reduced amount of HBpin (Fig. 4c). This allowed the observation of alkene intermediate **17**-**d**_**1**_ in the crude reaction mixture after 48 h, which was again *cis*-monodeuterated. Since the alkene hydroboration is well-known to proceed with *syn* selectivity, the elimination step that removes the boryl and fluoride substituents must proceed via an *anti* mechanism to be in agreement with the observed *cis*-deuterated product.

Due to the strength of the B-F bond, a boron Lewis acid-assisted C–F cleavage followed by rapid fluoride-methoxide exchange was a possible scenario, in particular because FBpin readily interconverts into MeOBpin and LiF in the presence of LiOMe (see Supplementary Notes 9), which would explain the absence of B-F byproducts. However, using a separately prepared α-trifluoromethyl alkylborane, we could experimentally confirm that the 1,2-elimination is triggered by the addition of LiOMe without the requirement of a boron-based Lewis acid (see Supplementary Notes 9). Hence, a fluoride abstraction mechanism under direct formation of LiF and without intermediary B-F species is most likely.

We then recorded a time-course plot of the reaction of **1e** using both NMR and GC-MS analysis, which showed that the reaction reached completion already after 4h and revealed alkenes **8** and **9** as main intermediates. The other species **10**–**15** were detected in relative amounts of <5% and are not plotted for reasons of clarity. More intriguing, however, is the observation of a sigmoid curve for the substrate conversion and thus an initiation period. In the past, it was proposed that alkene hydroborations with HBpin require a catalytic amount of BH_3_ to be present, and the slow formation of the latter could explain this behavior^46,47^. However, distilling HBpin, which may remove trace amounts of BH_3_, did not affect the reaction outcome. Neither did the addition of BH_3_•THF (10 mol%) improve the initial conversion rate. Instead, it was discovered that pre-stirring HBpin and LiOMe for 1 h at 85 °C led to a dramatically improved conversion to **2e** within the first 30 min (50% vs 0%). ^11^B NMR revealed the formation of [(MeO)HBpin]^–^ together with MeOBpin and BH_4_^–^ during the pre-stirring at 85 °C, without detection of BH_3_ (or B_2_H_6_). Presumably, this ate complex has a stronger hydricity than HBpin and is the active hydroboration agent. We further suspect that this premature conversion of HBpin into non-productive MeOBpin and BH_4_^–^ leads to the requirement of an excess of borane reagent. It should be further noted that for alkenes in conjugation with an arene such as **2a**, the overall reaction was significantly slower, requiring 18 h to reach completion (see Supplementary Fig. 29).

Altogether, these results are in agreement with the mechanism shown in Fig. 4d. It starts with the hydroboration of the alkene function, which is accelerated by the presence of LiOMe through boron-ate complex formation. The regioselectivity is governed by the increased charge density at the CF_3_-bearing alkene carbon, favoring the borylation at this position and leading to intermediate **A**^48,49^. This is supported by the Hirshfeld charge density values at these alkene carbons (for R = 4-MeOPh)^50^ that were calculated at the RI-TPSS-D4-CPCM(2-MeTHF)/def2-TZVP level with Orca 6^51–56^. The lithium cation then activates one of the fluorine atoms in the β-position, which facilitates the *anti*−1,2-elimination via the shown transition state to give *gem*-difluoroalkene intermediate **B**. Hydroboration then leads to the formation of **C**, which is in agreement with the species observed by GC-MS and by NMR in the time-course experiment. Repeated elimination and hydroboration events then result in the formation of fully defluorinated alkene **D**, which undergoes a final hydroboration with usual linear selectivity to give the final product. The reaction entries in Fig. 2b with *gem*-difluoroalkenes as substrates strongly support **B** as an intermediate.

In conclusion, a straightforward, exhaustive defluorination-monoborylation has been developed that “zips off” all fluorine atoms from linear perfluoroalkyls attached to an alkene function and transforms them into linear alkyl pinacolboranes. The reaction is concluded to proceed via an iterative hydroboration/*anti*-defluoroborylation mechanism with *gem*-difluoroalkenes as intermediates that by themselves undergo the polydefluorinative borylation as well. The reaction is a unique example of a PFAS defluorination reaction that keeps the carbon framework intact, gives high yields even for longer perfluoroalkyl chains, and installs a new functional group. The newly introduced terminal boryl group enables further utilization in synthesis, rendering this work the first example of the conversion of environmentally problematic long-chain PFAS into useful synthesis building blocks. While this addresses the problem of a selective chemical conversion of certain PFAS, the method is likely transferable to other perhalogenated linear alkyls that present environmental hazards as well.

## Methods

### General procedure for the polydefluorination-monoborylation of polyfluoroalkylated alkenes 1

To a flame-dried and argon-filled Schlenk tube were added the substrate containing a C_m_F_n_ chain (0.5 mmol, 1.0 equiv), LiOMe (*n* + 1 equiv), 2-MeTHF (2.0 M with respect to HBpin) and HBpin (3*n* + 1 equiv). Occasionally, slight gas formation was observed upon the addition of HBpin. The tube was sealed, and the reaction mixture was stirred at 85 °C for 18 h. The reaction was allowed to cool down to room temperature. The reaction was quenched with MeOH (0.5 mL, *CAUTION*: hydrogen gas evolution!), diluted with Et_2_O (10 mL). The mixture was filtered over Celite^®^, the filter was washed with Et_2_O (20 mL), and the solvent was removed under reduced pressure. The crude reaction mixture was purified by column chromatography on silica gel to give the product. The linear/branched ratio of the product was determined by ^1^H NMR.

### General procedure for the polydefluorination-monoborylation of difluoro alkenes 3

To a flame-dried and argon-filled Schlenk tube was added the substrate (0.5 mmol, 1.0 equiv), LiOMe (75.9 mg, 2.0 mmol, 4 equiv), 2-MeTHF (0.2 M, 2.5 mL) and HBpin (0.73 mL, 5.0 mmol, 10 equiv). Occasionally, slight gas formation was observed upon the addition of HBpin. The tube was sealed, and the reaction mixture was stirred at 85 °C for 18 h. The reaction was allowed to cool down to room temperature. The reaction was quenched with MeOH (0.5 mL, *CAUTION*: hydrogen gas evolution!), diluted with Et_2_O (10 mL). The mixture was filtered over Celite^®^, the filter was washed with Et_2_O (20 mL) and the solvent was removed under reduced pressure. The crude reaction mixture was purified by column chromatography on silica gel to give the product. The linear/branched ratio of the product was determined by ^1^H NMR.

## Supplementary information

Supplementary Information

Transparent Peer Review file

## Source data

Source Data

## Data Availability

The experimental procedures, further optimization and control studies, analytical data, computational details, and spectra supporting the findings of this work are available in the Supplementary Information. Raw NMR data and computational output files generated in this study have been deposited in the Zenodo database under the accession code 18460740 and are available free of charge at https://doi.org/10.5281/zenodo.18460740. The data for the time-course plots and the coordinates of the computed structures are also provided as Source Data with this paper. All data is available from the corresponding author upon request. Source data are provided with this paper.
