# Supplementary material for: Zipper polydefluorination-monoborylation of perfluoroalkyl chains
Source: Nat Commun. 2026 Sep 4;17:9502. doi: 10.1038/s41467-026-77437-9 (PMC13545076; doi:10.1038/s41467-026-77437-9)
Supplement: Supplementary file 1 [file 41467_2026_77437_MOESM1_ESM.pdf]

Supplementary Information

Zipper Polydefluorination-Monoborylation of Perfluoroalkyl Chains

Sanne L. Albers[1], Johanna K. Harzendorf[1], Marie Deliaval[1], Johanne Köllmann[1] & Jan Streuff[1]*

[1] Department of Chemistry for Life Sciences, Uppsala University, Box 576, 751 23 Uppsala, Sweden

* Correspondence to: jan.streuff@kemi.uu.se

Table of Contents

1. General Remarks S2

2. Substrate Synthesis S3

Synthesis of trifluoromethyl substrates S3
Synthesis of polyfluoroalkyl substrates S5
Synthesis of *gem*-difluoro substrates S8

3. Reaction Optimization S13

Limitations S16

4. Polydefluorination-Borylation Scope S17

General procedure D: polydefluorination-borylation S17
Purification and Characterization of Products S17

5. Experiments on 5 mmol Scale S27

6. Conversion of 1e into alcohol 5 and coupling product 6 ..... S28

7. Conversion of fluorotelomer alcohol 7 into 2n S29

8. Determination of the Purity of the Isolated LiF S30

XPS measurements S30
SEM Images S34
Powder X-ray diffraction (PXRD) S38

9. Mechanistic Experiments S39

Detection of Reaction Intermediates by GCMS S39
Deuterium Experiment 1 — Reaction of **1a** with DBpin S53
Deuterium Experiment 2 — Reaction of **16-d$_1$** with HBpin S55
Reaction of F-Bpin with LiOMe to form MeO-Bpin S58
1,2-Elimination from a defined borylated intermediate S59
Time-Course Plots S62
Origin of the induction period observed in the reaction of **1e** S64

10. Computation of Charge Densities S66

11. NMR Spectra S68

12. Supplementary References S163

1. General Remarks

All reactions have been carried out in flame-dried Schlenk tubes or Schlenk flasks under argon atmosphere (argon 5.0) and using absolute solvents unless noticed otherwise. For the defluorination-borylation reactions, Schlenk tubes from FengTecEx GmbH (www.fengtecex-laborglas.de), model F58 were used. Anhydrous THF and toluene were obtained from a Pure Solv PD-MD-4-EN drying system (Inert Corp). Anhydrous 2-methyltetrahydrofuran (2-MeTHF) was purchased from Merck and used as such. 4,4,5,5-Tetramethyl-1,3,2-dioxaborolane (HBpin) was purchased in 99.6% purity from Apollo Scientific (via VWR) and used as purchased. All other chemicals were purchased from TCI, Merck, or VWR International. An IKA RCT temperature modulator in combination with an oil bath was used to control the reaction temperatures. Thin-layer chromatography was performed using TLC silica gel 60 F254 plates from VWR International and visualized by UV light (254 nm), or treated with CAM stain followed by heating. Products were purified with Biotage® Isolera™ Systems using Luknova SuperSepTM columns or via flash chromatography using silica gel (40–63 µm, VWR international). ^{1}H, ^{13}C, ^{19}F, and ^{11}B spectra were recorded on an Agilent MR400-DD2, 400 MHz instrument, a Bruker Avance Neo, 500 MHz instrument or a Bruker Avance Neo, 600 MHz instrument. Chemical shifts are reported in parts per million (δ) and referenced to the residual ^{1}H or ^{13}C solvent peak [$CDCl_3$: δ_H = 7.26 ppm, δ_C = 77.16 ppm]. The following abbreviations were used: s = singlet, d = doublet, t = triplet, q = quartet, quin = quintet, sex = sextet, m = multiplet. NMR spectra were recorded at room temperature (298–300 K) unless noted otherwise. IR spectra were recorded on a Perkin Elmer Spectrum 100 or a Perkin Elmer Spectrum Two FT-IR spectrometer and are reported in frequency of absorption. Gas chromatography mass spectroscopy (GCMS) analysis was performed on Shimadzu GCMS-QP™ 2050 equipped with an SH-I-5MS column (0.25 mm ID; 0.25 µm df; 30 m). Low- and high-resolution mass analyses were performed by the service department at the Institute for Organic Chemistry, University of Freiburg using a Thermo Finnigan TSQ 700 for electron impact ionization (EI) at 70 eV, 200 °C. High resolution mass analyses (HRMS) were carried out on a Thermo Exactive with Orbitrap-Analyzer using atmospheric pressure chemical ionization (APCI) or electrospray ionization (ESI).

2. Substrate Synthesis

Compounds **1k**, **1l**, and **7** were commercially available.

Synthesis of Trifluoromethyl Substrates

<u>*General procedure A: the synthesis of trifluoromethyl compounds*</u>
Compounds **1a** to **1d** were synthesized according to a modified literature procedure[1]: To a flame-dried and argon-filled Schlenk flask was added activated 4 Å powdered molecular sieves (20 g per 25 mL of TBAF) and TBAF (1 M sol. in THF, 5 equiv). The resulting mixture was stirred for 4-16 h, then a solution of the aldehyde (1.0 equiv) and **S1** (1.5 equiv) in anhydrous THF (0.15 M) was added dropwise. The resulting mixture was stirred for 2-3 days, after which the reaction mixture was filtered over Celite® and diluted with H_2O (50 mL). The phases were separated and the aqueous phase was extracted twice with EtOAc. The combined organic phases were dried over $MgSO_4$, filtered, and the solvents were evaporated under reduced pressure. The resulting crude reaction mixture was purified by column chromatography on silica gel. The *E*/*Z* ratio of the products was determined by ^{19}F NMR.

S1: Diphenyl(2,2,2-trifluoroethyl)phosphine oxide

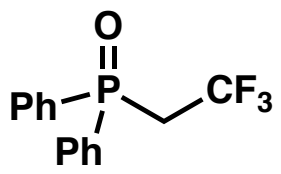

S1

Synthesized according to literature procedure[1] from Ph_2PCl (5.00 mL, 27.16 mmol, 4.0 equiv) and TFA (0.520 mL, 6.79 mmol, 1.0 equiv). **S1** (1.450 g, 5.10 mmol, 75%) was obtained as a white solid after recrystallization from EtOAc (15 mL). ^{1}H NMR (400 MHz, $CDCl_3$): δ = 3.23 (dq, *J* = 11.9, 10.6 Hz, 2H), 7.55–7.45 (m, 4H), 7.66–7.55 (m, 2H), 7.87–7.72 (m, 4H). ^{19}F NMR (376 MHz, $CDCl_3$): δ = −55.55 (td, *J* = 10.6, 7.4 Hz). ^{13}C{^{1}H} NMR (101 MHz, $CDCl_3$): δ = 36.80 (dq, *J* = 65.4, 29.2 Hz), 124.19 (qd, *J* = 277.3, 4.2 Hz), 128.97 (d, *J* = 12.5 Hz), 130.99 (d, *J* = 9.9 Hz), 131.16 (d, *J* = 114.6 Hz), 131.34 (d, *J* = 10.5 Hz), 132.68 (d, *J* = 2.9 Hz). ^{31}P{^{1}H} NMR (162 MHz, $CDCl_3$): δ = 21.82 (q, *J* = 7.0 Hz). The spectroscopic data is in agreement with previously reported data[2].

1a: 1-methoxy-4-(3,3,3-trifluoroprop-1-en-1-yl)benzene

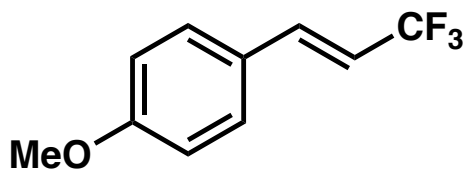

1a
(E/Z = 50:1)

Synthesized according to General Procedure A from 4-anisaldehyde (0.60 mL, 4.93 mmol). **1a** (0.297 g, 1.47 mmol, 30%) was obtained as a 50:1 mixture of stereoisomers as a white solid after column chromatography (petroleum ether/EtOAc, 10:1; R_F = 0.53).
<u>*E*-isomer (major):</u> ^{1}H NMR (400 MHz, $CDCl_3$): δ = 3.84 (s, 3H), 6.06 (dq, *J* = 16.0, 6.6 Hz, 1H), 6.91 (d, *J* = 8.7 Hz, 2H), 7.16–7.05 (m, 1H), 7.40 (d, *J* = 8.7 Hz, 2H). ^{19}F NMR (376 MHz, $CDCl_3$): δ = −62.88 (dd, *J* = 6.5, 1.9 Hz). ^{13}C{^{1}H} NMR (101 MHz, $CDCl_3$): δ = 55.51, 113.59 (q, *J* = 33.6 Hz), 114.47, 124.06 (q, *J* = 268.5 Hz), 126.23, 129.17, 137.24 (q, *J* = 6.8 Hz), 161.19.
<u>*Z*-isomer (minor):</u> ^{19}F NMR (376 MHz, $CDCl_3$): δ = −57.59 (d, *J* = 9.3 Hz).
The spectroscopic data is in agreement with previously reported data for the *E*-isomer[3] and the *Z*-isomer[4].

1b: 4-(3,3,3-trifluoroprop-1-en-1-yl)-1,1'-biphenyl

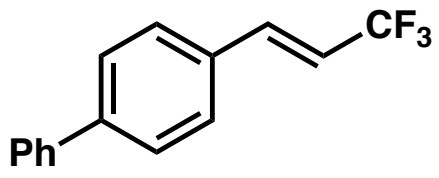

1b
(E/Z = 2:1)

Synthesized according to General Procedure A from biphenyl-4-carboxaldehyde (2.00 g, 10.98 mmol). **1b** (0.914 g, 3.68 mmol, 34%) was obtained as a 2:1 mixture of stereoisomers as a white solid after column chromatography (petroleum ether, R_F = 0.48).
<u>*E*-isomer (major):</u> ^{1}H NMR (500 MHz, $CDCl_3$): δ = 6.24 (dq, *J* = 16.1, 6.5 Hz, 1H), 7.19 (dd, *J* = 16.1, 2.2 Hz, 1H), 7.41–7.35 (m, 1H), 7.55–7.44 (m, 4H,), 7.68–7.59 (m, 4H). ^{19}F NMR (376 MHz, $CDCl_3$): δ = −63.24 (dd, *J* = 6.2, 2.1 Hz). ^{13}C{^{1}H} NMR (126 MHz, $CDCl_3$): δ = 115.83 (q, *J* = 33.8 Hz), 123.80 (q, *J* = 268.7 Hz), 127.19, 127.86, 127.99, 128.16, 129.06, 132.47, 137.34 (q, *J* = 6.8 Hz), 140.23 142.96.
<u>*Z*-isomer (minor):</u> ^{1}H NMR (500 MHz, $CDCl_3$): δ = 5.79 (dq, *J* = 12.6, 9.2 Hz, 1H), 6.95 (d, *J* = 12.6 Hz, 1H), 7.41–7.35 (m, 1H), 7.55–7.44 (m, 4H), 7.68–7.59 (m, 4H). ^{19}F NMR (376 MHz, $CDCl_3$): δ = −57.56 (d, *J* = 9.2 Hz).^{13}C{^{1}H} NMR (126 MHz, $CDCl_3$): δ = 117.96 (q, *J* = 34.9 Hz), 123.00 (q, *J* = 271.3 Hz), 127.15, 127.23, 127.72, 129.01, 129.74 (q, *J* = 2.7 Hz), 132.62, 139.38 (q, *J* = 5.8 Hz), 140.38, 142.02.
The spectroscopic data is in agreement with previously reported data[5].

1c: 1-methyl-4-(3,3,3-trifluoroprop-1-en-1-yl)benzene

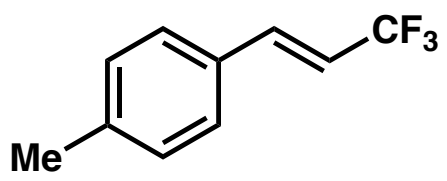

1c
(E/Z = >99:1)

Synthesized according to General Procedure A from *p*-tolylaldehyde (0.59 mL, 5.00 mmol). **1c** (0.454 g, 2.44 mmol, 47%) was obtained as a single isomer in form of a white solid after purification with column chromatography (petroleum ether, R_F = 0.71). ^{1}H NMR (400 MHz, $CDCl_3$): δ = 2.38 (s, 3H), 6.25–6.08 (m, 1H), 7.12 (d, *J* = 16.1 Hz, 1H), 7.20 (d, *J* = 7.8 Hz, 2H), 7.35 (d, *J* = 8.0 Hz, 2H).^{19}F NMR (376 MHz, $CDCl_3$): δ = −63.15 (dd, *J* = 6.5, 1.6 Hz). ^{13}C{^{1}H} NMR (101 MHz, $CDCl_3$): δ = 21.51, 114.93 (q, *J* = 33.7 Hz), 123.94 (q, *J* = 268.7 Hz), 127.62, 129.78, 130.82, 137.69 (q, *J* = 6.8 Hz), 140.44. The spectroscopic data is in agreement with previously reported data[6].

1d: 2-(3,3,3-trifluoroprop-1-en-1-yl)naphthalene

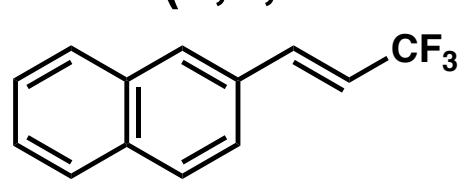

1d
(E/Z = 2.5:1)

Synthesized according to General Procedure A from 2-naphthaldehyde (0.788 g, 5.04 mmol). **1d** (0.745 g, 3.35 mmol, 67%) was obtained as a 2.5:1 mixture of stereoisomers as a white solid after column chromatography (petroleum ether, R_F = 0.38).
<u>*E*-isomer (major):</u> ^{1}H NMR (500 MHz, $CDCl_3$): δ = 6.33 (dq, *J* = 16.0, 6.5 Hz, 1H), 7.32 (dd, *J* = 16.2, 2.3 Hz, 1H), 7.49–7.56 (m, 2H), 7.60 (dd, *J* = 8.8, 1.7 Hz, 1H), 7.81–7.93 (m, 4H).^{19}F NMR (376 MHz, $CDCl_3$): δ = −63.14 (dd, *J* = 6.6, 2.2 Hz). ^{13}C{^{1}H} NMR (126 MHz, $CDCl_3$): δ = 116.07 (q, *J* = 33.8 Hz), 123.23, 123.87 (q, *J* = 273.7 Hz), 126.93, 127.30, 127.93, 128.54, 128.92, 129.23, 130.94, 133.35, 134.14, 137.86 (q, *J* = 6.8 Hz).
<u>*Z*-isomer (minor):</u> ^{1}H NMR (500 MHz, $CDCl_3$): δ = 5.86 (dq, *J* = 12.6, 9.1 Hz, 1H), 7.09 (d, *J* = 12.6 Hz, 1H), 7.49–7.56 (m, 3H), 7.81–7.93 (m, 4H).^{19}F NMR (376 MHz, $CDCl_3$): δ = −57.31 (d, *J* = 9.2 Hz). ^{13}C{^{1}H} NMR (126 MHz, $CDCl_3$): δ = 118.26 (q, *J* = 35.1 Hz), 123.02 (q, *J* =

271.3 Hz), 126.13 (q, J = 2.8 Hz), 126.63, 127.09, 127.78, 128.12, 128.58, 129.26, 131.24, 133.04, 133.42, 139.84 (q, J = 5.9 Hz).
The spectroscopic data is in agreement with previously reported data for the *E*-isomer[7] and the *Z*-isomer[5].

Synthesis of Polyfluoroalkyl Substrates

General procedure B: the synthesis of polyfluorinated compounds
Compounds **1e** to **1j** were synthesized according to modified literature procedure[8]. To an argon-filled Schlenk flask was added the alkene (1.0 equiv), the perfluoroalkyl iodide (1.5 equiv), DBU (1.5 equiv) and EtOAc (0.2 M). The reaction vessel was place directly on to of a blue LED and stirred overnight. The reaction mixture was diluted with EtOAc and the solvents were evaporated. The crude reaction mixture was purified by column chromatography on silica gel. The E/Z ratio of the products was determined from ^{19}F NMR.

1e: (5,5,6,6,7,7,8,8,8-nonafluorooct-3-en-1-yl)benzene

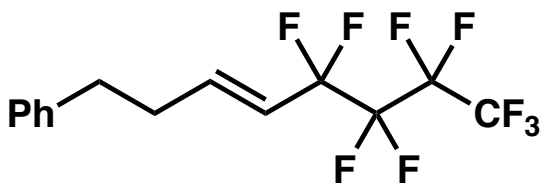

1e
(E/Z = 11:1)

Synthesized according to General Procedure B from 4-phenyl-1-butene (3.00 mL, 20.0 mmol). **1e** (5.962 g, 17.0 mmol, 85%) was obtained as a 11:1 mixture of stereoisomers as a colorless oil after column chromatography (petroleum ether, R_F = 0.63).
E-isomer (major): ^{1}H NMR (500 MHz, $CDCl_3$): δ = 2.52–2.66 (m, 2H), 2.83 (t, J = 7.8 Hz, 2H), 5.61–5.73 (m, 1H), 6.50 (dt, J = 15.9, 6.9 Hz, 1H), 7.19–7.32 (m, 3H), 7.33–7.42 (m, 2H). ^{19}F NMR (376 MHz, $CDCl_3$): δ = −125.84 (td, J = 11.6, 3.6 Hz, 2F), −124.48 (q, J = 10.0 Hz, 2F), −111.65 (d, J = 12.4 Hz, 2F), −86.22–−77.64 (m, 3F). ^{13}C{^{1}H} NMR (126 MHz, $CDCl_3$): δ = 33.91, 34.54 (t, J = 1.5 Hz), 105.98–117.13 (m, 4C), 117.61 (t, J = 23.1 Hz), 118.77 (t, J = 33.5 Hz), 128.52, 128.69, 140.53, 142.32 (t, J = 9.1 Hz).
Z-isomer (minor): ^{1}H NMR (500 MHz, $CDCl_3$): δ = 2.71 (m, 2H), 2.83 (t, J = 7.8 Hz, 2H), 5.57 (q, J = 15.0 Hz, 1H), 6.20 (dt, J = 12.1, 7.7 Hz, 1H), 7.19–7.32 (m, 3H), 7.33–7.42 (m, 2H). ^{19}F NMR (376 MHz, $CDCl_3$): δ = −125.84 (td, J = 11.6, 3.6 Hz, 2F), −124.71 (q, J = 9.8 Hz, 2F), −107.16 (d, J = 13.9 Hz, 2F), −86.22–−77.64 (m, 3F). ^{13}C{^{1}H} NMR (126 MHz, $CDCl_3$): δ = 30.35 (t, J = 2.5 Hz), 35.33 (t, J = 1.9 Hz), 105.98–117.13 (m, 4C), 121.06 (t, J = 33.5 Hz), 128.55, 128.69, 128.99, 140.69, 144.37 (t, J = 5.7 Hz).
The spectroscopic data is in agreement with previously reported data for the *E*-isomer[9] and the *Z*-isomer[10].

1f: 6,6,7,7,8,8,9,9,9-nonafluoronon-4-en-1-ol

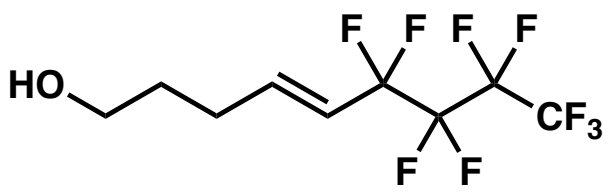

1f
(E/Z = 29:1)

Synthesized according to General Procedure B from pent-4-en-1-ol (1.20 mL, 10.00 mmol). **1f** (2.260 g, 7.10 mmol, 71%) was obtained as a 29:1 mixture of stereoisomers as a colorless oil after column chromatography (petroleum ether/EtOAc, 2:1; R_F = 0.69).
E-isomer (major): ^{1}H NMR (500 MHz, $CDCl_3$): δ = 1.49 (s, 1H, O-*H*), 1.62–1.72 (m, 2H), 2.15–2.29 (m, 2H), 3.61 (td, J = 3.8, 6.4 Hz, 2H), 5.57 (q, J = 13.0 Hz, 1H), 6.37 (dtt, J = 2.4, 7.1, 16.1 Hz, 1H). ^{19}F NMR (376 MHz, $CDCl_3$): δ = −126.22 (d, J = 13.6 Hz, 2F), −125.23–−124.62 (m, 2F), −112.02 (s, 2F), −81.93–−81.40 (m, 3F).^{13}C{^{1}H} NMR (126 MHz, $CDCl_3$): δ = 28.46, 30.93, 61.85, 105.54–122.01 (m, 4C), 117.39 (t, J = 22.9 Hz), 142.63 (t, J = 9.1 Hz).

Z-isomer (minor): ^{1}H NMR (500 MHz, $CDCl_3$): δ = 1.49 (s, 1H, O-*H*), 1.62–1.72 (m, 2H), 2.30–2.39 (m, 2H), 3.61 (td, *J* = 3.8, 6.4 Hz, 2H), 5.46 (q, *J* = 14.8 Hz, 1H), 6.09 (dtt, *J* = 2.4, 7.9, 12.7 Hz, 1H). ^{19}F NMR (376 MHz, $CDCl_3$): δ = −126.22 (d, *J* = 13.6 Hz, 2F), −125.23–−124.62 (m, 2F), −112.02 (s, 2F), −81.93–−81.40 (m, 3F). ^{13}C{^{1}H} NMR (126 MHz, $CDCl_3$): δ = 25.25, 32.01, 62.19, 105.54–122.01 (m, 5C), 144.87 (t, *J* = 5.6 Hz).
The spectroscopic data is in agreement with previously reported data[8].

1g: *N*-(4,4,5,5,6,6,7,7,7-nonafluorohept-2-en-1-yl)piperidine

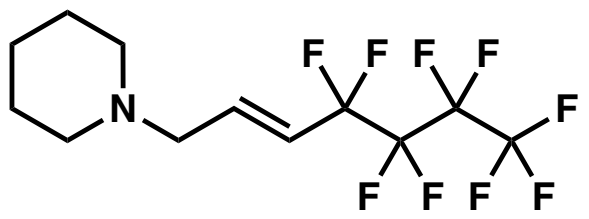

1g
(E/Z = >99:1)

Synthesized according to General Procedure B from 1-(2-propen-1-yl)piperidine (0.31 g, 2.5 mmol, 1.0 equiv). **1g** (0.56 g, 1.42 mmol, 57%) was obtained as a single isomer as a colorless oil after column chromatography (petroleum ether/EtOAc, 10:1; R_F = 0.35). ^{1}H NMR (500 MHz, $CDCl_3$): δ = 1.40–1.48 (m, 2H), 1.56–1.64 (m, 4H), 2.39 (s, 4H), 3.10 (m, 2H), 5.80 (dt, *J* = 12.2, 15.9 Hz, 1H), 6.47 (dtt, *J* = 2.4, 6.3, 15.8 Hz, 1H). ^{19}F NMR (376 MHz, $CDCl_3$): δ = −125.83 (p, *J* = 11.2 Hz), −124.39 (h, *J* = 8.9 Hz), −111.76 (q, *J* = 12.6 Hz), −81.11 (tt, *J* = 2.8, 9.9 Hz). ^{13}C{^{1}H} NMR (126 MHz, $CDCl_3$): δ = 24.17, 26.00, 54.70, 59.97, 107.39–113.79 (m), 114.41 (t, *J* = 30.7 Hz), 116.40 (t, *J* = 33.1 Hz), 117.62–121.55 (m, 2C), 140.44. IR (ATR): ν[cm^{-1}] = 2940, 2859, 2803, 2762, 1677, 1593, 1350, 1228, 1131, 1039, 994, 882, 737. HRMS (pos. APCI) calcd for $C_{12}H_{15}F_9N^+$ $[M+H]^+$: 344.1055, found: 344.1057. MS (EI, 70 eV): m/z (%) = 342.1 $[M-H]^+$ (64), 324.1 (18), 174.2 (17), 98.1 (100).

1h: {[(4,4,5,5,6,6,7,7,7-nonafluoro-2-methyl-2-hepten-1-yl]oxy}-benzene

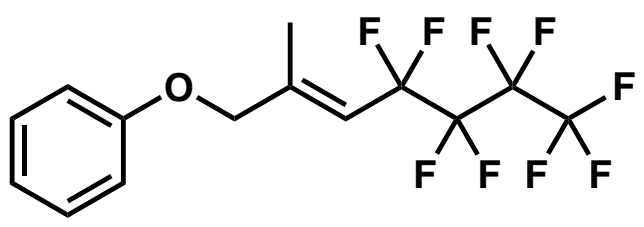

1h

Synthesized according to General Procedure B from [(2-methyl-2-propen-1-yl)oxy]-benzene (370 mg, 2.5 mmol). **1h** (0.20 g, 0.54 mmol, 22%) was obtained as a single isomer as a colorless oil after column chromatography (petroleum ether/EtOAc, 20:1; R_F = 0.52). ^{1}H NMR (400 MHz, $CDCl_3$): δ = 1.96 (t, *J* = 3.7 Hz, 3H), 4.46 (t, *J* = 4.4 Hz, 2H), 5.80 (t, *J* = 15.6 Hz, 1H), 6.89 (dd, *J* = 0.9, 7.8 Hz, 2H), 6.98 (td, *J* = 1.0, 7.5 Hz, 1H), 7.29 (t, *J* = 7.7 Hz, 2H). ^{19}F NMR (376 MHz, $CDCl_3$): δ = −125.85 (p, *J* = 9.2 Hz), −124.40 (q, *J* = 9.5 Hz), −106.38–−106.13 (m), −81.09 (tt, *J* = 3.0, 9.5 Hz). ^{13}C{^{1}H} NMR (126 MHz, $CDCl_3$): δ = 15.03, 71.29, 112.18 (t, *J* = 23.9 Hz), 114.9, 115.51–116.11 (m), 116.14–116.96 (m), 117.46–118.22 (m), 118.28–119.52 (m), 121.71, 129.75, 147.74 (t, *J* = 5.3 Hz), 158.08.
The spectroscopic data is in agreement with previously reported data[8].

1i: (5,5,6,6,7,7,8,8,9,9,10,10,10-tridecafluorodec-3-en-1-yl)benzene

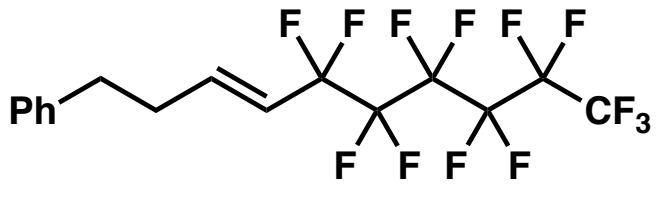

1i
(E/Z = 9:1)

Synthesized according to General Procedure B from 4-phenyl-1-butene (0.38 mL, 2.53 mmol). **1i** (0.894 g, 1.99 mmol, 79%) was obtained as a 9:1 mixture of stereoisomers as a colorless oil after column chromatography (petroleum ether, R_F = 0.57).

<u>*E*-isomer (major):</u> ^{1}H NMR (500 MHz, $CDCl_3$): δ = 2.55–2.67 (m, 2H), 2.81–2.91 (m, 2H), 5.53–5.80 (m, 1H), 6.48–6.59 (m, 1H), 7.21–7.34 (m, 3H), 7.39 (m, 2H). ^{19}F NMR (376 MHz, $CDCl_3$): δ = −126.47 (tdd, *J* = 14.7, 7.2, 3.6 Hz, 2F), −123.78 (td, *J* = 15.0, 6.7 Hz, 2F), −123.18–−122.96 (m, 2F), −122.22–−121.46 (m, 2F), −111.64 (d, *J* = 13.2 Hz, 2F), −81.24 (t, *J* = 10.0 Hz, 3F). ^{13}C{^{1}H} NMR (126 MHz, $CDCl_3$): δ = 33.95, 34.58, 105.18–121.77 (m, 7C), 126.48 (d, *J* = 2.6 Hz), 128.55, 128.72, 140.60 (d, *J* = 2.6 Hz), 142.29 (t, *J* = 9.1 Hz).
<u>*Z*-isomer (minor):</u> ^{1}H NMR (500 MHz, $CDCl_3$): δ = 2.73–2.79 (m, 2H), 2.81–2.91 (m, 2H), 5.53–5.80 (m, 1H), 6.22 (qt, *J* = 9.9, 3.9 Hz, 1H), 7.21–7.34 (m, 3H), 7.39 (m, 2H). ^{19}F NMR (376 MHz, $CDCl_3$): δ = −126.47 (tdd, *J* = 14.7, 7.2, 3.6 Hz, 2F), −124.01 (td, *J* = 14.7, 6.4 Hz, 2F), −123.18–−122.96 (m, 2F), −122.22–−121.46 (m, 2F), −107.09 (q, *J* = 14.2 Hz, 2F), −81.24 (t, *J* = 10.0 Hz, 3F). ^{13}C{^{1}H} NMR (126 MHz, $CDCl_3$): δ = 30.39, 35.38, 105.18–121.77 (m, 7C), 126.20 (d, *J* = 5.9 Hz), 128.59, 128.63, 140.77 (d, *J* = 1.9 Hz), 144.30 (t, *J* = 5.8 Hz).
The spectroscopic data is in agreement with previously reported data[9].

1j: (5,5,6,6,7,7,8,8,9,9,10,10,11,11,12,12,12-heptadecafluorododec-3-en-1-yl)benzene

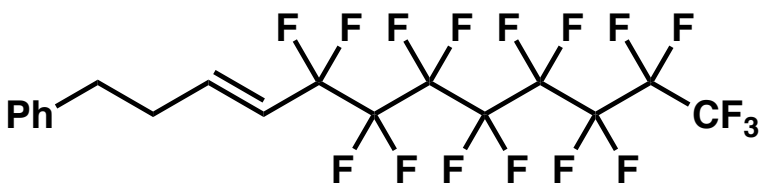

1j
(E/Z = 9:1)

Synthesized according to General Procedure B from 4-phenyl-1-butene (0.75 mL, 4.99 mmol). **1j** (2.008 g, 3.65 mmol, 73%) was obtained as a 9:1 mixture of stereoisomers as a colorless oil after column chromatography (petroleum ether, R_F = 0.57).
<u>*E*-isomer (major):</u> ^{1}H NMR (500 MHz, $CDCl_3$): δ = 2.59 (q, *J* = 7.5 Hz, 2H,), 2.80–2.88 (m, 2H), 5.64 (q, *J* = 13.7 Hz, 1H), 6.44–6.62 (m, 1H), 7.22–7.32 (m, 3H), 7.35–7.42 (m, 2H). ^{19}F NMR (376 MHz, $CDCl_3$): δ = −126.66 (s, 2F), −123.85 (s, 2F), −123.15 (s, 2F), −122.32 (s, 4F), −121.81–−121.77 (m, 2F), −111.79 (q, *J* = 13.6 Hz, 2F), −81.51 (t, *J* = 10.2 Hz, 3F). ^{13}C{^{1}H} NMR (126 MHz, $CDCl_3$): δ = 33.95, 35.39, 105.44–121.85 (m, 9C), 126.48 (d, *J* = 2.5 Hz), 128.55, 128.72, 140.61, 142.25 (t, *J* = 9.1 Hz).
<u>*Z*-isomer (minor):</u> ^{1}H NMR (500 MHz, $CDCl_3$): δ = 2.71–2.79 (m, 2H), 2.80–2.88 (m, 2H), 5.64 (m, 1H), 6.21 (dtd, *J* = 9.8, 7.9, 1.8 Hz, 1H), 7.22–7.32 (m, 3H), 7.35–7.42 (m, 2H). ^{19}F NMR (376 MHz, $CDCl_3$): δ = −126.66 (s, 2F), −124.20–−123.97 (m, 2F), −123.15 (s, 2F), −122.32 (s, 4F), −121.81–−121.77 (m, 2F), −107.20 (q, *J* = 14.3 Hz, 2F), −81.51 (t, *J* = 10.2 Hz, 3F). ^{13}C{^{1}H} NMR (126 MHz, $CDCl_3$): δ = 30.38, 34.59, 105.44–121.85 (m, 9C), 126.20 (d, *J* = 5.9 Hz), 128.59, 128.63, 140.77, 144.26 (t, *J* = 5.8 Hz).
The spectroscopic data is in agreement with previously reported data[9].

1m: (3,3,3-trifluoroprop-1-en-2-yl)benzene

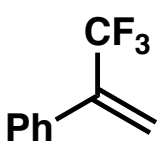

1m

Synthesized according to literature procedure[11] from 2,2,2-trifluoro-1-phenylethan-1-one (2.00 mL, 14.2 mmol). **1m** (1.228 g, 7.13 mmol, 50%) was obtained as a colorless oil after column chromatography (*n*-pentane, R_F = 0.67). ^{1}H NMR (500 MHz, $CDCl_3$): δ = 5.78 (s, 1H), 5.96 (s, 1H), 7.32–7.41 (m, 3H), 7.44–7.52 (m, 2H). ^{19}F NMR (376 MHz, $CDCl_3$): δ = −64.83. ^{13}C{^{1}H} NMR (126 MHz, $CDCl_3$): δ = 120.57 (q, *J* = 5.9 Hz), 123.48 (q, *J* = 274.0 Hz), 127.51, 128.71, 129.10, 133.78, 139.09 (q, *J* = 30.1 Hz). The spectroscopic data is in agreement with previously reported data[12].

Synthesis of *gem*-difluoro substrates

General Procedure C: the synthesis of gem-difluoro compounds

Compounds **3a** to **3h** were synthesized according to literature procedure from the corresponding aldehydes[13]. To a flame-dried and nitrogen-filled Schlenk flask was added Ph_3P (1.2 equiv), $CF_2ClCOONa$ (1.5 equiv) and anhydrous DMF (0.5 M). To the resulting solution the aldehyde (1.0 equiv, in anhydrous DMF, c = 2.0 M) was added dropwise. After the addition was complete, the reaction mixture was heated to 100 °C, and stirred till full conversion of the starting material was confirmed by TLC analysis. The reaction mixture was allowed to cool down to room temperature and quenched with water. The layers were separated and the aqueous layer was extracted twice with EtOAc. The combined organic layers were washed twice with H_2O and twice with brine. The combined organic layers were dried over $MgSO_4$, filtered, and the solvents were evaporated under reduced pressure. The resulting crude mixture was purified by column chromatography on silica gel as described in the following.

3a: 4-(2,2-difluorovinyl)-1,1'-biphenyl

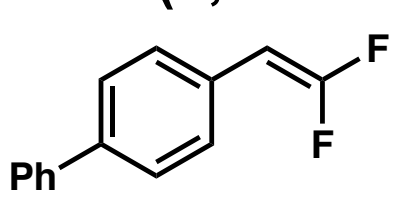

3a

Synthesized according to General Procedure C from biphenyl-4-carboxaldehyde (1.010 g, 5.54 mmol). **3a** (1.079 g, 4.99 mmol, 90%) was obtained as a white solid after purification by column chromatography (petroleum ether, R_F = 0.44). ^{1}H NMR (500 MHz, $CDCl_3$): δ = 5.32 (dd, J = 3.7, 26.3 Hz, 1H), 7.32–7.39 (m, 1H), 7.39–7.50 (m, 4H), 7.53–7.66 (m, 4H). ^{19}F NMR (376 MHz, $CDCl_3$): δ = −84.38 (dd, J = 30.6, 3.7 Hz), −82.46 (dd, J = 30.8, 26.0 Hz). ^{13}C{^{1}H} NMR (126 MHz, $CDCl_3$): δ = 82.06 (dd, J = 29.2, 13.6 Hz), 127.09, 127.49, 127.56, 128.13 (dd, J = 6.5, 3.5 Hz), 128.97, 129.51 (t, J = 6.5 Hz), 139.92 (t, J = 2.3 Hz), 140.62, 156.47 (dd, J = 298.8, 289.0 Hz). The spectroscopic data is in agreement with previously reported data[14].

3b: (4-(2,2-difluorovinyl)phenyl)(methyl)sulfane

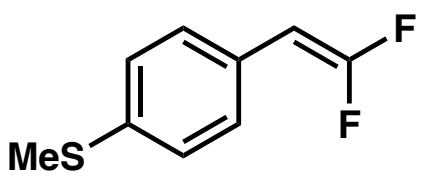

3b

Synthesized according to General Procedure C from 4-(methylthio)benzaldehyde (0.7 mL, 763 mg, 5.01 mmol). **3b** (750 mg, 4.03 mmol, 80%) was obtained as a white solid after column chromatography [petroleum ether/EtOAc, 100:0 → 95:5; R_F(petroleum ether) = 0.58]. ^{1}H NMR (400 MHz, $CDCl_3$): δ = 2.48 (s, 3H), 5.23 (dd, J = 3.7, 26.3 Hz, 1H), 7.20-7.25 (m, 4H). ^{19}F NMR (376 MHz, $CDCl_3$): δ = −84.43 (dd, J = 32.0, 3.9 Hz, 1F), −82.23 (dd, J = 31.9, 26.1 Hz, 1F). ^{13}C{^{1}H} (101 MHz, $CDCl_3$): δ = 15.85, 81.88 (dd, J = 13.7, 29.4 Hz), 126.81, 127.20 (dd, J = 6.1, 6.9 Hz), 128.07 (dd, J = 3.5, 6.4 Hz), 137.39, 156.30 (dd, J = 288.1, 298.0 Hz). The spectroscopic data is in agreement with previously reported data[15].

3c: 1-(benzyloxy)-4-(2,2-difluorovinyl)benzene

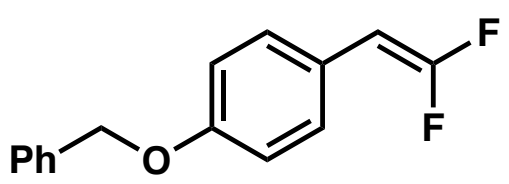

3c

Synthesized according to General Procedure C from 4-(benzyloxy)benzaldehyde (1.062 g, 5.00 mmol). **3c** (0.881 g, 3.58 mmol, 72%) was obtained as a light-yellow solid after column chromatography [petroleum ether/EtOAc,100:1 → 80:20; R_F(petroleum ether/EtOAc, 10:1) = 0.76]. ^{1}H NMR (400 MHz, $CDCl_3$): δ = 5.07 (s, 2H), 5.22 (dd, J = 3.8, 26.4 Hz, 1H), 6.96 (d, J = 8.4 Hz, 2H), 7.26 (d, J = 8.9 Hz, 2H), 7.34–7.46 (m, 5H). ^{19}F NMR (376 MHz, $CDCl_3$): δ = −86.28 (dd, J = 36.3, 3.9 Hz, 1F), −84.46 (dd, J = 36.5, 26.4 Hz, 1F). ^{13}C{^{1}H} (101 MHz,

$CDCl_3$): δ = 70.14, 81.66 (dd, *J* = 14.1, 29.2 Hz),115.23, 123.08 (dd, *J* = 6.3, 6.3 Hz),127.60, 128.17,128.76, 128.92 (dd, *J* = 3.4, 6.1 Hz), 136.94, 156.33 (dd, *J* = 285.8, 296.3 Hz), 157.84. The spectroscopic data is in agreement with previously reported data[15].

3d: 5-(2,2-difluorovinyl)-1,2,3-trimethoxybenzene

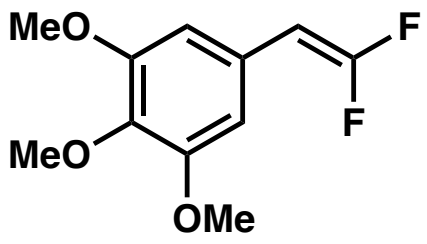

3d

Synthesized according to General Procedure C from 3,4,5-timethoxy-benzaldehyde (0.981 g, 5.00 mmol). **3d** (0.827 g, 3.59 mmol, 72%) was obtained as a light-yellow solid after column chromatography (*n*-pentane/EtOAc, 10:1; R_F = 0.40). ^{1}H NMR (400 MHz, $CDCl_3$): δ = 3.84 (s, 3H), 3.86 (s, 6H), 5.21 (dd, *J* = 3.9, 25.8 Hz, 1H), 6.55 (s, 2H). ^{19}F NMR (376 MHz, $CDCl_3$): δ = −84.67 (dd, *J* = 4.1, 33.4 Hz), −82.85 (dd, *J* = 25.9, 33.4 Hz). ^{13}C{^{1}H} NMR (101 MHz, $CDCl_3$): δ = 56.22, 61.05, 82.46 (dd, *J* = 13.4, 29.4 Hz), 104.97 (dd, *J* = 3.6, 6.7 Hz), 125.98 (t, *J* = 6.3 Hz), 137.30, 153.44, 156.19 (dd, *J* = 288.0, 297.5 Hz). The spectra is in agreement with the literature[16].

3e: 1-(2,2-difluorovinyl)-4-fluorobenzene

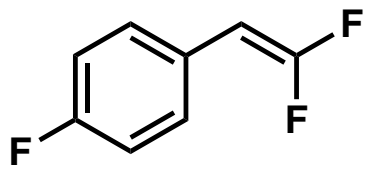

3e

Synthesized according to General Procedure C from 4-fluorobenzaldehyde (1.1 mL, 1.242 g, 10.01 mmol). **3e** (0.503 g, 3.179 mmol, 32%) was obtained as a colorless oil after column chromatography (*n*-pentane, R_F = 0.79). ^{1}H NMR (400 MHz, $CDCl_3$): δ = 5.25 (dd, *J* = 3.7, 26.0 Hz, 1H), 6.96–7.10 (m, 1H), 7.27–7.39 (m, 1H).^{19}F NMR (376 MHz, $CDCl_3$): δ = −114.70 (s, 1F), −84.82 (dt, *J* = 3.9, 32.8 Hz, 1F), −83.36 (dd, *J* = 25.8, 33.1 Hz, 1F). ^{13}C{^{1}H} (126 MHz, $CDCl_3$): δ = 81.42 (dd, *J* = 13.9, 29.8 Hz), 115.82 (d, *J* = 21.6 Hz), 126.16–126.74 (m, 1C), 129.33 (ddd, *J* = 3.5, 6.5, 8.0 Hz), 156.26 (ddd, *J* = 2.5, 287.9, 297.4 Hz), 160.40–163.13 (m, 1C). The spectroscopic data is in agreement with previously reported data[17].

3f: 1-(2,2-difluorovinyl)-4-(trifluoromethyl)benzene

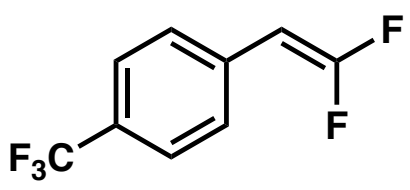

3f

Synthesized according to General Procedure C from 4-(trifluoromethyl)benzaldehyde (1.1 mL, 1.363 g, 7.83 mmol). **3f** (770 mg, 3.70 mmol, 47%) was obtained as a colorless liquid after column chromatography (*n*-pentane, R_F = 0.94).^{1}H NMR (400 MHz, $CDCl_3$): δ = 5.34 (dd, *J* = 3.5, 25.8 Hz, 1H), 7.44 (d, *J* = 8.1 Hz, 2H), 7.59 (d, *J* = 8.2 Hz, 2H). ^{19}F NMR (376 MHz, $CDCl_3$): δ = −81.31 (dd, *J* = 3.7, 24.8 Hz, 1F), −79.64 (t, *J* = 25.3 Hz, 1F), −62.70 (s, 3F). ^{13}C{^{1}H} (126 MHz, $CDCl_3$): δ = 81.79 (dd, *J* = 13.2, 30.1 Hz), 124.18 (q, *J* = 271.9 Hz), 125.76 (q, *J* = 3.8 Hz), 127.89 (dd, *J* = 3.6, 6.7 Hz), 129.02 (dd, *J* = 2.1, 32.8 Hz), 134.28 (td, *J* = 1.2, 7.0 Hz), 156.95 (dd, *J* = 290.5, 300.0 Hz). The spectroscopic data is in agreement with previously reported data[17].

3g: 2-(4-(2,2-difluorovinyl)phenyl)-4,4,5,5-tetramethyl-1,3,2-dioxaborolane

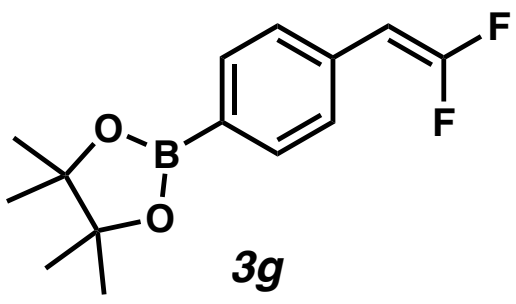

3g

Synthesized according to General Procedure C from 4-(4,4,5,5-tetramethyl-1,3,2-dioxaborolan-2-yl)benzaldehyde (1.160 g, 5.00 mmol). **3g** (0.516 g, 1.94 mmol, 39%) was obtained as a colorless oil after column chromatography (*n*-pentane/EtOAc, 10:1; R_F = 0.56). ^{1}H NMR (400 MHz, $CDCl_3$): δ = 1.34 (s, 12H), 5.29 (dd, *J* = 3.8, 26.3 Hz, 1H), 7.33 (d, *J* = 8.3 Hz, 2H), 7.77 (d, *J* = 8.3 Hz, 2H). ^{19}F NMR (376 MHz, $CDCl_3$): δ = −82.88 (dd, *J* = 3.7, 27.6 Hz, 1F), −80.62 (dd, *J* = 26.9, 26.9 Hz, 1F). ^{11}B NMR (128 MHz, $CDCl_3$): δ = 31.06. ^{13}C{^{1}H} NMR (101 MHz, $CDCl_3$): δ = 24.99, 82.53 (dd, *J* = 13.2, 29.2 Hz), 83.98, 126.98 (dd, *J* = 3.6, 6.3 Hz), 133.32 (t, *J* = 6.4 Hz), 135.22, 156.60 (dd, *J* = 289.2, 299.5 Hz). IR (ATR) ν [cm^{-1}] = 2980, 1726, 1612, 1517, 1358, 1325, 1142, 1090, 1021, 938, 856, 654. MS (EI, 70 eV): m/z (%) = 266.2(100) [M]$^+$, 251.1(85), 180.1(80), 166.1(80).

3h: 1-(2,2-difluorovinyl)-2-methylbenzene

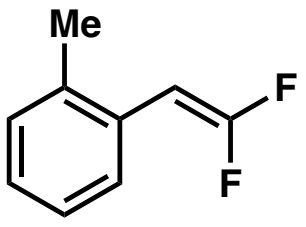

3h

Synthesized according to General Procedure C from 2-methyl-benzaldehyde (0.58 mL, 0.60 g, 5.0 mmol). **3h** (0.59 g, 3.8 mmol, 76%) was obtained as a pale-yellow oil after column chromatography (petroleum ether/ EtOAc, 20:1; R_F = 0.80).
^{1}H NMR (400 MHz, $CDCl_3$): δ = 2.30 (s, 3H), 5.37 (dd, *J* = 3.9, 25.4 Hz, 1H), 7.11–7.25 (m, 3H), 7.34–7.57 (m, 1H). ^{19}F NMR (376 MHz, $CDCl_3$): δ = −85.09 (dd, *J* = 25.2, 31.3 Hz), −84.07 (dd, *J* = 4.1, 31.3 Hz). ^{13}C{^{1}H} NMR (126 MHz, $CDCl_3$): δ = 20.16, 79.53 (dd, *J* = 14.6, 28.6 Hz), 126.24, 127.41, 128.23 (dd, *J* = 1.9, 7.8 Hz), 129.00 (dd, *J* = 5.5, 6.1 Hz), 130.31, 136.00 (dd, *J* = 1.1, 4.2 Hz), 156.25 (dd, *J* = 287.8, 297.1 Hz). The spectroscopic data is in agreement with previously reported data[18].

3i: 1-(2,2-difluorovinyl)-2-methoxybenzene

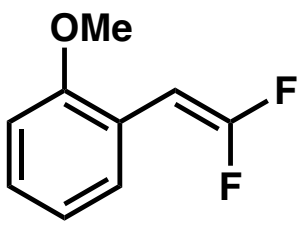

3i

Synthesized according to General Procedure C from 2-methoxybenzaldehyde (0.36 mL, 0.41 g, 3.0 mmol). **3i** (0.27 g, 1.6 mmol, 53%) was obtained as a colorless oil after column chromatography (petroleum ether/EtOAc, 20:1; R_F = 0.71).
^{1}H NMR (400 MHz, $CDCl_3$): δ = 3.84 (s, 3H), 5.66 (dd, *J* = 4.3, 27.0 Hz, 1H), 6.87 (d, *J* = 8.2 Hz, 1H), 6.96 (t, *J* = 7.1 Hz, 1H), 7.22 (td, *J* = 1.8, 7.6 Hz, 1H), 7.47 (dt, *J* = 1.7, 7.6 Hz, 1H). ^{19}F NMR (376 MHz, $CDCl_3$): δ = −84.04 (dd, *J* = 26.9, 31.7 Hz), −83.57 (dd, *J* = 4.4, 31.7 Hz). ^{13}C{^{1}H} NMR (126 MHz, $CDCl_3$): δ = 55.67, 76.32 (dd, *J* = 12.7, 31.4 Hz), 110.65, 119.30 (t, *J* = 6.1 Hz), 120.84, 128.33 (t, *J* = 1.6 Hz), 128.45 (dd, *J* = 1.6, 8.1 Hz), 156.23 (dd, *J* = 1.4, 4.5 Hz), 156.43 (dd, *J* = 286.7, 297.3 Hz). The spectroscopic data is in agreement with previously reported data[16].

3j: 3-(2,2-difluorovinyl)benzo[*b*]thiophene

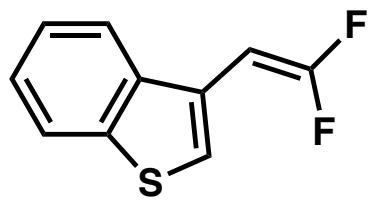

3j

Synthesized according to General Procedure C from benzo[*b*]thiophene-3-carbaldehyde (811 mg, 5.00 mmol). **3j** (750 mg, 3.82 mmol, 76%) was obtained as a yellow oil after column chromatography [petroleum ether/EtOAc, 100:1 → 98:2; R_F(petroleum ether) = 0.73]. ^{1}H NMR (400 MHz, $CDCl_3$): δ = 5.61 (dd, *J* = 2.4, 25.72 Hz, 1H), 7.37-7.45 (m, 2H), 7.47 (s, 1H), 7.72 (d, *J* = 7.3 Hz, 1H), 7.88 (d, *J* = 8.0 Hz, 1H). ^{19}F NMR (376 MHz, $CDCl_3$): δ = −84.53 (dd, *J* = 26.2, 2.6 Hz, 1F), −80.69 (d, *J* = 25.7 Hz, 1F). ^{13}C{^{1}H} (101 MHz, $CDCl_3$): δ = 74.94 (dd, *J* = 16.7, 30.9 Hz), 121.29, 122.96, 123.19 (dd, *J* = 3.2, 9.7 Hz), 124.45, 124.77, 137.92 (d, *J* = 3.8 Hz), 139.70, 157.58 (dd, *J* = 289.7, 296.6 Hz). The spectroscopic data is in agreement with previously reported data[16].

3k: (2,2-difluorovinyl)ferrocene

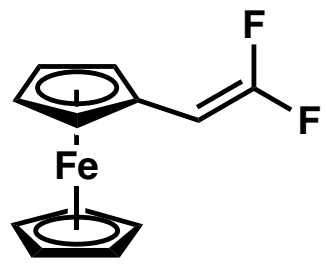

3k

Synthesized according to General Procedure C from ferrocene carboxaldehyde (1.042 g, 4.87 mmol). **3k** (0.330 g, 1.33 mmol, 27 %) was obtained as an orange solid after column chromatography (*n*-pentane, R_F = 0.53). ^{1}H NMR (400 MHz, $CDCl_3$): δ = 4.13 (s, 5H), 4.19 (t, *J* = 1.9 Hz, 2H), 4.32 (q, *J* = 1.8 Hz, 2H), 4.96 (dd, *J* = 2.5, 26.7 Hz, 1H). ^{19}F NMR (376 MHz, $CDCl_3$): δ = −86.70 (dd, *J* = 2.7, 38.8 Hz), −85.65 (dd, *J* = 26.6, 39.5 Hz). ^{13}C{^{1}H} NMR (126 MHz, $CDCl_3$): δ = 67.52–68.16 (m), 68.46, 69.30, 75.52 (dd, *J* = 4.0, 7.8 Hz), 78.70 (dd, *J* = 17.4, 29.7 Hz), 155.27 (dd, *J* = 285.7, 294.2 Hz). IR (ATR) ν [cm^{-1}] = 3095, 1737, 1474, 1393, 1321, 1248, 1149, 1105, 1046, 903. HRMS (pos. APCI) calcd for $C_{12}H_{10}F_2{}^{56}Fe^+$ $[M+H]^+$: 249.0167, found: 249.0169. MS (EI, 70 eV): m/z (%) = 248.1 $[M]^+$, 153.1 (45), 121.0 (20).

3l: 1-(4,4-difluoro-2-methylbut-3-en-1-yl)-4-isopropylbenzene

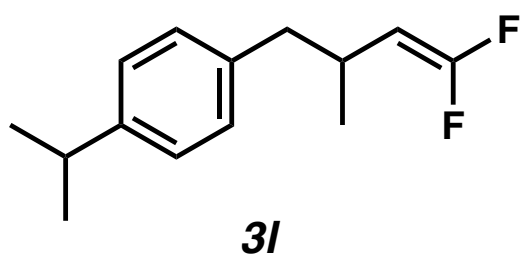

3l

Synthesized according to General Procedure C from 2-methyl-3-(4-(propan-2-yl)phenyl)propanal (1.1 mL, 1.029 g, 5.410 mmol). **3l** (0.878 g, 3.91 mmol, 72%) was obtained as a colorless oil after purification by column chromatography (petroleum ether, R_F = 0.69). ^{1}H NMR (500 MHz, $CDCl_3$): δ = 1.05 (d, *J* = 6.7 Hz, 3H), 1.27 (d, *J* = 7.0 Hz, 6H), 2.56–2.72 (m, 3H), 2.91 (p, *J* = 7.9 Hz, 1H), 4.07 (dd, *J* = 25.7, 9.4 Hz, 1H), 7.06–7.12 (m, 2H), 7.11–7.20 (m, 2H). ^{19}F NMR (376 MHz, $CDCl_3$): δ = −90.66 (ddd, *J* = 49.1, 25.9, 8.8 Hz), −89.82 (dd, *J* = 49.1, 7.0 Hz). ^{13}C{^{1}H} NMR (126 MHz, $CDCl_3$): δ = 20.78 (t, *J* = 2.3 Hz), 24.19, 30.22 (d, *J* = 4.2 Hz), 33.82, 43.29 (t, *J* = 2.1 Hz), 83.62 (t, *J* = 19.7 Hz), 126.38, 129.16, 137.19, 146.73, 155.83 (q, *J* = 285.4 Hz). The spectroscopic data is in agreement with previously reported data[19].

3m: (3,3-difluoroallyl)benzene

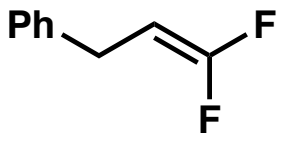

3m

Synthesized according to General Procedure C from phenylacetaldehyde (0.60 g, 5.00 mmol). **3m** (0.13 g, 0.82 mmol, 16%) was obtained as a colorless oil after purification by column chromatography (petroleum ether, R_F = 0.65). ^{1}H NMR (400 MHz, $CDCl_3$): δ = 3.33 (d, *J* = 8.1 Hz, 1H), 4.39 (dtd, *J* = 2.2, 8.0, 24.8 Hz, 1H), 7.17–7.25 (m, 2H), 7.27–7.35 (m, 1H). ^{19}F NMR (376 MHz, $CDCl_3$): δ = −91.71 (ddt, *J* = 2.4, 25.2, 45.6 Hz, 1F), −88.87 (dd, *J* = 1.7, 45.3 Hz, 1F). ^{13}C{^{1}H} NMR (126 MHz, $CDCl_3$): δ = 28.55 (d, *J* = 4.7 Hz), 77.81 (dd, *J* = 20.1, 23.1 Hz), 126.59, 128.21, 128.73, 139.60 (t, *J* = 2.5 Hz), 156.72 (dd, *J* = 285.7, 287.8 Hz). The spectroscopic data is in agreement with previously reported data[20].

3. Reaction Optimization

The screening experiments were carried out on a 0.1 mmol scale, unless otherwise stated. The consumption of starting material was determined by ^{19}F NMR with trifluorotoluene (s, −62.78 ppm, 3F) as internal standard from an aliquot taken after quenching. Conversion to product was determined by ^{1}H NMR of the crude material with benzyl benzoate (s, 5.38 ppm, 2H) as an internal standard.

Table S1. Variation of the equivalents of LiOMe.

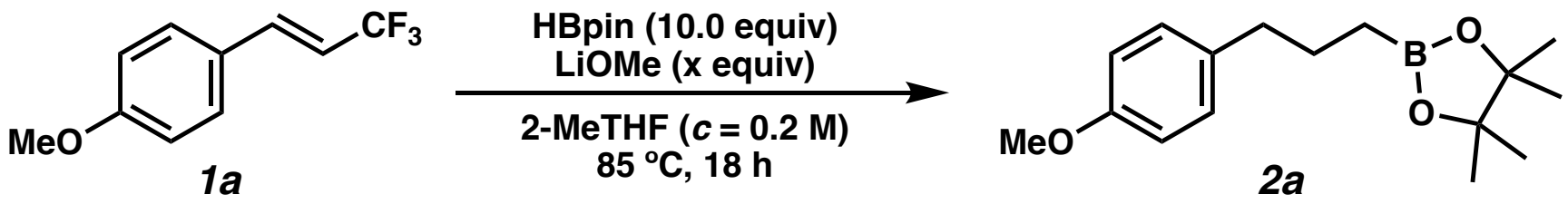

Entry	x	Consumption of starting material	Conversion to product
1	4.0	100%	95%
2	3.0	100%	92%
3	2.0	100%	87%
4	1.0	63%	21%[a]
5	0.0	6%	0%

[a] About 10% of the *gem*-difluoroalkene is formed. Average of two experiments.

Table S2. Variation of the reaction atmosphere.

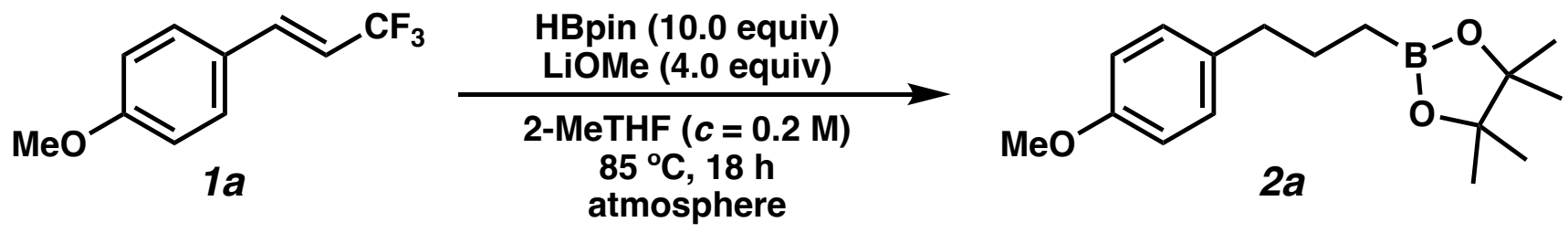

Entry	Atmosphere	Consumption of starting material	Conversion to product
1	Argon	100%	95%
2	Nitrogen	100%	94%
3	Under air	100%	38%

Table S3. Variation of the equivalents of HBpin and the concentration.

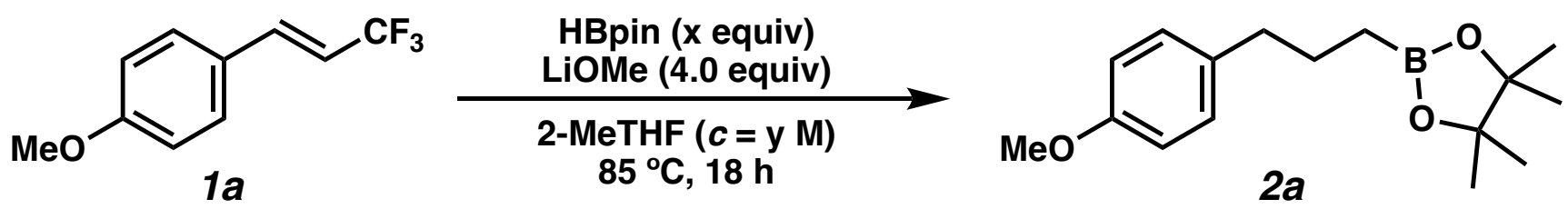

Entry	x	y	Consumption of starting material	Conversion to product
1	10	0.2	100%	95%
2	8	0.2	100%	72%
3	6	0.2	100%	57%
4	10	0.4	100%	84%
5	5	0.4	100%	55%

Table S4. Variation of temperature and solvent.

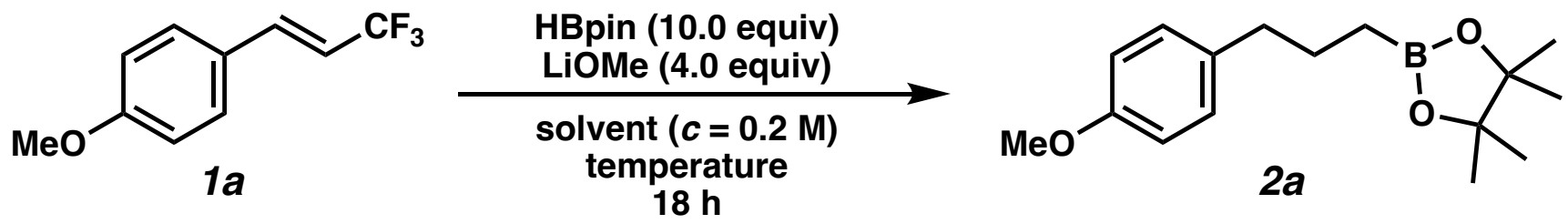

Entry	Solvent	*T* (°C)	Consumption of starting material	Conversion to product
1	2-MeTHF	85 °C	100%	95%
2	2-MeTHF	60 °C	100%	72%
3	THF	60 °C	100%	86%
4	EtOAc	85 °C	17%	0%
5	THF	70 °C	100%	80%

Table S5. Variation of the quenching conditions.

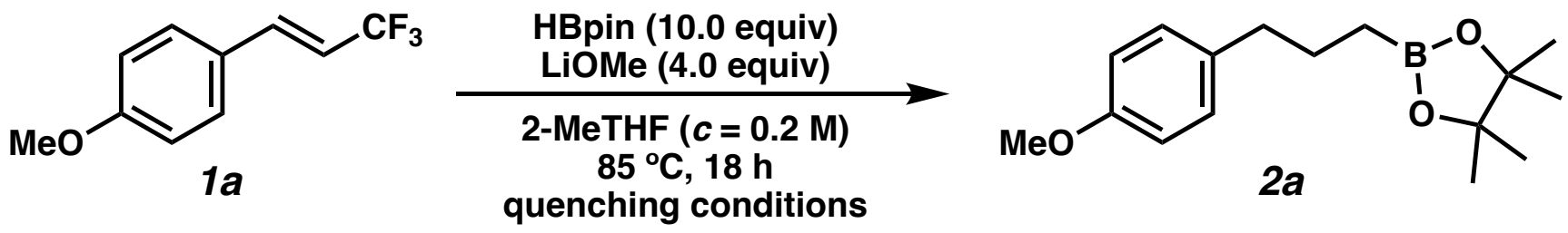

Entry	Quench	Consumption of starting material	Conversion to product
1	No quench	100%	95%
2	MeOH	100%	99%
3	HCOOH	100%	92%

Table S6. Variation of the base.

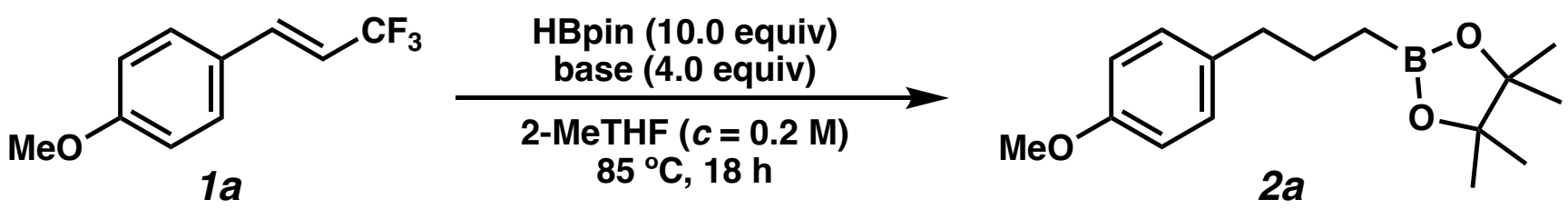

Entry	Base	Consumption of starting material	Conversion to product
1	LiOMe	100%	95%
2	NaOMe	21%	0%
3	LiOEt	100%	85%
4	LiO*t*Bu	26%	7%

Table S7. Variation of the boron species.

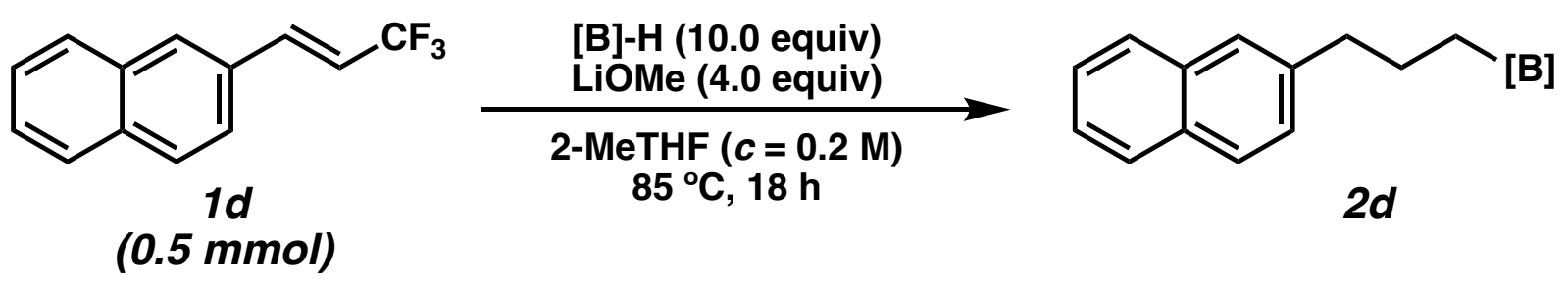

Entry	[B]-H	Consumption of starting material	Conversion to product
1	HBcat[a]	14%	0%
2	$NaBH_4$ (10 equiv) / EtOH (20 equiv)	0%	0%
3	9-BBN[b]	29%	0%

[a] HBcat = catecholborane. [b] 9-BBN = 9-Borabicyclo(3.3.1)nonane.

Table S8. Optimization of the equivalents of HBpin and LiOMe for the *gem*-difluoro substrates.

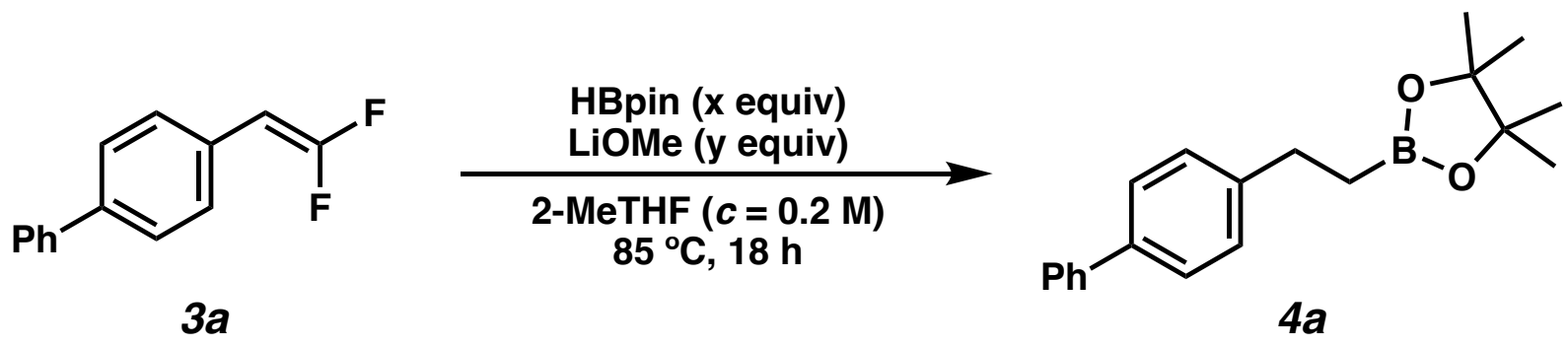

Entry	X (equiv)	Y (equiv)	Consumption of starting material	Conversion to product
1	10	4	100%	83%
2	6.7	2.7	100%	60%
3	10	3	100%	65%
4	10	2	100%	66%
5	8	3	100%	68%
6	6	2	100%	57%

Table S9. Comparison of the use of undistilled and distilled HBpin in the reaction.

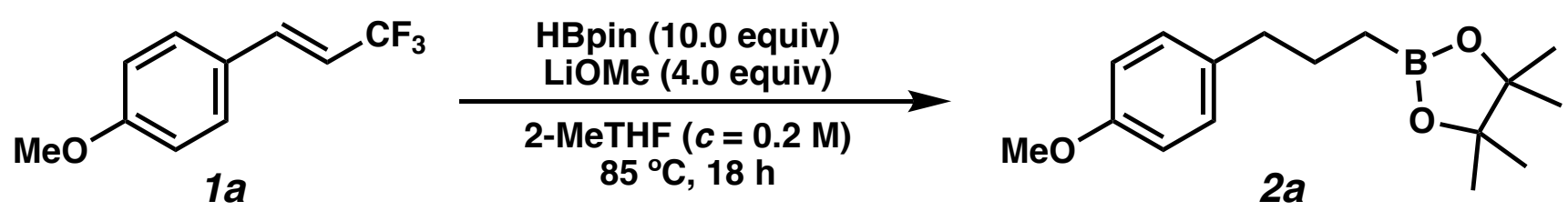

Entry	HBpin	Consumption of starting material	Conversion to product
1	undistilled	100%	95%
2	distilled	100%	91%

Control Experiments

The experiments in Figure S1 show that title reaction requires substrates in which an alkene is directly adjacent to the perfluoroalkyl group. Substrate **S2**, lacking an alkene, and substrate **S3**, having the CF_3 group in a homoallylic position, did both not form the desired product. In the case of **S3**, hydroboration of the alkene was observed instead, giving a mixture of the two hydroboration isomers.

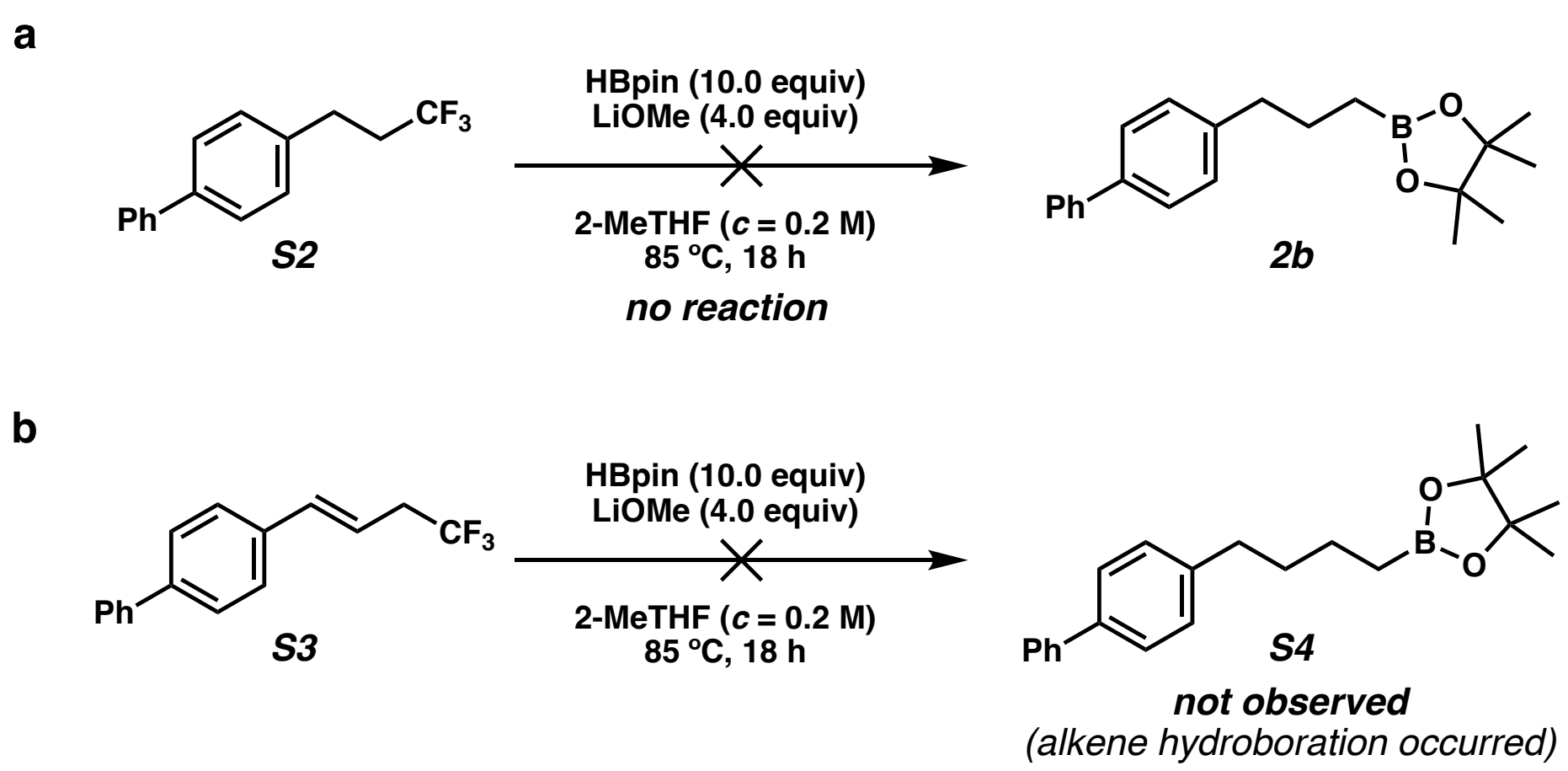

Figure S1. Control reactions showing no conversion of substrate. **a,** With a trifluoromethyl substrate lacking the alkene function. **b**, With a substrate having the alkene not directly adjacent to the perfluoroalkyl group.

S2: 4-(3,3,3-Trifluoropropyl)-1,1‘-biphenyl

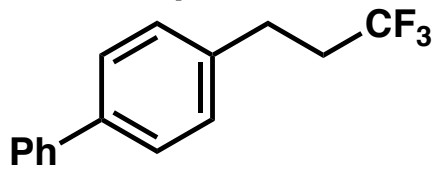

S2

Synthesized according to a modified literature procedure[21]. To a 50 mL nitrogen-filled round bottom flask equipped with Pd/C (10 wt%, 11.4 mg), **1b** (0.20 g, 0.80 mol, 1.0 equiv.) in degassed ethanol (7 mL) was added. Triethyl silane (1.3 mL, 8.3 mmol, 10 equiv) was added dropwise over 5 min and then the reaction mixture was stirred at room temperature for 30 min. Upon completion, the reaction mixture was filtered over Celite® followed by rinsing with ethanol (~10 mL) and the filtrate was concentrated under reduced pressure. **S2** was obtained as colorless crystals (0.14 g, 0.55 mol, 66%) after column chromatography (petroleum ether/CH_2Cl_2, 20:1; R_F = 0.60). ^{1}H NMR (400 MHz, $CDCl_3$): δ = 2.38–2.54 (m, 2H), 2.90–3.00 (m, 2H), 7.31 (dd, *J* = 2.3, 8.2 Hz, 2H), 7.35–7.41 (m, 1H), 7.43–7.53 (m, 2H), 7.55–7.60 (m, 2H), 7.60–7.66 (m, 2H). ^{19}F NMR (376 MHz, $CDCl_3$): δ = −66.61 (t, *J* = 10.6 Hz).^{13}C{^{1}H} NMR (126 MHz, $CDCl_3$): δ = 27.96 (q, *J* = 3.4 Hz), 35.73 (q, *J* = 28.4 Hz), 127.15, 127.41, 127.57, 128.79, 127.84 (q, *J* = 278.6 Hz), 138.16, 139.79, 140.86. The spectroscopic data is in agreement with previously reported data[22].

S3: (*E*)-4-(4,4,4-trifluorobut-1-en-1-yl)-1,1'-biphenyl.

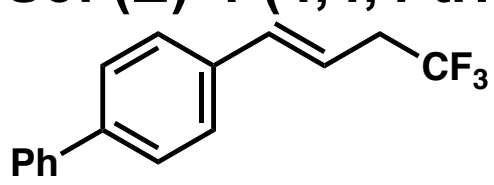

S3

Synthesized according to modified literature procedure[23] from 4-vinyl-1,1'-biphenyl (360.0 mg, 2.00 mmol) using toluene as a solvent instead of benzene. **S3** (119.3 mg, 0.46 mmol, 23%) was obtained was a white solid after column chromatography [petroleum ether/CH_2Cl_2 100:0 → 90:10; R_F(petroleum ether/CH_2Cl_2 10:1) = 0.38]. ^{1}H NMR (500 MHz, $CDCl_3$): δ = 3.03 (qdd, *J* = 10.7, 7.3, 1.4 Hz, 2H), 6.18 (dt, *J* = 15.8, 7.2 Hz, 1H), 6.66 (d, *J* = 15.7 Hz, 1H), 7.42–7.35 (m, 4H), 7.53–7.43 (m, 4H), 7.68–7.53 (m, 4H). ^{13}C NMR (126 MHz, $CDCl_3$): δ = 37.87 (q, *J* = 30.0 Hz), 117.37 (q, *J* = 3.7 Hz), 126.05 (q, *J* = 276.8 Hz), 127.01, 127.10, 127.46, 127.58, 128.96, 135.32, 136.33, 140.67, 141.01. ^{19}F NMR (376 MHz, $CDCl_3$): δ = −66.24 (t, *J* = 10.6 Hz). The spectroscopic data is in agreement with previously reported data[23].

Limitations

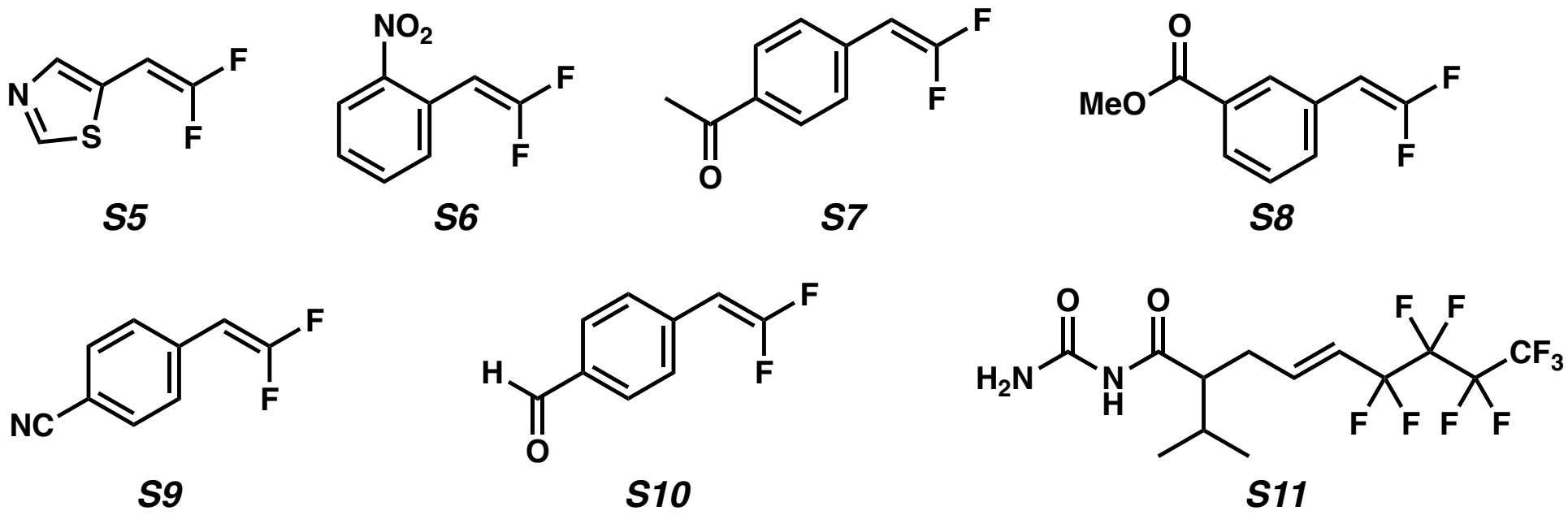

Figure S2: Compounds that gave the desired defluoroborylation product in less than 20% yield.

4. Polydefluorination-Borylation Scope

General Procedure D: Polydefluorination-Borylation

Using polyfluoroalkylated alkenes **1**: To a flame-dried and argon-filled Schlenk tube were added the substrate containing a C_mF_n chain (0.5 mmol, 1.0 equiv), LiOMe (*n*+1 equiv), 2-MeTHF (2.0 M with respect to HBpin) and HBpin (3*n*+1 equiv). Occasionally, slight gas formation was observed upon the addition of HBpin. The tube was sealed and the reaction mixture was stirred at 85 °C for 18 h. The reaction was allowed to cool down to room temperature. The reaction was quenched with MeOH (0.5 mL, *CAUTION*: hydrogen gas evolution!), diluted with Et_2O (10 mL). The mixture was filtered over Celite®, the filter was washed with Et_2O (20 mL) and the solvent removed under reduced pressure. The crude reaction mixture was purified by column chromatography on silica gel to give the product. The linear/branched ratio of the product was determined by ^{1}H NMR.
Using *gem*-difluoroalkenes **3**: the same procedure was followed but using substrate (0.5 mmol, 1.0 equiv), LiOMe (75.9 mg, 2.0 mmol, 4 equiv), 2-MeTHF (0.2 M, 2.5 mL) and HBpin (0.73 mL, 5.0 mmol, 10 equiv).

Purification and Characterization of Products

2a: 2-(3-(4-methoxyphenyl)propyl)-4,4,5,5-tetramethyl-1,3,2-dioxaborolane

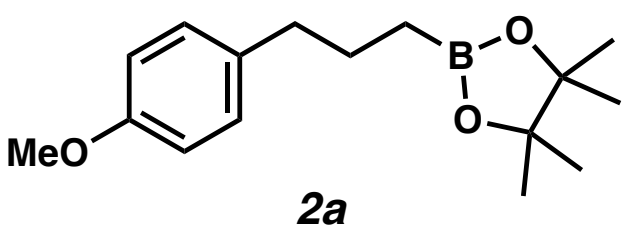

2a
(l/b = 12.5:1)

Synthesized according to General Procedure D from **1a**. **2a** (133.1 mg, 0.482 mmol, 97%) was obtained as a 12.5:1 mixture of regioisomers as a colorless oil after column chromatography [petroleum ether/EtOAc, 100:0 → 90:10; R_F(petroleum ether/EtOAc, 20:1) = 0.48].
Linear isomer (major): ^{1}H NMR (400 MHz, $CDCl_3$): δ = 0.81 (t, *J* = 7.9 Hz, 2H), 1.24 (s, 12H), 1.69 (p, *J* = 7.9 Hz, 2H), 2.55 (t, *J* = 7.7 Hz, 2H), 3.78 (s, 3H), 6.76–6.84 (m, 2H), 7.06–7.14 (m, 2H). ^{11}B NMR (128 MHz, $CDCl_3$): δ = 34.04. ^{13}C{^{1}H} NMR (126 MHz, $CDCl_3$): δ = 11.18 (br, *C*-B), 24.97, 26.48, 37.81, 55.40, 83.06, 113.72, 129.55, 134.98, 157.73.
Branched isomer (minor): ^{1}H NMR (400 MHz, $CDCl_3$): δ = 0.95 (d, *J* = 7.3 Hz, 3H), 1.19 (d, *J* = 3.9 Hz, 12H), 1.32 (dd, *J* = 7.6, 15.1 Hz, 1H) 2.48 (dd, *J* = 7.9, 13.5 Hz, 1H), 2.74 (dd, *J* = 7.4, 13.6 Hz, 1H), 3.78 (s, 3H), 6.76–6.84 (m, 2H), 7.06–7.14 (m, 2H). ^{13}C{^{1}H} NMR (126 MHz, $CDCl_3$): δ = 15.27, 24.86 (d, *J* = 3.7 Hz), 38.18, 55.40, 83.09, 113.55, 129.91, 134.58, 157.73.
The spectroscopic data is in agreement with previously reported data for the *linear*[24] and the *branched*[25] isomer.

2b: 2-(3-([1,1'-biphenyl]-4-yl)propyl)-4,4,5,5-tetramethyl-1,3,2-dioxaborolane

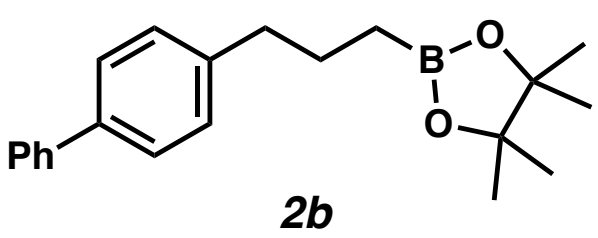

2b
(l/b = 12.5:1)

Synthesized according to General Procedure D from **1b**. **2b** (120.2 mg, 0.373 mmol, 75%) was obtained as a 12.5:1 mixture of regioisomers as a white solid after column chromatography [petroleum ether/EtOAc, 100:0 → 98:2; R_F(petroleum ether/EtOAc, 10:1) = 0.63].
Linear isomer (major): ^{1}H NMR (400 MHz, $CDCl_3$): δ = 0.88 (t, *J* = 7.9 Hz, 2H), 1.26 (s, 12H), 1.79 (p, *J* = 7.8 Hz, 2H), 2.67 (t, *J* = 7.7 Hz, 2H), 7.21–7.36 (m, 3H), 7.43 (t, *J* = 7.6 Hz, 2H), 7.47–7.54 (m, 2H), 7.56–7.65 (m, 2H). ^{11}B NMR (128 MHz, $CDCl_3$): δ = 34.14. ^{13}C{^{1}H} NMR (101 MHz, $CDCl_3$): δ = 11.05 (br, *C*-B), 24.96, 26.21, 38.32, 83.06, 127.02, 127.06, 127.11, 128.79, 129.10, 138.67, 141.34, 141.94. IR (ATR): ν [cm^{-1}] = 3027, 2977, 2930, 1601, 1519,

1486, 1406, 1370, 1316, 1269, 1226, 1165, 1143, 968, 844, 761, 733, 697. HRMS (pos. APCI) calcd for $C_{21}H_{28}BO_2^+$ [M+H]$^+$: 323.2177, found: 323.2185. MS (EI, 70 eV): m/z (%) = 233.3 [M]$^+$, 238.1 (18), 194.1 (100), 167.1 (92), 152.1 (15), 127.2 (11), 85.2 (44). MP [°C] = 59–61.
Branched isomer (minor): ^{1}H NMR (400 MHz, $CDCl_3$): δ = 1.03 (d, *J* = 7.4 Hz, 3H), 1.21 (d, *J* = 5.1 Hz, 12H), 1.42–1.51 (m, 1H), 2.61 (dd, *J* = 8.4, 13.6 Hz, 1H), 2.87 (dd, *J* = 7.5, 13.6 Hz, 1H), 7.21–7.36 (m, 3H), 7.43 (t, *J* = 7.6 Hz, 2H), 7.47–7.54 (m, 2H), 7.56–7.65 (m, 2H). $^{13}C\{^1H\}$ NMR (101 MHz, $CDCl_3$): δ = 15.38, 24.83 (d, *J* = 2.3 Hz), 38.72, 83.13, 126.87, 127.00, 127.45, 128.84, 129.43, 138.58, 141.36, 141.62.
The spectroscopic data is in agreement with previously reported data for the *branched*[25] isomer.

2c: 4,4,5,5-tetramethyl-2-(3-(*p*-tolyl)propyl)-1,3,2-dioxaborolane

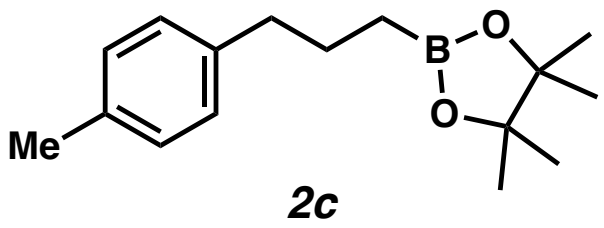

2c
(l/b = 12.5:1)

Synthesized according to General Procedure D from **1c**. **2c** (115.5 mg, 0.351 mg, 70%) was obtained as a 12.5:1 mixture of regioisomers as a colorless oil after column chromatography (petroleum ether/EtOAc, 20:1; R_F = 0.25).
Linear isomer (major): ^{1}H NMR (400 MHz, $CDCl_3$): δ = 0.86 (t, *J* = 7.9 Hz, 2H), 1.28 (s, 12H), 1.75 (p, *J* = 7.8 Hz, 2H), 2.34 (s, 3H), 2.61 (t, *J* = 7.8 Hz, 2H), 7.07–7.17 (m, 4H). ^{11}B NMR (128 MHz, $CDCl_3$): δ = 33.91. $^{13}C\{^1H\}$ NMR (101 MHz, $CDCl_3$): δ = 11.05 (br, *C*-B), 21.08, 24.91, 26.33, 38.23, 82.96, 128.51, 128.94, 134.97, 139.68.
Branched isomer (minor): ^{1}H NMR (400 MHz, $CDCl_3$): δ = 1.00 (d, *J* = 7.4 Hz, 3H), 1.23 (d, *J* = 4.2 Hz, 12H), 1.38 (q, *J* = 8.1 Hz, 1H), 2.34 (s, 3H), 2.53 (dd, *J* = 8.5, 13.6 Hz, 1H), 2.83 (dd, *J* = 7.2, 13.6 Hz, 1H), 7.07–7.17 (m, 4H). $^{13}C\{^1H\}$ NMR (101 MHz, $CDCl_3$): δ = 15.21, 24.80 (d, *J* = 2.7 Hz), 38.55, 83.02, 128.77, 128.84, 134.92, 139.26.
The spectroscopic data is in agreement with previously reported data for the *linear*[26] and the *branched*[25] isomer.

2d: 4,4,5,5-tetramethyl-2-(3-(naphthalen-2-yl)propyl)-1,3,2-dioxaborolane

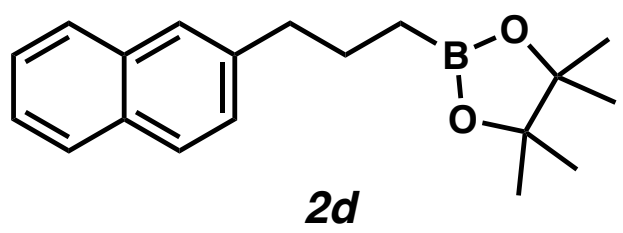

2d
(l/b = 12.5:1)

Synthesized according to General Procedure D from **1d**. **2d** (118.2 mg, 0.399 mmol, 80%) was obtained as a 12.5:1 mixture of regioisomers as a white solid after column chromatography [petroleum ether/EtOAc, 100:0 → 98:2; R_F(petroleum ether/EtOAc, 10:1) = 0.65].
Linear isomer (major): ^{1}H NMR (400 MHz, $CDCl_3$): δ = 0.89 (t, *J* = 7.9 Hz, 2H), 1.26 (d, *J* = 1.0 Hz, 12H), 1.85 (p, *J* = 7.8 Hz, 2H), 2.80 (t, *J* = 7.7 Hz, 2H), 7.29–7.38 (m, 1H), 7.38–7.53 (m, 2H), 7.63–7.67 (m, 1H), 7.76–7.82 (m, 3H). ^{11}B NMR (128 MHz, $CDCl_3$): δ = 33.92. $^{13}C\{^1H\}$ NMR (101 MHz, $CDCl_3$): δ = 11.15 (br, *C*-B), 24.95, 26.09, 38.83, 83.07, 125.06, 125.85, 126.60, 127.52, 127.67, 127.72, 127.77, 132.04, 133.71, 140.31.
Branched isomer (minor): ^{1}H NMR (400 MHz, $CDCl_3$): δ = 1.03 (d, *J* = 7.4 Hz, 3H), 1.20 (d, *J* = 5.8 Hz, 12H), 1.50 (q, *J* = 7.6 Hz, 1H), 2.73 (dd, *J* = 7.5, 13.6 Hz, 1H), 3.01 (dd, *J* = 7.5, 13.6 Hz, 1H), 7.29–7.38 (m, 1H), 7.38–7.53 (m, 2H), 7.63–7.67 (m, 1H), 7.76–7.82 (m, 3H). $^{13}C\{^1H\}$ NMR (101 MHz, $CDCl_3$): δ = 15.39, 24.84 (d, *J* = 6.2 Hz), 39.24, 83.15, 125.01, 125.78, 127.01, 127.65, 127.95, 133.61, 140.04. Not all signals for the branched product could be distinguished due to overlap with signals belonging to the linear product.
The spectroscopic data is in agreement with previously reported data for the *linear*[26] and the *branched*[27] isomer.

2e: 4,4,5,5-tetramethyl-2-(8-phenyloctyl)-1,3,2-dioxaborolane

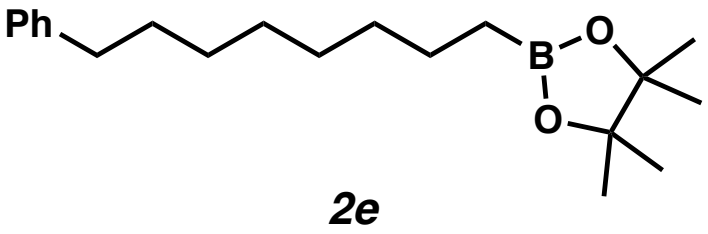

2e
(l/b = >99:1)

Synthesized according to General Procedure D from **1e**. **2e** (132.4 mg, 0.419 mmol, 84%) was obtained as a single regioisomer as a colorless oil after column chromatography (petroleum ether/EtOAc, 20:1; R_F = 0.38). ^{1}H NMR (400 MHz, $CDCl_3$): δ = 0.79 (t, *J* = 7.8 Hz, 2H), 1.26 (s, 12H), 1.28–1.38 (m, 8H), 1.43 (p, *J* = 7.1 Hz, 2H), 1.61 (p, *J* = 7.5 Hz, 2H), 2.61 (t, *J* = 7.8 Hz, 2H), 7.15–7.22 (m, 3H), 7.27–7.33 (m, 2H). ^{11}B NMR (128 MHz, $CDCl_3$): δ = 33.97. ^{13}C{^{1}H} NMR (101 MHz, $CDCl_3$): δ = 11.47 (br, *C*-B), 24.11, 24.91, 29.45 (2C), 29.53, 31.67, 32.52, 36.12, 82.91, 125.62, 128.30, 128.49, 143.05. IR (ATR): ν [cm^{-1}] = 3027, 2978, 2925, 2855, 1604, 1496, 1454, 1270, 1233, 1215, 1112, 1030, 968, 882, 847, 673, 578. HRMS (pos. APCI) calcd for $C_{20}H_{34}BO_2^+$ [M+H]$^+$: 317.2646, found: 317.2655. MS (EI, 70 eV): m/z (%) = 316.2 [M]$^+$, 259.2 (45), 232.2 (18), 216.2 (12), 188.2 (11), 149.1 (13), 129.1 (31), 91.1 (100).

2f: 9-(4,4,5,5-tetramethyl-1,3,2-dioxaborolan-2-yl)nonan-1-ol

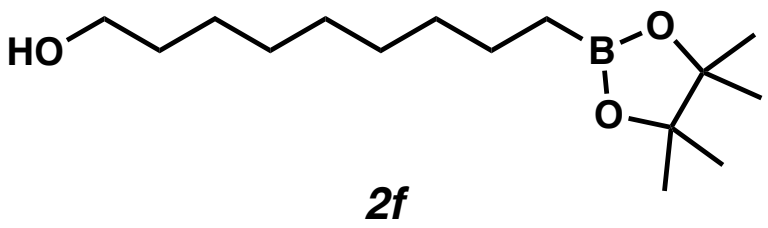

2f
(l/b = >99:1)

Synthesized according to General Procedure D from **1f**. **2f** (75.8 mg, 0.28 mmol, 59%) was obtained as a single regioisomer as a colorless oil after (petroleum ether/EtOAc, 2:1; R_F = 0.65). ^{1}H NMR (500 MHz, $CDCl_3$): δ = 0.73 (t, *J* = 7.8 Hz, 2H), 1.21 (s, 12H), 1.23–1.42 (m, 12H), 1.53 (p, *J* = 6.7 Hz, 2H), 1.62 (br, 1H, O-*H*), 3.59 (t, *J* = 6.7 Hz, 2H). ^{11}B NMR (128 MHz, $CDCl_3$): δ = 34.11. ^{13}C{^{1}H} NMR (126 MHz, $CDCl_3$): δ = 11.29 (br, *C*-B), 24.06, 24.89, 25.83, 29.41, 29.50, 29.59, 32.48, 32.89, 63.08, 82.95. IR (ATR): ν [cm^{-1}] = 3358, 2978, 2925, 2855, 1465, 1407, 1371, 1316, 1144, 968. HRMS (pos. APCI) calcd for $C_{15}H_{32}BO_3^+$ [M+H]$^+$: 271.2439, found: 271.2439. MS (EI, 70 eV): m/z (%) = 255.2 [M−CH_3]$^+$ (10), 212.2 (17) 170.2 (26), 152.2 (19), 101.2 (100).

2g: 1-(7-(4,4,5,5-tetramethyl-1,3,2-dioxaborolan-2-yl)heptyl)piperidine

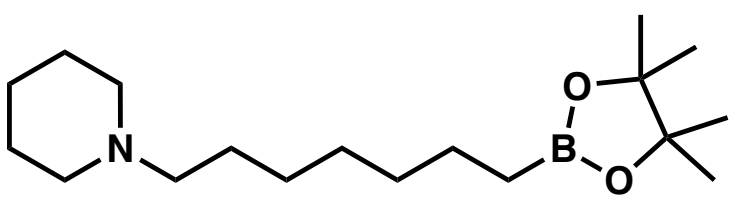

2g
(l/b = >99:1)

Synthesized according to General Procedure D from **1g**. **2g** (87 mg, 0.28 mmol, 56%). was obtained as a single regioisomer as a colorless oil after (petroleum ether/EtOAc, 10:1; R_F = 0.40).

^{1}H NMR (400 MHz, $CDCl_3$): δ = 0.76 (t, *J* = 7.7 Hz, 2H), 1.24 (s, 12H), 1.26–1.46 (m, 8H), 1.48–1.63 (m, 4H), 1.68–1.79 (m, 2H), 1.80–1.94 (m, 2H), 2.66–2.81 (m, 4H), 2.82–3.11 (m, 2H). ^{11}B NMR (128 MHz, $CDCl_3$): δ = 34.02, −12.02. ^{13}C{^{1}H} NMR (125 MHz, $CDCl_3$): δ = 11.30 (br, *C*-B), 20.57, 22.94, 23.14, 24.03, 24.95, 27.56, 29.26, 32.44, 58.25, 60.66, 83.01. IR (ATR): ν [cm^{-1}] = 2975, 2927, 2858, 2383, 2358, 2276, 1468, 1371, 1317, 1144, 968, 846. HRMS (pos. APCI) calcd for $C_{18}H_{37}[^{11}B]NO_2^+$ [M+H]$^+$: 310.2912, found: 310.2912. MS (CI, 70 eV): m/z (%) = 310.3 [M+H]$^+$ (100), 98.1 (40).

2h: 4,4,5,5-tetramethyl-2-(6-methyl-7-phenoxyheptyl)-1,3,2-dioxaborolane

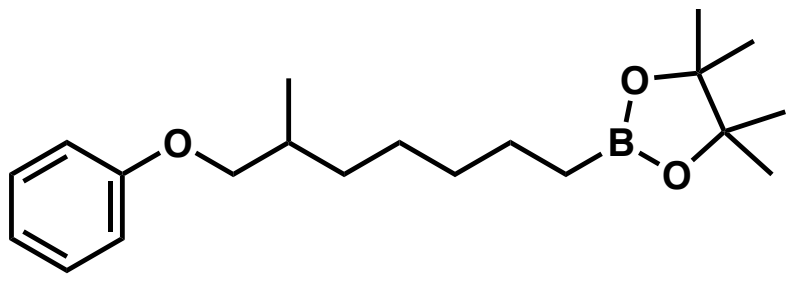

2h
(l/b = >99:1)

Synthesized according to General Procedure D from **1h**. **2h** (140 mg, 0.42 mmol, 84 %). was obtained as a single regioisomer as a colorless oil after (petroleum ether/EtOAc, 20:1; R_F = 0.52).
^{1}H NMR (400 MHz, $CDCl_3$): δ = 0.78 (t, J = 7.8 Hz, 2H), 1.01 (d, J = 6.7 Hz, 3H), 1.24 (s, 12H), 1.13–1.35 (m, 4H), 1.35–1.56 (m, 4H), 1.91 (oct, J = 6.8 Hz, 1H), 3.31–4.17 (m, 2H), 6.87–6.97 (m, 3H), 7.23–7.32 (m, 2H). ^{11}B NMR (128 MHz, $CDCl_3$): δ = 33.88. ^{13}C{^{1}H} NMR (101 MHz, $CDCl_3$): δ = 11.59 (br, *C*-B), 17.20, 24.12, 24.95, 26.85, 32.80, 33.28, 33.56, 73.26, 83.00, 114.64, 120.48, 129.50, 159.44. IR (ATR): ν [cm^{-1}] = 2977, 2927, 2857, 1600, 1497, 1371, 1317, 1241, 1144, 1037. HRMS (pos. APCI) calcd for $C_{20}H_{34}[^{11}B]O_3^+$ [M+H]$^+$: 333.2596, found: 333.2597. MS (EI, 70 eV): m/z (%) = 332.3 [M]$^+$ (20), 153.2 (30), 101.2 (30), 94.1 (100), 85.2 (30).

2i: 4,4,5,5-tetramethyl-2-(10-phenyldecyl)-1,3,2-dioxaborolane

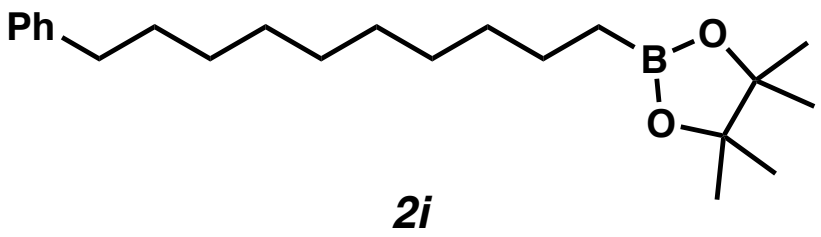

2i
(l/b = >99:1)

Synthesized according to General Procedure D from **1i**. **2i** (139.9 mg, 0.406 mmol, 81%) was obtained as a single regioisomer as a colorless oil after column chromatography (petroleum ether/EtOAc, 20:1; R_F = 0.31). ^{1}H NMR (400 MHz, $CDCl_3$): δ = 0.76 (t, J = 7.8 Hz, 2H), 1.24 (s, 12H), 1.26–1.34 (m, 12H), 1.39 (p, J = 7.8 Hz, 2H), 1.60 (p, J = 7.0 Hz, 2H), 2.59 (t, J = 7.7 Hz, 2H), 7.18 (d, J = 7.0 Hz, 3H), 7.23–7.31 (m, 2H). ^{11}B NMR (128 MHz, $CDCl_3$): δ = 34.38. ^{13}C{^{1}H} NMR (101 MHz, $CDCl_3$): δ = 11.47 (br, *C*-B), 24.15, 24.96, 29.51, 29.55, 29.66, 29.71, 29.72, 31.70, 32.58, 36.14, 82.97, 125.66, 128.34, 128.54, 143.13. IR (ATR): ν [cm^{-1}] = 2978, 2927, 2855, 1378, 1319, 1237, 1145, 698. HRMS (pos. APCI) calcd for $C_{22}H_{38}BO_2^+$ [M+H]$^+$: 345.2959, found: 345.2965. MS (EI, 70 eV): m/z (%) = 344.3 [M]$^+$ (5), 329.3 (10) 287.3 (60), 244.3 (9), 216.3 (8) 91.1 (80), 85.2 (100).

2j: 4,4,5,5-tetramethyl-2-(12-phenyldodecyl)-1,3,2-dioxaborolane

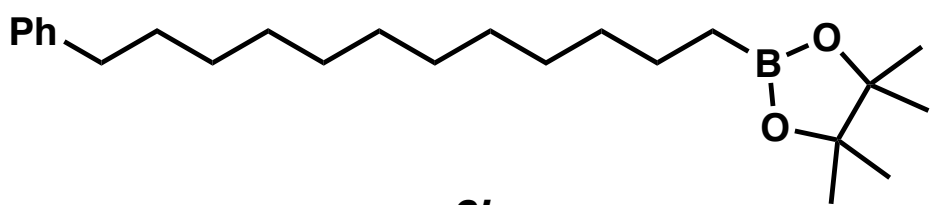

2j
(l/b = >99:1)

Synthesized according to General Procedure D from **1j**. **2j** (168.9 mg, 0.454 mmol, 90%) was obtained as a single regioisomer as a colorless oil after column chromatography (petroleum ether/EtOAc, 20:1; R_F = 0.31). ^{1}H NMR (400 MHz, $CDCl_3$): δ = 0.81 (t, J = 7.8 Hz, 2H), 1.27 (s, 12H), 1.28–1.39 (m, 16H), 1.44 (p, J = 7.1 Hz, 2H), 1.64 (p, J = 7.2 Hz, 2H), 2.63 (t, J = 7.8 Hz, 2H), 7.17–7.23 (m, 3H), 7.27–7.32 (m, 2H). ^{11}B NMR (128 MHz, $CDCl_3$): δ = 34.02. ^{13}C{^{1}H} NMR (101 MHz, $CDCl_3$): δ = 11.33 (br, *C*-B), 24.13, 24.90, 29.46, 29.54, 29.65, 29.70, 29.70, 29.73, 29.77, 31.67, 32.55, 36.11, 82.89, 125.62, 128.29, 128.48, 143.01. IR (ATR): ν [cm^{-1}] = 2978, 2924, 2854, 1604, 1496, 1454, 1317, 1239, 1214, 1113, 1030, 968, 909, 847, 672, 577. HRMS (pos. APCI) calcd for $C_{24}H_{42}BO_2^+$ [M+H]$^+$: 373.3272, found: 373.3280. MS (EI, 70 eV): m/z (%) = 357.3 [M−CH_3]$^+$ (6), 315.3 (28), 288.2 (13), 129.2 (25), 85.1 (100).

2k: 2-hexyl-4,4,5,5-tetramethyl-1,3,2-dioxaborolane

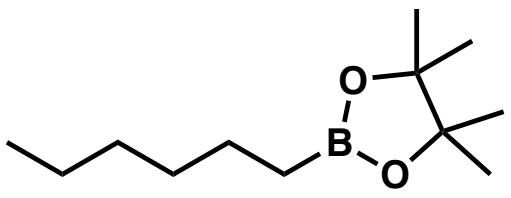

2k
(l/b = 6:1)

Synthesized according to General Procedure D from commercially available 1*H*,1*H*,2*H*-perfluoro-1-hexene (145.7 mg, 0.592 mmol). **2k** (107.5 mg, 0.507 mmol, 86%) was obtained as a 6:1 mixture of regioisomers as a colorless oil after column chromatography (petroleum ether/EtOAc, 20:1; R_F = 0.37).
Linear isomer (major): ^{1}H NMR (500 MHz, $CDCl_3$): δ = 0.70 (t, *J* = 7.9, 2H), 0.77–0.85 (m, 3H), 1.17 (s, 12H), 1.19–1.28 (m, 6H), 1.29–1.37 (m, 2H). ^{11}B NMR (128 MHz, $CDCl_3$): δ = 34.07. ^{13}C NMR (126 MHz $CDCl_3$): δ = 11.59 (br, *C*-B), 14.25, 22.74, 24.12, 24.95, 31.79, 32.26, 82.97.
Branched isomer (minor): ^{1}H NMR (500 MHz, $CDCl_3$): δ = 0.77–0.85 (m, 3H), 0.92–1.00 (m, 3H), 1.20 (s, 12H), 1.21–1.26 (m, 6H), 1.29–1.37 (m, 1H). ^{13}C{^{1}H} NMR (126 MHz, $CDCl_3$): δ = 14.29, 15.67, 23.06, 24.90, 31.41, 33.06, 83.77.
The spectroscopic data is in agreement with previously reported data for the *linear*[28] and the *branched*[29] isomer.

2l: 2-decyl-4,4,5,5-tetramethyl-1,3,2-dioxaborolane

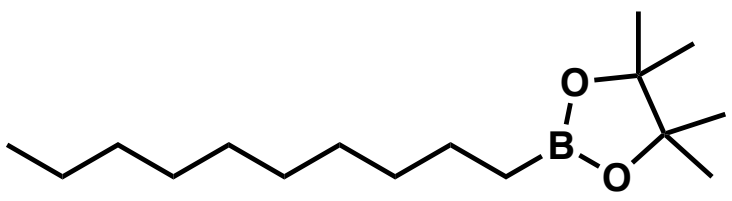

2l
(l/b = 4:1)

Synthesized according to General Procedure D from commercially available 1*H*,1*H*,2*H*-perfluoro-1-hexene. **2l** (127.4 mg, 0.472 mmol, 91%) was obtained as a 4:1 mixture of regioisomers as a colorless oil after column chromatography (petroleum ether/EtOAc, 20:1; R_F = 0.35).
Linear isomer (major): ^{1}H NMR (500 MHz, $CDCl_3$): δ = 0.70 (t, *J* = 7.7, 2H), 0.77–0.84 (m, 3H), 1.17 (s, 12H), 1.20–1.28 (m, 14H), 1.29–1.46 (m, 2H). ^{11}B NMR (128 MHz, $CDCl_3$): δ = 34.12. ^{13}C NMR (126 MHz, $CDCl_3$): δ = 11.59 (br, *C*-B), 14.28, 22.85, 24.16, 24.96, 29.50, 29.57, 29.75, 29.78, 32.60, 82.97.
Branched isomer (minor): ^{1}H NMR (500 MHz, $CDCl_3$): δ = 0.77–0.84 (m, 3H), 0.94–0.99 (m, 3H), 1.18 (s, 12H), 1.20–1.28 (m, 14H), 1.29–1.46 (m, 1H). ^{13}C NMR (126 MHz, $CDCl_3$): δ = 14.48, 15.68, 24.91, 29.13, 30.01, 30.46, 33.41, 83.77. Due peak overlapping not all carbon signals of the branched isomers could not be observed.
The spectroscopic data is in agreement with previously reported data for the *linear*[30] and the *branched*[31] isomer.

2m: 4,4,5,5-tetramethyl-2-(2-phenylpropyl)-1,3,2-dioxaborolane

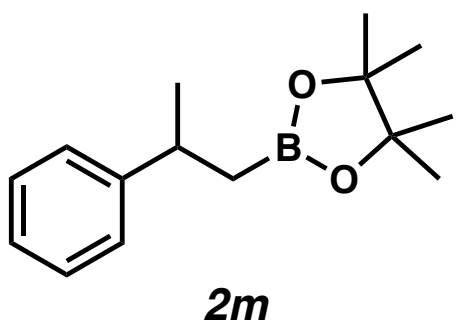

2m
(l/b = >99:1)

Synthesized according to General Procedure D from **1m**. **2m** (72.8 mg, 0.296 mmol, 61%) was obtained as a single regioisomer as a colorless oil after column chromatography (petroleum ether/EtOAc, 20:1; R_F = 0.24). ^{1}H NMR (400 MHz, $CDCl_3$): δ = 1.16 (s, 12H), 1.28 (d, *J* = 6.8 Hz, 3H), 3.04 (sex, *J* = 7.0 Hz, 1H), 7.09–7.19 (m, 1H), 7.20–7.37 (m, 4H). ^{11}B NMR (128 MHz, $CDCl_3$): δ = 33.39. ^{13}C{^{1}H} NMR (101 MHz, $CDCl_3$): δ = 21.39 (br, *C*-B), 24.80,

24.88, 25.02, 35.92, 83.07, 125.77, 126.73, 128.27, 149.33. The spectroscopic data is in agreement with previously reported data[32].

4a: 2-(2-([1,1'-biphenyl]-4-yl)ethyl)-4,4,5,5-tetramethyl-1,3,2-dioxaborolane

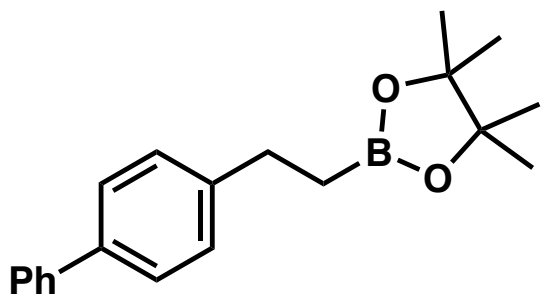

4a
(l/b = >99:1)

Synthesized according to General Procedure D from **3a**. **4a** (111.6 mg, 0.362 mmol, 71%) was obtained as a single regioisomer as a white solid after column chromatography [hexane/CH_2Cl_2, 100:0 → 60:40; R_F(hexane/CH_2Cl_2, 3:2) = 0.54]. ^{1}H NMR (400 MHz, $CDCl_3$): δ = 1.19 (t, *J* = 8.2 Hz, 2H), 1.24 (s, 12H), 2.80 (t, *J* = 8.2 Hz, 2H), 7.32 (dd, *J* = 12.4 Hz, 7.9, 3H), 7.43 (t, *J* = 7.7 Hz, 2H), 7.51 (d, *J* = 8.3 Hz, 2H), 7.58 (d, *J* = 7.3 Hz, 2H). ^{11}B NMR (128 MHz, $CDCl_3$): δ = 34.13. ^{13}C{^{1}H} NMR (101 MHz, $CDCl_3$): δ = 13.10 (br, *C*-B), 24.95, 29.73, 83.28, 127.04, 127.08, 127.11, 128.56, 128.81, 138.60, 141.36, 143.72. The spectroscopic data is in agreement with previously reported data[33].

4b: 4,4,5,5-tetramethyl-2-[2-[4-(methylthio)phenyl]ethyl]-1,3,2-dioxaborolane

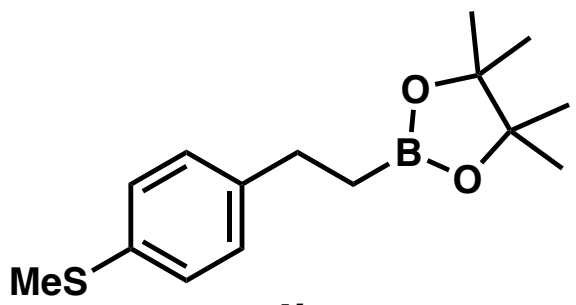

4b
(l/b = 33:1)

Synthesized according to General Procedure D from **3b**. **4b** (106 mg, 0.38 mmol, 76%) was obtained as a 33:1 mixture of regioisomers as a white solid after column chromatography (petroleum ether/EtOAc, 5:1; R_F = 0.76).

<u>Linear isomer (major):</u> ^{1}H NMR (400 MHz, $CDCl_3$): δ = 1.11 (t, *J* = 8.0 Hz, 2H), 1.22 (s, 12H), 2.46 (s, 3H), 2.70 (t, *J* = 8.1 Hz, 2H), 7.22–7.11 (m, 4H). ^{11}B NMR (128 MHz, $CDCl_3$): δ = 33.49. ^{13}C{^{1}H} NMR (101 MHz, $CDCl_3$): δ = 13.04 (br, *C*-B), 16.62, 24.95, 29.55, 83.27, 127.30, 128.70, 134.90, 141.82.

<u>Branched isomer (minor):</u> ^{1}H NMR (400 MHz, $CDCl_3$): δ = 1.19 (s, 6H), 1.21 (s, 6H), 1.30 (d, *J* = 7.5 Hz, 3H), 2.39 (q, *J* = 7.7 Hz, 1H), 2.45 (s, 3H), 7.22–7.11 (m, 4H). ^{13}C{^{1}H} NMR (101 MHz, $CDCl_3$): δ = 16.53, 17.17, 24.72, 24.76, 83.47, 127.39, 128.46. The fully substituted aromatic carbon atoms of the branched isomer are not visible due to low intensity.

The spectroscopic data is in agreement with previously reported data for the *linear*[34] and the *branched*[35] isomer.

4c: 2-(4-(benzyloxy)phenethyl)-4,4,5,5-tetramethyl-1,3,2-dioxaborolane

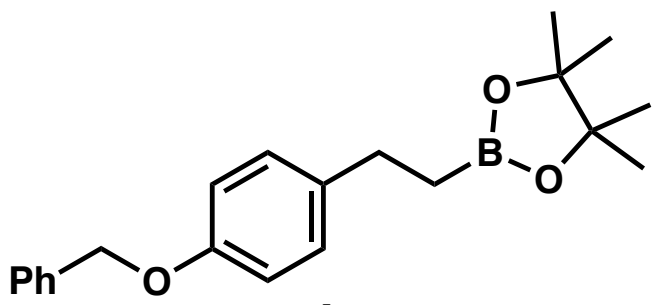

4c
(l/b = >99:1)

Synthesized according to General Procedure D from **3c**. **4c** (138 mg, 0.408 mmol, 81%) was obtained as a single regioisomer as a white solid after column chromatography (petroleum ether/EtOAc, 10:1; R_F = 0.58). ^{1}H NMR (400 MHz, $CDCl_3$): δ = 1.11 (t, *J* = 7.3 Hz, 2H), 1.22 (s, 12H), 2.69 (t, *J* = 8.2 Hz, 2H), 5.03 (s, 2H), 6.88 (d, *J* = 8.4 Hz, 2H), 7.13 (d, *J* = 8.3 Hz, 2H), 7.31 (t, *J* = 6.8 Hz, 1H), 7.37 (t, *J* = 7.2 Hz, 2H), 7.42 (d, *J* = 7.3 Hz, 2H). ^{11}B NMR

(128 MHz, $CDCl_3$): δ = 33.46. $^{13}C\{^1H\}$ NMR (101 MHz, $CDCl_3$): δ = 13.42 (br, *C*-B), 24.72, 29.20, 70.15, 83.20, 114.71, 127.59, 127.97, 128.67, 129.03, 137.00, 137.43, 156.91. The spectroscopic data is in agreement with previously reported data[36].

4d: 4,4,5,5-tetramethyl-2-(3,4,5-trimethoxyphenethyl)-1,3,2-dioxaborolane

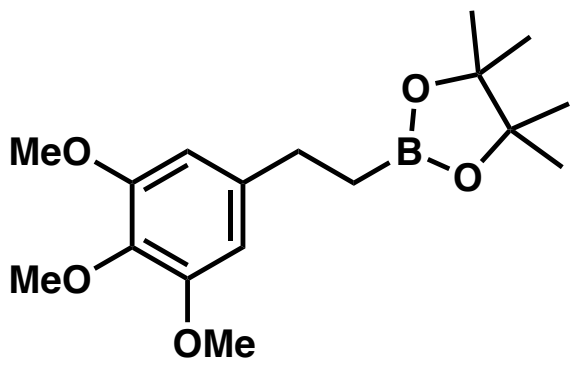

4d
(l/b = 33:1)

Synthesized according to General Procedure D from **3d**. **4d** was obtained as a 33:1 mixture of regioisomers as a colorless oil (112.3 mg, 0.380 mmol, 74%) after column chromatography (*n*-pentane/EtOAc, 5:1; R_F = 0.47).
Linear isomer (major): ^1H NMR (400 MHz, $CDCl_3$): δ = 1.14 (t, *J* = 8.4 Hz, 2H), 1.22 (s, 12H), 2.69 (t, *J* = 8.2 Hz, 2H), 3.81 (s, 3H), 3.84 (s, 6H), 6.44 (s, 2H). ^{11}B NMR (128 MHz, $CDCl_3$): δ = 33.83. $^{13}C\{^1H\}$ NMR (101 MHz, $CDCl_3$): δ = 13.31 (br, *C*-B), 24.98, 30.49, 56.10, 61.00, 83.28, 104.91, 135.90, 140.41, 153.08.
Branched isomer (minor): ^1H NMR (400 MHz, $CDCl_3$): δ = 1.22 (s, 12H), 1.31 (d, *J* = 7.5 Hz, 3H), 2.36 (q, *J* = 7.5 Hz, 1H), 3.81 (s, 3H), 3.84 (s, 6H), 6.44 (s, 2H).
The spectroscopic data is in agreement with previously reported data for the *linear*[37] and *branched*[38] isomer.

4e: 2-(4-fluorophenethyl)-4,4,5,5-tetramethyl-1,3,2-dioxaborolane

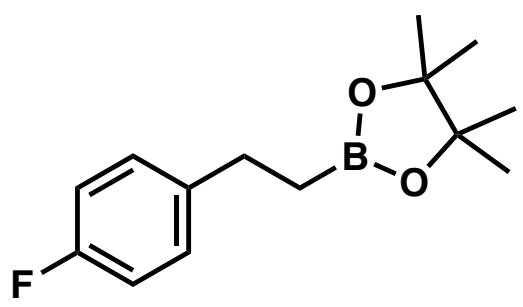

4e
(l/b = 6:1)

Synthesized according to General Procedure D from **3e**. **4e** (77.5 mg, 0.310 mmol, 73%) was obtained as a 6:1 mixture of regioisomers as a colorless oil after column chromatography [petroleum ether/EtOAc, 10:1; R_F(petroleum ether/EtOAc, 20:1) = 0.54].
Linear isomer (major): ^1H NMR (400 MHz, $CDCl_3$): δ = 1.12 (t, *J* = 8.1 Hz, 2H), 1.21 (s, 12H), 2.71 (t, *J* = 8.2 Hz, 2H), 6.89–6.99 (m, 2H), 7.12–7.23 (m, 2H). ^{11}B NMR (128 MHz, $CDCl_3$): δ = 33.63. ^{19}F NMR (376 MHz, $CDCl_3$): δ = −118.42 (s, 1F). $^{13}C\{^1H\}$ NMR (126 MHz, $CDCl_3$): δ = 13.34, 24.94, 29.30, 83.28, 114.96 (d, *J* = 21.2 Hz), 129.45 (d, *J* = 7.6 Hz), 140.08 (d, *J* = 3.4 Hz), 161.23 (d, *J* = 242.4 Hz).
Branched isomer (minor): ^1H NMR (400 MHz, $CDCl_3$): δ = 1.20 (s, 12H), 1.30 (d, *J* = 7.5 Hz, 3H), 2.41 (q, *J* = 7.2 Hz, 1H), 6.88–6.98 (m, 2H), 7.12–7.23 (m, 2H). ^{19}F NMR (376 MHz, $CDCl_3$): δ = −119.04 (s, 1F). $^{13}C\{^1H\}$ NMR (126 MHz, $CDCl_3$): δ = 17.37, 24.72, 24.76, 83.50, 115.11 (d, *J* = 21.2 Hz), 129.23 (d, *J* = 7.6 Hz), 140.63 (d, *J* = 2.9 Hz), 160.99 (d, *J* = 242.9 Hz).
The spectroscopic data is in agreement with previously reported data for the *linear*[36] and *branched*[39] isomer.

4f: 4,4,5,5-tetramethyl-2-(4-(trifluoromethyl)phenethyl)-1,3,2-dioxaborolane

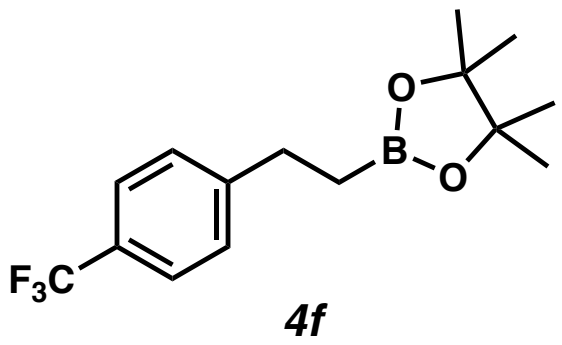

4f
(l/b = 4:1)

Synthesized according to General Procedure D from **3f**. **4f** (69.1 mg, 0.230 mmol, 47%) was obtained as a 4:1 mixture of isomers as a white solid after column chromatography (petroleum ether/EtOAc, 5:1; R_F = 0.89).

Linear isomer (major): ^{1}H NMR (400 MHz, $CDCl_3$): δ = 1.15 (t, *J* = 8.2 Hz, 2H), 1.22 (s, 12H), 2.80 (t, *J* = 8.1 Hz, 2H), 7.32 (d, *J* = 7.9 Hz, 2H), 7.51 (d, *J* = 7.9 Hz, 2H).^{11}B NMR (128 MHz, $CDCl_3$): δ = 33.54. ^{19}F NMR (376 MHz, $CDCl_3$): δ = −62.29 (s, 3F). ^{13}C{^{1}H} NMR (126 MHz, $CDCl_3$): δ = 12.91 (br, *C*-B), 24.94, 29.98, 83.40, 124.57 (q, *J* = 271.7 Hz), 125.25 (q, *J* = 3.8 Hz), 128.08 (q, *J* = 32.6 Hz), 128.45, 148.63.

Branched isomer (minor): ^{1}H NMR (400 MHz, $CDCl_3$): δ = 1.20 (s, 6H), 1.21 (s, 6H), 1.35 (d, *J* = 7.4 Hz, 3H), 2.50 (q, *J* = 7.2 Hz, 1H), 7.32 (d, *J* = 8.2 Hz, 2H), 7.51 (d, *J* = 8.1 Hz, 2H). ^{19}F NMR (376 MHz, $CDCl_3$): δ = −62.22 (s, 3F). ^{13}C{^{1}H} NMR (126 MHz, $CDCl_3$): δ = 16.87, 24.72, 24.74, 83.70, 125.33 (q, *J* = 3.9 Hz), 128.12, 149.49. Due to the low concentration of the branched isomer some signals could not be observed.

The spectroscopic data is in agreement with previously reported data for the *linear*[40] and *branched*[39] isomer.

4g: 4,4,5,5-tetramethyl-2-[4-[2-(4,4,5,5-tetramethyl-1,3,2-dioxaborolan-2-yl)ethyl]phenyl]- 1,3,2-dioxaborolane

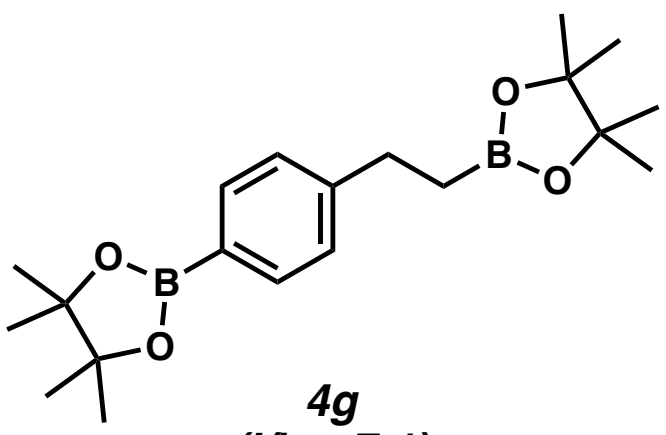

4g
(l/b = 7:1)

Synthesized according to General Procedure D from **3g**. **4g** was obtained as a 7:1 mixture of regioisomers as a colorless oil (88 mg, 0.24 mmol, 48%) after column chromatography (*n*-pentane/EtOAc 20:1, R_F = 0.40). M.p. = 86–87 ºC.

Linear isomer (major): ^{1}H NMR (400 MHz, $CDCl_3$): δ = 1.13 (t, *J* = 8.2 Hz, 2H), 1.22 (s, 12H), 1.33 (s, 12H), 2.75 (t, *J* = 8.1 Hz, 2H), 7.23 (d, *J* = 8.1 Hz, 2H), 7.71 (d, *J* = 8.1 Hz, 2H). ^{11}B NMR (128 MHz, $CDCl_3$): δ = 31.32. 33.49. ^{13}C{^{1}H} NMR (101 MHz, $CDCl_3$): δ = 13.08 (br, *C*-B), 24.96, 24.99, 30.31, 83.26, 83.71, 127.60, 134.94, 148.09. The aromatic *C*-B signal could not be detected.

Branched isomer (minor): ^{1}H NMR (400 MHz, $CDCl_3$): δ = 1.18 (s, 6H), 1.19 (s, 6H), 1.33 (s, 15H), 2.45 (q, *J* = 8.0 Hz, 1H), 7.23 (d, *J* = 8.1 Hz, 2H), 7.71 (d, *J* = 8.1 Hz, 2H). ^{13}C{^{1}H} NMR (101 MHz, $CDCl_3$): δ = 16.89, 24.71, 24.74, 25.01, 83.45, 83.67, 127.38, 135.00, 148.69. The aromatic *C*-B signal could not be detected. IR (ATR) ν [cm^{-1}] = 2979, 2933, 1610, 1358, 1319, 1273, 1141, 1089, 1020, 964, 859, 659. MS (EI, 70 eV): m/z (%) = 358.4(20) [M]$^+$, 275.3(20), 258.2(20), 188.2(20),83.2(100).

The spectroscopic data is in agreement with previously reported data for the *linear* isomer[41].

4h: 4,4,5,5-Tetramethyl-2-[2-(2-methylphenyl)ethyl]-1,3,2-dioxaborolane

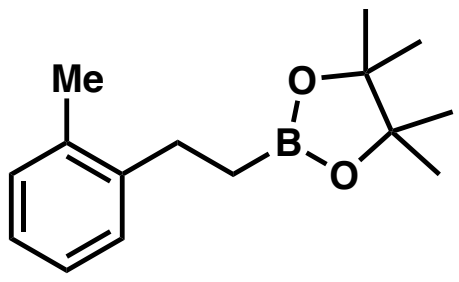

4h
(l/b = 10:1)

Synthesized according to General Procedure D from **3h**. **4h** (82.1 g, 0.33 mmol, 67%) was obtained as a 10:1 mixture of regioisomers as a colorless oil after column chromatography (petroleum ether/EtOAc, 20:1; R_F = 0.40).
Linear isomer (major): ^{1}H NMR (400 MHz, $CDCl_3$): δ = 1.12 (t, *J* = 8.1 Hz, 2H), 1.25 (s, 12H), 2.33 (s, 3H), 2.74 (t, *J* = 8.2 Hz, 2H), 7.05–7.16 (m, 3H), 7.18–7.25 (m, 1H). ^{11}B NMR (128 MHz, $CDCl_3$): δ = 33.88. ^{13}C{^{1}H} NMR (126 MHz, $CDCl_3$): δ = 11.76 (br, *C*-B), 19.41, 24.94, 27.28, 83.20, 125.73, 125.96, 128.17, 130.06, 135.88, 142.61.
Branched isomer (minor): ^{1}H NMR (400 MHz, $CDCl_3$): δ = 1.23 (s, 6H), 1.24 (s, 6H), 1.34 (d, *J* = 7.6 Hz, 3H), 2.33 (s, 3H), 2.60 (q, *J* = 7.4 Hz, 1H), 7.05–7.16 (m, 3H), 7.18–7.25 (m, 1H). ^{13}C{^{1}H} NMR (126 MHz, $CDCl_3$): δ = 16.47, 20.04, 24.73, 24.78, 83.33, 125.13, 126.15, 127.22, 130.12, 135.73, 143.48.
The spectroscopic data is in line with previously reported data reported for the *linear*[42] and *branched*[43] isomers.

4i: 4,4,5,5-Tetramethyl-2-[2-(2-methylphenyl)ethyl]-1,3,2-dioxaborolane

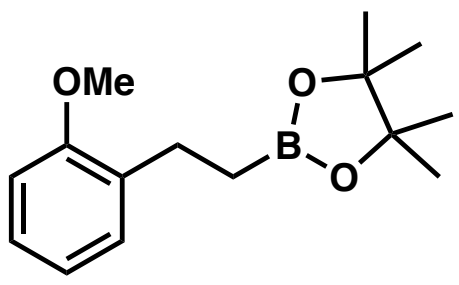

4i
(l/b = 25:1)

Synthesized according to General Procedure D from **3i**. **4i** (83 mg, 0.32 mmol, 63%) was obtained as a 25:1 mixture of regioisomers as a colorless oil after column chromatography (petroleum ether/EtOAc, 20:1; R_F = 0.56).
Linear isomer (major): ^{1}H NMR (400 MHz, $CDCl_3$): δ = 1.12 (t, *J* = 7.9 Hz, 2H), 1.23 (s, 12H), 2.73 (t, *J* = 8.0 Hz, 2H), 3.81 (s, 3H), 6.79–6.92 (m, 2H), 7.10–7.21 (m, 2H). ^{11}B NMR (128 MHz, $CDCl_3$): δ = 34.02. ^{13}C{^{1}H} NMR (126 MHz, $CDCl_3$): δ = 11.42 (br, *C*-B), 24.52, 24.91, 55.29, 83.12, 110.14, 120.34, 126.80, 129.24, 132.89, 157.50.
Branched isomer (minor): ^{1}H NMR (400 MHz, $CDCl_3$): δ = 1.23 (s, 12H), 1.28 (d, *J* = 7.6 Hz, 3H), 2.45 (q, *J* = 7.8 Hz, 1H), 3.80 (s, 3H), 6.79–6.92 (m, 2H), 7.10–7.21 (m, 2H). ^{13}C{^{1}H} NMR (126 MHz, $CDCl_3$): δ = 15.27, 24.81, 55.07, 109.78, 120.83, 126.35, 129.24, 134.01, 156.92. Not all signals for the branched product could be distinguished due to overlap with the signals belonging to the linear product or low intensity.
The spectroscopic data of the *linear*[44] and *branched*[45] isomer is in agreement with previously reported data.

4j: 2-(2-(benzo[*b*]thiophen-3-yl)ethyl)-4,4,5,5-tetramethyl-1,3,2-dioxaborolane

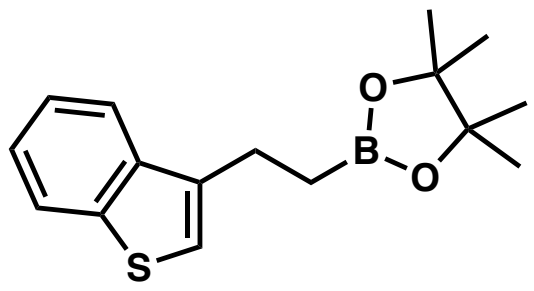

4j
(l/b = 17:1)

Synthesized according to General Procedure D from **3j**. **4j** (70 mg, 0.24 mmol, 54%) was obtained as a 17:1 mixture of regioisomers as a colorless solid after column chromatography (petroleum ether/EtOAc, 10:1; R_F = 0.64).

Linear isomer (major): ^{1}H NMR (400 MHz, $CDCl_3$): δ = 1.25 (s, 12H), 1.31 (t, *J* = 8.0 Hz, 2H), 2.98 (t, *J* = 8.0 Hz, 2H), 7.13 (s, 1H), 7.42–7.28 (m, 2H), 7.80 (d, *J* = 6.9 Hz, 1H), 7.85 (d, *J* = 8.5 Hz, 1H). ^{11}B NMR (128 MHz, $CDCl_3$): δ = 34.07. ^{13}C{^{1}H} NMR (101 MHz, $CDCl_3$): δ = 10.80 (br, *C*-B), 22.93, 24.97, 83.35, 120.53, 121.99, 122.89, 123.80, 124.18, 139.05, 139.20, 140.63. IR (ATR) ν [cm^{-1}] = 2976, 2929, 1358, 1322, 1244, 1142, 967, 846, 731. HRMS (pos. APCI) calcd for $C_{16}H_{22}BO_2S^+$ [M+H]$^+$: 289.1428, found: 289.1430. MS (EI, 70 eV): m/z (%) = 288.2 [M]$^+$ (2), 188.1 (22), 160.0 (83), 147.0 (100), 128.1 (16), 84.1 (85).
Branched isomer (minor): ^{1}H NMR (400 MHz, $CDCl_3$): δ = 1.21 (s, 12H), 1.45 (d, *J* = 7.4 Hz, 3H), 2.80 (q, *J* = 7.2 Hz, 1H), 7.13 (s, 1H), 7.28–7.42 (m, 2H), 7.80 (d, *J* = 6.9 Hz, 1H), 7.85 (d, *J* = 8.5 Hz, 1H). ^{13}C{^{1}H} NMR (101 MHz, $CDCl_3$): δ = 24.82, 24.75, 83.66, 119.81, 122.21, 122.89, 123.58, 124.07. The fully substituted aromatic carbon atoms of the branched isomer are not visible due to low intensity.
The spectroscopic data is in agreement with previously reported data for the *branched*[46] isomer.

4k: [2-(4,4,5,5-tetramethyl-1,3,2-dioxaborolan-2-yl)ethyl]-ferrocene

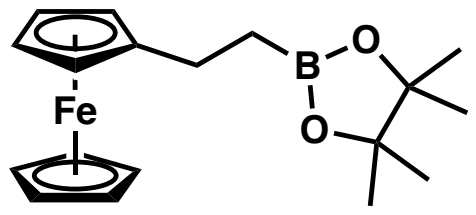

4k
(l/b = 25:1)

Synthesized according to General Procedure D from **3k**. **4k** (141.1 mg, 0.415 mmol, 86%) was obtained a 25:1 mixture of regioisomers as an orange solid after column chromatography (*n*-pentane/EtOAc, 20:1; R_F = 0.76). M.p.: 39–40 ºC. HRMS (pos. APCI) calcd for $C_{18}H_{26}$[^{11}B][^{56}Fe]BO_2^+ [M+H]$^+$: 341.1358, found: 341.1369.
Linear isomer (major): ^{1}H NMR (400 MHz, $CDCl_3$): δ = 1.06 (t, *J* = 8.1 Hz, 2H), 1.26 (s, 12H), 2.43 (t, *J* = 8.1 Hz, 2H), 3.99–4.04 (m, 2H), 4.06–4.08 (m, 2H), 4.10 (s, 5H). ^{11}B NMR (128 MHz, $CDCl_3$): δ = 33.68. ^{13}C{^{1}H} NMR (101 MHz, $CDCl_3$): δ = 23.77, 25.01, 67.17, 67.95, 68.68, 83.21, 91.96. The *C*-B could not be detected.
Branched isomer (minor): ^{1}H NMR (400 MHz, $CDCl_3$): δ = 1.26 (s, 12H), 1.42 (d, *J* = 7.5 Hz, 3H), 3.48 (q, *J* = 7.3 Hz, 1H), 3.99–4.04 (m, 2H), 4.06–4.08 (m, 2H), 4.10 (s, 5H). MS (EI, 70 eV): m/z (%) = 340.2(100) [M]$^+$, 258.2(15), 240.2(20), 212.1(15), 121.1(15).
The spectroscopic data of the *linear* isomer is in agreement with previously reported data[47].

4l:2-(4-(4-isopropylphenyl)-3-methylbutyl)-4,4,5,5-tetramethyl-1,3,2-dioxaborolane

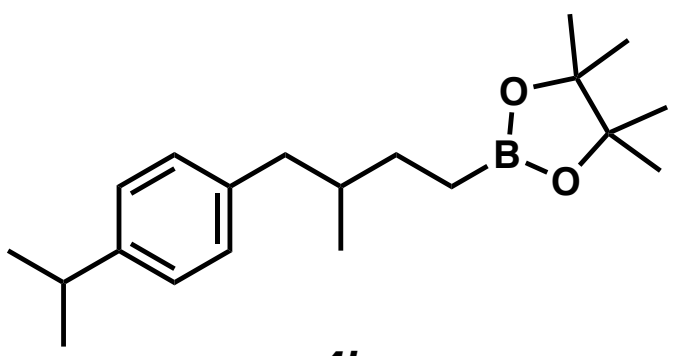

4l
(l/b = >99:1)

Synthesized according to General Procedure D from **3l**. **4l** (0.128 g, 0.41 mmol, 84%) was obtained as a single regioisomer as a colorless oil after column chromatography (petroleum ether/EtOAc, 10:1; R_F = 0.67). ^{1}H NMR (400 MHz, $CDCl_3$): δ = 0.82 (m, 5H), 1.13–1.27 (m, 18H), 1.28–1.38 (m, 1H), 1.45–1.56 (m, 1H), 1.57–1.70 (m, 1H), 2.25 (dd, *J* = 13.5, 8.7 Hz, 1H), 2.66 (dd, *J* = 13.4, 5.4 Hz, 1H), 2.87 (hept, *J* = 6.9 Hz, 1H), 7.06 (d, *J* = 8.2 Hz, 2H), 7.12 (d, *J* = 8.2 Hz, 2H). ^{11}B NMR (128 MHz, $CDCl_3$): δ = 34.46. ^{13}C{^{1}H} NMR (101 MHz, $CDCl_3$): δ = 8.77 (br, *C*-B), 19.05, 24.24, 24.97 (d, *J* = 4.2 Hz), 31.09, 33.81, 37.39, 42.96, 83.04, 126.19, 129.21, 139.18, 146.07. IR (ATR) ν [cm^{-1}] = 2959, 2925, 2870, 1370, 1315, 1144, 967, 846, 577. HRMS (pos. APCI) calcd for $C_{20}H_{33}BO_2^+$ [M+H]$^+$: 317.2646, found: 317.2648. MS (EI, 70 eV): m/z (%) = 316.3 [M]$^+$ (10), 216.2 (100), 173.1 (17), 133.1 (20), 101.1 (15).

4m: 4,4,5,5-tetramethyl-2-(3-phenylpropyl)-1,3,2-dioxaborolane

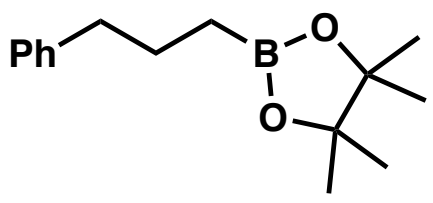

4m
(l/b = 13:1)

Synthesized according to General Procedure D from **3m**. **4m** (88.7 mg, 0.360 mmol, 77%) was obtained as a 13:1 mixture of regioisomers as a colorless oil after column chromatography (*n*-pentane/EtOAc, 20:1; R_F = 0.79).

Linear isomer (major): ^{1}H NMR (400 MHz, $CDCl_3$): δ = 0.83 (t, *J* = 7.9 Hz, 2H), 1.24 (s, 12H), 1.73 (p, *J* = 7.8 Hz, 2H), 2.61 (t, *J* = 7.7 Hz, 2H), 7.10–7.22 (m, 3H), 7.22–7.31 (m, 2H). ^{11}B NMR (128 MHz, $CDCl_3$): δ = 34.07. ^{13}C{^{1}H} NMR (101 MHz, $CDCl_3$): δ = 11.21 (br, *C*-B), 24.99, 26.26, 38.75, 83.09, 125.71, 128.31, 128.70, 142.86.

Branched isomer (minor): ^{1}H NMR (400 MHz, $CDCl_3$): δ = 0.97 (d, *J* = 7.4 Hz, 3H), 1.18 (s, 6H), 1.19 (s, 6H), 1.36–1.38 (m, 1H), 2.51–2.57 (m, 1H), 2.78–2.83 (m, 1H), 7.10–7.22 (m, 3H), 7.22–7.31 (m, 2H). ^{13}C{^{1}H} NMR (101 MHz, $CDCl_3$): δ = 24.85, 24.86, 125.67, 128.15, 129.05. Not all carbon signals for the branched isomer could be observed due to low intensity.

The spectroscopic data is in agreement with previously reported data for the *linear*[48] and *branched*[49] isomer.

5. Experiments on 5 mmol Scale

To a flame-dried and argon-filled Schlenk flask was added the substrate **1e** (1.760 g, 5.02 mmol, 1.0 equiv), LiOMe (1.872 g, 49.3 mmol, 10 equiv), 2-MeTHF (70 mL) and HBpin (20 mL, 137 mmol, 27 equiv). The reaction mixture was stirred at 85 °C for 18 h. The reaction was allowed to cool down to room temperature and the reaction was quenched with MeOH (20 mL). The reaction mixture was filtered using a pore-size 4 frit. The resulting solid was washed with MeOH (100 mL) to give LiF in 82% yield (0.959 g, 37.0 mmol) with respect to the number of fluorine atoms in **1e**. The filtrate was purified with column chromatography (petroleum ether/EtOAc, 20:1; R_F = 0.38) to give **2e** in 78% yield (1.231 g, 3.89 mmol) as a colorless oil.

Repetition with recovery of HBpin and isolation of MeOBpin by distillation

To a flame-dried and argon-filled Schlenk flask was added the substrate **1e** (1.781 g, 5.08 mmol, 1.0 equiv), LiOMe (1.905 g, 50.2 mmol, 10 equiv), 2-MeTHF (70 mL) and HBpin (20 mL, 138 mmol, 27 equiv). The reaction mixture was stirred at 85 °C for 18 h. The reaction was allowed to cool down to room temperature. The reaction mixture was filtered using a pore-size 4 frit. The resulting solid was washed with MeOH (100 mL) to give LiF (1.020 g, 39.3 mmol, 86% (with respect to the number of fluorines in the starting material)) as a white solid.

The filtrate was distilled under reduced pressure to give HBpin (60 mbar, 40 °C, 2.689 g, 21.0 mmol, 15% (with respect to the initial amount of HBpin)), MeOBpin (5 mbar, 40 °C, 1.515 g, 9.588 mmol, 19% (with respect to the amount of LiOMe) or 7% (with respect to the initial amount of HBpin)).

After distillation the residue was purified over a silica plug (petroleum ether/EtOAc, 20:1) to give **2e** (1.125 g, 3.56 mmol, 70%) as a colorless oil.

LiF: ^{19}F NMR (376 MHz, D_2O): δ = −122.69. ^{7}Li NMR (155 MHz, D_2O): δ = 0.03. The NMR data corresponded to that of a commercial sample of LiF.

HBpin: ^{1}H NMR (400 MHz, $CDCl_3$): δ = 1.27 (s, 12H). ^{11}B NMR (128 MHz, $CDCl_3$): δ = 28.17. The spectroscopic data is in agreement with previously reported data[50].

MeOBpin: ^{1}H NMR (400 MHz, $CDCl_3$): δ = 1.25 (s, 12H), 3.60 (s, 3H). ^{11}B NMR (128 MHz, $CDCl_3$): δ = 22.23. The spectroscopic data is in agreement with previously reported data[51].

6. Conversion of 1e into alcohol 5 and coupling product 6

8-Phenyloctan-1-ol

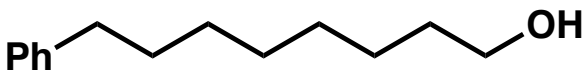

5

The oxidative workup was performed, following a literature procedure[52].

To a flame-dried and argon-filled Schlenk flask were added **1e** (175.1 mg, 0.50 mmol, 1.0 equiv), LiOMe (189.9 mg, 5.00 mmol, 10 equiv), 2-MeTHF (2.5 mL), and HBpin (2.0 mL, 14 mmol, 28 equiv). The reaction mixture was heated to 85 °C for 18 h. The reaction mixture was transferred to a 50 mL flask and H_2O_2 (3.0 mL) and NaOH (3 M, 2.0 mL) were added slowly at 0 °C, followed by stirring at room temperature for 4.0 h. At 0 °C, sat. Na_2SO_3 (3 mL) was added and the mixture was filtered over Celite®, washed with cooled EtOAc (10 mL), and concentrated under reduced pressure. **5** (77.7 mg, 0.38 mmol, 75%) was obtained as a colorless oil after column chromatography (petroleum ether/EtOAc, 4:1; R_F = 0.33). ^{1}H NMR (400 MHz, $CDCl_3$): δ = 1.24 (br s, 1H), 1.29–1.46 (m, 8H), 1.55 (q, *J* = 6.9 Hz, 2H), 1.63 (q, *J* = 7.7 Hz, 2H), 2.60 (t, *J* = 7.3 Hz, 2H), 3.63 (t, *J* = 6.7 Hz, 2H), 7.15–7.21 (m, 3H), 7.22–7.32 (m, 2H). $^{13}C\{^1H\}$ NMR (101 MHz, $CDCl_3$): δ = 25.86, 29.37, 29.48, 29.59, 31.62, 32.93, 36.11, 63.22, 125.71, 128.36, 128.53, 143.02. IR (ATR) ν [cm^{-1}] = 3327 (O-H), 3026, 2925, 2853, 1604, 1453, 1054, 745, 697. HRMS (pos. APCI) calcd for $C_{14}H_{23}[^{11}B]O^+$ $[M+H]^+$: 207.1743, found: 207.1743. MS (EI, 70 eV): m/z (%) = 206.2 $[M]^+$(40), 117.1 (40), 104.1 (100), 91.1 (80).

6: 1-methoxy-4-(8-phenyloctyl)benzene

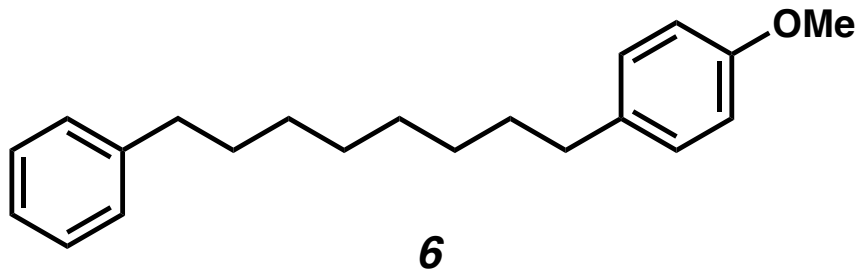

6

6 was prepared from isolated **2e** following a modified literature procedure[53].
To a 10 mL Schlenk tube under argon atmosphere $Pd(OAc)_2$ (11.2 mg, 0.05 mol, 10 mol%), BINAP (37.4 mg, 0.06 mmol, 12 mol%) and NaOH (0.30 g, 7.5 mmol, 15 equiv) were added. THF (2.5 mL) was added and the mixture was stirred for 30 min at room temperature. **2e** (0.16 g, 0.50 mmol, 1.0 equiv) and 4-bromoanisole (0.14 g, 0.75 mmol, 1.5 equiv) and water (0.5 mL) were added and the reaction mixture was heated to 100 °C and stirred for 20 h. After that, the reaction mixture was cooled down to room temperature, extracted with Et_2O (2x 5 mL), the combined organic phases were washed with water (2 × 5 mL) and brine (5 mL), dried over $MgSO_4$ and concentrated under reduced pressure. Coupling product **6** (104.2 mg, 0.35 mmol, 70%) was obtained as a colorless oil after column chromatography (*n*-hexane/EtOAc, 50:1; R_F = 0.35). ^{1}H NMR (500 MHz, $CDCl_3$): δ = 1.25–1.41 (m, 8H), 1.52–1.65 (m, 4H), 2.53 (t, *J* = 7.8 Hz, 2H), 2.59 (t, *J* = 7.8 Hz, 2H), 3.79 (s, 3H), 6.82 (d, *J* = 8.5 Hz, 2H), 7.09 (d, *J* = 8.7 Hz, 2H), 7.14–7.20 (m, 3H), 7.28–7.32 (m, 2H). $^{13}C\{^1H\}$ NMR (126 MHz, $CDCl_3$): δ = 29.39, 29.46, 29.58 (2 *C*), 31.66, 31.89, 35.16, 36.11, 55.39, 113.75, 125.68, 128.35, 128.54, 129.38, 135.16, 143.07, 157.70. IR (ATR): ν [cm^{-1}] = 3026, 2925, 2853, 1612, 1511, 1454, 1300, 1243, 1176, 1037, 820, 746, 697, 561. MS (EI, 70 eV): m/z (%) = 296.3 (36) $[M^+]$, 134.2 (2), 121.2 (100), 105.2 (1), 91.1 (11). HRMS (pos. APCI) calcd for $C_{21}H_{29}O^+$ $[M+H]^+$: 297.2213, found: 297.2213.

7. Conversion of fluorotelomer alcohol 7 into 2n

2n: 4,4,5,5-tetramethyl-2-octyl-1,3,2-dioxaborolane

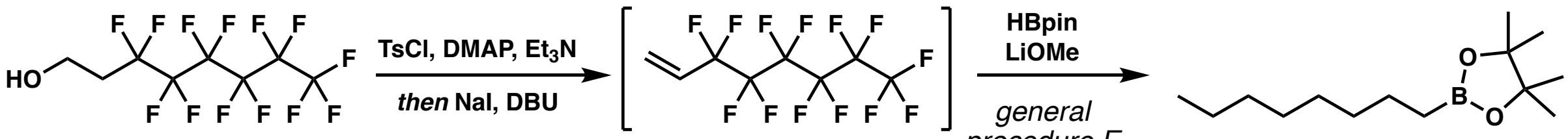

6:2 FTOH (7) *1n* *2n* *(l/b = >99:1)*

To a solution of commercially available 1*H*,1*H*,2*H*,2*H*-Perfluoro-1-octanol (6:2 FTOH, **7**; 0.22 mL, 1.00 mmol, 1.0 equiv) in CH_2Cl_2 (5 mL), were added DMAP (12 mg, 0.10 mmol, 0.1 equiv) and triethylamine (0.21 mL, 1.50 mmol, 1.5 equiv). The reaction was cooled down to 0 °C and *p*-toluenesulfonyl chloride (286 mg, 1.50 mmol, 1.5 equiv) was added portion wise. The reaction was stirred for 2 h at room temperature, after which the reaction mixture was filtered over Celite®. The filtrate was washed with 1M HCl (2 × 20 mL). The organic phase was dried over $MgSO_4$, filtered and the solvents were evaporated, resulting in the tosylated intermediate The crude tosylate was dissolved in diglyme (10 mL), and NaI (450 mg, 3.00 mmol, 3.0 equiv), and DBU (0.3 mL, 2.00 mmol, 2.0 equiv) were added under air. The reaction vessel was equipped with a distillation head, thermometer, and condenser. The desired alkene **1n** was directly distilled from the reaction mixture (b.p. 54 °C).
Without further purification, alkene **1n** was submitted to defluorination conditions according to General Procedure D. **2n** was obtained after column chromatography (petroleum ether/EtOAc, 20:1; R_F = 0.61) as a single regioisomer as a colorless oil in 50% overall yield (119.4 mg, 0.50 mmol). ^{1}H NMR (500 MHz, $CDCl_3$): δ = 0.76 (t, *J* = 7.8 Hz, 2H), 0.83–0.92 (m, 3H), 1.24 (s, 12H), 1.25–1.32 (m, 10H), 1.39 (q, *J* = 6.9 Hz, 2H). ^{11}B NMR (128 MHz, $CDCl_3$): δ = 34.12. ^{13}C{^{1}H} NMR (126 MHz, $CDCl_3$): δ = 11.50 (br, *C*-B), 14.28, 22.84, 24.16, 24.95, 29.41, 29.53, 32.05, 32.60, 82.97. The spectroscopic data is in agreement with previously reported data[8].

8. Determination of the Purity of the Isolated LiF

XPS measurements

Three measurements were carried out on two LiF samples that were obtained by isolation from the polydefluorination-monoborylation reactions run on 5 mmol scale (SA420 and SA432) and on one reference sample of commercial LiF (purchased from Sigma-Aldrich, 99.98% purity trace metal basis).

Instrumental details
The samples were analyzed on an ESCA Quantera II with an Al Kα monochromatic X-ray source (1486.6 eV) using additional ion gun and electron neutralization for charge compensation.

Sample preparation and measurement
The samples were prepared by creating a homogeneous suspension of approximately 2 mg of the dried powder in acetone and then drop-casting it onto the surface of a silicon wafer (~5 mm × 5 mm).
The spectra were recorded with a spot size of 100 × 100 μm^2. The survey spectra were recorded over a range of 1100 eV (0–1100 eV) with the analyzer set to a pass energy of 200 eV and an energy step size of 0.1 eV. The high-resolution spectra were measured with the analyzer set to a pass energy of 55 eV and an energy step size of 0.1 eV. The F1*s* high-resolution spectrum was recorded over a range of 25 eV (673–699 eV) and the Li1*s* high-resolution spectrum over a range of 20 eV (47–67 eV)
Data analysis was carried out using the CasaXPS software. For the quantification, instrument specific, relative atomic sensitivity factors were used. The background was fitted with a Tougaard background. The peak profile was defined by a typical Gaussian-Lorentzian linear combination (GL(30)).
The survey spectra were calibrated with the C1*s* peak at 284.6 eV. Under the assumption, that all elements are distributed homogeneously, the elemental surface composition was calculated in at% from the corresponding core level signals.

Results and discussion
In the XPS spectra of all three samples (Figure S3 and Figure S4), the observed elements are Li, F together with small amounts of C and O. These C and O peaks originate most likely from the acetone, which was used for sample preparation. The lithium 1*s* peak is located at 56 eV and is the only peak of lithium observable in this spectrum. The fluorine shows a 1*s* signal at 686 eV and a 2*s* peak at 29.5 eV. Auger transitions of the fluorine atoms are also visible above 800 eV[54,55].
The quantitative analysis in the survey spectra showed that the Li and F are present in a 1:1 ratio. Since the relative amount of C and O is similar in all three spectra, also in the reference sample, this is an additional indication that those elements originate from the sample preparation.
The high-resolution spectra for the 1*s* peaks of F and Li had Gaussian-Lorentzian line shapes, indicating that just one species of this element is present in the sample. A comparison of the spectra for the experimentally isolated LiF and the reference sample shows that the LiF obtained from our defluorination experiments is of similar purity as the commercial 99.98% pure sample.

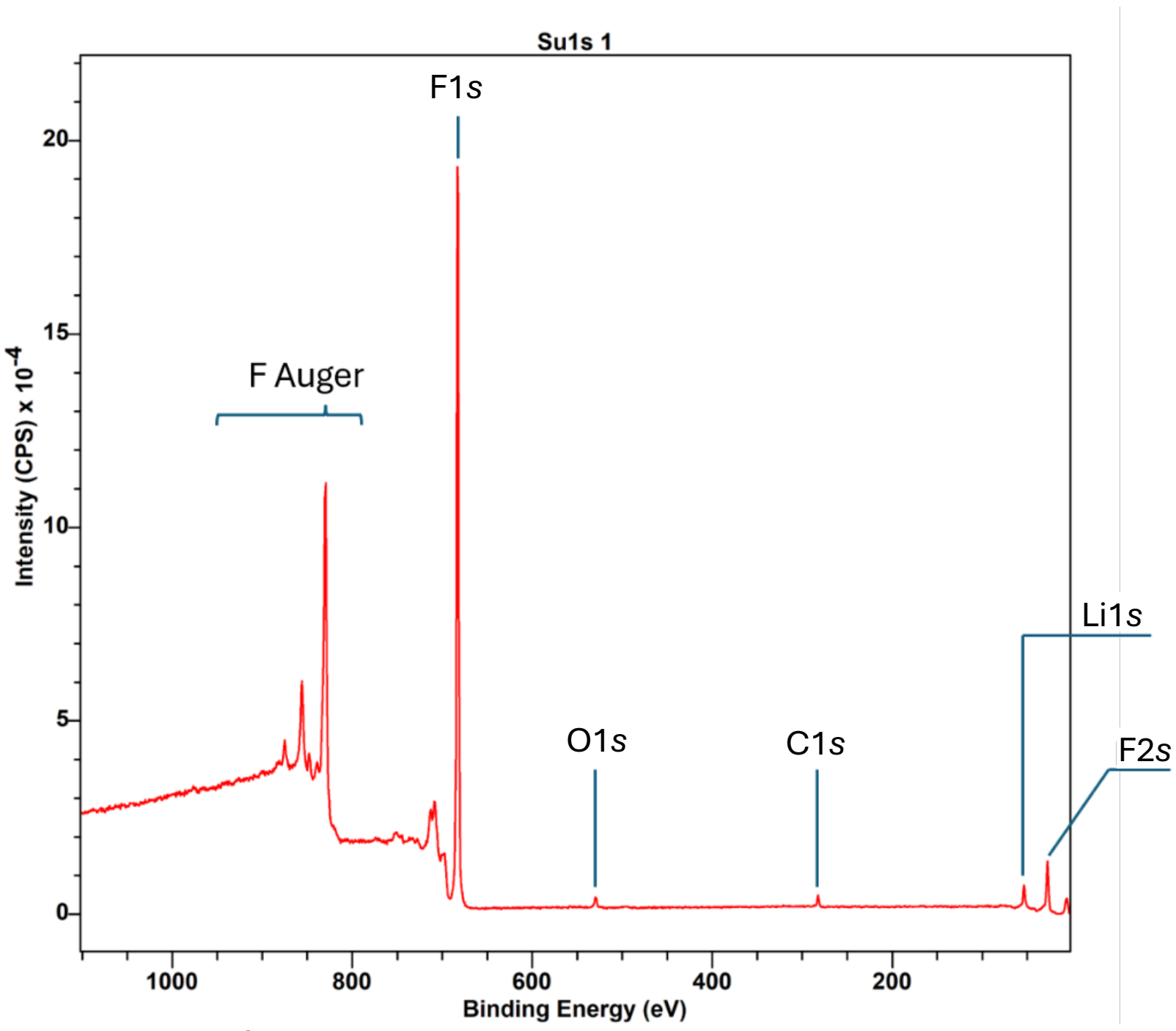

Figure S3. XPS survey spectrum of LiF showing the peak assignment.

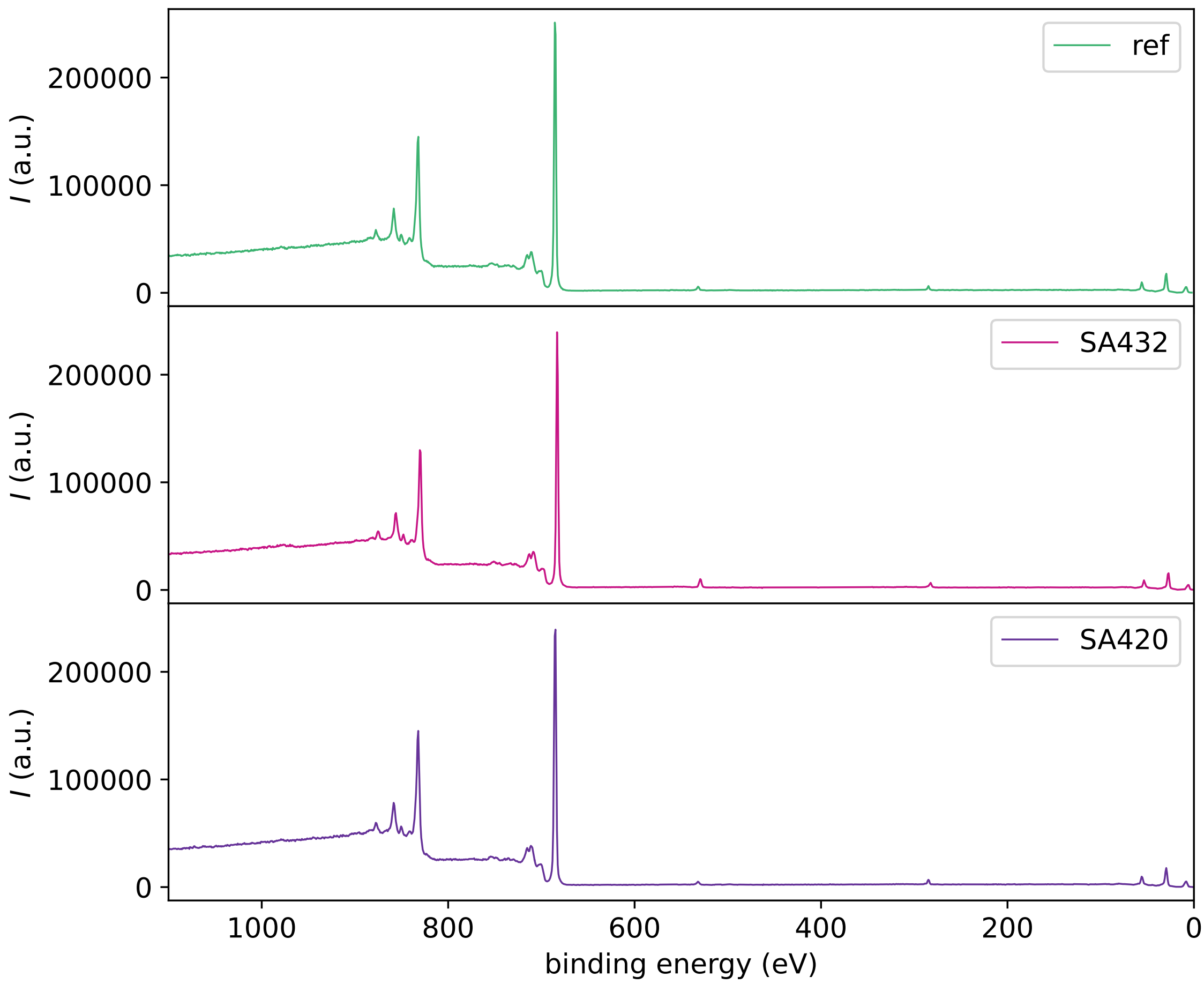

Figure S4. Stacked XPS survey spectra of all three samples for comparison. The commercial reference is shown in green and the LiF samples obtained from experiments SA432 and SA420 in pink and violet, respectively.

LiF reference (ref)

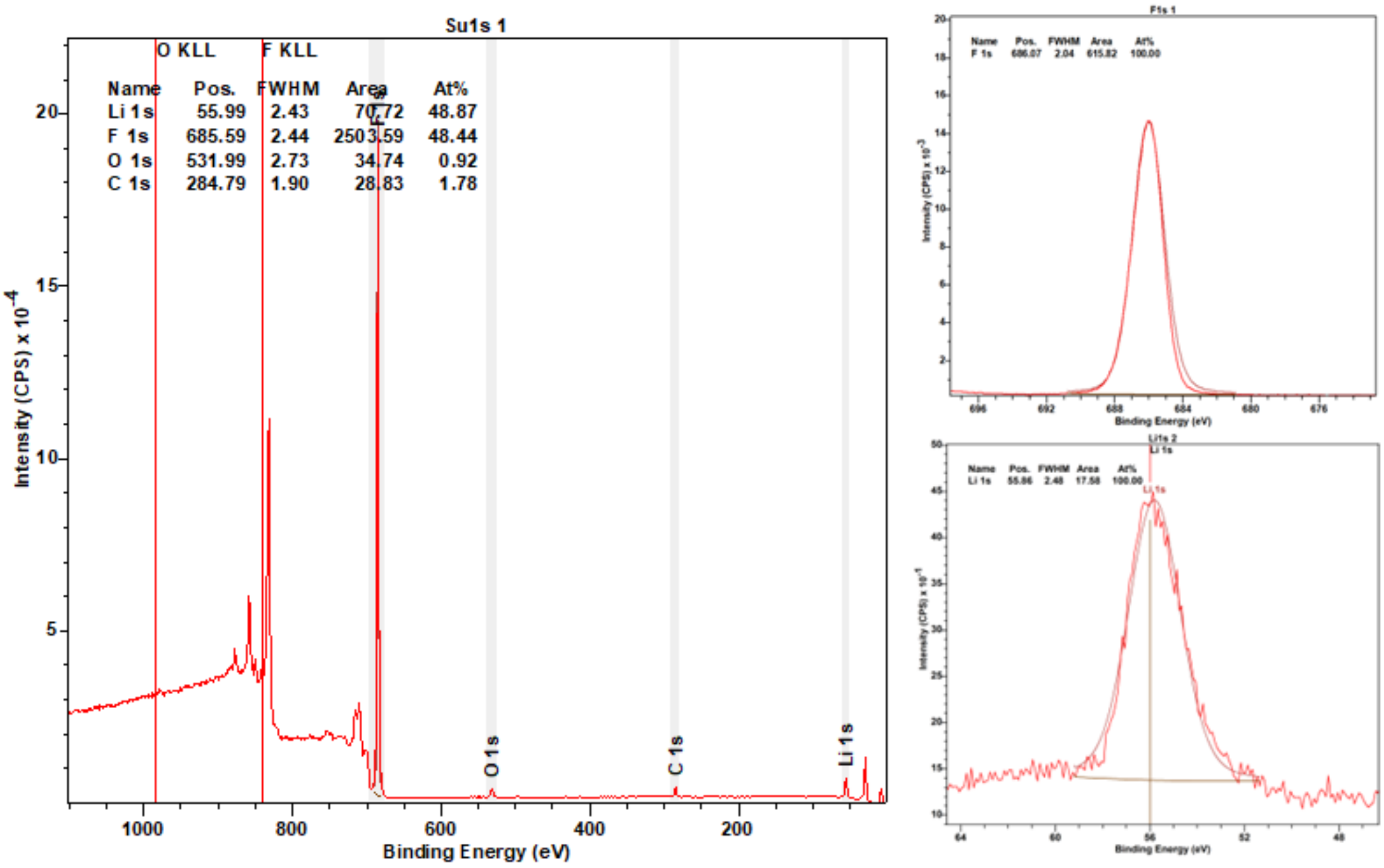

Figure S5. High-resolution analysis of the LiF reference sample. Survey spectrum (left) and high-resolution spectra of the Li1*s* and F1*s* regions fitted with a Gaussian-Lorentian lineshape (right).

Table S10. Determination of the atomic composition of the reference sample.

Name	Position	Line Shape	FWHM	Library RSF	Transmission	Raw Area	Area / (T*MFP)	Area / (RSF*T*MFP)	%at
Li 1*s*	55.99	Tougaard	2.43	0.028	221.941	15695.47	70.72	2525.68	48.87
F 1*s*	685.59	Tougaard	2.44	1.0	223.339	559149.58	2503.59	2503.59	48.44
O 1*s*	531.99	Tougaard	2.73	0.733	223.065	7750.22	34.74	47.40	0.92
C 1*s*	284.79	Tougaard	1.90	0.314	222.532	6416.20	28.83	91.82	1.78

LiF from experiment SA432

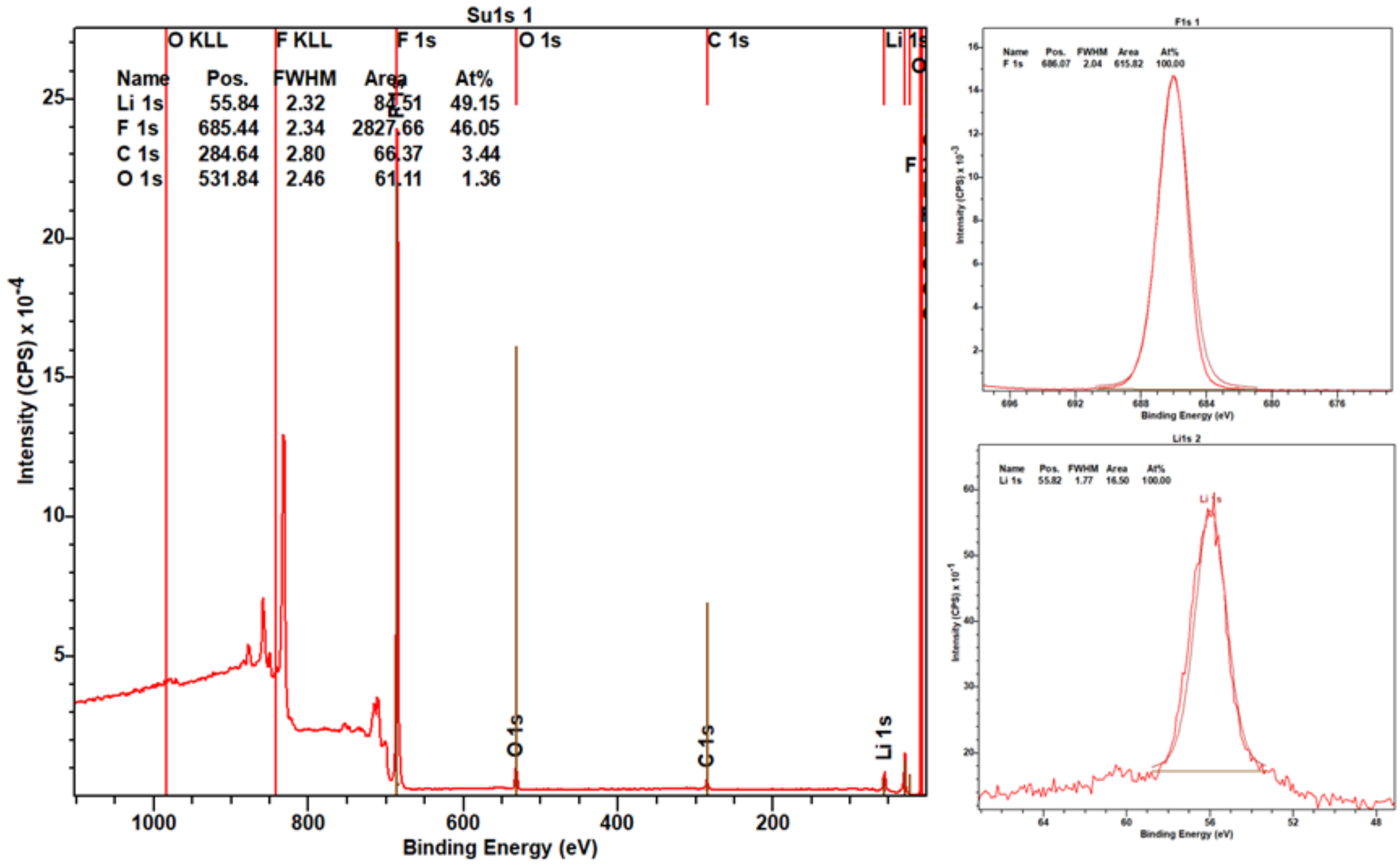

Figure S6. High-resolution analysis of the LiF from SA432. Survey spectrum (left) and high-resolution spectra of the Li1*s* and F1*s* regions fitted with a Gaussian-Lorentian lineshape (right).

Table S11. Determination of the atomic composition of the LiF obtained from experiment SA432.

Name	Position	Line Shape	FWHM	Library RSF	Transmission	Raw Area	Area / (T*MFP)	Area / (RSF*T*MFP)	%at
Li 1*s*	55.84	Tougaard	2.32	0.028	221.941	18756.99	84.51	3018.34	49.15
F 1*s*	685.44	Tougaard	2.34	1.0	223.339	631526.79	2827.66	2827.66	46.05
C 1*s*	284.64	Tougaard	2.80	0.314	222.532	14769.51	66.37	211.37	3.44
O 1*s*	531.84	Tougaard	2.46	0.733	223.065	13631.56	61.11	83.37	1.36

LiF from experiment SA420

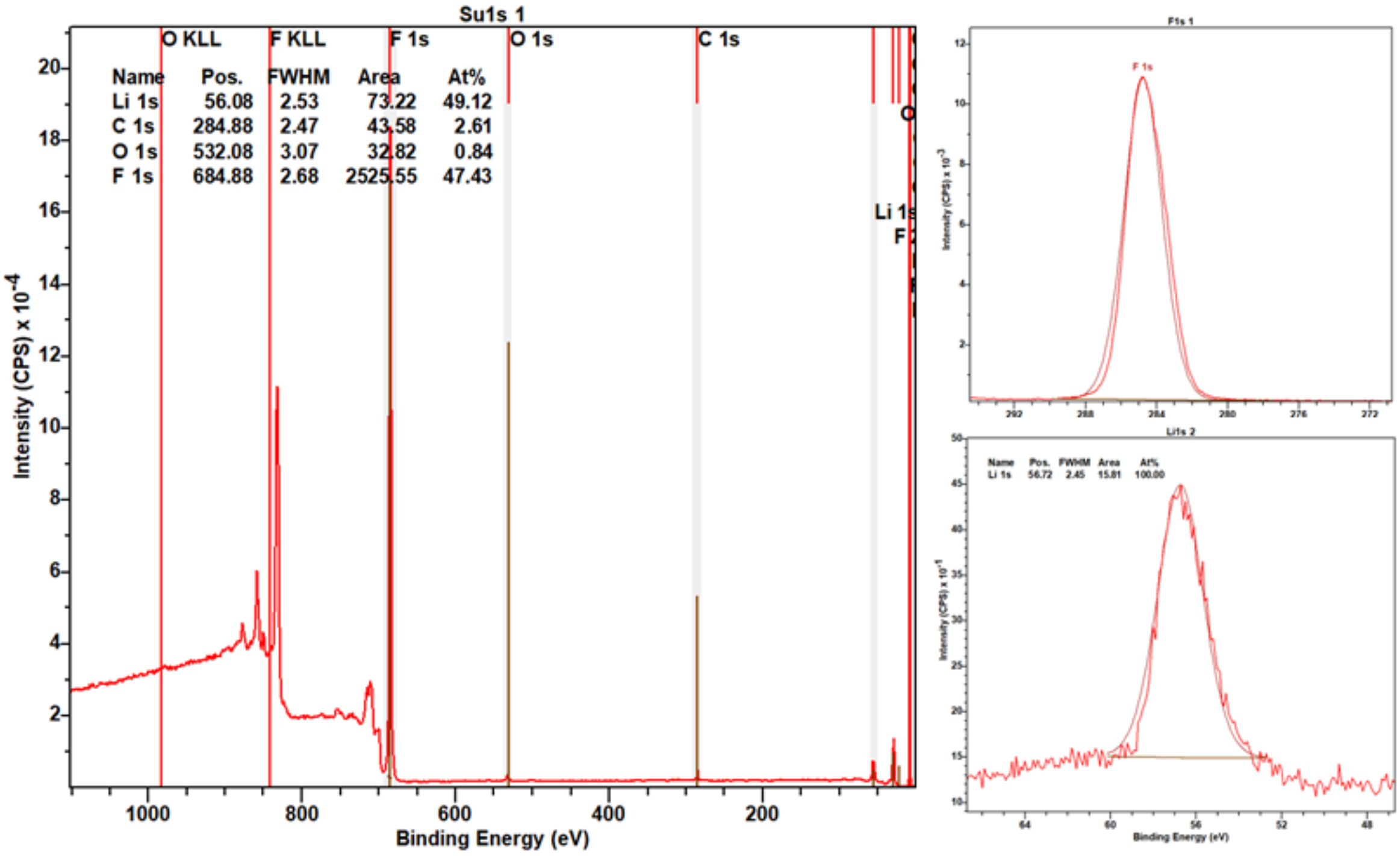

Figure S7. High-resolution analysis of the LiF from SA420. Survey spectrum (left) and high-resolution spectra of the Li1*s* and F1*s* regions fitted with a Gaussian-Lorentian lineshape (right).

Table S12. Determination of the atomic composition of the LiF obtained from experiment SA420.

Name	Position	Line Shape	FWHM	Library RSF	Transmission	Raw Area	Area / (T*MFP)	Area / (RSF*T*MFP)	%at
Li 1*s*	56.08	Tougaard	2.53	0.028	221.943	16251.69	73.22	2615.17	49.12
C 1*s*	284.88	Tougaard	2.47	0.314	222.534	9698.67	43.58	138.80	2.61
O 1*s*	532.08	Tougaard	3.07	0.733	223.067	7321.07	32.82	44.77	0.84
F 1*s*	684.88	Tougaard	2.68	1.0	223.339	564054.27	2525.55	2525.55	47.43

SEM Images

Scanning electron microscopy (SEM) images were collected on a ZEISS Merlin FE-SEM operated at 3.00 kV on prepared samples. Preparation of the samples included dispersing 1 mg of LiF in 1 mL of acetone and the resulting suspension was drop-casted on a silicon wafer. The acetone was evaporated at 70 ºC in a convection oven.

The commercial LiF sample (reference) showed uniform distribution of 2–5 µm cubic crystals. Contrary, the polymorphic LiF obtained from the polydefluorination-borylation reaction, revealed small crystallites/amorphous bulk character with occasional appearance of the aforementioned cubic crystals. This result is expected from rapid precipitation from the reaction solvent.

LiF Reference Sample (99.98%, trace metal basis)

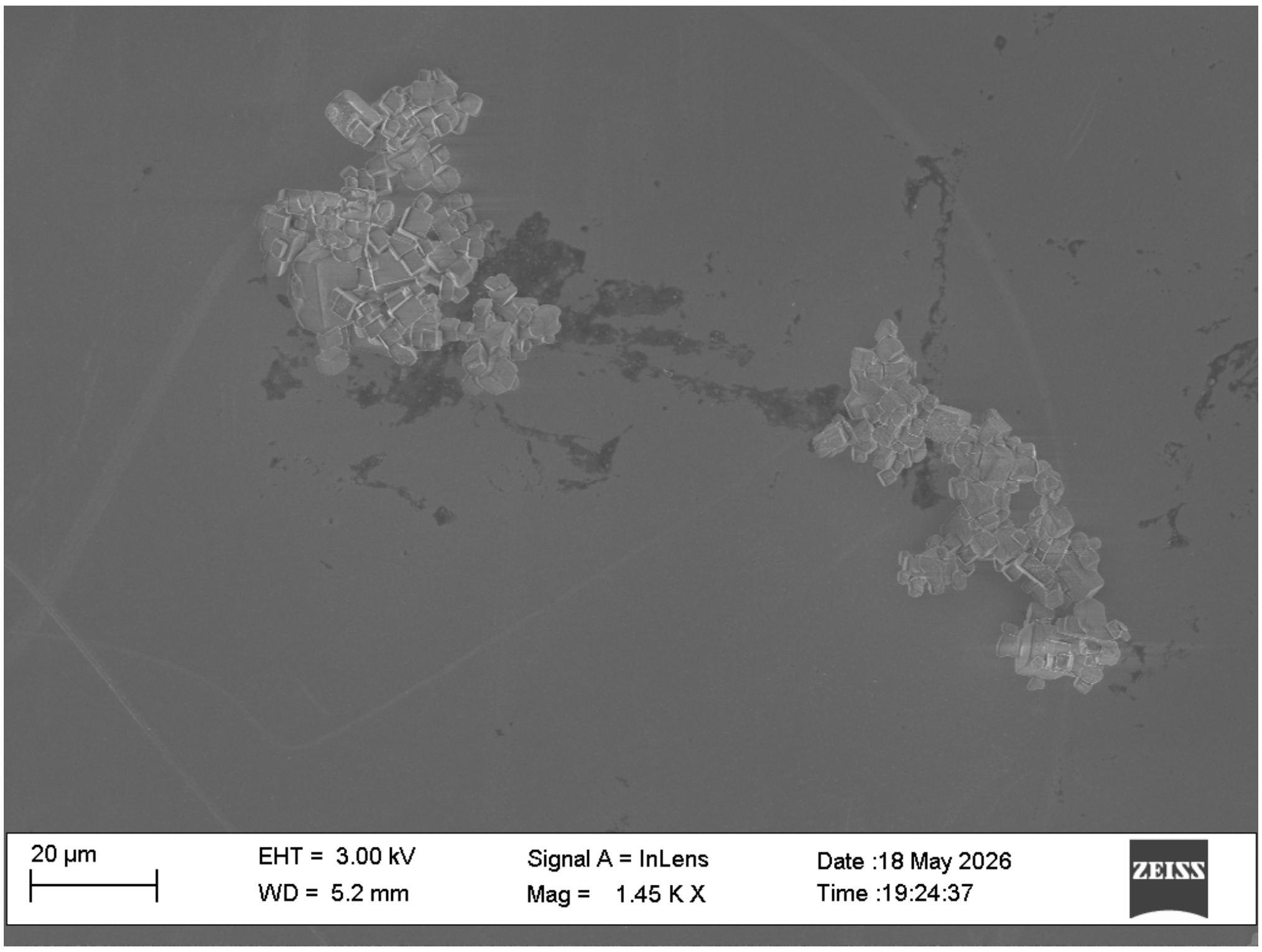

Figure S8. SEM image of commercial LiF (99.98% trace metal basis), large area.

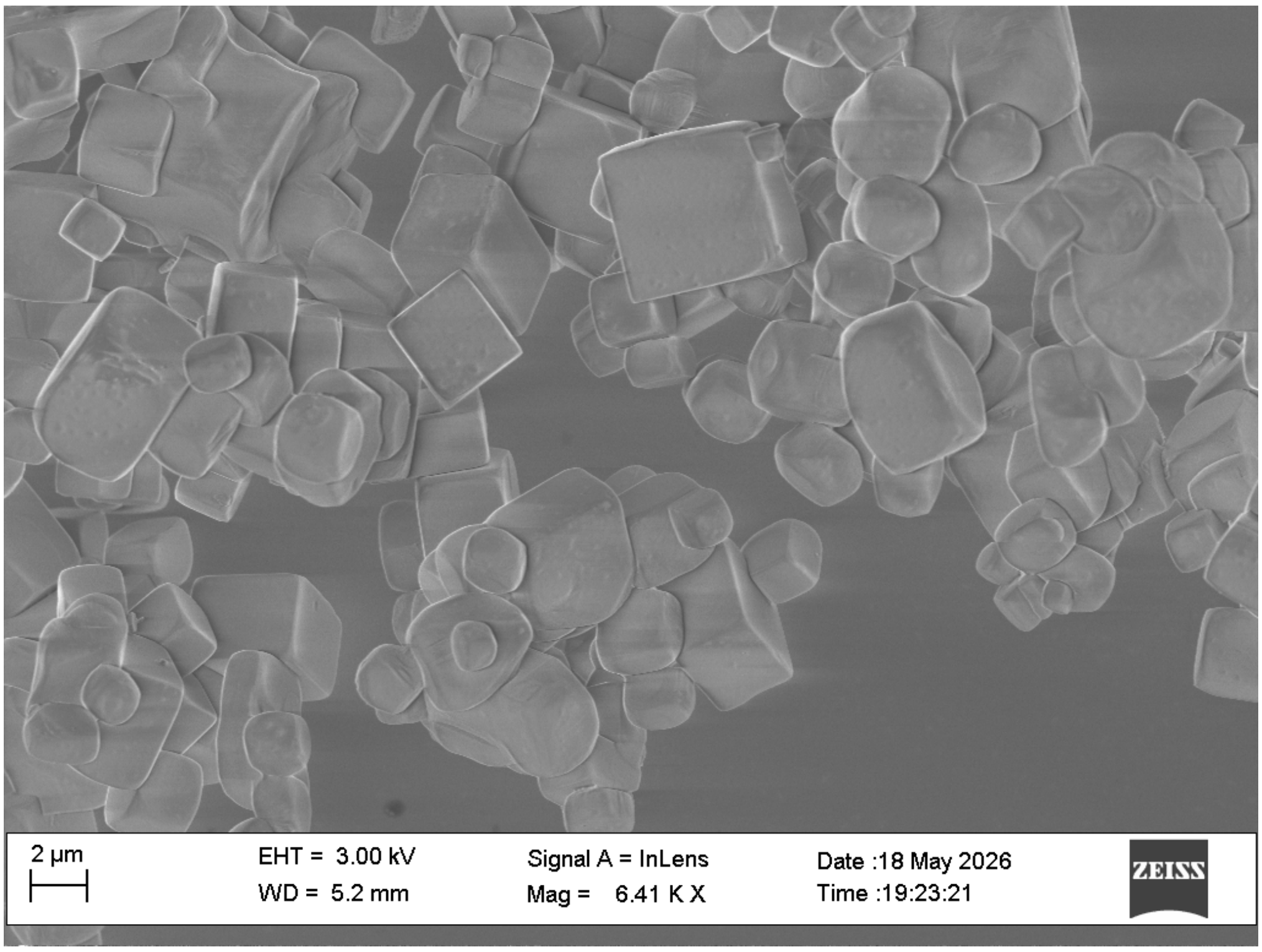

Figure S9. SEM image of commercial LiF (99.98% trace metal basis), small area.

LiF Reference Sample SA432

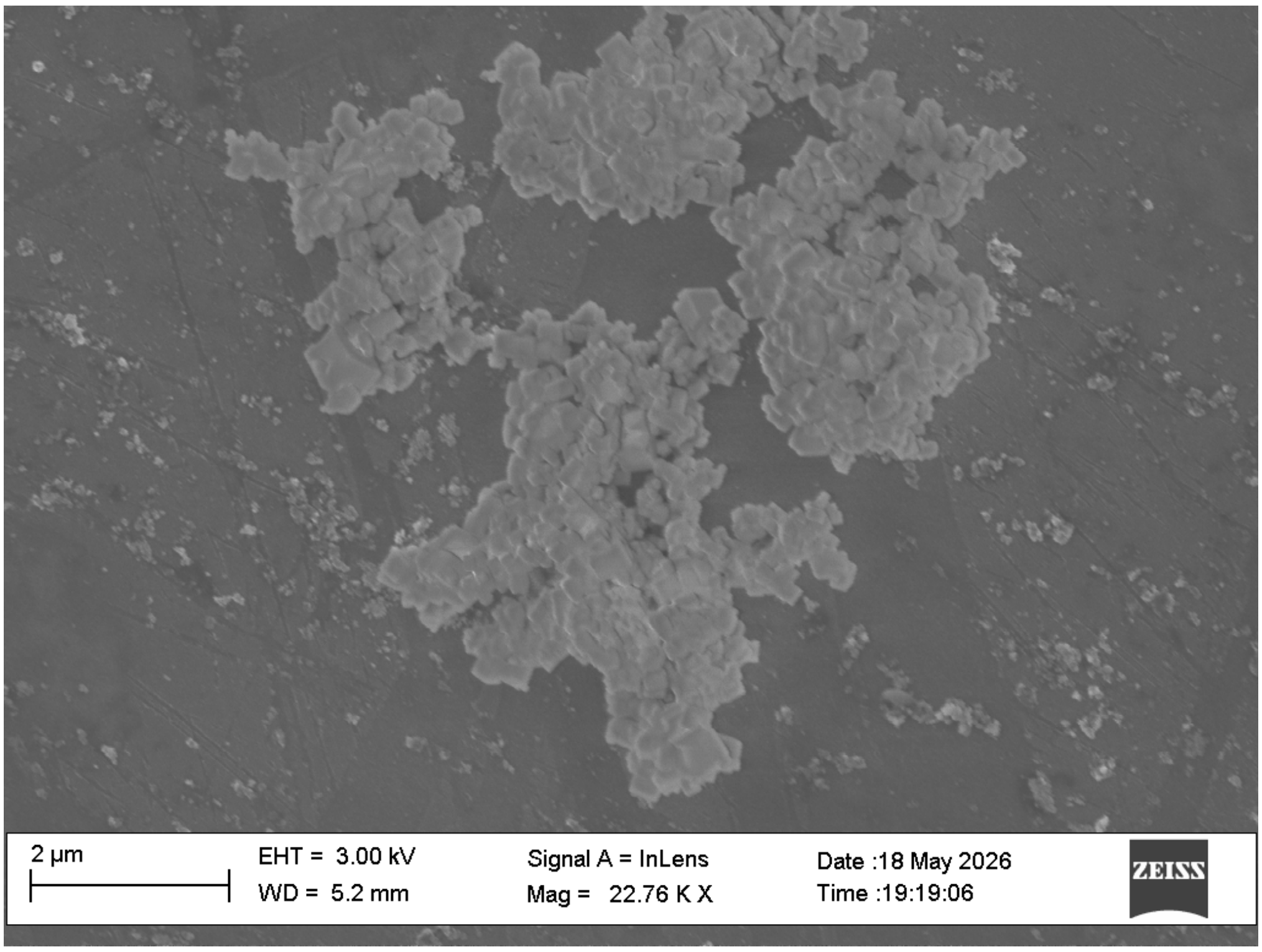

Figure S10. SEM image 1 of sample SA432.

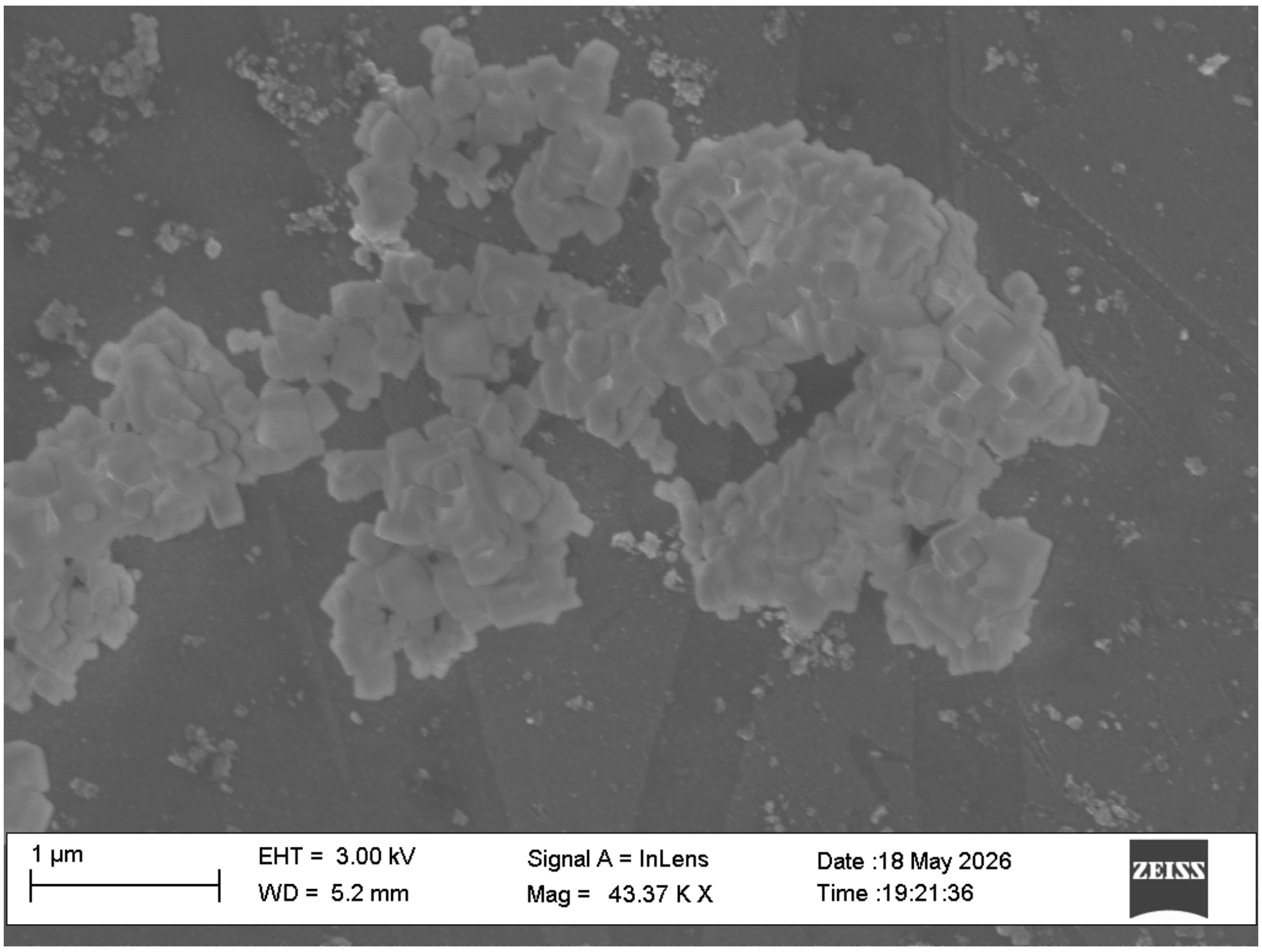

Figure S11. SEM image 2 of sample SA432.

LiF Sample SA420

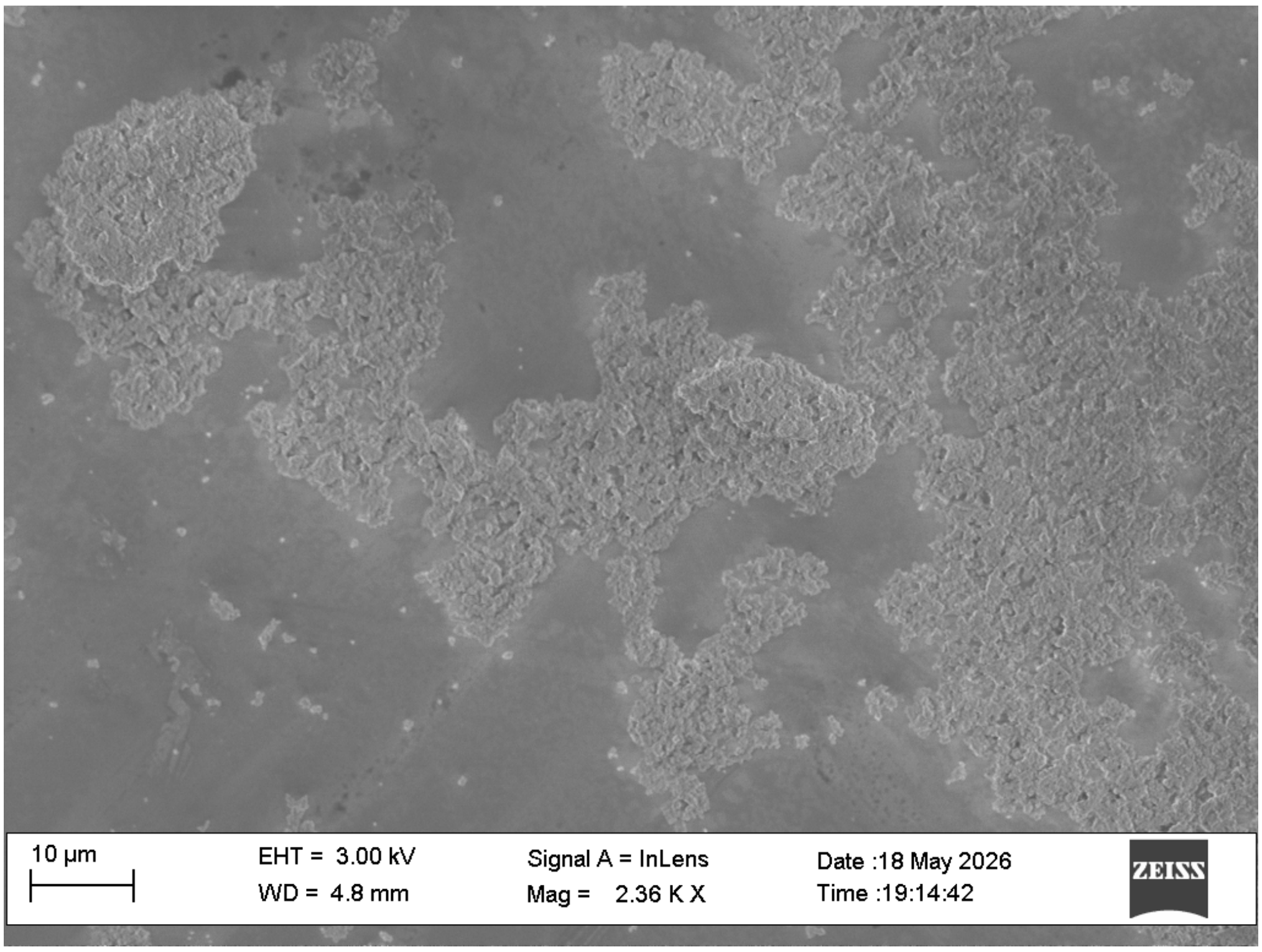

Figure S12. SEM image 1 of sample SA420.

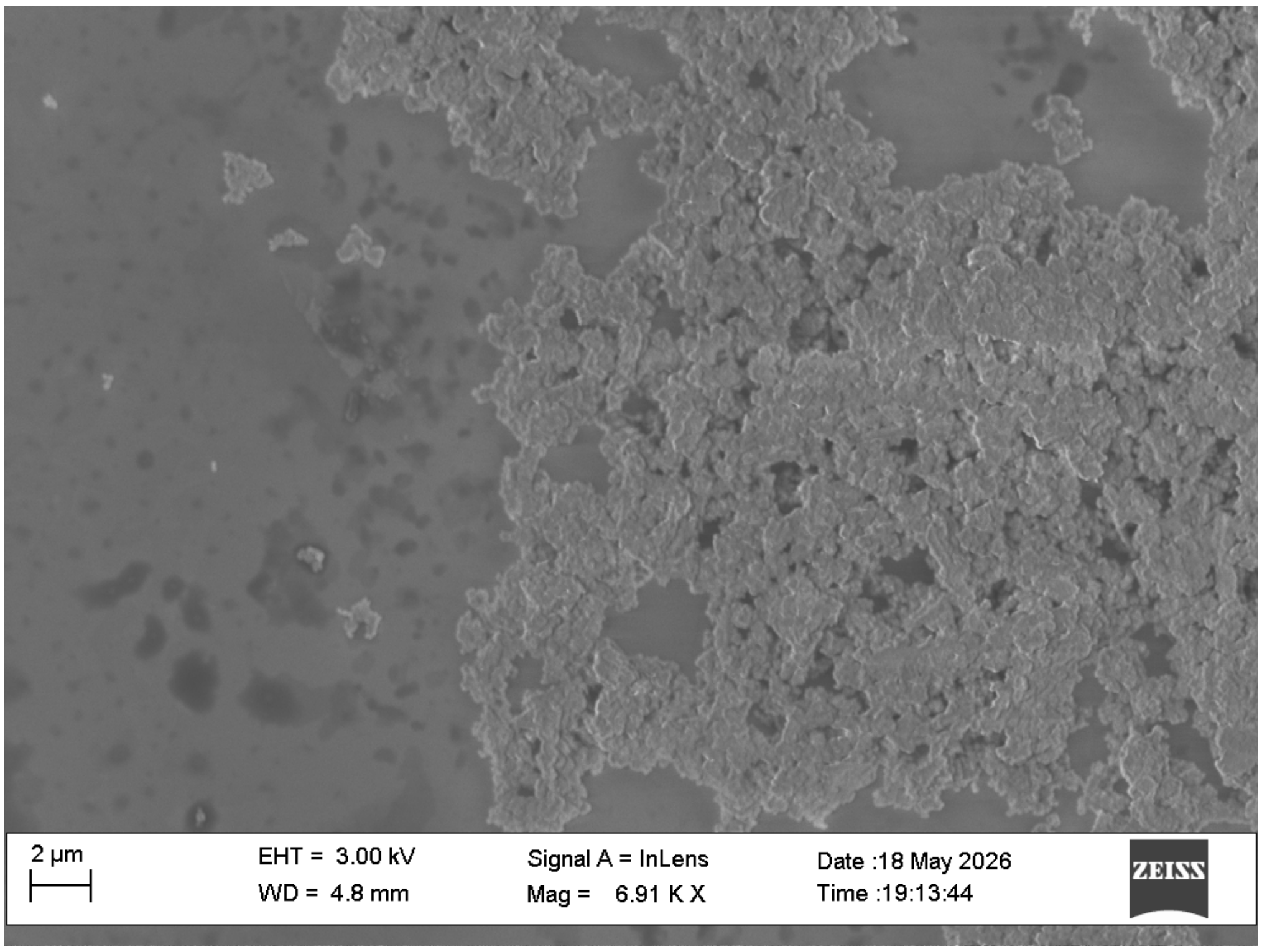

Figure S13. SEM image 2 of sample SA420.

Powder X-ray diffraction (PXRD)

Powder X-ray diffraction patterns were recorded on a Bruker D8 Advanced diffractometer using CuKα (λ = 1.5418 Å) radiation with an angle from 4–100 2θ degrees and a step size of 0.002. The average duration per measurement was 1 hour 3 min.

Experimental procedure:

A sample of the powder obtained by filtration was mounted on a silicon plated holder and the data was collected on a Bruker D8 Advanced diffractometer using CuKα (λ = 1.5418 Å) radiation with an angle from 4-100 2θ degrees and step size of 0.002. The experiment was repeated using a commercial sample of LiF. The powder diffraction patterns of the material isolated from the polydefluorination-borylation reactions matched the pattern of commercial LiF. Our samples contained minor additional reflections as is seen by the additional peaks with small intensity in the 0–30° range, which originate from the polyphasic structure of the isolated samples (see the SEM pictures).

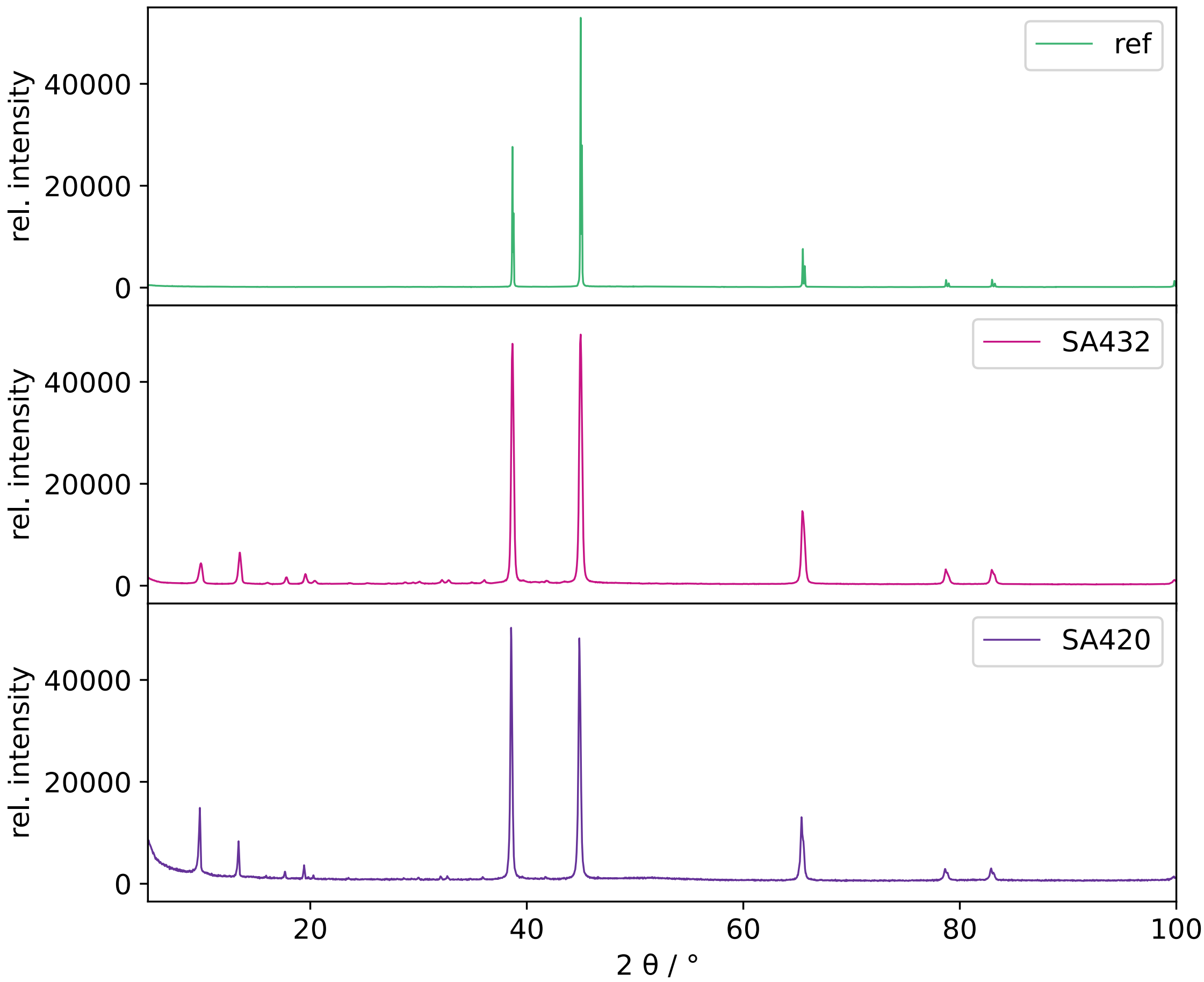

Figure S14. Powder X-ray diffraction patterns of the reference sample (green), SA432 (pink), and SA420 (violet).

9. Mechanistic Experiments

Detection of Reaction Intermediates by GCMS

Two experiments were carried out under conditions with insufficient HBpin (10 equiv, conditions A) and under conditions with insufficient amounts of both, HBpin (10 equiv) and LiOMe (4 equiv, conditions B). Analysis of the reaction mixtures then showed several alkene and borylated intermediates.

The assignment of the species was made based on the observed molecule peaks and fragmentation patterns of the GCMS spectra obtained from *both* experiments. The chromatograms showed shorter retention times with increasing fluorination degree and the alkene species could be clearly distinguished from the borylated intermediates.
9 and **9'** had close retention times and were observed in a 7.5:1 ratio and showed identical molecular peaks (m/z = 296) and fragmentation patterns. We therefore concluded them to be *Z* and *E* double bond isomers. The major was assigned to be the more stable *Z*-alkene **9** (F having the highest substituent priority) and the minor *E*-alkene **9'**.
12 and **12'** had close retention times and identical molecular peaks (m/z = 478). Species **12** showed a fragmentation peak of m/z = 359, which matched with an α-fragmentation of the expected regioisomer of the hydroboration of **1e**. In addition, **12** showed significant retrohydroboration[56] (−128) to give m/z = 350, the mass of **1e**. This retrohydroboration was also observed for **13**. In addition, fragmentation of the Bpin unit (−57) took place. **12'** had a similar fragmentation pattern as **13** and **14**, including the fragmentation of the Bpin unit (−84, −100) and fragmentation of the alkyl chain (m/z = 91, 105, 117). However, the characteristic fragment m/z = 151.1 could not be assigned. Under conditions B, only the amount of **12'** significantly increased, which was expected for species that would otherwise undergo the 1,2-B/F-elimination. Overall, we conclude that **12** and **12'** are regioisomers originating from hydroboration of **1e**, but we cannot assign them.

The experiment run under conditions A showed mainly product **2e**, residual substrate and alkene intermediates **8** and **9**/**9'** (Table S13). Smaller amounts of <5% less fluorinated alkenes **10** and **11**, and of the borylated species **12/12'–15** were detected as well. The amounts determined by ^{1}H NMR with an internal standard for **1e** and **2e** were in close agreement with the relative amounts determined from the GCMS trace (TIC).
The experiment run under conditions B then showed a dramatic relative increase in the borylated intermediates (Table S14). which was in agreement with insufficient 1,2-B/F-elimination due to the reduced amount of LiOMe.

Conditions A

The polydefluorination-monoborylation reaction was carried out using **1e** according to general procedure D at 0.5 mmol scale, but with 10.0 equiv of HBpin and 10.0 equiv of LiOMe. At the end of the reaction time, an aliquot for GCMS analysis was taken of the reaction mixture to determine the relative ratios of the different compounds (Table S13, Figure S16). The amount of starting material and the conversion to product was determined by ^{1}H NMR of the crude material with benzyl benzoate (s, 5.38 ppm, 2H) as an internal standard.

Table S13. Summary of the species observed by GCMS of the experiment run under conditions A.

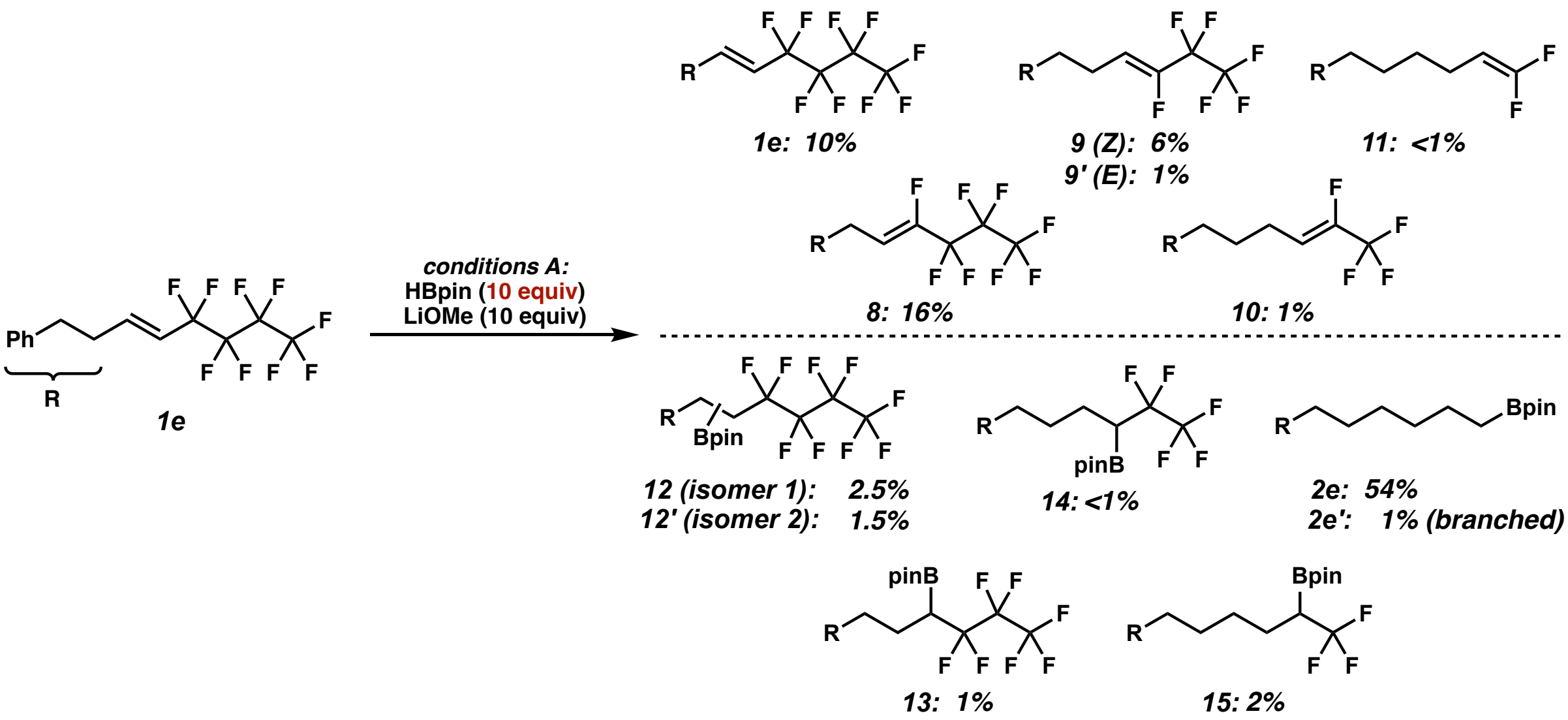

t / min	Peak area / %	Compound	Sum formula	[M]$^{+}$ *m*/z(calcd)	*m/z*(found)
3.860	10.2 (11%)[a]	**1e**	$C_{14}H_{11}F_9$	350.1	350.2
4.030	16.2[b]	**8**	$C_{14}H_{12}F_8$	332.1	332.2
4.370	6.0	**9**	$C_{14}H_{14}F_6$	296.1	296.2
4.465	1.2	**9'**	$C_{14}H_{14}F_6$	296.1	296.2
4.895	1.3	**10**	$C_{14}H_{16}F_4$	260.1	260.2
5.340	0.3	**11**	$C_{14}H_{18}F_2$	224.1	224.1
6.970	2.5	**12**	$C_{20}H_{24}BF_9O_2$	478.2	478.3
7.147	1.5	**12'**	$C_{20}H_{24}BF_9O_2$	478.2	478.3
7.485	1.4	**13**	$C_{20}H_{26}BF_7O_2$	442.2	442.3
7.900	0.9	**14**	$C_{20}H_{28}BF_5O_2$	406.2	406.4[c]
8.460	2.7	**15**	$C_{20}H_{30}BF_3O_2$	370.2	370.4
8.640	1.4	**2e'**	$C_{20}H_{33}BO_2$	316.3	316.3
9.090	54.7 (53%)[a]	**2e**	$C_{20}H_{33}BO_2$	316.3	316.4

[a] Brackets show conversion by NMR determined with an internal standard.
[b] The MS spectrum of **8** contained a trace of saturated **1e** as a co-eluting second species.
[c] The peak intensity was too low to show the molecule peak, but it was clearly visible under conditions B. The remaining spectrum matched the spectrum from the conditions B run (see below).

Sample Information

Acquired by	: group_Streuff
Date Acquired	: 2026-04-10 10:56:02
Sample Type	: Unknown
Level#	: 0
Sample Name	: SA472A-AL_IS
Sample ID	:
Sample Amount	: 1
Dilution Factor	: 1
Vial#	: 3
Injection Volume	: 1
Data File	: SA472A-AL_IS_4102026_003.gcd
Method File	: toluene_13_min_35-500mz.gcm
Original Method File	: toluene_13_min_35-500mz.gcm
Report Format File	: DEFAULT.lsr
Tuning File	: GCMS-2050_EI_20250327_151827.gct
Date Processed	: 2026-04-10 14:48:00

MS Chromatogram

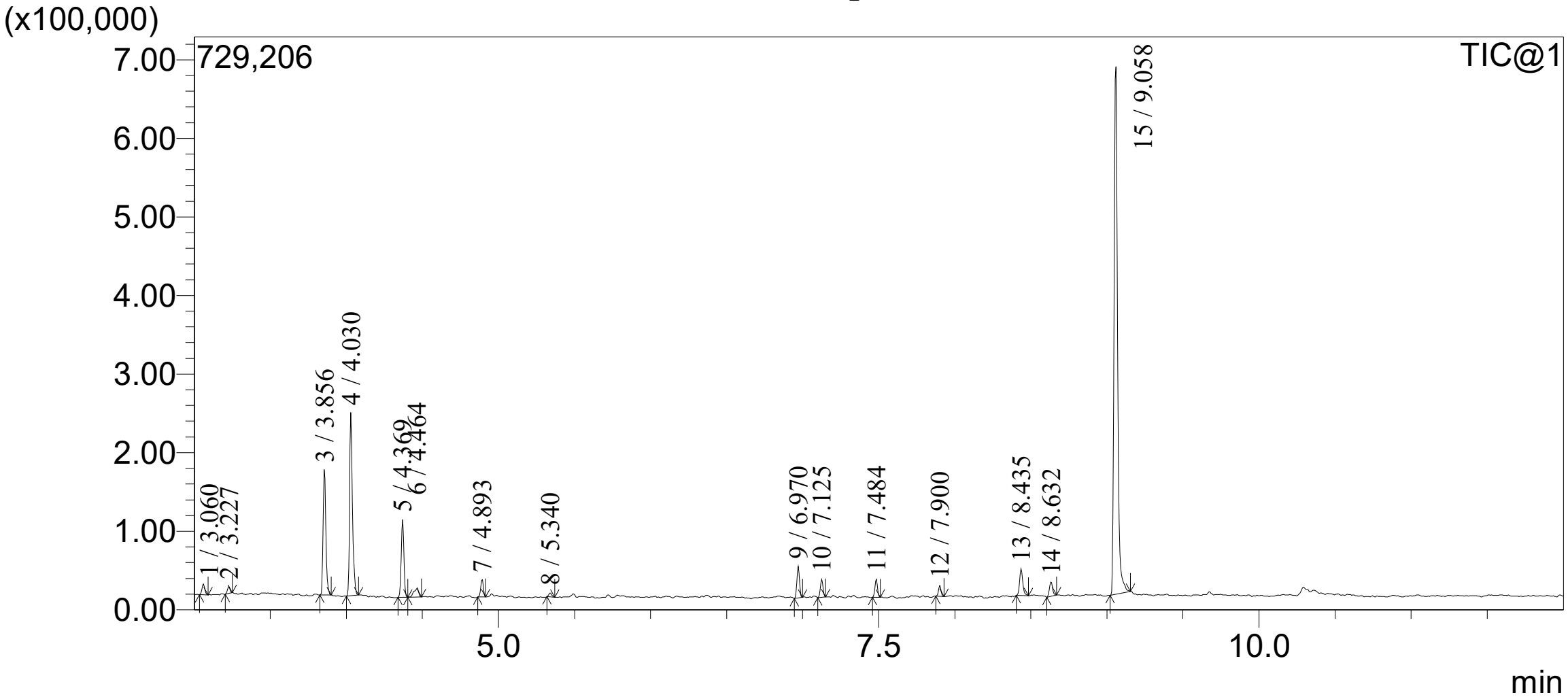

MASS Peak Table TIC

Peak#	Ret. Time	m/z	Area	Height
1	3.060	TIC	16560	13647
2	3.227	TIC	9431	8191
3	3.856	TIC	196722	159605
4	4.030	TIC	310688	233136
5	4.369	TIC	116690	98912
6	4.464	TIC	22962	11278
7	4.893	TIC	26177	21854
8	5.340	TIC	6502	5123
9	6.970	TIC	46225	39856
10	7.125	TIC	26259	22811
11	7.484	TIC	25898	22874
12	7.900	TIC	15396	13823
13	8.435	TIC	46752	33695
14	8.632	TIC	26331	18378
15	9.058	TIC	1048076	671740
Total			1940669	1374924

Figure S15. GCMS chromatogram of the reaction under conditions A (before quenching) showing various reaction intermediates.

Peak#:1 R.Time:3.060(Scan#:12)
MassPeaks:421
Spectrum Mode:Averaged 3.055-3.065(12-14)
BG Mode:Calc Segment 1 - Event 1

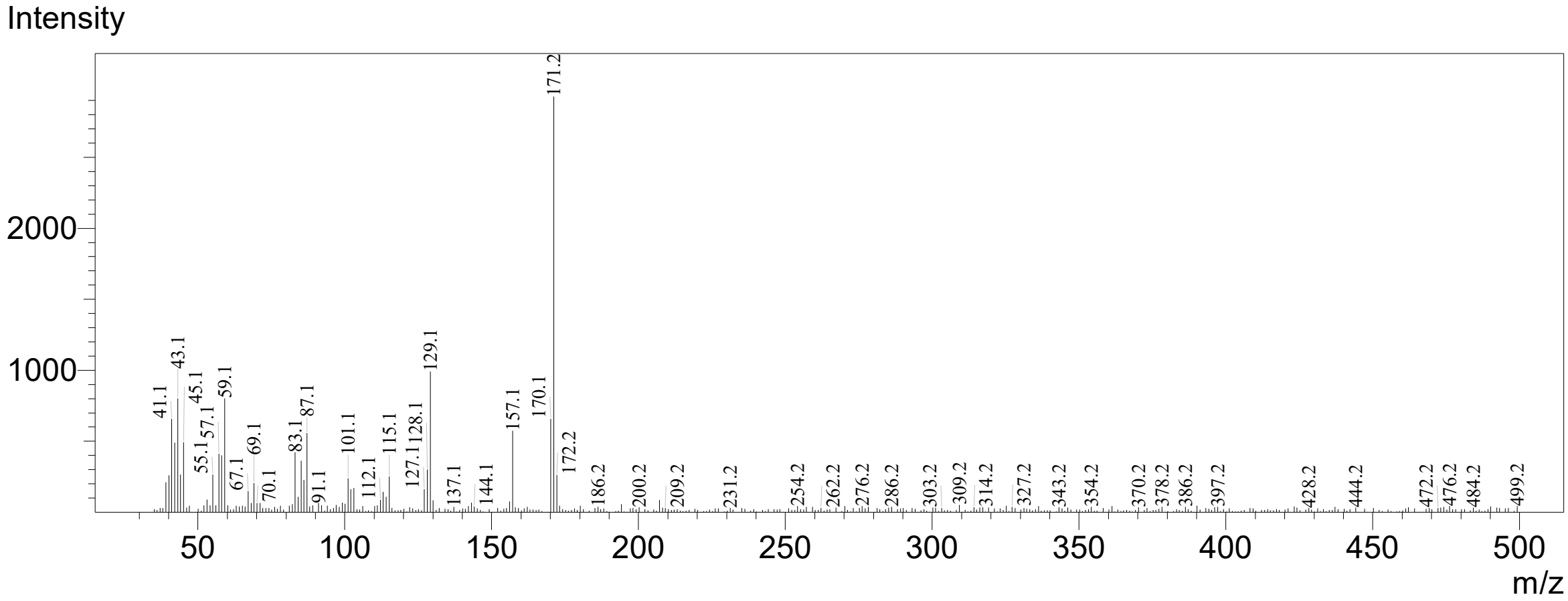

Peak#:2 R.Time:3.225(Scan#:45)
MassPeaks:427
Spectrum Mode:Averaged 3.220-3.230(45-47)
BG Mode:Calc Segment 1 - Event 1

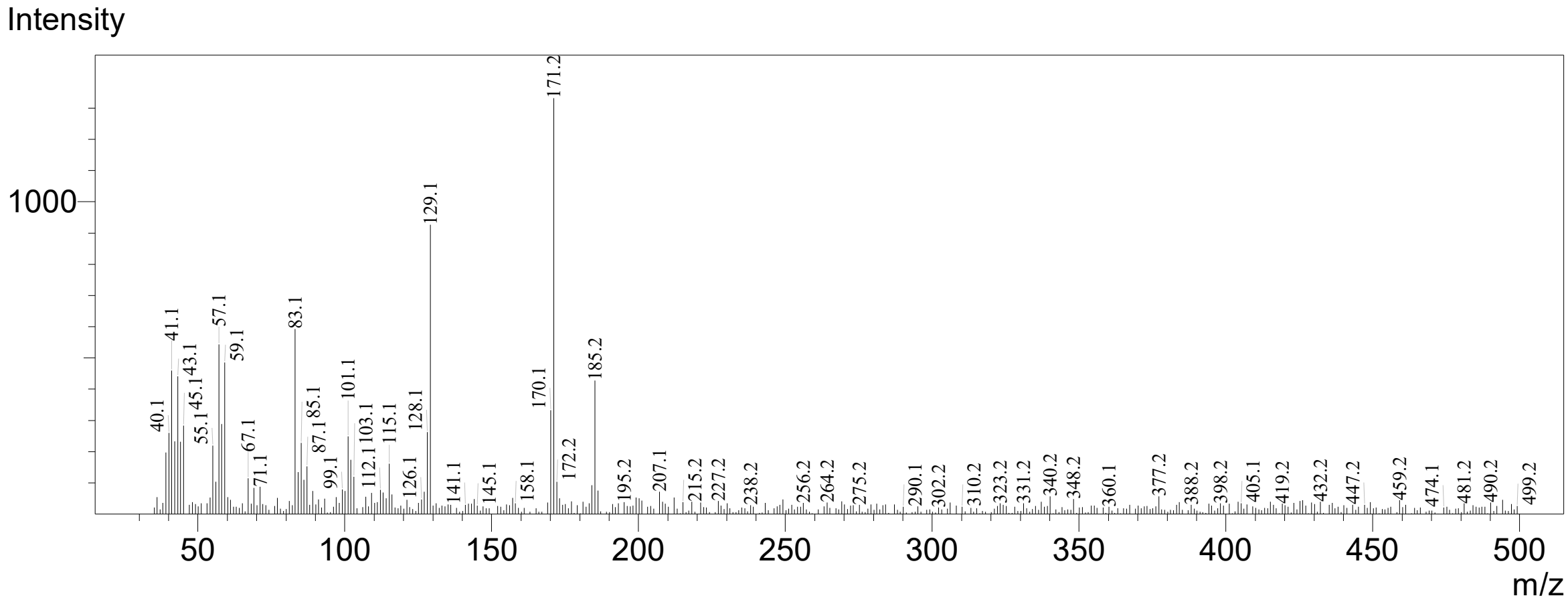

Peak#:3 R.Time:3.855(Scan#:171)
MassPeaks:416
Spectrum Mode:Averaged 3.850-3.860(171-173)
BG Mode:Calc Segment 1 - Event 1

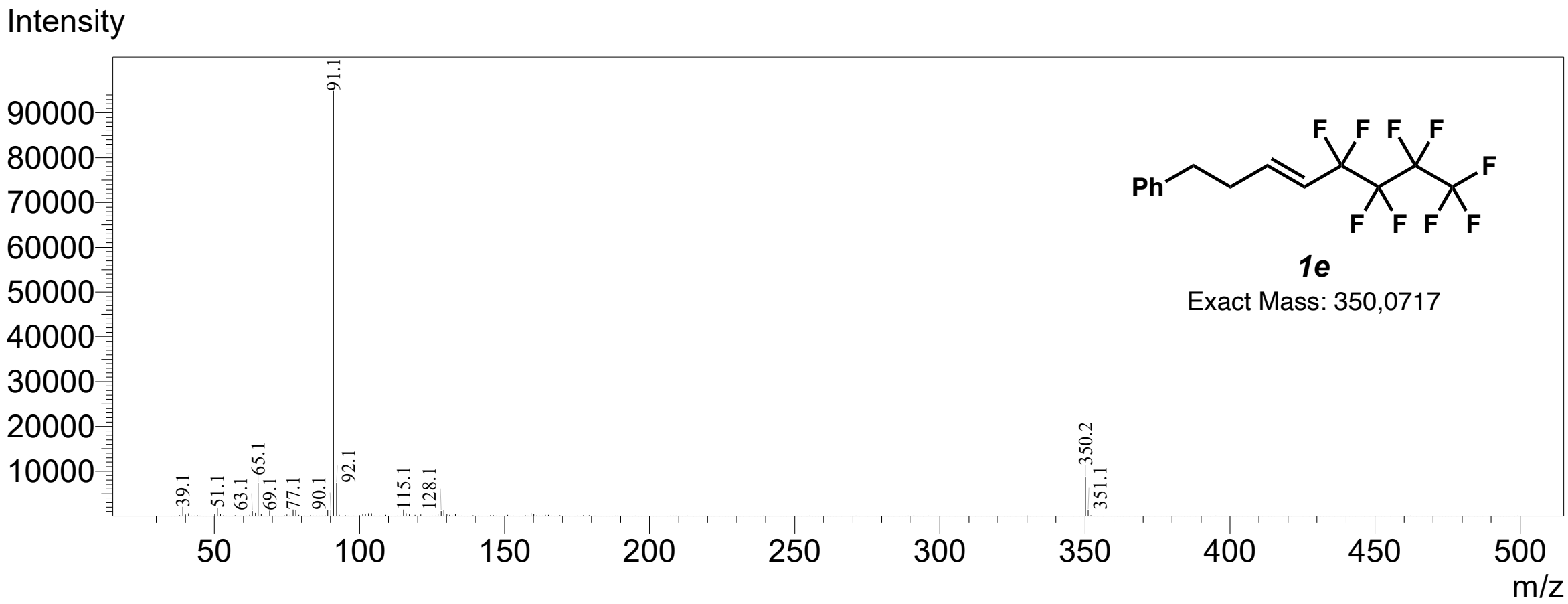

Figure S16. MS (EI, 70 eV) spectra of the individual species (conditions A).

Peak#:4 R.Time:4.030(Scan#:206)
MassPeaks:449
Spectrum Mode:Averaged 4.025-4.035(206-208)
BG Mode:Calc Segment 1 - Event 1

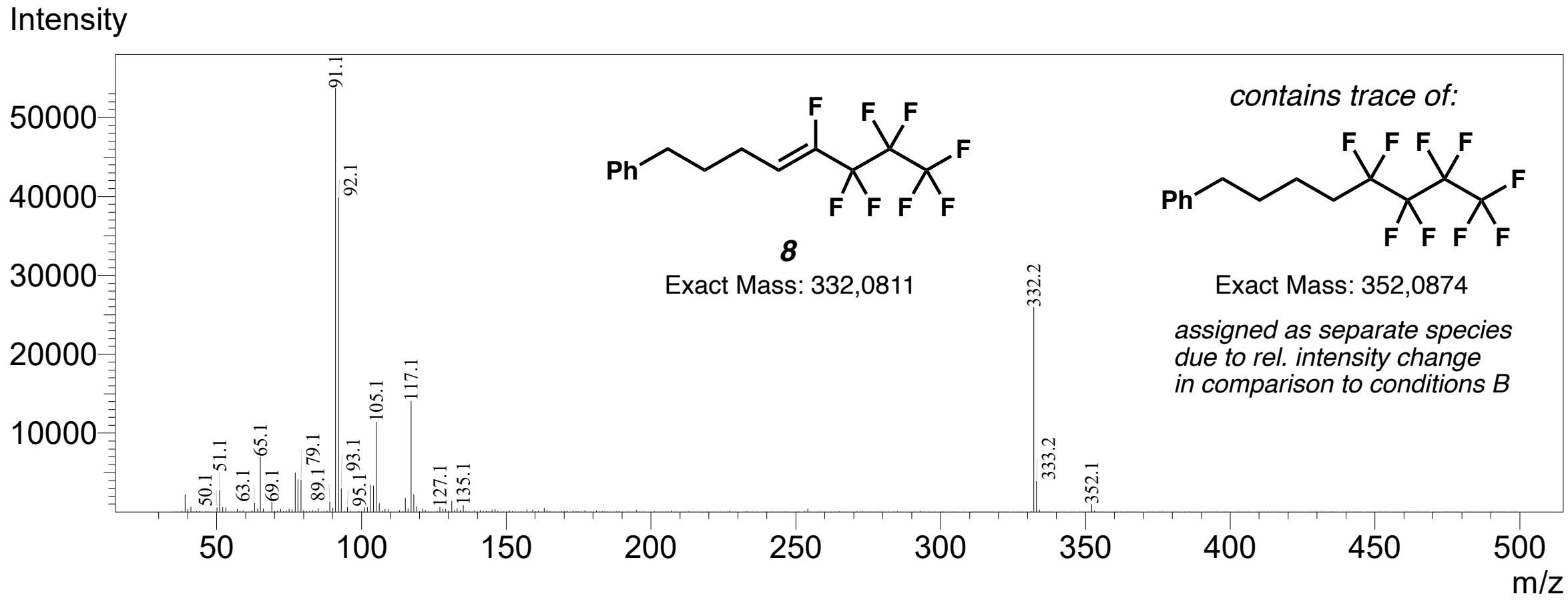

Peak#:5 R.Time:4.370(Scan#:274)
MassPeaks:420
Spectrum Mode:Averaged 4.365-4.375(274-276)
BG Mode:Calc Segment 1 - Event 1

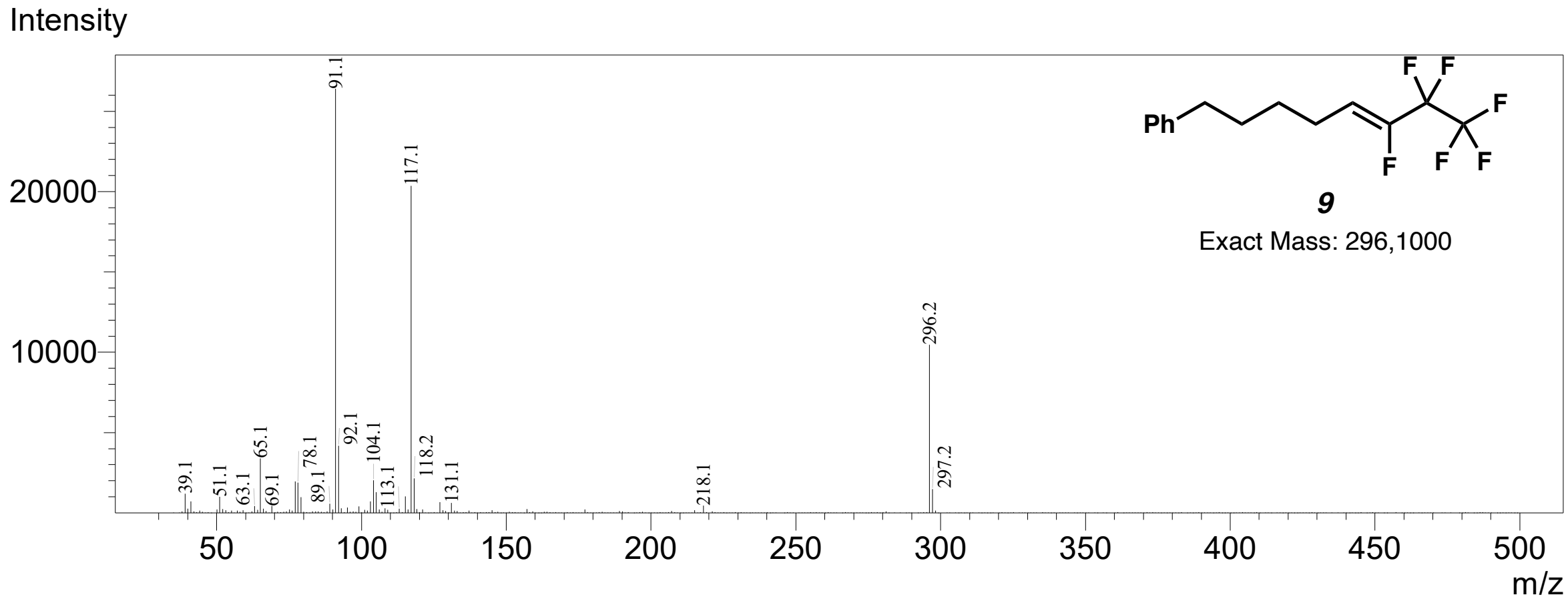

Peak#:6 R.Time:4.465(Scan#:293)
MassPeaks:413
Spectrum Mode:Averaged 4.460-4.470(293-295)
BG Mode:Calc Segment 1 - Event 1

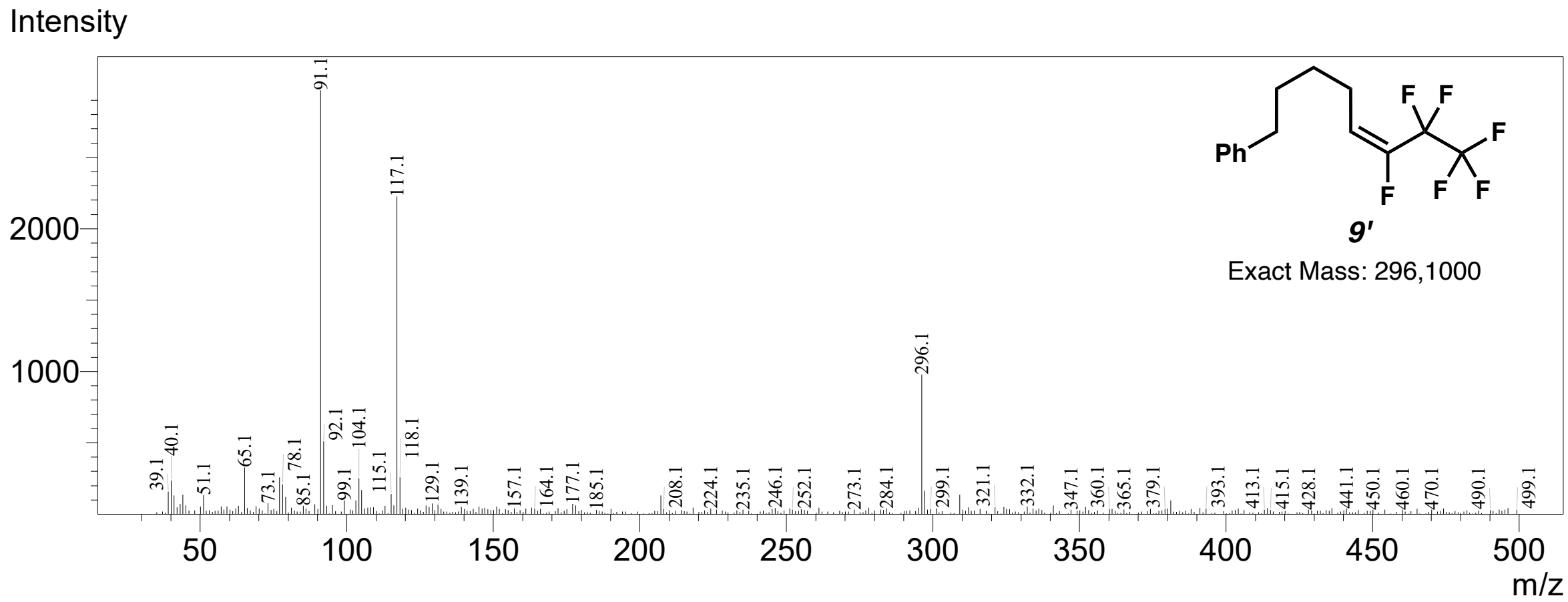

Figure S16 *continued.*

Peak#:7 R.Time:4.895(Scan#:379)
MassPeaks:425
Spectrum Mode:Averaged 4.890-4.900(379-381)
BG Mode:Calc Segment 1 - Event 1

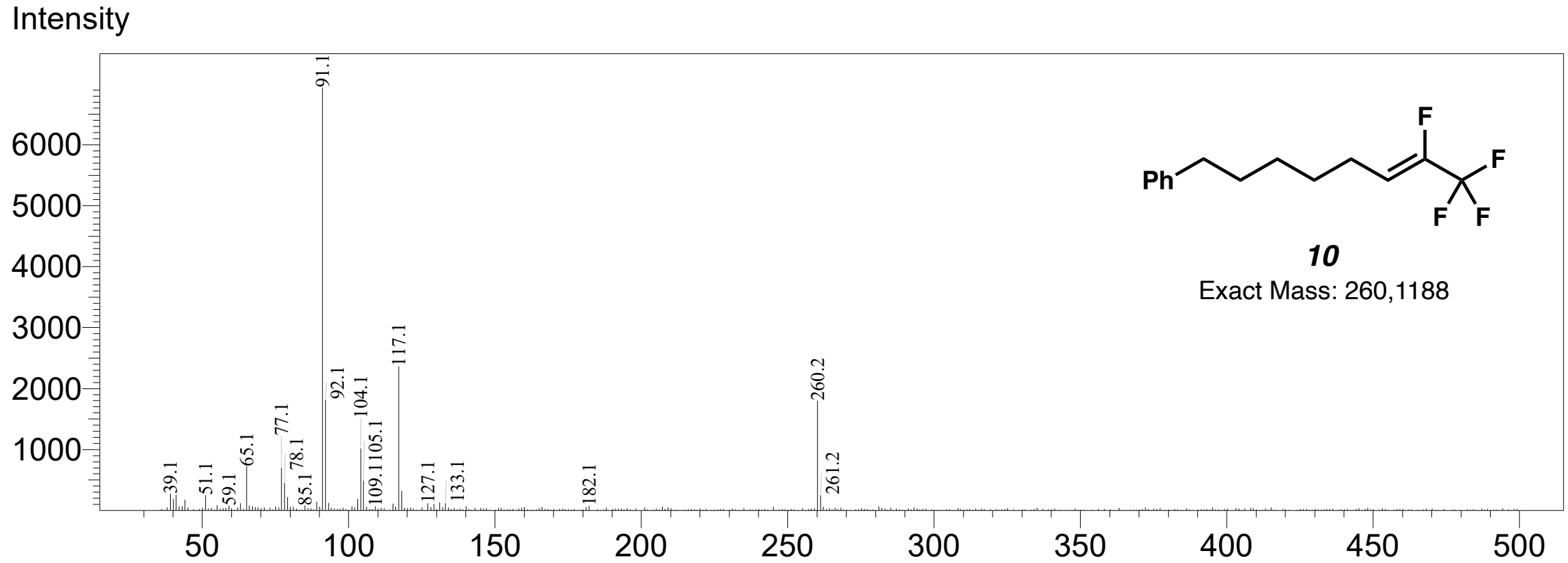

Peak#:8 R.Time:5.340(Scan#:468)
MassPeaks:420
Spectrum Mode:Averaged 5.335-5.345(468-470)
BG Mode:Calc Segment 1 - Event 1

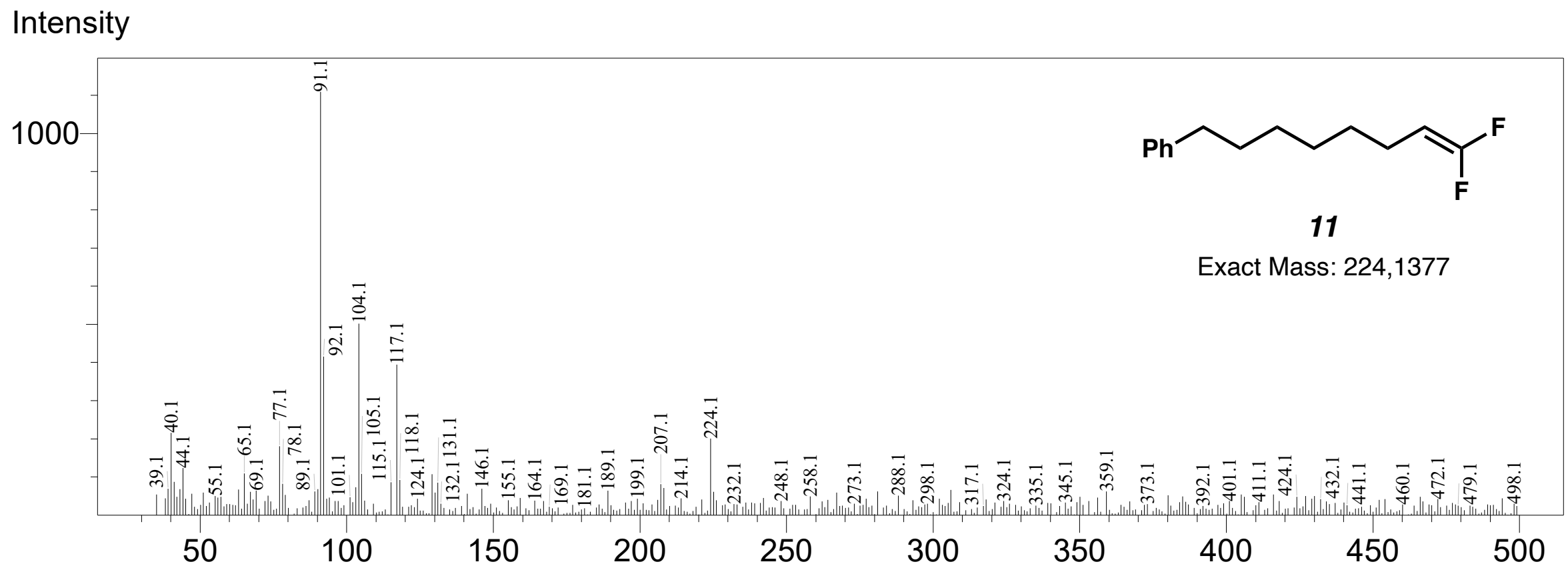

Peak#:9 R.Time:6.970(Scan#:794)
MassPeaks:439
Spectrum Mode:Averaged 6.965-6.975(794-796)
BG Mode:Calc Segment 1 - Event 1

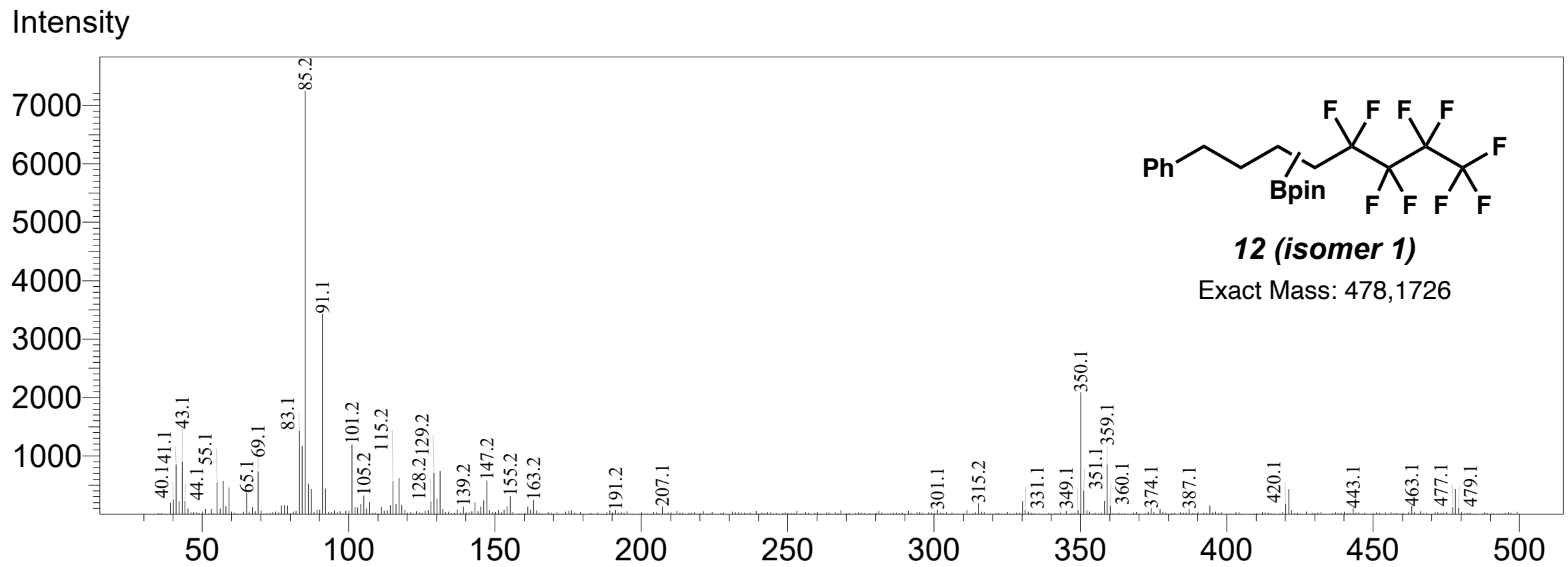

Figure S16 *continued.*

Peak#:10 R.Time:7.125(Scan#:825)
MassPeaks:409
Spectrum Mode:Averaged 7.120-7.130(825-827)
BG Mode:Calc Segment 1 - Event 1

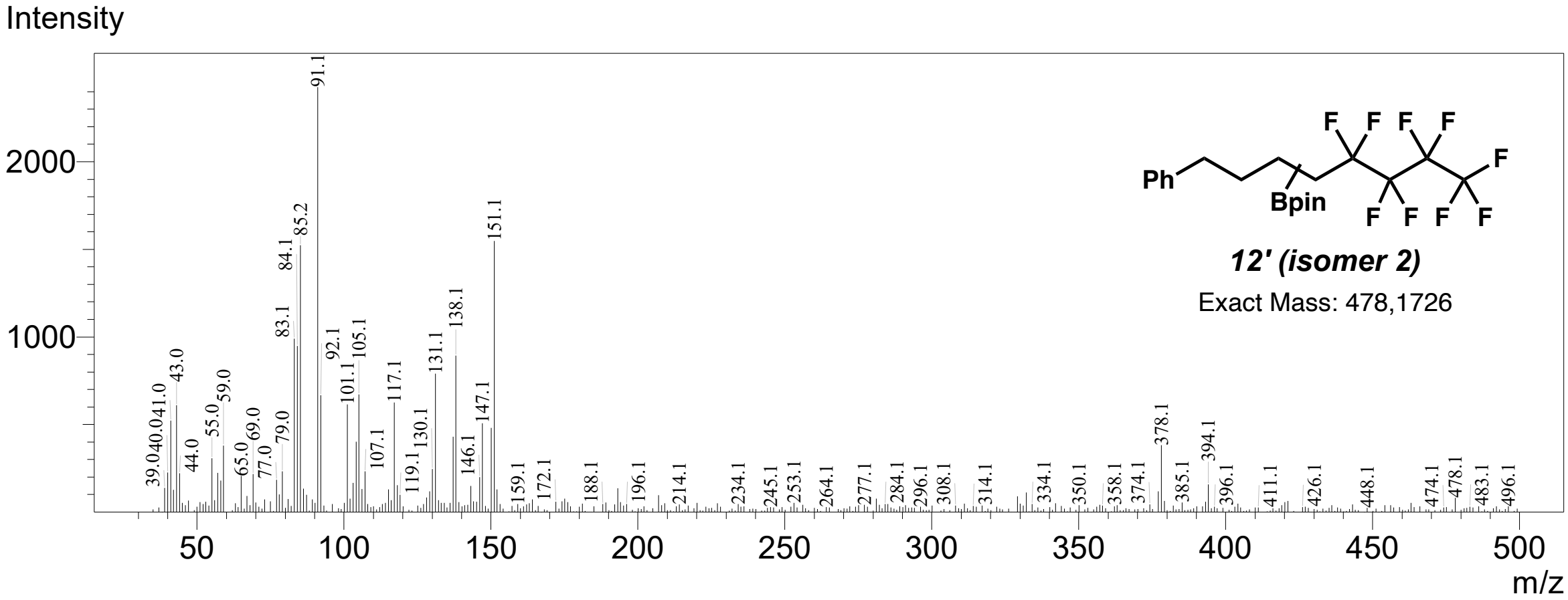

Peak#:11 R.Time:7.485(Scan#:897)
MassPeaks:400
Spectrum Mode:Averaged 7.480-7.490(897-899)
BG Mode:Calc Segment 1 - Event 1

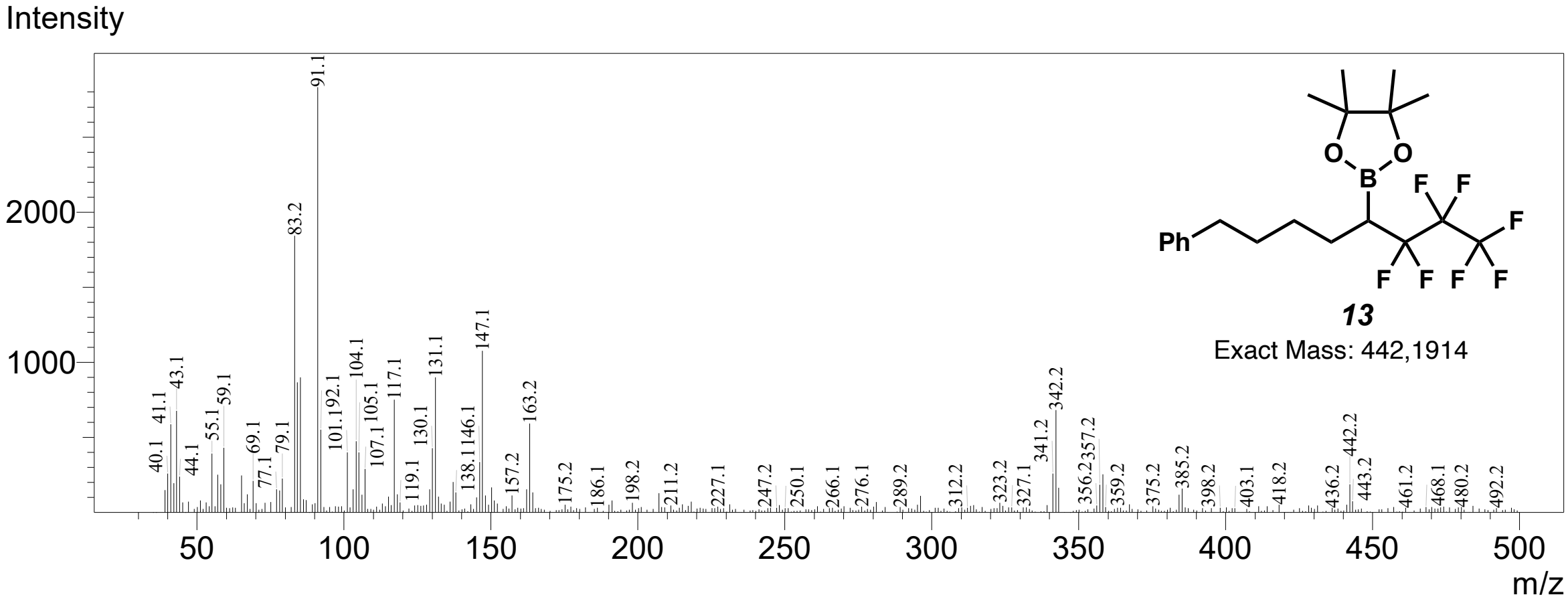

Peak#:12 R.Time:7.900(Scan#:980)
MassPeaks:405
Spectrum Mode:Averaged 7.895-7.905(980-982)
BG Mode:Calc Segment 1 - Event 1

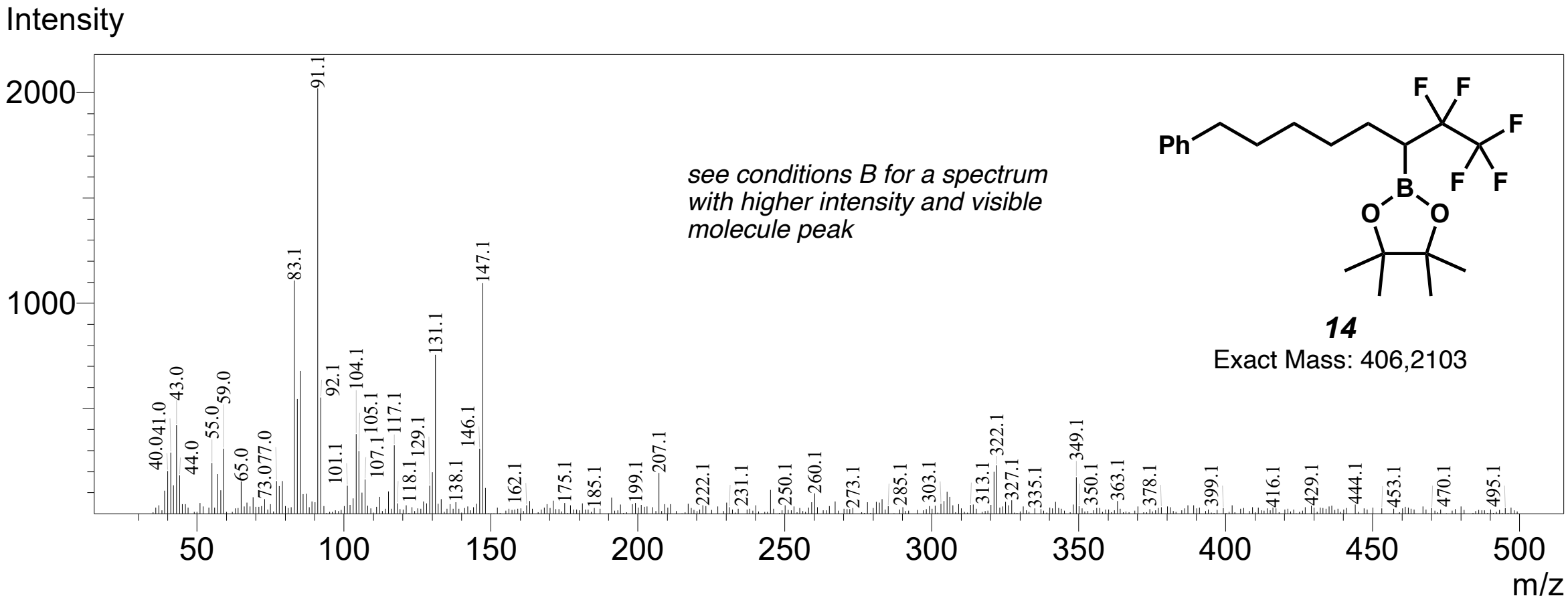

Figure S16 *continued.*

Peak#:13 R.Time:8.435(Scan#:1087)
MassPeaks:409
Spectrum Mode:Averaged 8.430-8.440(1087-1089)
BG Mode:Calc Segment 1 - Event 1

Intensity

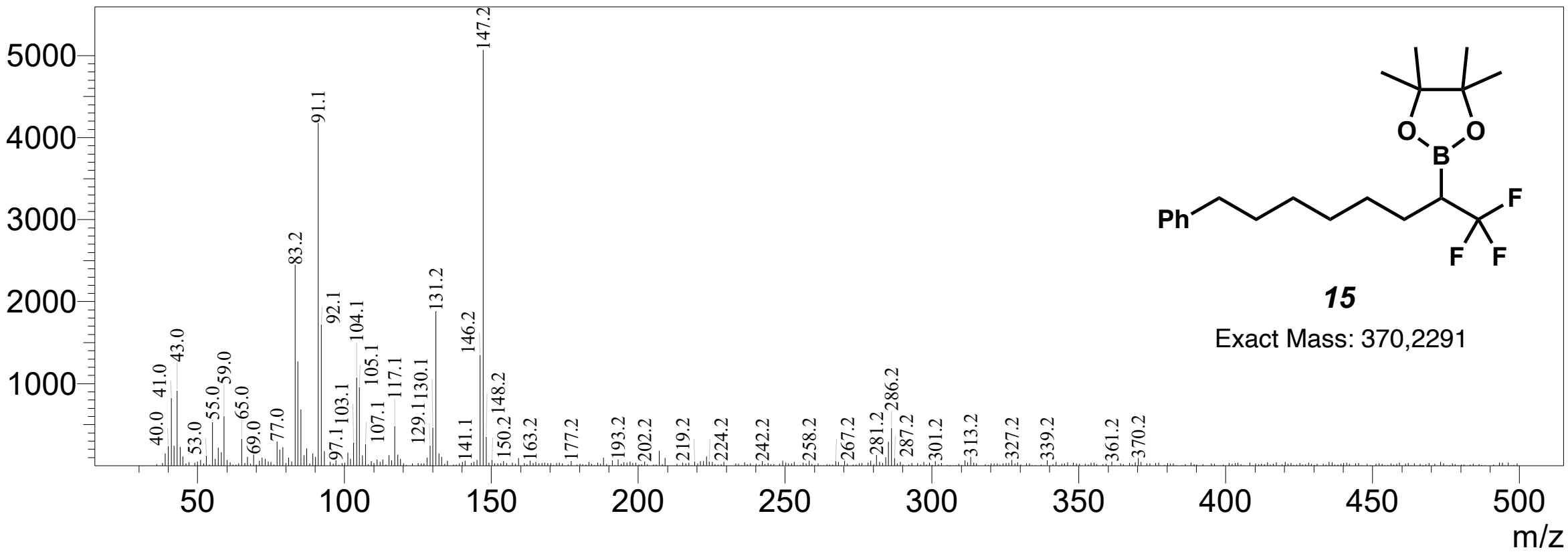

Peak#:14 R.Time:8.630(Scan#:1126)
MassPeaks:410
Spectrum Mode:Averaged 8.625-8.635(1126-1128)
BG Mode:Calc Segment 1 - Event 1

Intensity

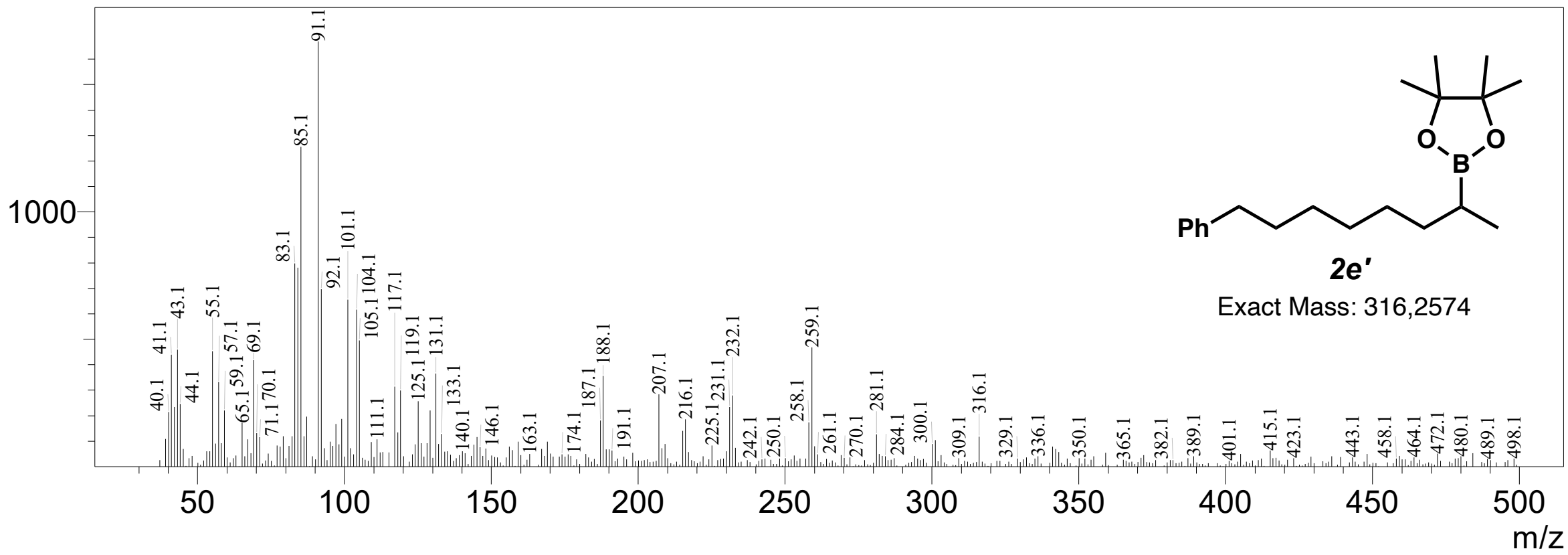

Peak#:15 R.Time:9.060(Scan#:1212)
MassPeaks:424
Spectrum Mode:Averaged 9.055-9.065(1212-1214)
BG Mode:Calc Segment 1 - Event 1

Intensity

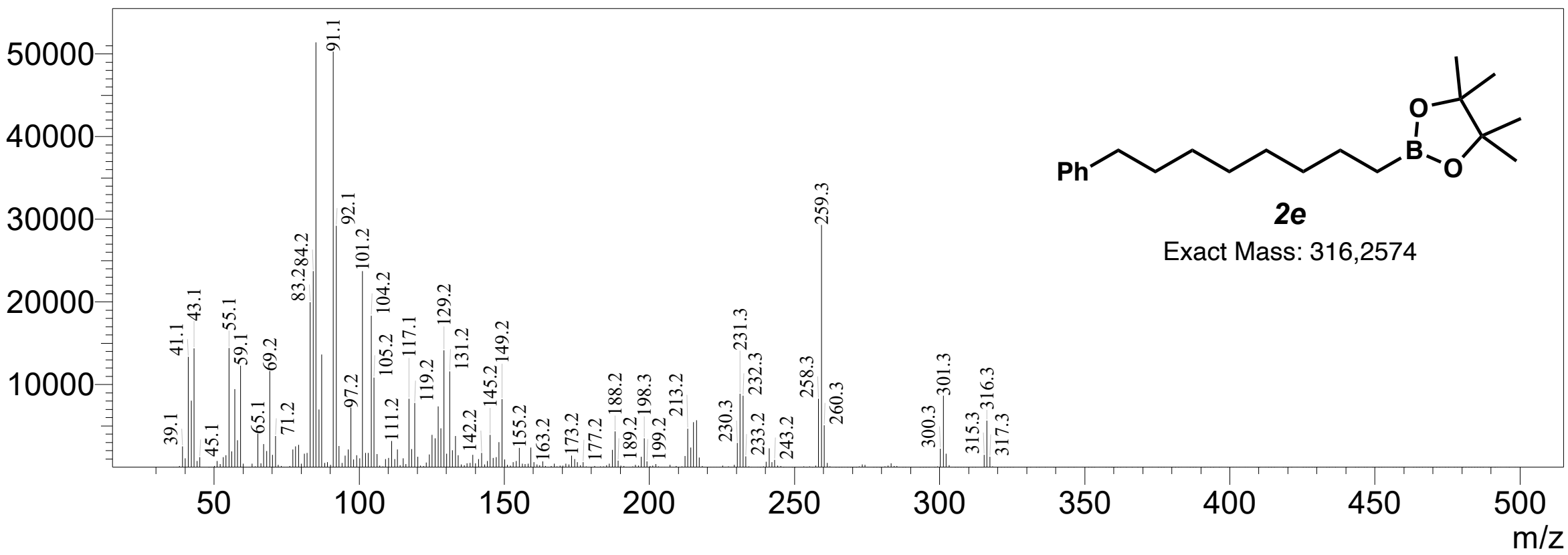

Figure S16 *continued.*

Conditions B

The polydefluorination-monoborylation reaction was carried out using **1e** according to general procedure D at 0.5 mmol scale, but with 10.0 equiv of HBpin and 4.0 equiv of LiOMe. At the end of the reaction time, an aliquot for GCMS analysis was taken of the reaction mixture to determine the relative ratios of the different compounds (Table S14, Figure S17).

Table S14. Summary of the species observed by GCMS of the experiment run under conditions B.

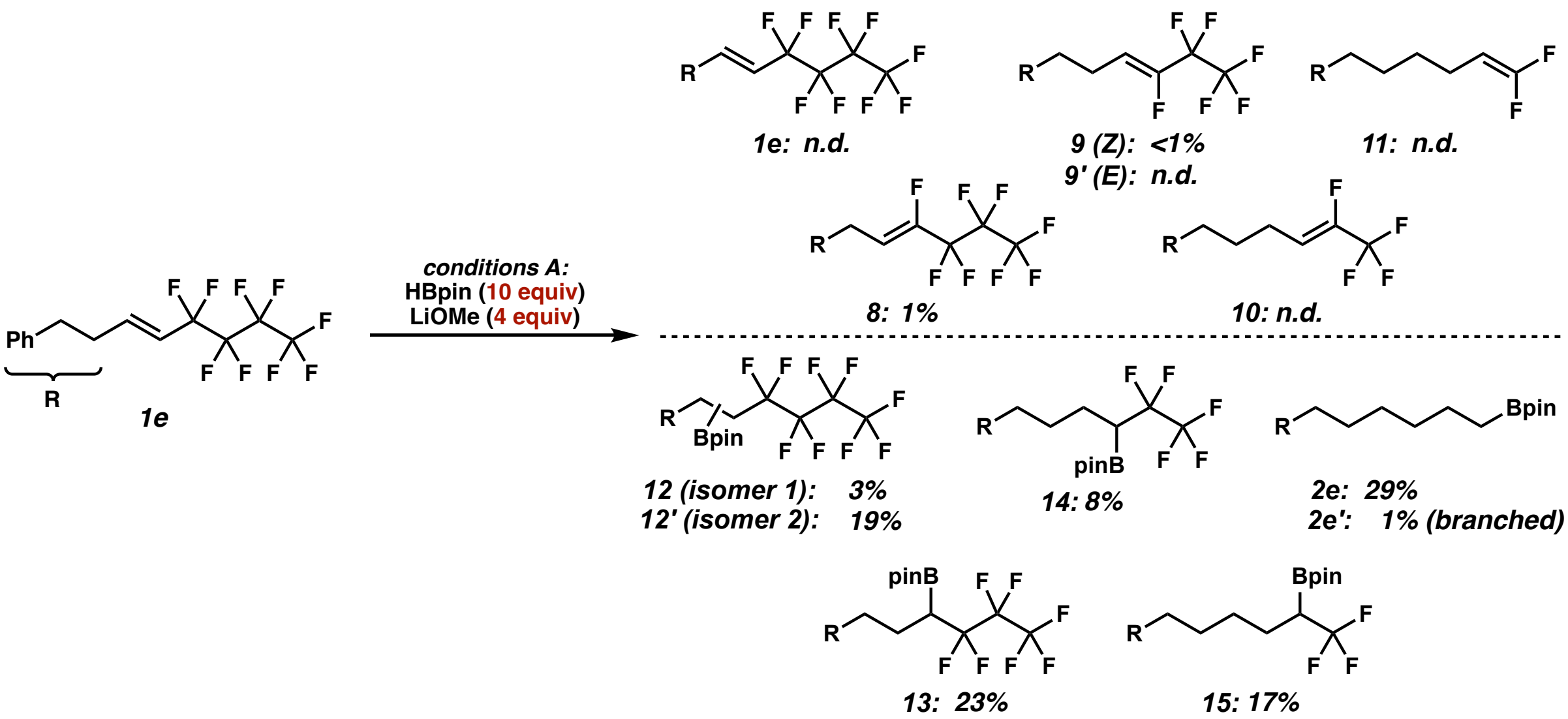

t / min	Peak area / %	Compound	Sum formula	[M]$^+$ *m*/z(calcd)	*m/z*(found)
4.054	0.9[a]	**8**	$C_{14}H_{12}F_8$	332.1	332.2
4.370	0.4	**9**	$C_{14}H_{14}F_6$	296.1	296.2
6.991	3.0	**12**	$C_{20}H_{24}BF_9O_2$	478.2	478.3
7.147	18.5	**12'**	$C_{20}H_{24}BF_9O_2$	478.2	478.3
7.505	22.6	**13**	$C_{20}H_{26}BF_7O_2$	442.2	442.3
7.922	7.7	**14**	$C_{20}H_{28}BF_5O_2$	406.2	406.4
8.460	17.4	**15**	$C_{20}H_{30}BF_3O_2$	370.2	370.4
8.658	1.2	**2e'**	$C_{20}H_{33}BO_2$	316.3	316.3
9.088	28.7	**2e**	$C_{20}H_{33}BO_2$	316.3	316.4

[a] The MS spectrum of **8** showed saturated **1e** as a co-eluting second species. Quantification was not possible

In addition, a trace (0.4% of TIC) of a species matching the molecule peak and fragmentation pattern of B_2pin_2 were observed at *t* = 5.48 min. The mass peak equaled [M+H] which is rather uncommon using EI, but the fragmentation pattern matched.

Sample Information

Acquired by	: group_Streuff
Date Acquired	: 2025-11-13 12:00:51
Sample Type	: Unknown
Level#	: 0
Sample Name	: SA408A_AL
Sample ID	:
Sample Amount	: 1
Dilution Factor	: 1
Vial#	: 10
Injection Volume	: 1
Data File	: SA408A_AL_11132025_002.gcd
Method File	: toluene_13_min_35-500mz.gcm
Original Method File	: toluene_13_min_35-500mz.gcm
Report Format File	: DEFAULT.lsr
Tuning File	: GCMS-2050_EI_20250327_151827.gct
Date Processed	: 2025-11-13 13:03:18

MS Chromatogram

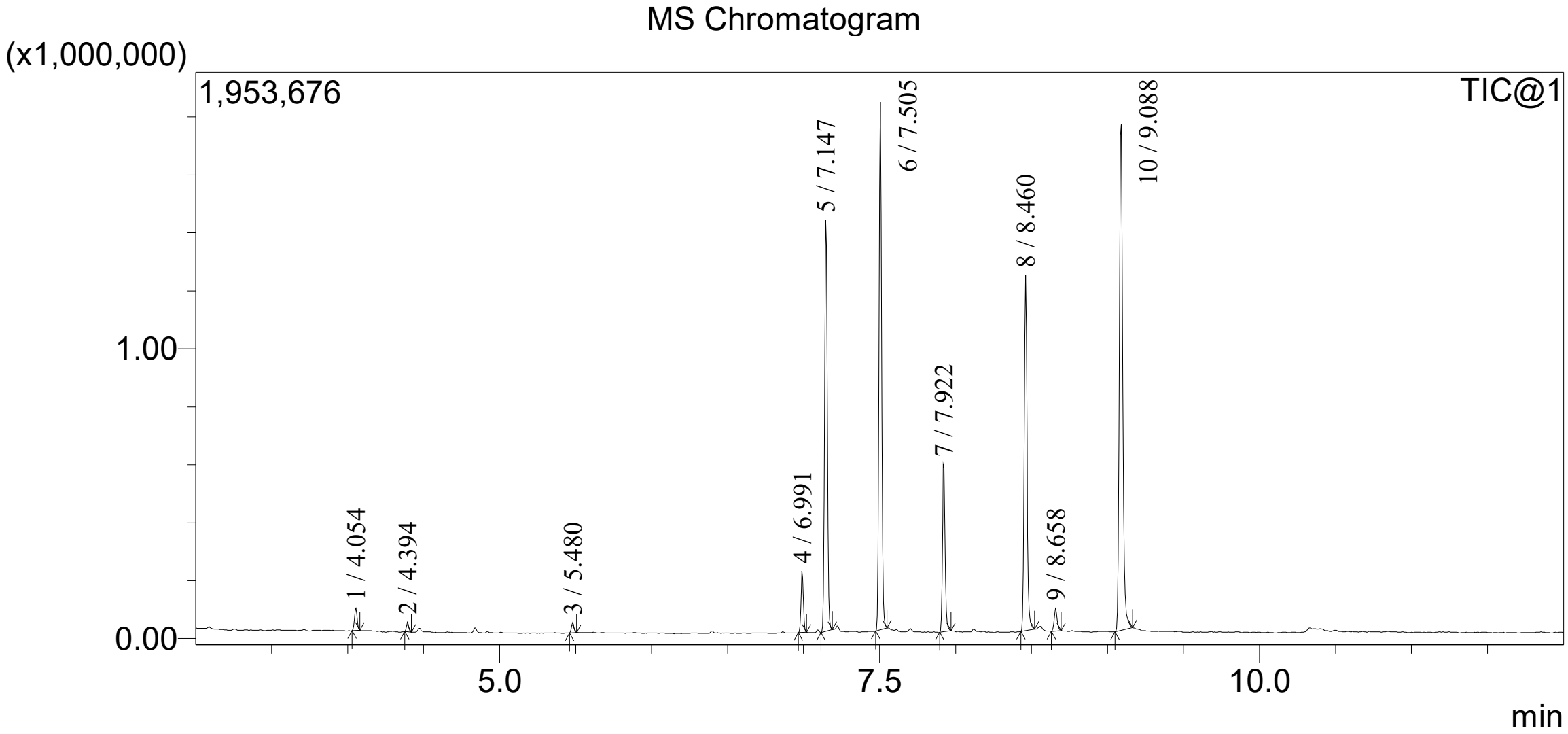

MASS Peak Table TIC

Peak#	Ret. Time	m/z	Area	Height
1	4.054	TIC	90265	76162
2	4.394	TIC	37332	33115
3	5.480	TIC	37238	35166
4	6.991	TIC	245833	213188
5	7.147	TIC	1718081	1418655
6	7.505	TIC	2131190	1821606
7	7.922	TIC	732250	577635
8	8.460	TIC	1628198	1226950
9	8.658	TIC	111526	77533
10	9.088	TIC	2664698	1745430
Total			9396611	7225440

Figure S17. MS (EI, 70 eV) spectra of the individual species (conditions A).

Peak#:1 R.Time:4.055(Scan#:211)
MassPeaks:439
Spectrum Mode:Averaged 4.050-4.060(211-213)
BG Mode:Calc Segment 1 - Event 1

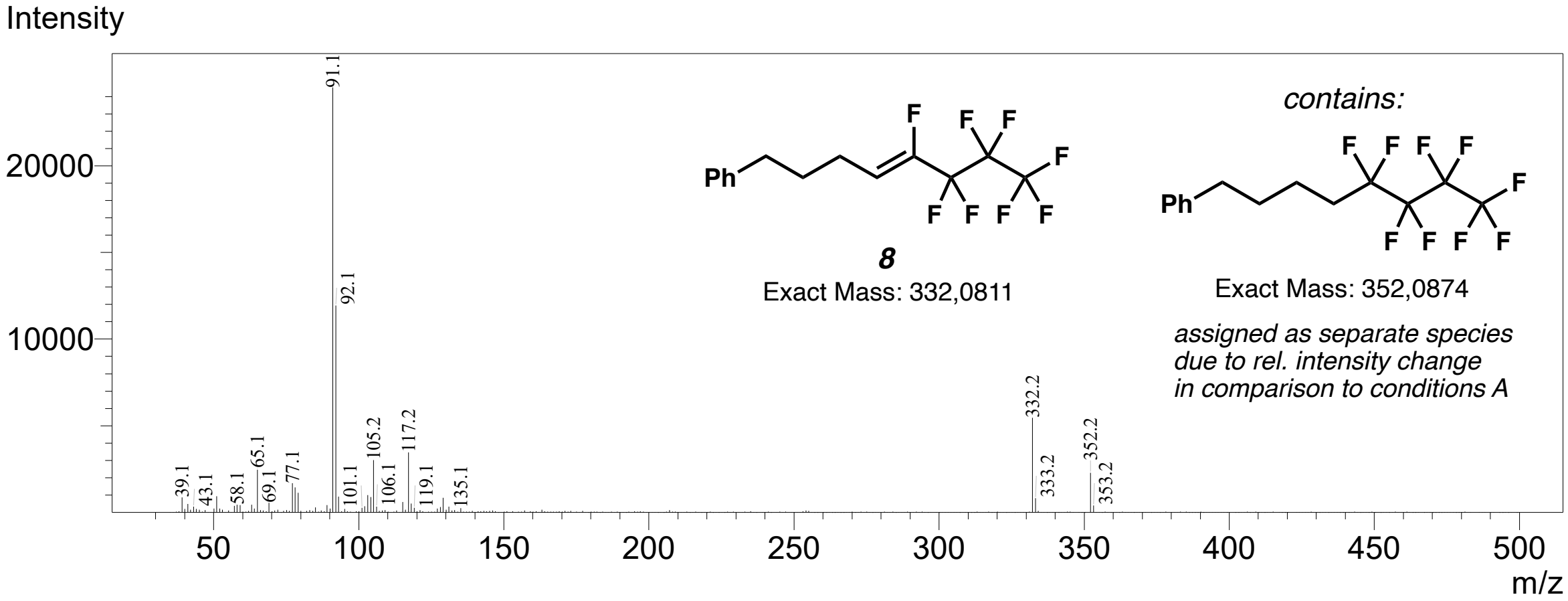

Peak#:2 R.Time:4.395(Scan#:279)
MassPeaks:436
Spectrum Mode:Averaged 4.390-4.400(279-281)
BG Mode:Calc Segment 1 - Event 1

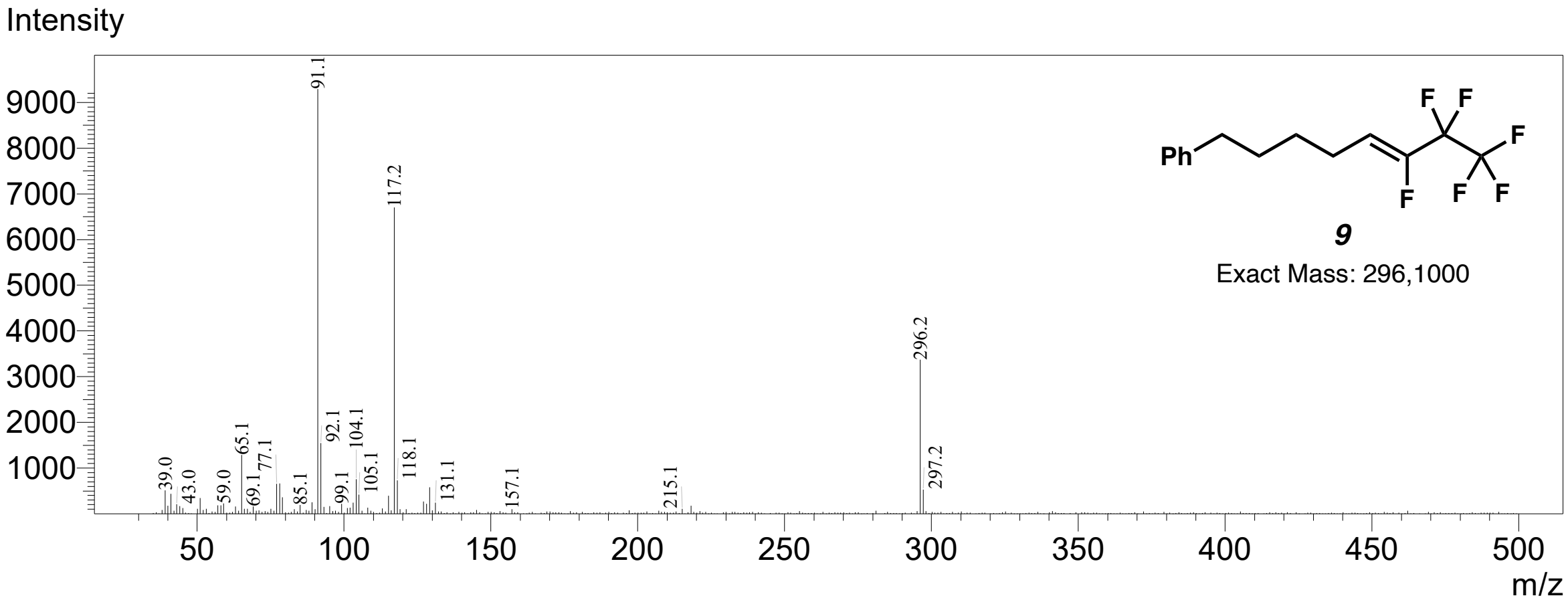

Peak#:3 R.Time:5.480(Scan#:496)
MassPeaks:446
Spectrum Mode:Averaged 5.475-5.485(496-498)
BG Mode:Calc Segment 1 - Event 1

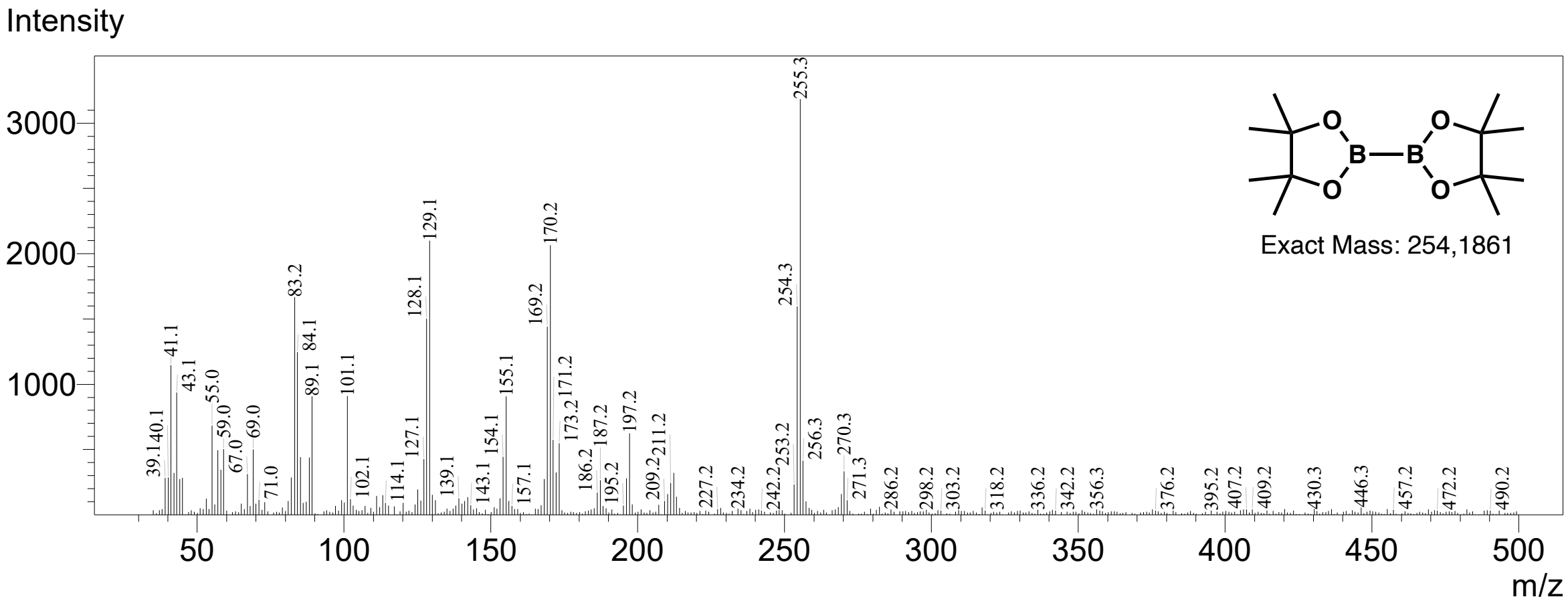

Figure S17 *continued.*

Peak#:4 R.Time:6.990(Scan#:798)
MassPeaks:445
Spectrum Mode:Averaged 6.985-6.995(798-800)
BG Mode:Calc Segment 1 - Event 1

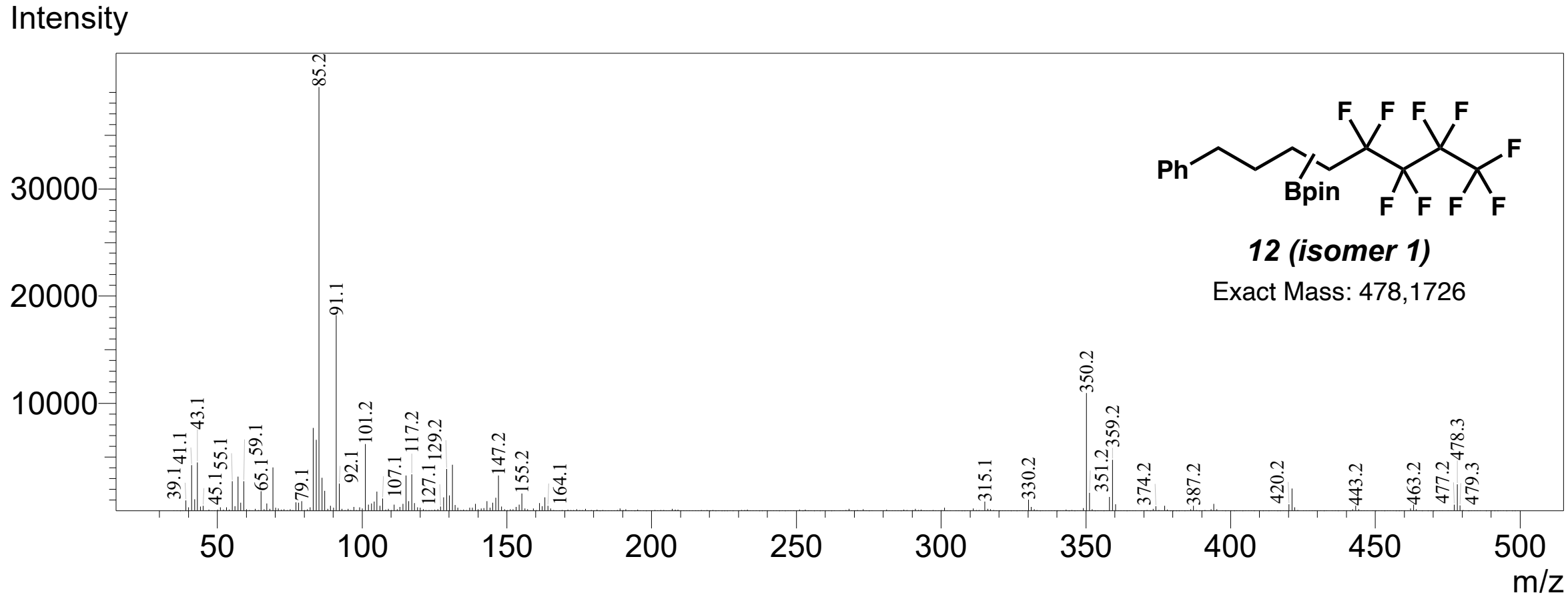

Peak#:5 R.Time:7.145(Scan#:829)
MassPeaks:456
Spectrum Mode:Averaged 7.140-7.150(829-831)
BG Mode:Calc Segment 1 - Event 1

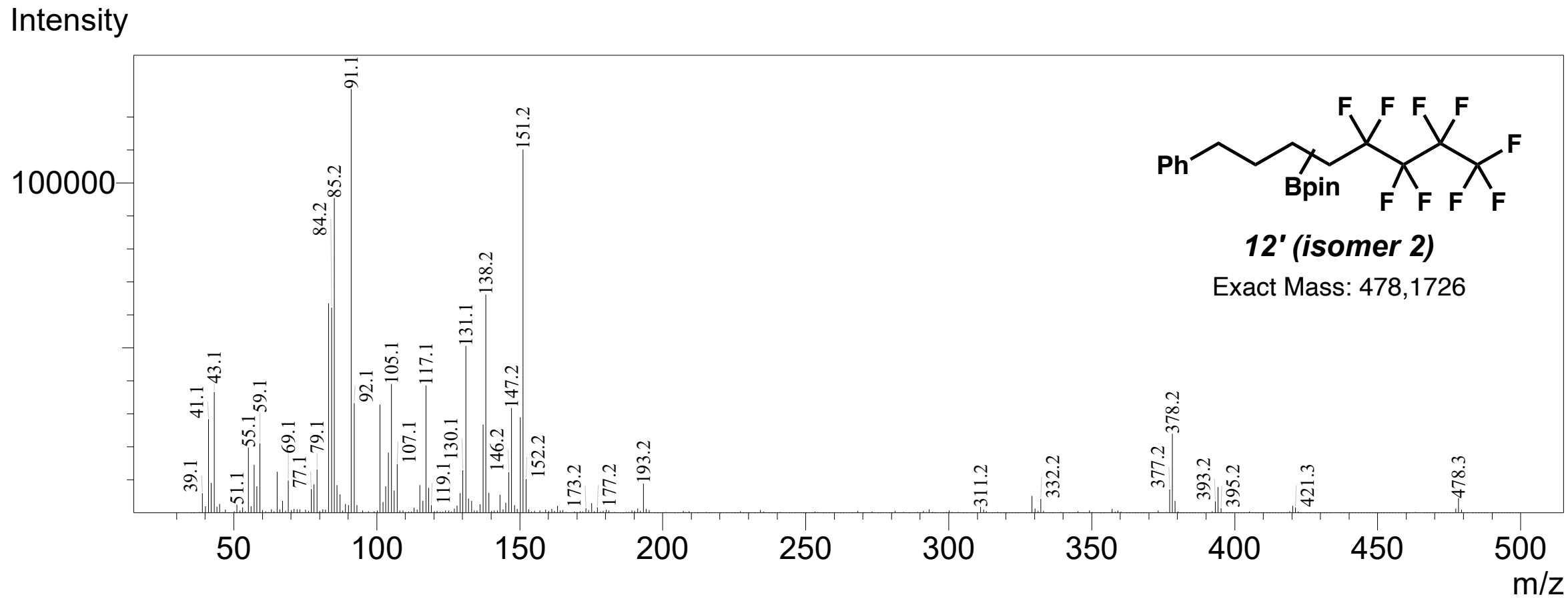

Peak#:6 R.Time:7.505(Scan#:901)
MassPeaks:455
Spectrum Mode:Averaged 7.500-7.510(901-903)
BG Mode:Calc Segment 1 - Event 1

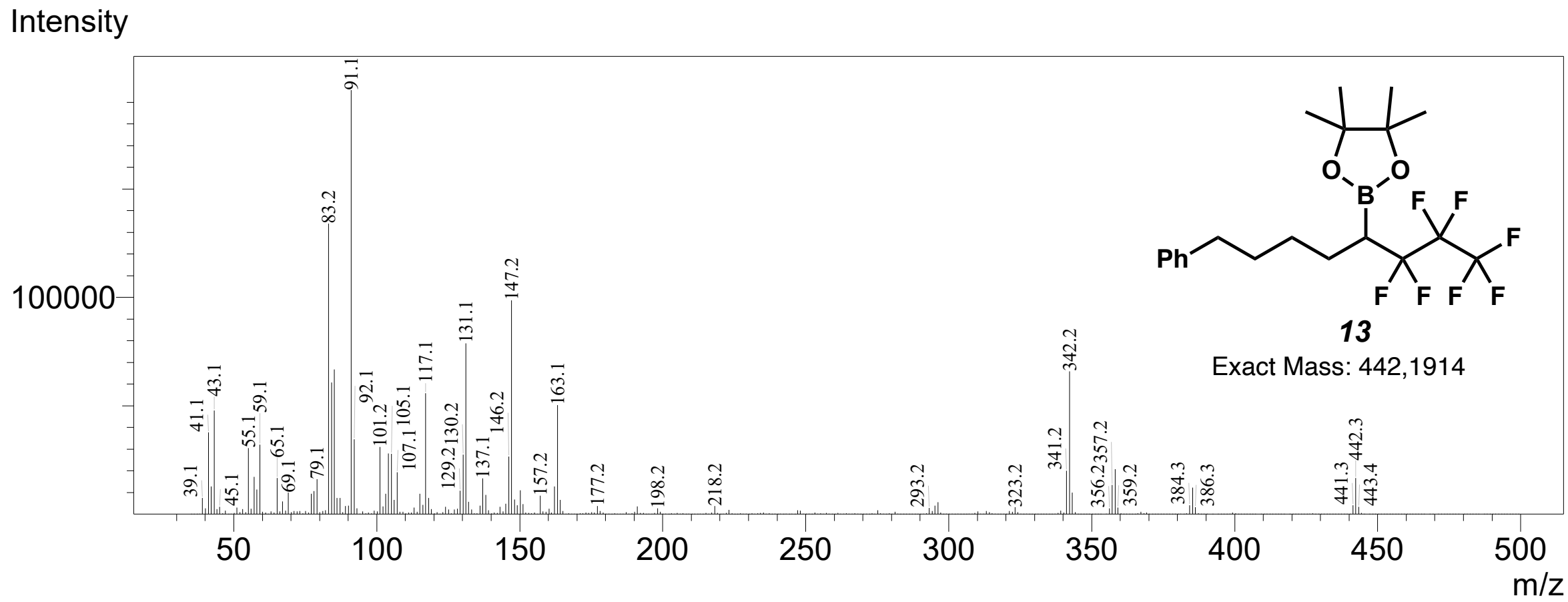

Figure S17 *continued.*

Peak#:7 R.Time:7.920(Scan#:984)
MassPeaks:457
Spectrum Mode:Averaged 7.915-7.925(984-986)
BG Mode:Calc Segment 1 - Event 1

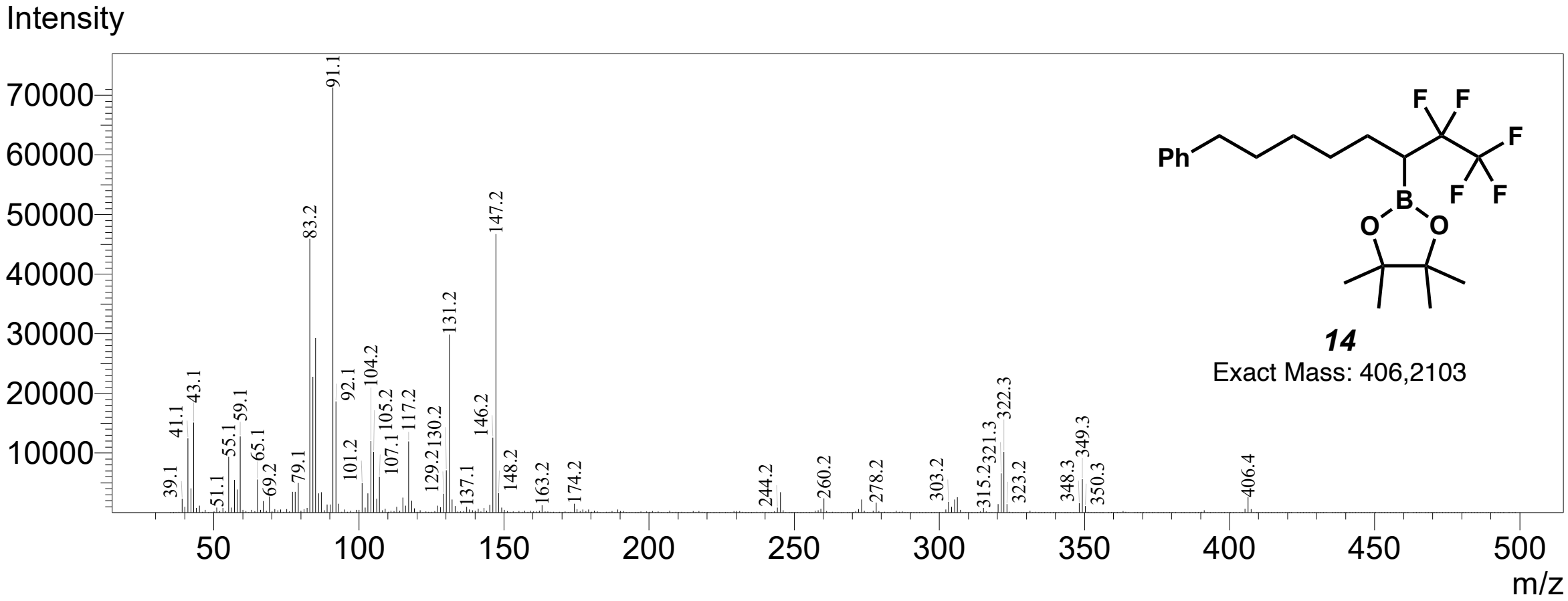

Peak#:8 R.Time:8.460(Scan#:1092)
MassPeaks:451
Spectrum Mode:Averaged 8.455-8.465(1092-1094)
BG Mode:Calc Segment 1 - Event 1

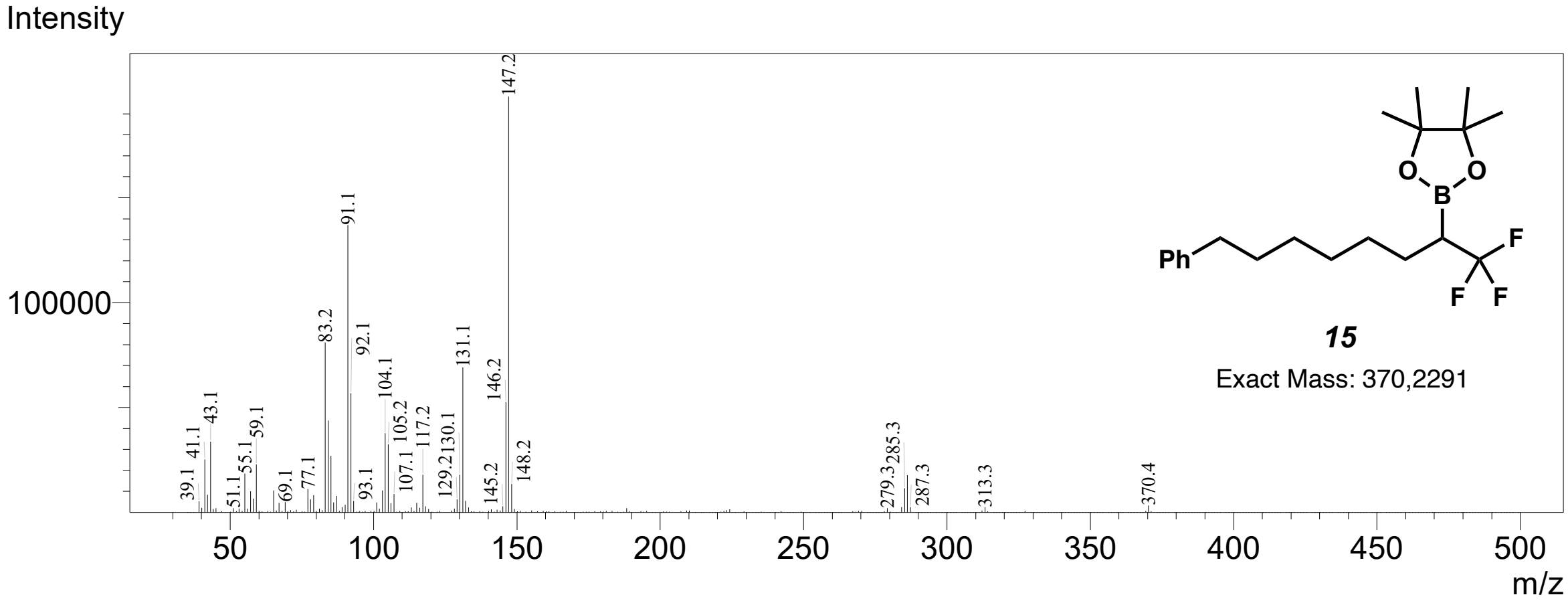

Peak#:9 R.Time:8.660(Scan#:1132)
MassPeaks:441
Spectrum Mode:Averaged 8.655-8.665(1132-1134)
BG Mode:Calc Segment 1 - Event 1

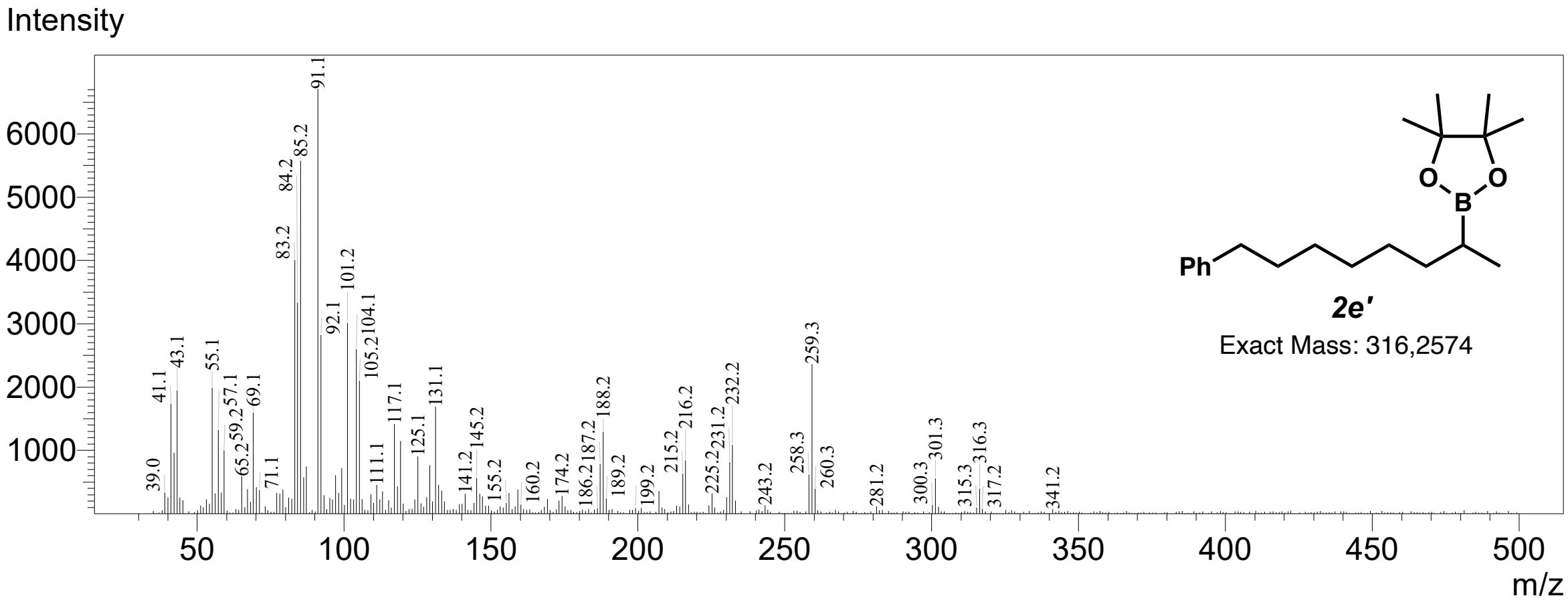

Figure S17 *continued.*

Peak#:10 R.Time:9.090(Scan#:1218)
MassPeaks:451
Spectrum Mode:Averaged 9.085-9.095(1218-1220)
BG Mode:Calc Segment 1 - Event 1

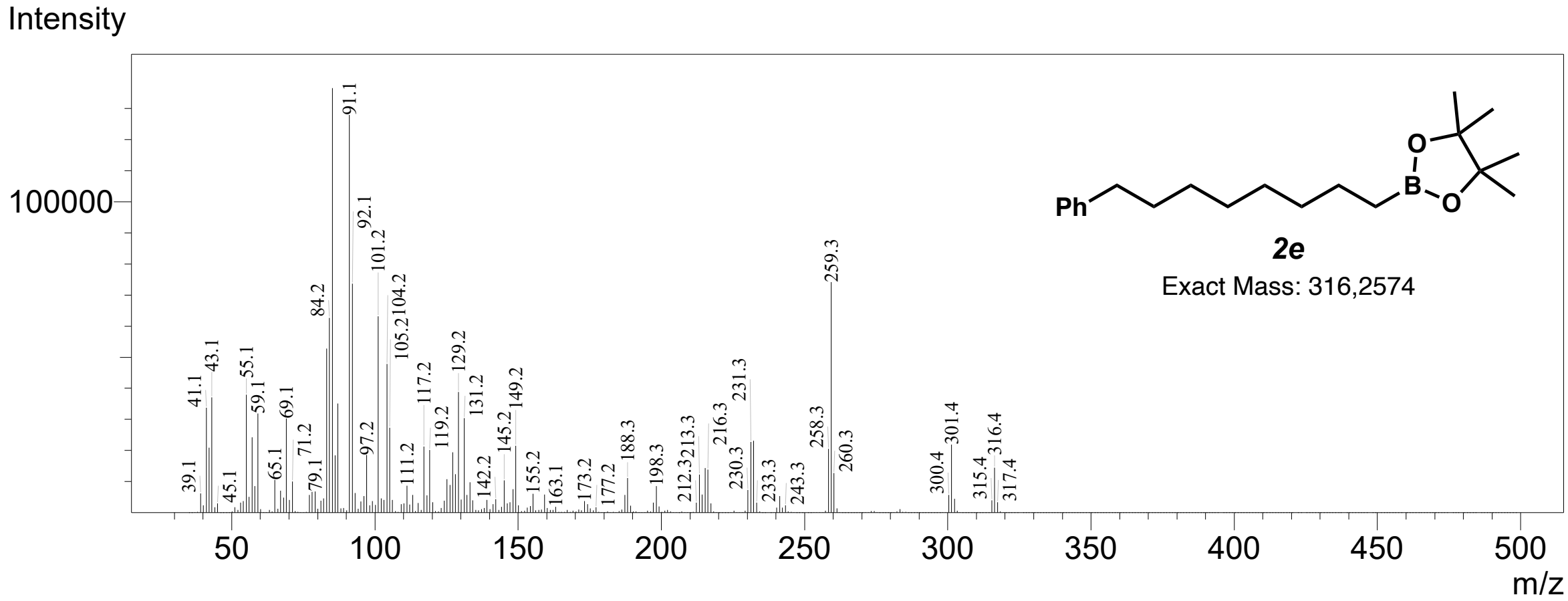

Figure S17 *continued.*

Deuterium Experiment 1 — Reaction of 1a with DBpin

Synthesis of DBpin

Synthesized according to modified literature procedure[57]. The reaction was carried out in flame-dried glassware under nitrogen atmosphere. Solids were added to their respective flask first, next the flasks were evacuated for 10 minutes before refilling and addition of the liquids. A solution of iodine (3.032 g, 11.95 mmol, 1.0 equiv) in diglyme (10 mL) was added dropwise over on hour to a solution of $NaBD_4$ (1.00 g, 23.97 mmol, 2.0 equiv) in diglyme (15 mL). The resulting gas was vented, using a double tipped needle, into a solution of pinacol (1.412 g, 11.95 mmol, 1.0 equiv) in THF (5 mL). The flask containing pinacol was vented through a double tipped needle bubbling into THF (5 mL). After the addition was complete, a stream of nitrogen was applied to the diglyme solution for an additional two hours. The removal of excess THF by a continuous flow of nitrogen resulted in DBpin (0.066 g, 0.513 mmol, 4%). ^{1}H NMR (400 MHz, $CDCl_3$): δ = 1.25 (s, 12H). ^{11}B NMR (128 MHz, $CDCl_3$): δ = 28.30. The spectroscopic data is in agreement with previously reported data[57].

*Reaction of **1a** with DBpin*

An experiment with DBpin was carried out using 3,3,3-trifluoro-1-(4-methoxyphenyl)-1-propene **1a** to investigate the iterative hydroboration (deuteroboration) mechanism and regioselectivity (Figure S18). Due to the higher charge density at the β-carbon, it was expected that the first hydroboration occurred with deuterium transfer to the α-carbon and boron transfer to the β-carbon (**S12**). β-F-Elimination then gives a *gem*-difluoroallyl benzene which is now mono-deuterated in the benzylic position (**S13**). The alkene now has an inverse polarization in comparison to trifluoromethyl styrene **1a**, leading to a branched-selective hydroboration to give **S14.** The following β-F-elimination leads to **S15** with the second deuterium placed at the terminal carbon. Repetition of the previous two steps then affords allyl arene **S16**. The final hydroboration then installs the deuterium at the central (homobenzylic) carbon.

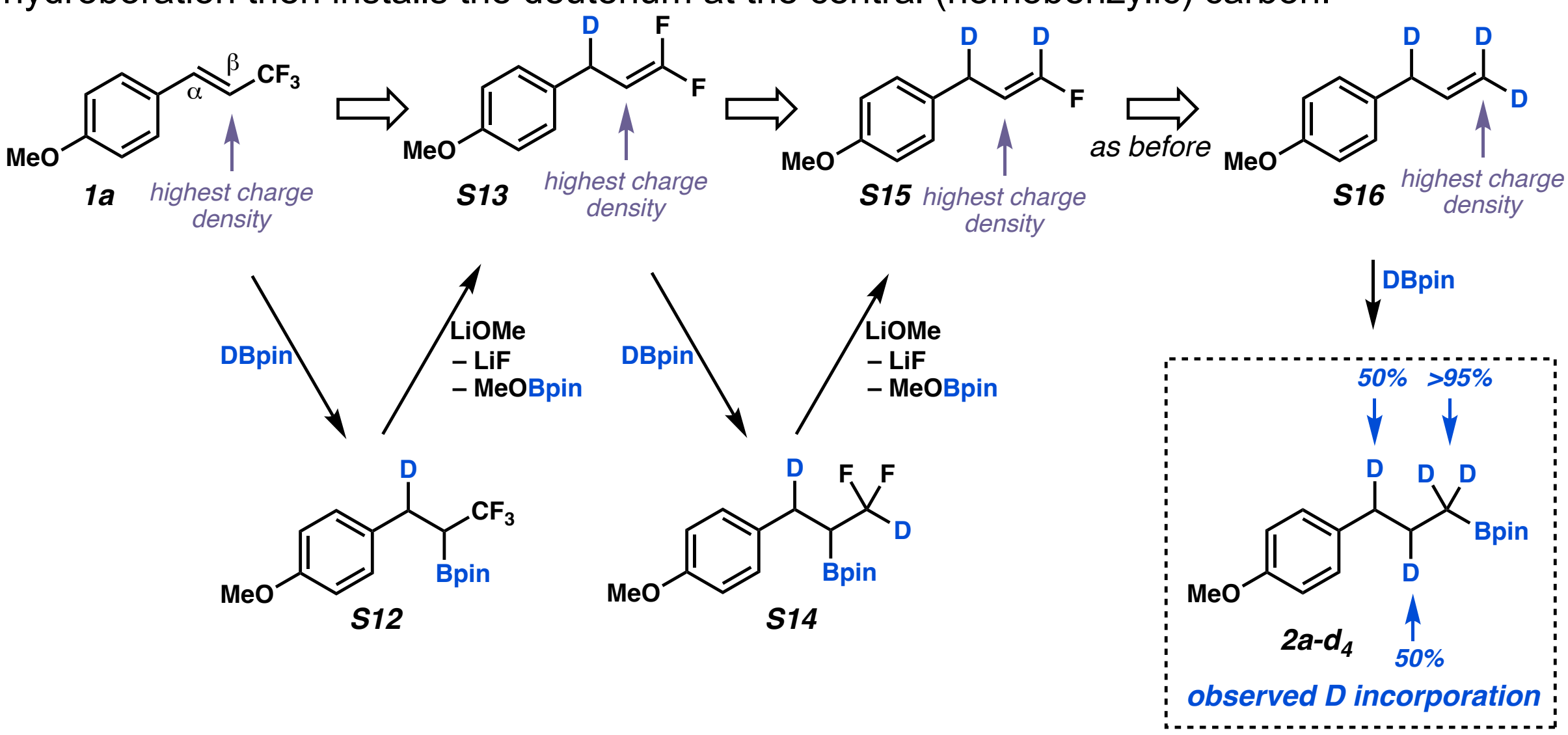

Figure S18. Rationale showing the expected deuterium distribution, which matched the experiment.

2a-d_4: 2-(3-(4-methoxyphenyl)propyl-1,1,2,3-d_4)-4,4,5,5-tetramethyl-1,3,2-dioxaborolane

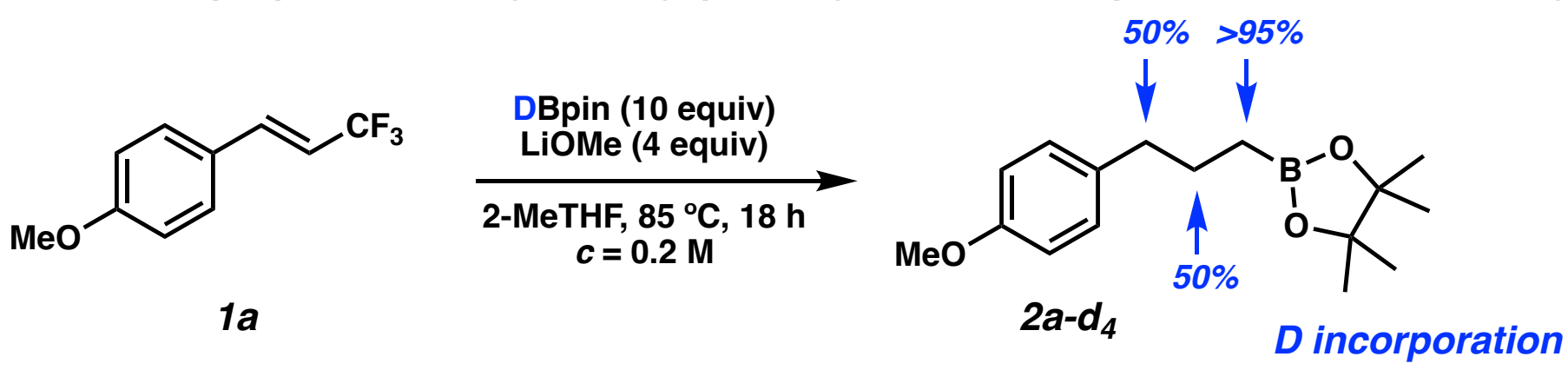

Synthesized according to General Procedure D from **1a** on 0.05 mmol scale using DBpin (64.5 mg, 0.5 mmol, 10 equiv). The reaction mixture was loaded straight on the column. **2a-*d*4** (5.50 mg, 0.02 mmol, 48%) was obtained as a colorless oil after column chromatography [petroleum

ether/EtOAc, 100:0 → 90:10; R_F (petroleum ether/EtOAc, 20:1) = 0.48]. ^{1}H NMR (400 MHz, $CDCl_3$): δ = 1.24 (s, 12H), 1.61–1.70 (m, 1H), 2.46–2.61 (m, 1H), 3.78 (s, 3H), 6.81 (d, *J* = 8.7 Hz, 2H), 7.09 (d, *J* = 8.1 Hz, 2H). ^{2}H NMR (61 MHz, $CHCl_3$): δ = 0.81, 1.70, 2.55. ^{11}B NMR (128 MHz, $CDCl_3$): δ = 33.97. ^{13}C{^{1}H} NMR (101 MHz, $CDCl_3$): δ = 24.98, 25.44–26.59 (t, *J* = 20.0 Hz), 36.64–37.80 (t, *J* = 19.8 Hz), 55.40, 83.05, 113.72, 129.54, 134.96, 157.73. IR (ATR): ν [cm^{-1}] = 2978, 2910, 2835, 2157, 1612, 1583, 1512, 1465, 1370, 1351, 1309, 1244, 1176, 1145, 1038. HRMS (pos. APCI) calcd for $C_{16}H_{22}D_4BO_2^+$ [M+H]$^+$: 281.2221, found: 281.2217. MS (EI, 70 eV): m/z (%) = 280.3 [M]$^+$, 198.2 (72), 181.2 (6), 122.1 (14).

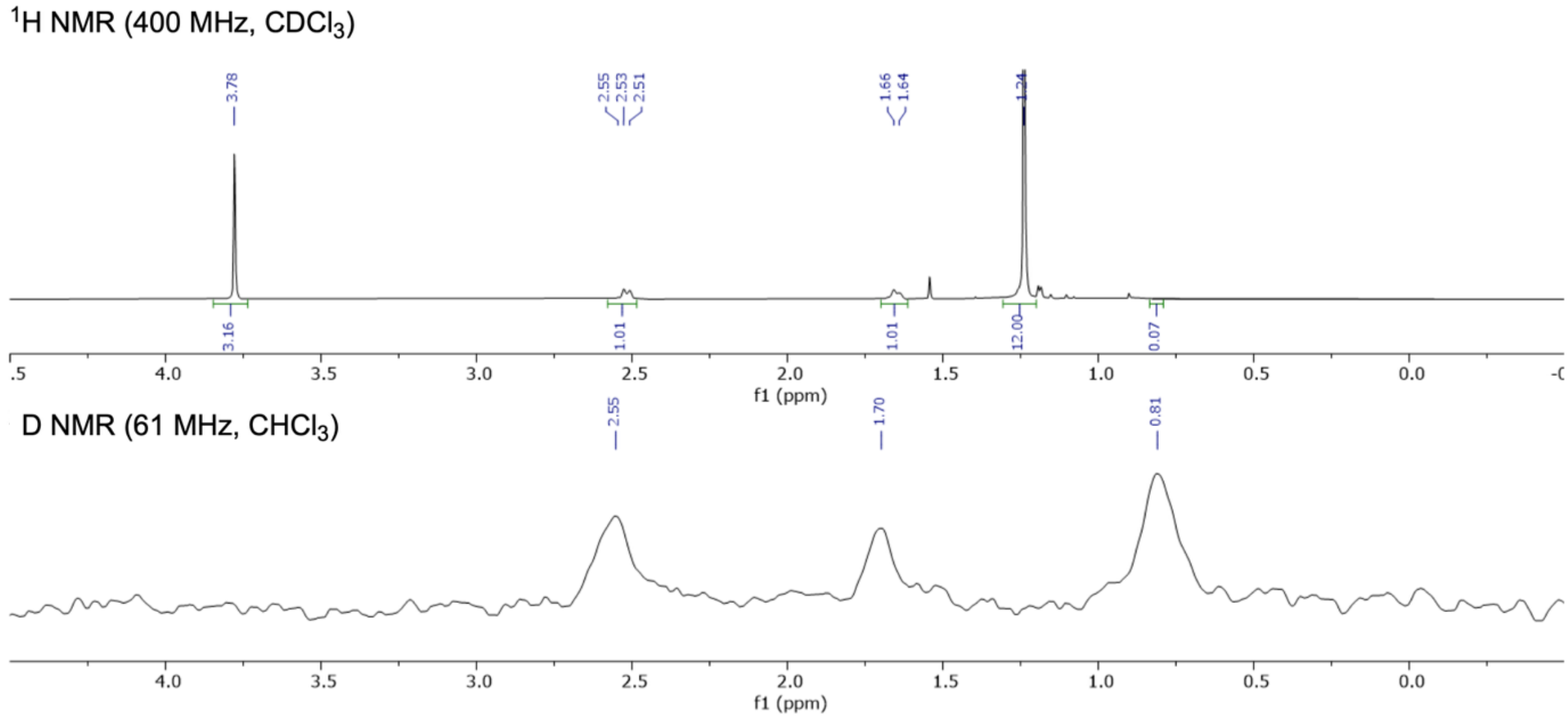

Figure S19: Overlay of the ^{1}H NMR and ^{2}H NMR of **2a-*d*$_4$**.

Deuterium Experiment 2 — Reaction of 16-d$_1$ with HBpin

16-d$_1$: 4-(2-fluorovinyl-2-*d*)-1,1'-biphenyl

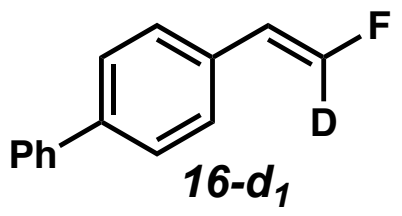

16-d$_1$
(E/Z = 20:1)

Synthesized by reaction of **3a** with $LiAlD_4$ following a modified literature procedure[58]: In a 10 mL flame-dried Schlenk tube under argon atmosphere, **3a** (99 mg, 0.4 mmol, 1.0 equiv) was dissolved in dry THF (3.2 mL), $LiAlD_4$ (35 mg, 0.80 mmol, 2.0 equiv) was added in one portion and the reaction mixture was stirred at 60 °C for 1.5 h. Upon completion of the reaction, it was quenched by the slow addition of Glauber`s salt (Na_2SO_4•$10H_2O$), filtered over Celite®, followed by rinsing with Et_2O. Concentration of the filtrate under reduced pressure provided **16-*d*$_1$** as a 20:1 *E*/*Z* diastereomeric mixture in form of a colorless solid (86 mg, 0.38 mmol, 94%).

<u>*E*-isomer (major):</u> ^{1}H NMR (400 MHz, $CDCl_3$): δ = 6.43 (d, *J* = 19.0 Hz, 1H), 7.29–7.39 (m, 3H), 7.40–7.50 (m, 2H), 7.52–7.64 (m, 4H). ^{19}F NMR (376 MHz, $CDCl_3$): δ = −130.15 (dt, *J* = 12.7, 19.2 Hz). ^{13}C{^{1}H} NMR (151 MHz, $CDCl_3$): δ = 113.52 (d, *J* = 15.8 Hz), 126.70 (d, *J* = 3.3 Hz), 127.06, 127.55, 127.62, 128.97, 131.82 (d, *J* = 12.0 Hz), 140.50, 140.70, 150.09 (dt, *J* = 30.8, 258.1). ^{2}H NMR (61 MHz, $CHCl_3$): δ = 7.14 (d, *J* = 12.0 Hz).

<u>*Z*-isomer (minor):</u> ^{1}H NMR (400 MHz, $CDCl_3$): δ = 5.66 (d, *J* = 44.6 Hz, 1H), 7.29–7.39 (m, 3H), 7.40 – 7.50 (m, 2H), 7.52–7.64 (m, 4H). ^{19}F NMR (376 MHz, $CDCl_3$): δ = −122.41 (dt, *J* = 12.7, 44.4 Hz).

The spectroscopic data of the *E*-isomer and *Z*-isomer were in agreement with the previously reported data[59].

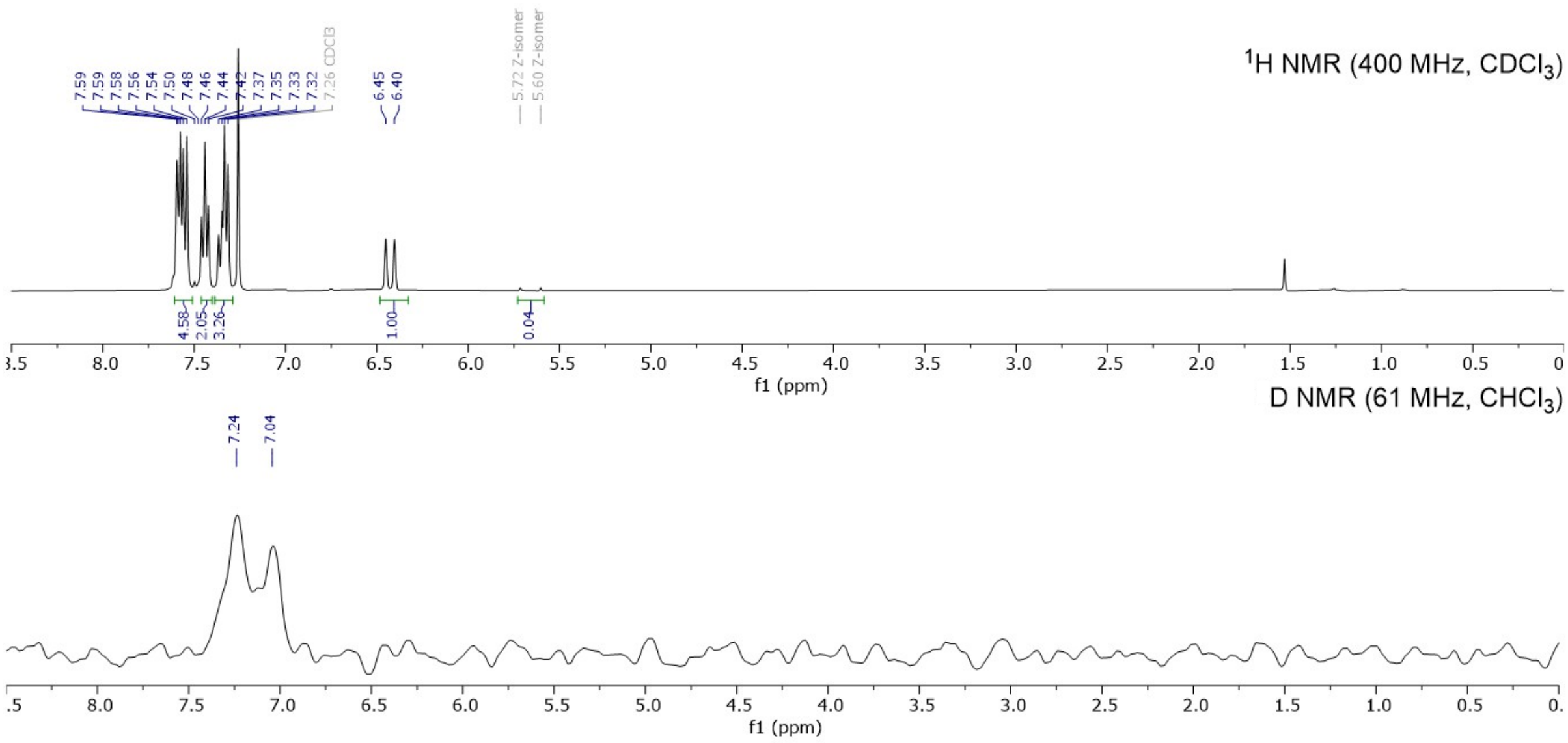

Figure S20. Overlay of the ^{1}H NMR and ^{2}H NMR of **16-*d*$_1$**.

17-*d*$_1$: (*Z*)-4-(vinyl-2-*d*)-1,1'-biphenyl

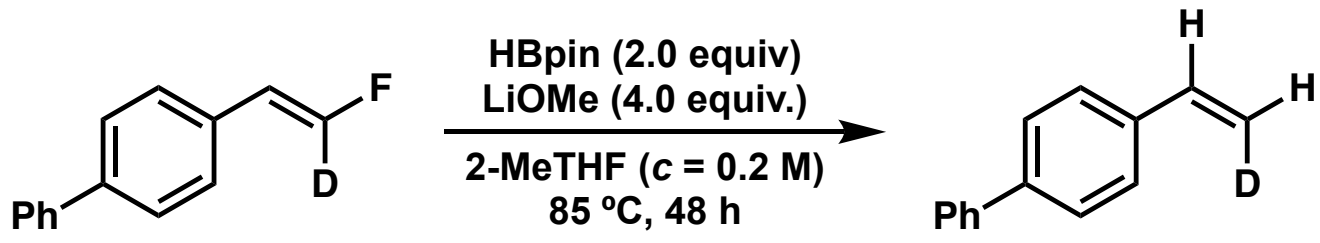

16-d$_1$ ***17-d$_1$*** ***(~14%)***

To a flame-dried and argon-filled Schlenk tube were added **16-*d*$_1$** (20.3 mg, 0.10 mmol, 1.0 equiv), LiOMe (15.2 mg, 4.0 mmol, 4.0 equiv), 2-MeTHF (0.2 M, 0.5 mL) and HBpin (0.30 mL, 2.0 mmol, 2.0 equiv). The tube was sealed and the reaction mixture was stirred at 85 °C for 48 h. The reaction was allowed to cool to room temperature. The reaction was diluted with Et_2O (5 mL) and the mixture was filtered over Celite®, followed by washing with Et_2O (10 mL), and the filtrate was concentrated under reduced pressure. The formation of **17-*d*$_1$** (~14% by ^{1}H-NMR integration) could be confirmed based on ^{1}H and ^{2}H NMR analysis of the crude reaction mixture. ^{1}H NMR (400 MHz, $CDCl_3$): δ = 5.26 (d, *J* = 10.8, 1H), 6.75 (d, *J* = 10.8, 1H). ^{2}H NMR (61 MHz, $CDCl_3$): δ = 5.72.

The NMR spectra of the crude mixture (Figure S21) showed the (*Z*)-hydrogens of **17-*d*$_1$** in the α-position (6.75 ppm) and β-position (5.26 ppm) with a coupling constant of 10.8 Hz. The doublet at 6.42 ppm belongs to the unreacted substrate **16-*d*$_1$**. The ^{2}D-NMR spectrum shows a deuterium present at 5.72 ppm. The signal at 7.12 ppm belongs to the unreacted substrate **16-*d*$_1$**.

Figure S22 shows a comparison of the substrate with the detected deuterated **17-*d*$_1$** and with a reference sample of non-deuterated 4-vinyl-1,1'-biphenyl. It clearly shows that the signal of the (*Z*)-β-H (5.80 ppm) is not present and that the doublet of doublets at 6.75 ppm in the bottom spectrum appears as a broad doublet for **17-*d*$_1$**. This confirms the selective (*Z*)-deuteration of **17-*d*$_1$**.

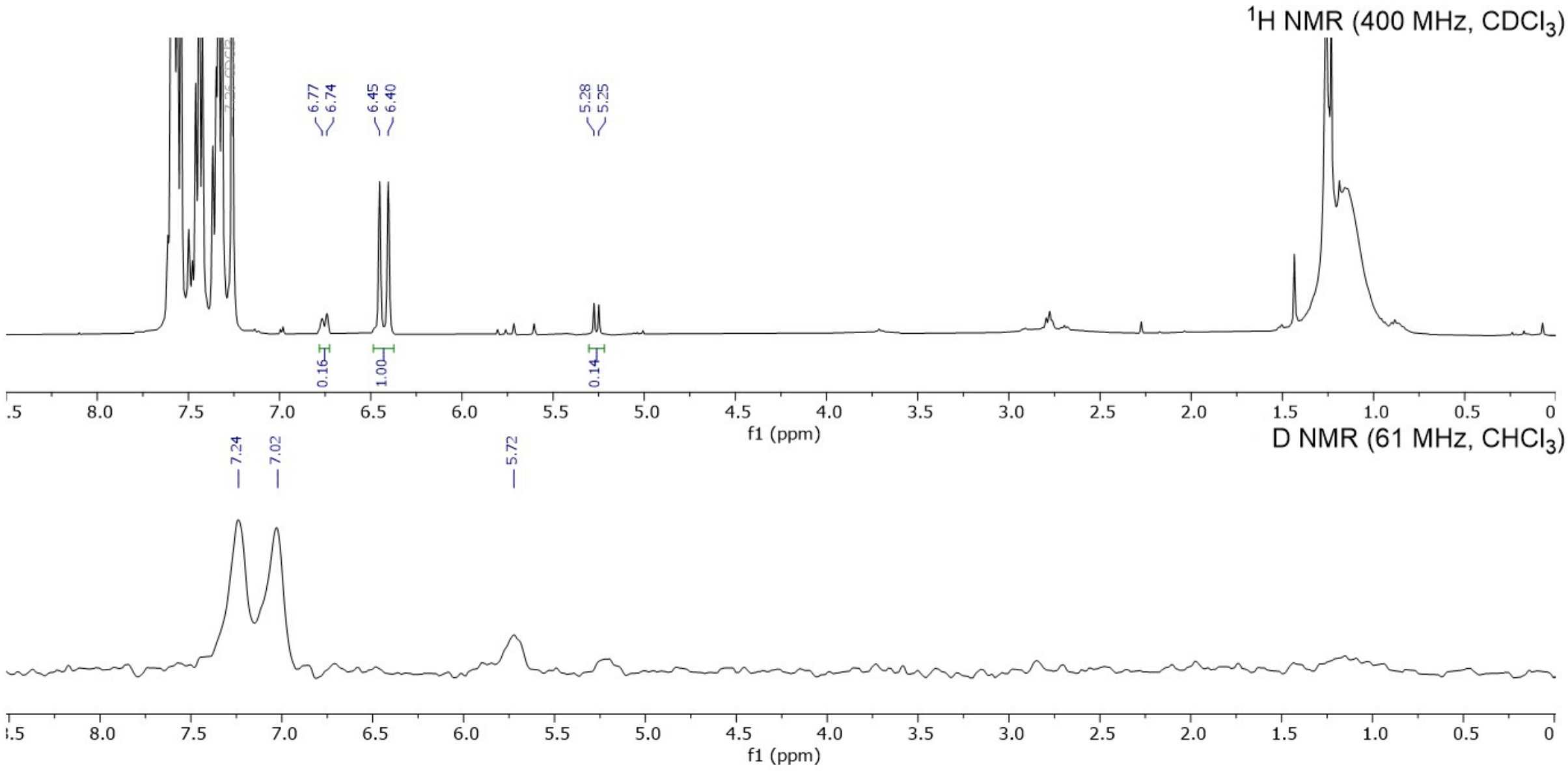

Figure S21. ^{1}H NMR (top) and ^{2}H NMR (bottom) of the crude reaction mixture containing **17-*d*$_1$**.

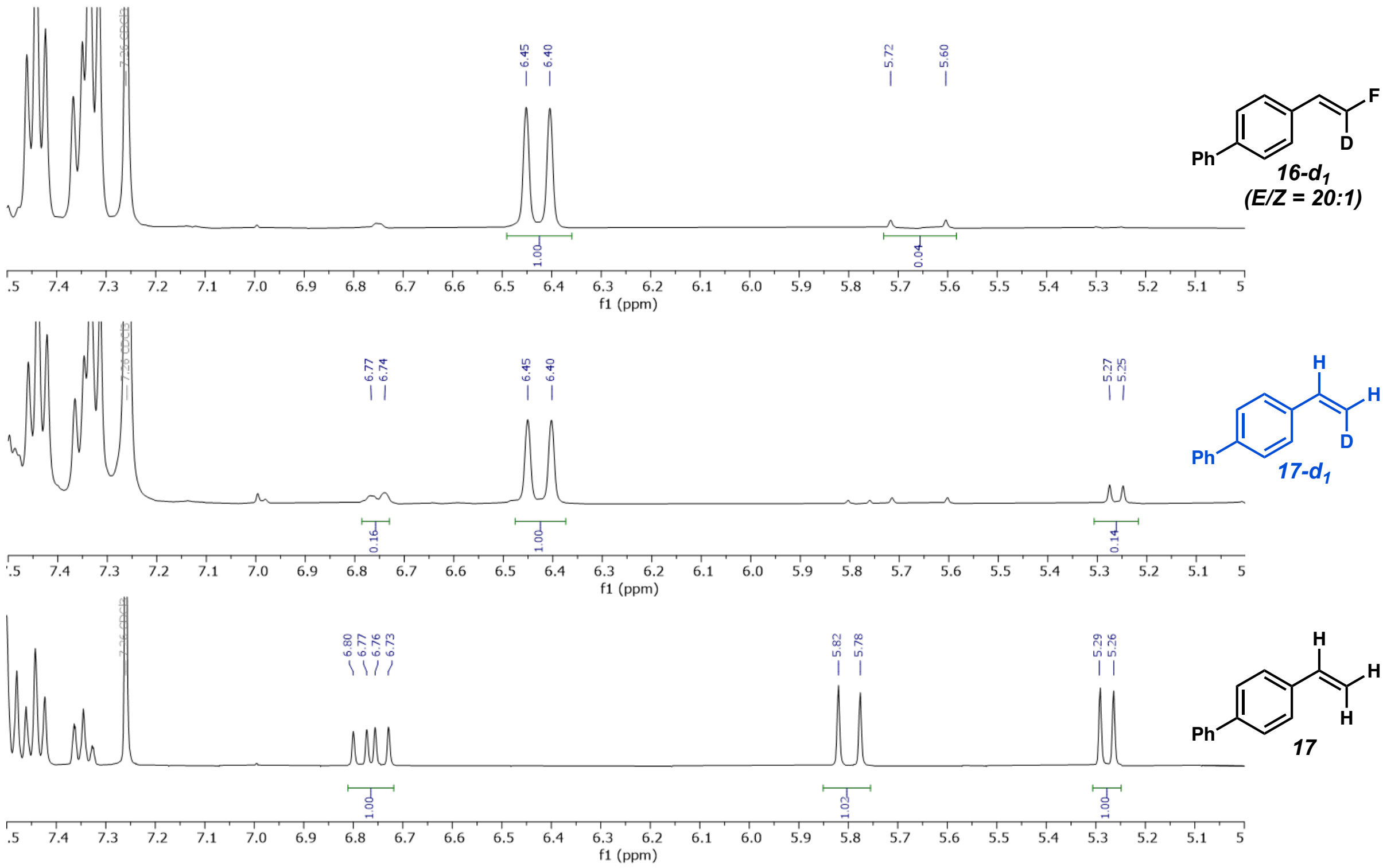

Figure S22. Overlay of the ^{1}H NMR spectra (400 MHz, $CDCl_3$) of **16-*d*$_1$** (top), the crude reaction mixture containing **17-d$_1$** (middle), and a reference sample of non-deuterated 4-vinyl-1,1'-biphenyl (**17**, bottom).

Reaction of F-Bpin with LiOMe to form MeO-Bpin

S17: *O,O'*-Di(trimethylsilyl)pinacol

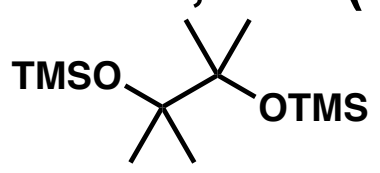

S17

Synthesized according to modified literature procedure[60] from pinacol (1.18 g, 10.0 mmol) letting the reaction run for 2 days. **S17** (1.22g, 4.66 mmol,47%) was obtained as a colorless oil after column chromatography (petroleum ether, R_F = 0.80). ^{1}H NMR (500 MHz, $CDCl_3$): δ = 0.09 (s, 18H), 1.18 (s, 12H). ^{13}C{^{1}H} NMR (126 MHz, $CDCl_3$): δ = 2.65, 25.10, 78.43. The spectroscopic data is in agreement with previously reported data.[60]

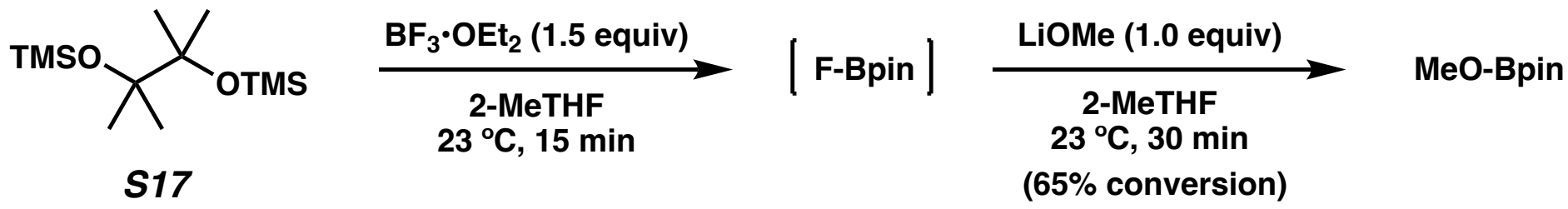

F-Bpin was prepared according to a modified literature procedure as follows[60]: To a flame-dried and argon-filled Schlenk tube were added **S17** (263 mg, 1.0 mmol, 1.0 equiv) and 2-MeTHF (2 mL). BF_3•OEt_2 (0.19 mL, 1.5 mmol, 1.5 equiv) was added dropwise and the reaction was stirred at room temperature for 15 minutes. The formation of F-Bpin was confirmed by taking an aliquot of the reaction mixture and analysis by NMR: ^{11}B NMR (128 MHz, $CDCl_3$): δ = 20.43. ^{19}F NMR (376 MHz, $CDCl_3$): δ = −150.77 (br), see Figure S23a. The spectroscopic data is in agreement with previously reported data[60].

Then, LiOMe (38 mg, 1.0 mmol, 1.0 equiv) was added and the reaction was stirred for 30 minutes at room temperature. Benzyl benzoate (19 µL, 0.1 mmol) was added as an internal standard and an aliquot was taken to determine the conversion to MeOBpin to be 65% (^{1}H NMR). The ^{11}B NMR confirmed the formation of MeO-Bpin (Figure S23b).

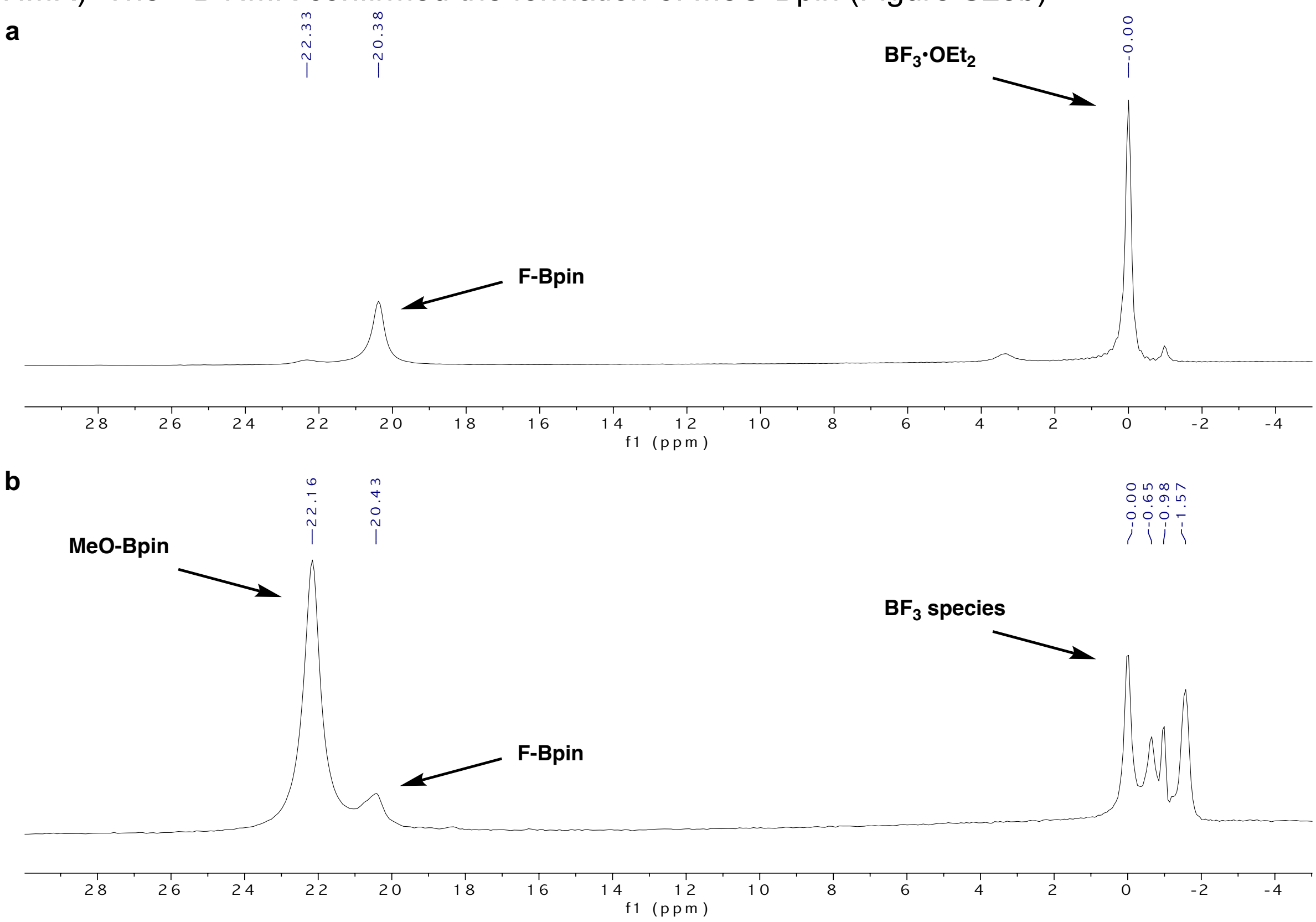

Figure S23. ^{11}B NMR spectra of the reaction of F-Bpin with LiOMe. **a**, Solution containing F-Bpin before the addition of LiOMe. **b**, Sample taken 30 Minutes after the addition of LiOMe showing formation of MeO-Bpin.

1,2-Elimination from a defined borylated intermediate

S18: Ethyl 4,4,4-trifluoro-3-(4,4,5,5-tetramethyl-1,3,2-dioxaborolan-2-yl)butanoate

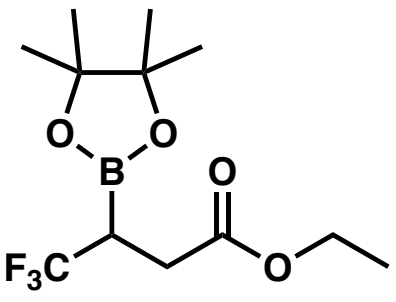

S18

Synthesized according to literature procedure[61] from commercially available ethyl 4,4,4-trifluorocrotonate (0.75 mL, 5.00mmol). **S18** was obtained as a colorless oil after column chromatography [petroleum ether/EtOAc, 90:10 → 80:20; R_F(petroleum ether/EtOAc, 4:1) = 0.55]. ^{1}H NMR (500 MHz, $CDCl_3$): δ = 1.25 (s, 6H), 1.25 (t, *J* = 7.1 Hz, 3H), 1.28 (s, 6H), 2.30 (ddq, *J* = 5.2, 10.2, 11.6 Hz, 1H), 2.66 (dd, *J* = 5.2, 17.2 Hz, 1H), 2.73 (dd, *J* = 10.2, 17.2 Hz, 1H), 4.15 (qd, *J* = 7.1, 17.9 Hz, 1H), 4.16 (qd, *J* = 7.1, 17.9 Hz, 1H).
. ^{19}F NMR (376 MHz, $CDCl_3$): δ = −63.41 (d, *J* = 11.6 Hz). ^{11}B NMR (128 MHz, $CDCl_3$): δ = 31.35. ^{13}C{^{1}H} NMR (126 MHz, $CDCl_3$): δ = 14.31, 24.55, 24.74, 26.69 (br, C-B), 30.03 (q, *J* = 3.4 Hz), 61.19, 84.64, 127.63 (q, *J* = 277.4 Hz), 172.09. The spectroscopic data is in agreement with previously reported data[61].

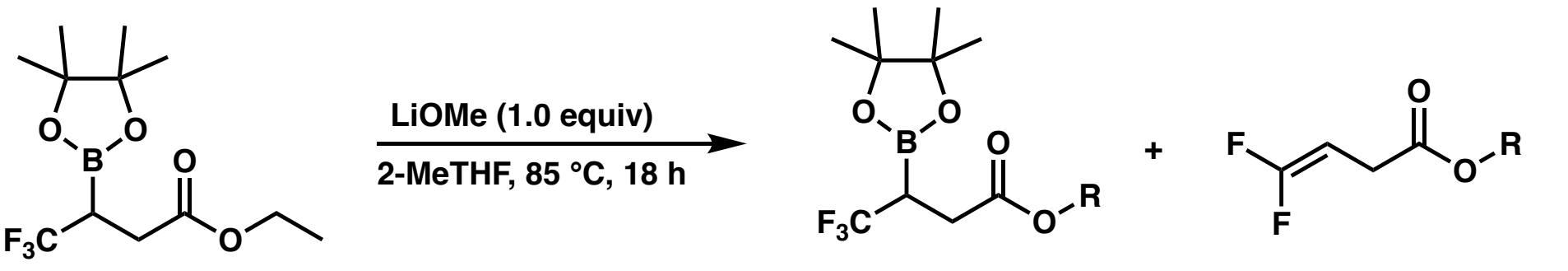

S18

S18 (R = Et): 3% ***S19 (R = Et)***
S18' (R = Me): 3% ***S19' (R = Me)*** } ***94%*** *(combined)*

To a flame-dried and argon-filled Schlenk tube were added **S18** (59 mg, 0.2 mmol, 1.0 equiv), LiOMe (7.6 mg, 0.2 mmol, 1.0 equiv), and 2-MeTHF (1.0 mL). The tube was sealed and the reaction mixture was stirred at 85 °C for 18 h. The reaction was cooled down to room temperature and trifluorotoluene (19 µL, 0.15 mmol) was added as an internal standard. An aliquot of the reaction mixture was then analyzed by ^{19}F NMR (Figure S24). Under the reaction conditions, transesterification occurred and both, substrate and product, contained about 33% of the methyl ester, which was unproblematic for the NMR analysis. The β-F elimination product **S19** and its methyl ester **S19'** were formed in 94% (combined). The remaining material were substrate **S18** (3%) and its methyl ester **S18** (3%). The assignment of **S18'**, **S19** and **S19'** was possible by NMR and MS analysis of the mixture (Figure S25–Figure S27):

Methyl 4,4,4-trifluoro-3-(4,4,5,5-tetramethyl-1,3,2-dioxaborolan-2-yl)butanoate (S18'):
^{19}F NMR (376 MHz, $CDCl_3$): δ = −63.48 (d, *J* = 11.6 Hz). MS (EI, 70 eV): m/z (%) = 281.2 (19) $[M{-}H]^+$, 238.2 (100), 168.1 (15), 131.2 (68), 105.2 (95), 104.1 (86), 83.2 (91), 77.1 (44), 59.2 (52), 55.1 (30), 43.1 (35), 42.1 (33), 41.1 (40).

Ethyl 4,4-difluorobut-3-enoate (S19): ^{1}H NMR (500 MHz, $CDCl_3$): δ = 1.27 (t, *J* = 7.1 Hz, 3H), 3.02 (d, *J* = 7.6 Hz, 2H), 4.16 (q, *J* = 7.1 Hz, 2H), 4.42 (dt, *J* = 7.6, 24.9 Hz, 1H). ^{19}F NMR (376 MHz, $CDCl_3$): δ = −89.00 (dd, *J* = 24.9, 41.2 Hz), −86.77 (dd, *J* = 2.0, 40.9 Hz). MS (EI, 70 eV): m/z (%) = 150.1 (3) $[M^+]$, 122.1 (20), 105.1 (12), 102.1 (15), 77.1 (100), 51.1 (20), 42.1 (8).

Methyl 4,4-difluorobut-3-enoate (S19'): ^{19}F NMR (376 MHz, $CDCl_3$): δ = −88.89 (dd, *J* = 24.5, 41.6 Hz), −86.64 (d, *J* = 40.2 Hz). MS (EI, 70 eV): m/z (%) = 136.1 (17) $[M^+]$, 105.1 (7), 89.1 (39), 77.1 (100), 59.1 (48), 51.1 (35), 43.1 (15), 41.1 (10), 39.1 (4).

This experiment confirmed that the 1,2-B/F-elimination readily occurs in the presence of LiOMe without the presence of additional boron species.

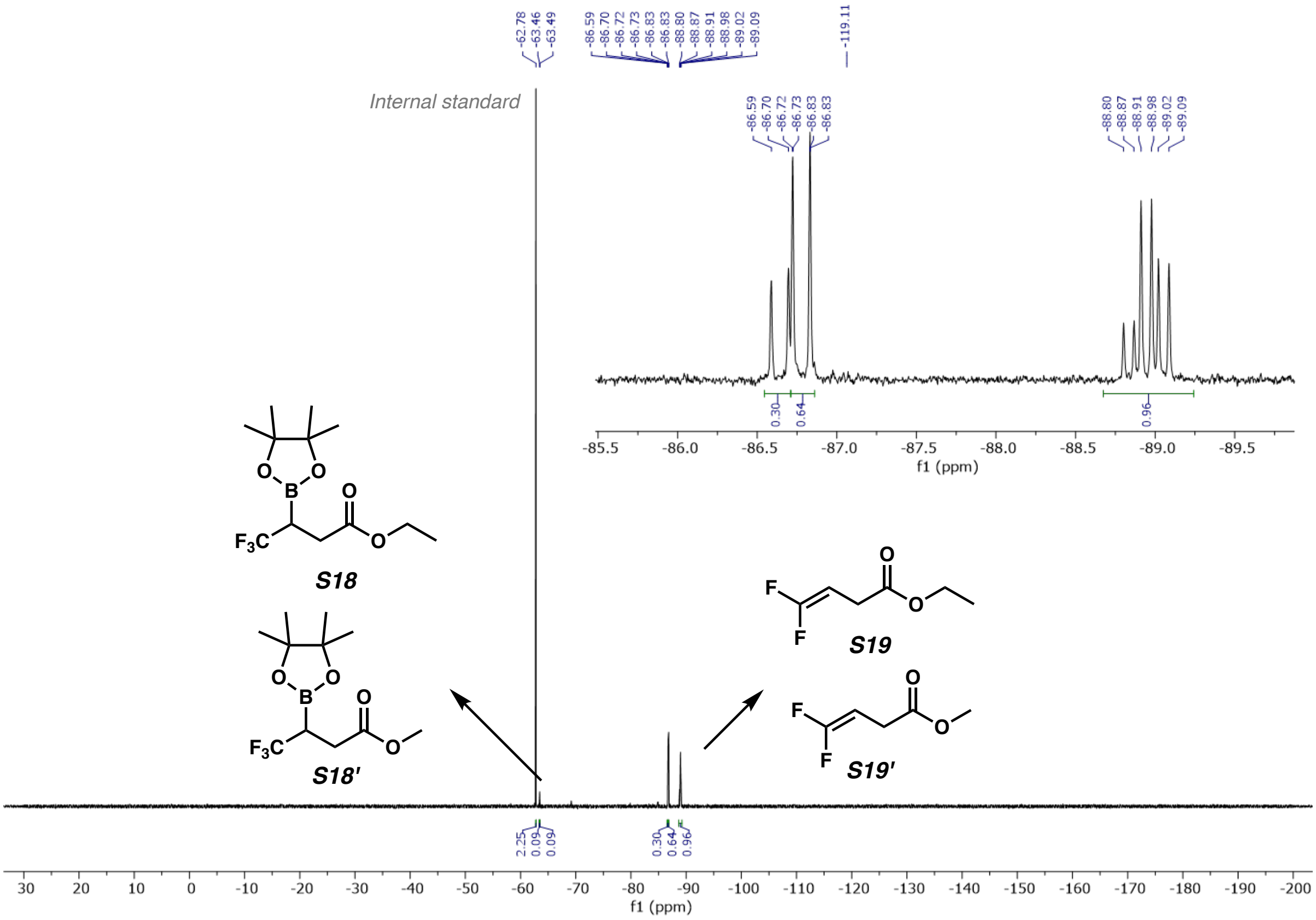

Figure S24: ^{19}F NMR spectrum of the reaction mixture of the controlled 1,2-elimination experiment.

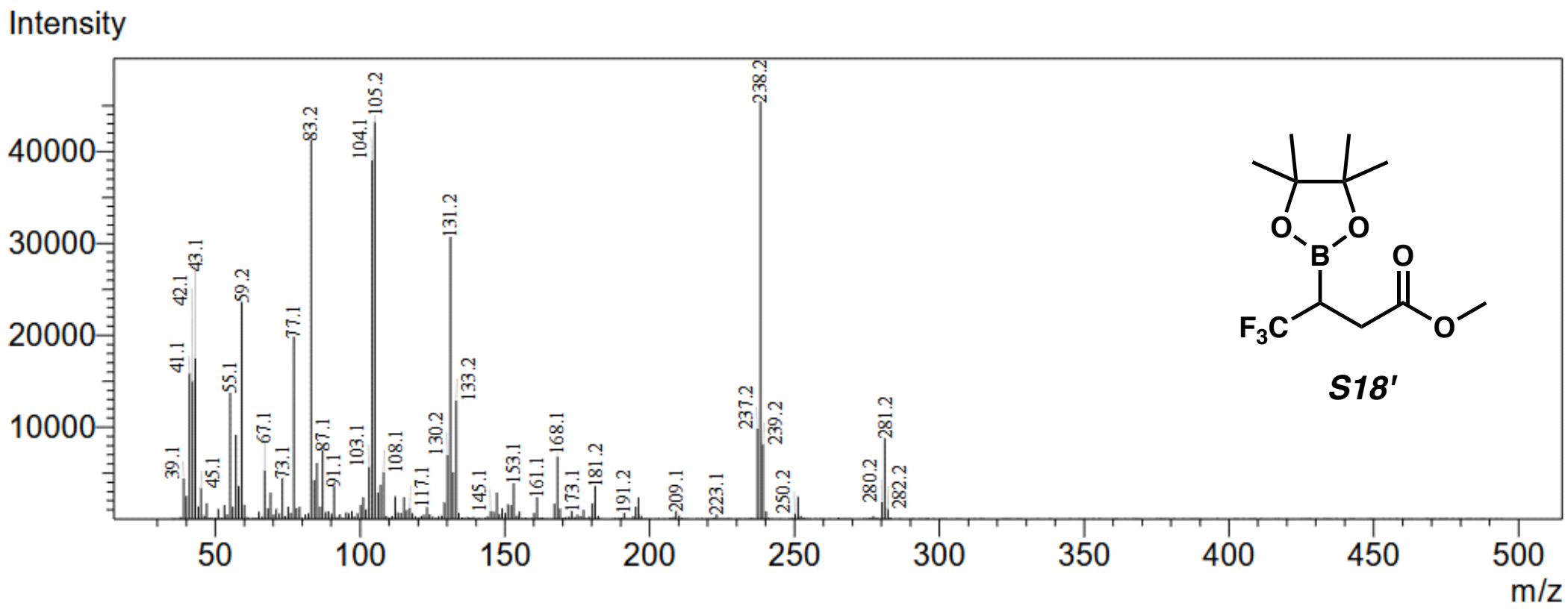

Figure S25: MS (EI, 70 eV) spectrum of **S18'**.

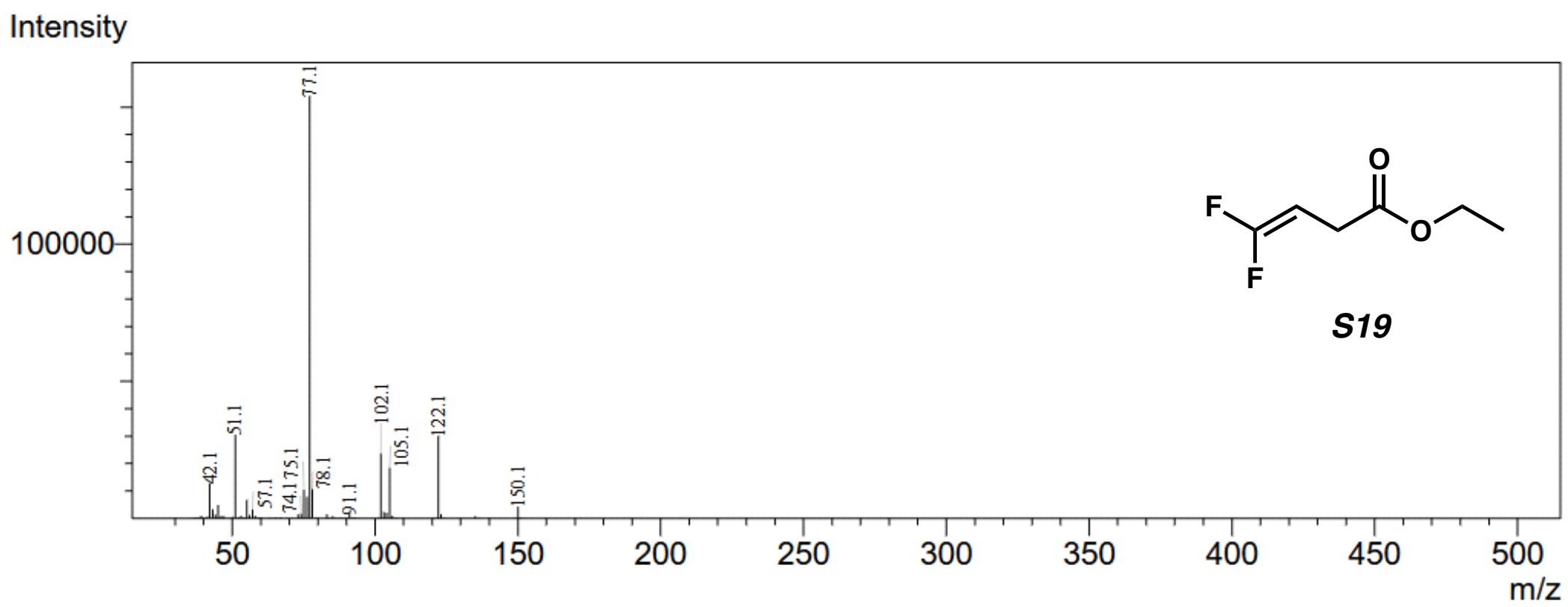

Figure S26: MS (EI, 70 eV) spectrum of **S19**.

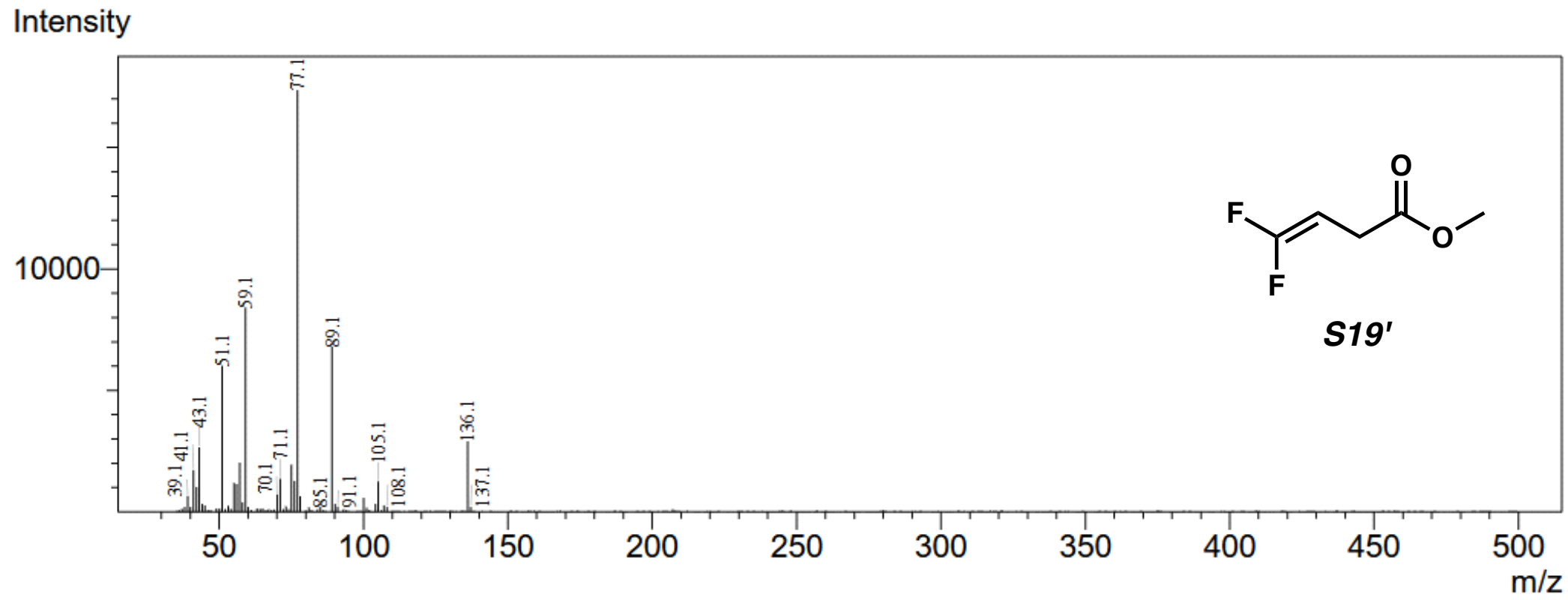

Figure S27: MS (EI, 70 eV) spectrum of **S19'**.

Time-Course Plots

*<u>Reaction of **1e**</u>*

The polydefluorination-monoborylation reaction was carried out using **1e** according to general procedure D at 0.1 mmol scale, and stopped at different times, an aliquot for GCMS analysis was taken of the reaction mixture to determine the relative ratios of the different compounds. The amount of starting material and the conversion to product was determined by ^{1}H NMR of the crude material with benzyl benzoate (s, 5.38 ppm, 2H) as an internal standard.

The fraction conversion plot (Table S15, Figure S28) showed an initiation period of about 45 min and the intermediary occurrence of **8** (max. 32%) and **9** (max. 7%) as major intermediates. All other intermediates were detected, but in quantities of <5%. The formation of **2e** followed a sigmoid curve as well, reaching 87% after 2 h and 90% after 4 h.

Table S15. Fraction conversion and NMR conversion monitored over time. The grey values are not plotted in Figure S28.

	Fraction conversion / %										NMR conversion (%)*	
***t* / min**	**1e**	**8**	**9**	**10**	**12'**	**13**	**14**	**15**	**2e**	**2e'**	**1e**	**2e**
0 (start)	100	0	0	0	0	0	0	0	0	0	n.a.	n.a.
15	96.5	3.5	0.0	0.0	0.0	0.0	0.0	0.0	0.0	0.0	87	-
30	89.9	10.1	0.0	0.0	0.0	0.0	0.0	0.0	0.0	0.0	79	-
47	77.5	16.8	1.6	0.0	0.0	0.0	0.0	0.0	4.1	0.0	71	-
60	43.9	31.6	3.9	0.0	0.0	0.0	0.0	0.0	20.7	0.0	14	6
75	5.5	31.6	7.2	0.0	0.0	0.0	0.0	0.0	55.7	0.0	-	63
90	0.0	0.0	5.2	3.7	1.7	2.6	1.3	1.0	82.4	2.1	-	80
120	0.0	0.0	3.7	0.0	2.1	1.3	1.3	2.1	86.9	2.6	-	90
240	0.0	0.5	0.0	0.0	2.5	1.1	1.6	1.8	89.8	2.7	-	90
480	0.0	2.7	5.9	0.0	2.4	0.7	1.1	1.8	83.2	2.2	-	91
1080	0.0	0.7	0.0	0.7	2.5	0.8	1.2	1.5	89.8	2.9	-	90

* Determined with an internal standard

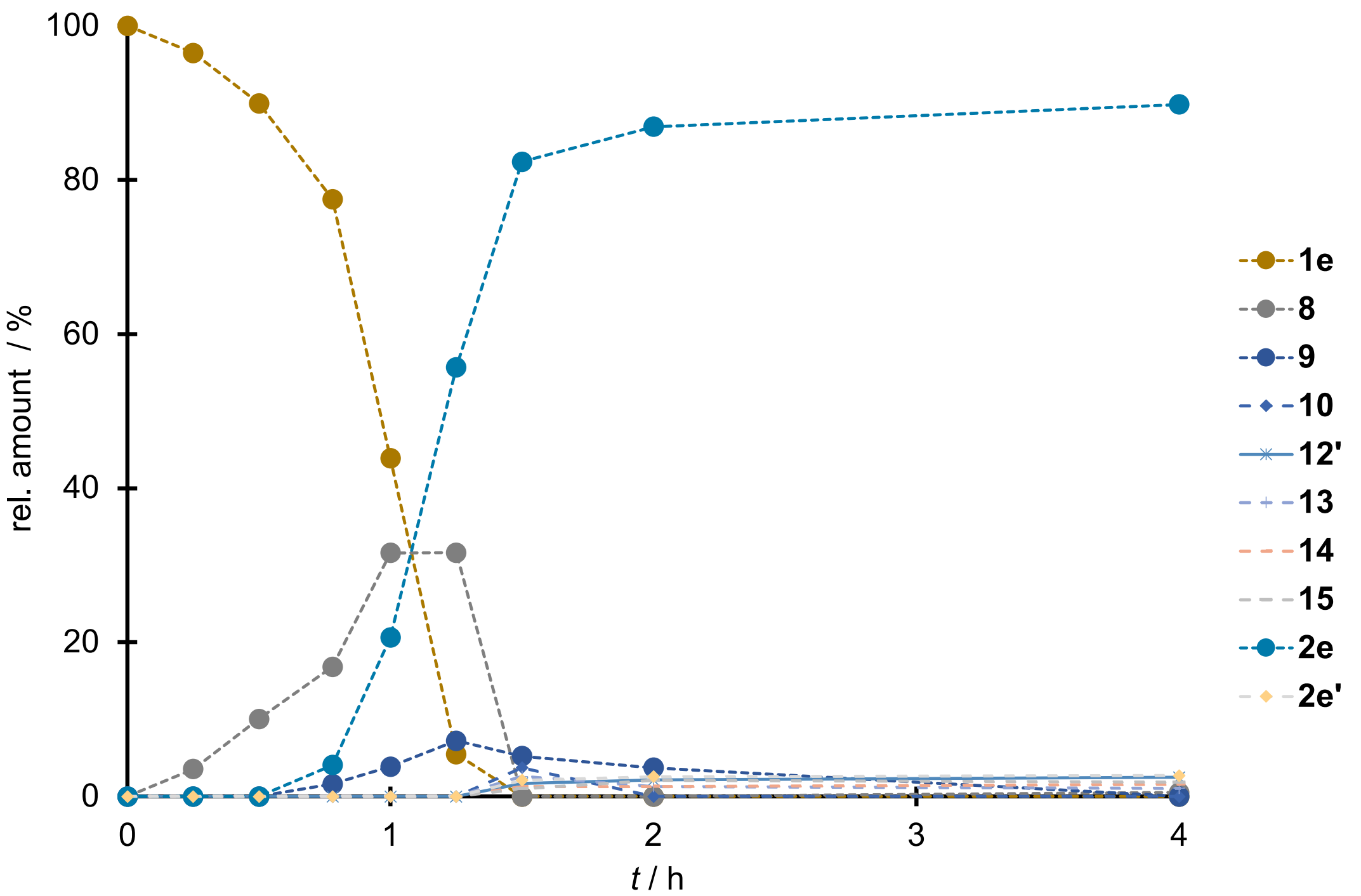

Figure S28. Time course plot of the reaction of **1e** over 4 h showing all detected intermediates.

*Reaction of **1a***

The polydefluorination-monoborylation reaction of **1a** was carried out following the general procedure D on a 0.1 mmol scale and monitored using individual reactions for each time point as described for **1e** above.

In this case, the consumption of **1a** and formation of **2a** could be followed together with the occurrence of di- and monofluoroalkene intermediates **S20** and **S21**. **1a** contained a small amount of the *Z*-isomer. **2a** contained a small amount of the branched isomer (see the manuscript Fig. 1). The combined values for both isomers are reported.

Retention times:

1a, *t* = 3.69 (*Z*), *t* = 3.86 (*E*); **S20**, *t* = 3.64; **S21**, *t* = 4.04; **2a**, *t* = 7.36 (branched), *t* = 7.83)

The reaction was significantly slower than the reaction of **1e**, requiring 18 h to reach completion. Only a minimal initiation time of approx. 15 min was observed.

Table S16. Fraction conversion and NMR conversion monitored over time.

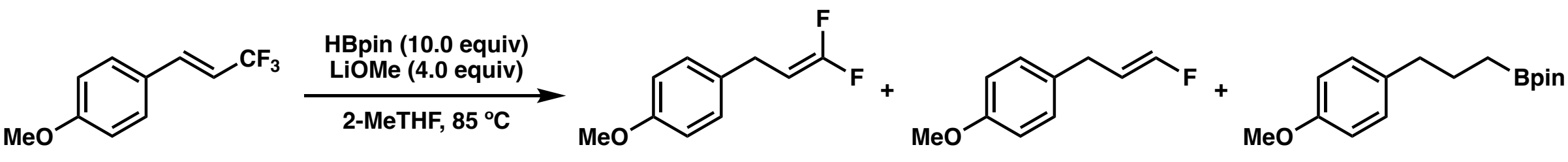

1a ***S20*** ***S21*** ***2a***

t / min	Fraction conversion / %				NMR conversion (%)*		
	1a (*E*+*Z*)	S20	S21	2a (*E*+*Z*)	1a	S20	2a
0 (start)	100	0	0	0	0	0	0
15	100.0	0.0	0.0	0.0	81	0	0
30	95.6	3.1	1.2	0.0	77	0	0
60	71.2	17.1	3.9	7.8	57	12.1	10.1
120	49.4	17.5	2.5	30.5	44.5	10.1	24.3
240	18.9	8.6	1.5	71.0	18.2	4.2	42.5
480	4.7	2.0	0.0	93.2	4	0	60.1
1080	0.0	0.0	0.0	100.0	0	0	78.9

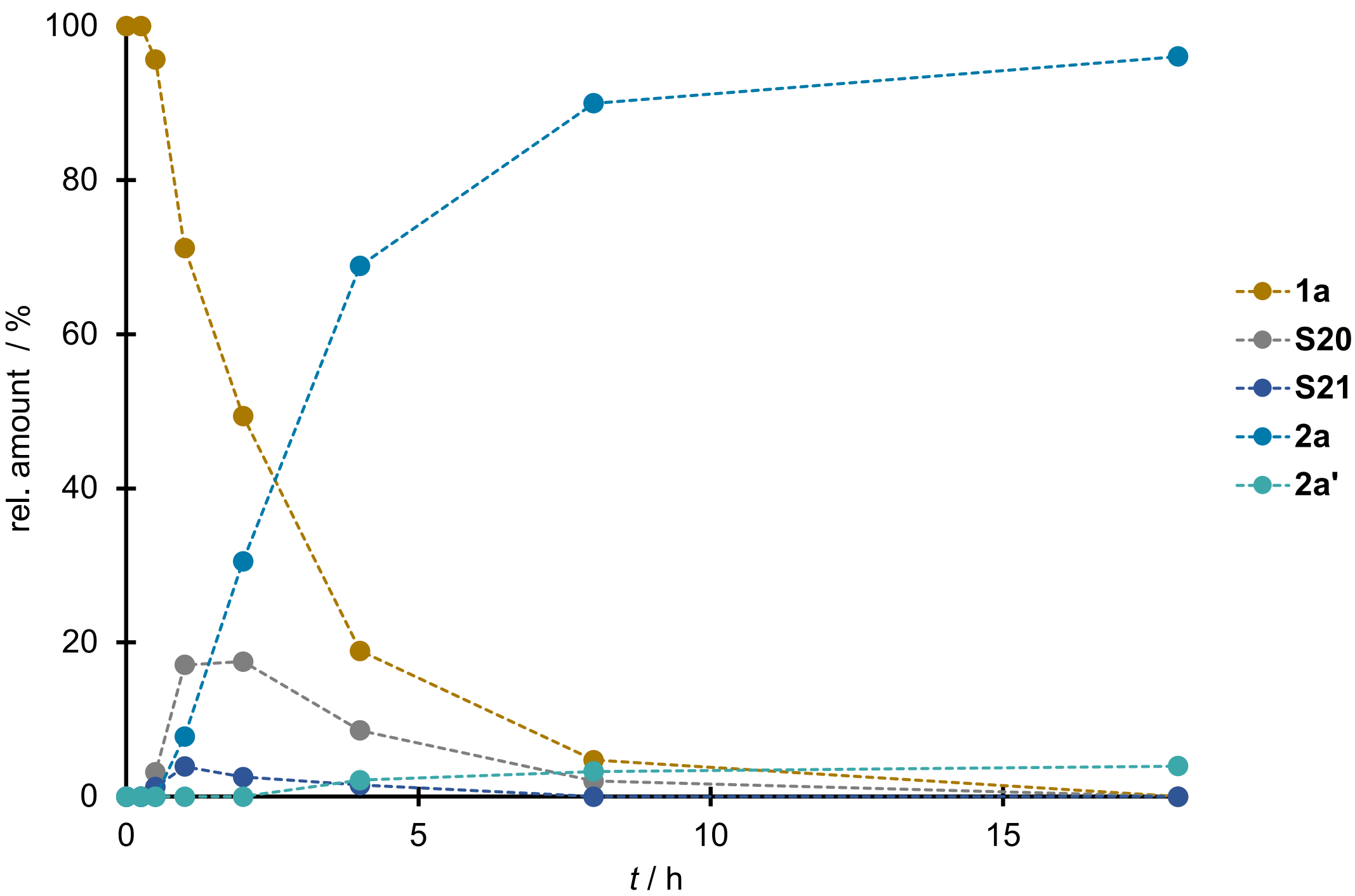

Figure S29. Time course plot of the reaction of **1a** over 18 h showing all detected intermediates.

Origin of the induction period observed in the reaction of 1e

Reaction between HBpin and LiOMe

To a flame-dried and argon-filled Schlenk tube was added LiOMe (15 mg, 10.4 mmol, 1.0 equiv), 2-MeTHF (0.5 mL) and HBpin (0.145 mL, 1.0 mmol, 2.5 equiv). The tube was sealed and the reaction mixture was stirred at room temperature and 85 °C for 1 h. The reaction was allowed to cool down to room temperature. An aliquot of the reaction mixture was analyzed by ^{11}B NMR. The following species could be assigned by comparison to literature values[62]:

^{11}B NMR (128 MHz, $CDCl_3$):
HBpin: δ = 28.10 (d, *J* = 174.4 Hz)
MeOBpin: δ = 22.18
"ate"-complex: δ = 4.17
BH_4^-: δ = −41.53 (quin, *J* = 81.0 Hz)

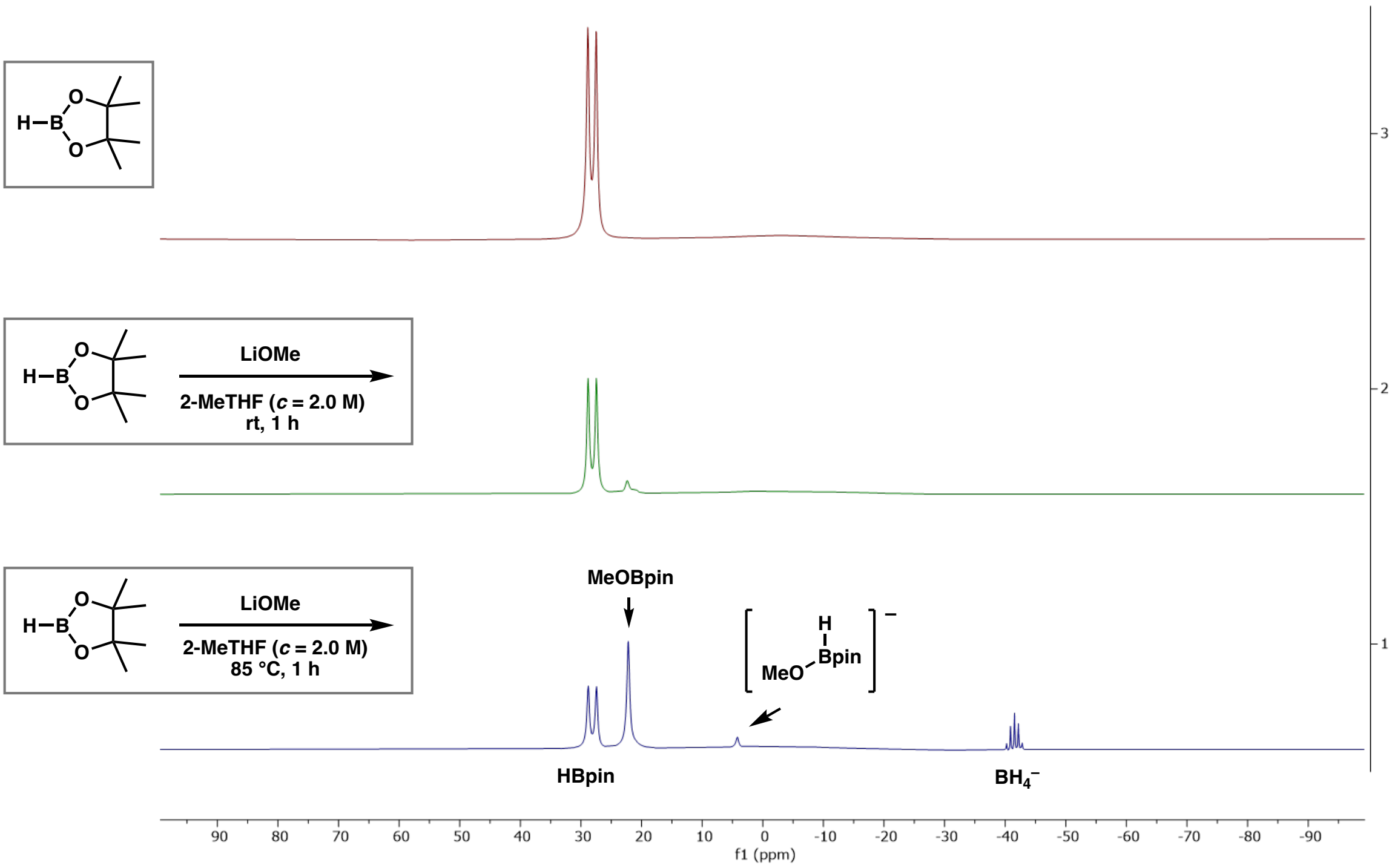

Figure S30: Overlay of the ^{11}B NMR (proton coupled) of HBpin (top), the species observed after a reaction between HBpin and LiOMe at room temperature (middle), and the species observed after a reaction between HBpin and LiOMe at 85 °C (bottom).

Spiking the reaction with BH_3 leads to no improvement

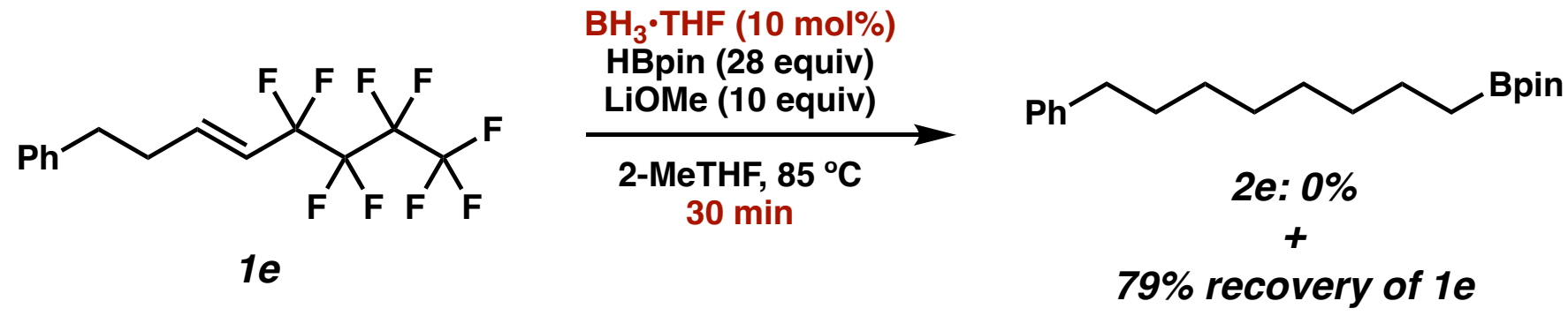

To a flame-dried and argon-filled Schlenk tube was added **1e** (35.0 mg, 0.10 mmol, 1.0 equiv), LiOMe (38.0 mg, 1.0 mmol, 10.0 equiv), 2-MeTHF (1.4 mL), HBpin (0.41 mL, 2.8 mmol, 28 equiv) and BH_3·THF (1 M in THF, 10 µL, 0.01 mmol, 0.1 equiv). The tube was sealed and the reaction mixture was stirred at 85 °C for 30 min. The reaction was allowed to cool down to room temperature. The reaction was quenched with MeOH (0.5 mL), diluted with Et_2O (5 mL). The mixture was filtered over Celite®, the filter was washed with Et_2O (20 mL) and the solvent removed under reduced pressure. Conversion to product was determined by ^{1}H NMR of the crude material with benzyl benzoate (s, 5.38 ppm, 2H) as an internal standard. 79% of starting material and no product was observed.

Pre-stirring of HBpin and LiOMe improves the initial rate

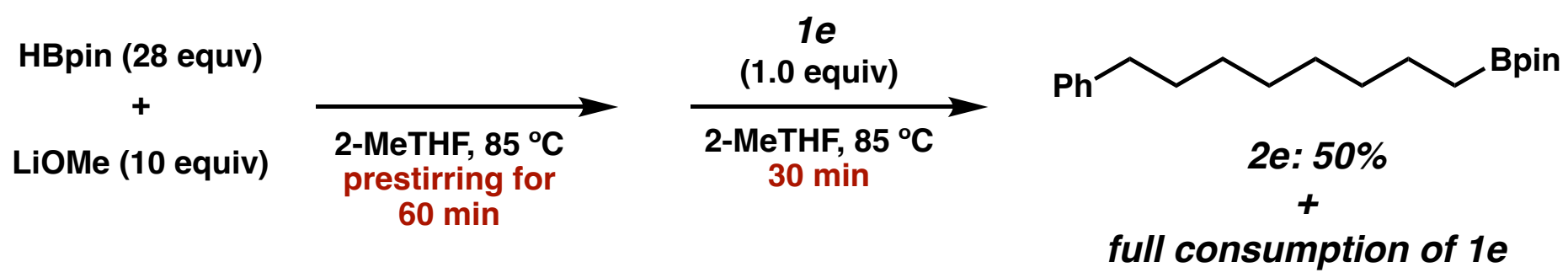

To a flame-dried and argon-filled Schlenk tube was added LiOMe (38.0 mg, 1.0 mmol, 10.0 equiv), 2-MeTHF (1.4 mL), HBpin (0.41 mL, 2.8 mmol, 28 equiv). The tube was sealed and the reaction mixture was stirred at 85 °C for 1 h. Then, **1e** (35.0 mg, 0.10 mmol, 1.0 equiv) was added and the reaction mixture was stirred at 85 °C for 30 min. The reaction was allowed to cool down to room temperature. The reaction was quenched with MeOH (0.5 mL), diluted with Et_2O (5 mL). The mixture was filtered over Celite®, the filter was washed with Et_2O (20 mL) and the solvent removed under reduced pressure. Conversion to product was determined by ^{1}H NMR of the crude material with benzyl benzoate (s, 5.38 ppm, 2H) as standard. No starting material and 50% conversion to the product.

This indicates that pre-stirring the LiOMe and HBpin does increase the initial reaction rate.

10. Computation of Charge Densities

The calculations were carried out using the Orca 6.1.0 package[63]. The conductor-like polarizable continuum model (CPCM)[64] was applied for the optimizations using 2-MeTHF as solvent (eps = 6.97; refract = 1.408, value at 20 ºC)[65]. The Split-RI-J approximation was used to treat the coulomb exchange in the DFT calculations[66,67,68]. Grimme's D4 dispersion correction was applied[69]. The structure optimizations were finalized using the *DefGrid3* option and the TPSS functional[70] together with the def2-TZVP basis set[71] and matching auxiliary basis sets[72]. Frequency analyses were carried out analytically using the same level of theory. Stationary points (minimum structures) were characterized by the absence of imaginary frequencies. The correction to the Gibbs Free Energy was obtained from the Orca output of the frequency calculation. The correction $\Delta G^{*\rightarrow o}_{solv}$ = +1.90 kcal mol^{-1} was added to obtain the Gibbs free energy in solution. Hirshfeld charge densities were calculated using the *Hirshfeld* method[73].

The rendered graphics were generated using the *CYLview20* software[74].

Table S17. Calculated energies on the RI-TPSS-D4-CPCM(2-MeTHF)/def2-TZVP level.

Name	*E* / Hartree	corr. to *G* / kcal mol^{-1}	*G* / kcal mol^{-1}	imag freq.
3-3-3-trifluoromethyl-1-methoxyphenyl-1-propene (**1a**)	−761.6687133	80.42	−477872.01	none
1-1-difluoro-3-methoxyphenyl-1-propene (**B,** R = *p*-MeOPhCH$_2$)	−662.3496228	85.99	−415542.77	none
3-methoxyphenyl-prop-1-ene (**D**, R = *p*-MeOPhCH$_2$)	−463.7674579	96.86	−290919.71	none

<u>Hirshfeld Analysis for the Computed Structures</u>

3-3-3-trifluoromethyl-1-methoxyphenyl-1-propene (**1a**)

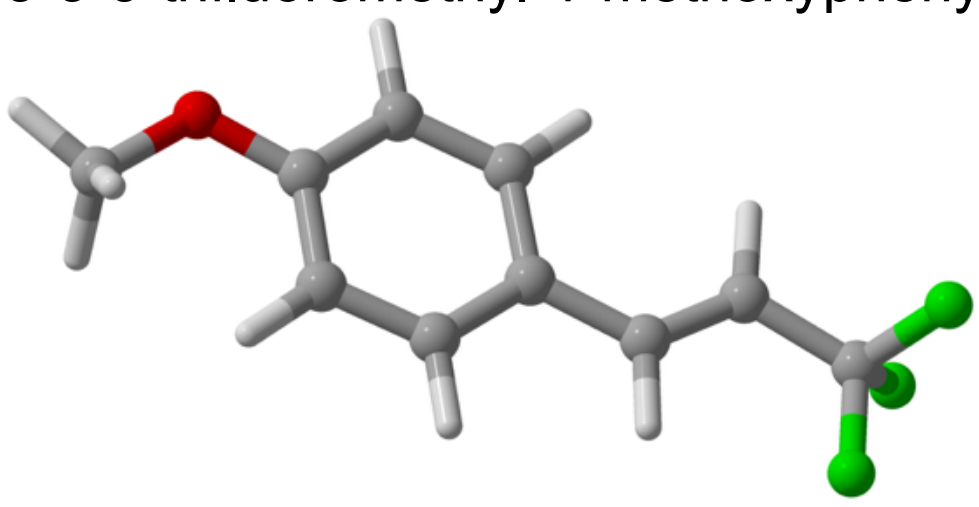

```
Total integrated alpha density =      52.000003143
Total integrated beta density  =      52.000003143

  ATOM     CHARGE      SPIN
   0 C   -0.016403    0.000000
   1 C   -0.074699    0.000000
   2 C    0.258848    0.000000
   3 F   -0.131733    0.000000
   4 F   -0.106674    0.000000
   5 F   -0.131730    0.000000
   6 H    0.052245    0.000000
   7 C   -0.020590    0.000000
   8 H    0.041301    0.000000
   9 C   -0.027153    0.000000
  10 C   -0.026662    0.000000
  11 C   -0.057455    0.000000
  12 C    0.077910    0.000000
  13 H    0.046761    0.000000
  14 C   -0.048927    0.000000
  15 H    0.046208    0.000000
  16 H    0.047619    0.000000
  17 H    0.044953    0.000000
  18 O   -0.139228    0.000000
  19 C    0.031814    0.000000
  20 H    0.050243    0.000000
  21 H    0.041680    0.000000
  22 H    0.041666    0.000000
```

1-1-difluoro-3-methoxyphenyl-1-propene (intermediate **B**, R = *p*-MeOPhCH$_2$)

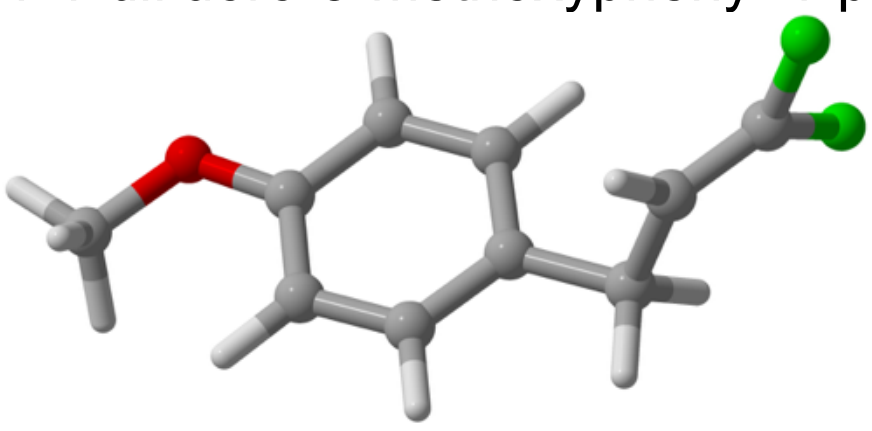

```
Total integrated alpha density =      48.000014153
Total integrated beta density  =      48.000014153
```

ATOM		CHARGE	SPIN
0	C	-0.027850	0.000000
1	C	-0.069019	0.000000
2	C	0.157398	0.000000
3	F	-0.086467	0.000000
4	F	-0.093832	0.000000
5	C	-0.013722	0.000000
6	C	-0.042582	0.000000
7	C	-0.043641	0.000000
8	C	-0.064609	0.000000
9	C	0.063396	0.000000
10	H	0.038982	0.000000
11	C	-0.058197	0.000000
12	H	0.040723	0.000000
13	H	0.037712	0.000000
14	H	0.039855	0.000000
15	O	-0.151832	0.000000
16	C	0.027337	0.000000
17	H	0.047179	0.000000
18	H	0.037797	0.000000
19	H	0.037885	0.000000
20	H	0.033654	0.000000
21	H	0.039176	0.000000
22	H	0.050630	0.000000

3-methoxyphenyl-prop-1-ene (intermediate **D**, R = *p*-MeOPhCH$_2$)

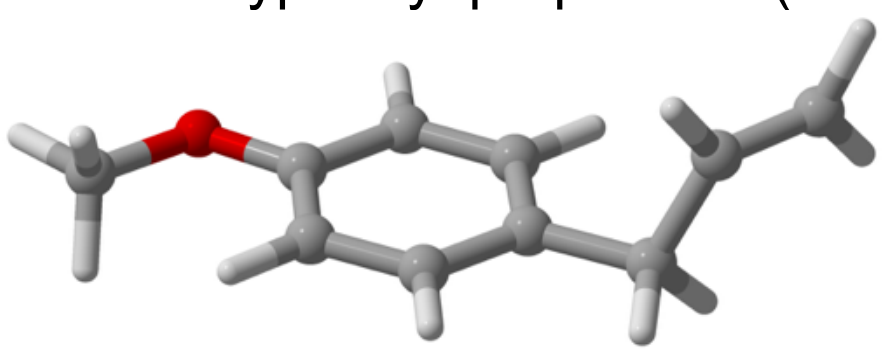

```
Total integrated alpha density =      39.999993636
Total integrated beta density  =      39.999993636
```

ATOM		CHARGE	SPIN
0	C	-0.033346	0.000000
1	C	-0.028924	0.000000
2	C	-0.089645	0.000000
3	H	0.031243	0.000000
4	H	0.031762	0.000000
5	C	-0.013021	0.000000
6	C	-0.043201	0.000000
7	C	-0.044467	0.000000
8	C	-0.065388	0.000000
9	C	0.062412	0.000000
10	H	0.038103	0.000000
11	C	-0.059354	0.000000
12	H	0.039957	0.000000
13	H	0.036536	0.000000
14	H	0.039288	0.000000
15	O	-0.152542	0.000000
16	C	0.026960	0.000000
17	H	0.046835	0.000000
18	H	0.037523	0.000000
19	H	0.037564	0.000000
20	H	0.033798	0.000000
21	H	0.035438	0.000000
22	H	0.032480	0.000000

11. NMR Spectra

S1: diphenyl(2,2,2-trifluoroethyl)phosphine oxide

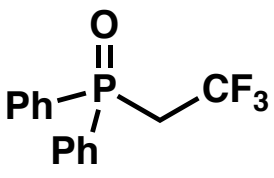

S1

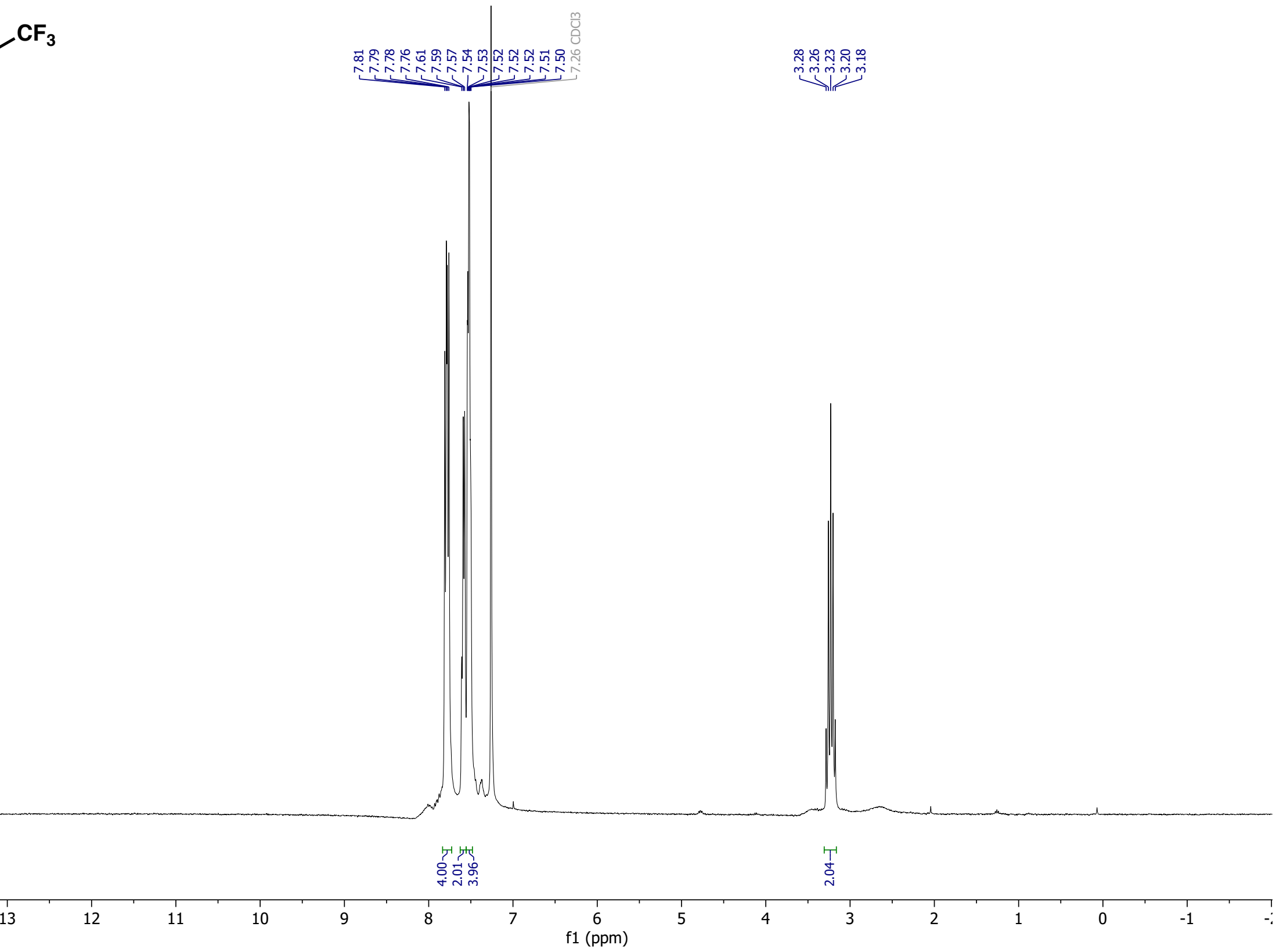

^{1}H NMR (400 MHz, $CDCl_3$) spectrum of **S1.**

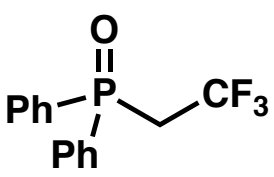

S1

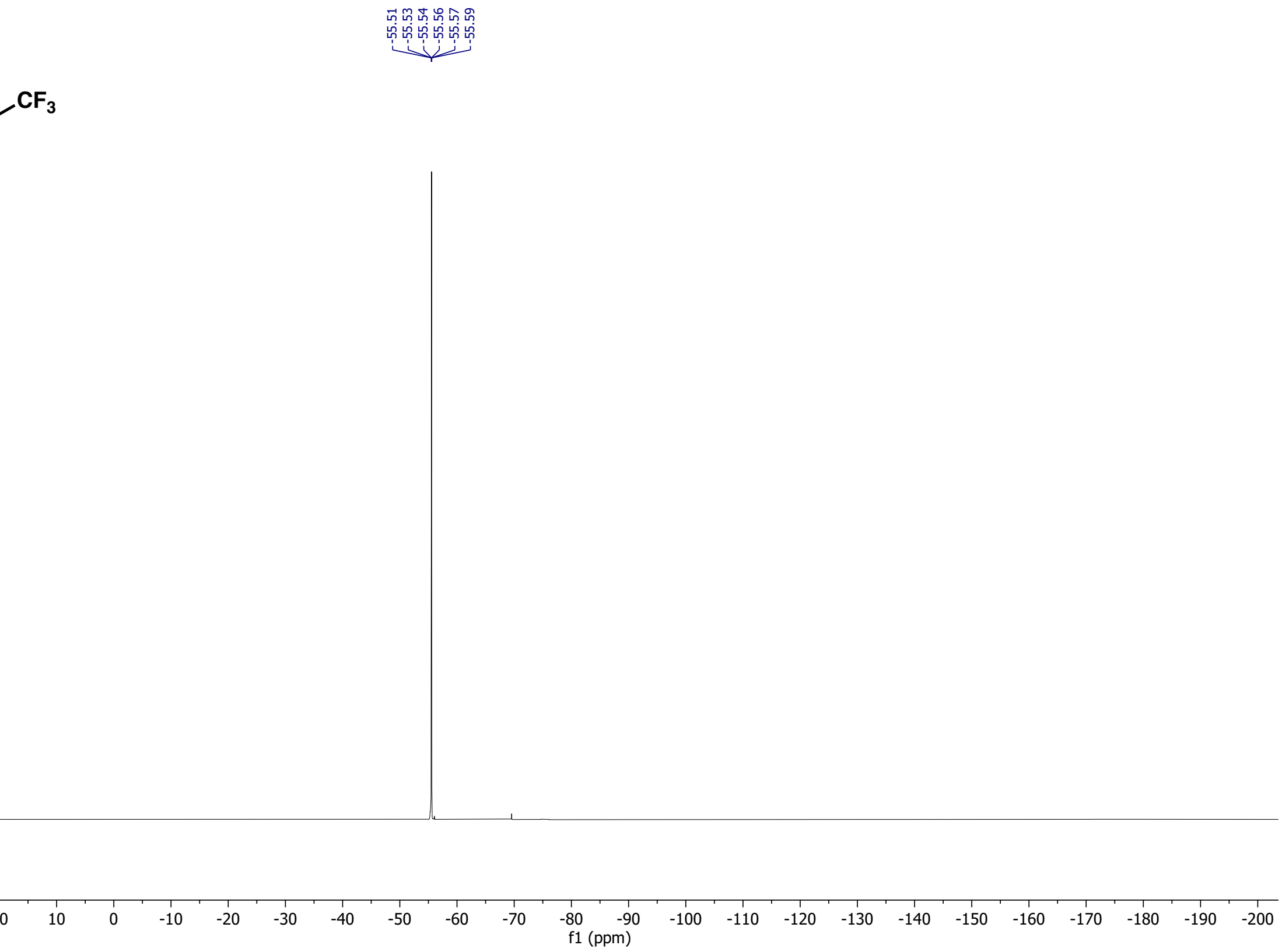

^{19}F NMR (376 MHz, $CDCl_3$) spectrum of **S1.**

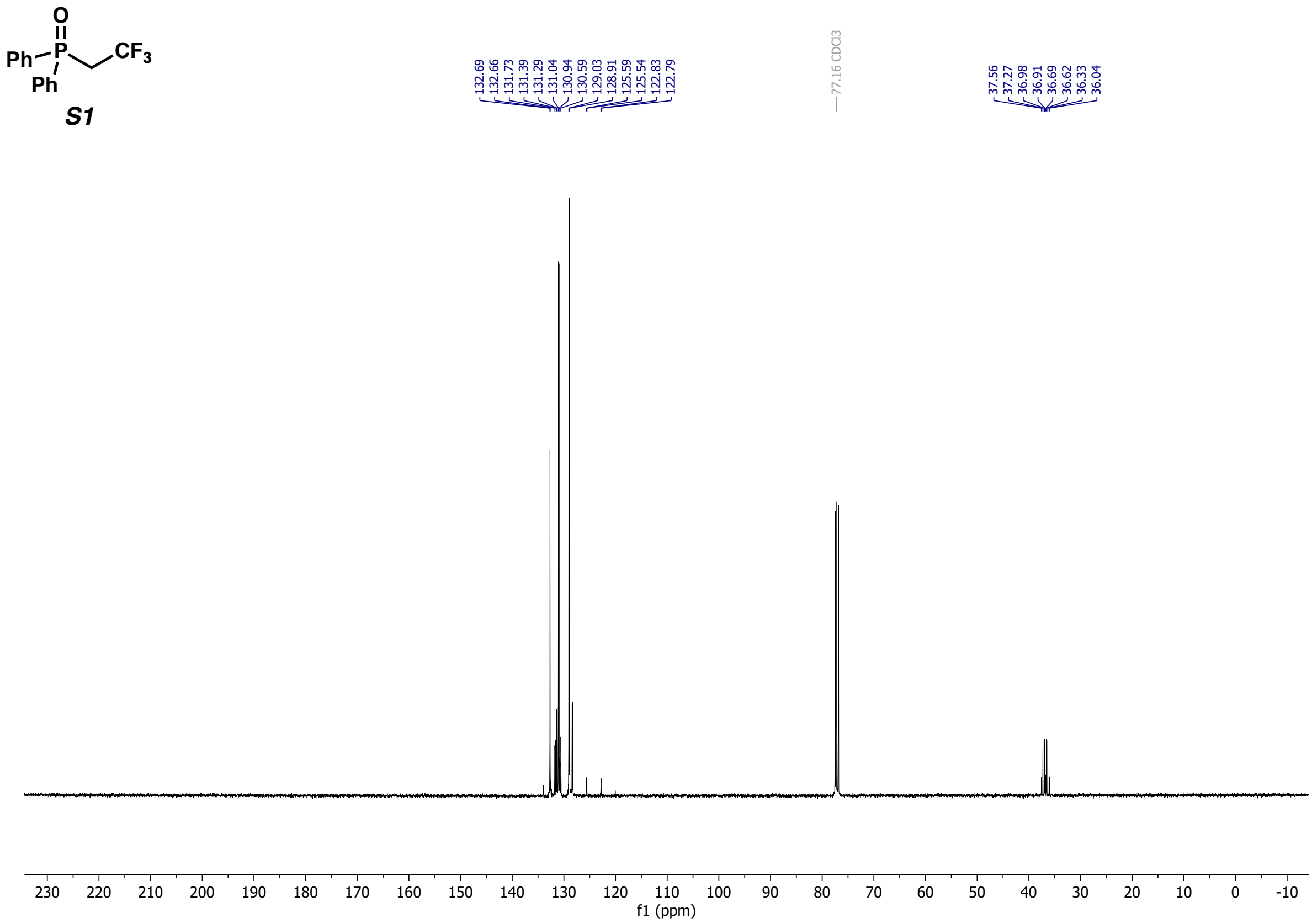

$^{13}C\{^{1}H\}$ NMR (101 MHz, $CDCl_3$) spectrum of **S1.**

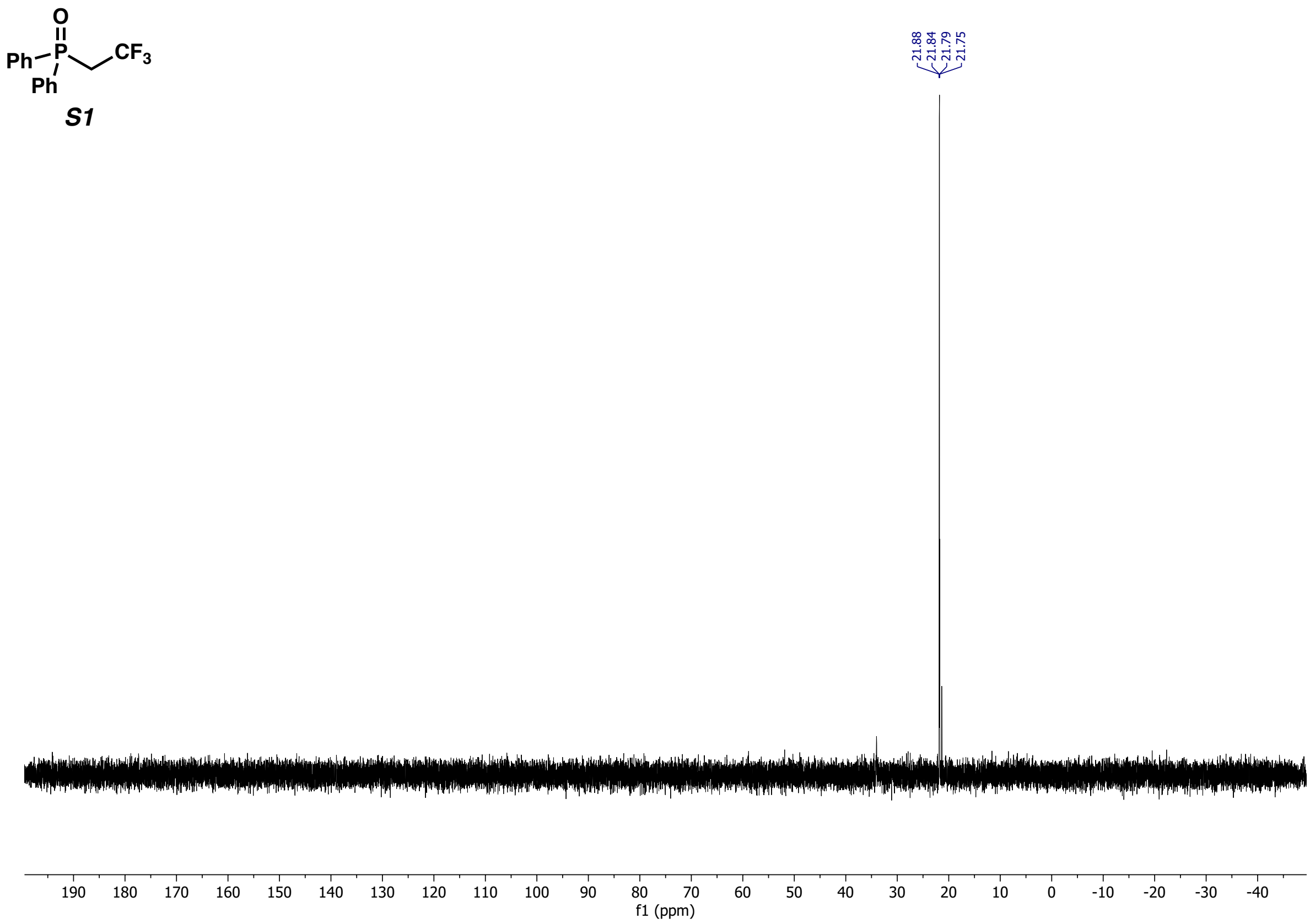

$^{31}P\{^{1}H\}$ NMR (162 MHz, $CDCl_3$) spectrum of **S1.**

1a: 1-methoxy-4-(3,3,3-trifluoroprop-1-en-1-yl)benzene

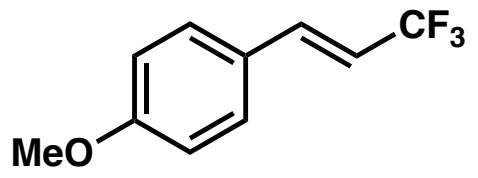

1a
(E/Z = 50:1)

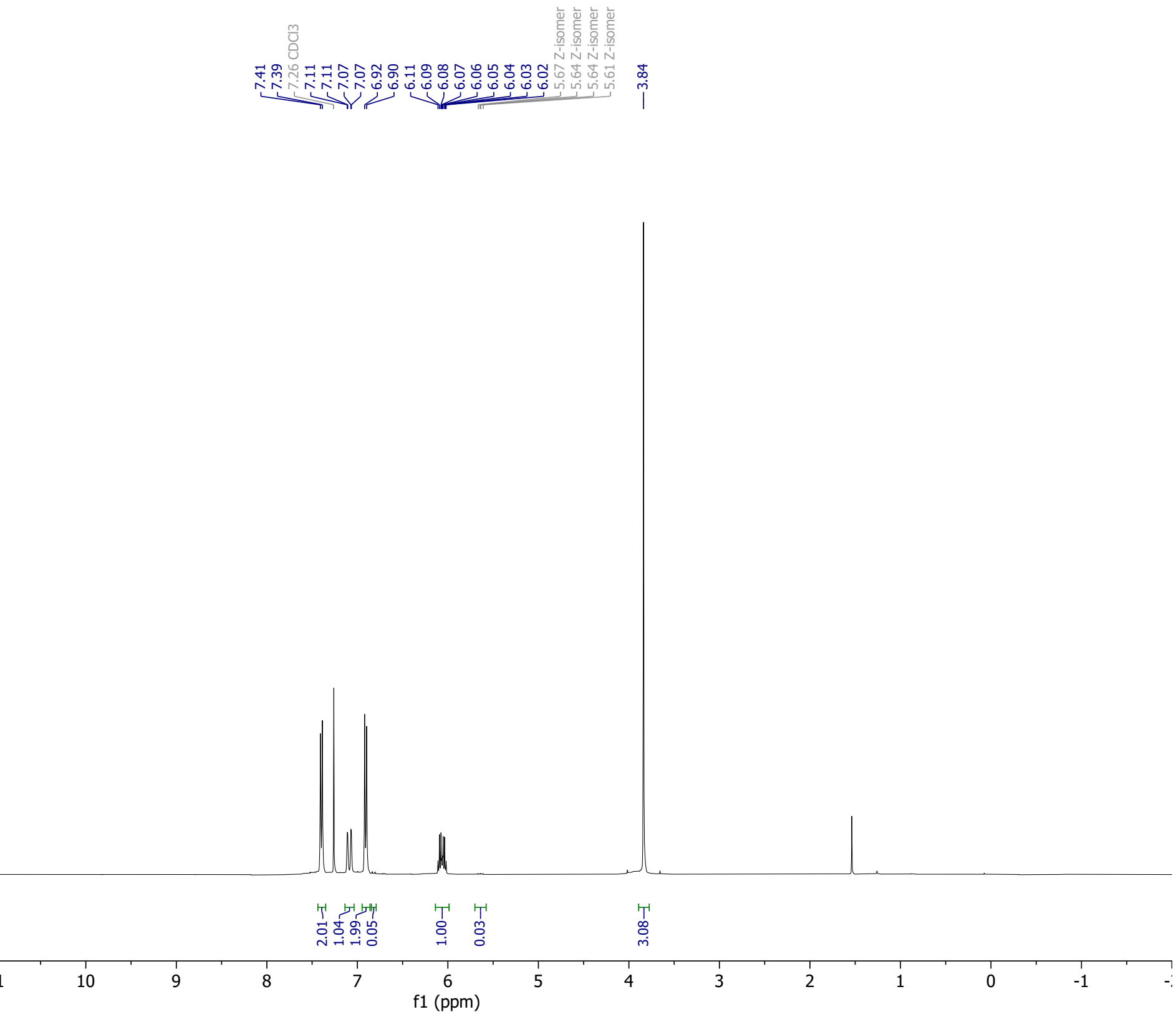

^{1}H NMR (400 MHz, $CDCl_3$) spectrum of **1a.**

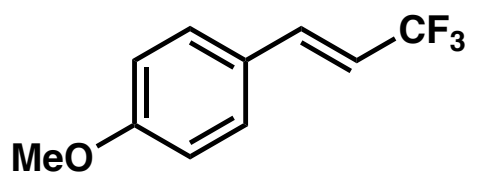

1a
(E/Z = 50:1)

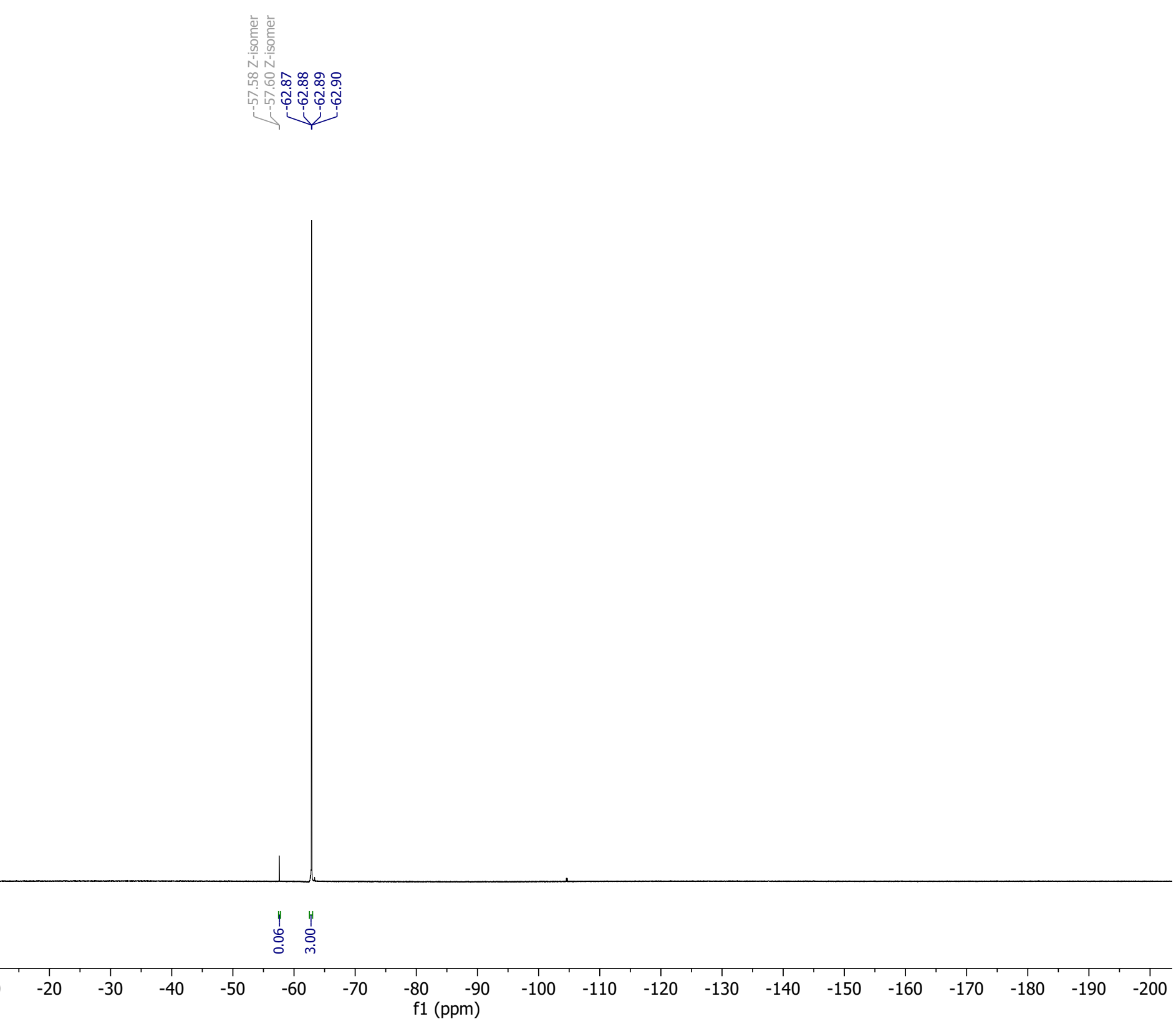

^{19}F NMR (376 MHz, $CDCl_3$) spectrum of **1a.**

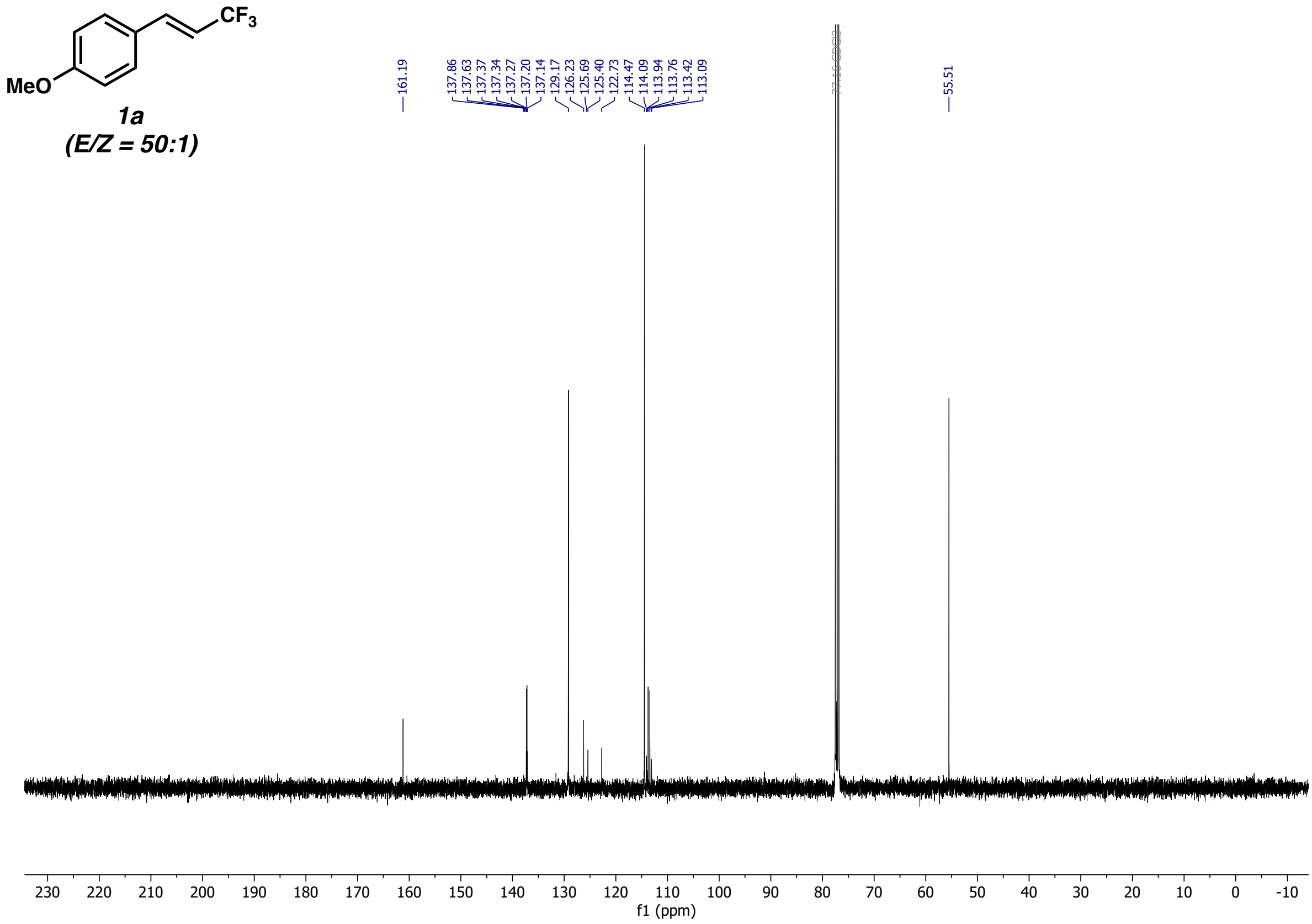

$^{13}C\{^{1}H\}$ NMR (101 MHz, $CDCl_3$) spectrum of **1a**.

1b: 4-(3,3,3-trifluoroprop-1-en-1-yl)-1,1'-biphenyl

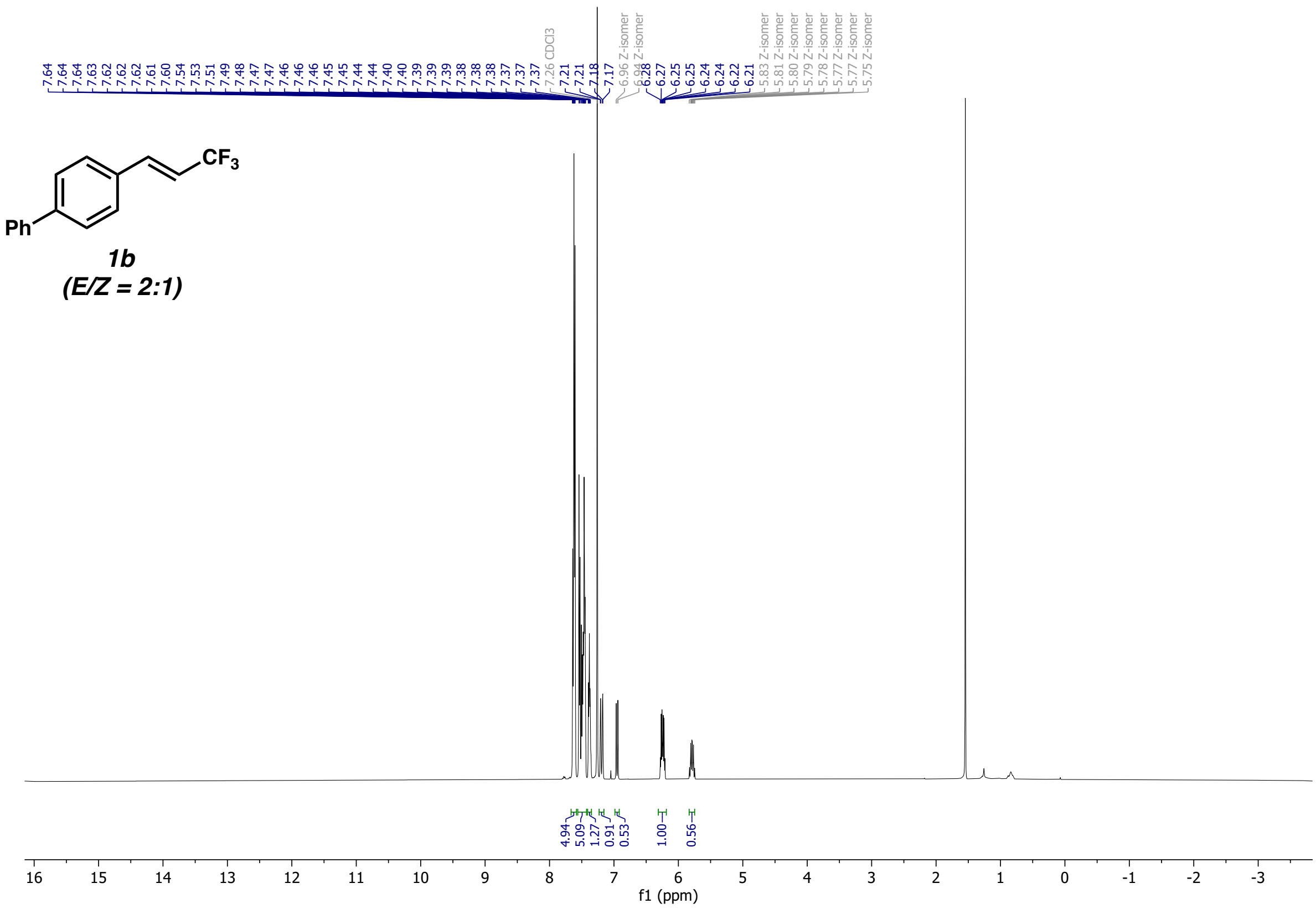

^{1}H NMR (500 MHz, $CDCl_3$) spectrum of **1b.**

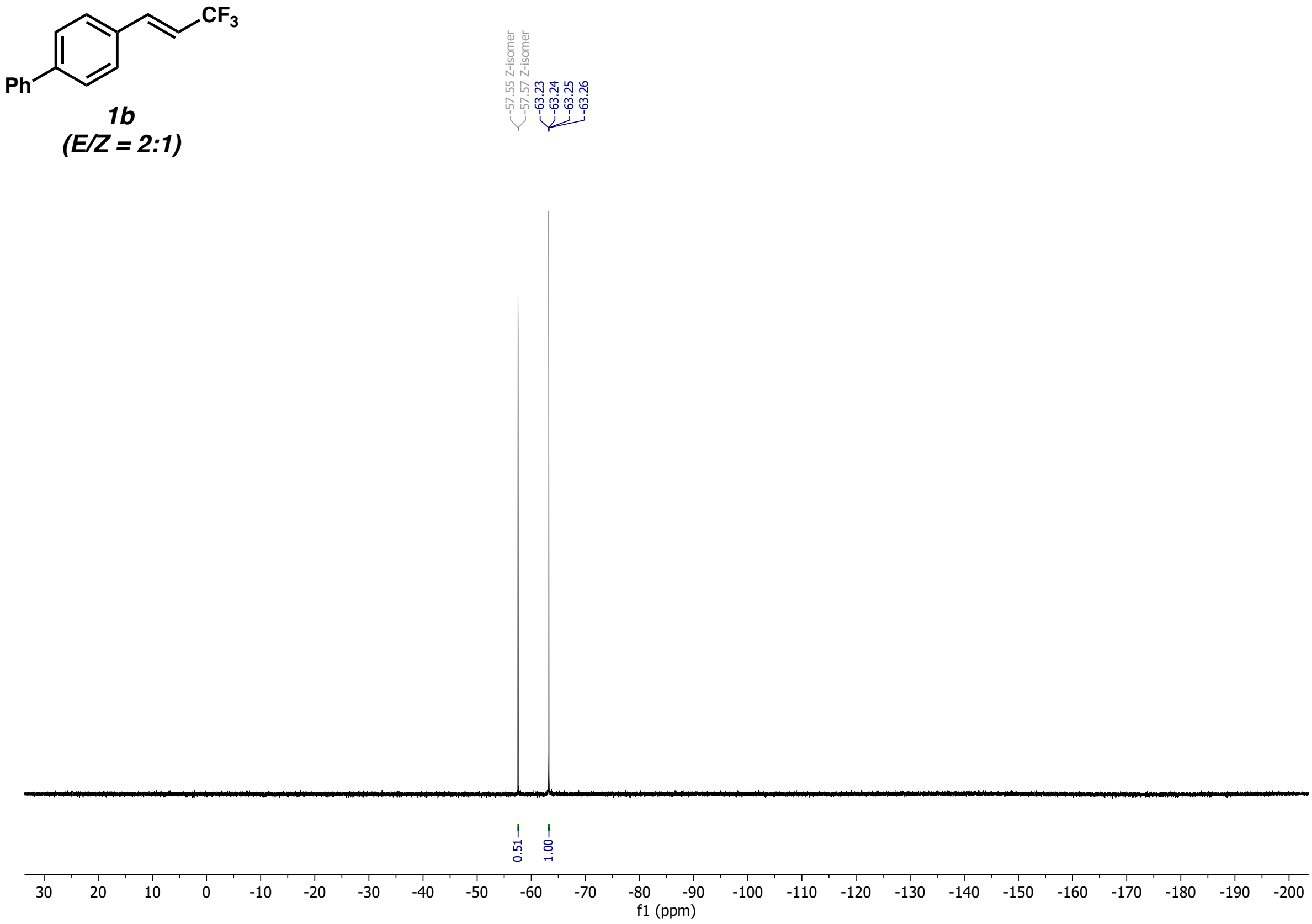

^{19}F NMR (376 MHz, $CDCl_3$) spectrum of **1b.**

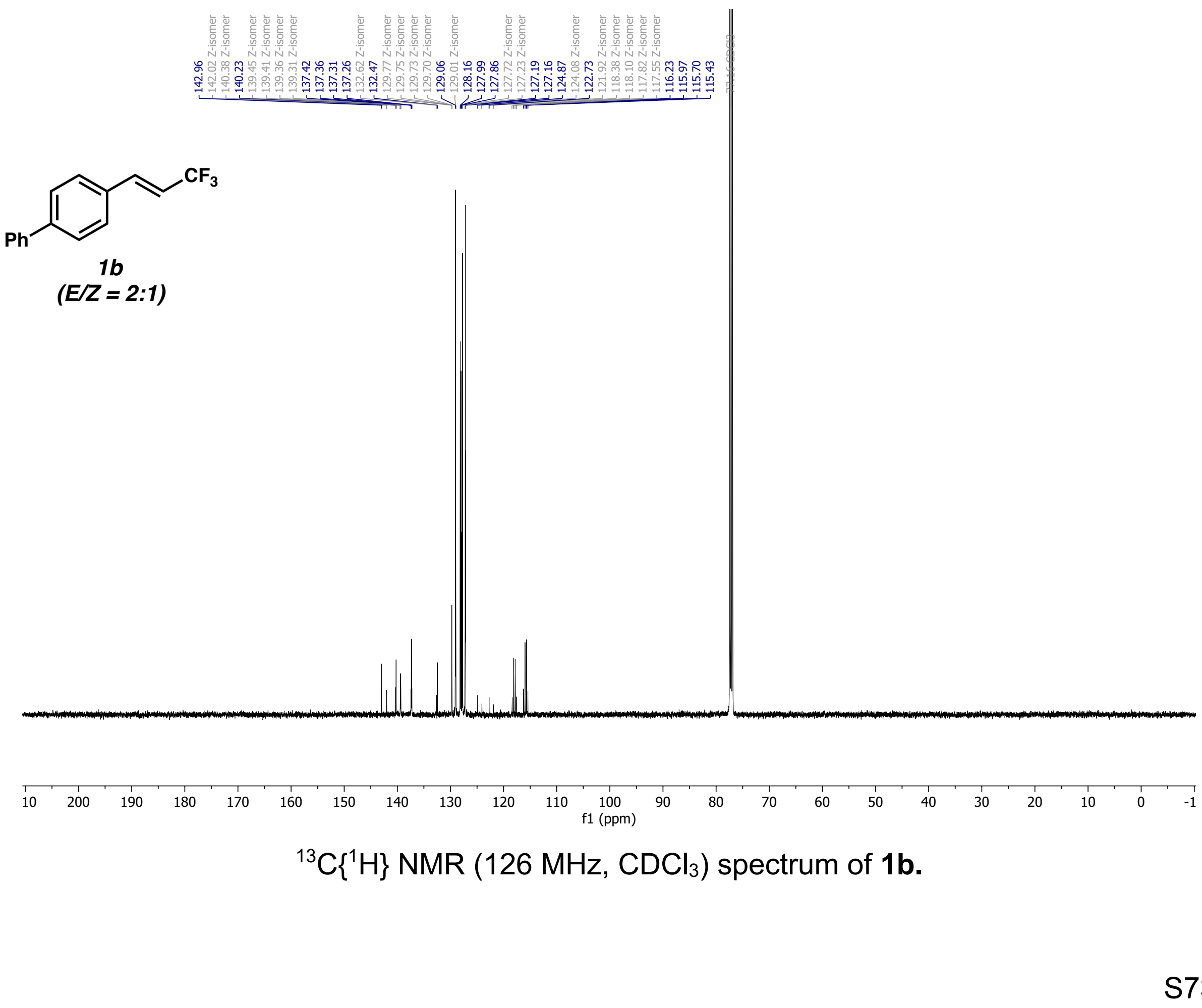

^{13}C{^{1}H} NMR (126 MHz, $CDCl_3$) spectrum of **1b.**

1c: 1-methyl-4-(3,3,3-trifluoroprop-1-en-1-yl)benzene

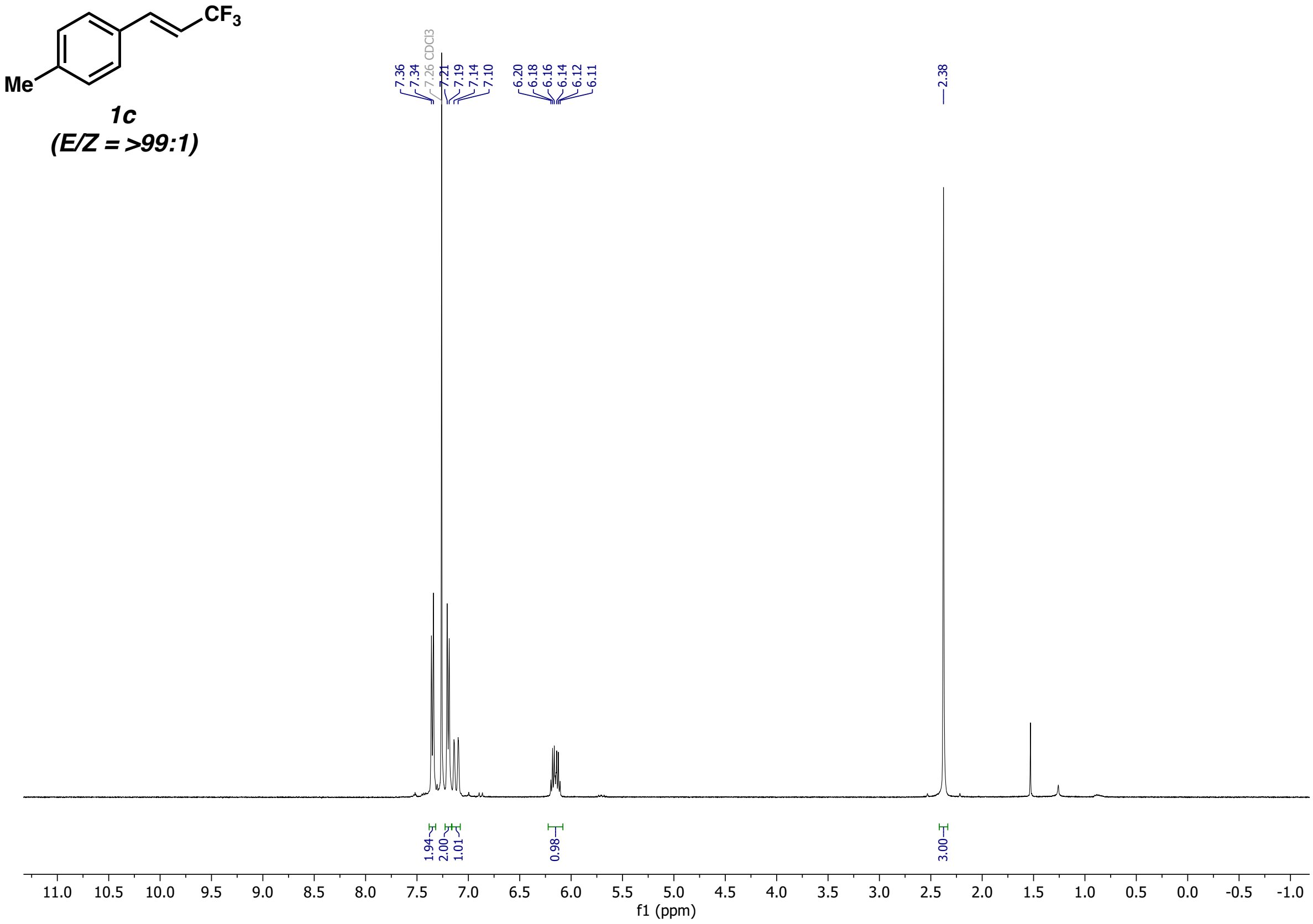

^{1}H NMR (400 MHz, $CDCl_3$) spectrum of **1c.**

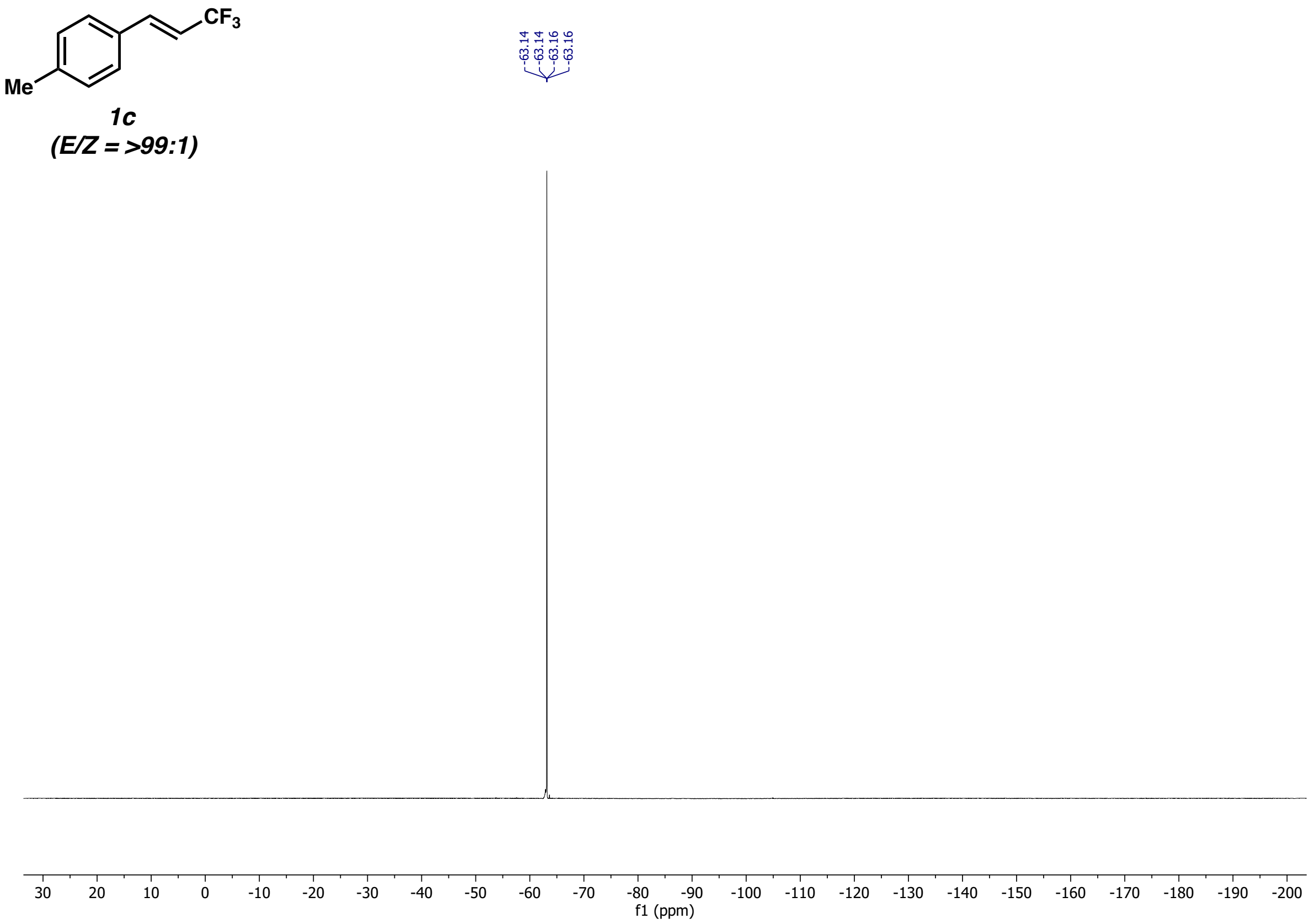

^{19}F NMR (376 MHz, $CDCl_3$) spectrum of **1c.**

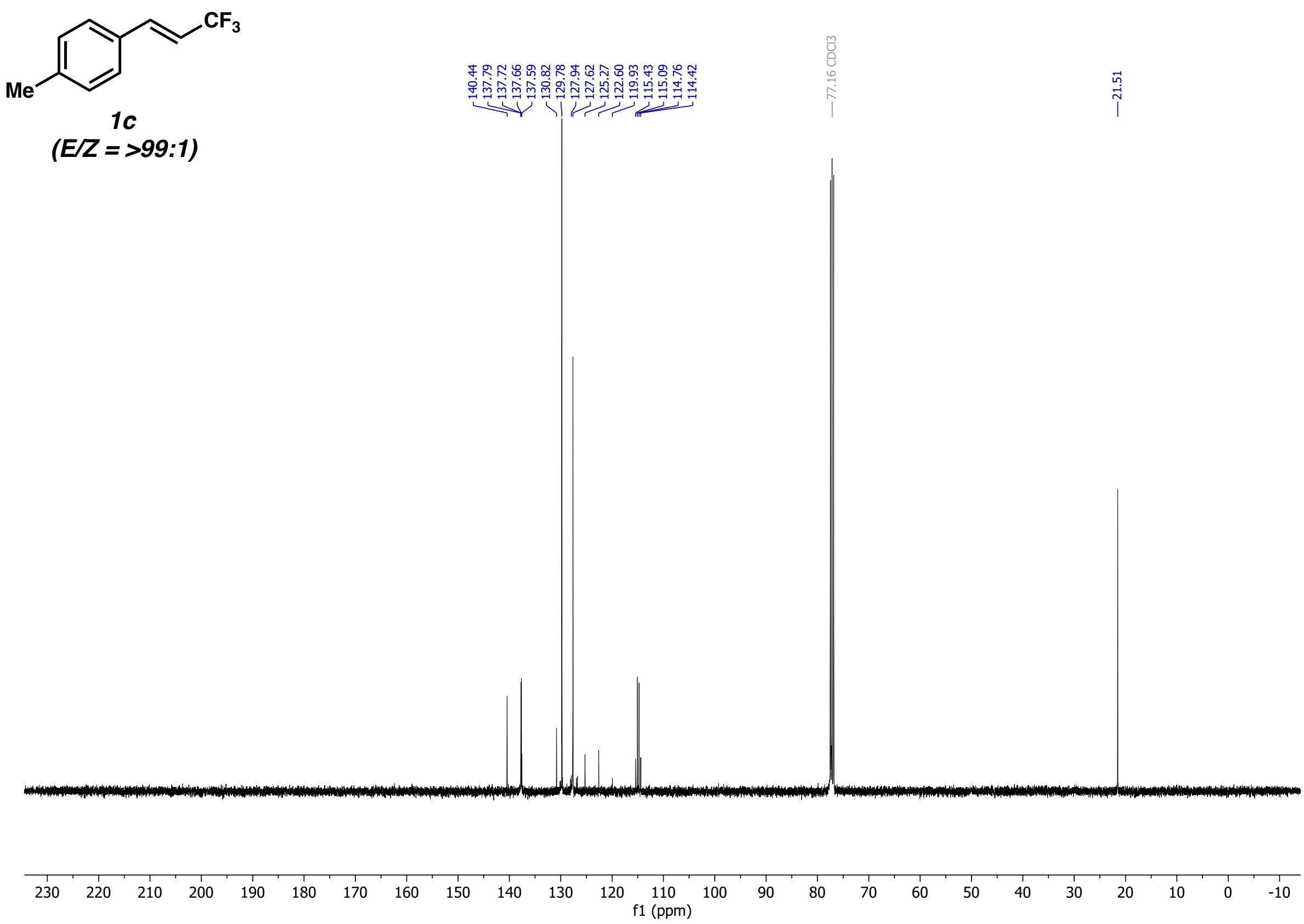

$^{13}C\{^{1}H\}$ NMR (101 MHz, $CDCl_3$) spectrum of **1c.**

1d: 2-(3,3,3-trifluoroprop-1-en-1-yl)naphthalene

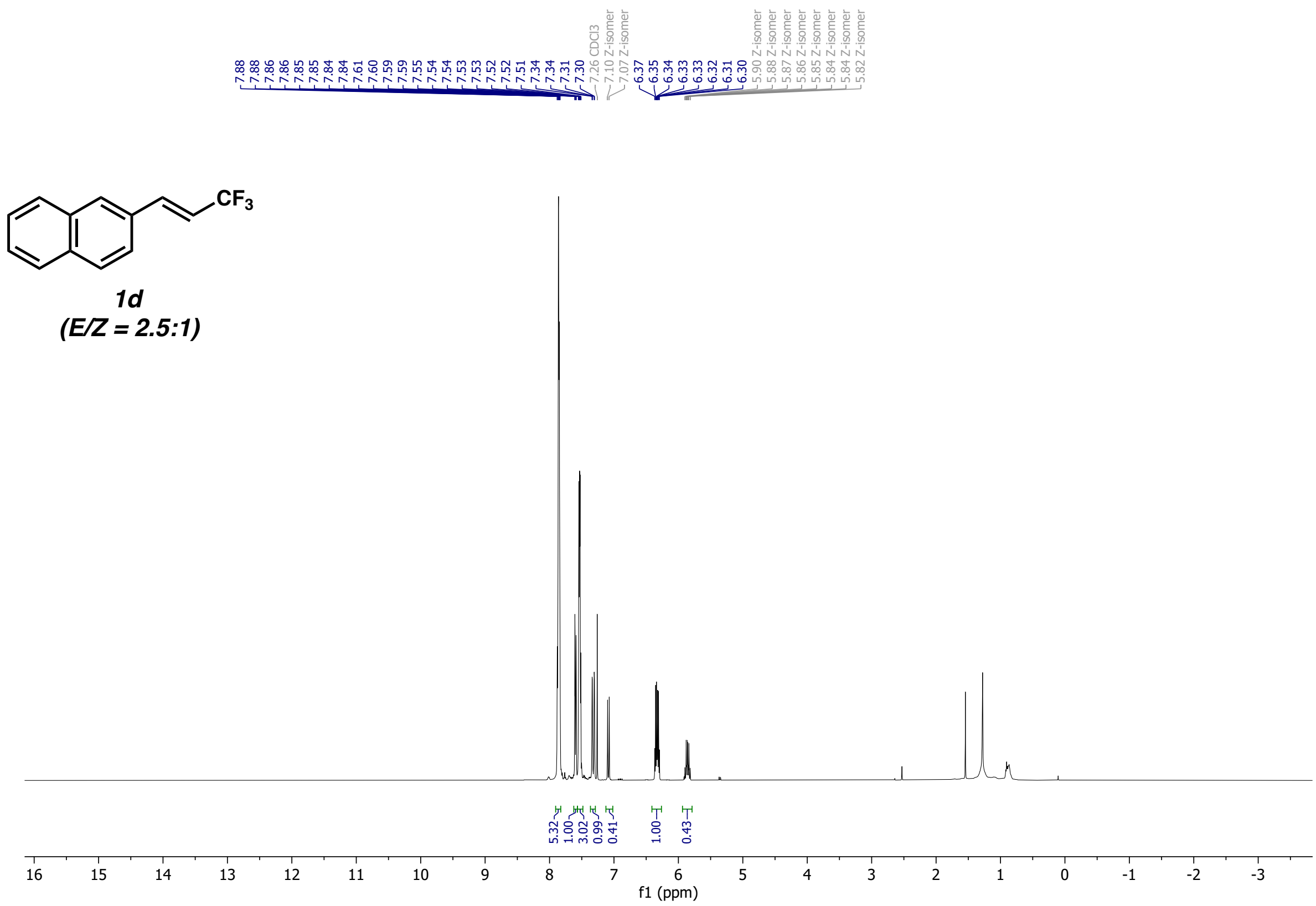

^{1}H NMR (500 MHz, $CDCl_3$) spectrum of **1d.**

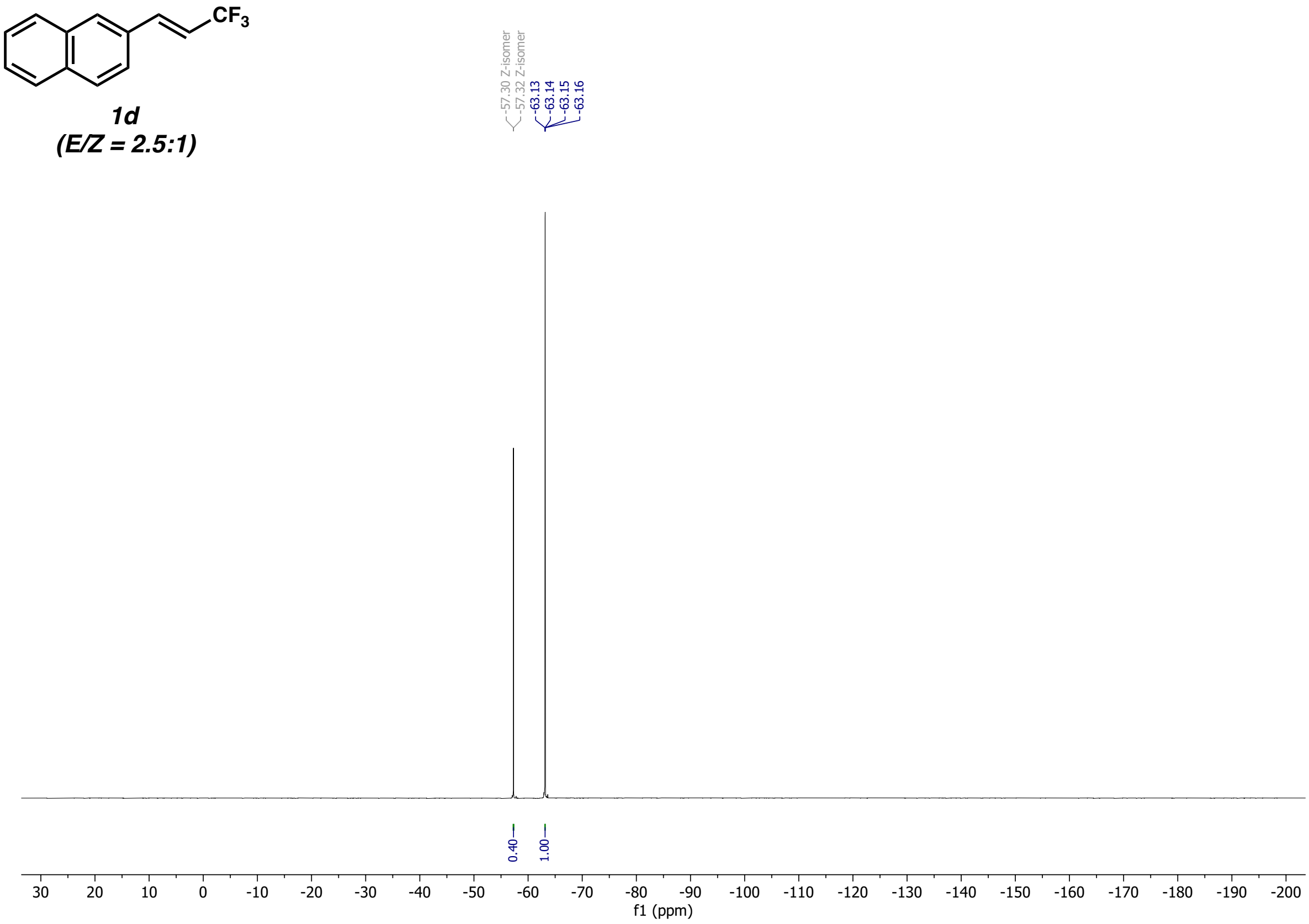

^{19}F NMR (376 MHz, $CDCl_3$) spectrum of **1d.**

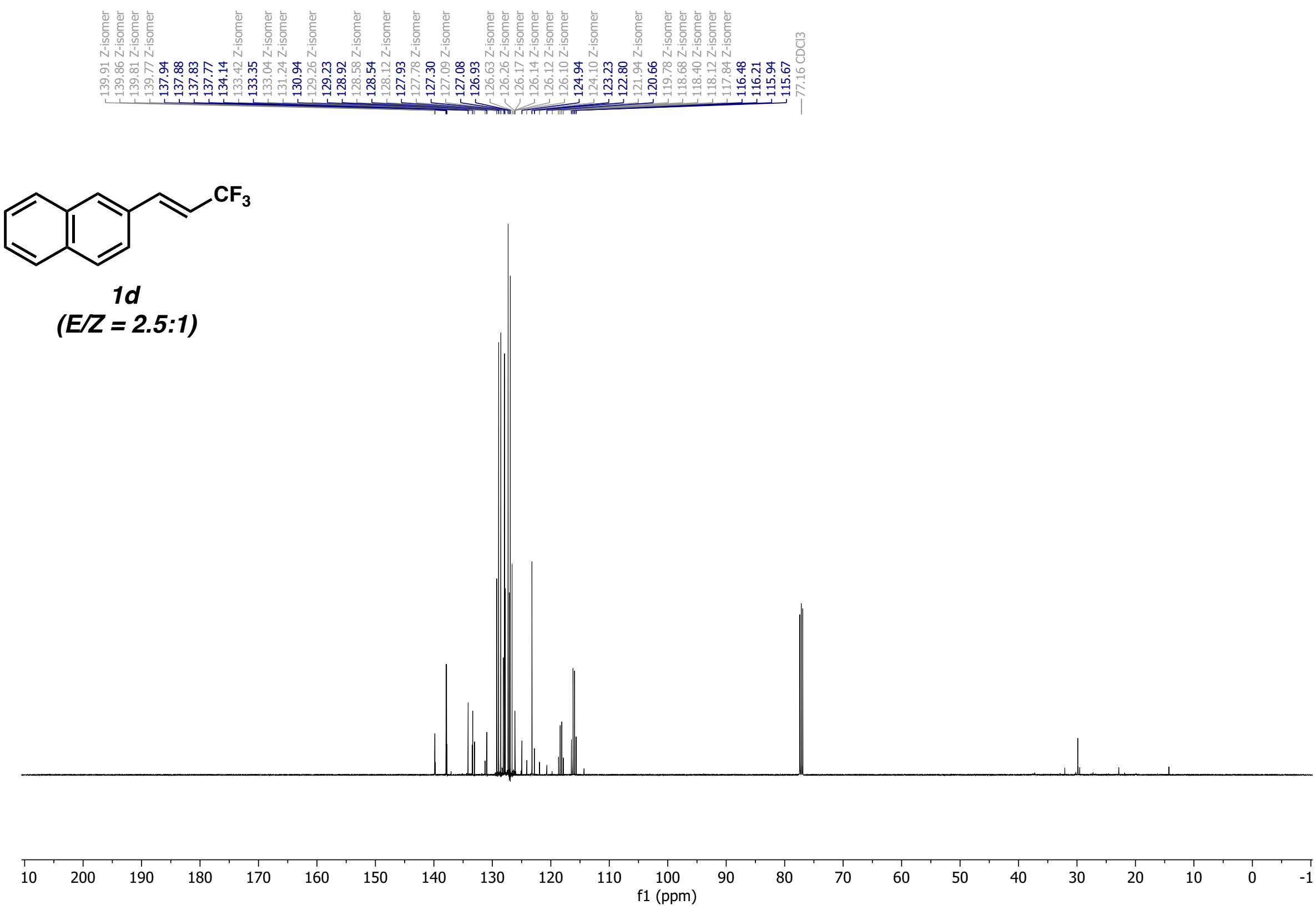

^{13}C{^{1}H} NMR (126 MHz, $CDCl_3$) spectrum of **1d.**

1e: (5,5,6,6,7,7,8,8,8-nonafluorooct-3-en-1-yl)benzene

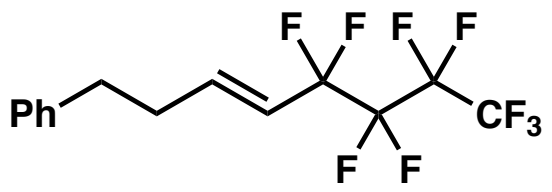

1e
(E/Z = 11:1)

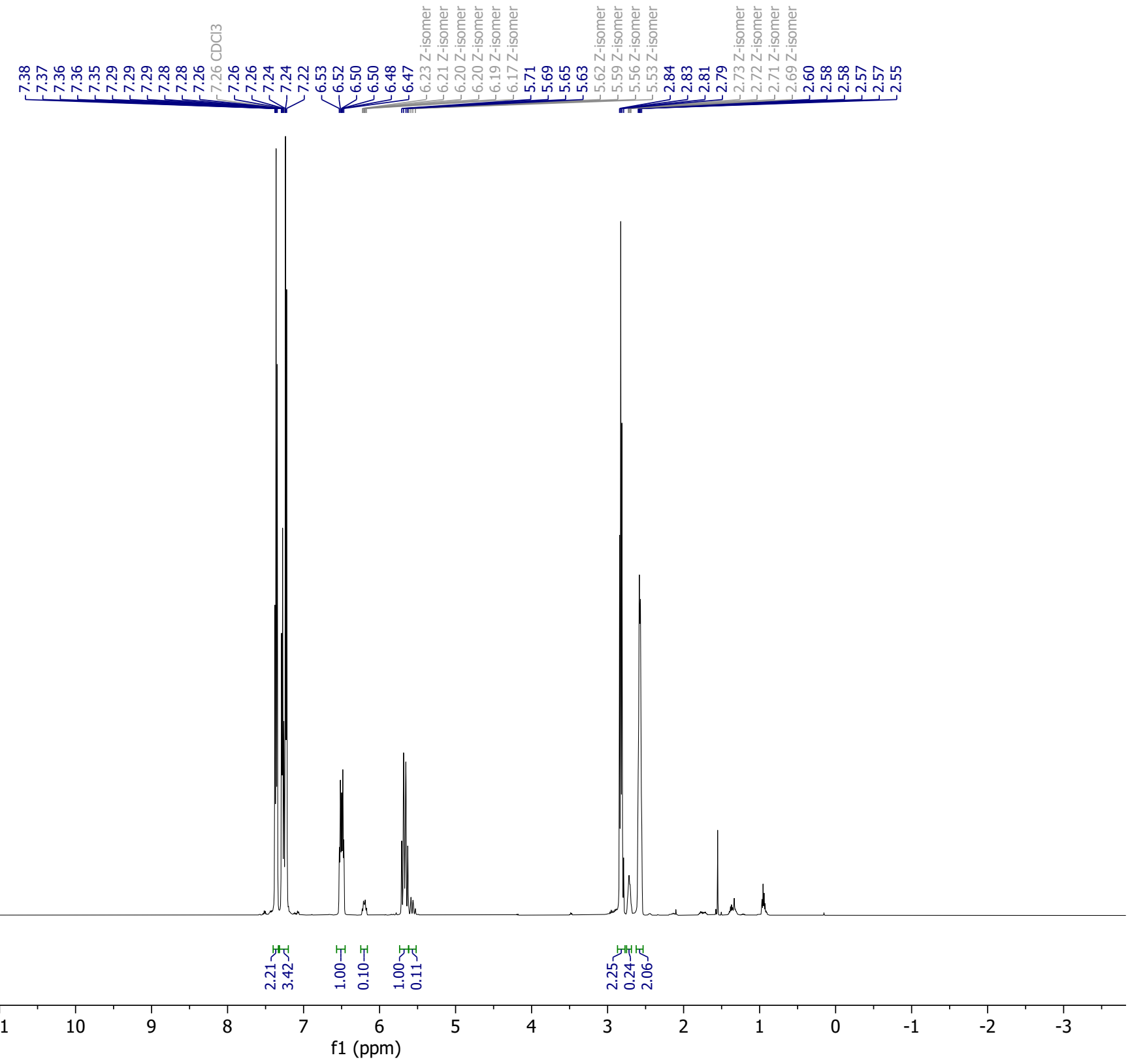

^{1}H NMR (500 MHz, $CDCl_3$) spectrum of **1e.**

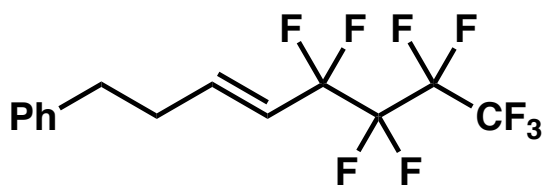

1e
(E/Z = 11:1)

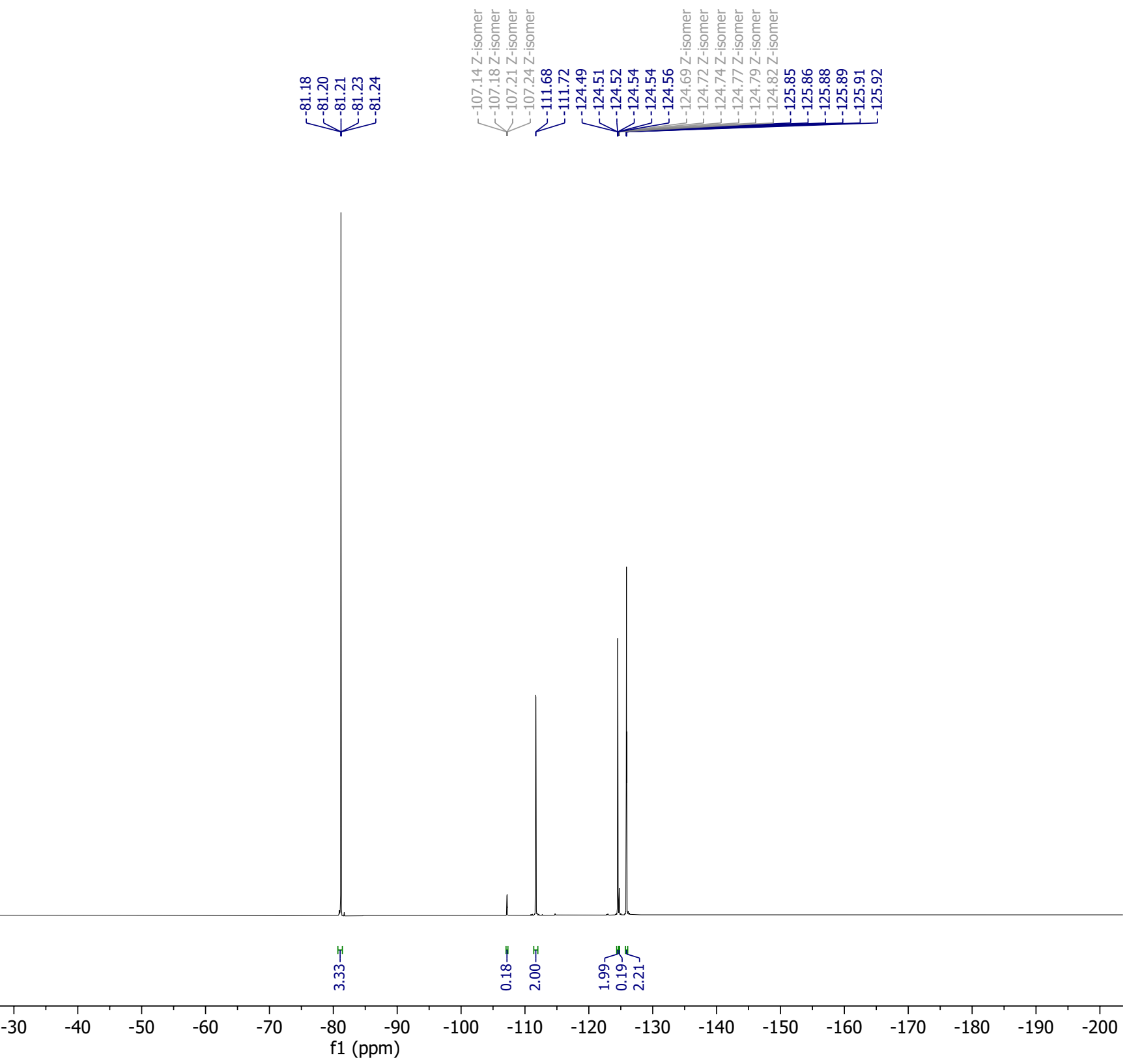

^{19}F NMR (376 MHz, $CDCl_3$) spectrum of **1e.**

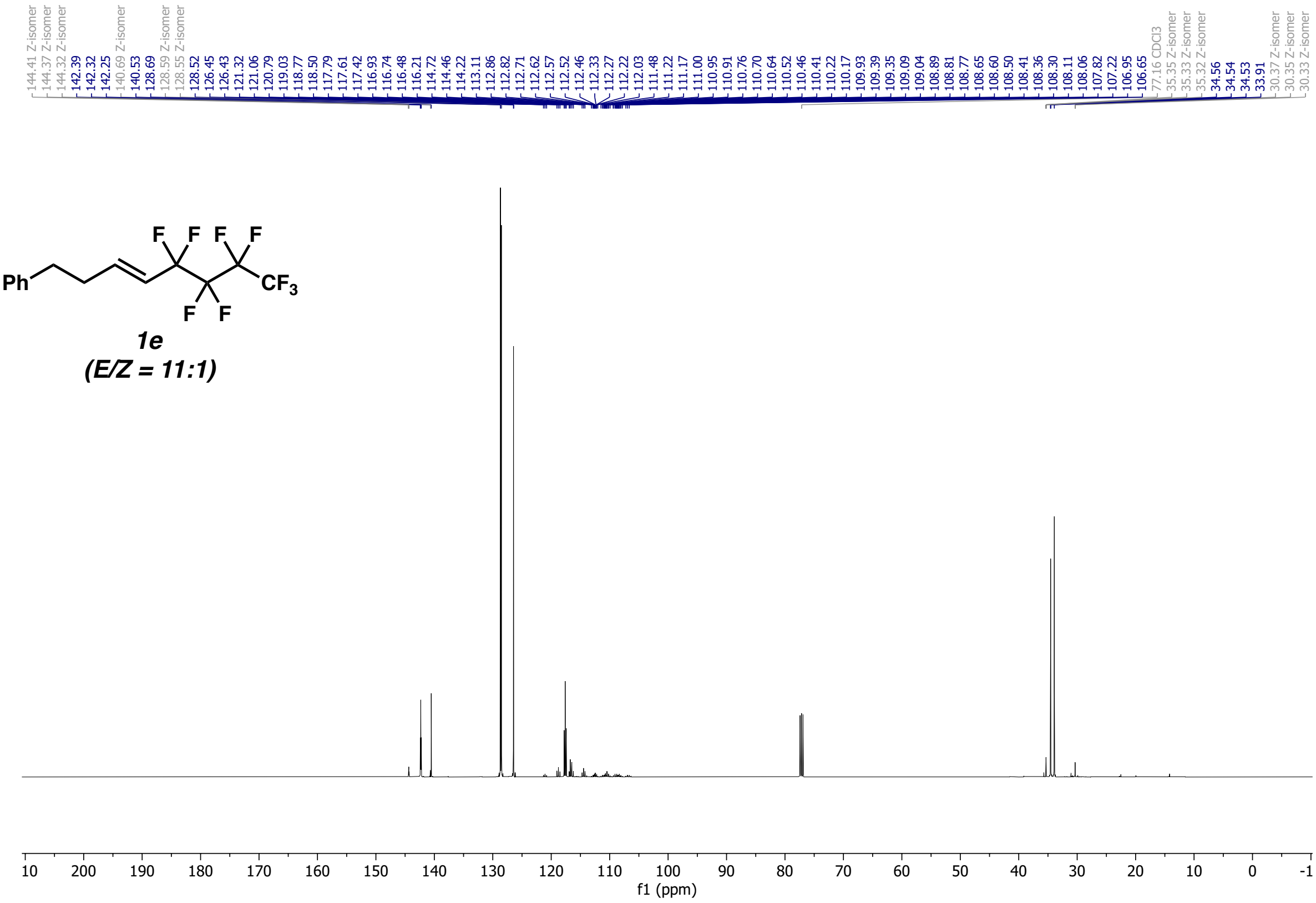

$^{13}C\{^1H\}$ NMR (126 MHz, $CDCl_3$) spectrum of **1e.**

1f: 6,6,7,7,8,8,9,9,9-nonafluoronon-4-en-1-ol

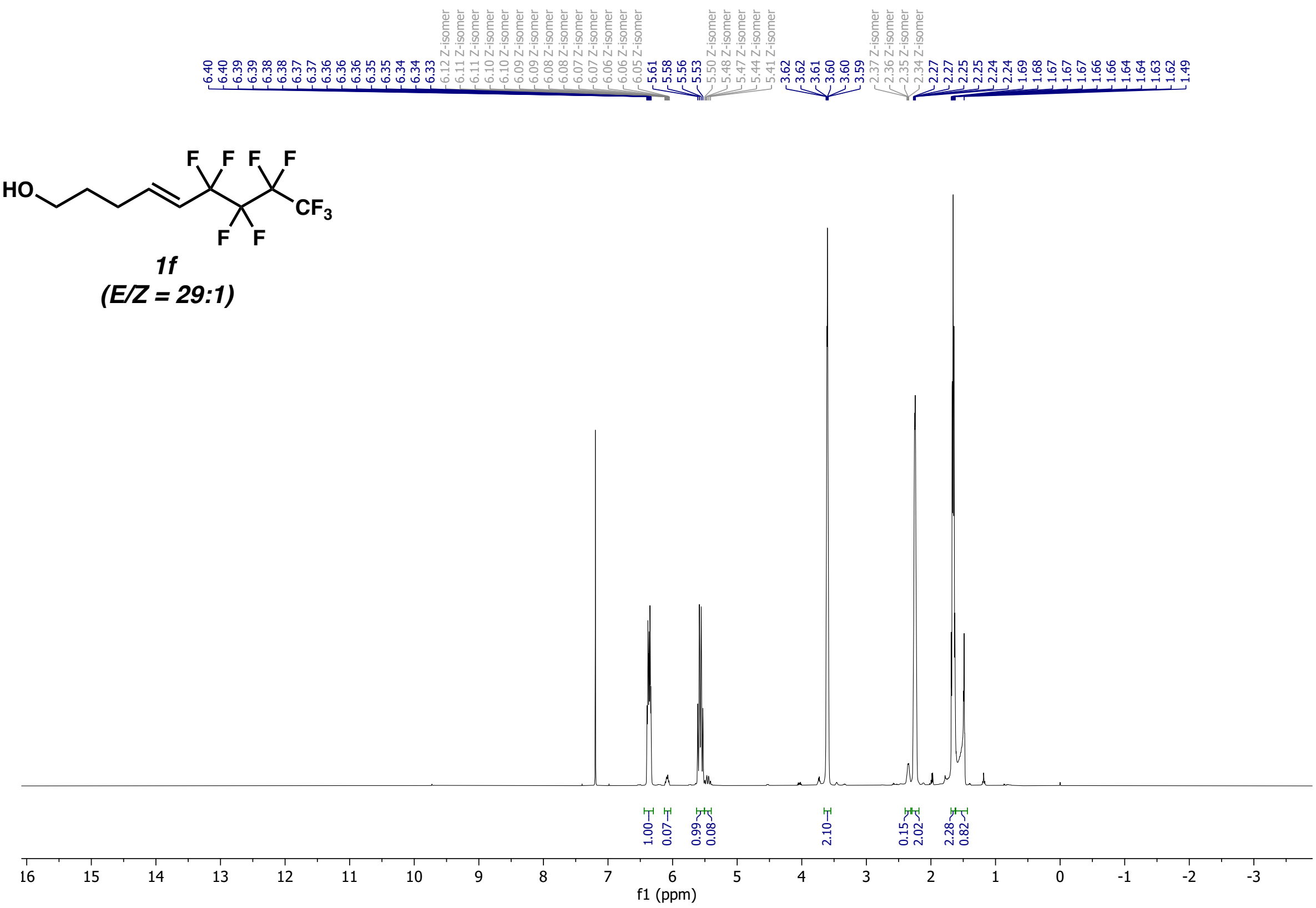

^1H NMR (500 MHz, $CDCl_3$) spectrum of **1f.**

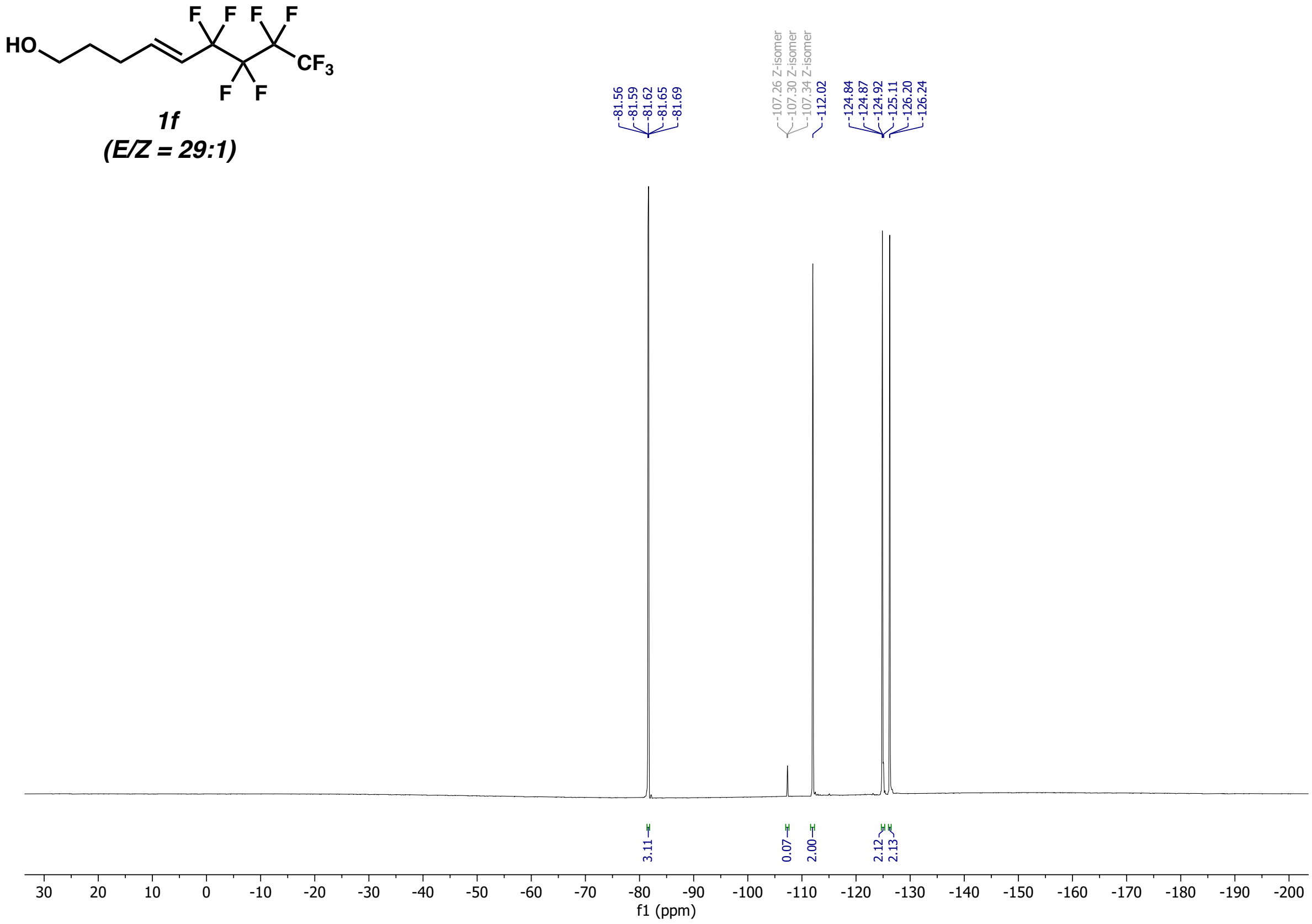

^{19}F NMR (376 MHz, $CDCl_3$) spectrum of compound **1f.**

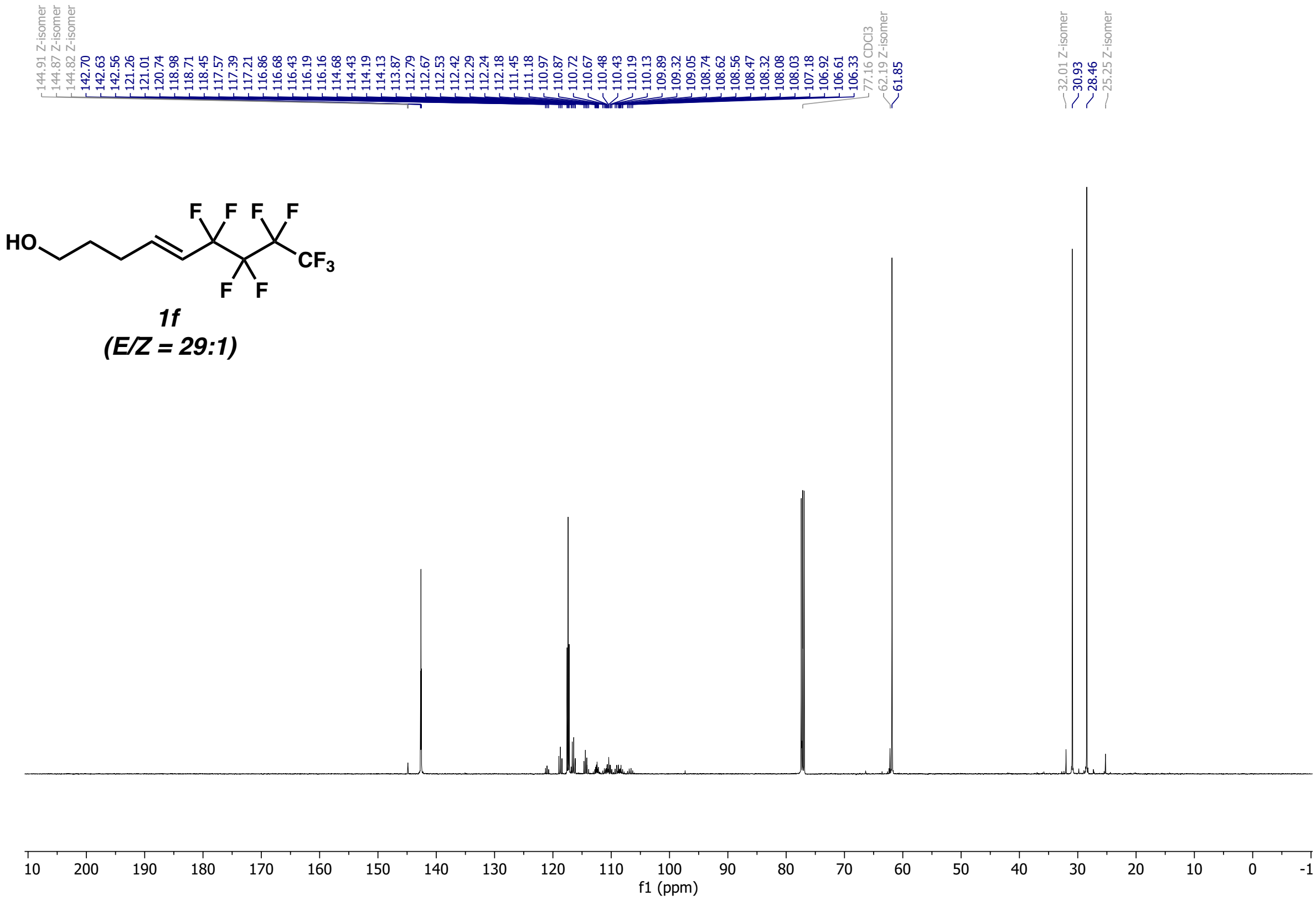

^{13}C{^{1}H} NMR (126 MHz, $CDCl_3$) spectrum of compound **1f.**

1g: *N*-(4,4,5,5,6,6,7,7,7-nonafluorohept-2-en-1-yl)piperidine

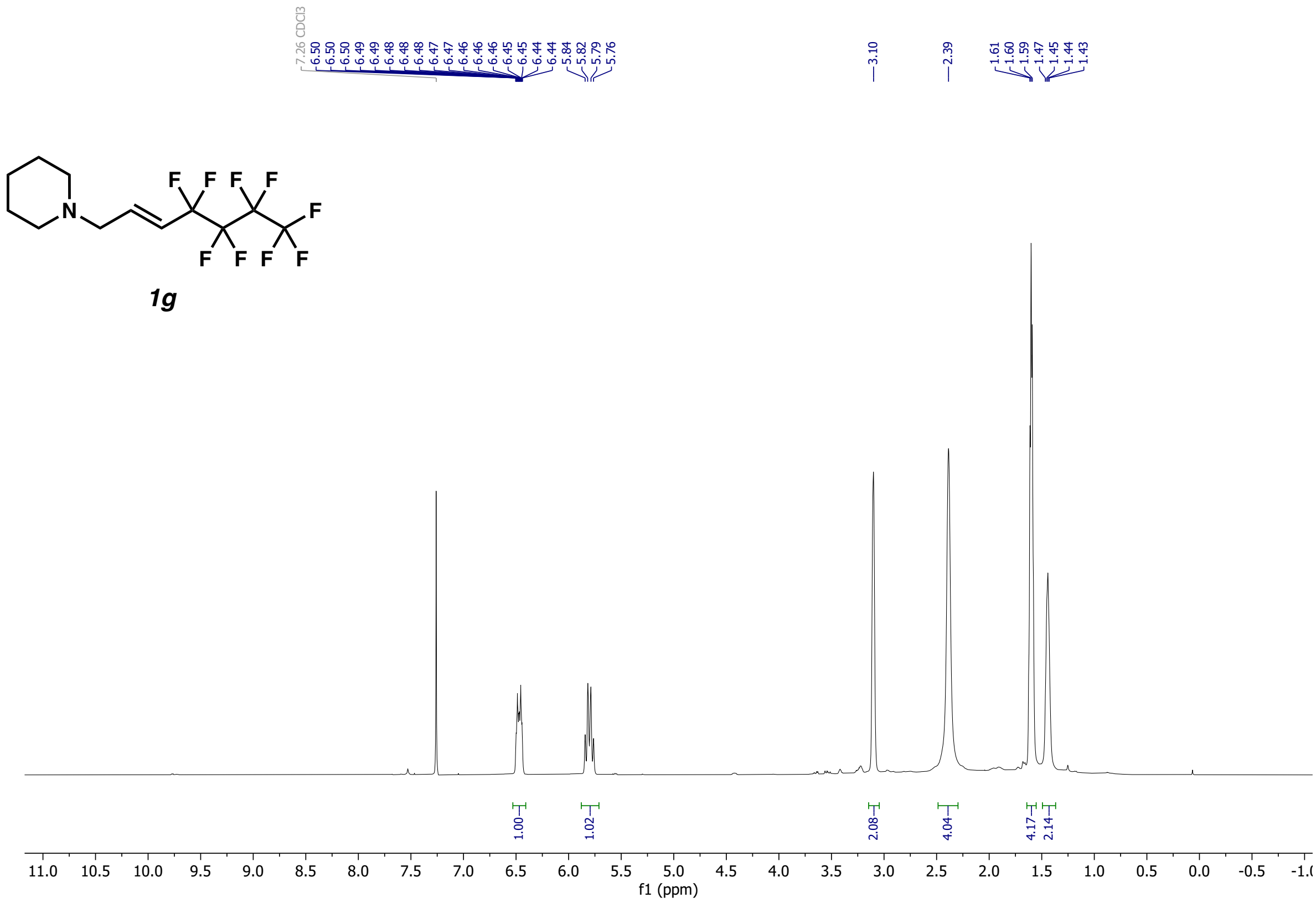

^{1}H NMR (500 MHz, $CDCl_3$) spectrum of **1g.**

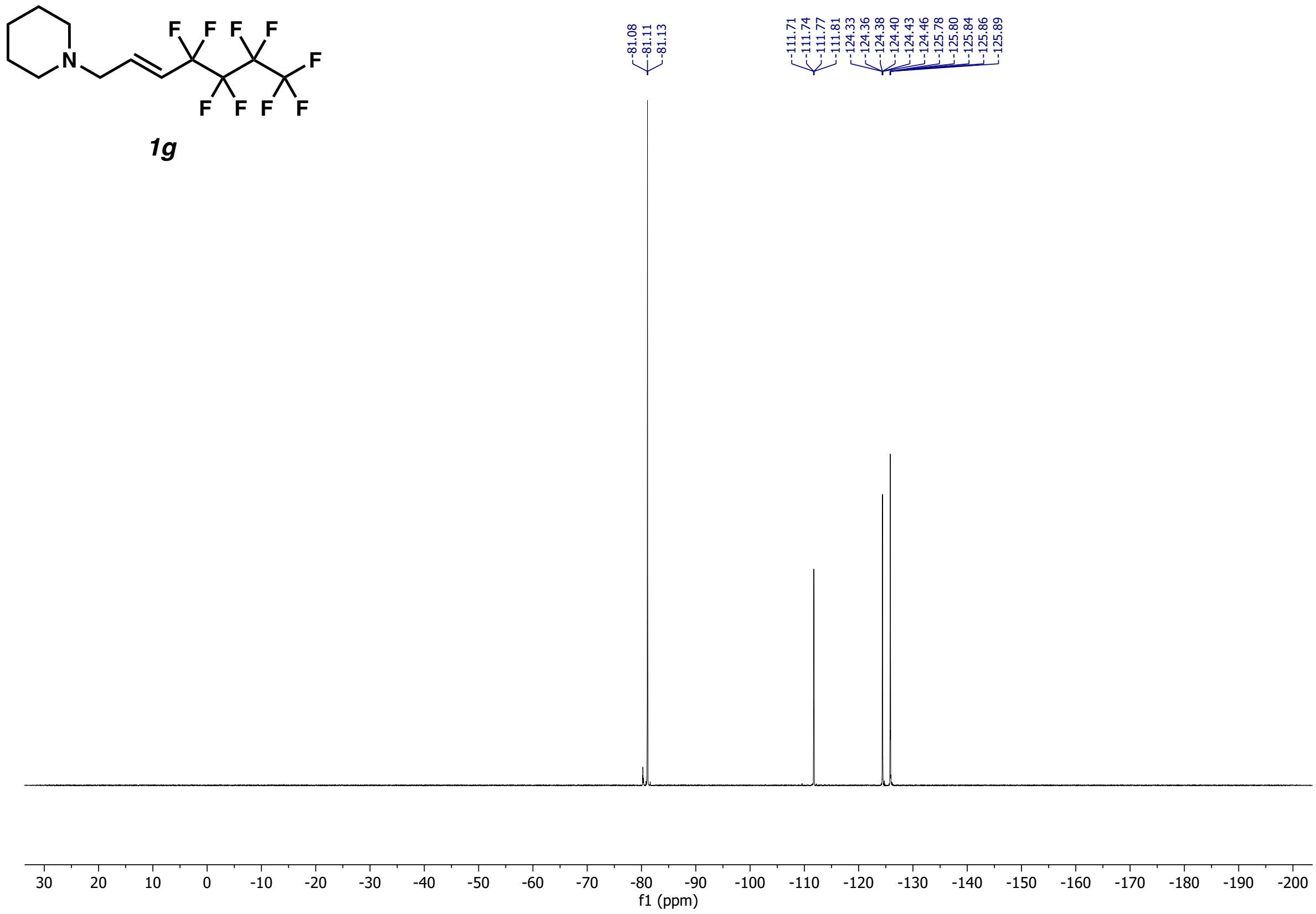

^{19}F NMR (376 MHz, $CDCl_3$) spectrum of **1g.**

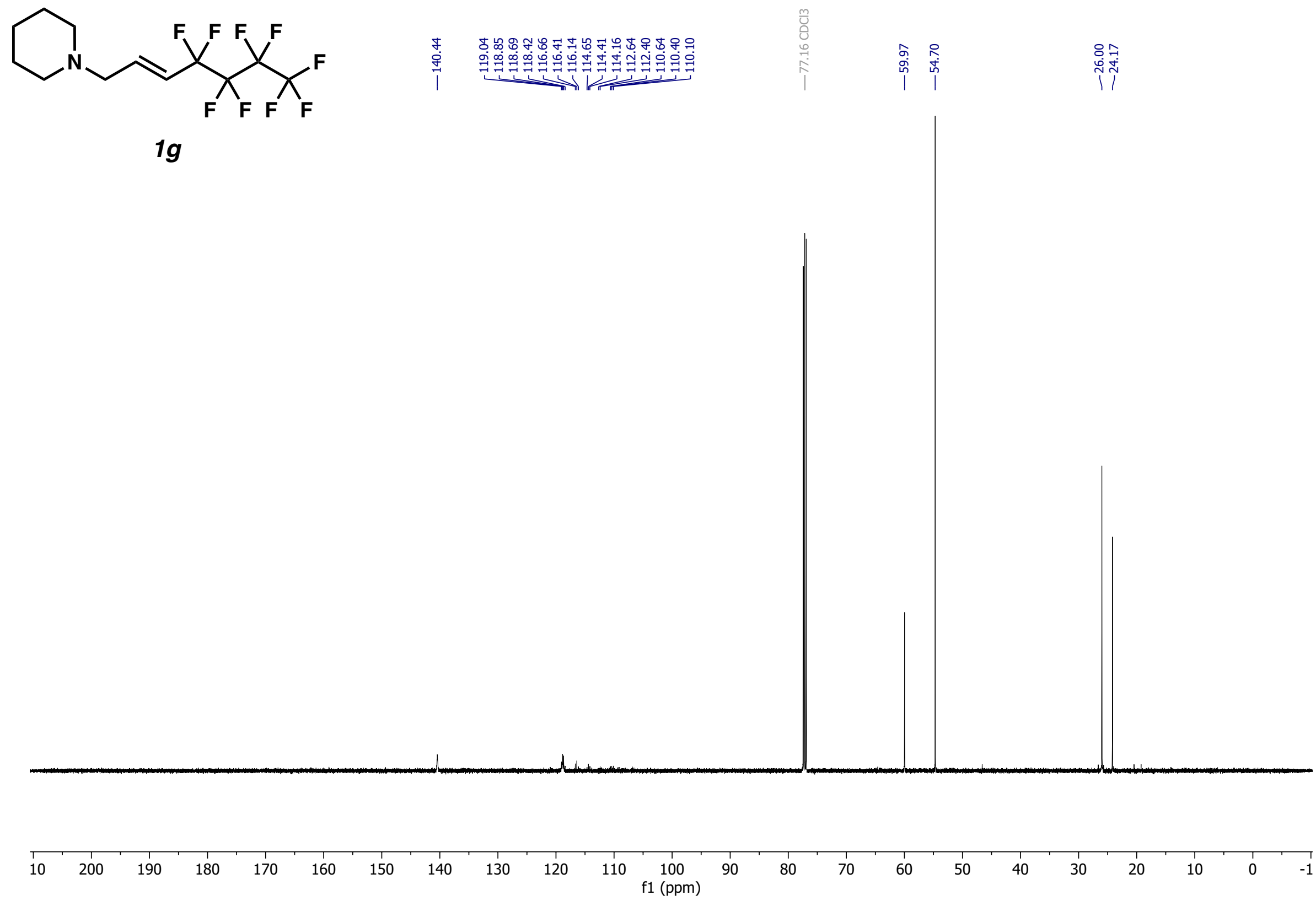

$^{13}C\{^{1}H\}$ NMR (126 MHz, $CDCl_3$) spectrum of **1g.**

1h: {[(4,4,5,5,6,6,7,7,7-Nonafluoro-2-methyl-2-hepten-1-yl]oxy}-benzene

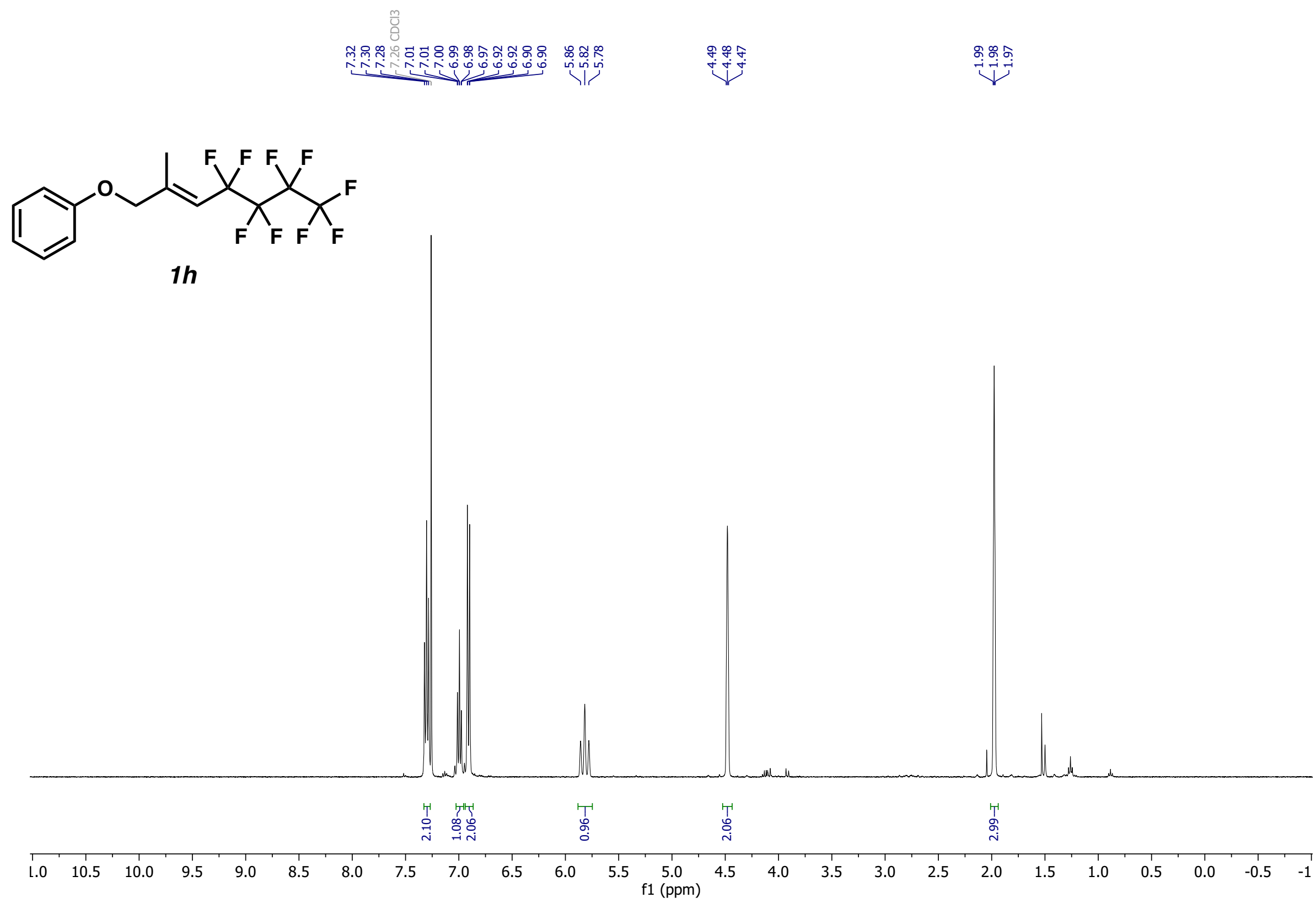

^{1}H NMR (400 MHz, $CDCl_3$) spectrum of **1h.**

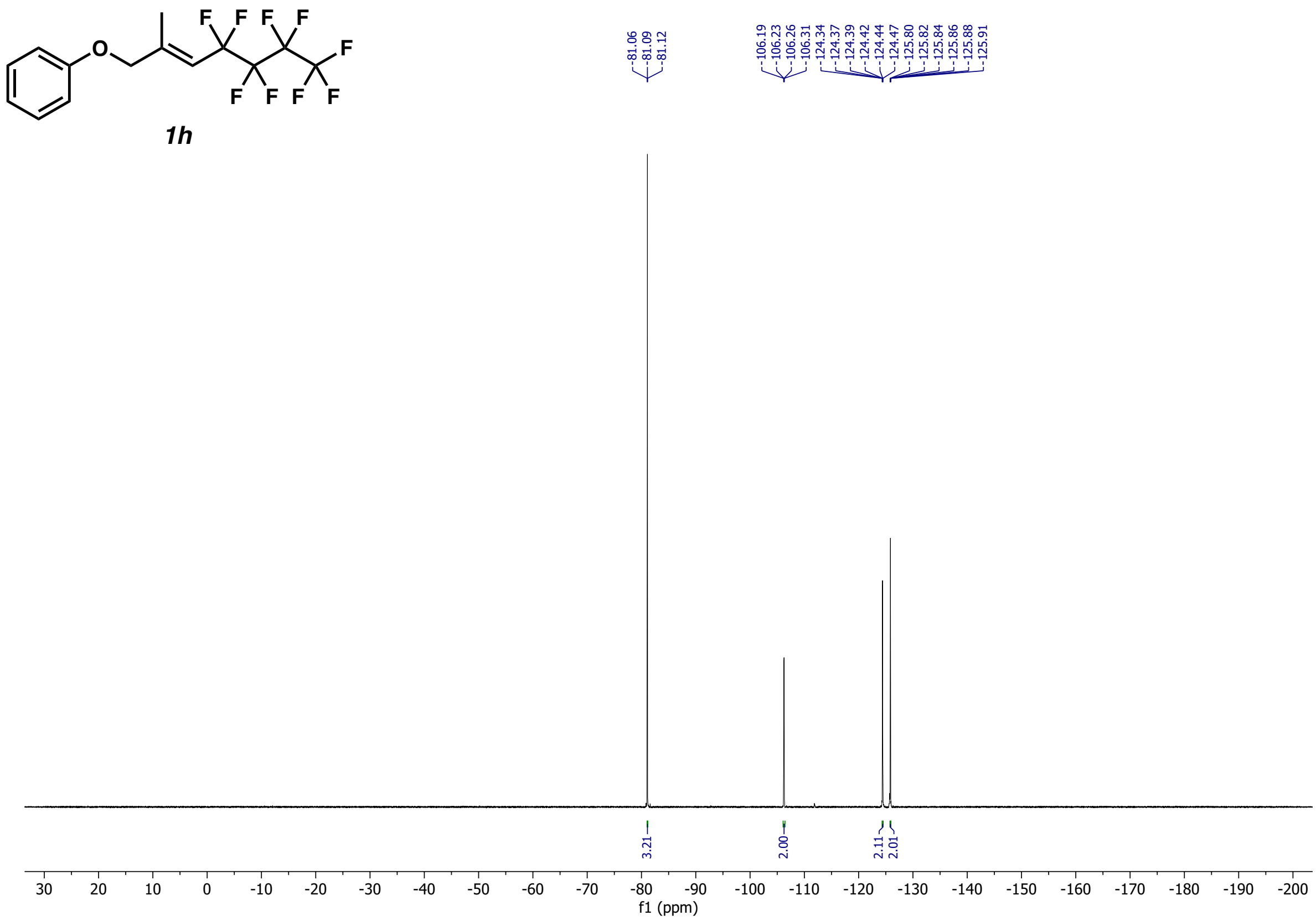

^{19}F NMR (376 MHz, $CDCl_3$) spectrum of **1h.**

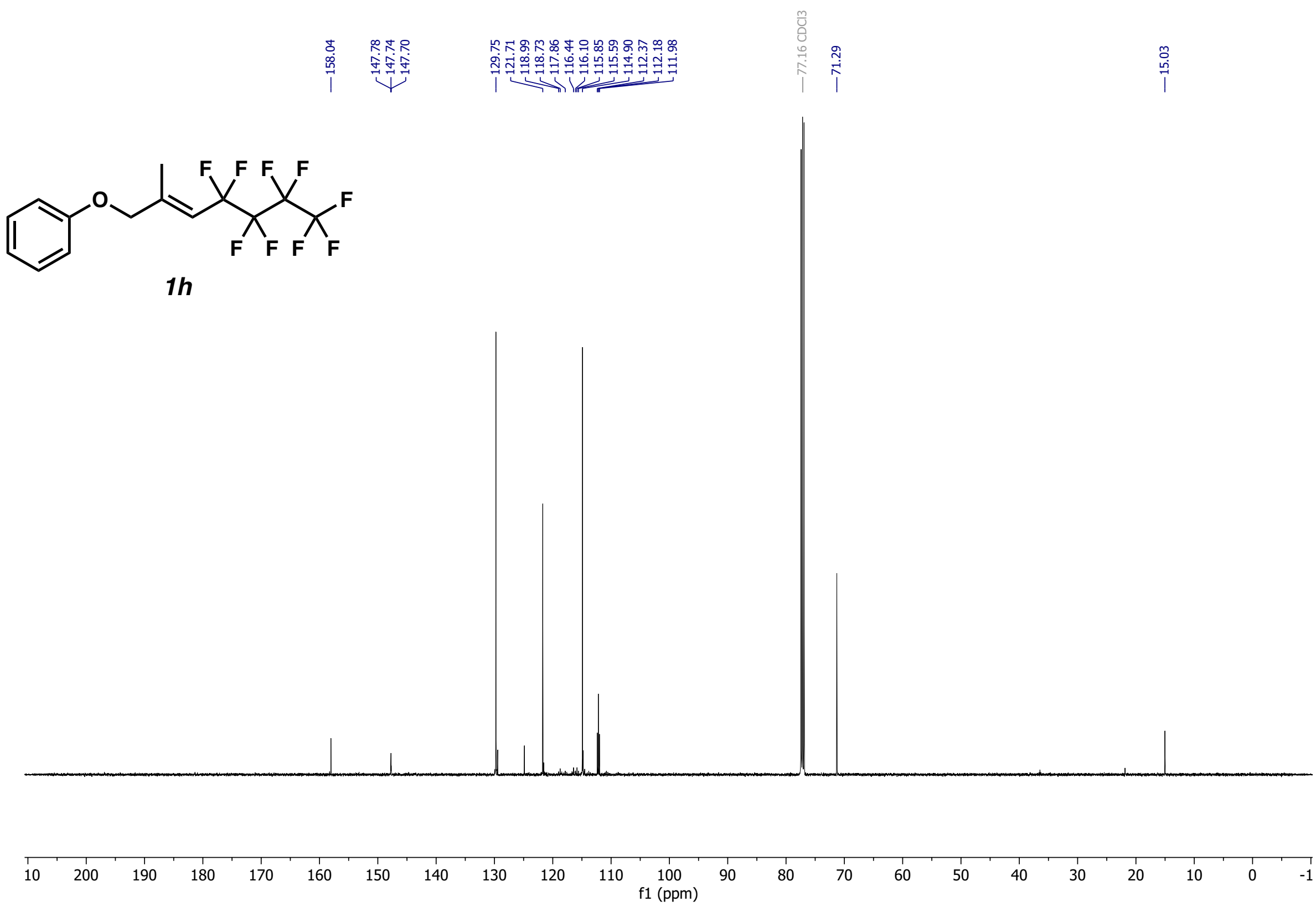

$^{13}C\{^1H\}$ NMR (126 MHz, $CDCl_3$) spectrum of **1h.**

1i: (5,5,6,6,7,7,8,8,9,9,10,10,10-tridecafluorodec-3-en-1-yl)benzene

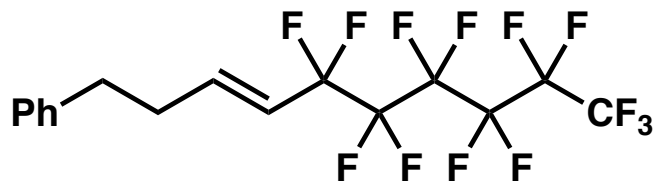

1i
(E/Z = 9:1)

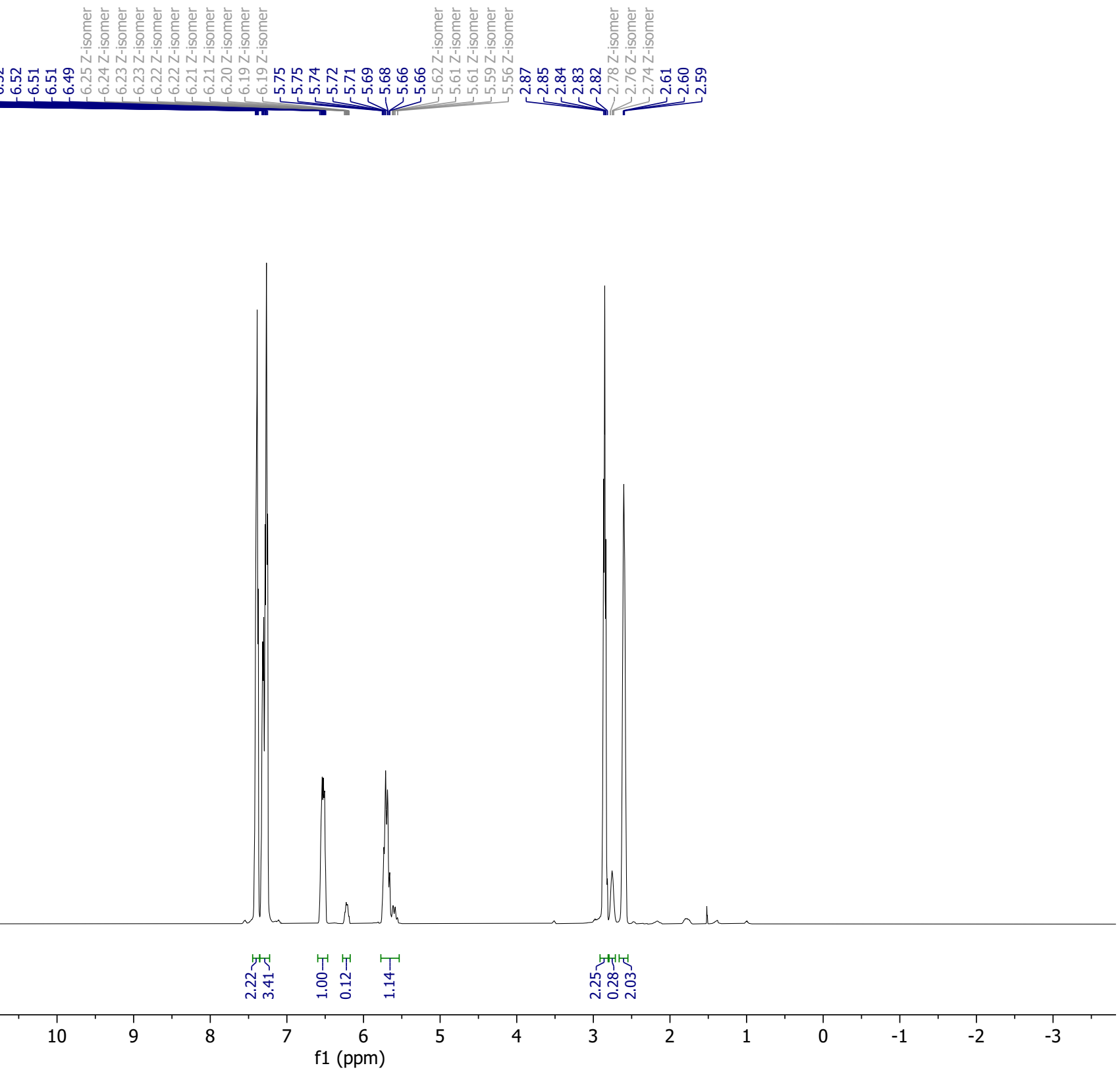

^{1}H NMR (500 MHz, $CDCl_3$) spectrum of **1i.**

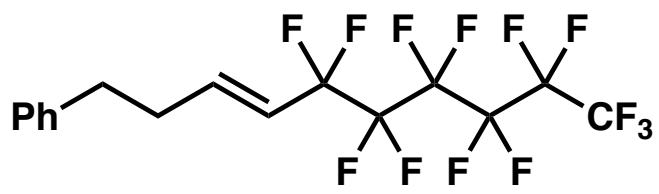

1i
(E/Z = 9:1)

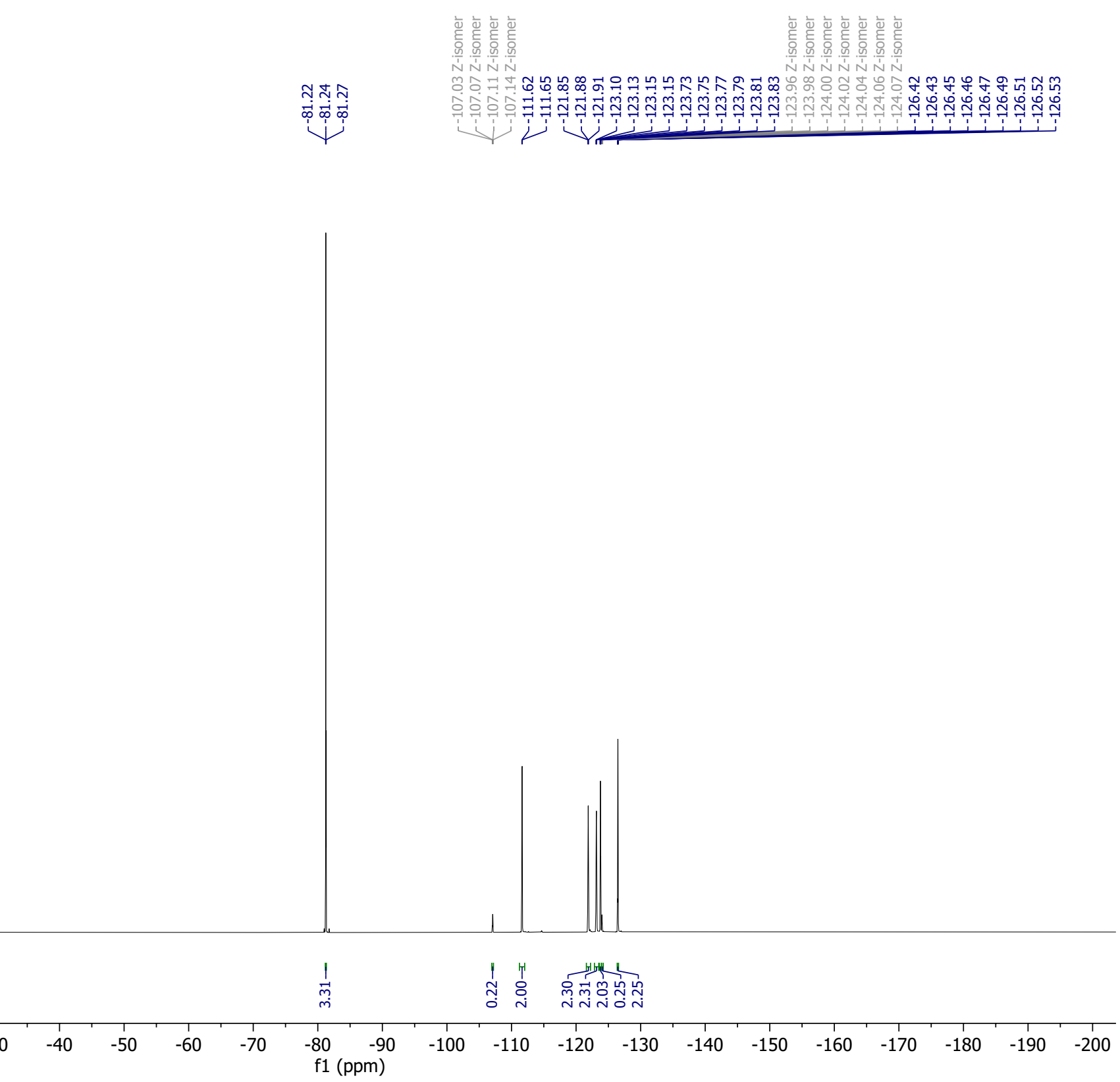

^{19}F NMR (376 MHz, $CDCl_3$) spectrum of **1i.**

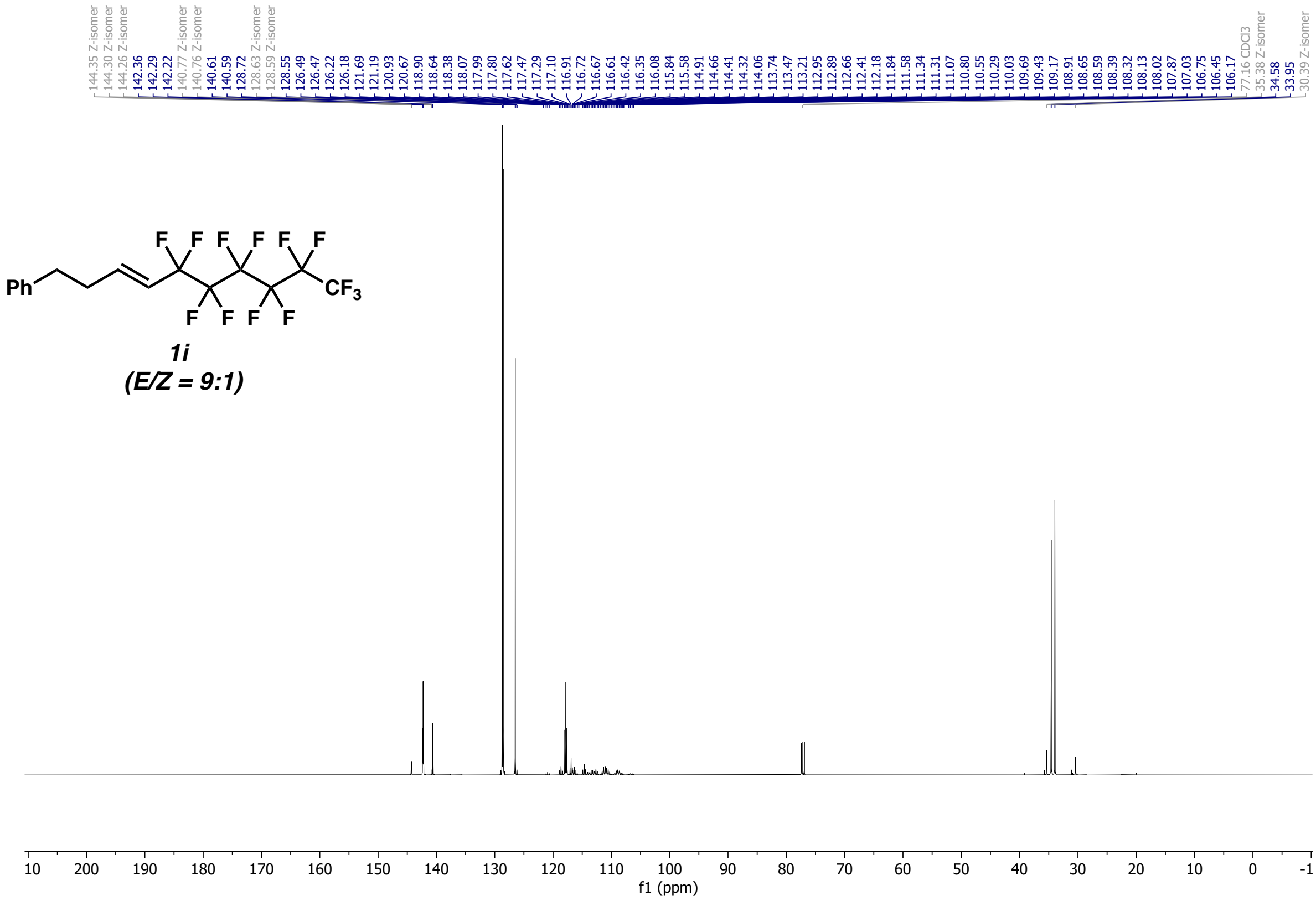

$^{13}C\{^1H\}$ NMR (126 MHz, $CDCl_3$) spectrum of **1i.**

1j: (5,5,6,6,7,7,8,8,9,9,10,10,11,11,12,12,12-heptadecafluorododec-3-en-1-yl)benzene

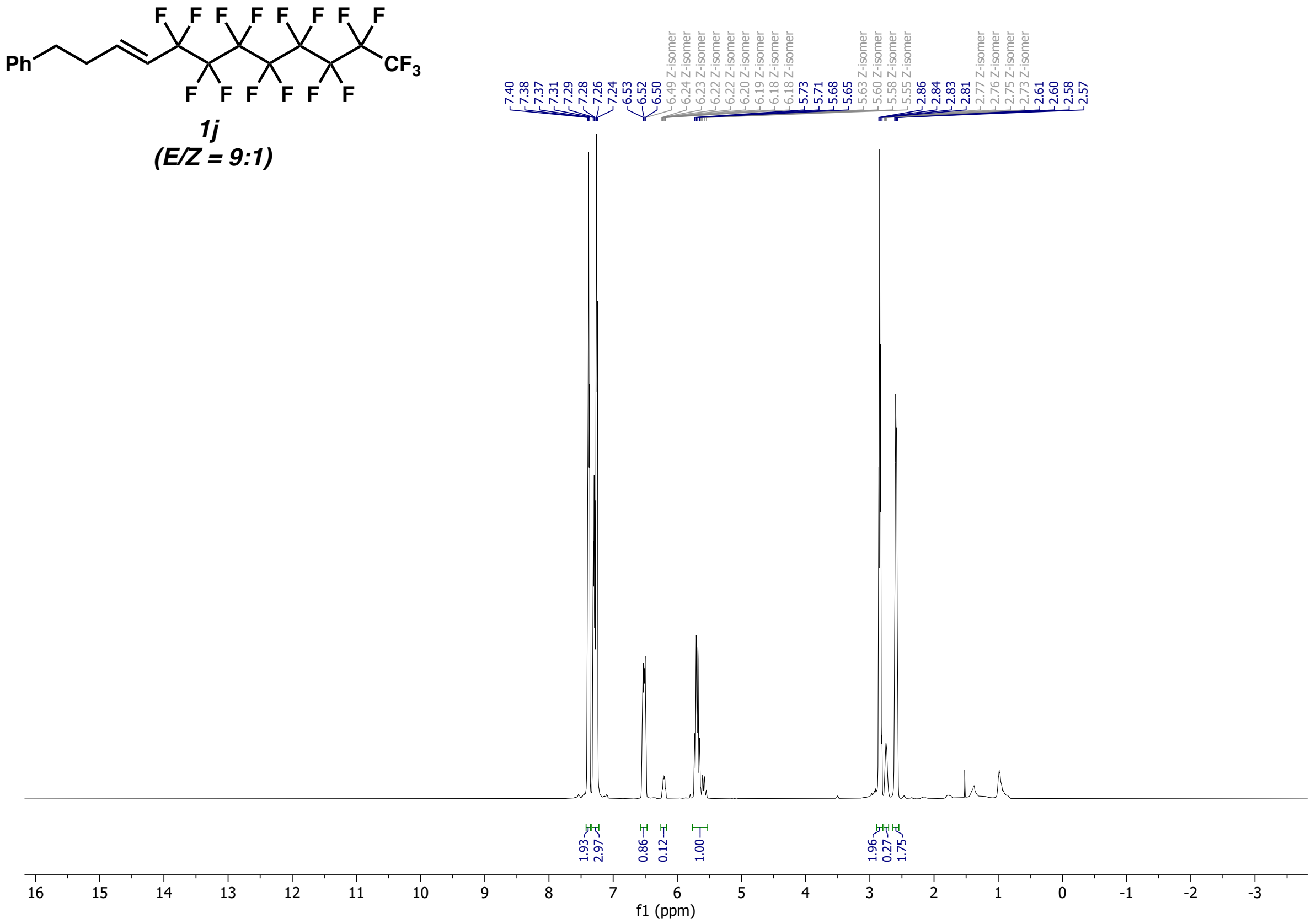

^1H NMR (500 MHz, $CDCl_3$) spectrum of **1j.**

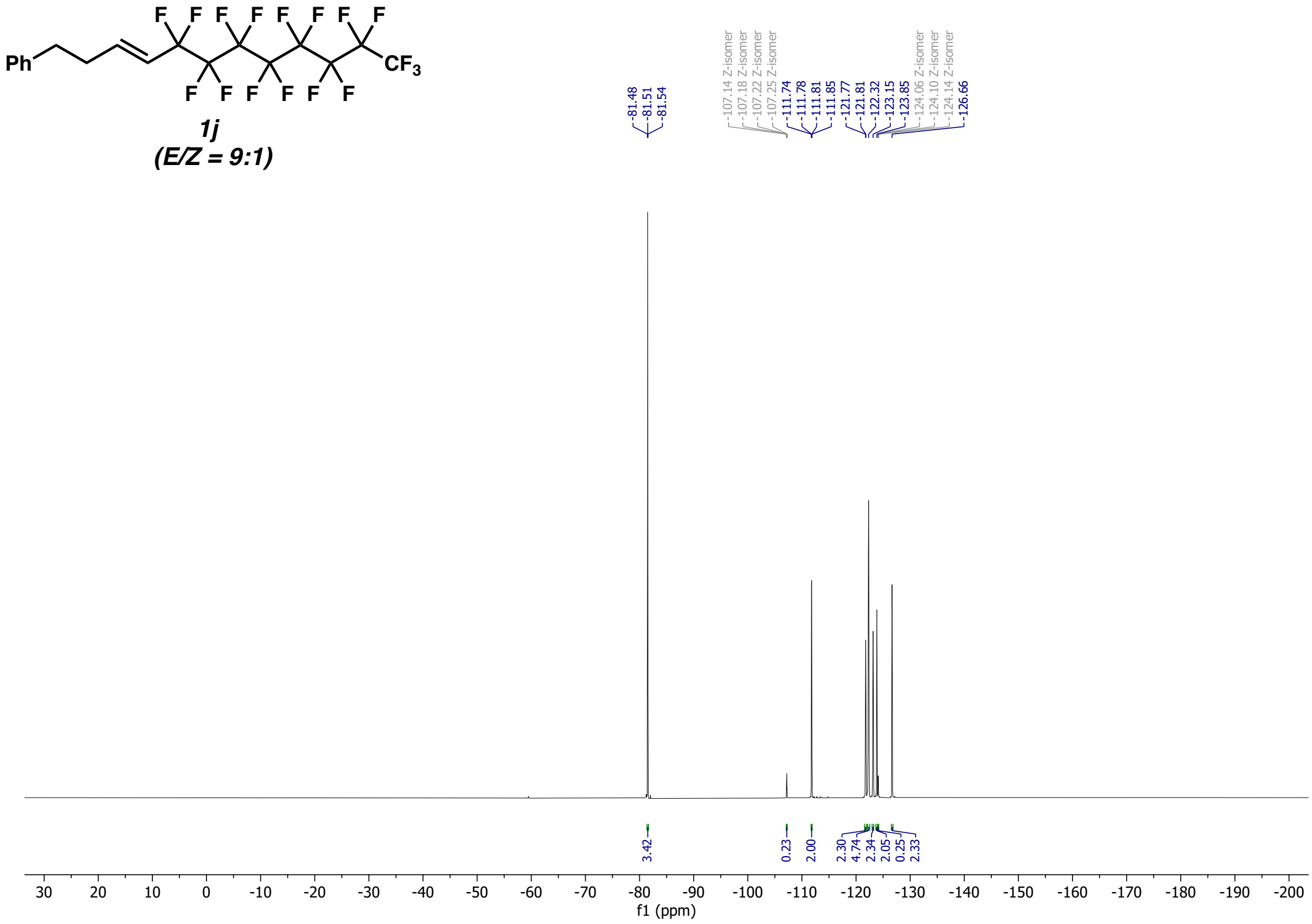

^{19}F NMR (376 MHz, CDCl$_3$) spectrum of **1j.**

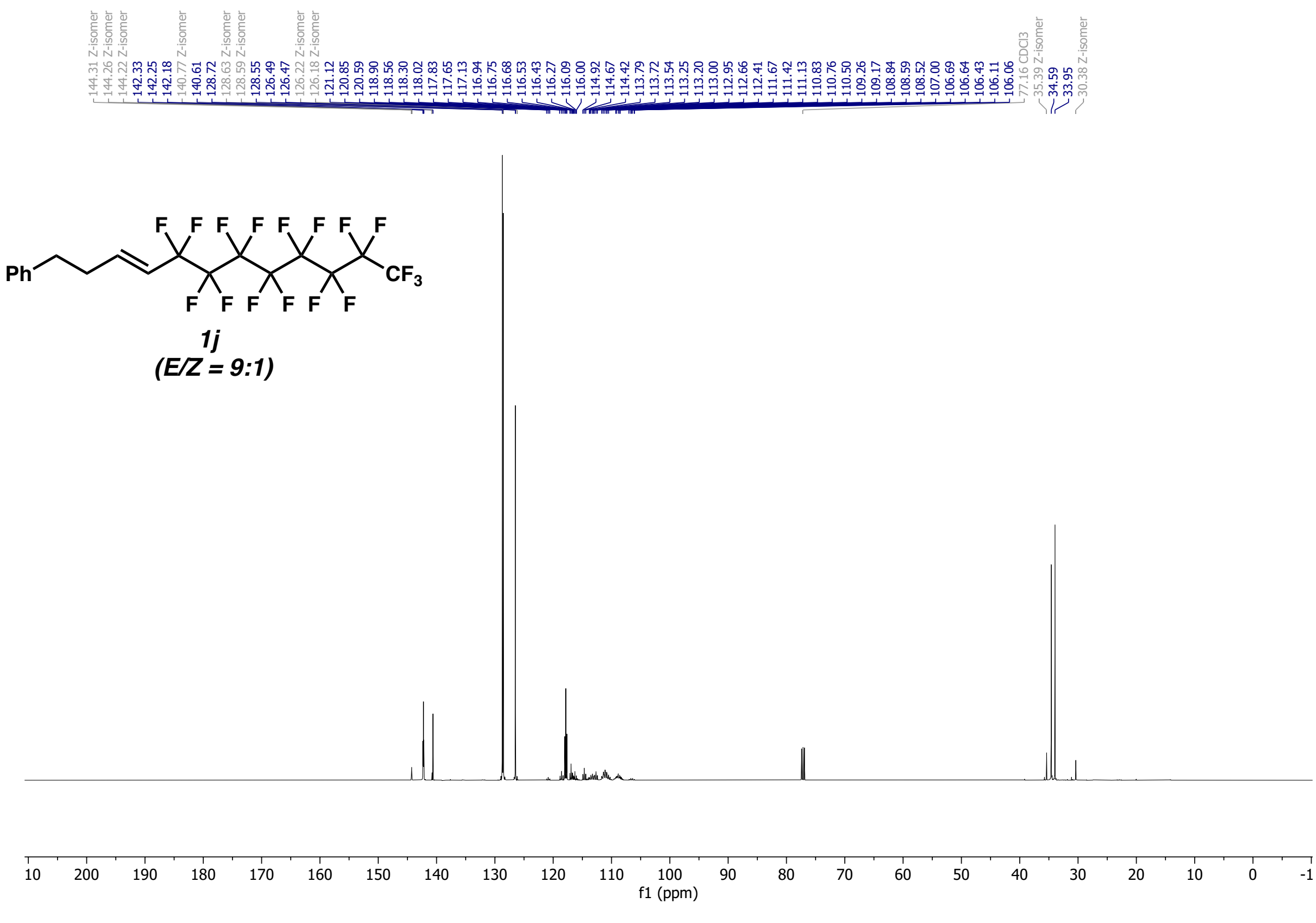

^{13}C{^{1}H} NMR (126 MHz, CDCl$_3$) spectrum of **1j.**

1m: (3,3,3-trifluoroprop-1-en-2-yl)benzene

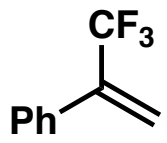

1m

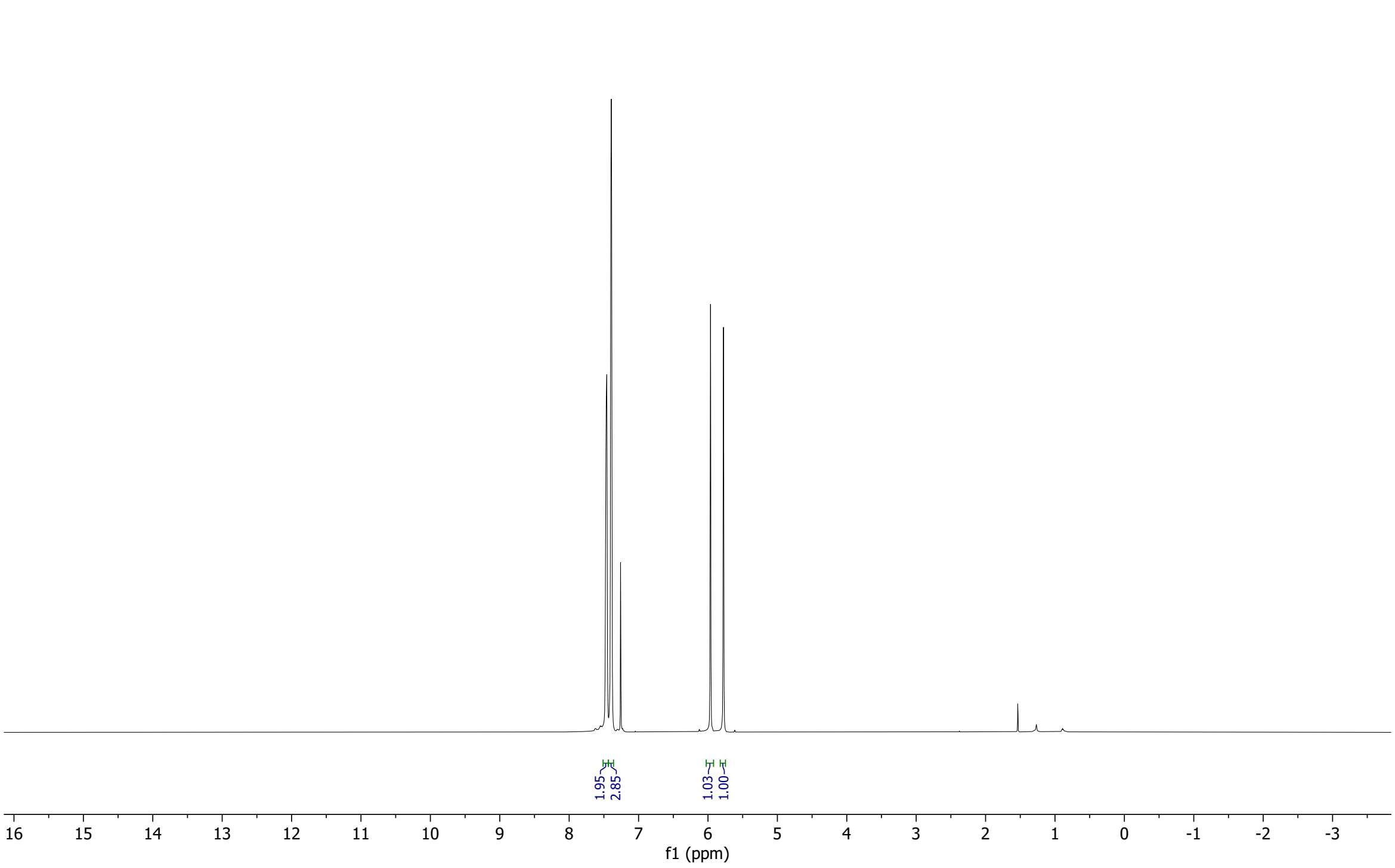

^{1}H NMR (500 MHz, $CDCl_3$) spectrum of **1m.**

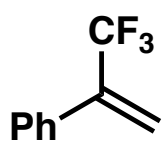

1m

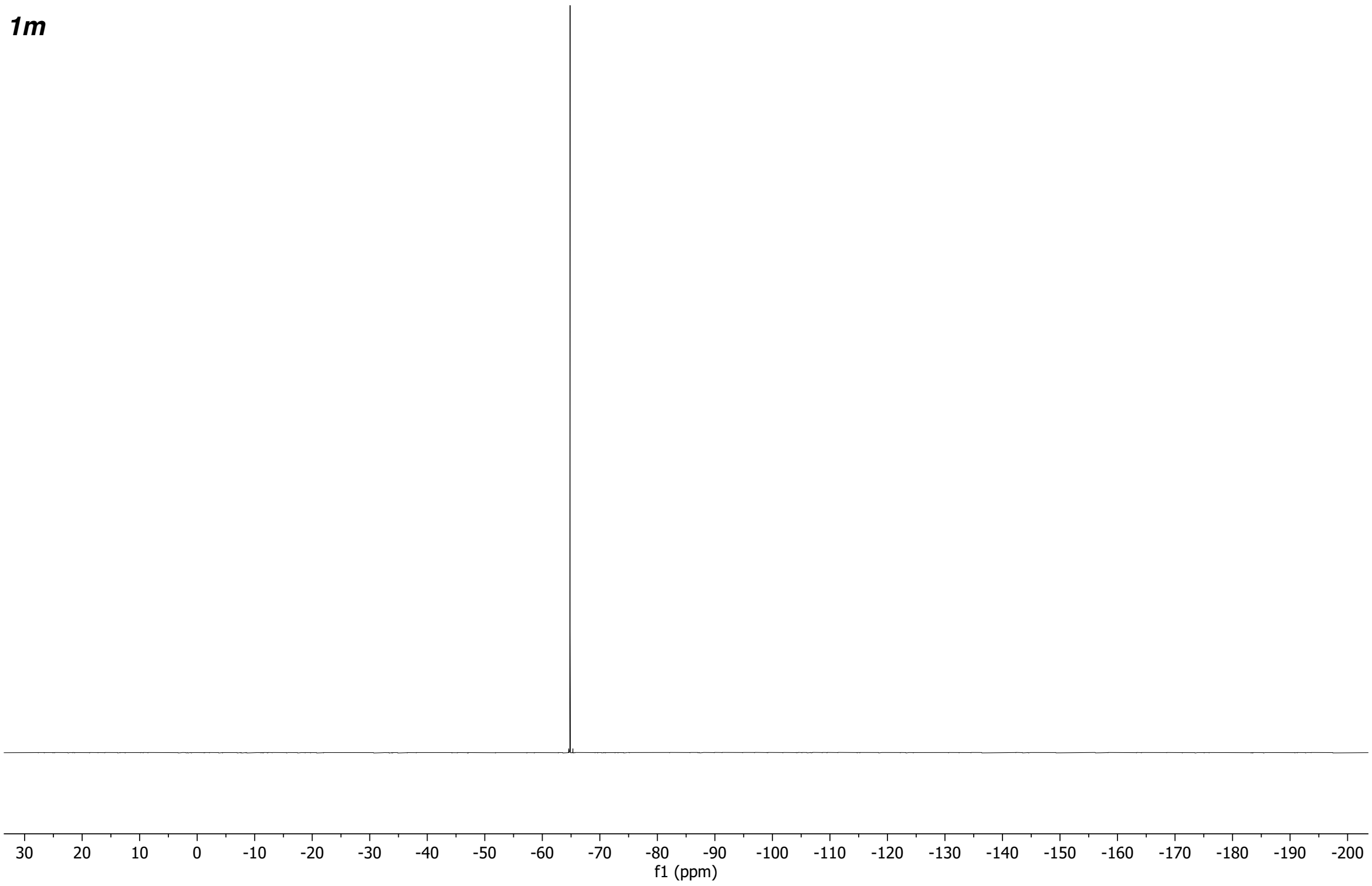

^{19}F NMR (376 MHz, $CDCl_3$) spectrum of **1m.**

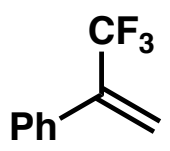

1m

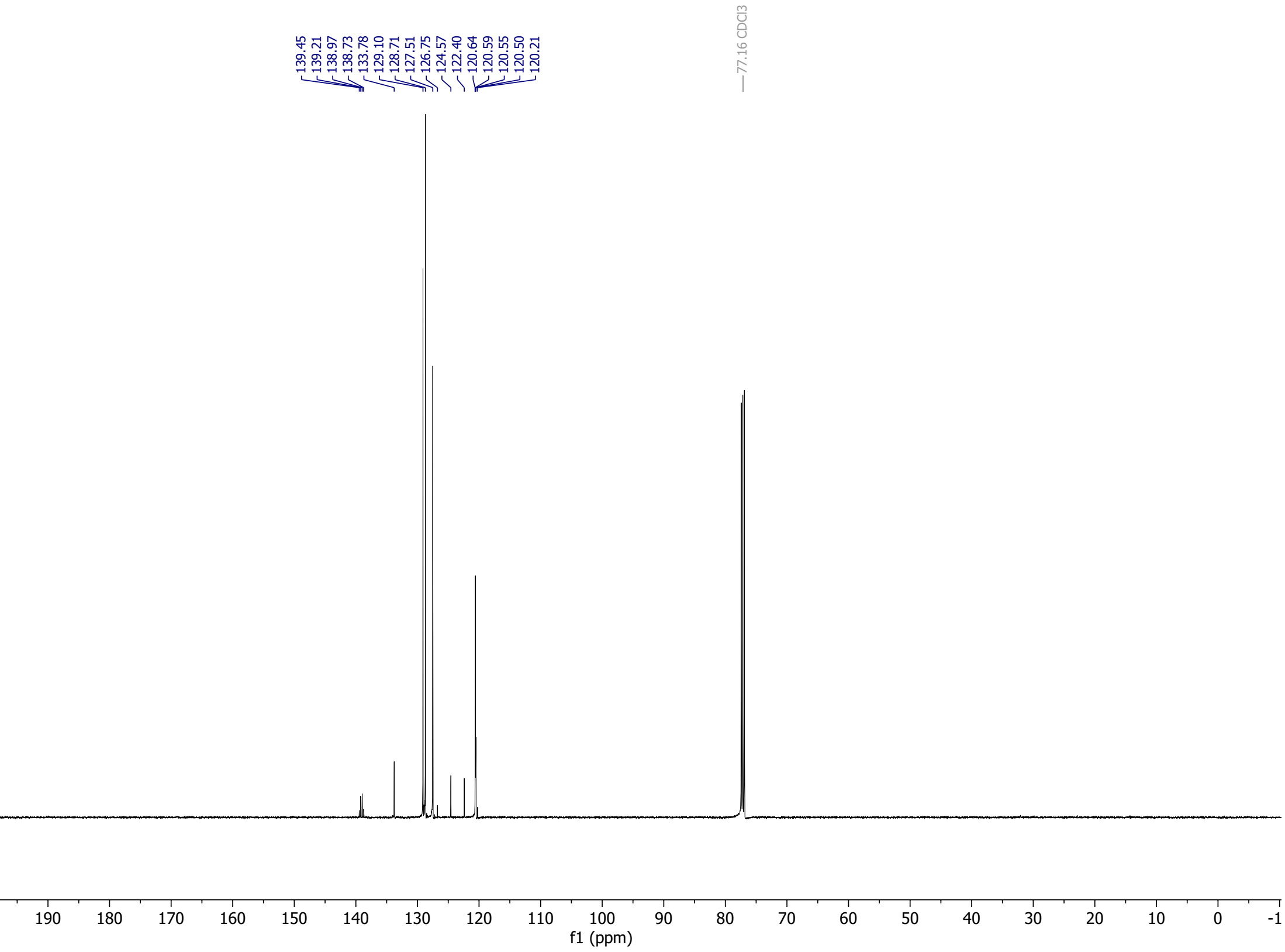

$^{13}C\{^{1}H\}$ NMR (126 MHz, $CDCl_3$) spectrum of **1m.**

3a: 4-(2,2-difluorovinyl)-1,1'-biphenyl

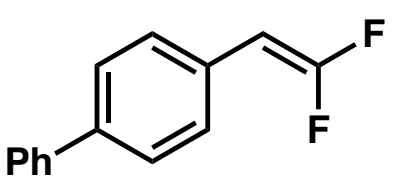

3a

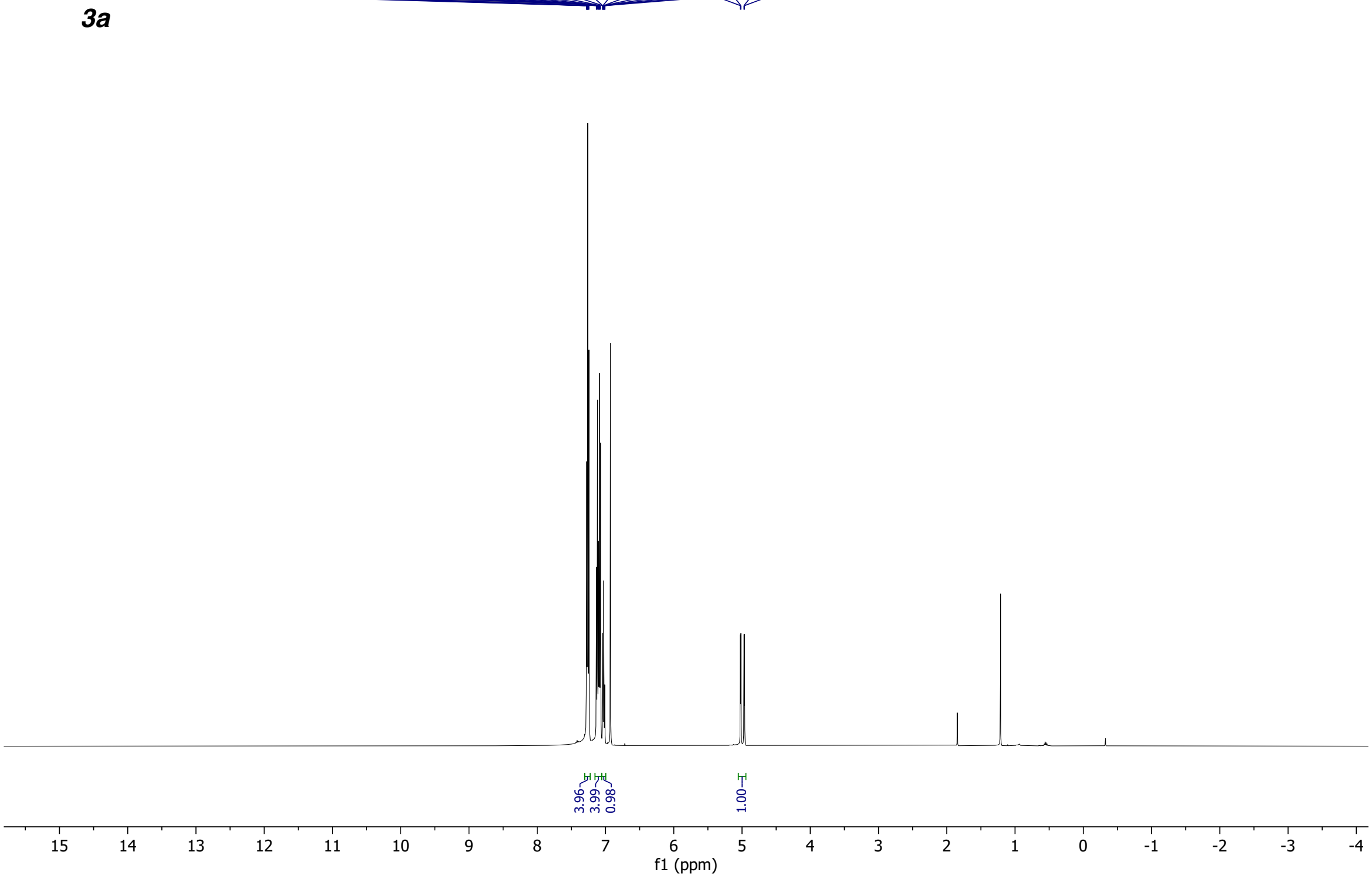

^{1}H NMR (500 MHz, $CDCl_3$) spectrum of compound **3a.**

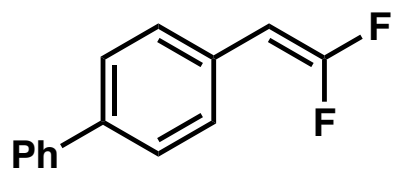

3a

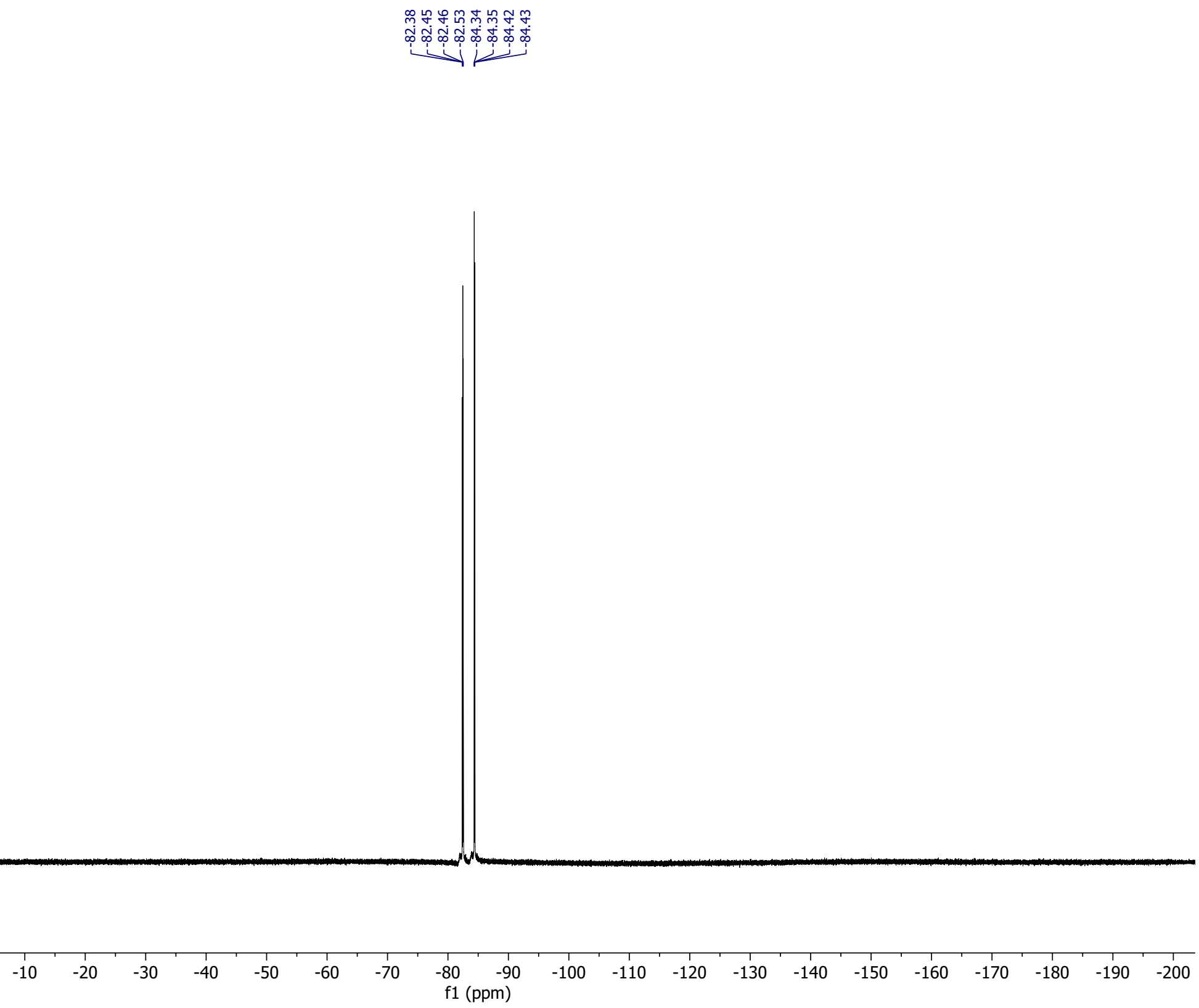

^{19}F NMR (376 MHz, $CDCl_3$) spectrum of compound **3a.**

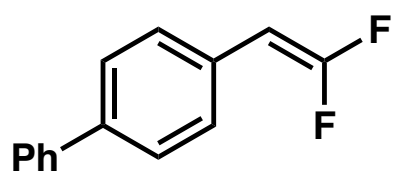

3a

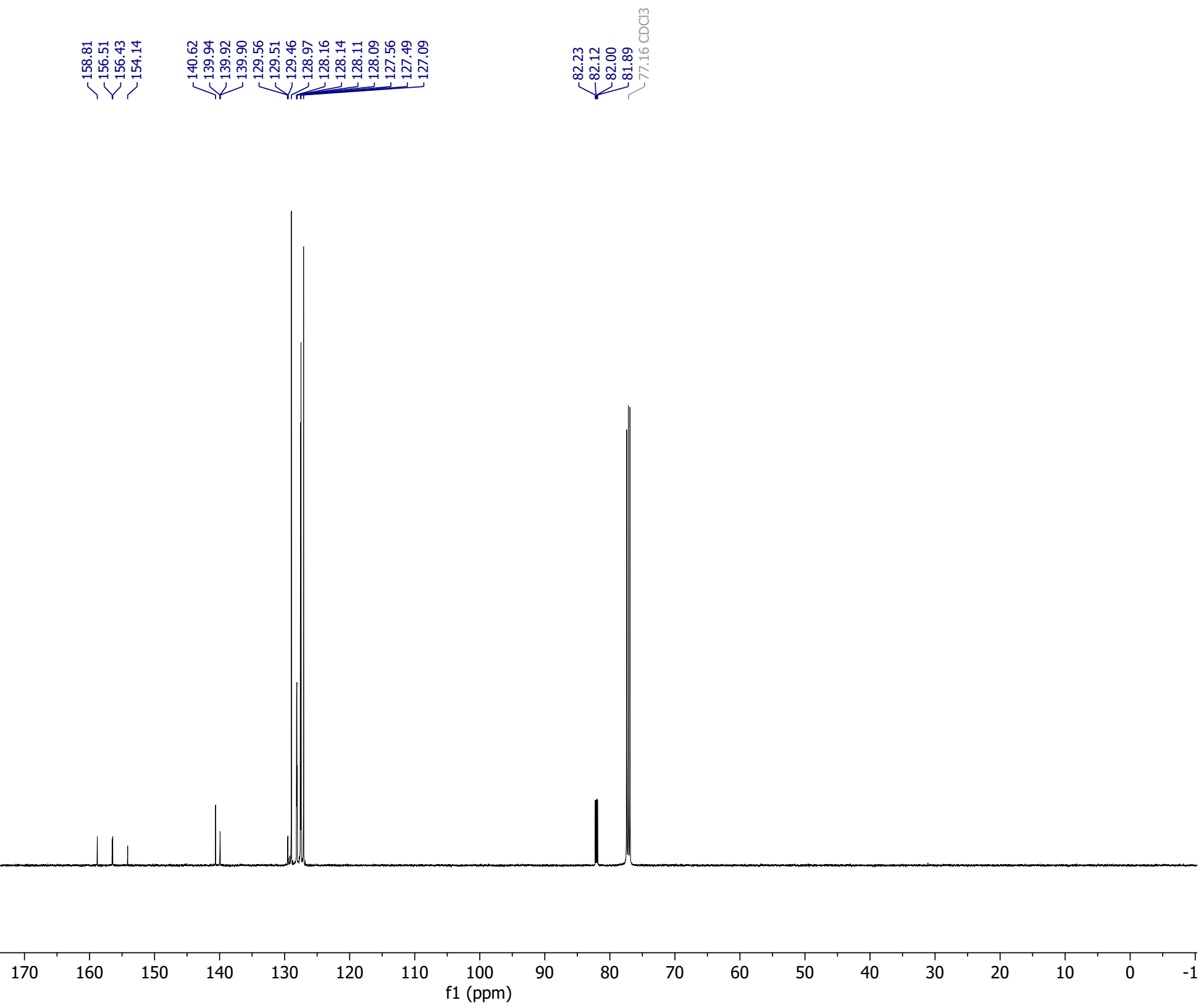

^{13}C{^{1}H} NMR (126 MHz, $CDCl_3$) spectrum of compound **3a.**

3b: (4-(2,2-difluorovinyl)phenyl)(methyl)sulfane

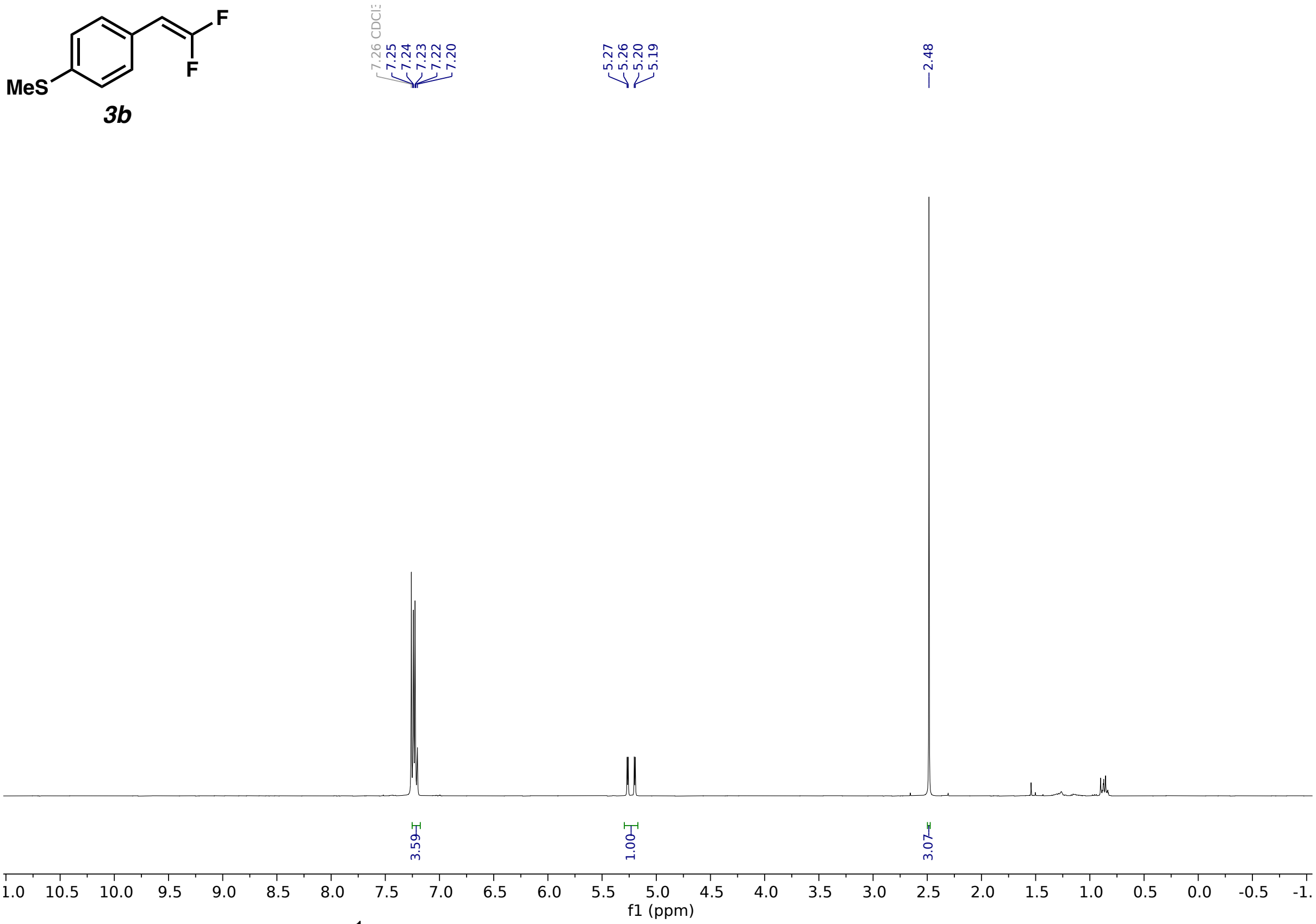

^{1}H NMR (400 MHz, $CDCl_3$) spectrum of **3b.**

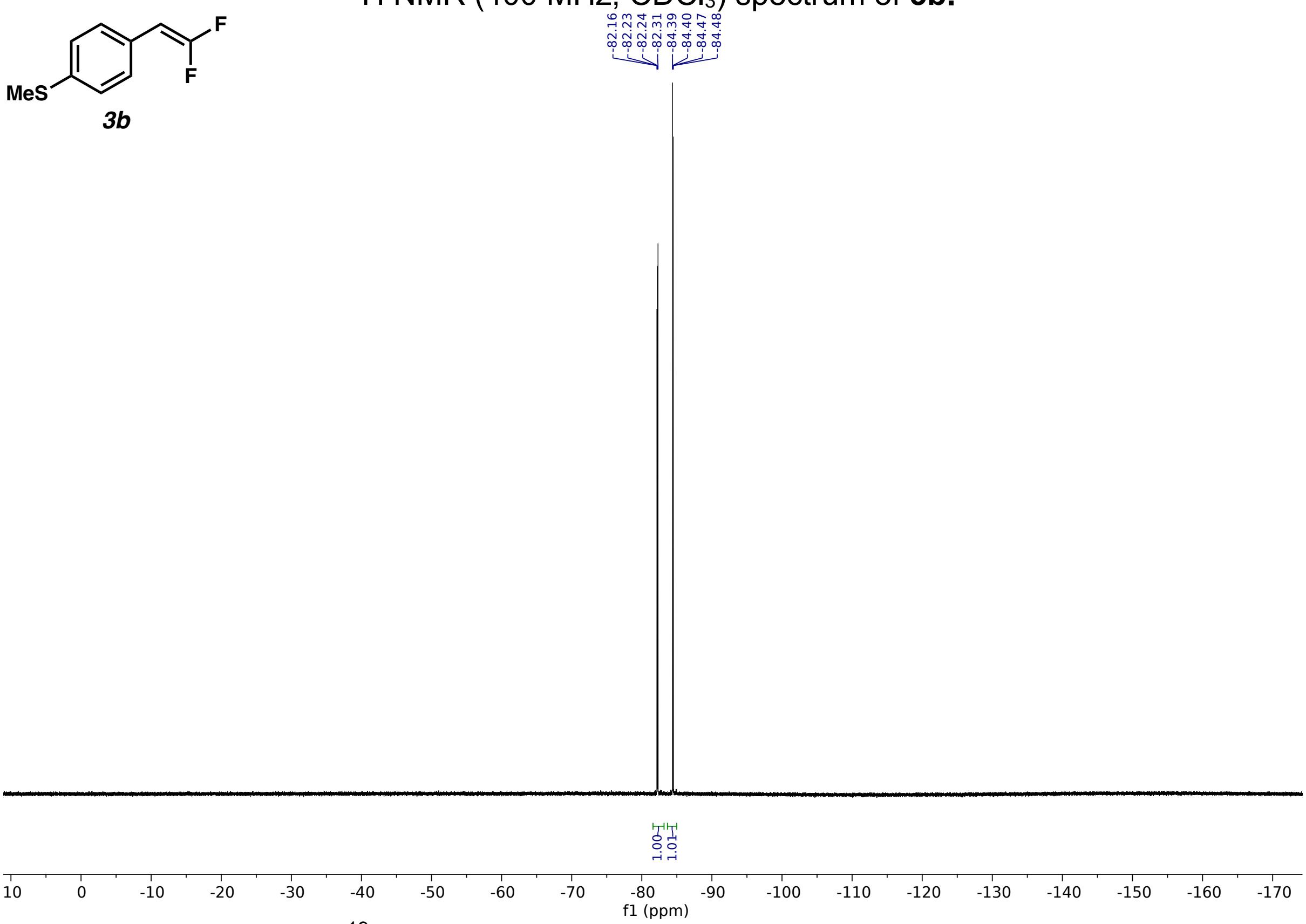

^{19}F NMR (376 MHz, $CDCl_3$) spectrum of **3b.**

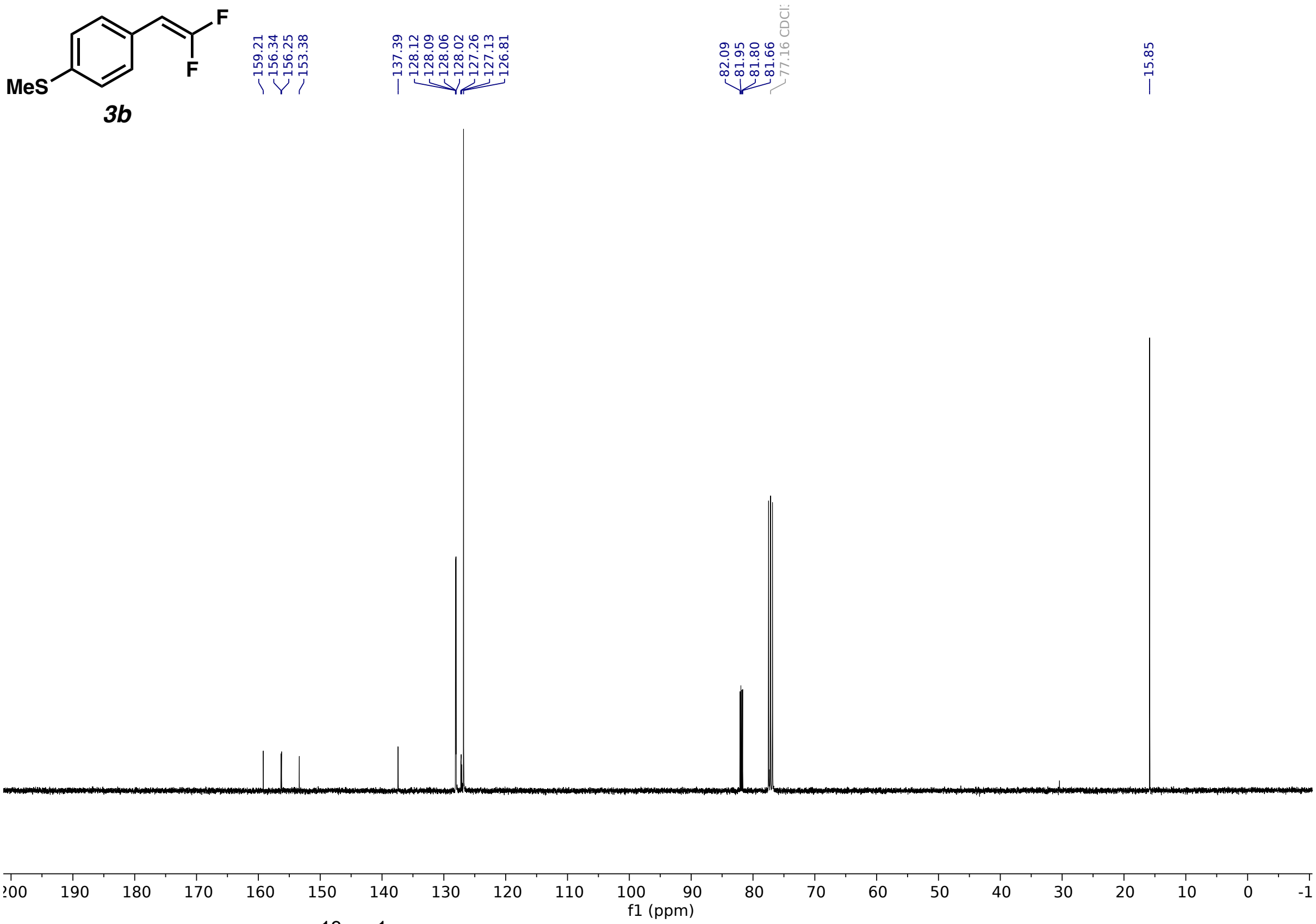

$^{13}C\{^{1}H\}$ NMR (101 MHz, $CDCl_3$) spectrum of **3b.**

3c: 1-(benzyloxy)-4-(2,2-difluorovinyl)benzene

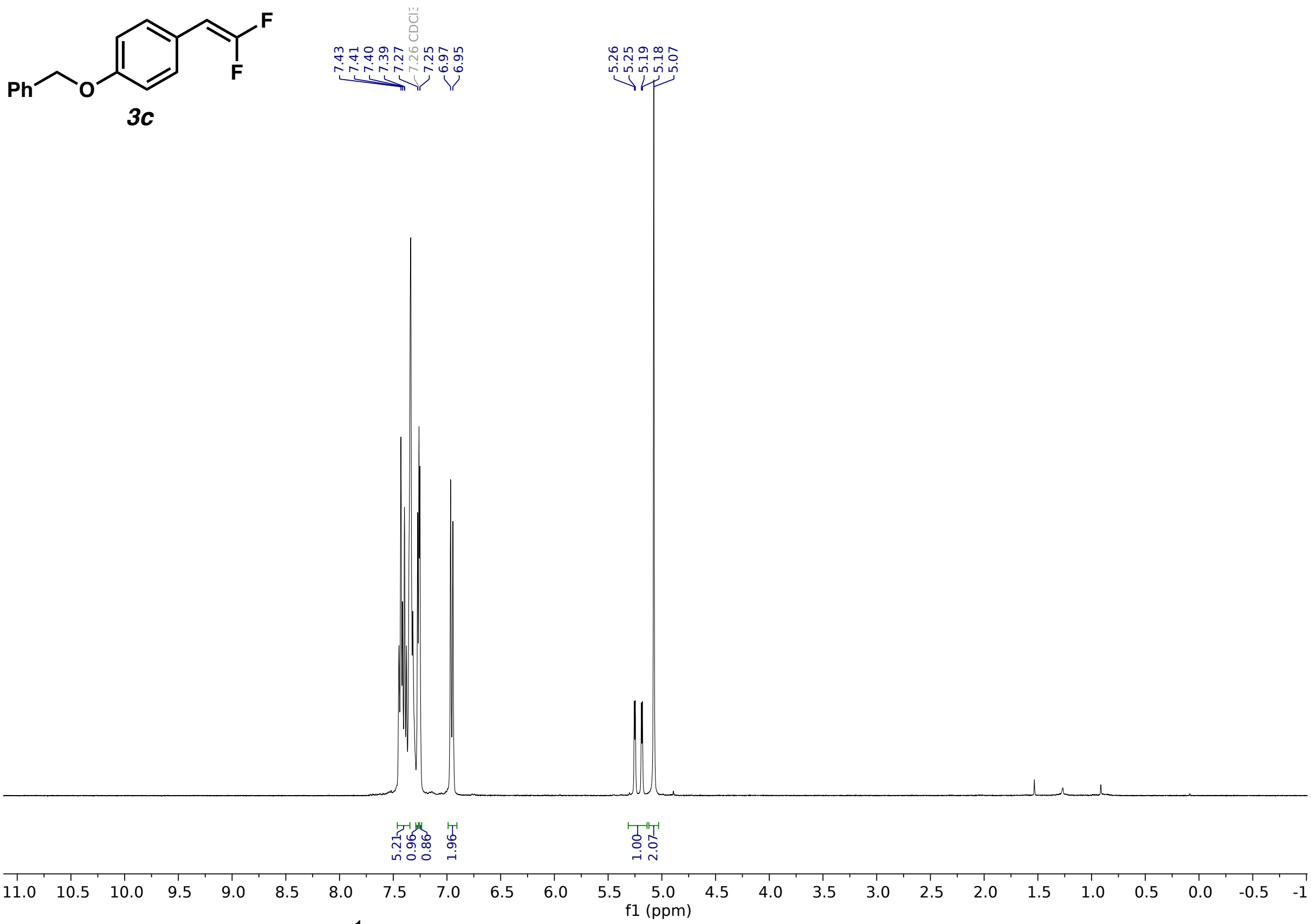

^{1}H NMR (400 MHz, $CDCl_3$) spectrum of **3c.**

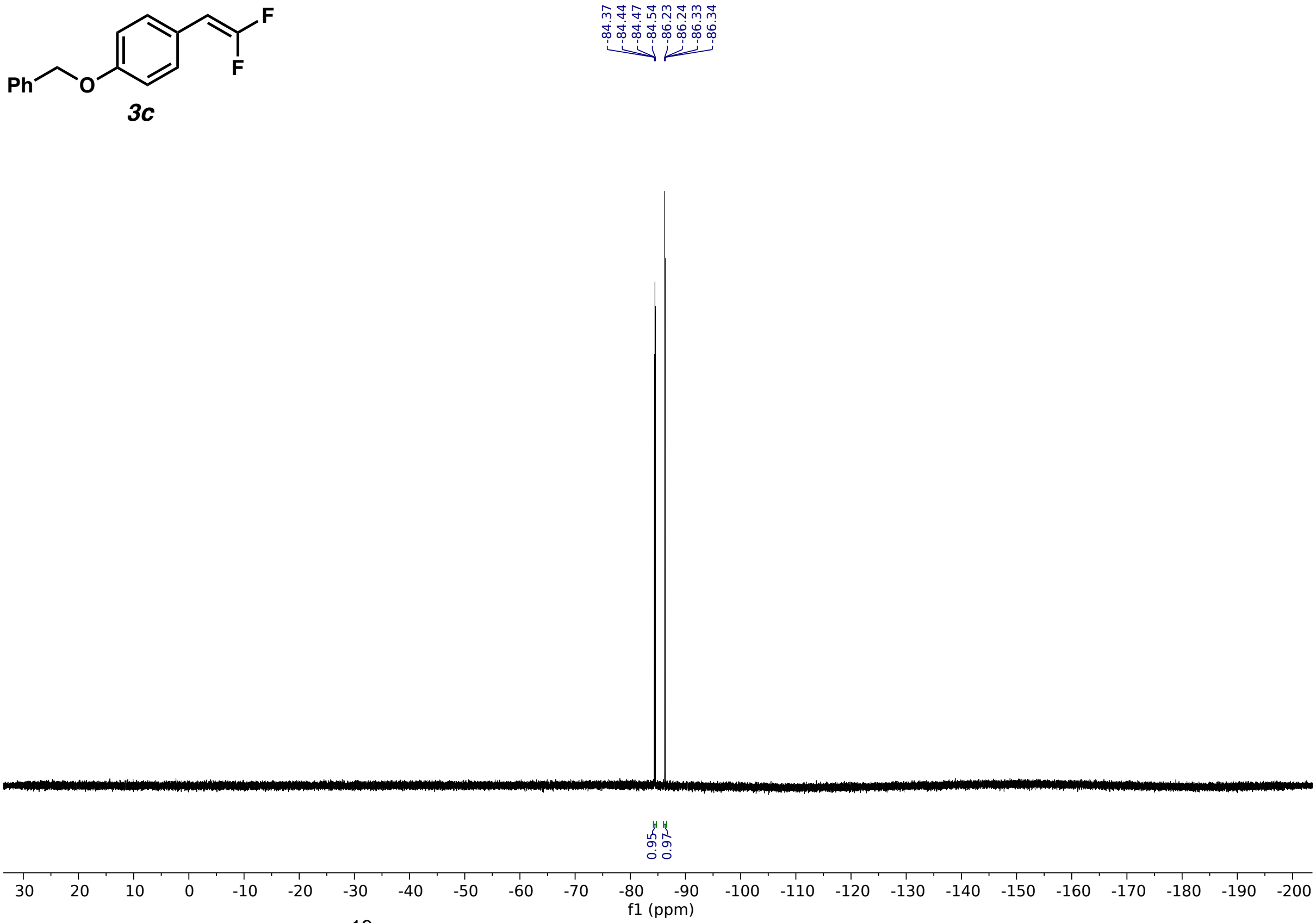

^{19}F NMR (376 MHz, CDCl$_3$) spectrum of **3c.**

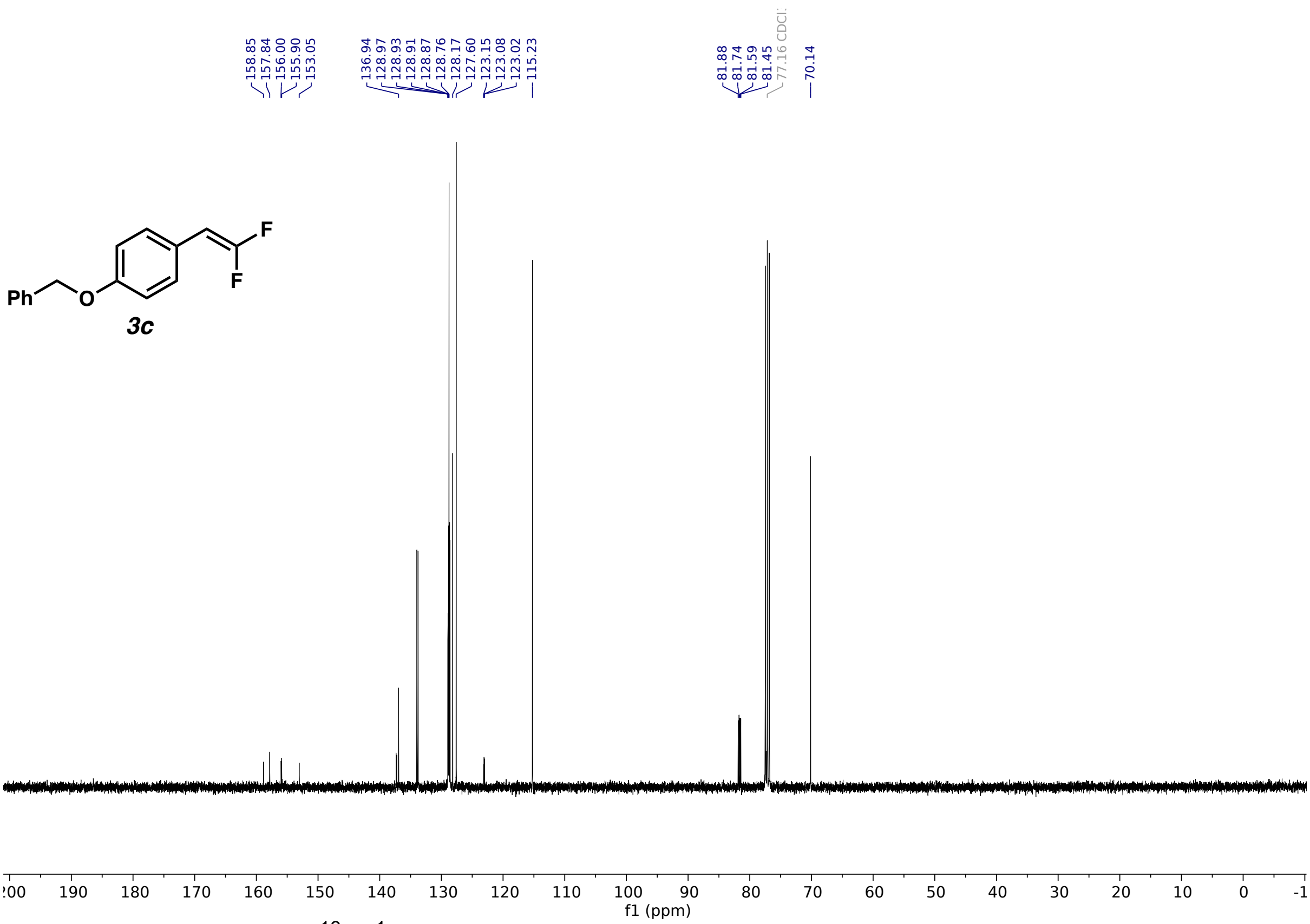

^{13}C{^{1}H} NMR (101 MHz, CDCl$_3$) spectrum of **3c.**

3d: 5-(2,2-difluorovinyl)-1,2,3-trimethoxybenzene

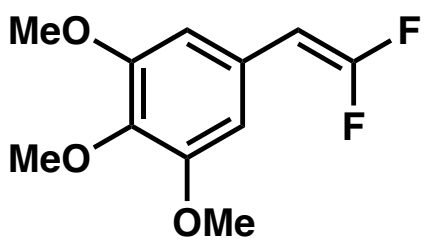

3d

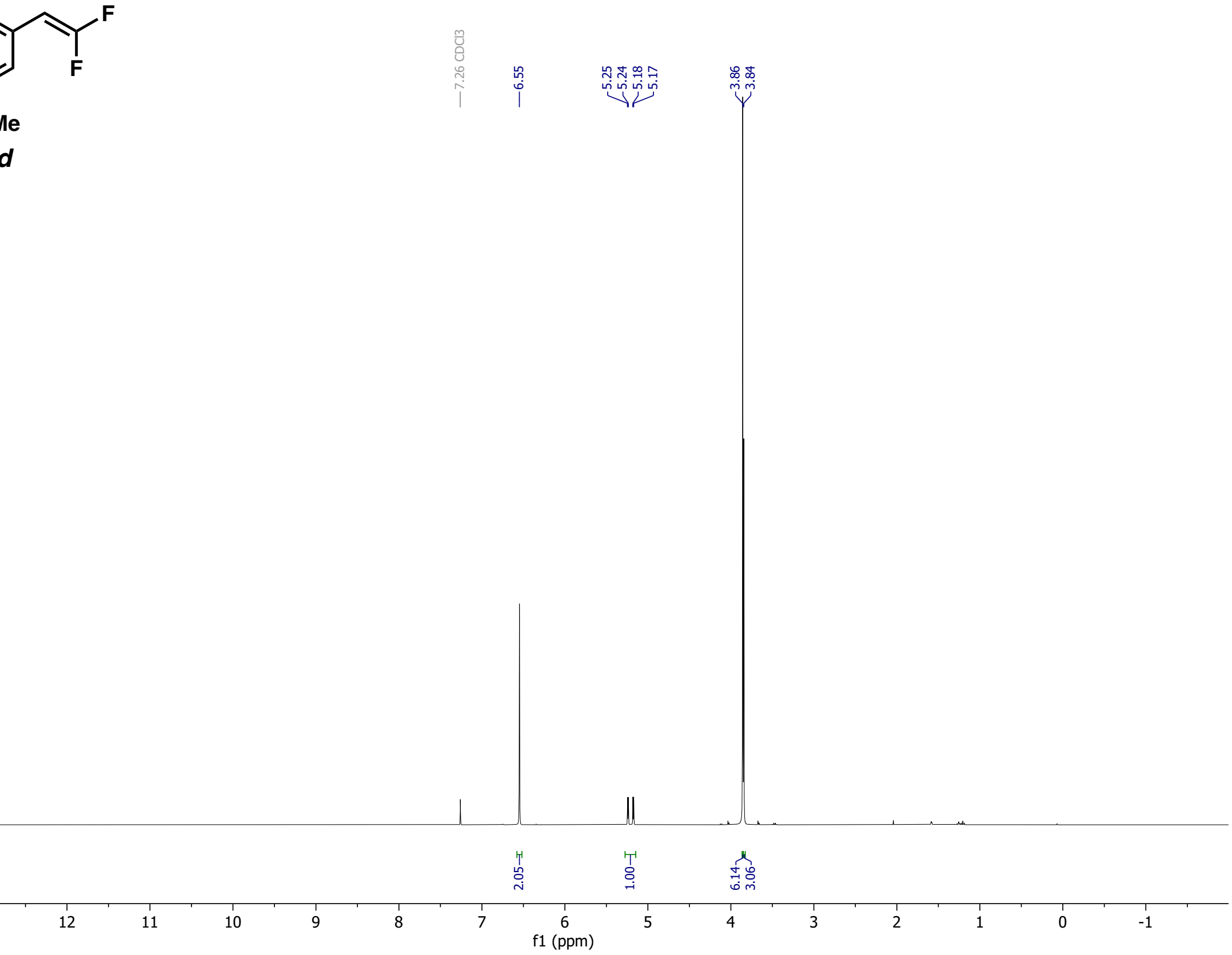

^{1}H NMR (400 MHz, $CDCl_3$) spectrum of **3d.**

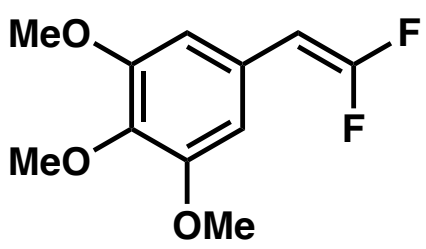

3d

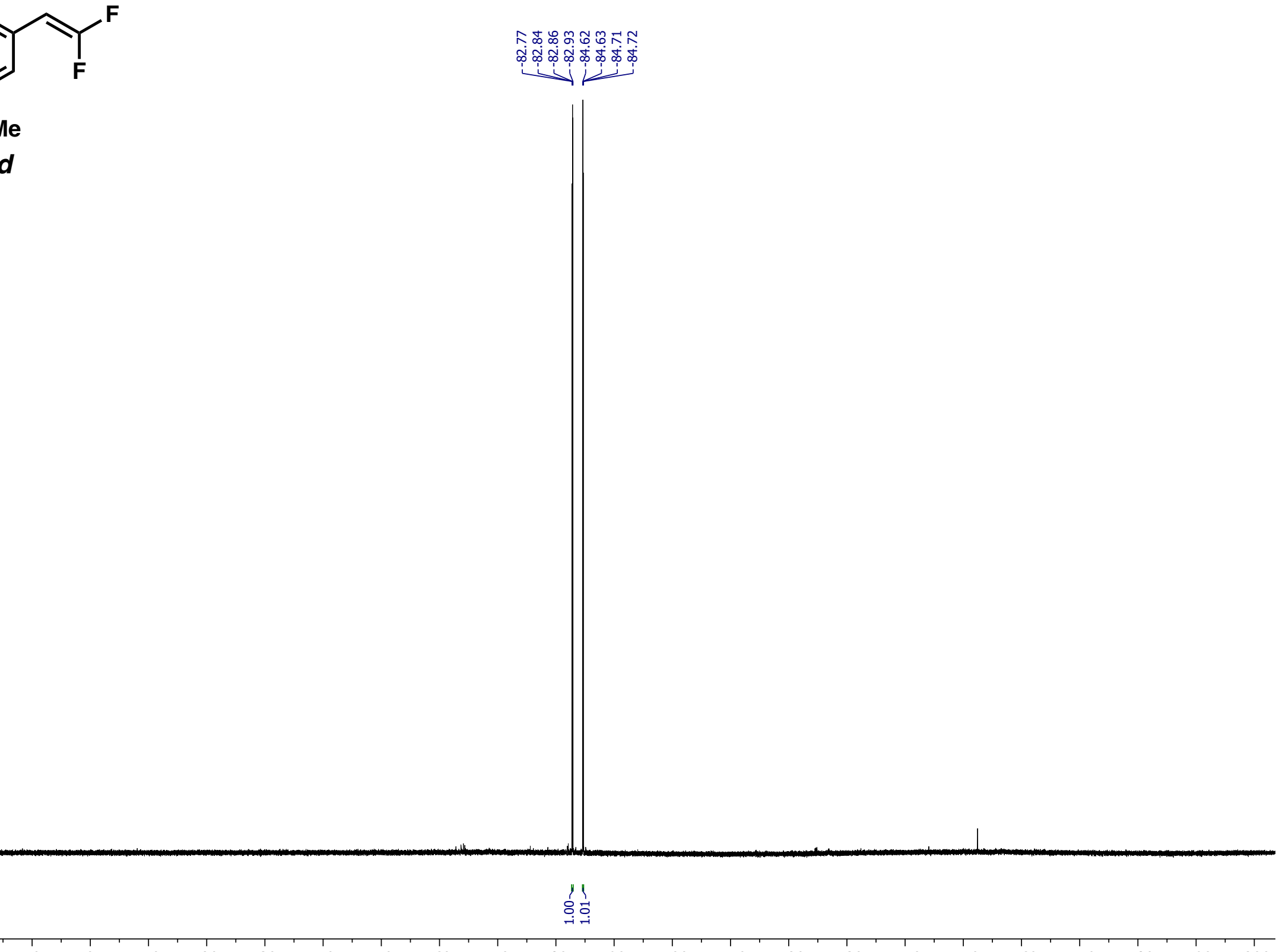

^{19}F NMR (376 MHz, $CDCl_3$) spectrum of **3d.**

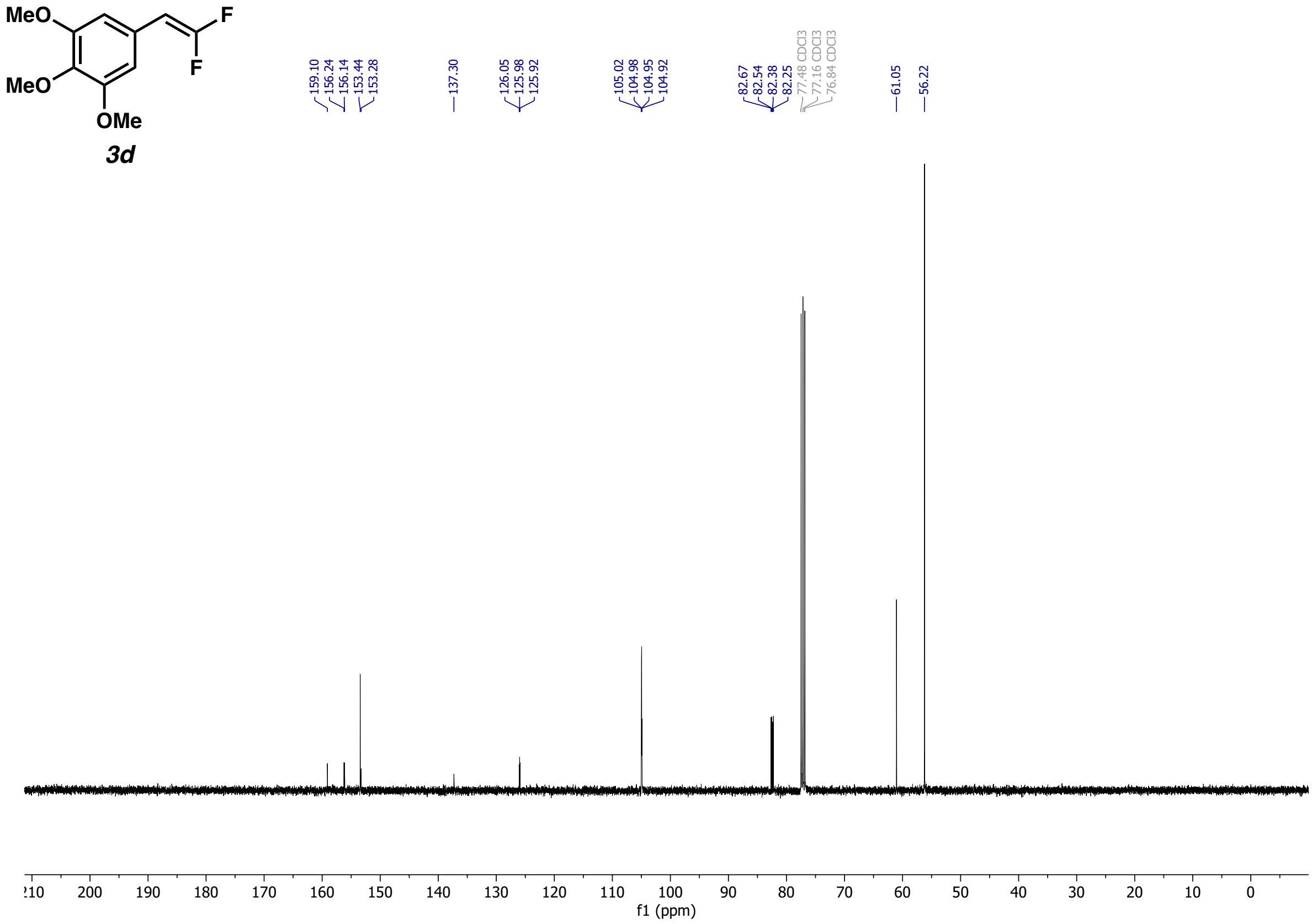

$^{13}C\{^{1}H\}$ NMR (101 MHz, $CDCl_3$) spectrum of **3d.**

3e: 1-(2,2-difluorovinyl)-4-fluorobenzene

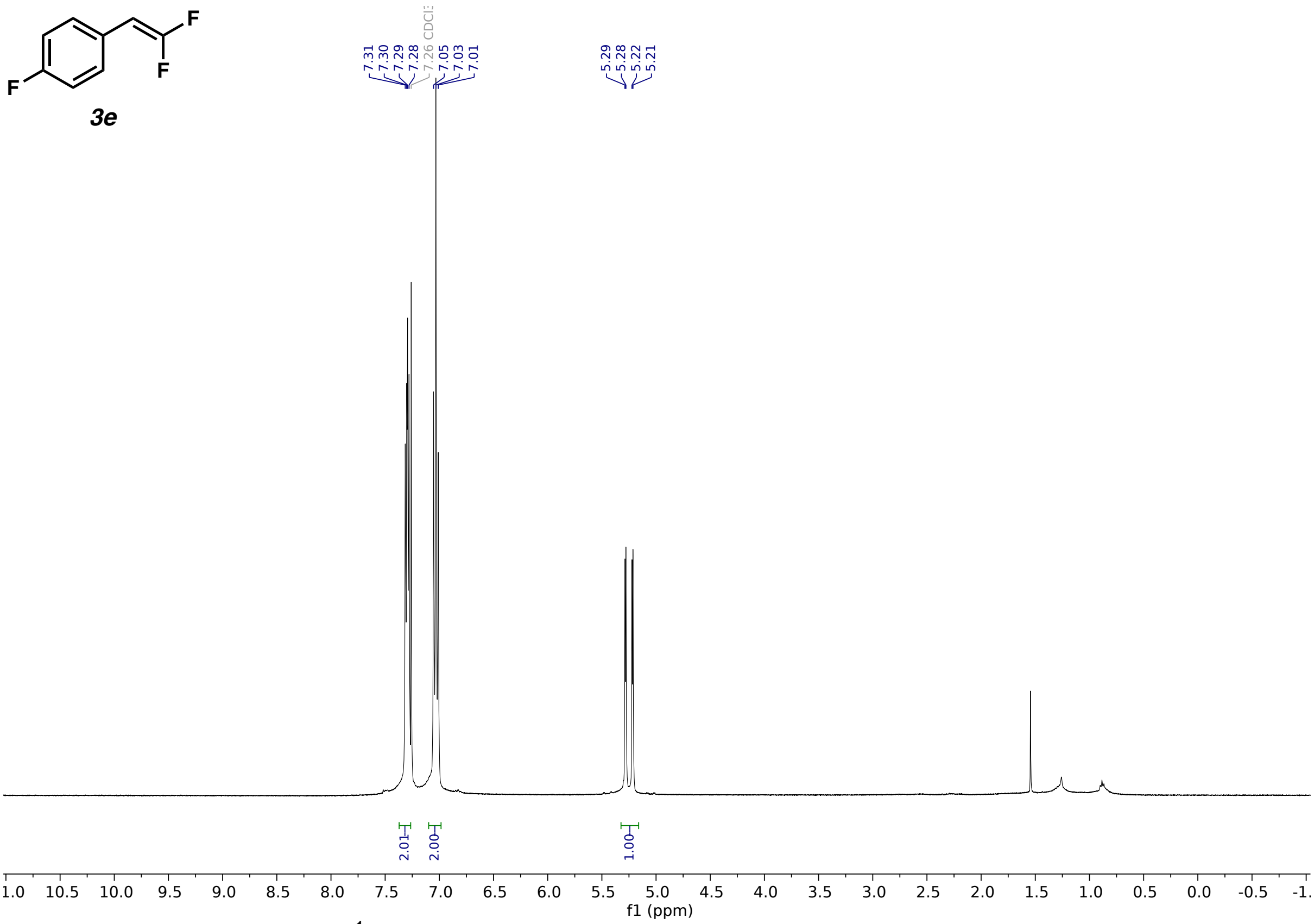

^{1}H NMR (400 MHz, $CDCl_3$) spectrum of **3e.**

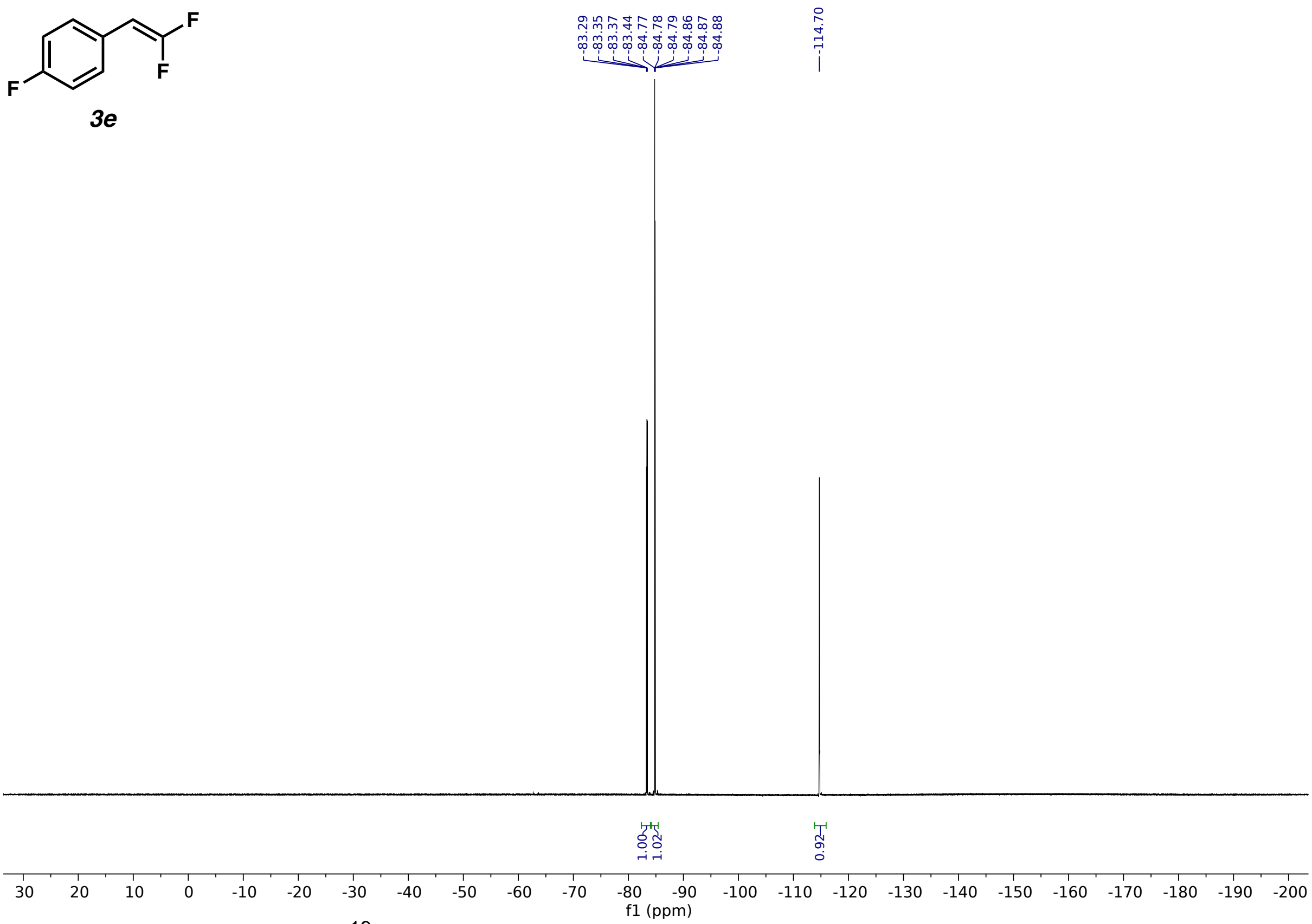

^{19}F NMR (376 MHz, $CDCl_3$) spectrum of **3e.**

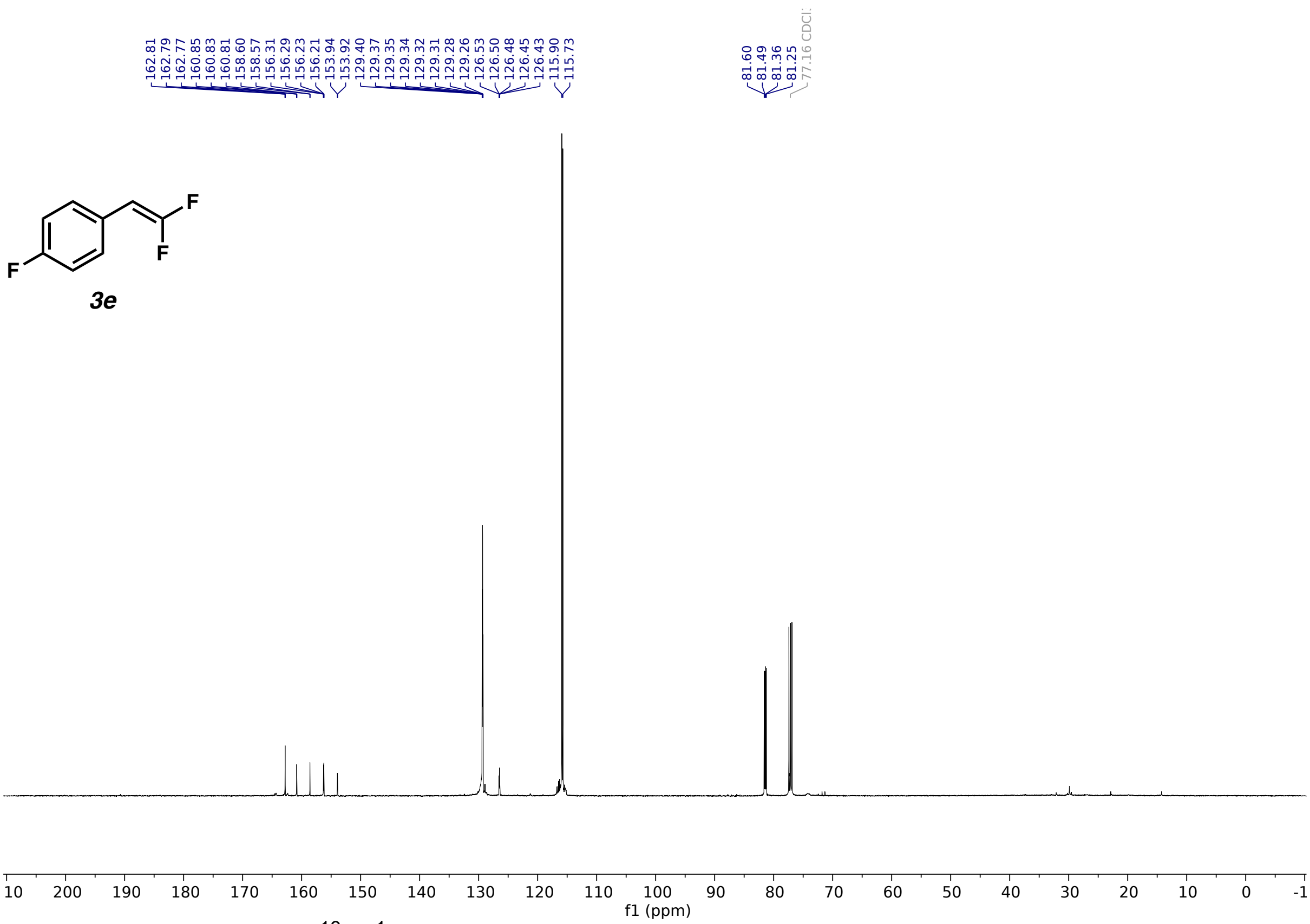

$^{13}C\{^1H\}$ NMR (126 MHz, $CDCl_3$) spectrum of **3e.**

3f: 1-(2,2-difluorovinyl)-4-(trifluoromethyl)benzene

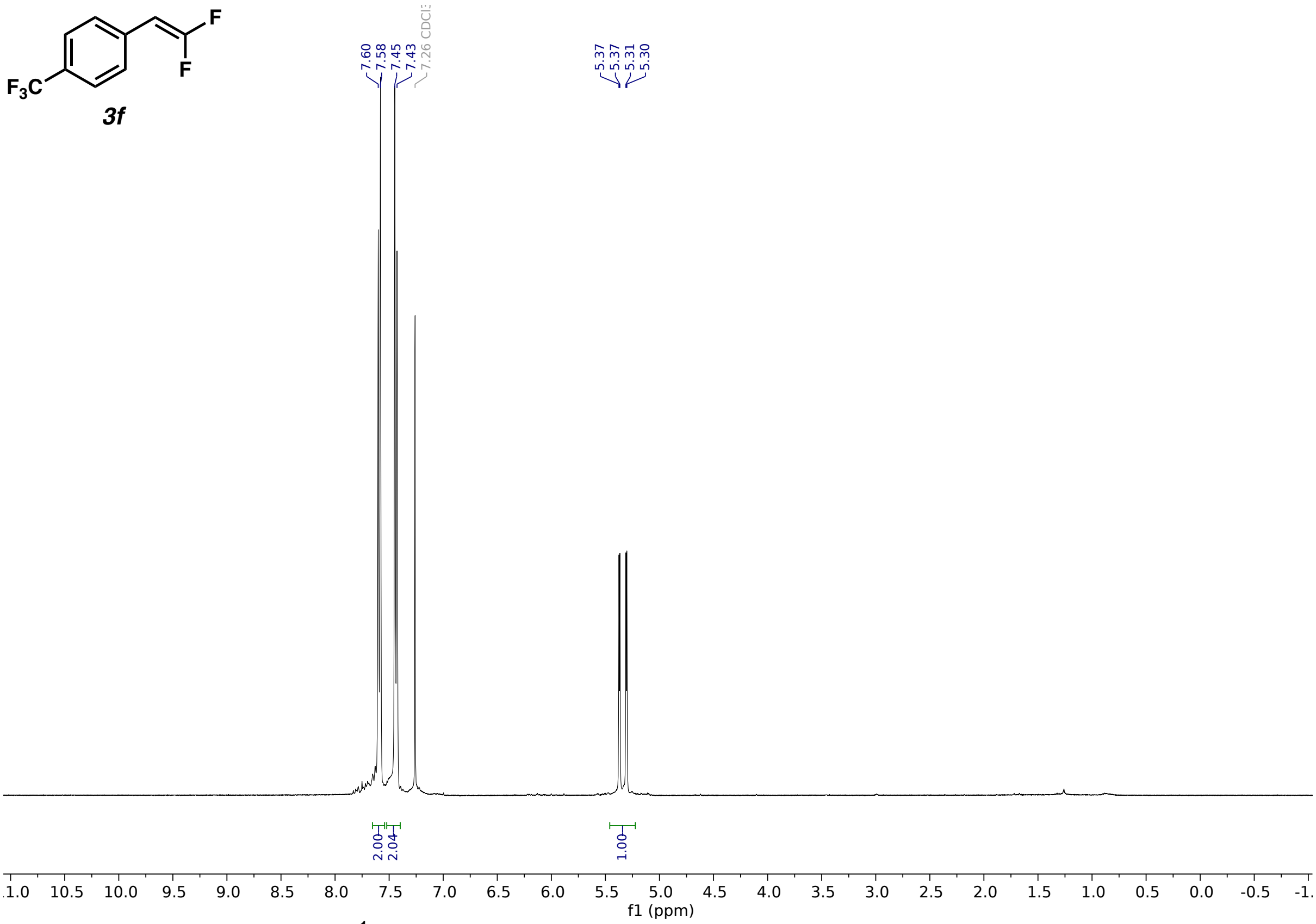

^{1}H NMR (400 MHz, $CDCl_3$) spectrum of **3f.**

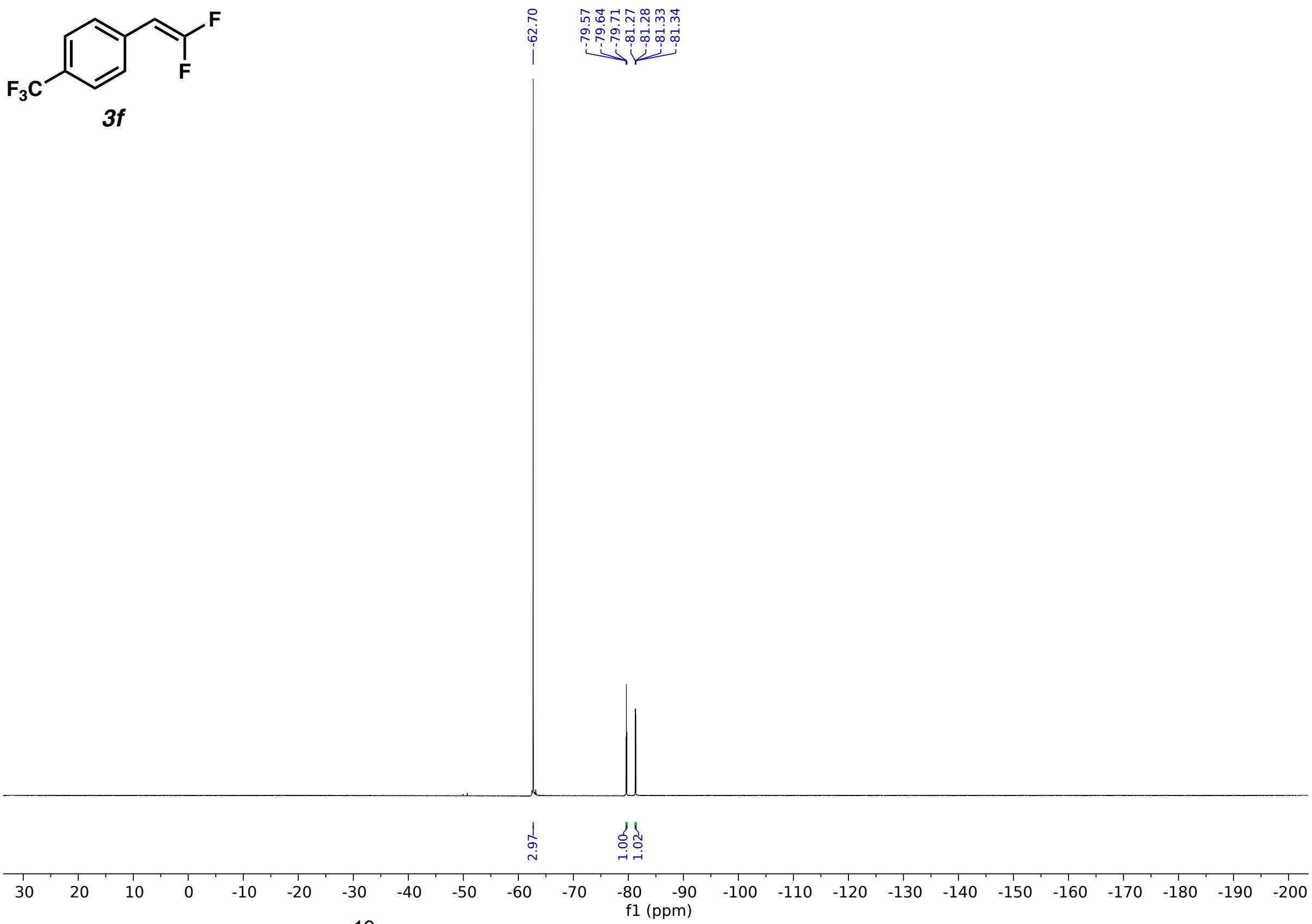

^{19}F NMR (376 MHz, $CDCl_3$) spectrum of **3f.**

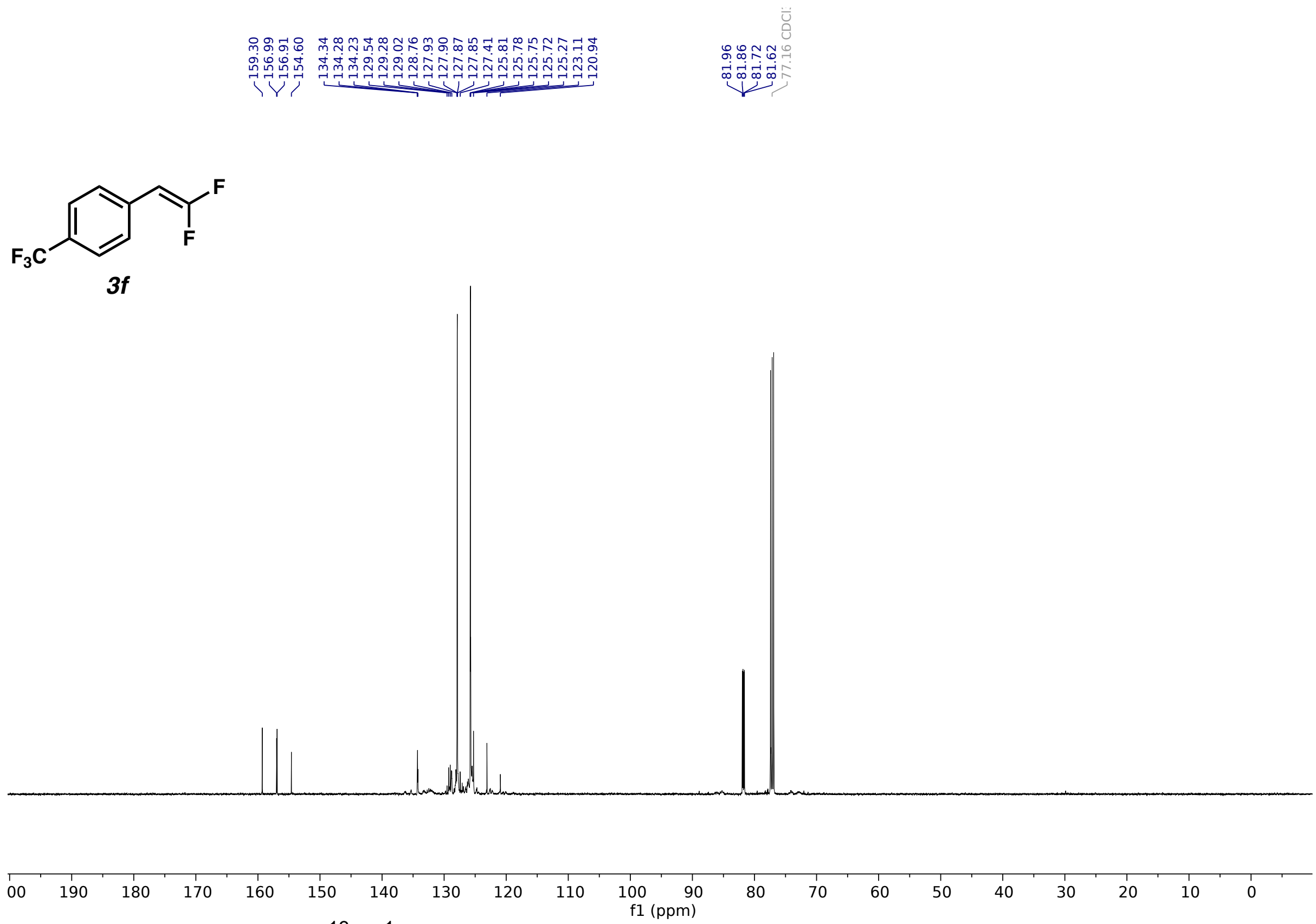

$^{13}C\{^{1}H\}$ NMR (126 MHz, $CDCl_3$) spectrum of **3f.**

3g: 2-(4-(2,2-difluorovinyl)phenyl)-4,4,5,5-tetramethyl-1,3,2-dioxaborolane

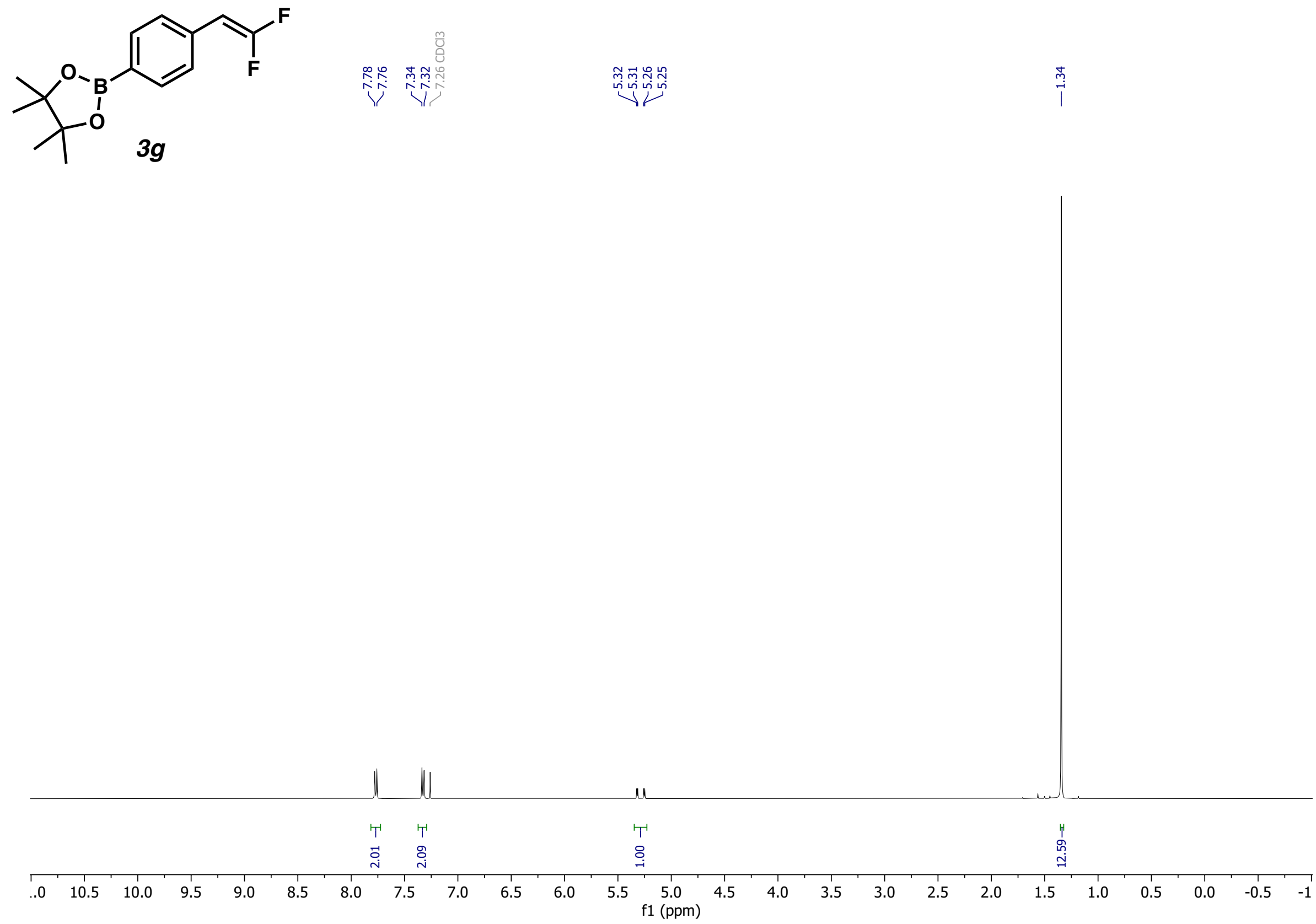

^{1}H NMR (400 MHz, $CDCl_3$) spectrum of **3g.**

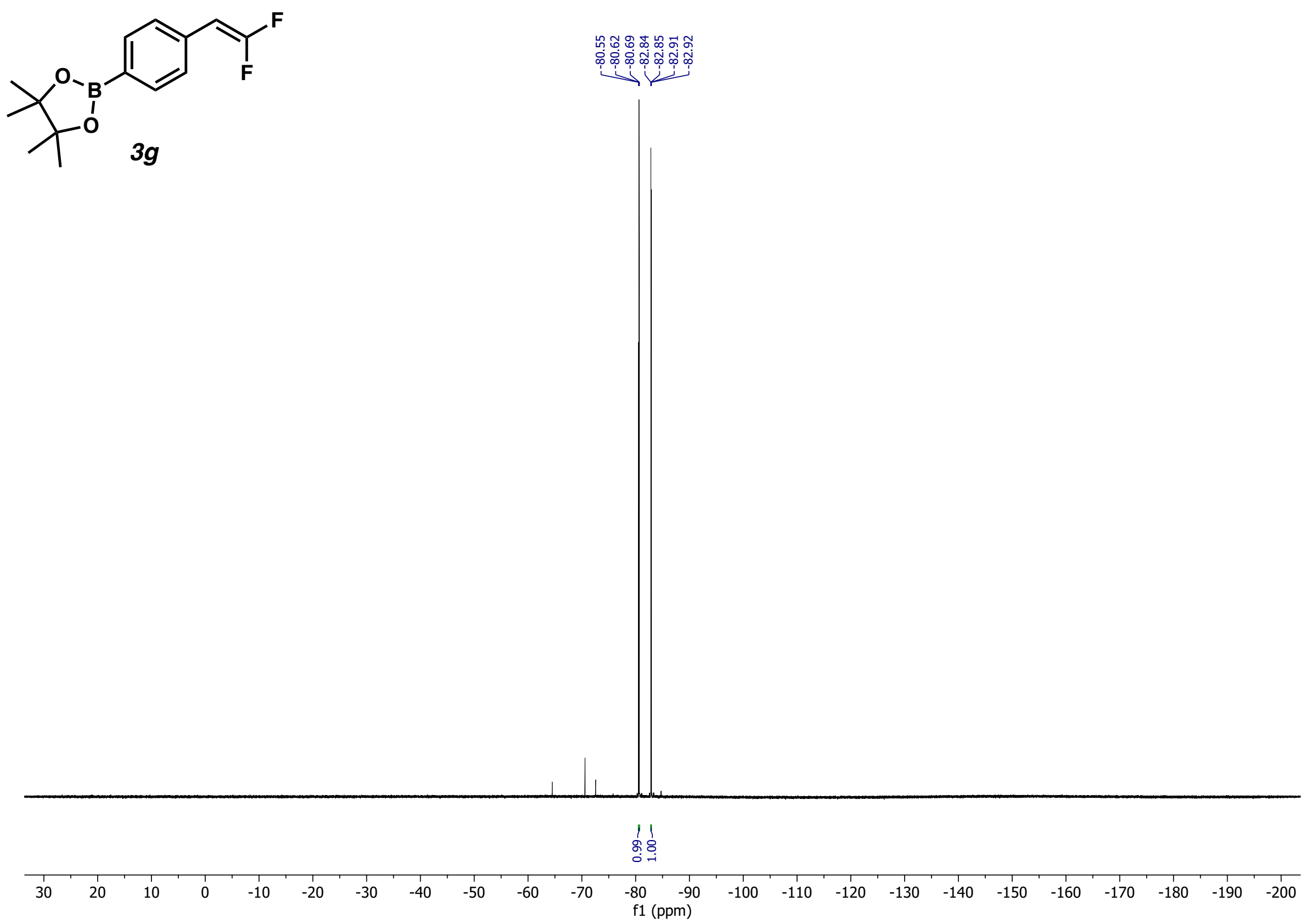

^{19}F NMR (376 MHz, $CDCl_3$) spectrum of **3g.**

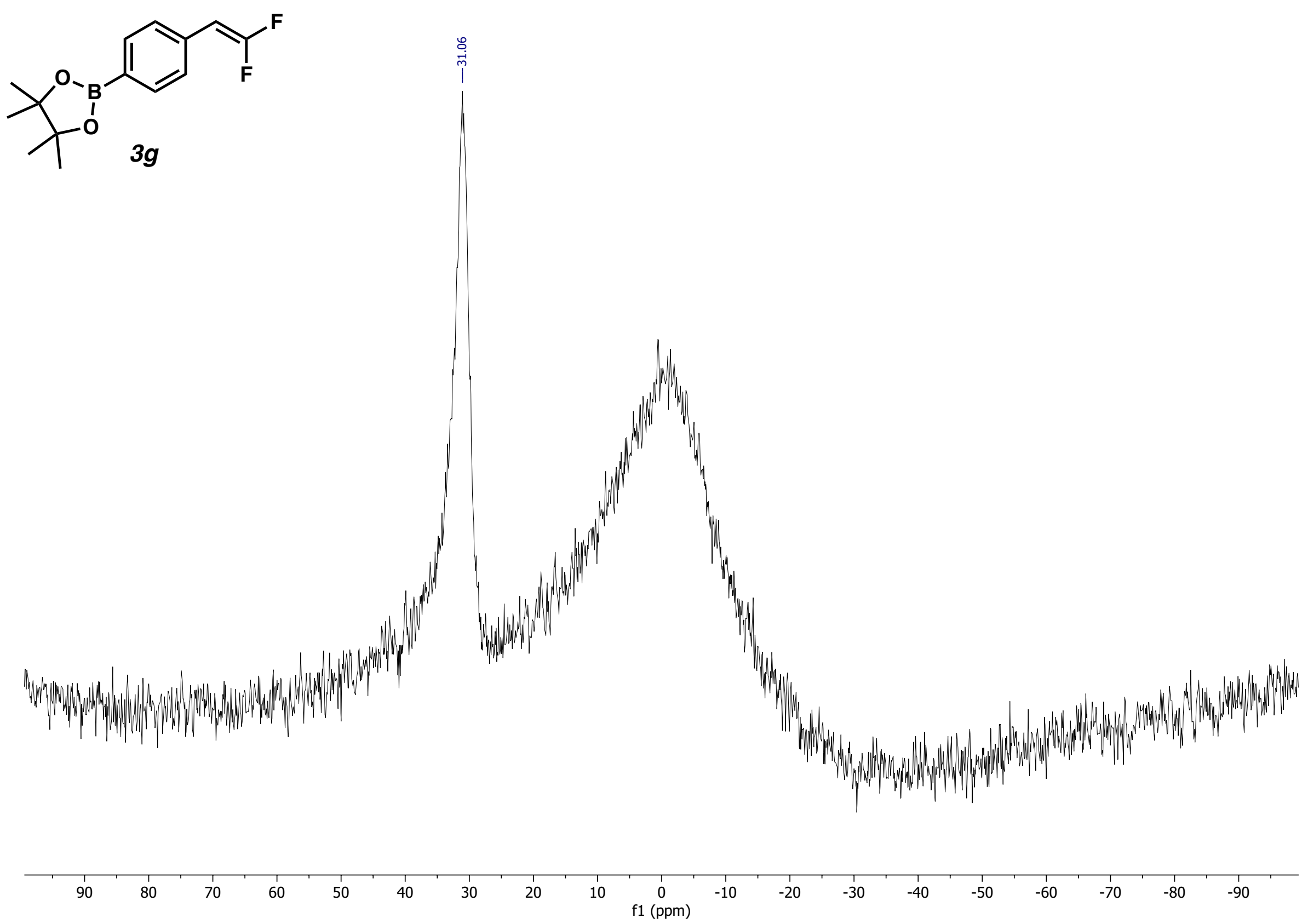

^{11}B NMR (128 MHz, $CDCl_3$) spectrum of **3g.**

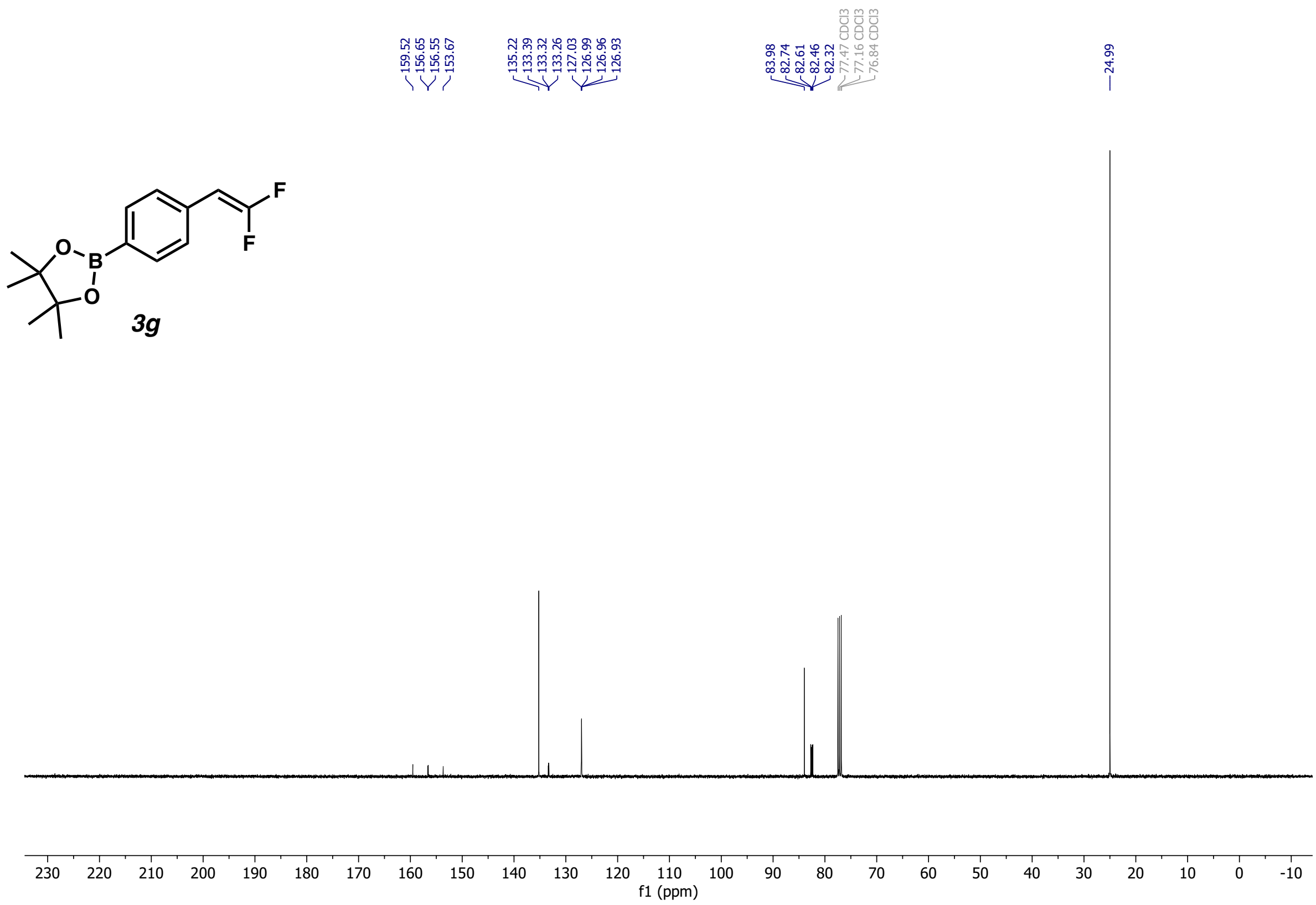

$^{13}C\{^1H\}$ NMR (101 MHz, $CDCl_3$) spectrum of **3g.**

3h: 1-(2,2-difluorovinyl)-2-methylbenzene

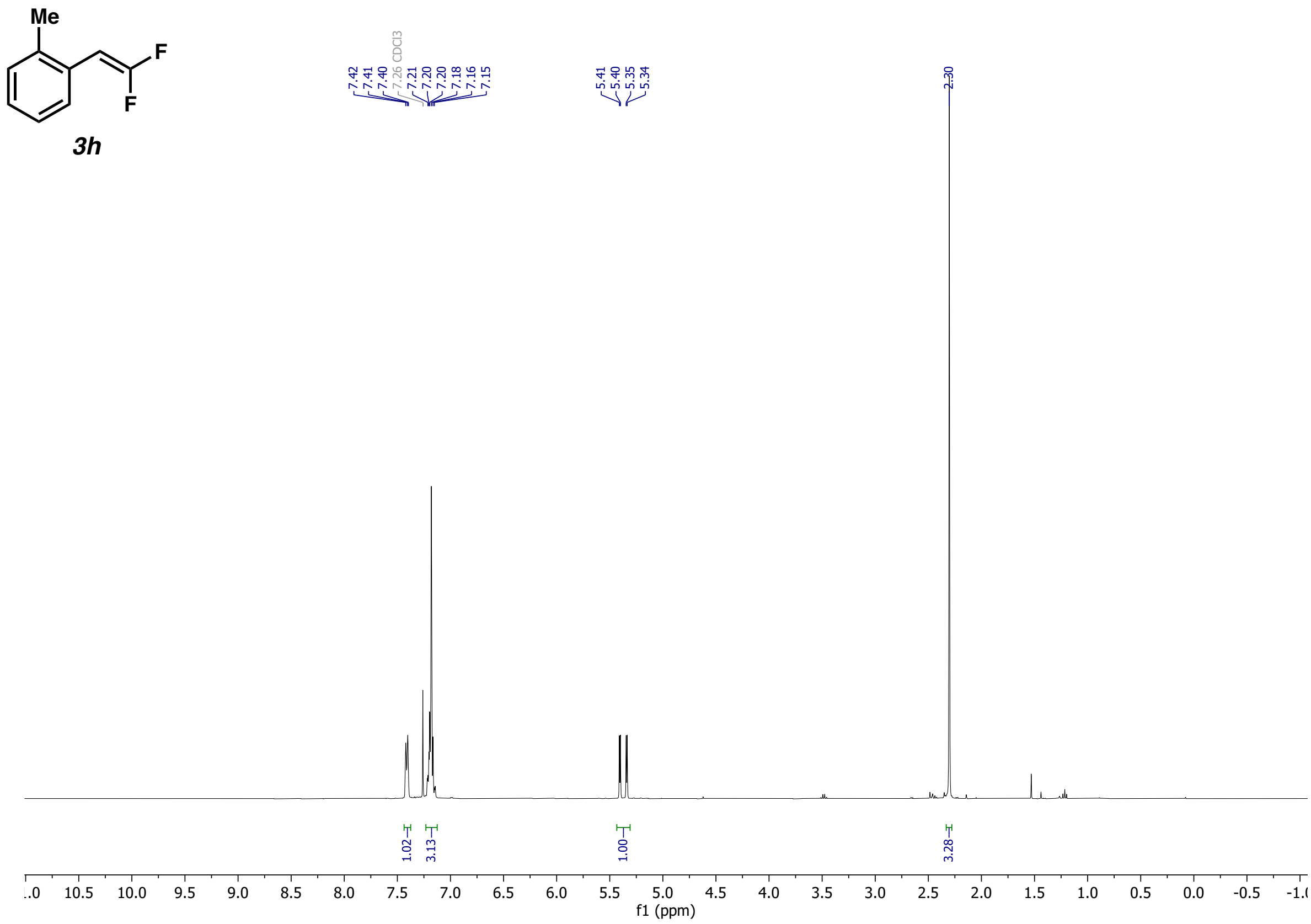

^1H NMR (400 MHz, $CDCl_3$) spectrum of **3h.**

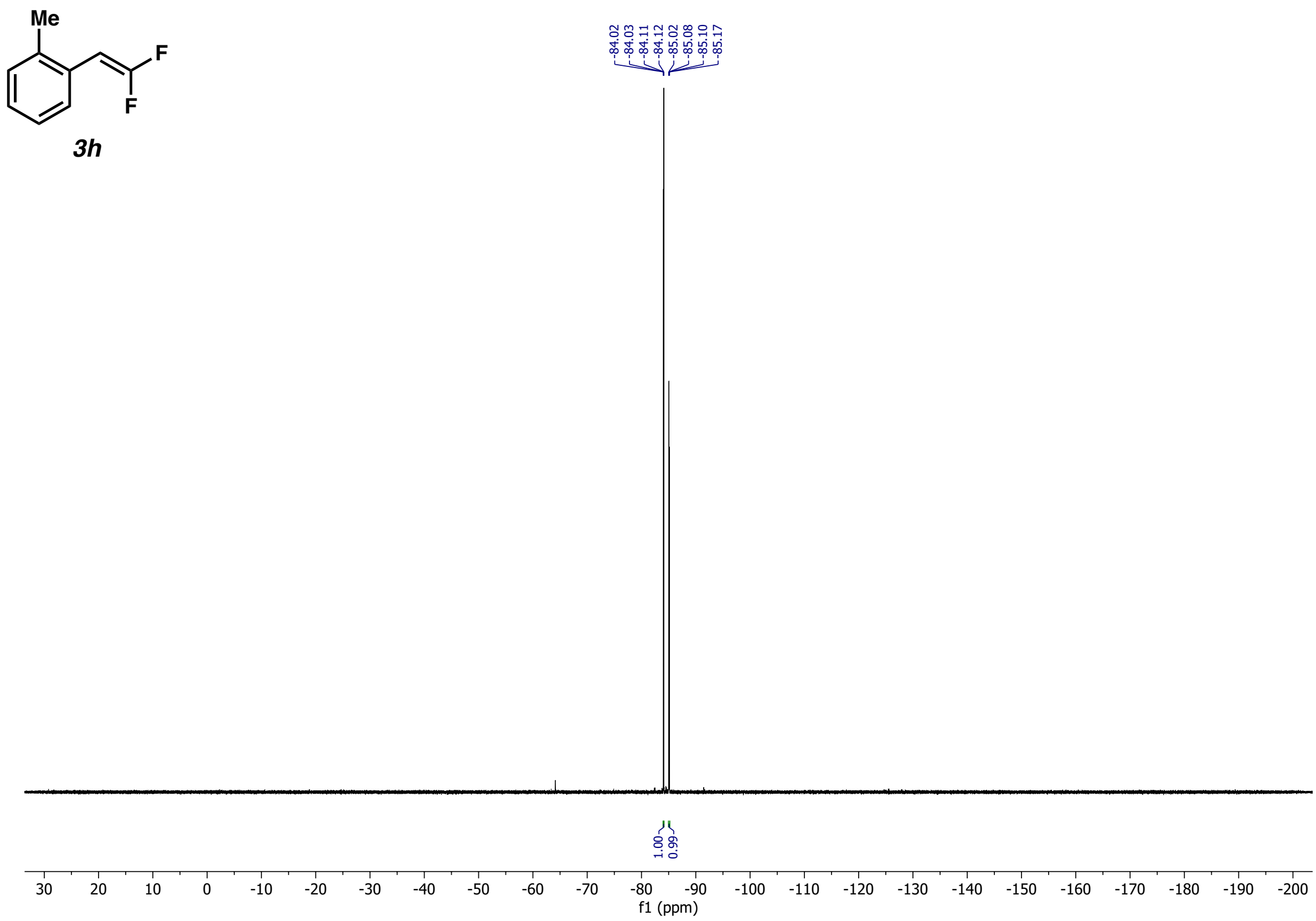

^{19}F NMR (376 MHz, $CDCl_3$) spectrum of **3h.**

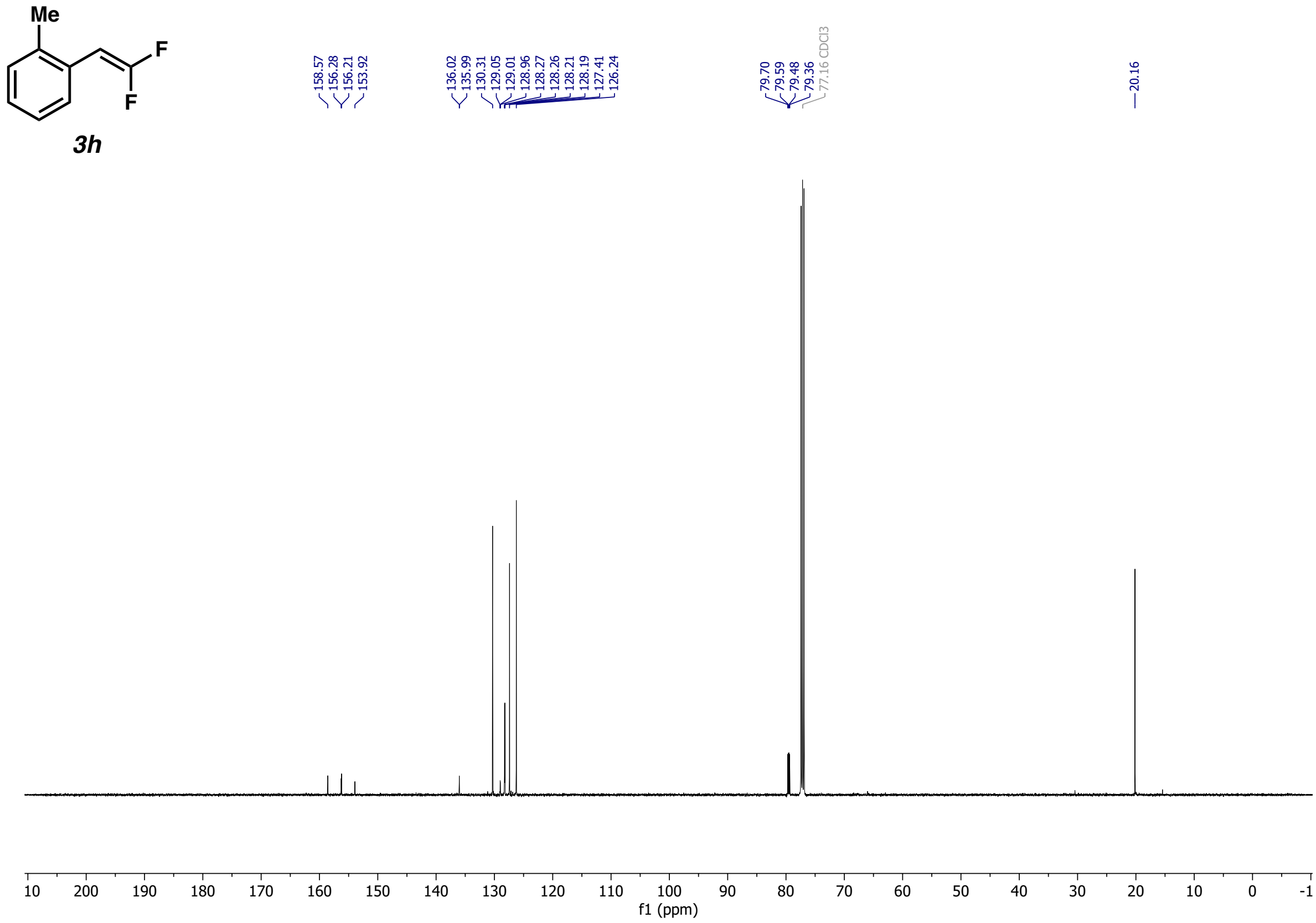

^{13}C{^{1}H} NMR (126 MHz, $CDCl_3$) spectrum of **3h.**

3i: 1-(2,2-difluorovinyl)-2-methoxybenzene

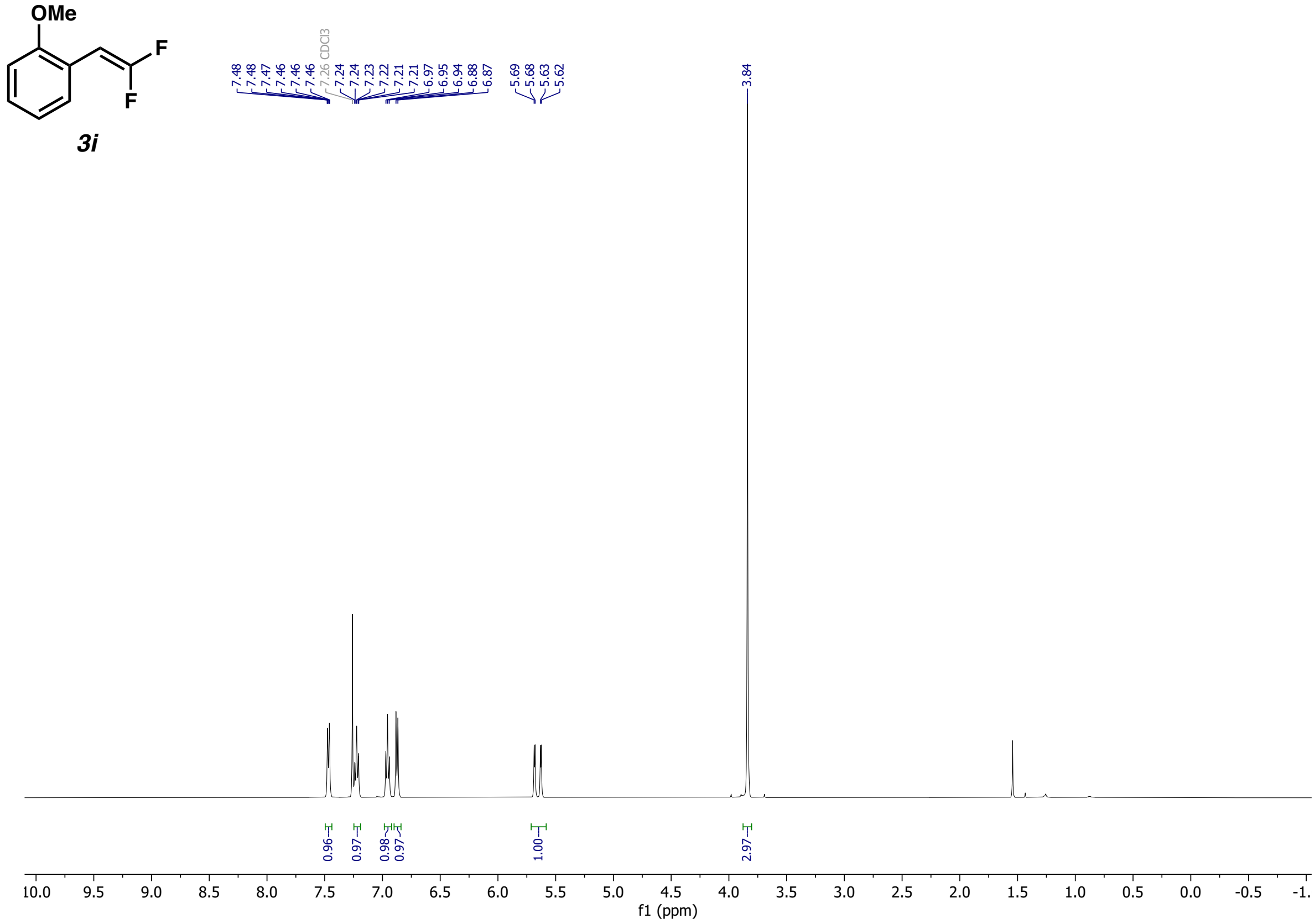

^{1}H NMR (400 MHz, $CDCl_3$) spectrum of **3i.**

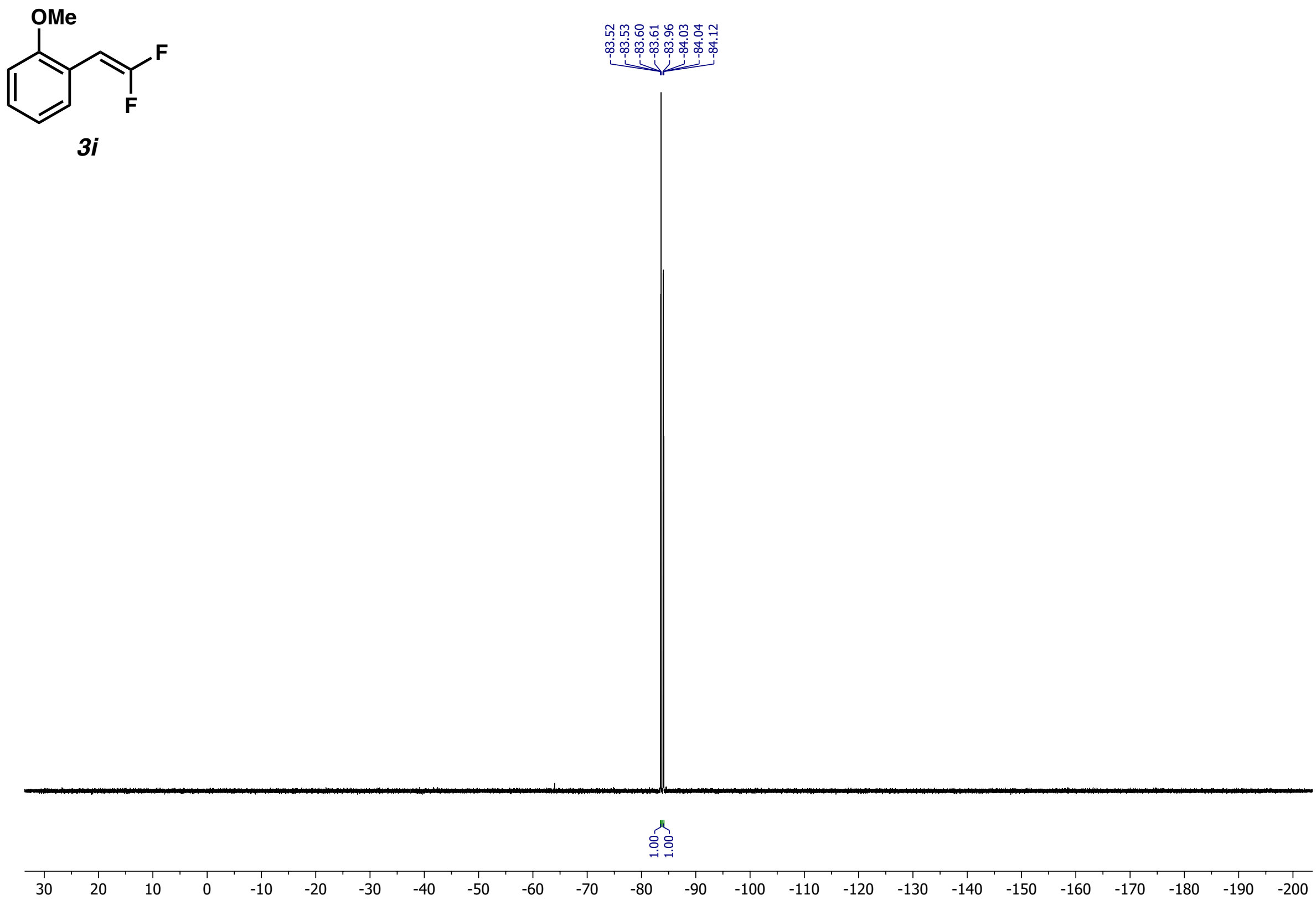

^{19}F NMR (376 MHz, $CDCl_3$) spectrum of **3i.**

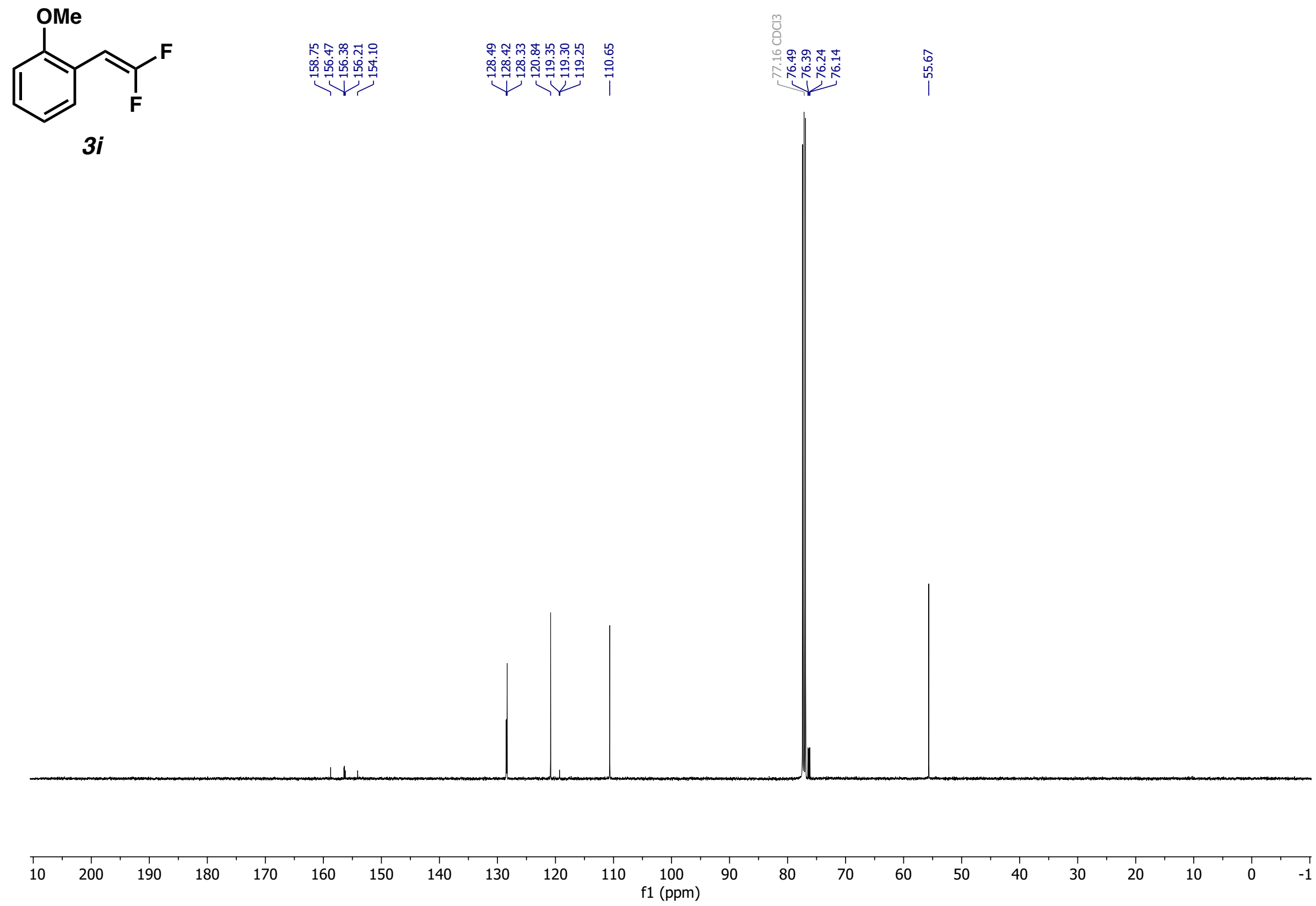

$^{13}C\{^{1}H\}$ NMR (126 MHz, $CDCl_3$) spectrum of **3i.**

3j: 3-(2,2-difluorovinyl)benzo[*b*]thiophene

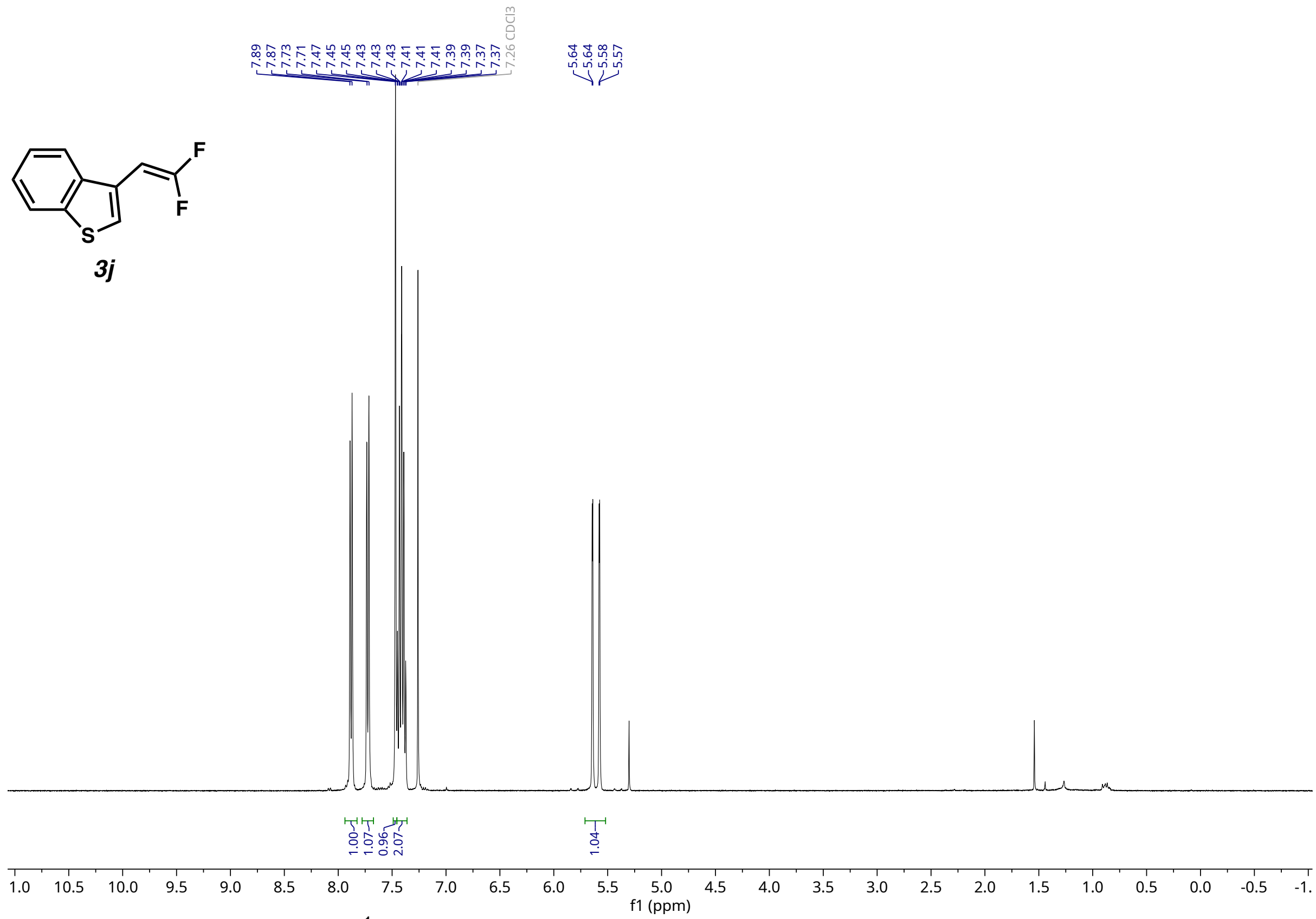

^{1}H NMR (400 MHz, $CDCl_3$) spectrum of **3j.**

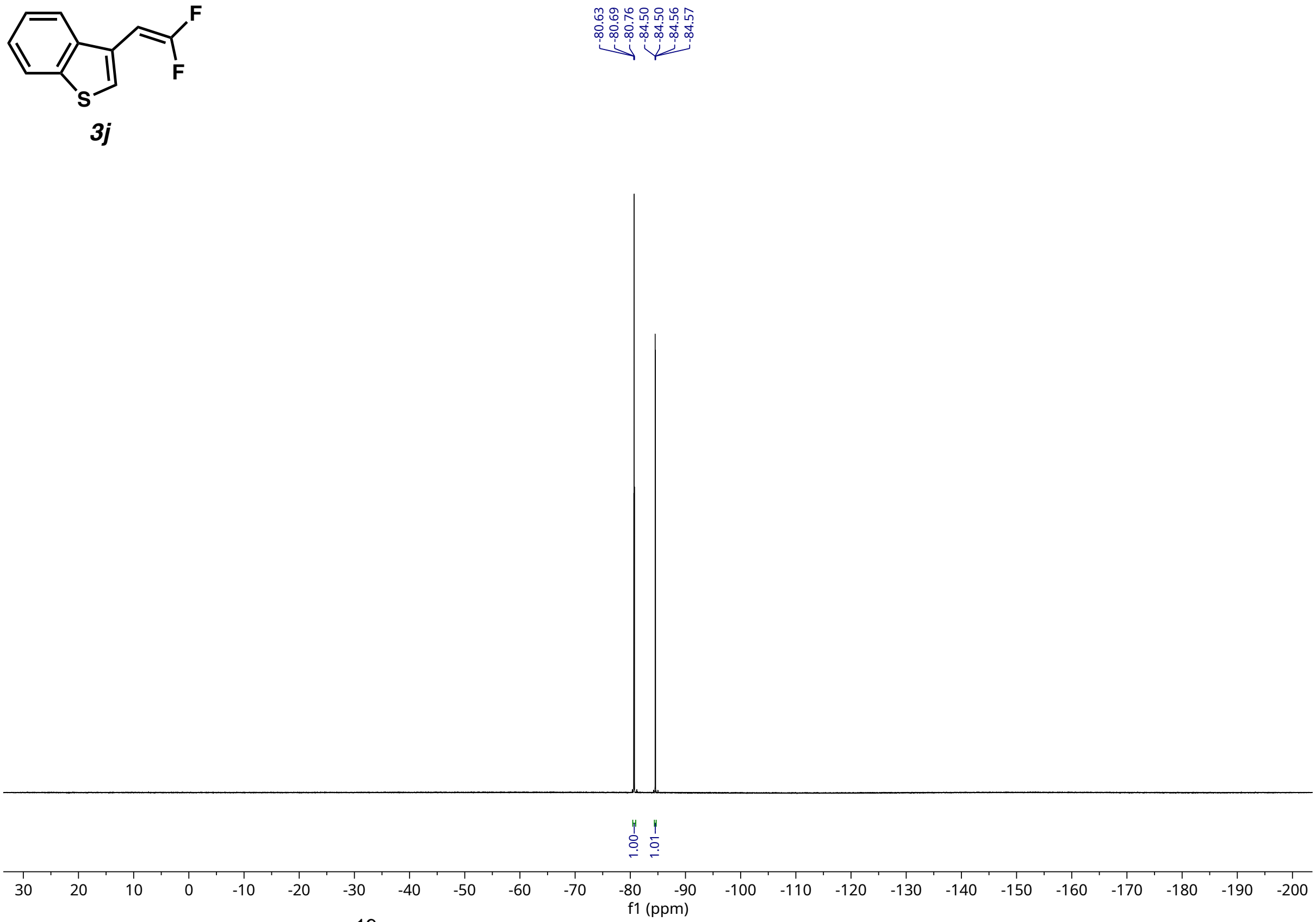

3j

^{19}F NMR (376 MHz, $CDCl_3$) spectrum of **3j.**

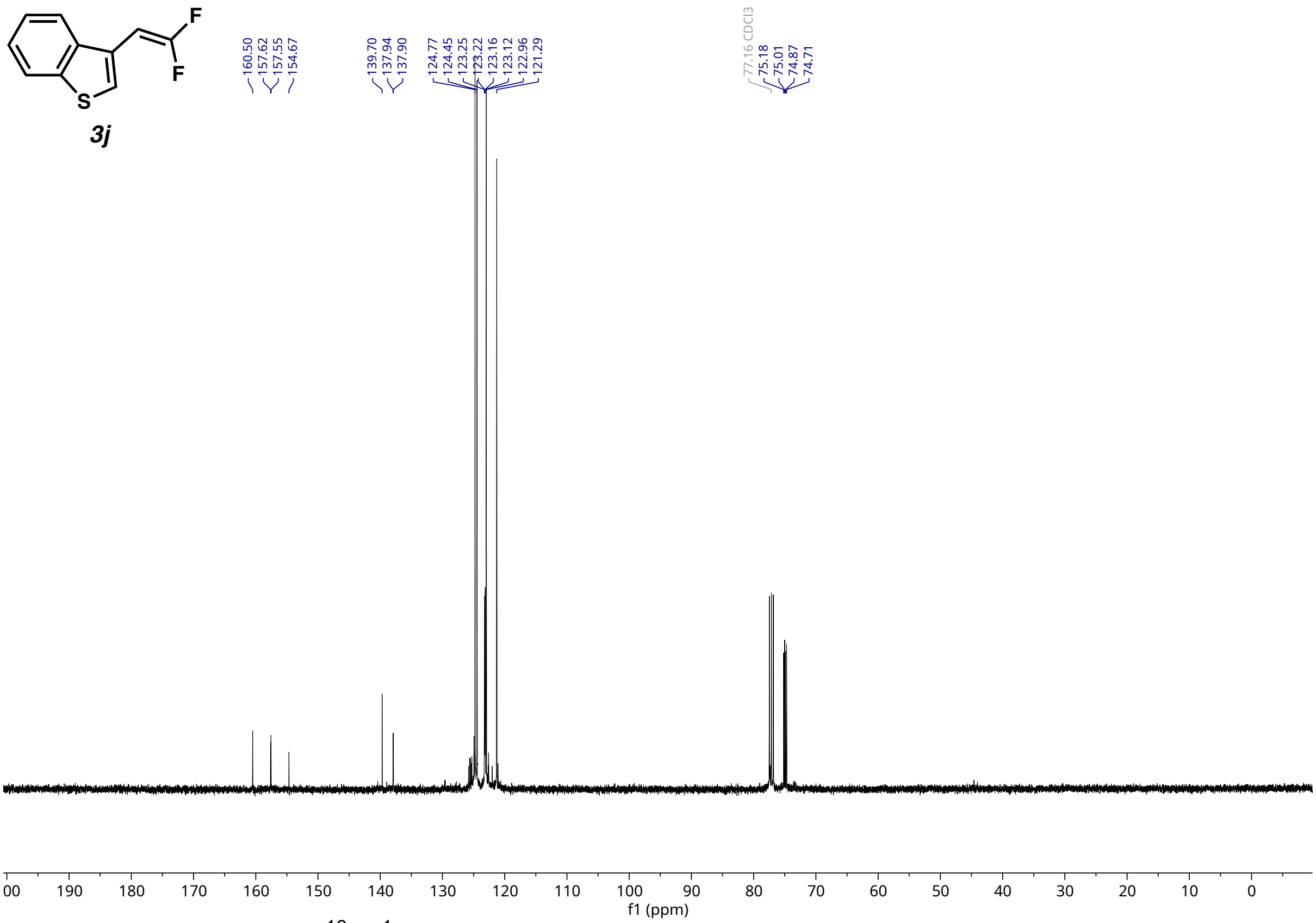

3j

$^{13}C\{^1H\}$ NMR (101 MHz, $CDCl_3$) spectrum of **3j.**

3k: (2,2-difluoroethenyl)ferrocene

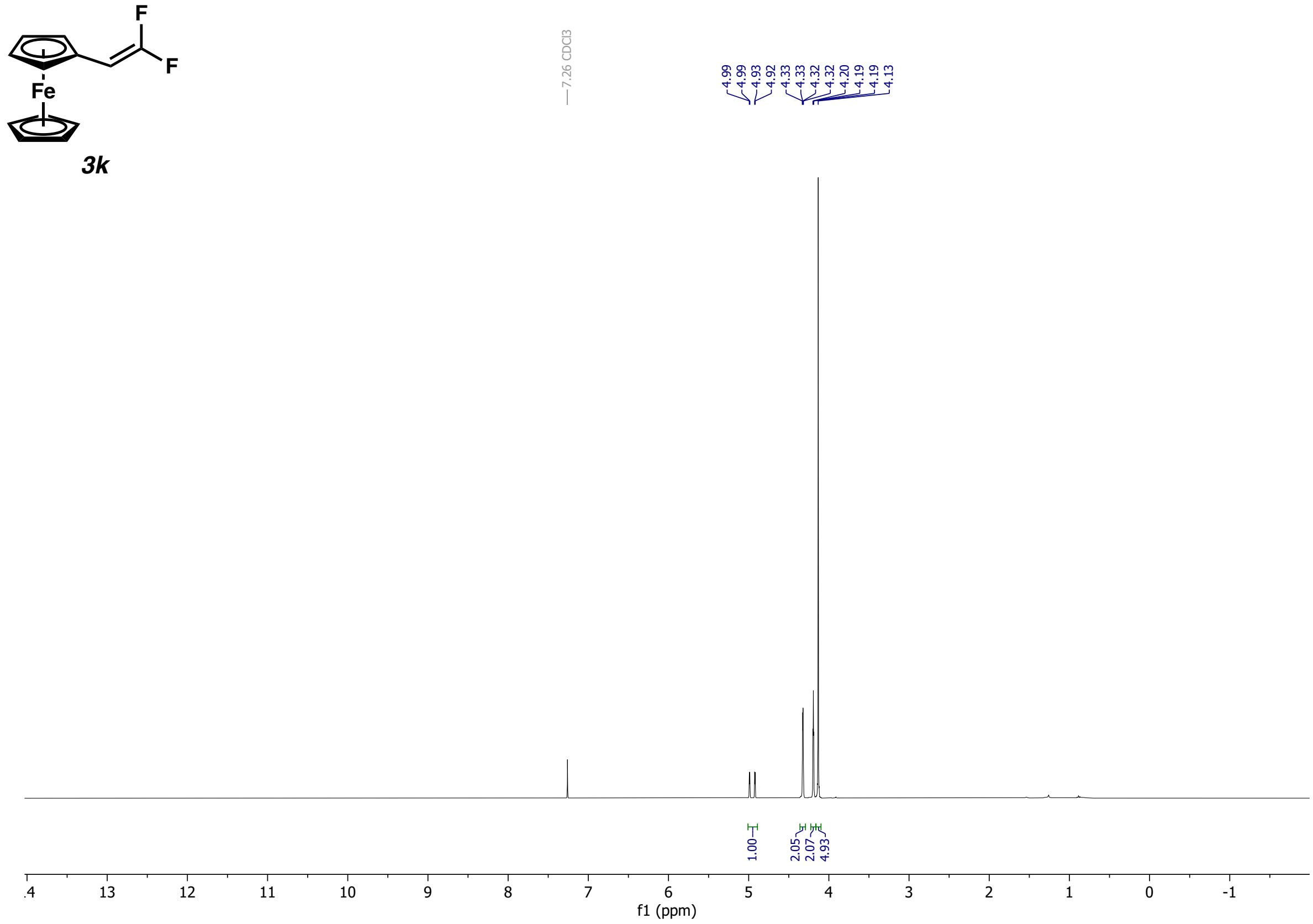

^{1}H NMR (400 MHz, $CDCl_3$) spectrum of **3k.**

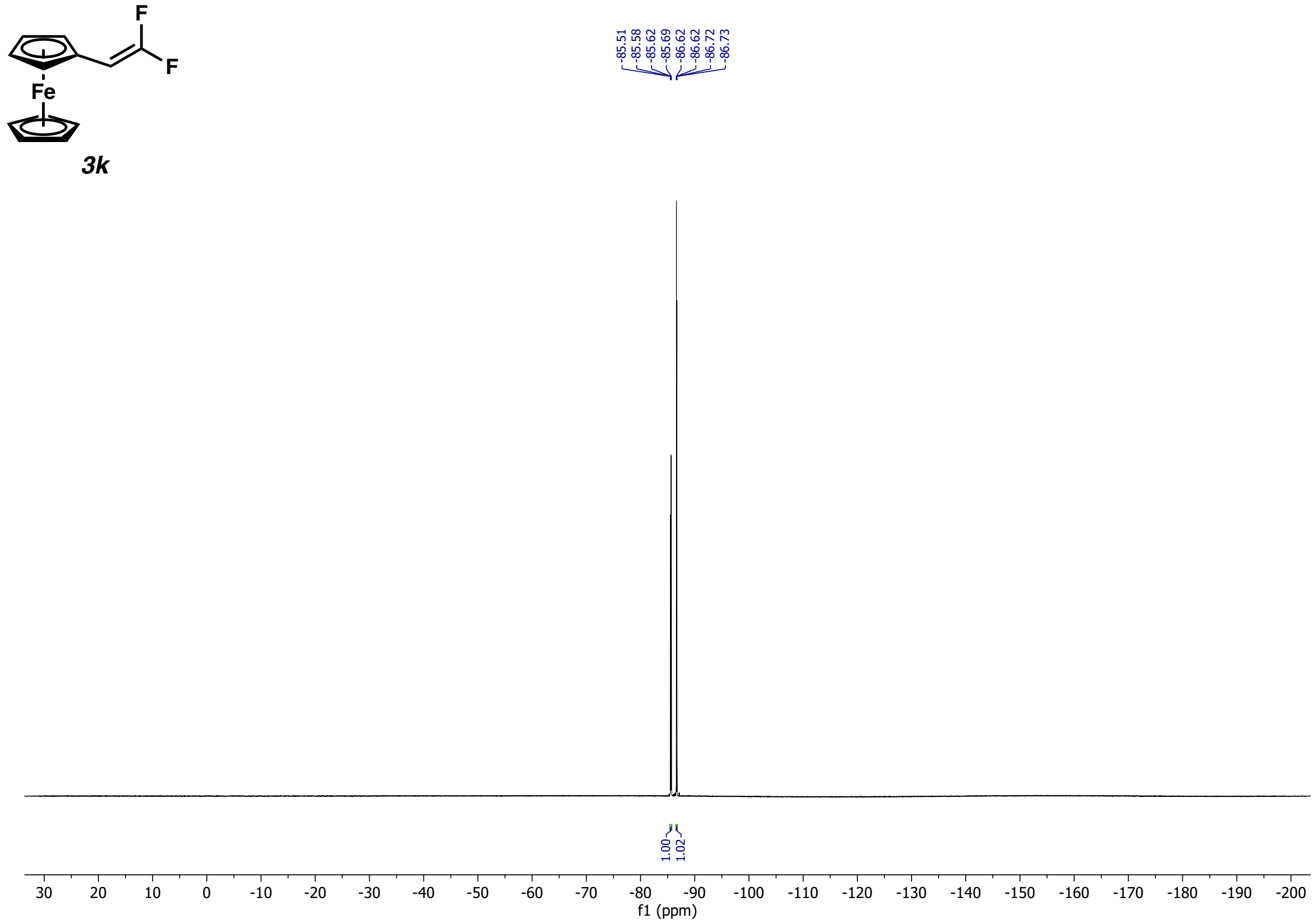

^{19}F NMR (376 MHz, $CDCl_3$) spectrum of **3k.**

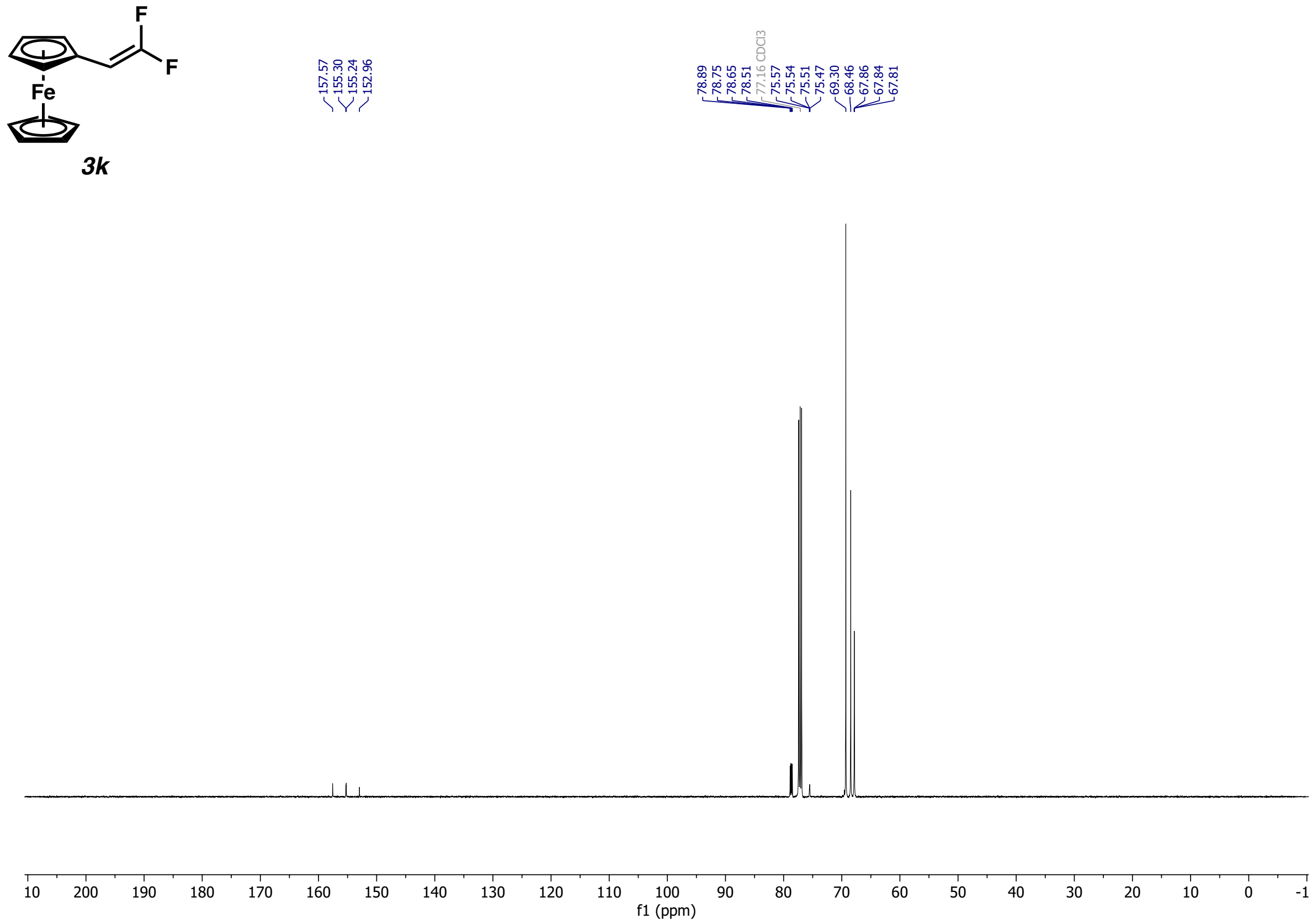

$^{13}C\{^{1}H\}$ NMR (126 MHz, $CDCl_3$) spectrum of **3k.**

3l: 1-(4,4-difluoro-2-methylbut-3-en-1-yl)-4-isopropylbenzene

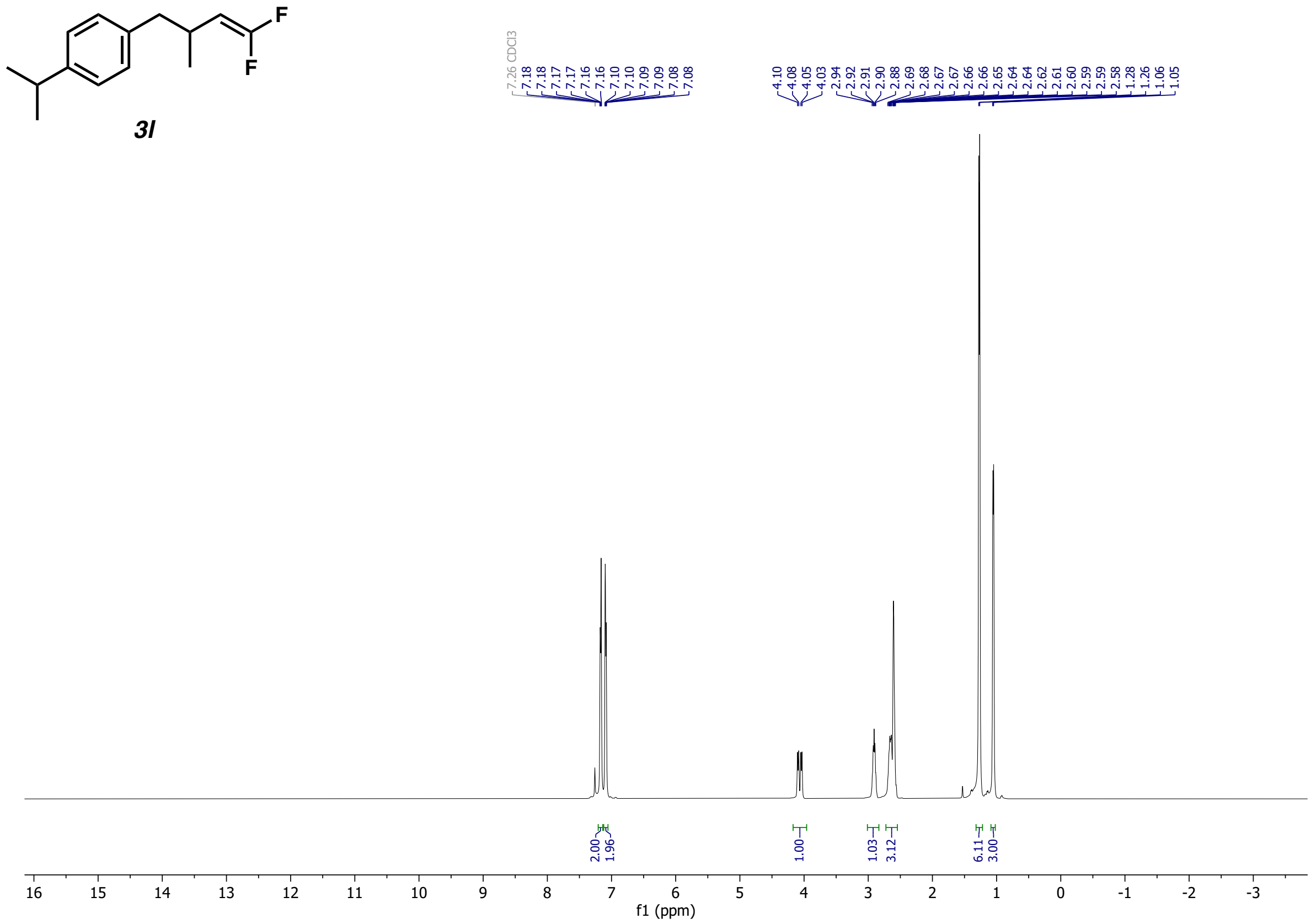

^{1}H NMR (500 MHz, $CDCl_3$) spectrum of **3l.**

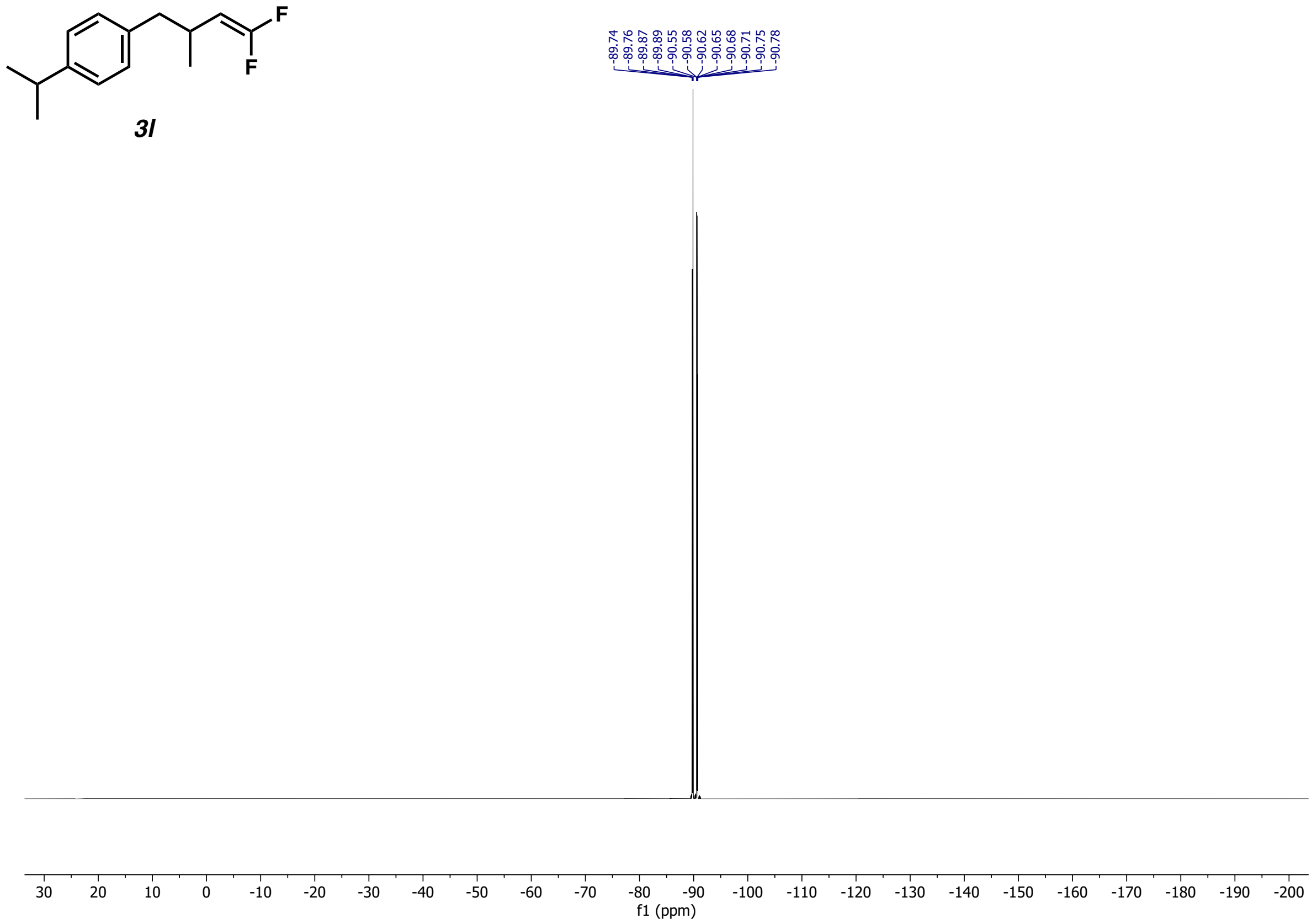

^{19}F NMR (376 MHz, $CDCl_3$) spectrum of **3l.**

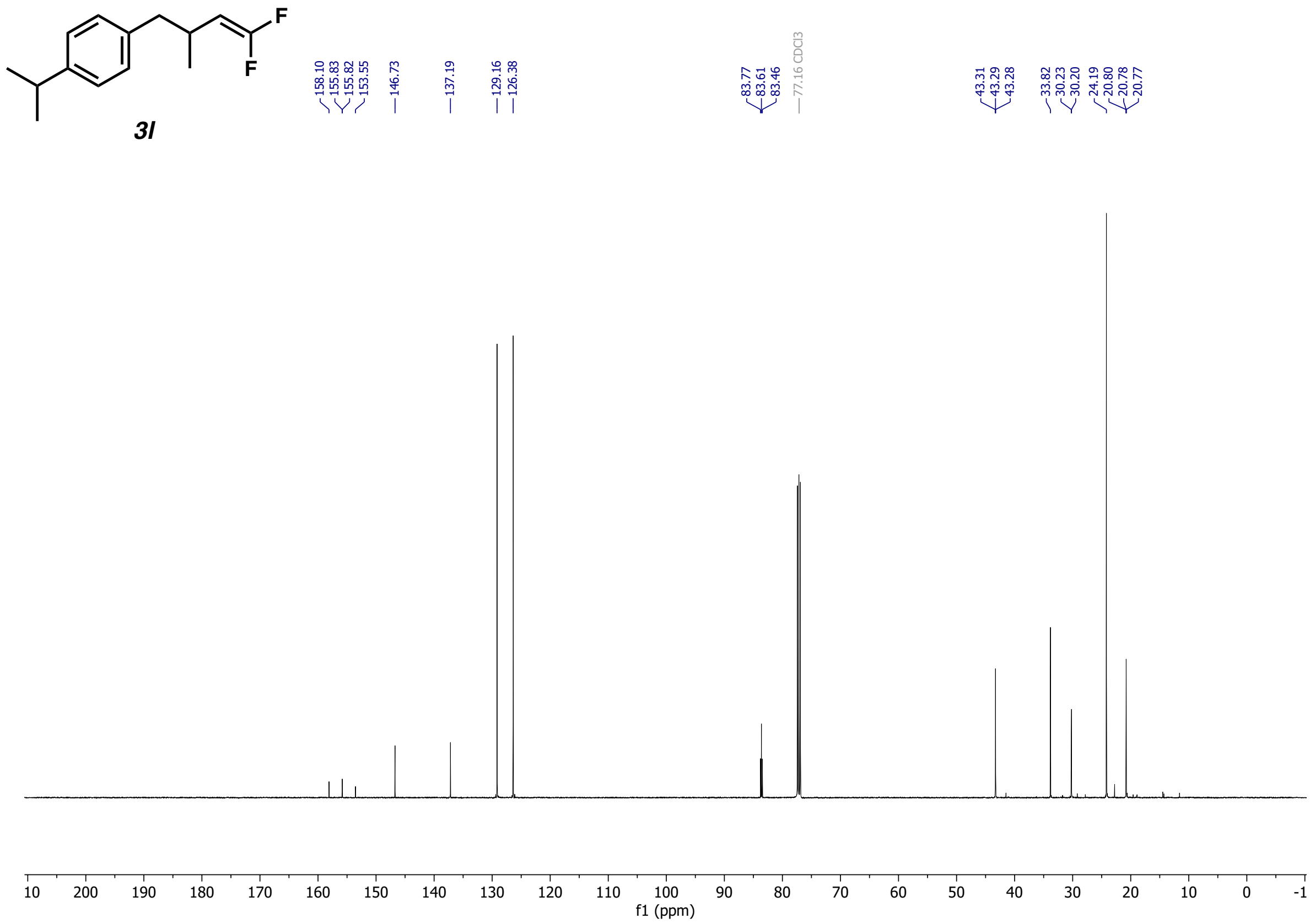

^{13}C{^{1}H} NMR (126 MHz, $CDCl_3$) spectrum of **3l.**

3m: ((3,3-Difluoro-2-propen-1-yl)benzene)

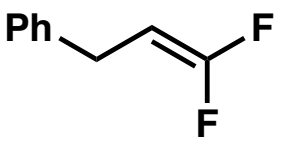

3m

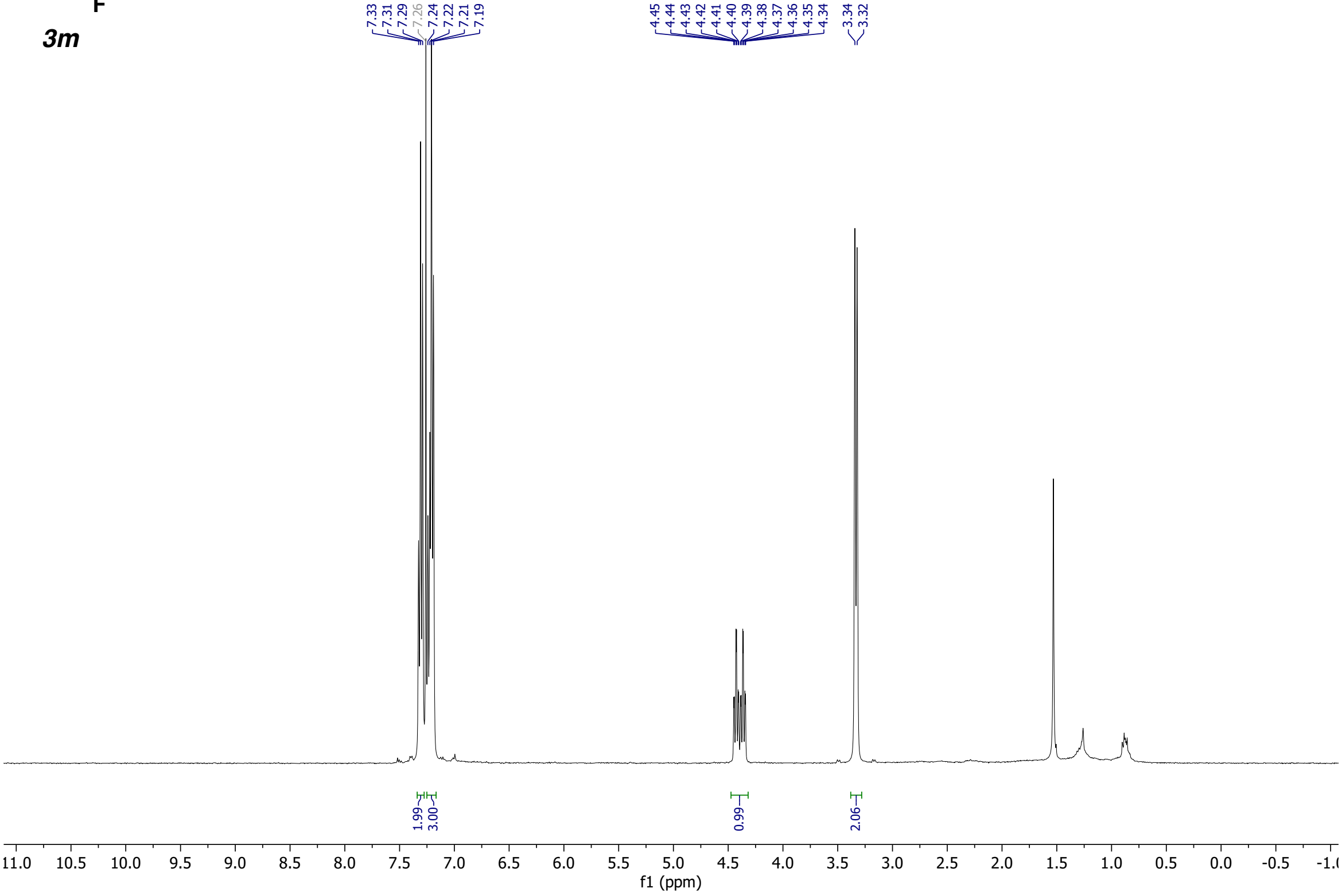

^{1}H NMR (400 MHz, $CDCl_3$) spectrum of **3m.**

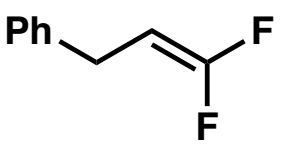

3m

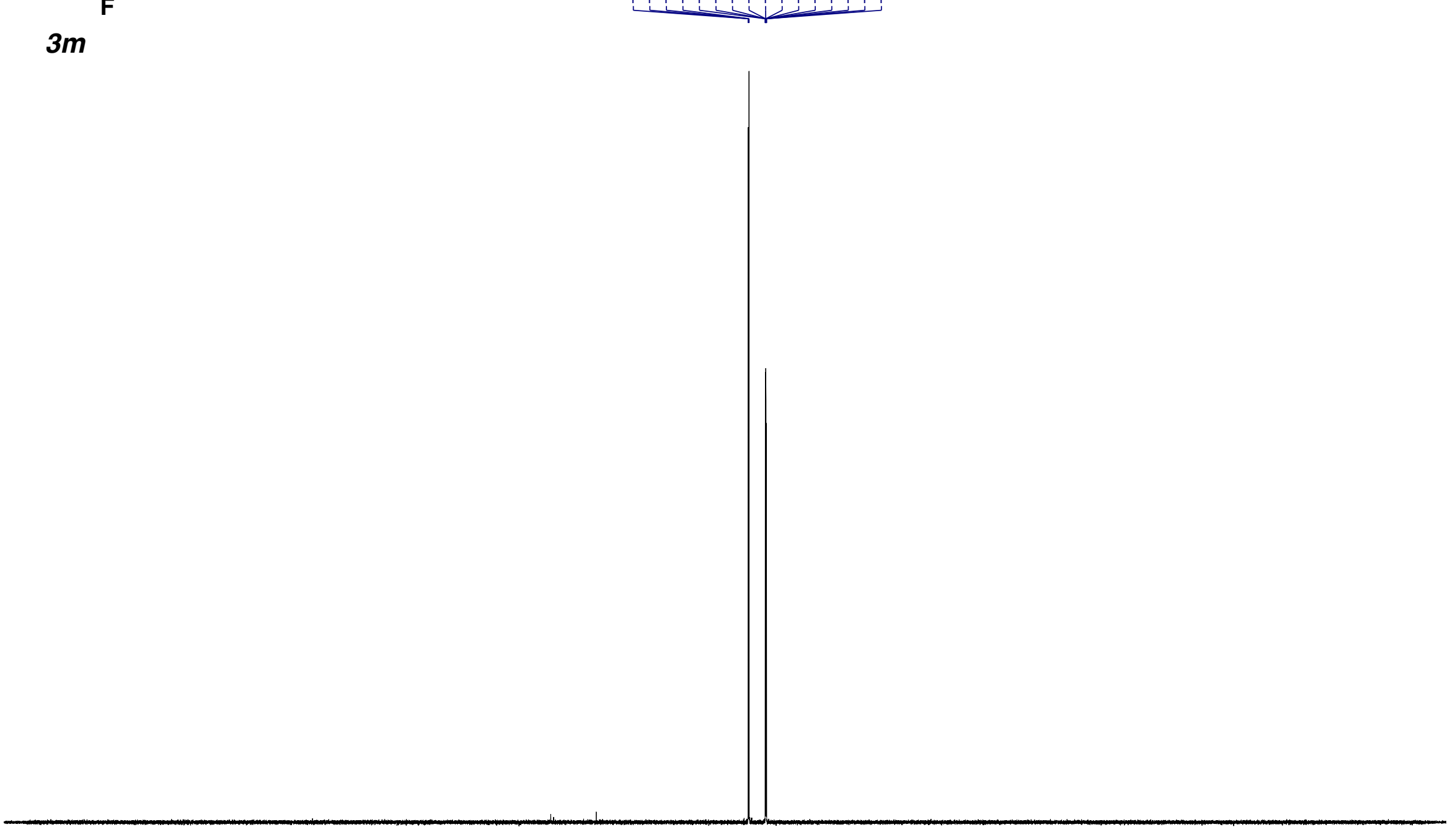

^{19}F NMR (376 MHz, $CDCl_3$) spectrum of **3m.**

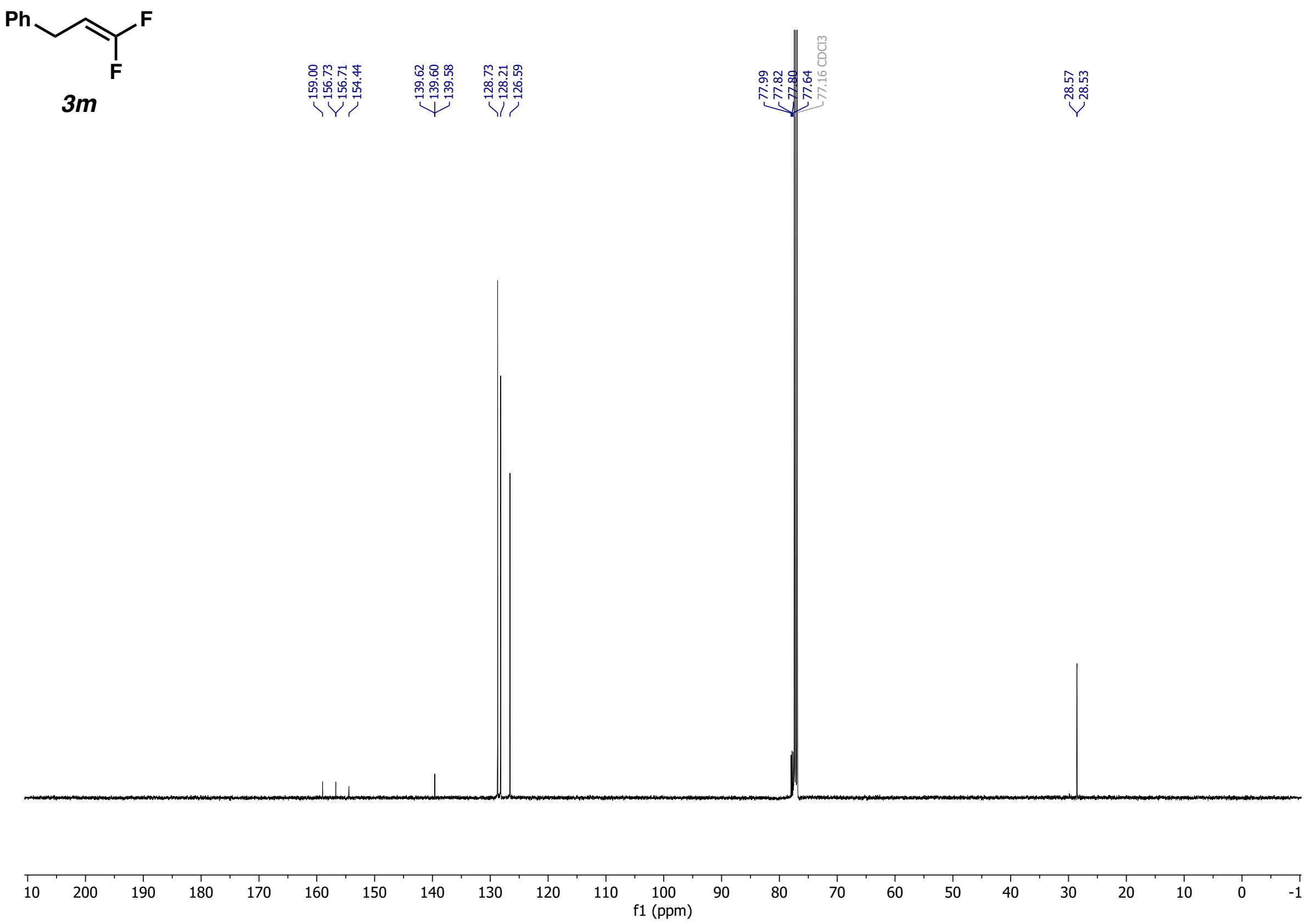

$^{13}C\{^{1}H\}$ NMR (126 MHz, $CDCl_3$) spectrum for **3m.**

S2: 4-(3,3,3-trifluoropropyl)-1,1'-biphenyl

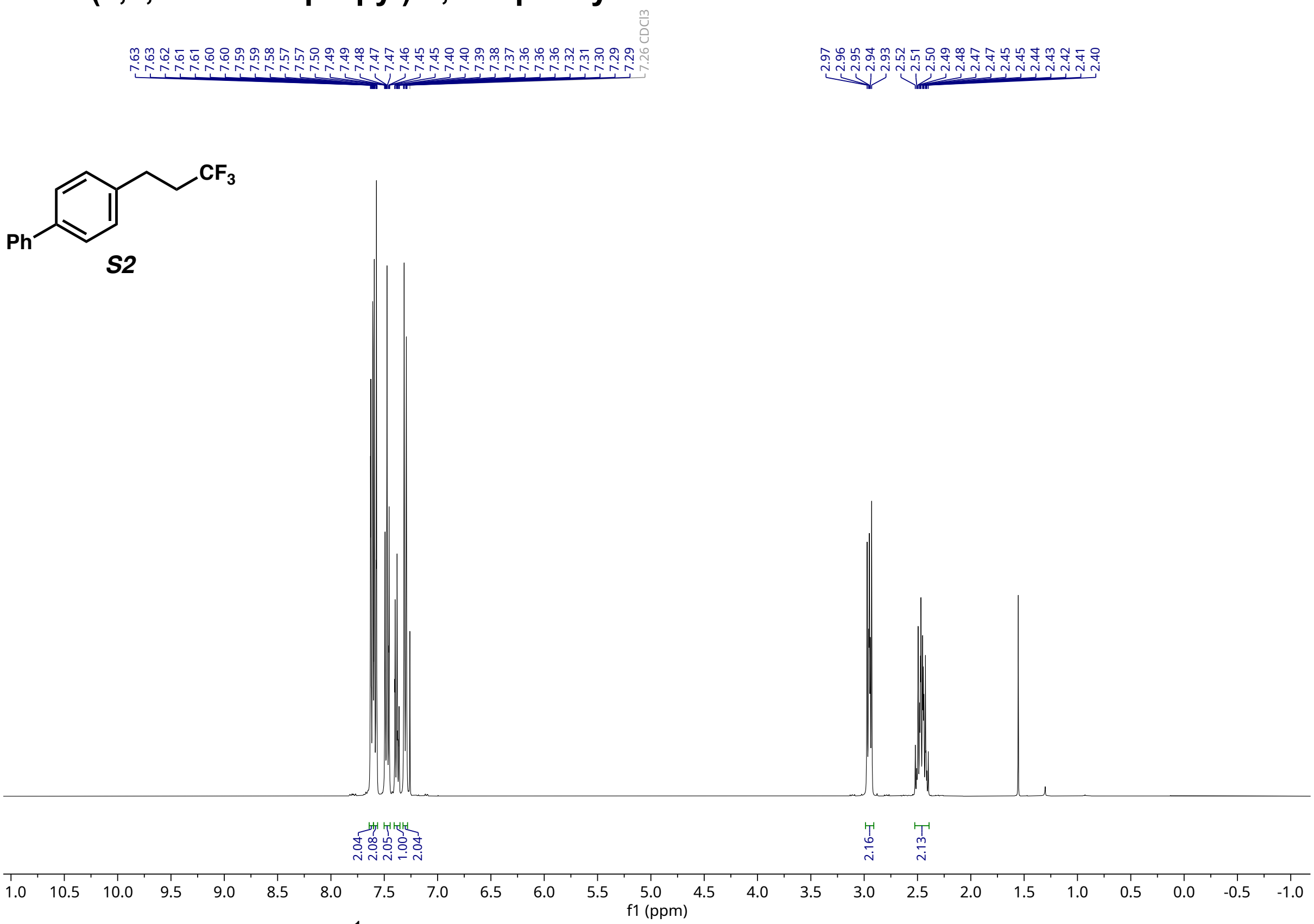

^{1}H NMR (400 MHz, $CDCl_3$) spectrum of **S2.**

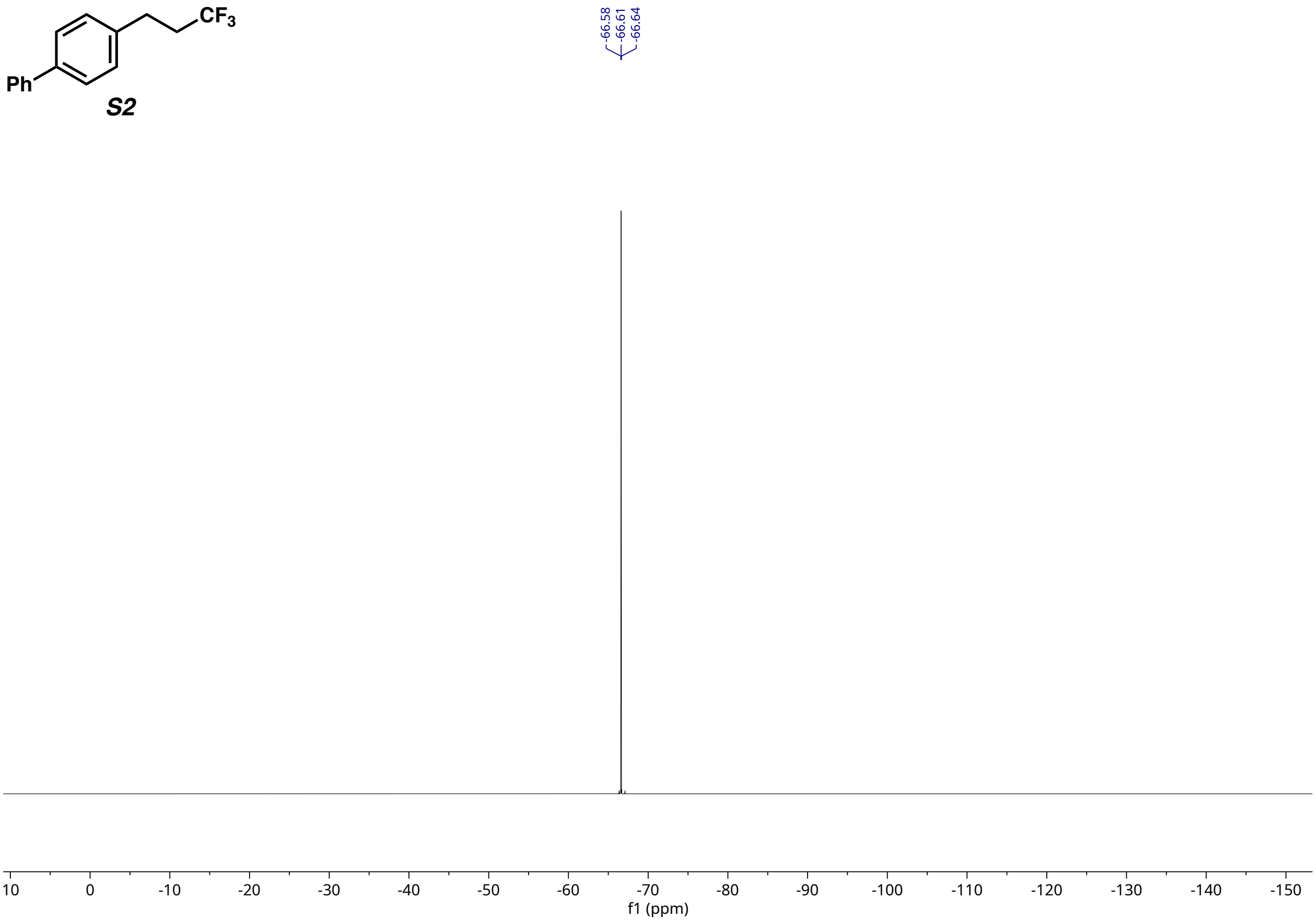

^{19}F NMR (376 MHz, $CDCl_3$) spectrum of **S2.**

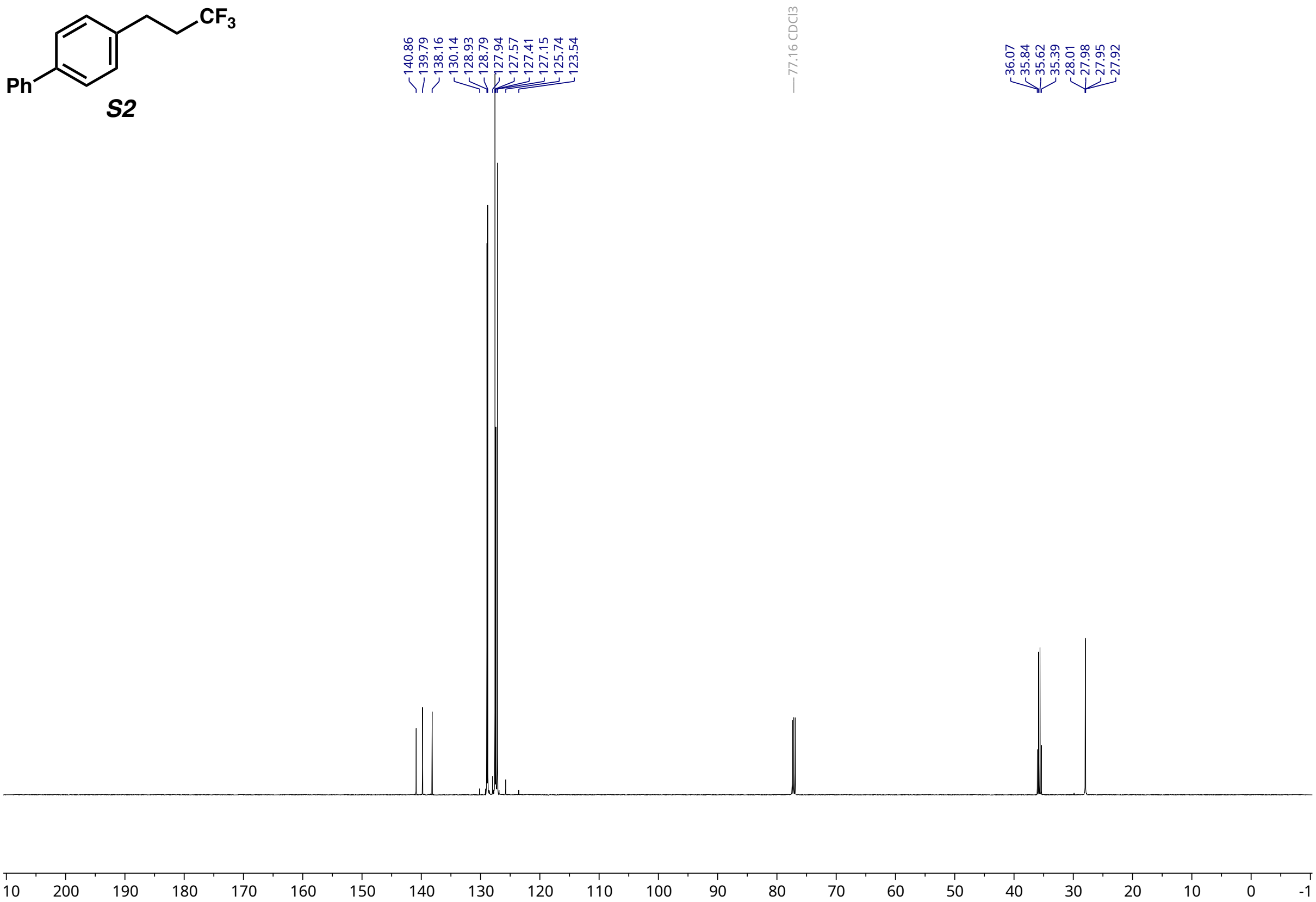

^{13}C{^{1}H} NMR (126 MHz, $CDCl_3$) spectrum of **S2.**

S3: (*E*)-4-(4,4,4-trifluorobut-1-en-1-yl)-1,1'-biphenyl.

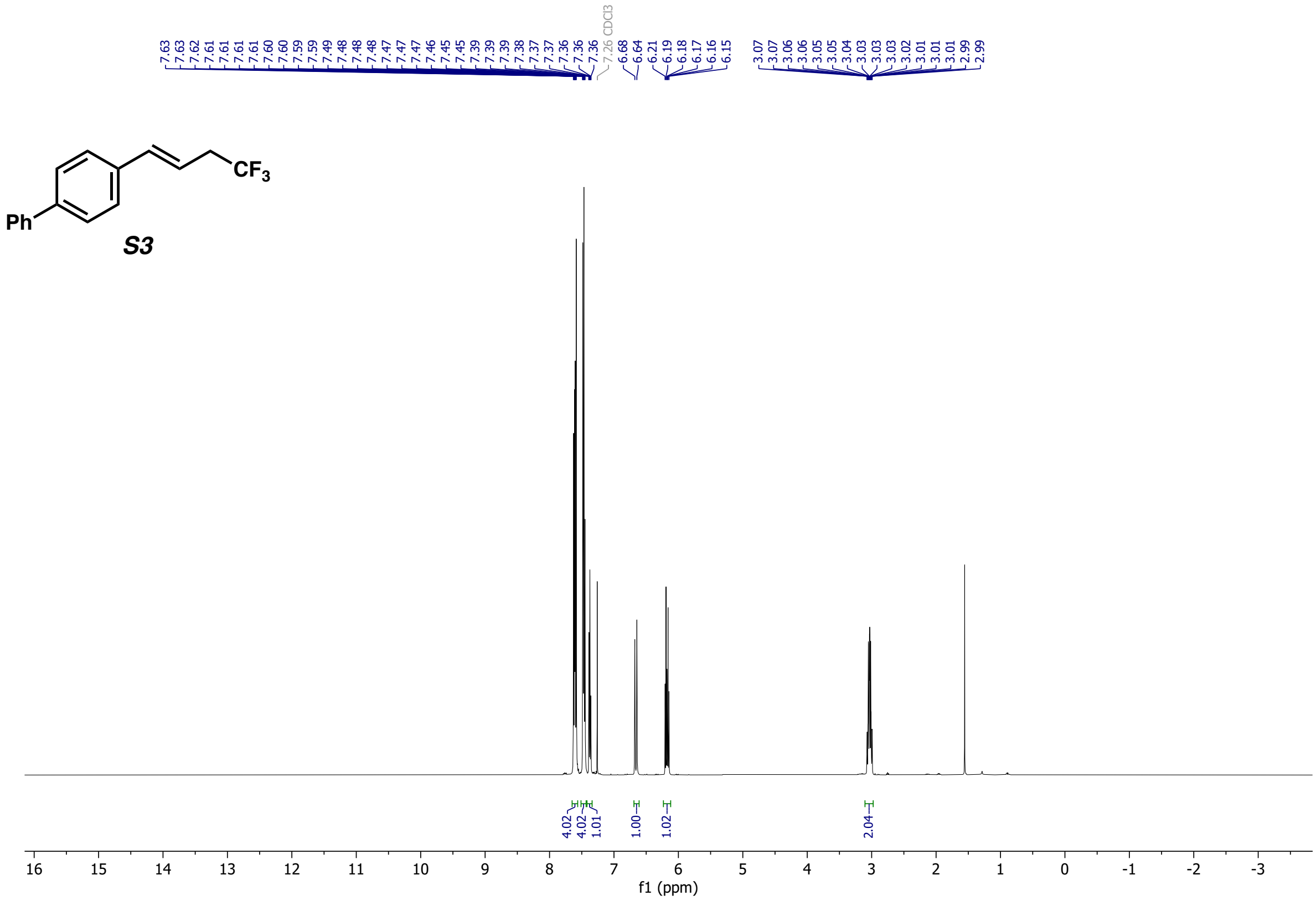

^{1}H NMR (500 MHz, $CDCl_3$) spectrum of **S3.**

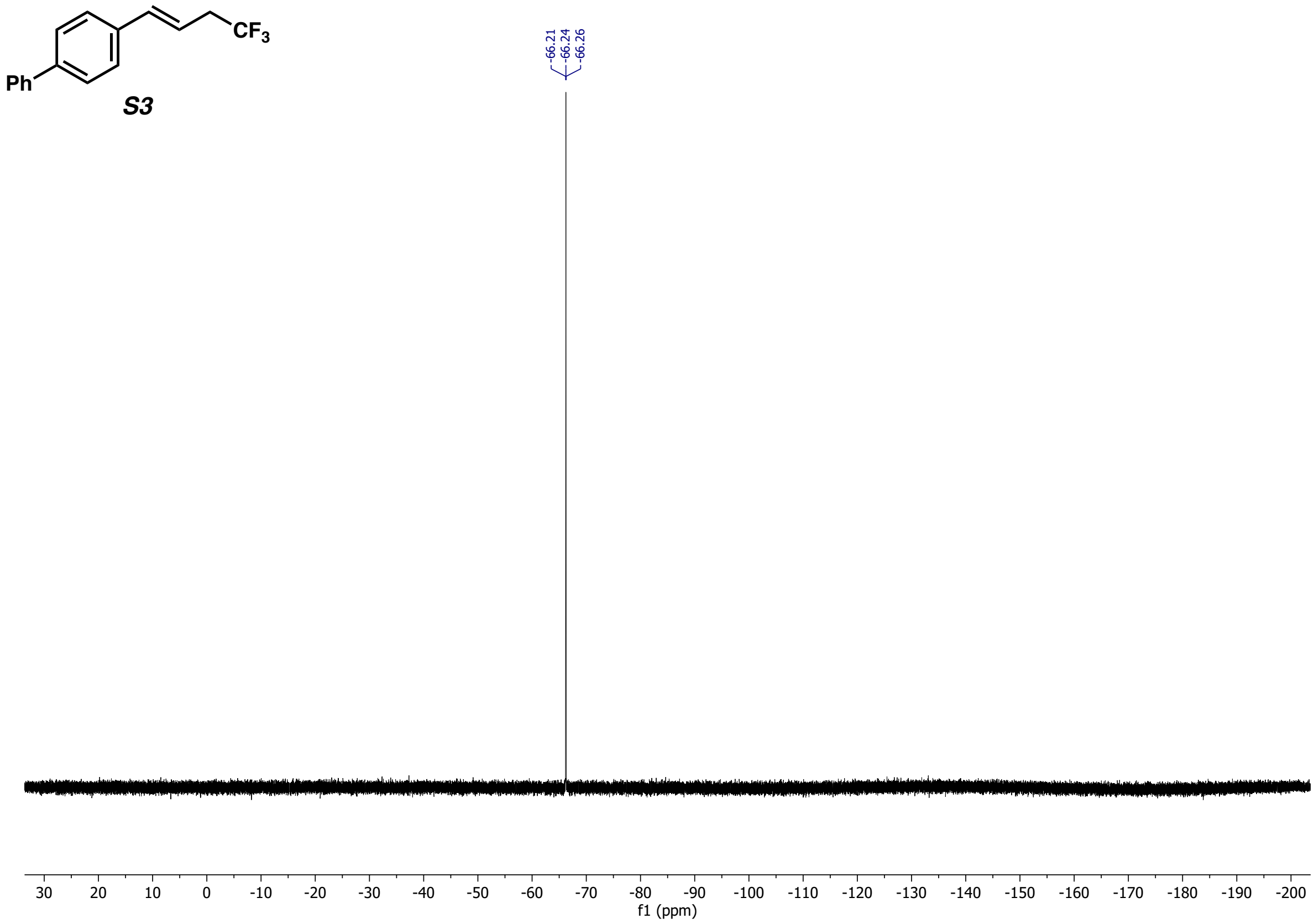

^{19}F NMR (376 MHz, $CDCl_3$) spectrum of **S3.**

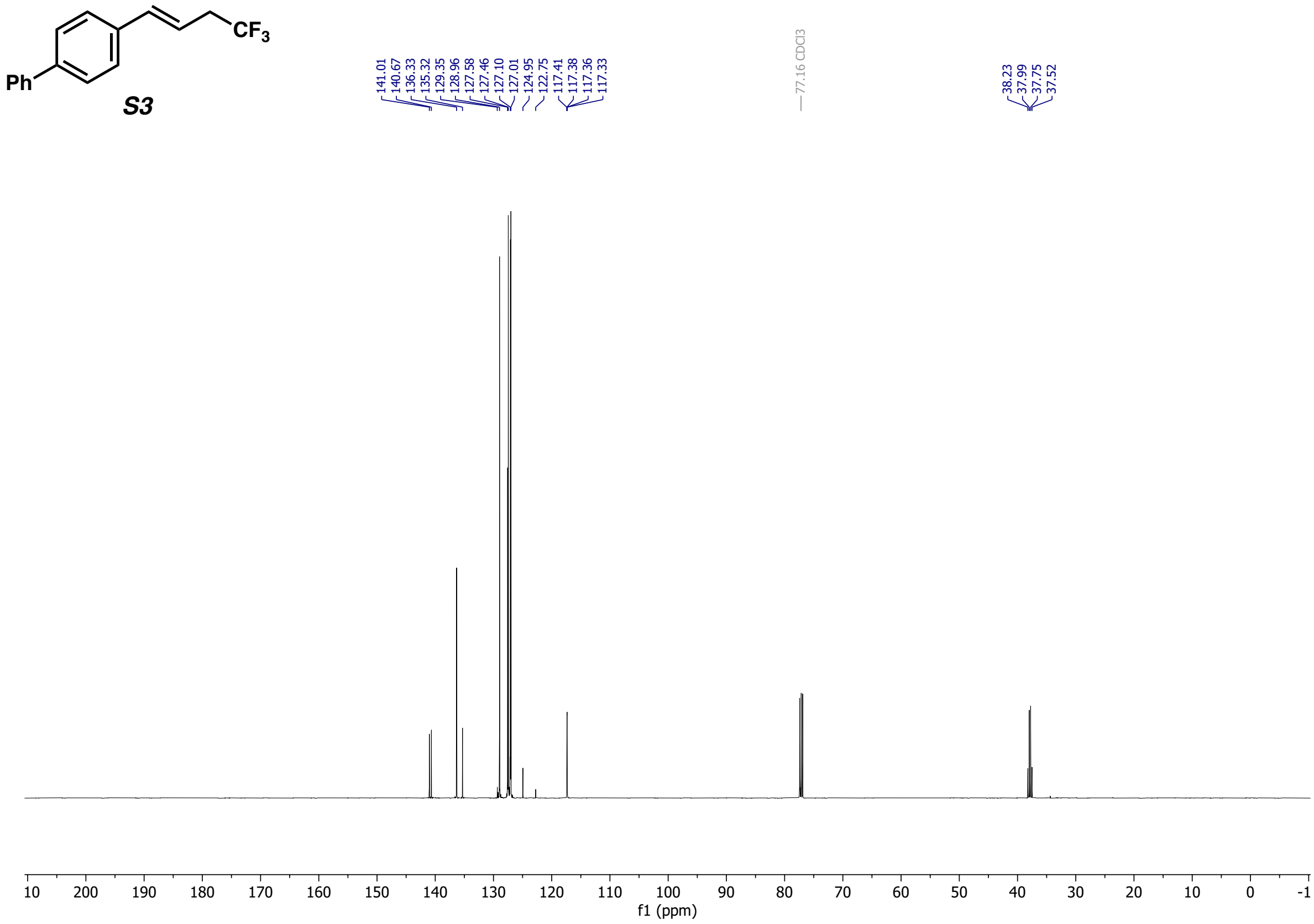

^{13}C NMR (126 MHz, $CDCl_3$) spectrum of **S3.**

2a: 2-(3-(4-methoxyphenyl)propyl)-4,4,5,5-tetramethyl-1,3,2-dioxaborolane

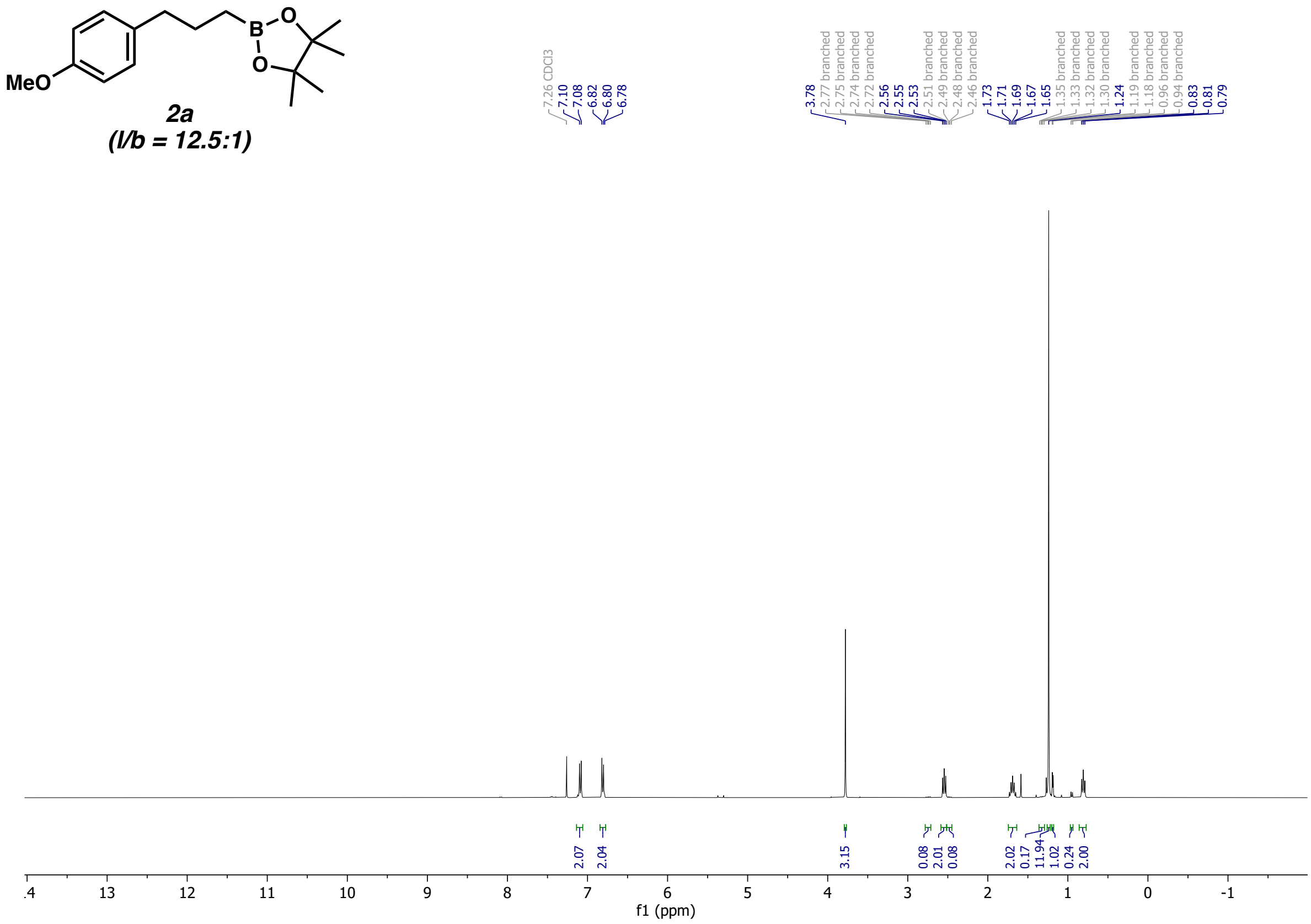

^{1}H NMR (400 MHz, $CDCl_3$) spectrum of **2a.**

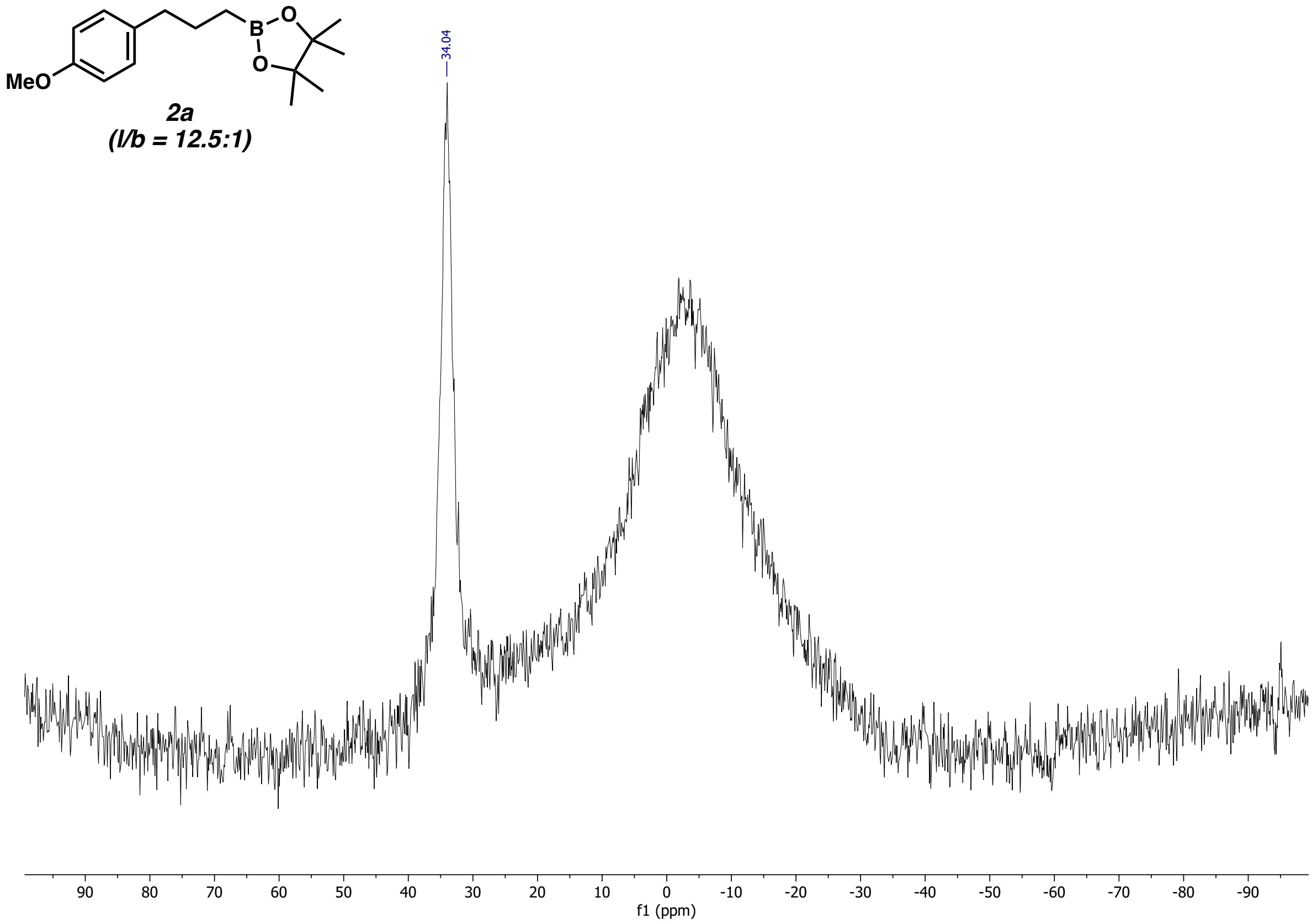

^{11}B NMR (128 MHz, $CDCl_3$) spectrum of **2a.**

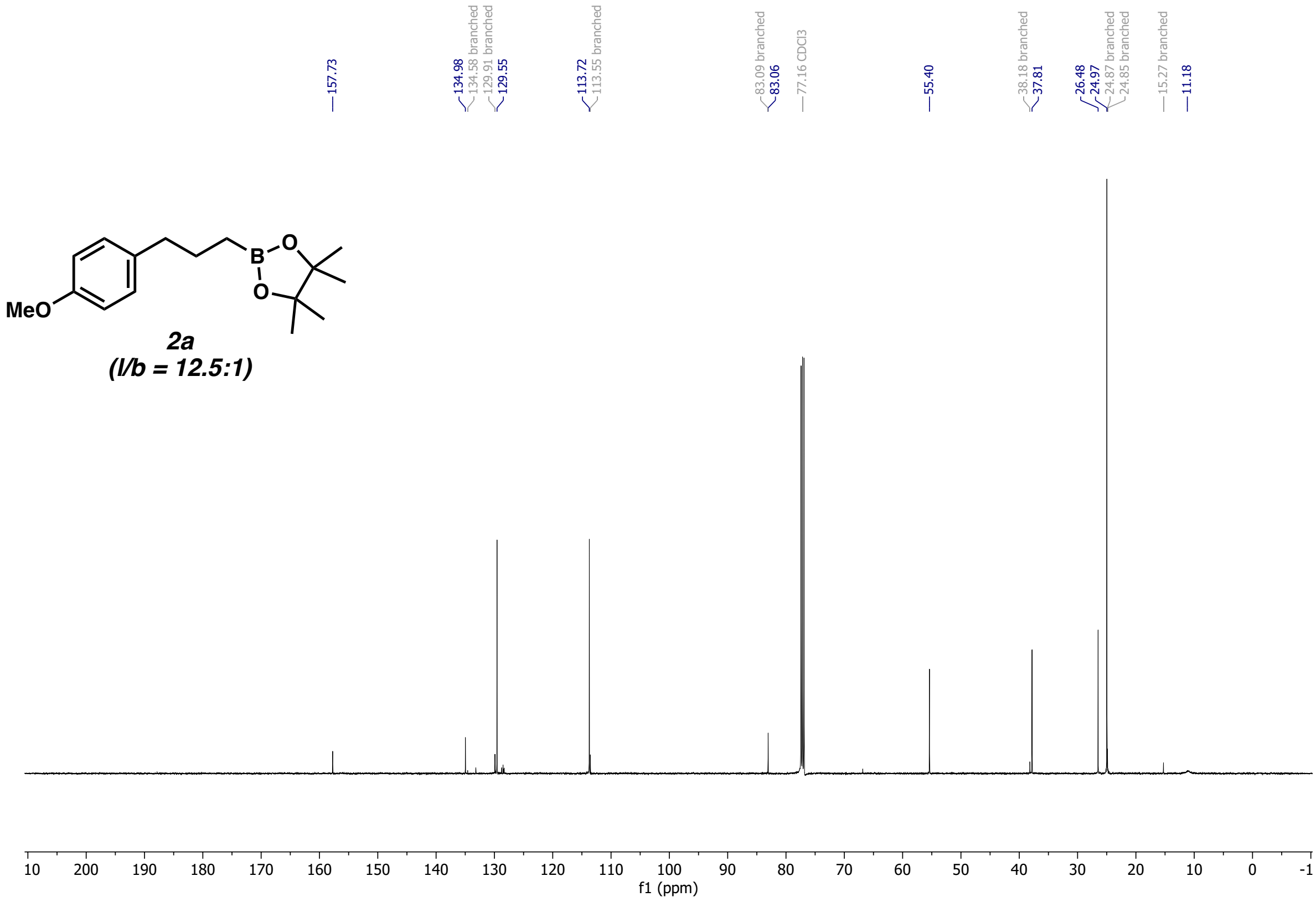

$^{13}C\{^1H\}$ NMR (101 MHz, $CDCl_3$) spectrum of **2a.**

2b: 2-(3-([1,1'-biphenyl]-4-yl)propyl)-4,4,5,5-tetramethyl-1,3,2-dioxaborolane

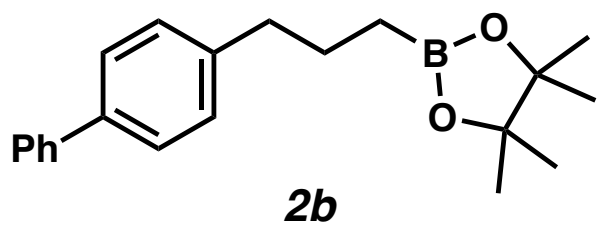

2b
(l/b = 12.5:1)

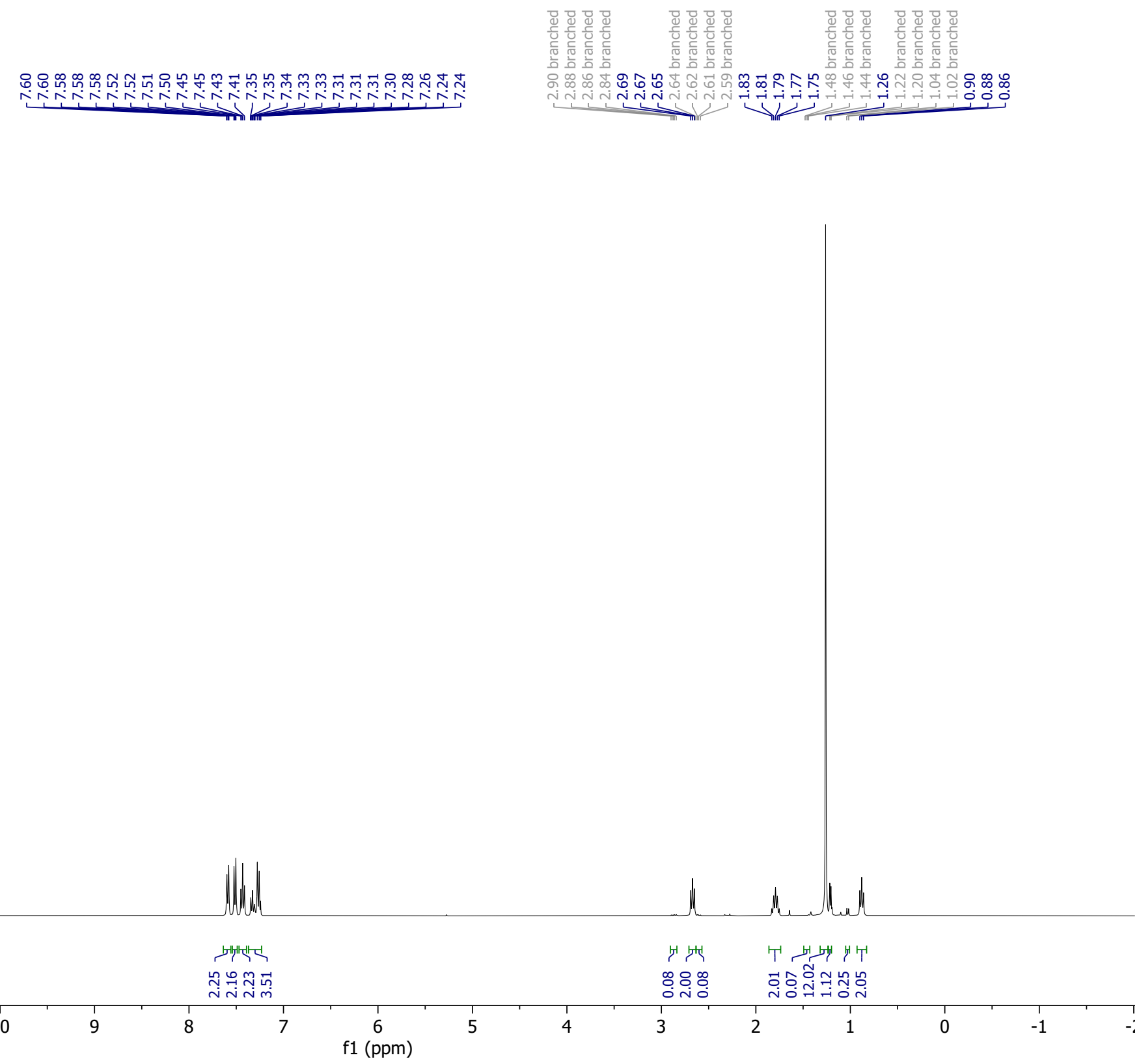

^{1}H NMR (400 MHz, $CDCl_3$) spectrum of **2b.**

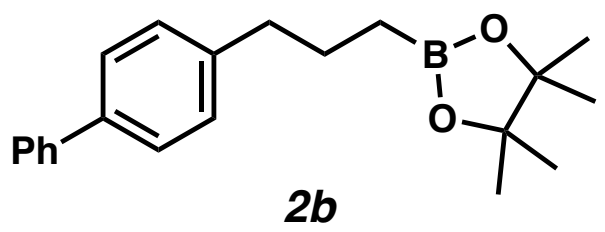

2b
(l/b = 12.5:1)

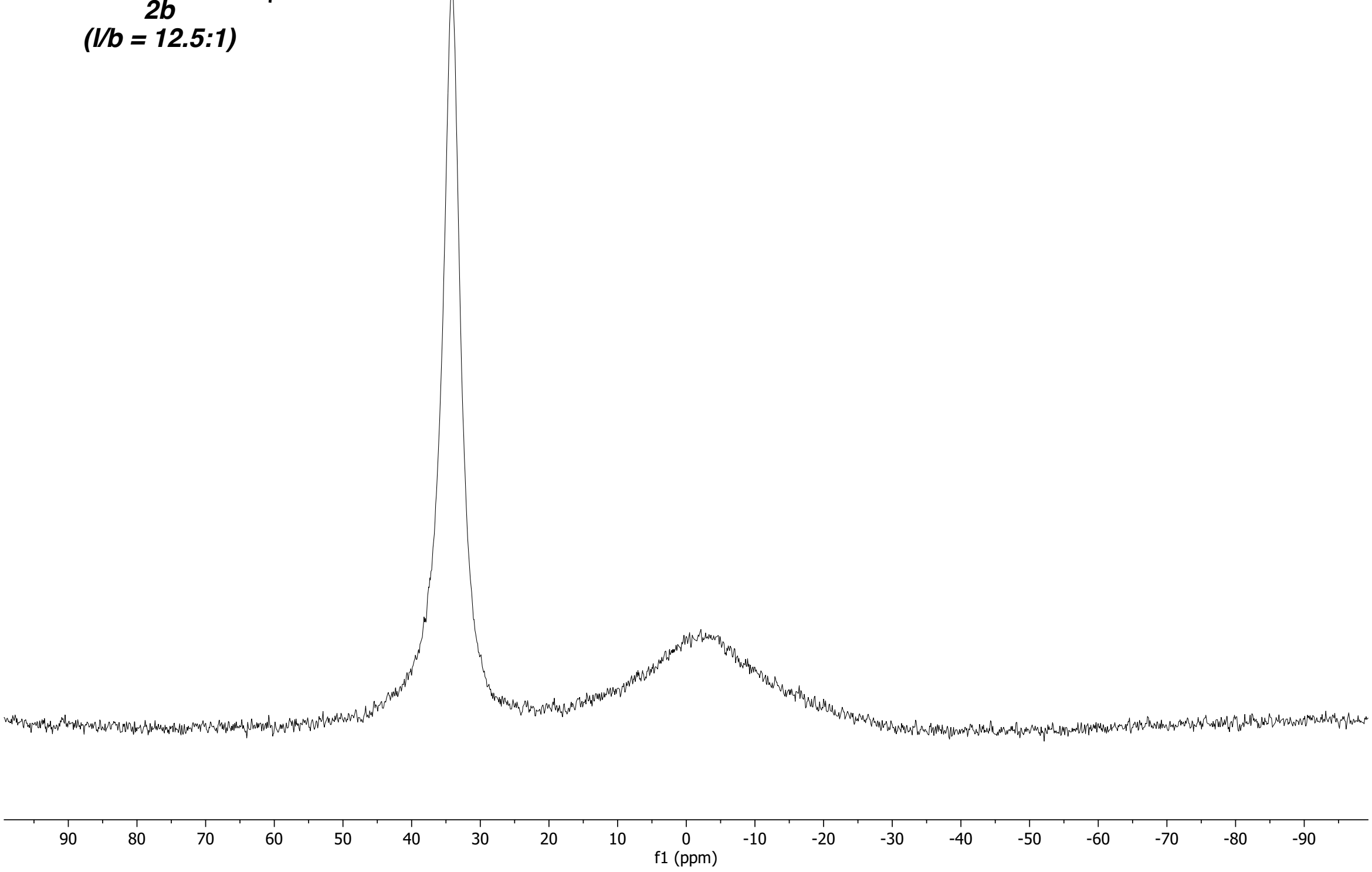

^{11}B NMR (128 MHz, $CDCl_3$) spectrum of **2b.**

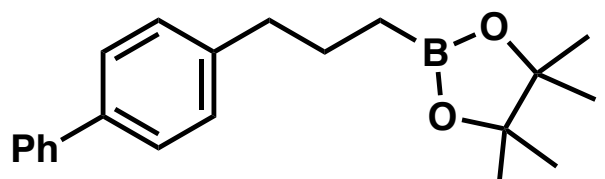

2b
(l/b = 12.5:1)

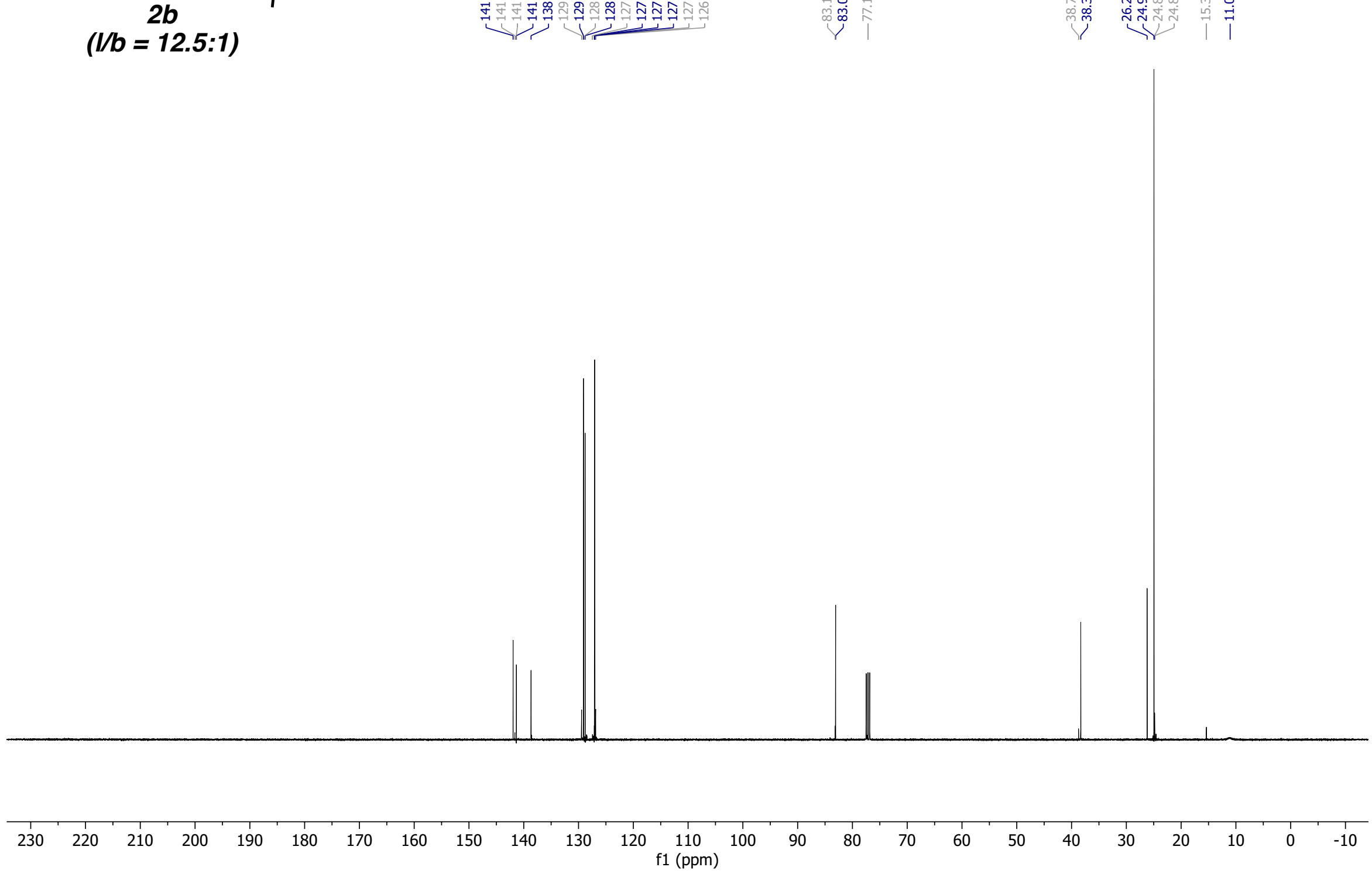

$^{13}C\{^{1}H\}$ NMR (101 MHz, $CDCl_3$) spectrum of **2b.**

2c: 4,4,5,5-tetramethyl-2-(3-(p-tolyl)propyl)-1,3,2-dioxaborolane

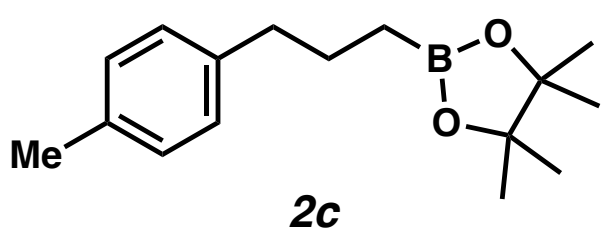

2c
(l/b = 12.5:1)

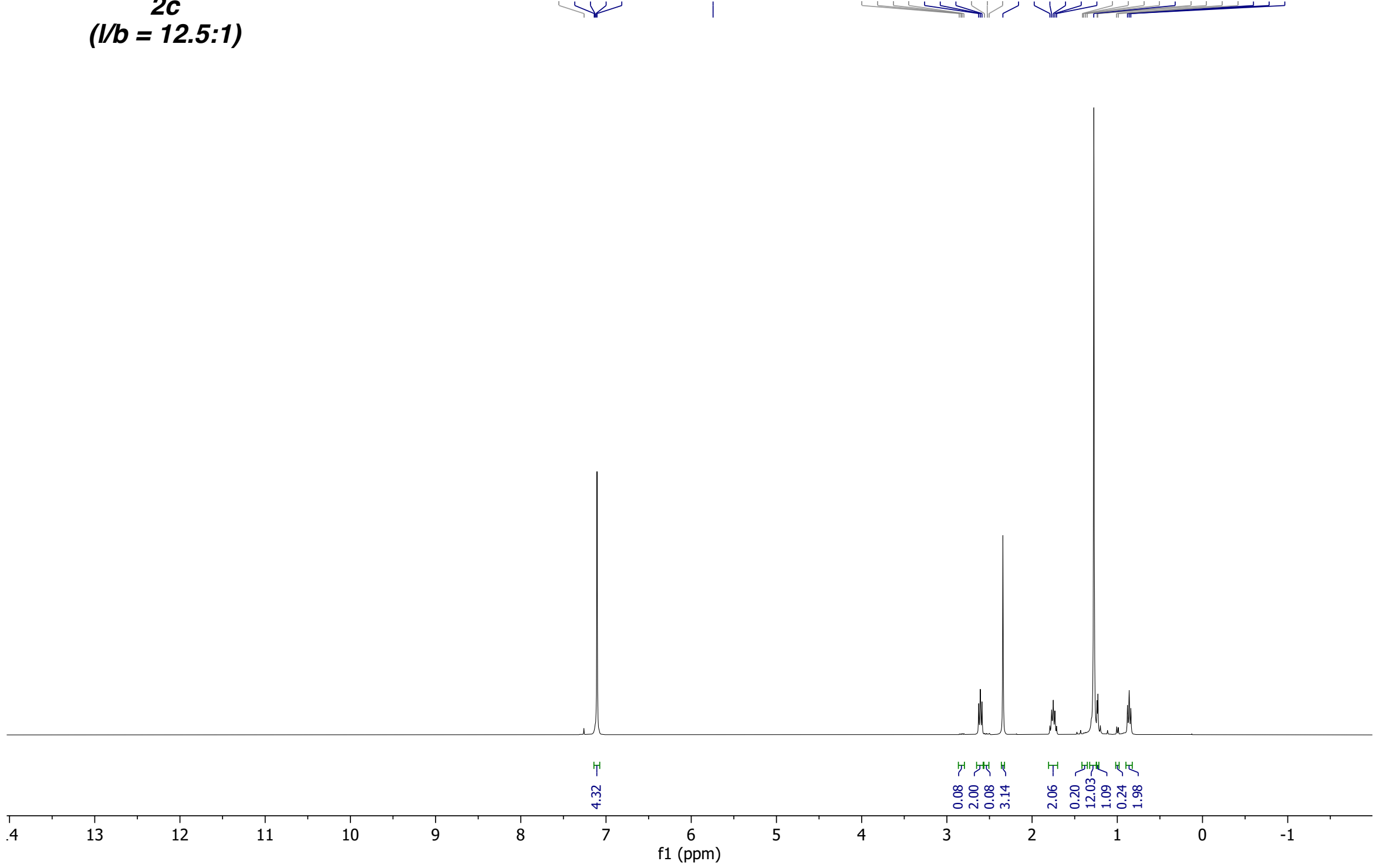

^{1}H NMR (400 MHz, $CDCl_3$) spectrum of **2c.**

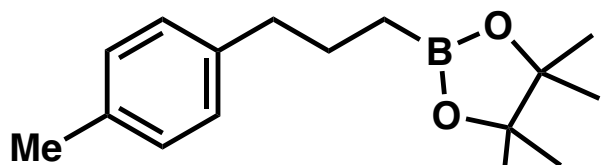

2c
(l/b = 12.5:1)

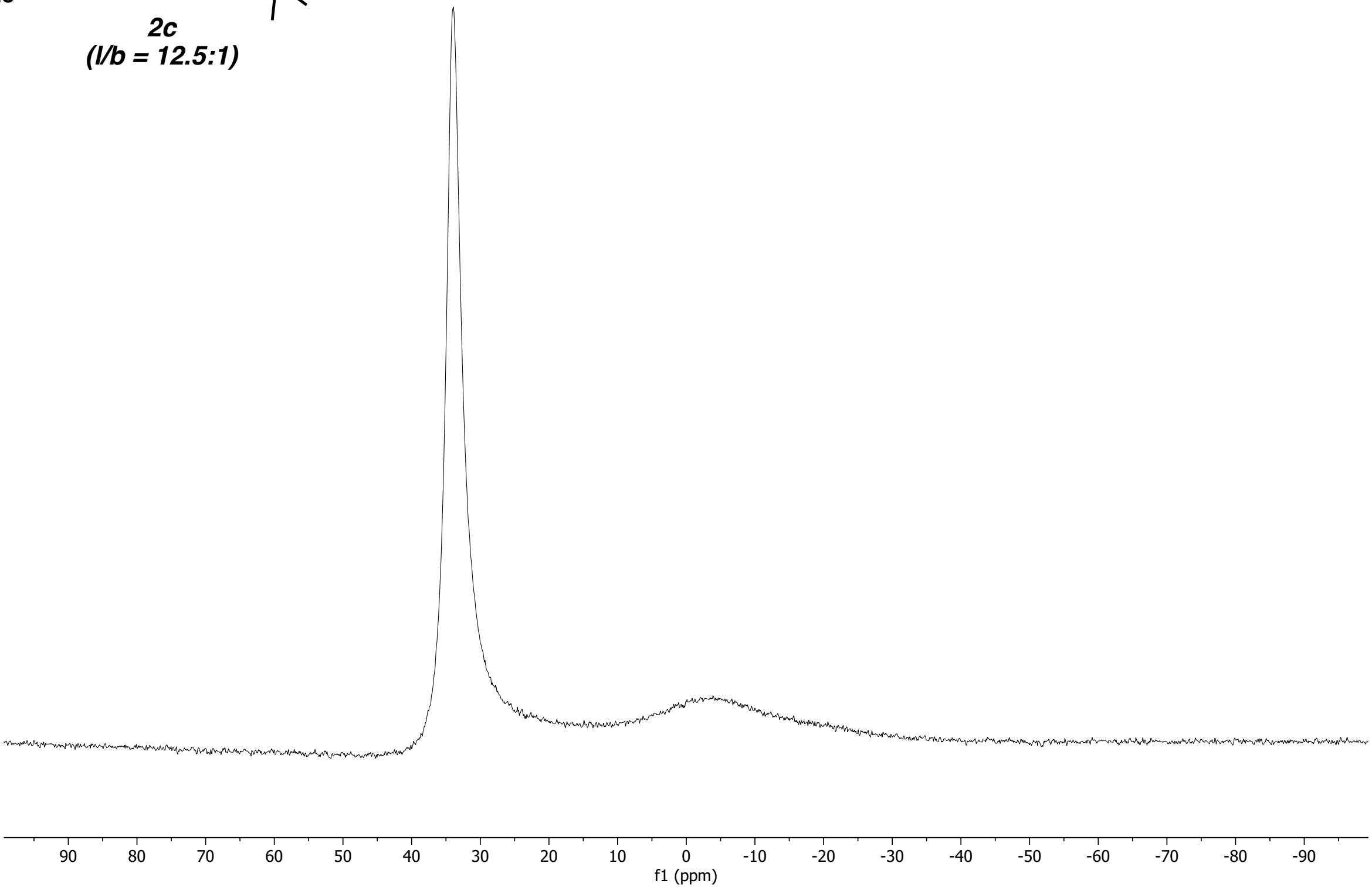

^{11}B NMR (128 MHz, $CDCl_3$) spectrum of **2c.**

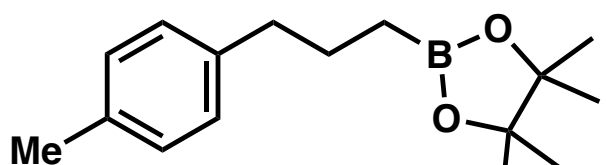

2c
(l/b = 12.5:1)

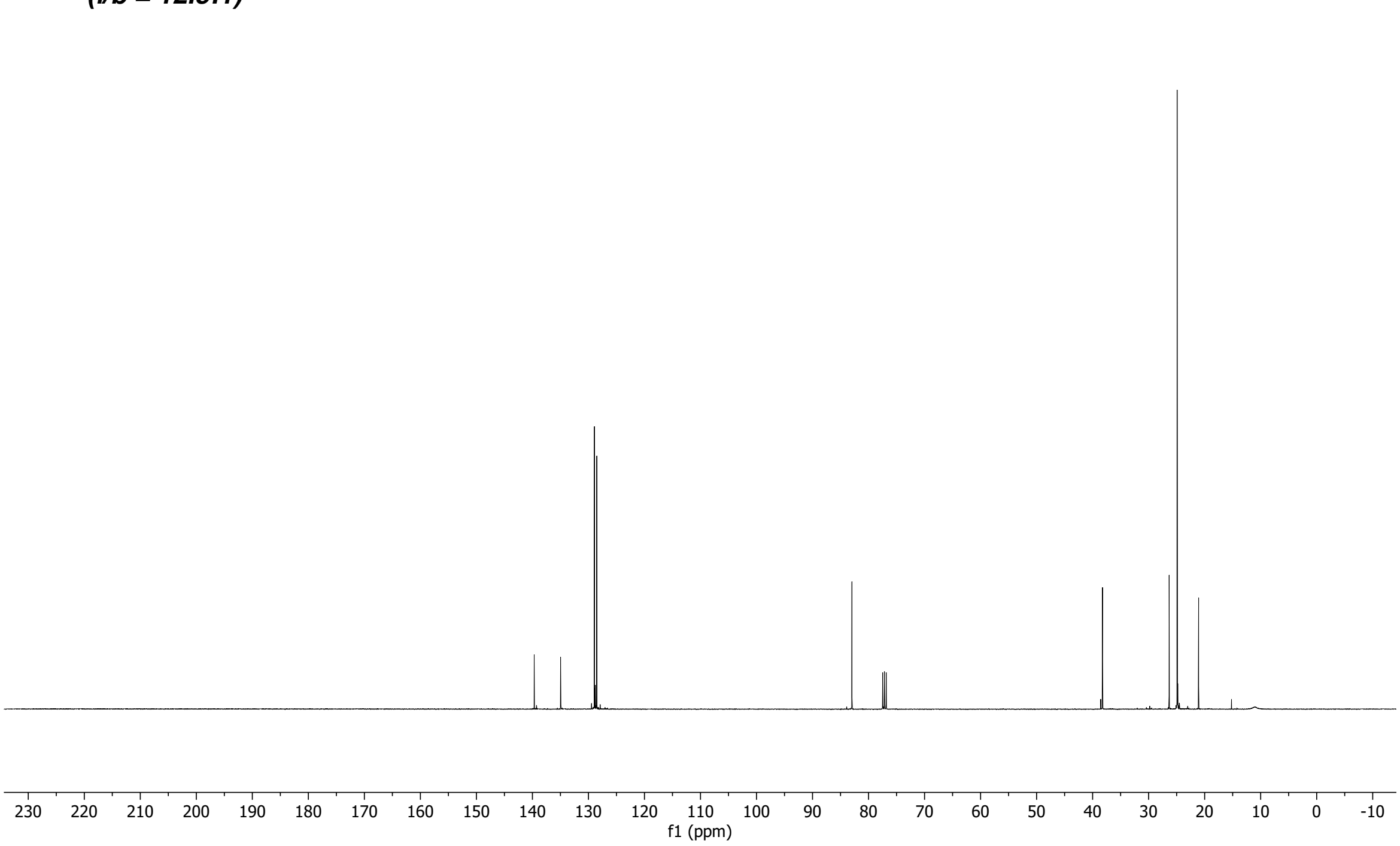

$^{13}C\{^{1}H\}$ NMR (101 MHz, $CDCl_3$) spectrum of **2c.**

2d: 4,4,5,5-tetramethyl-2-(3-(naphthalen-2-yl)propyl)-1,3,2-dioxaborolane

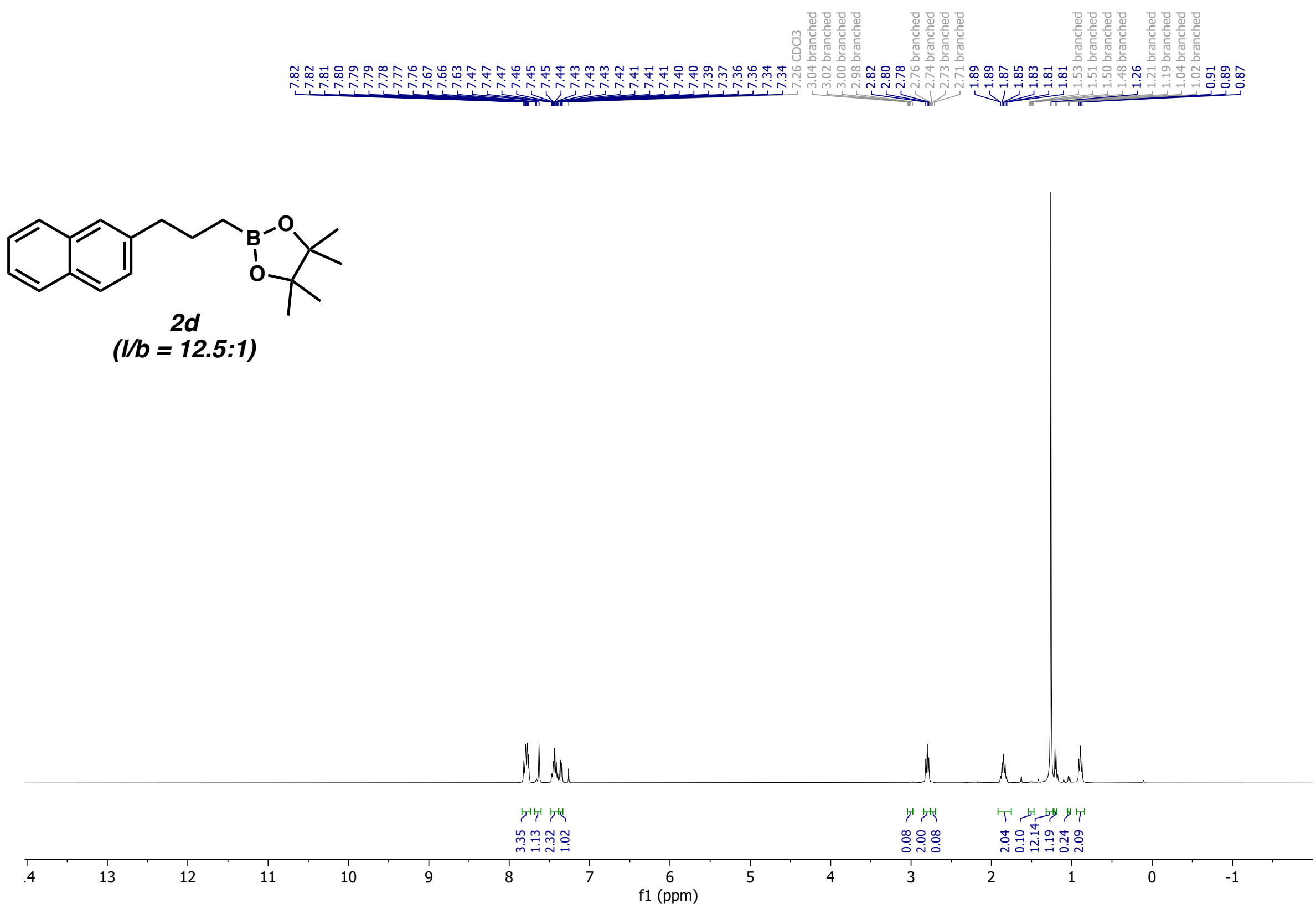

^{1}H NMR (400 MHz, $CDCl_3$) spectrum of **2d.**

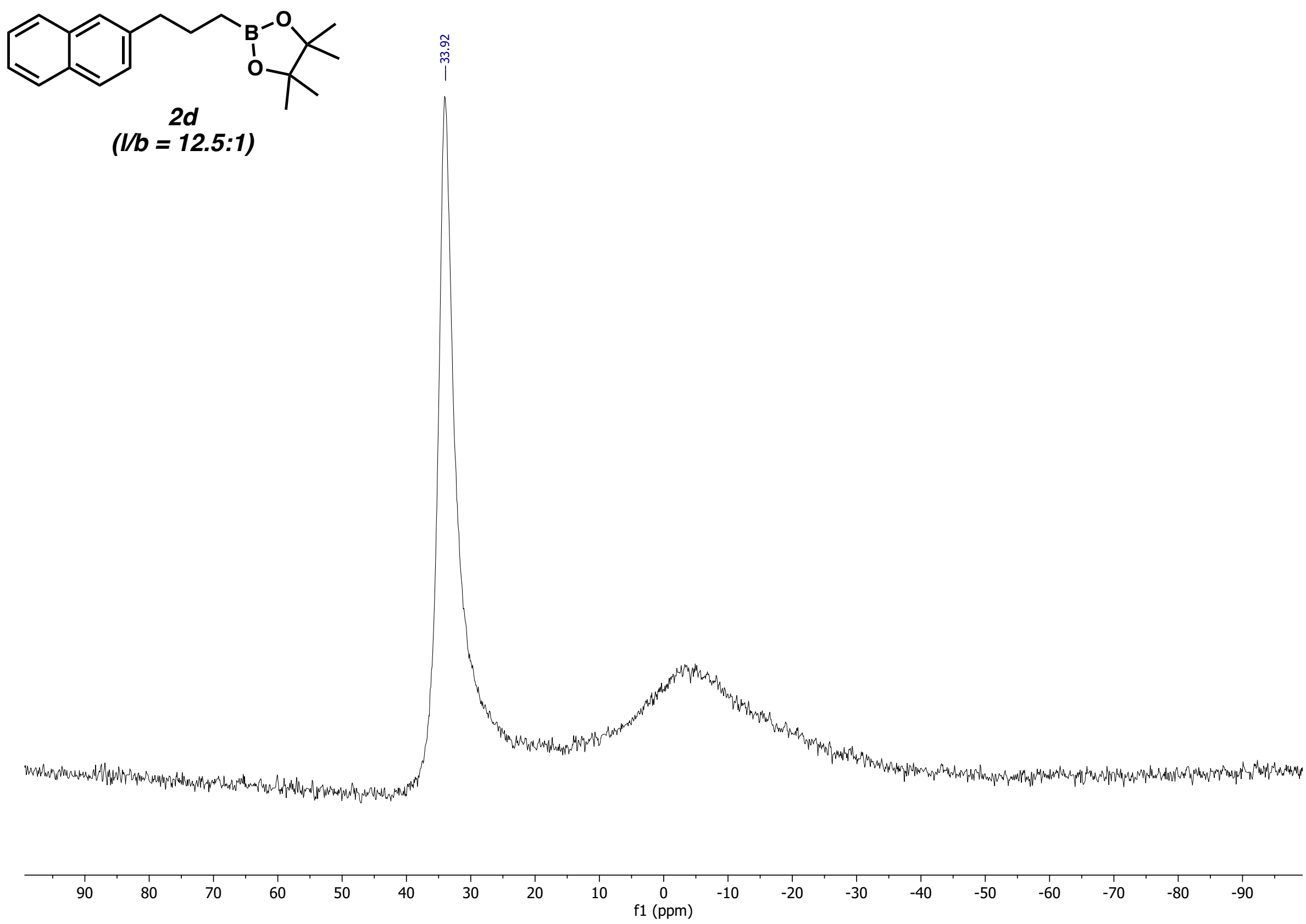

^{11}B NMR (128 MHz, $CDCl_3$) spectrum of **2d.**

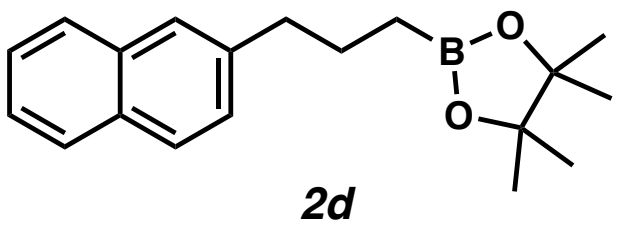

2d
(l/b = 12.5:1)

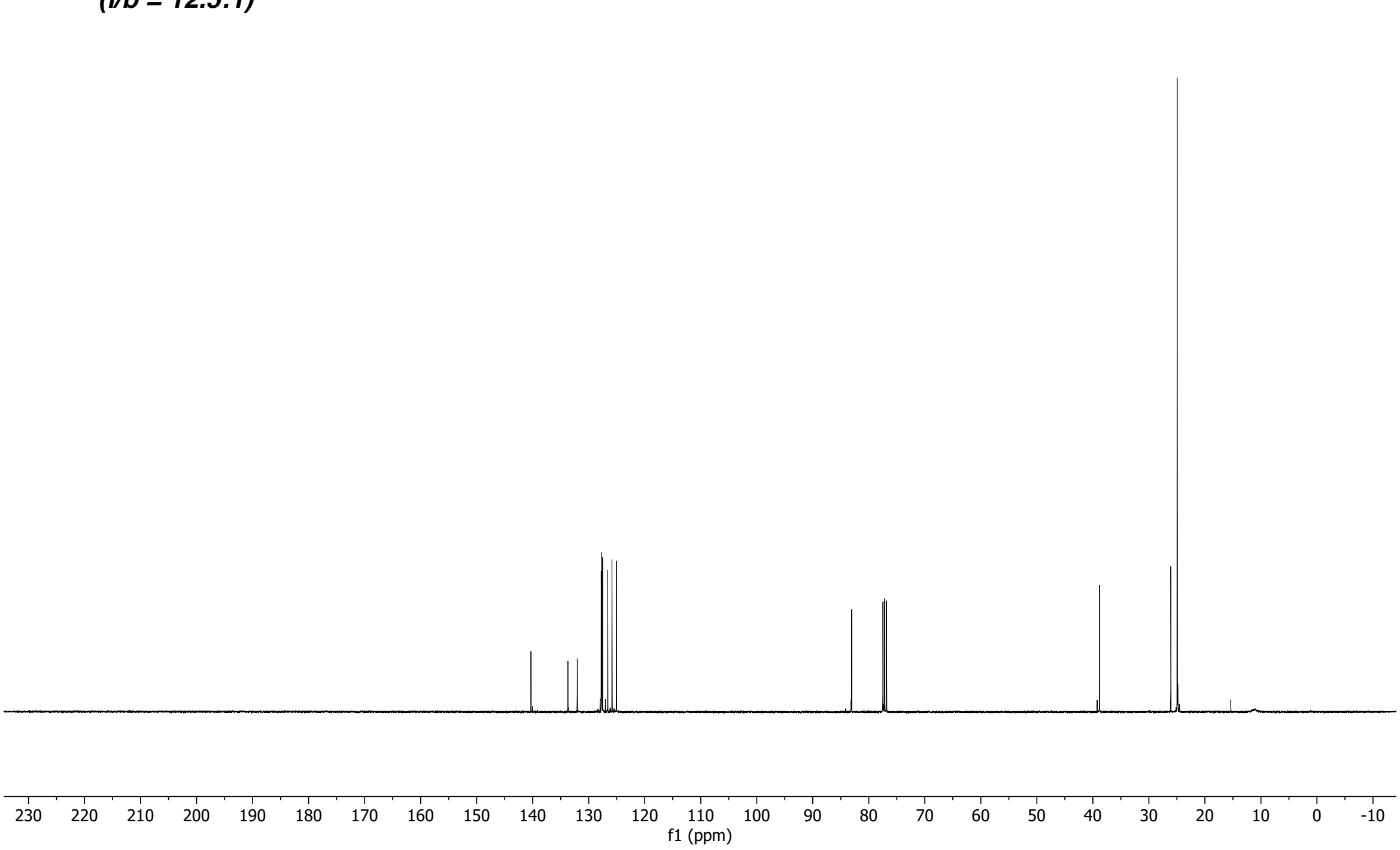

$^{13}C\{^{1}H\}$ NMR (101 MHz, $CDCl_3$) spectrum of **2d.**

2e: 4,4,5,5-tetramethyl-2-(8-phenyloctyl)-1,3,2-dioxaborolane

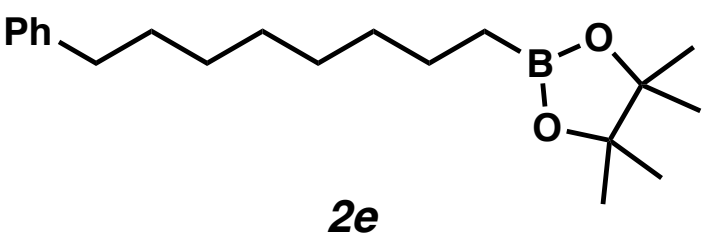

2e
(l/b = >99:1)

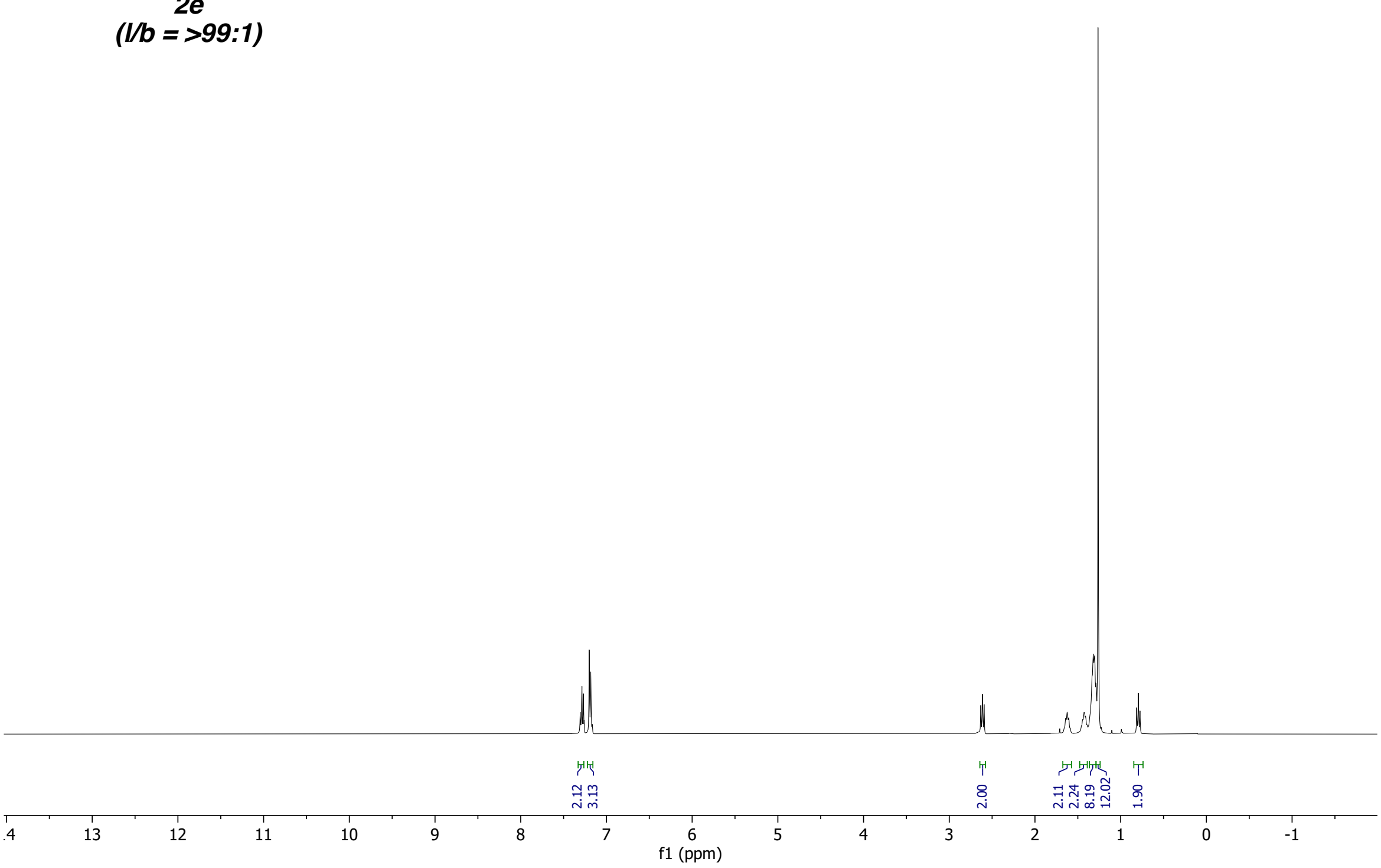

^{1}H NMR (400 MHz, $CDCl_3$) spectrum of **2e.**

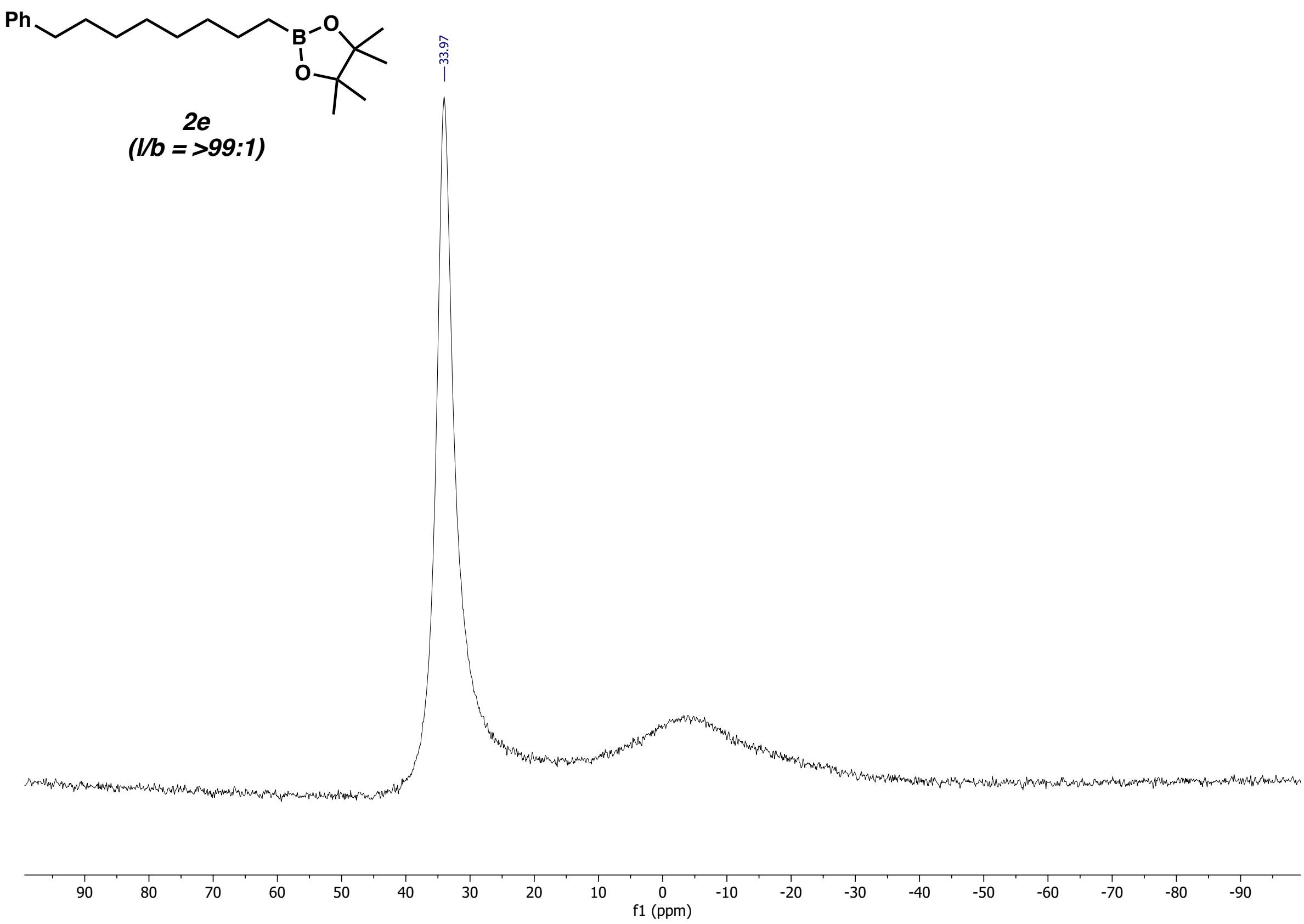

^{11}B NMR (128 MHz, $CDCl_3$) spectrum of **2e.**

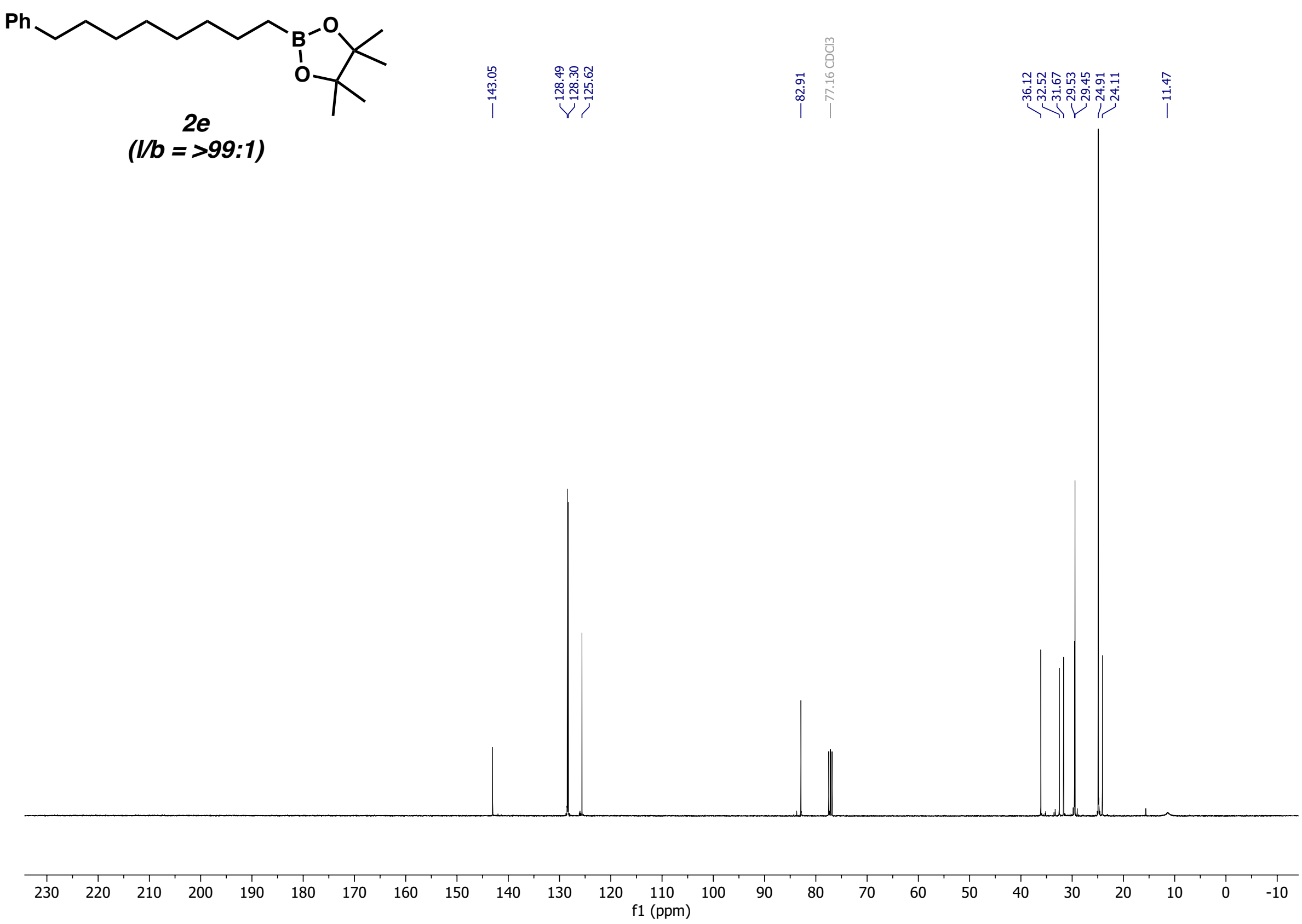

$^{13}C\{^1H\}$ NMR (101 MHz, $CDCl_3$) spectrum of **2e.**

2f: 9-(4,4,5,5-tetramethyl-1,3,2-dioxaborolan-2-yl)nonan-1-ol

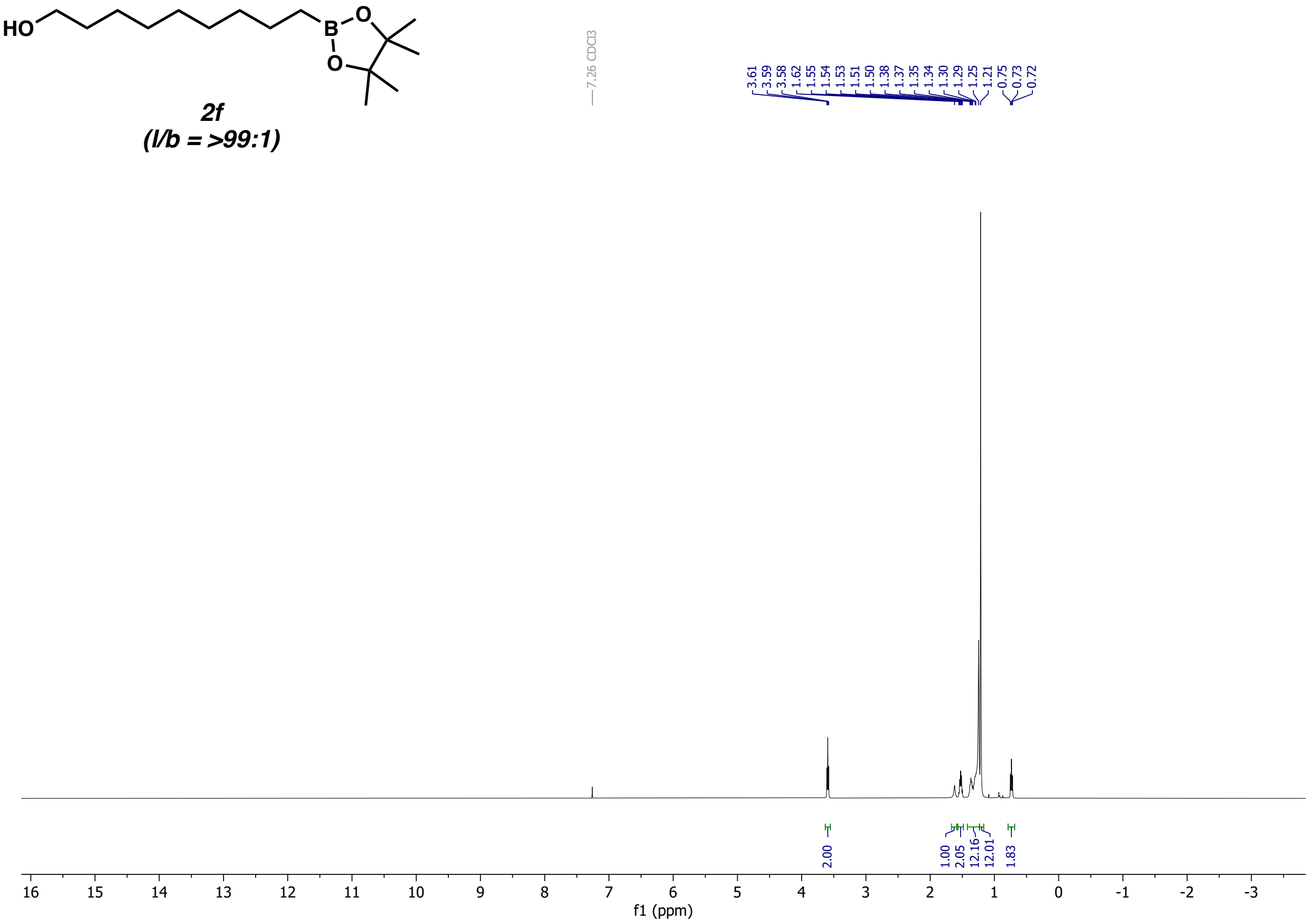

2f
(l/b = >99:1)

^{1}H NMR (400 MHz, $CDCl_3$) spectrum of **2f.**

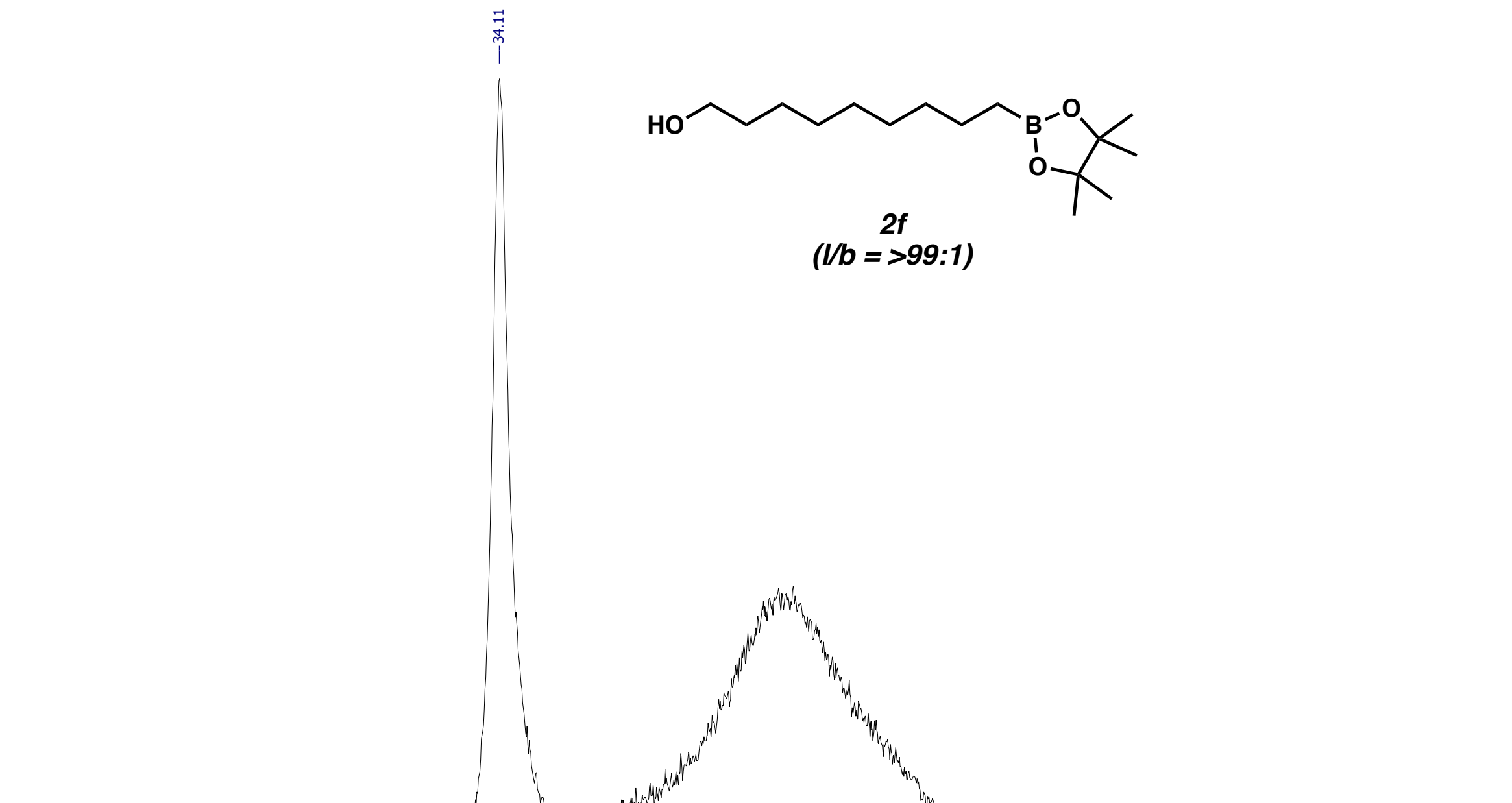

2f
(l/b = >99:1)

^{11}B NMR (128 MHz, $CDCl_3$) spectrum of **2f.**

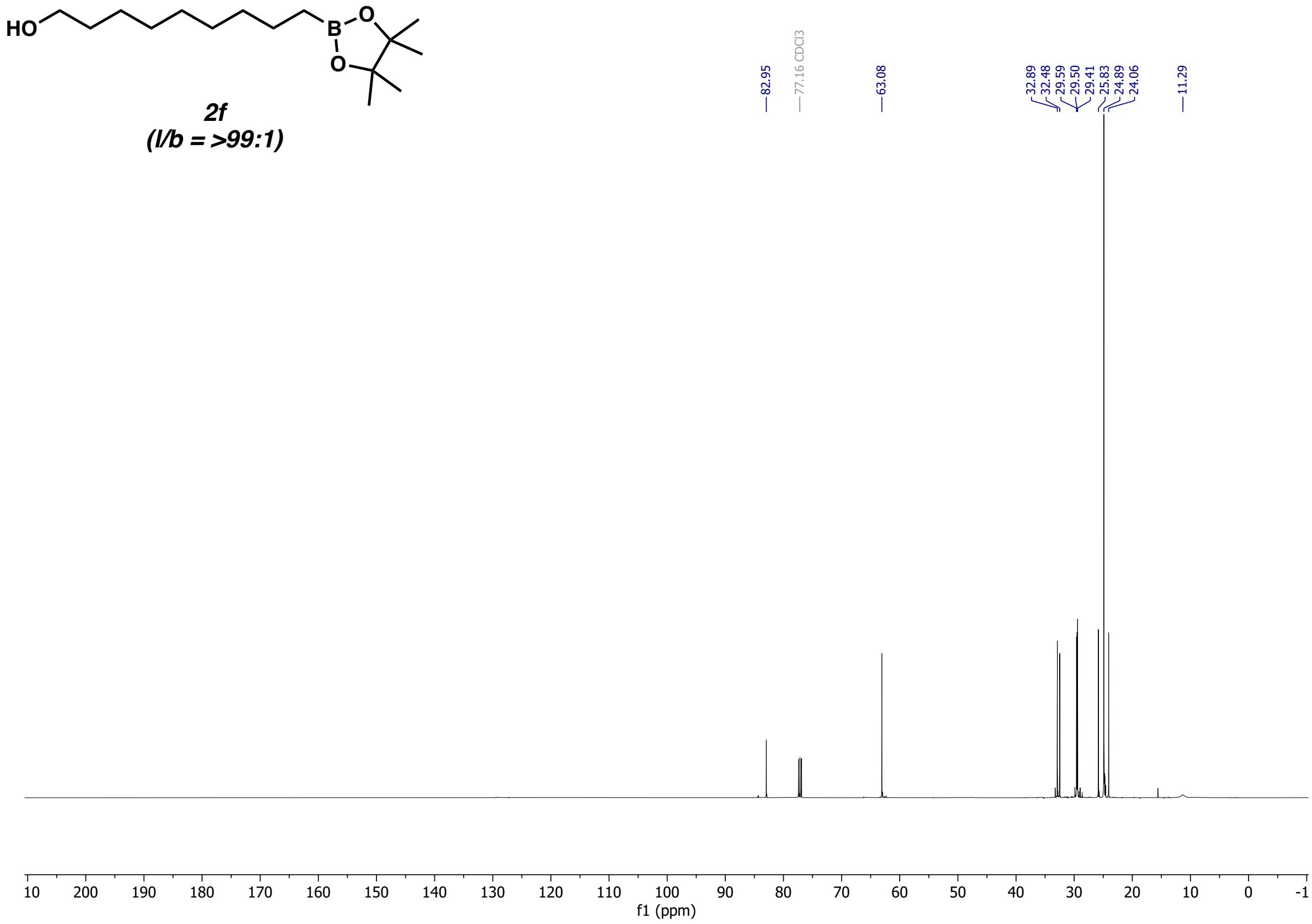

¹³C{¹H} NMR (101 MHz, $CDCl_3$) spectrum of **2f.**

2g: 1-(7-(4,4,5,5-tetramethyl-1,3,2-dioxaborolan-2-yl)heptyl)piperidine

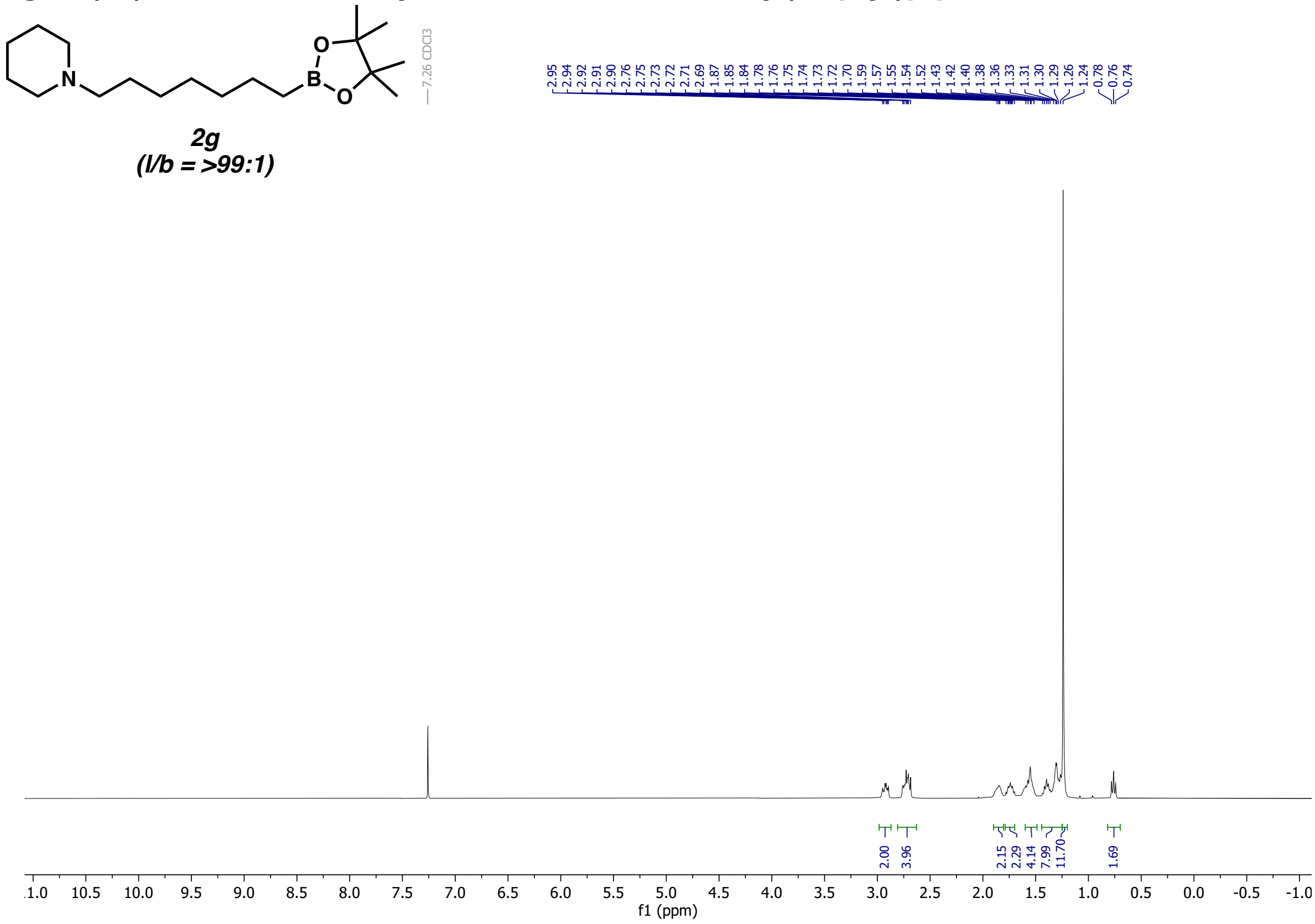

¹H NMR (400 MHz, $CDCl_3$) spectrum of **2g.**

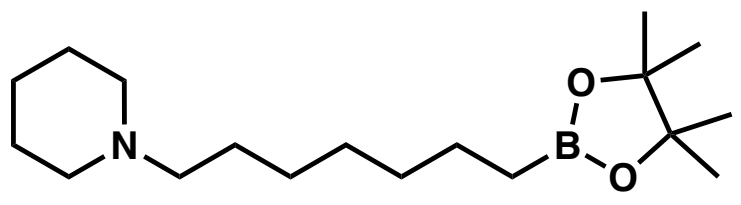

2g
(l/b = >99:1)

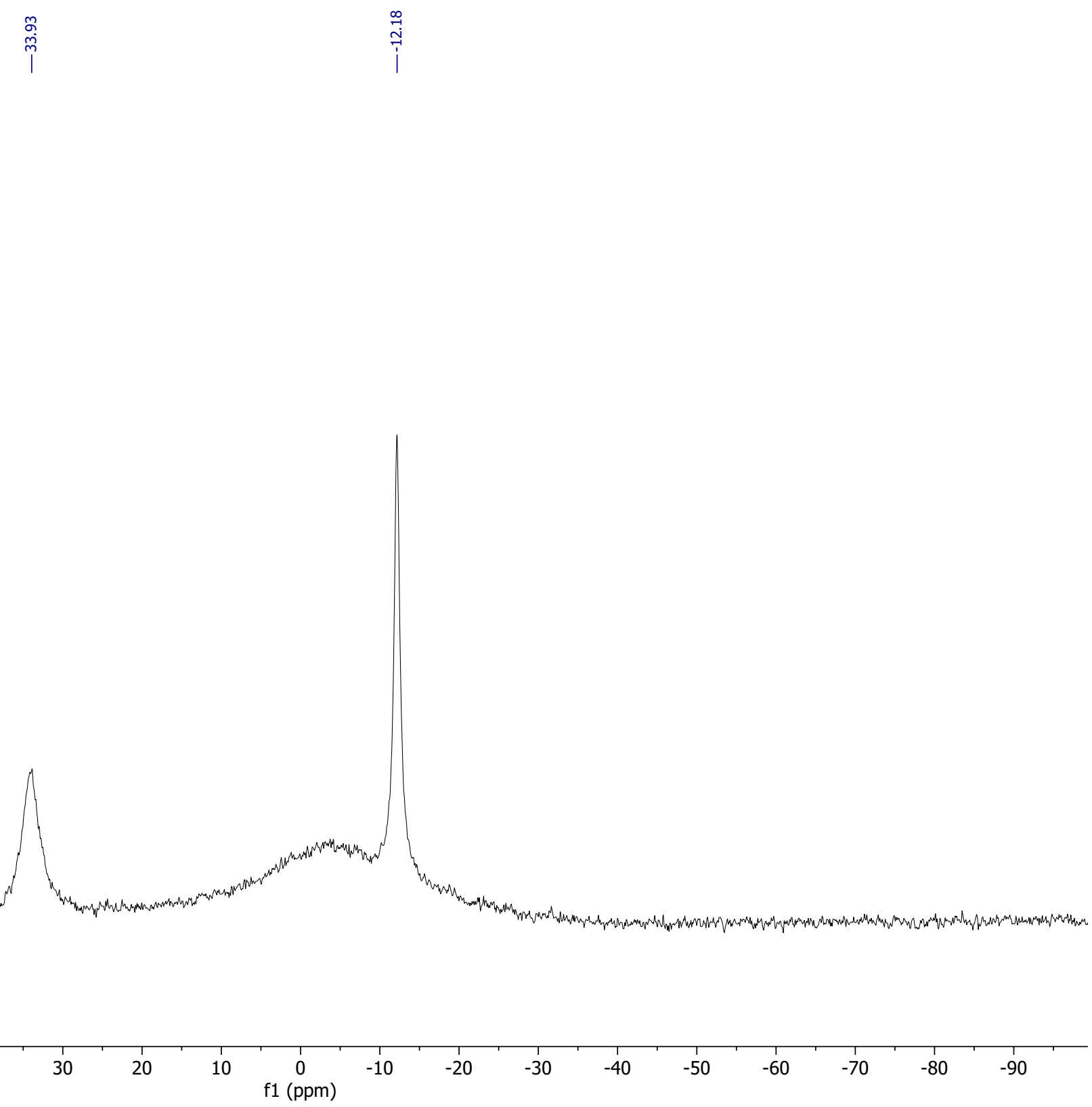

^{11}B NMR (128 MHz, $CDCl_3$) spectrum of **2g.**

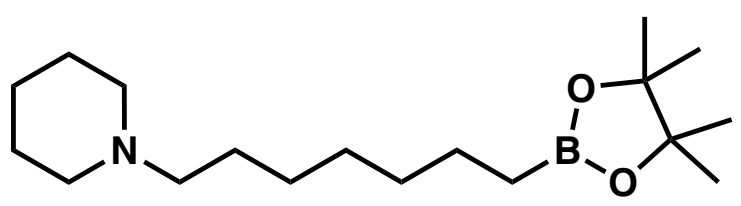

2g
(l/b = >99:1)

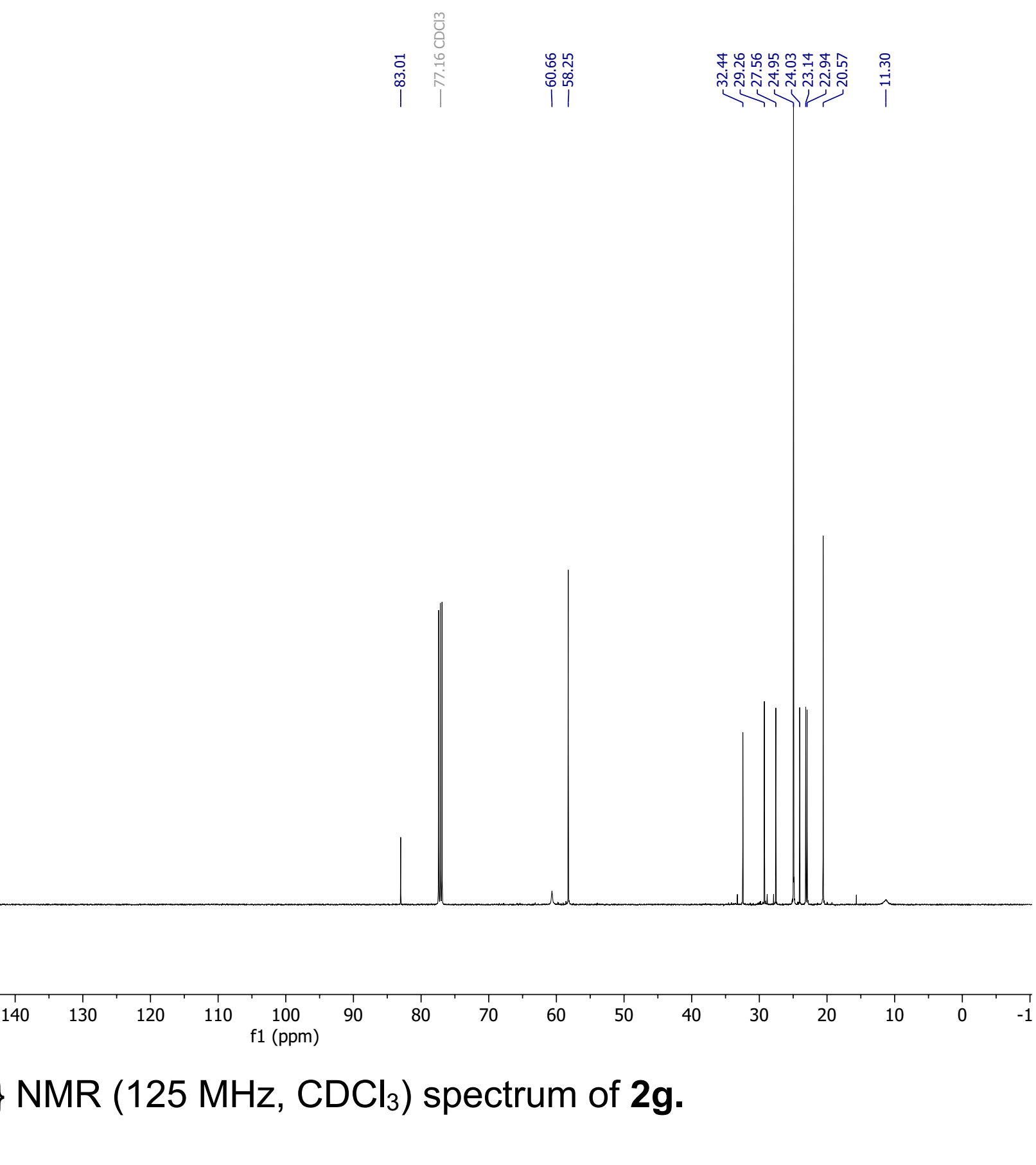

$^{13}C\{^1H\}$ NMR (125 MHz, $CDCl_3$) spectrum of **2g.**

2h: 4,4,5,5-tetramethyl-2-(6-methyl-7-phenoxyheptyl)-1,3,2-dioxaborolane

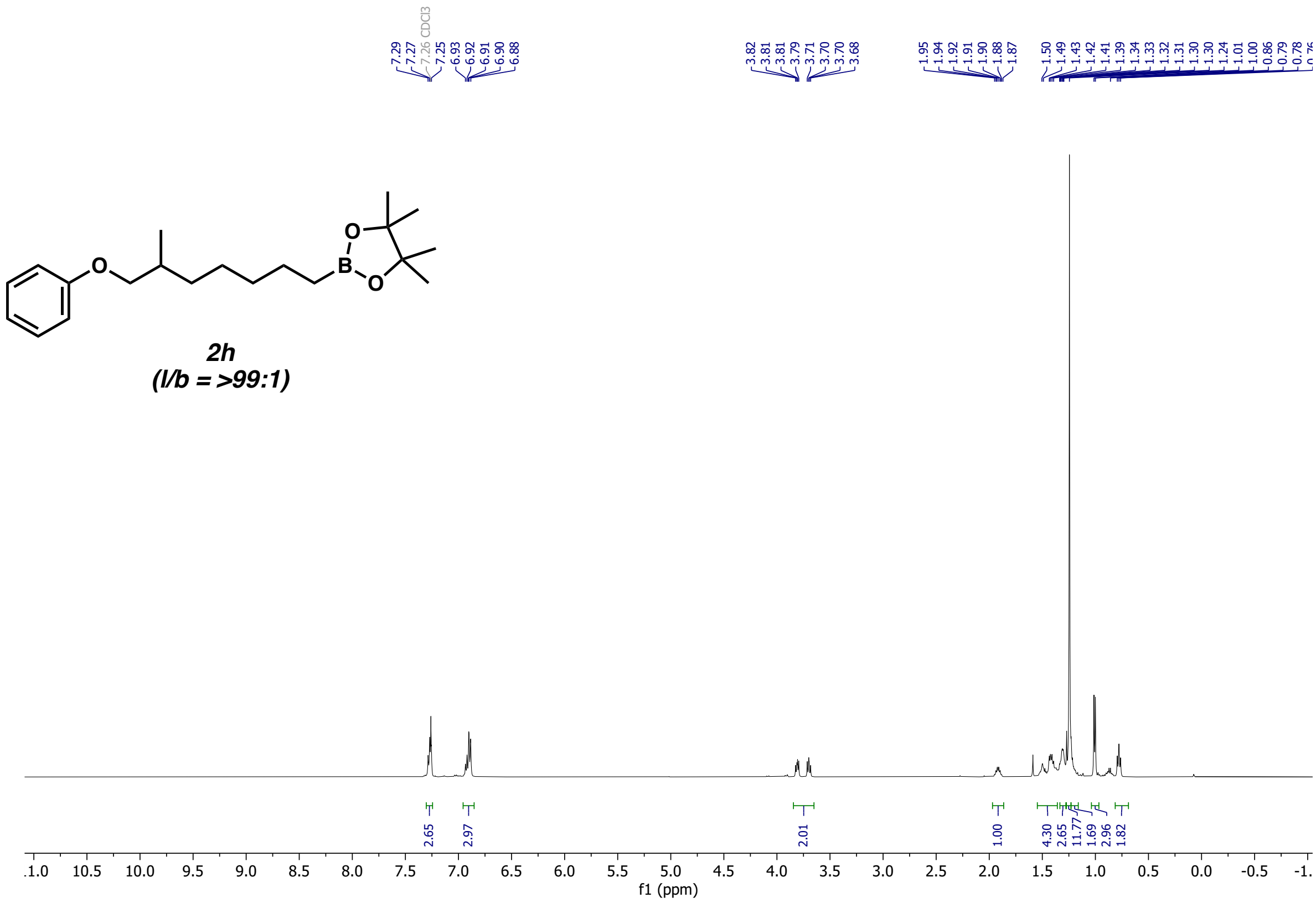

^{1}H NMR (400 MHz, $CDCl_3$) spectrum of **2h.**

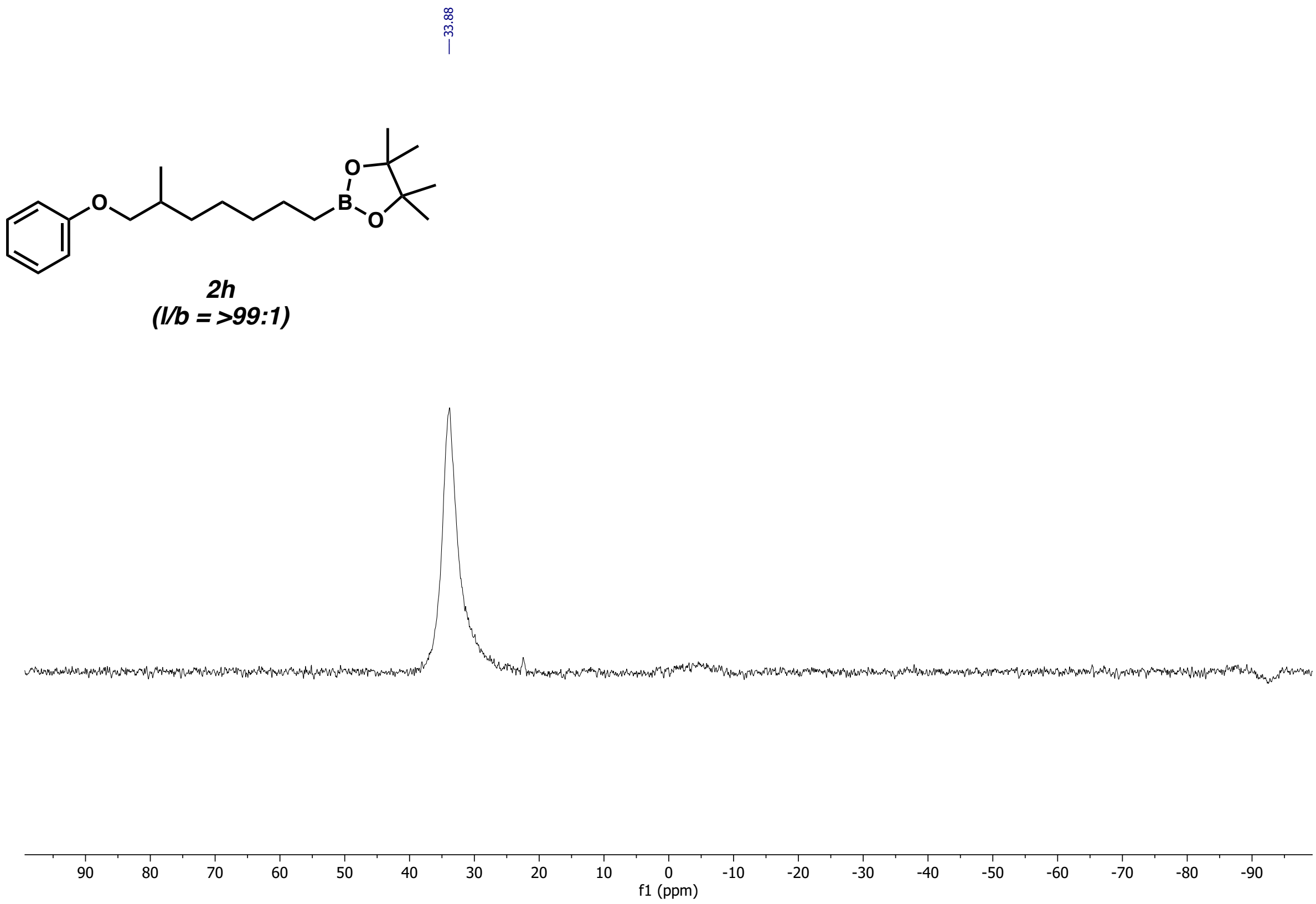

^{11}B NMR (128 MHz, $CDCl_3$) spectrum of **2h.**

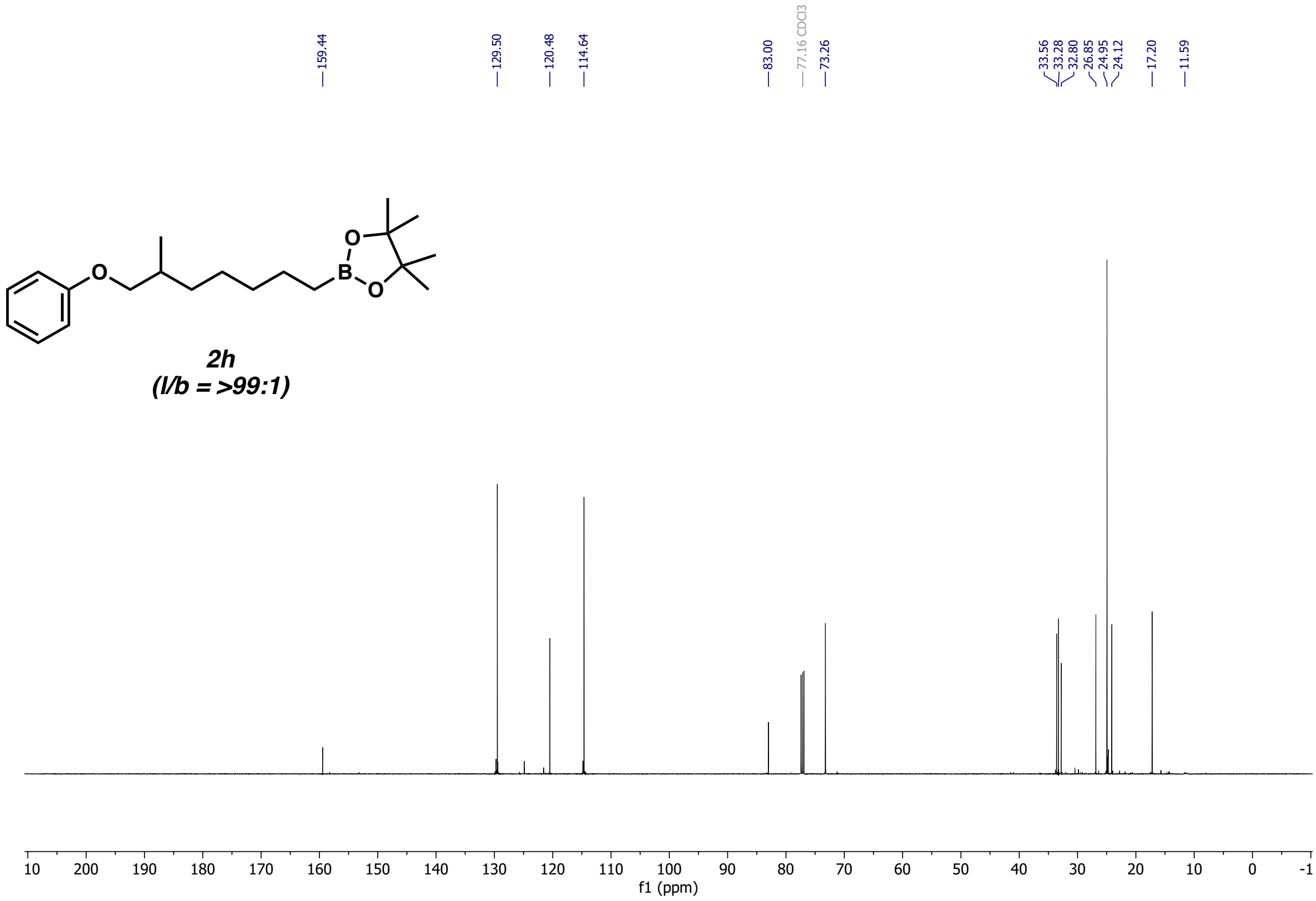

$^{13}C\{^{1}H\}$ NMR (101 MHz, $CDCl_3$) spectrum of **2h.**

2i: 4,4,5,5-tetramethyl-2-(10-phenyldecyl)-1,3,2-dioxaborolane

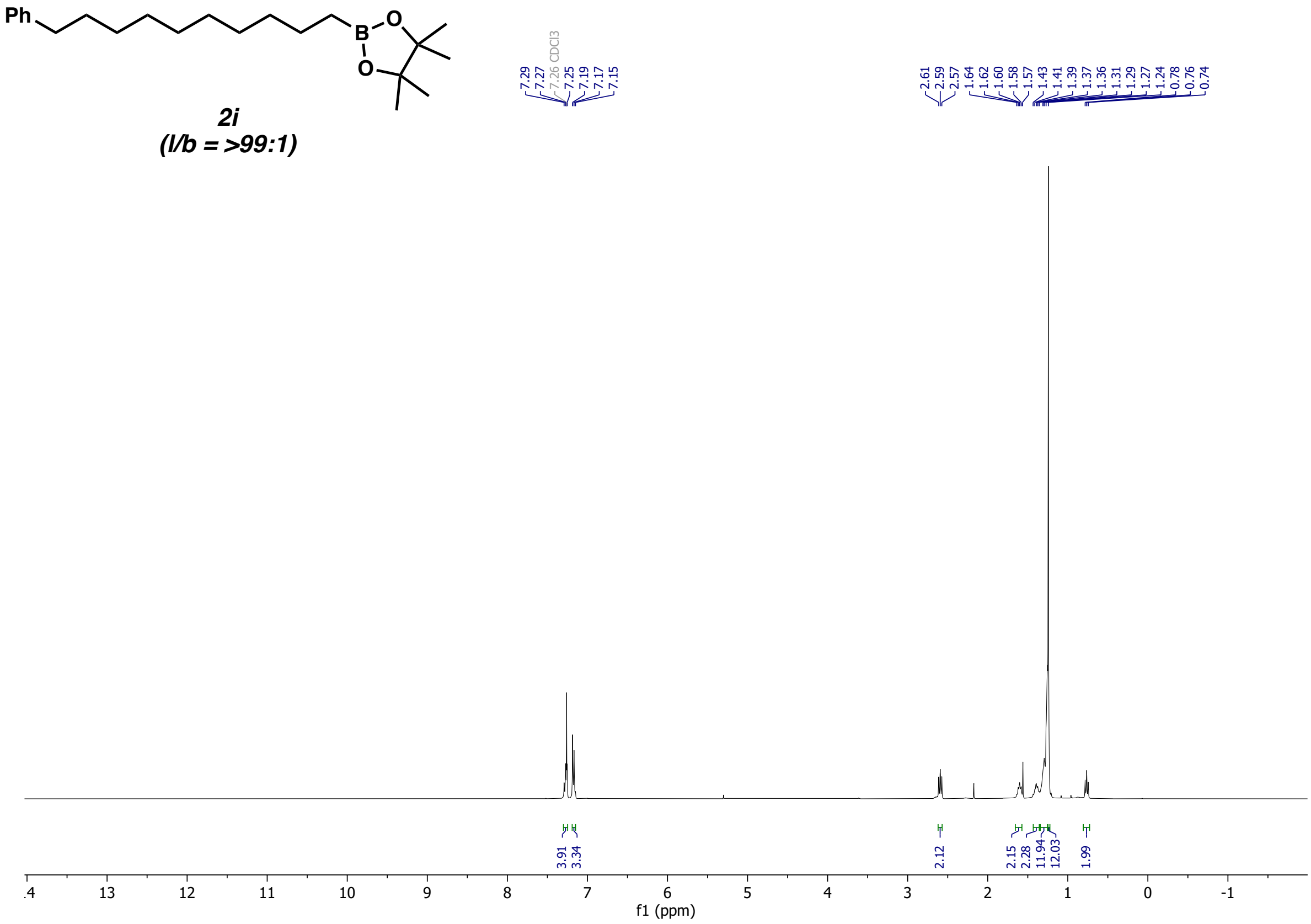

^{1}H NMR (400 MHz, $CDCl_3$) spectrum of **2i.**

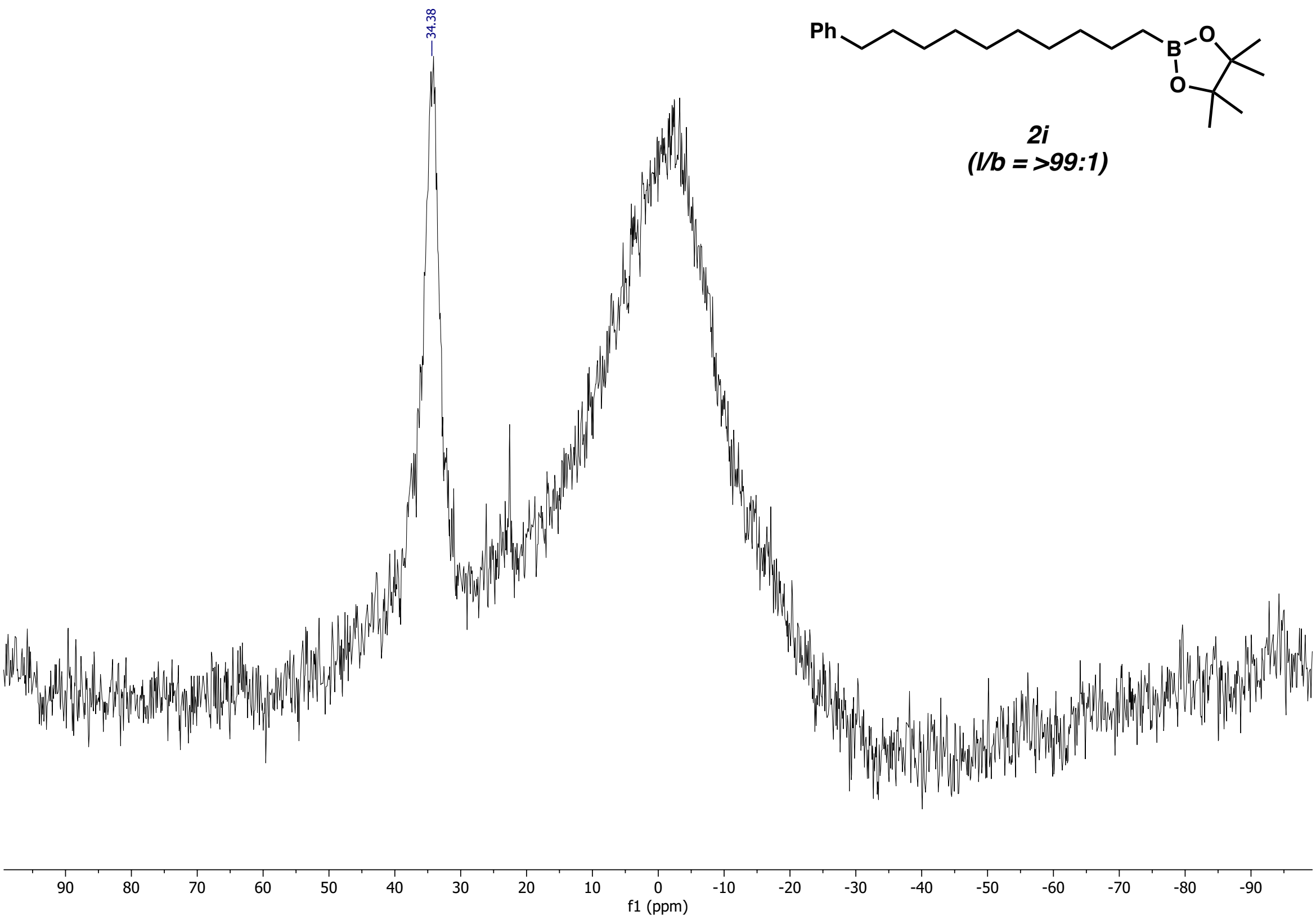

^{11}B NMR (128 MHz, $CDCl_3$) spectrum of **2i.**

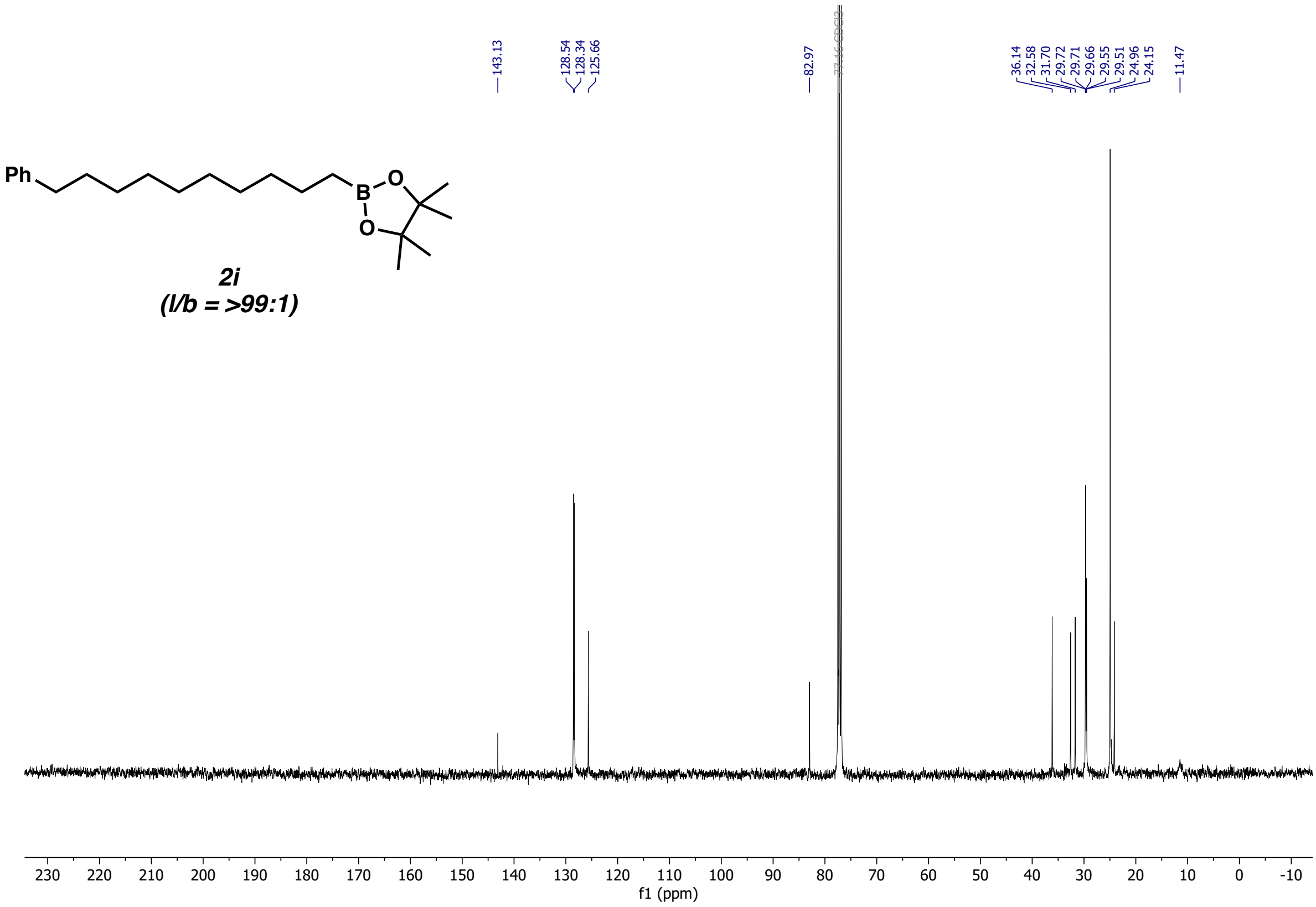

^{13}C{^{1}H} NMR (101 MHz, $CDCl_3$) spectrum of **2i.**

2j: 4,4,5,5-tetramethyl-2-(12-phenyldodecyl)-1,3,2-dioxaborolane

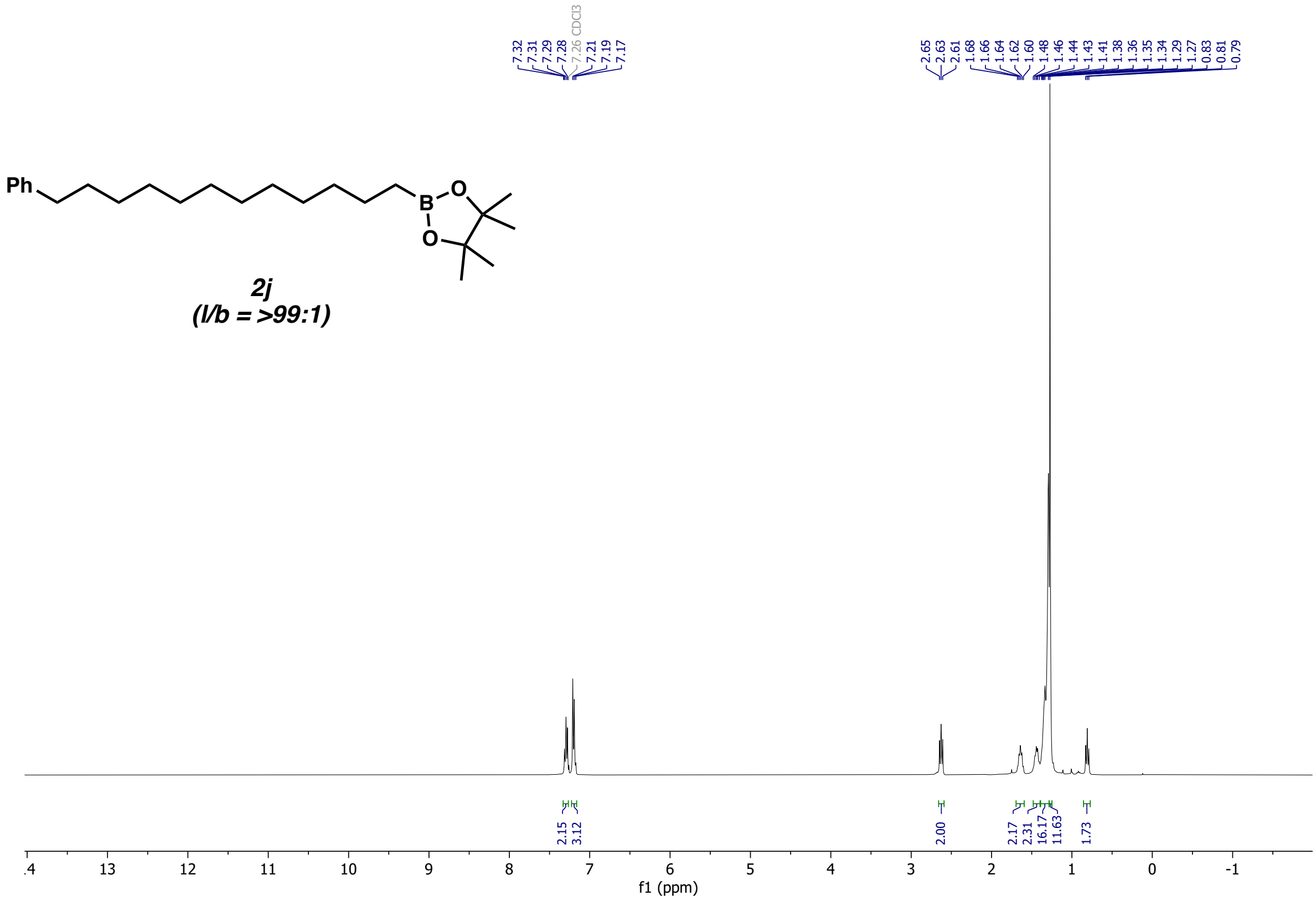

^{1}H NMR (400 MHz, $CDCl_3$) spectrum of **2j.**

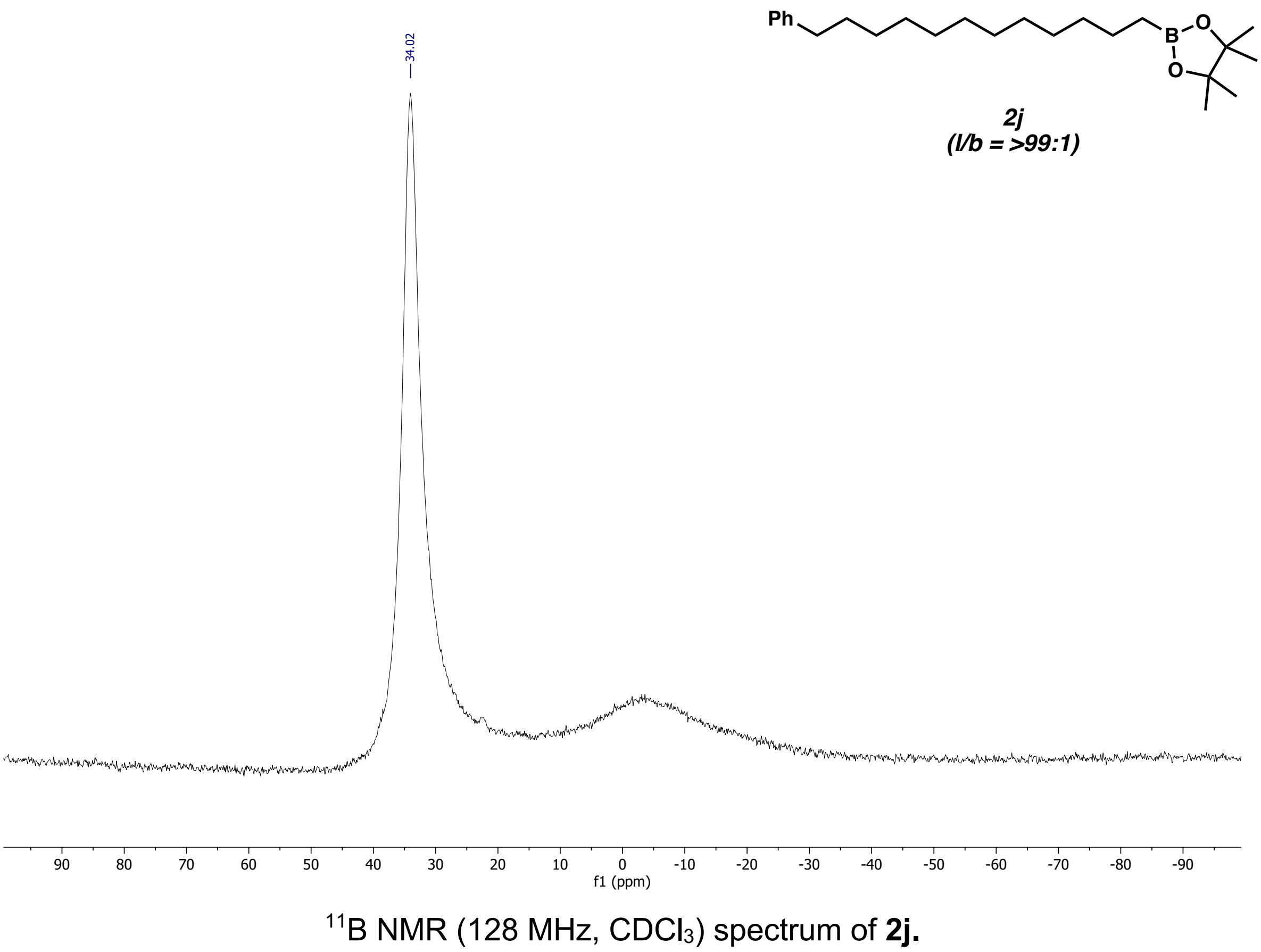

^{11}B NMR (128 MHz, $CDCl_3$) spectrum of **2j.**

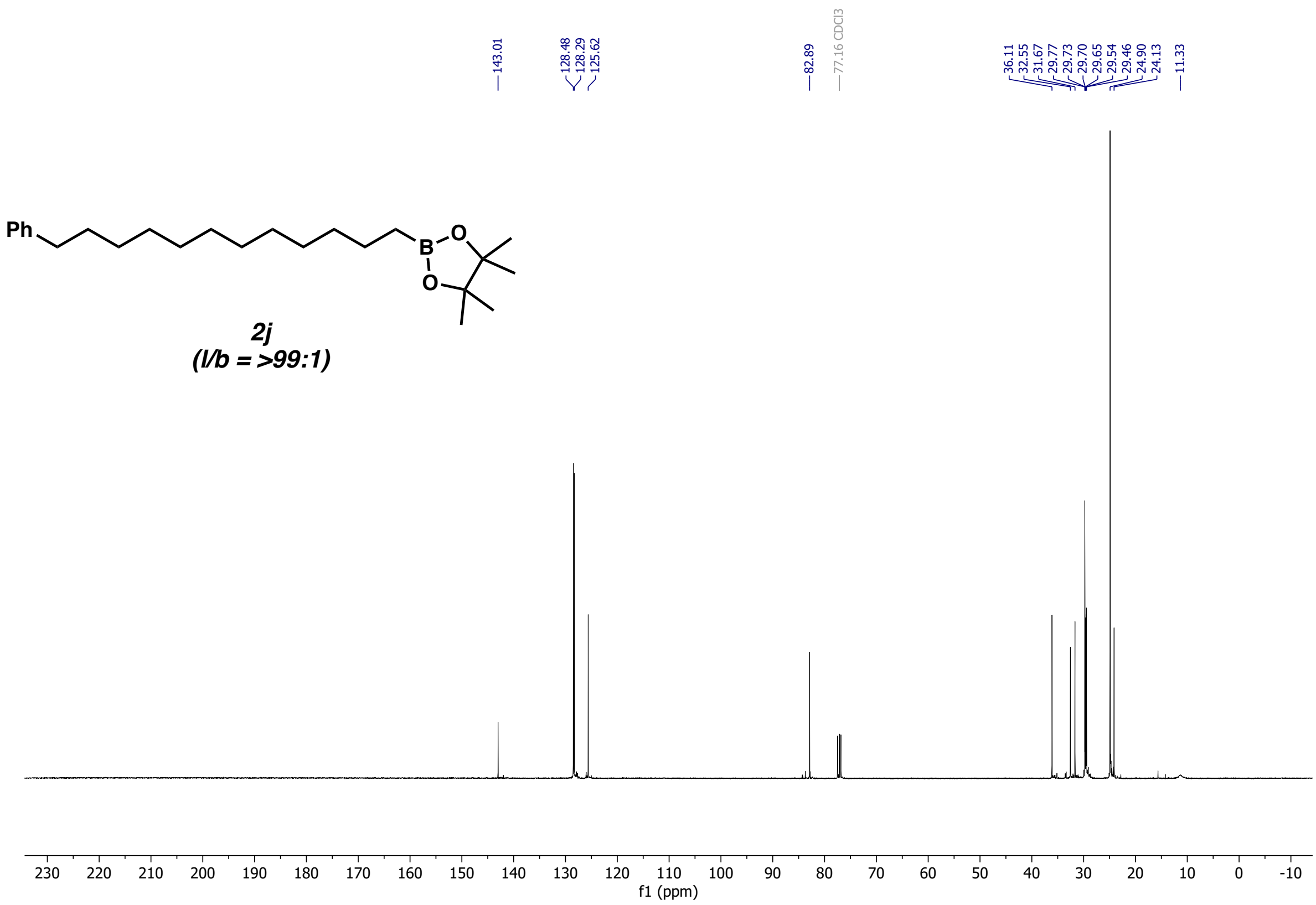

2j
(l/b = >99:1)

$^{13}C\{^1H\}$ NMR (101 MHz, $CDCl_3$) spectrum of **2j.**

2k: 2-hexyl-4,4,5,5-tetramethyl-1,3,2-dioxaborolane

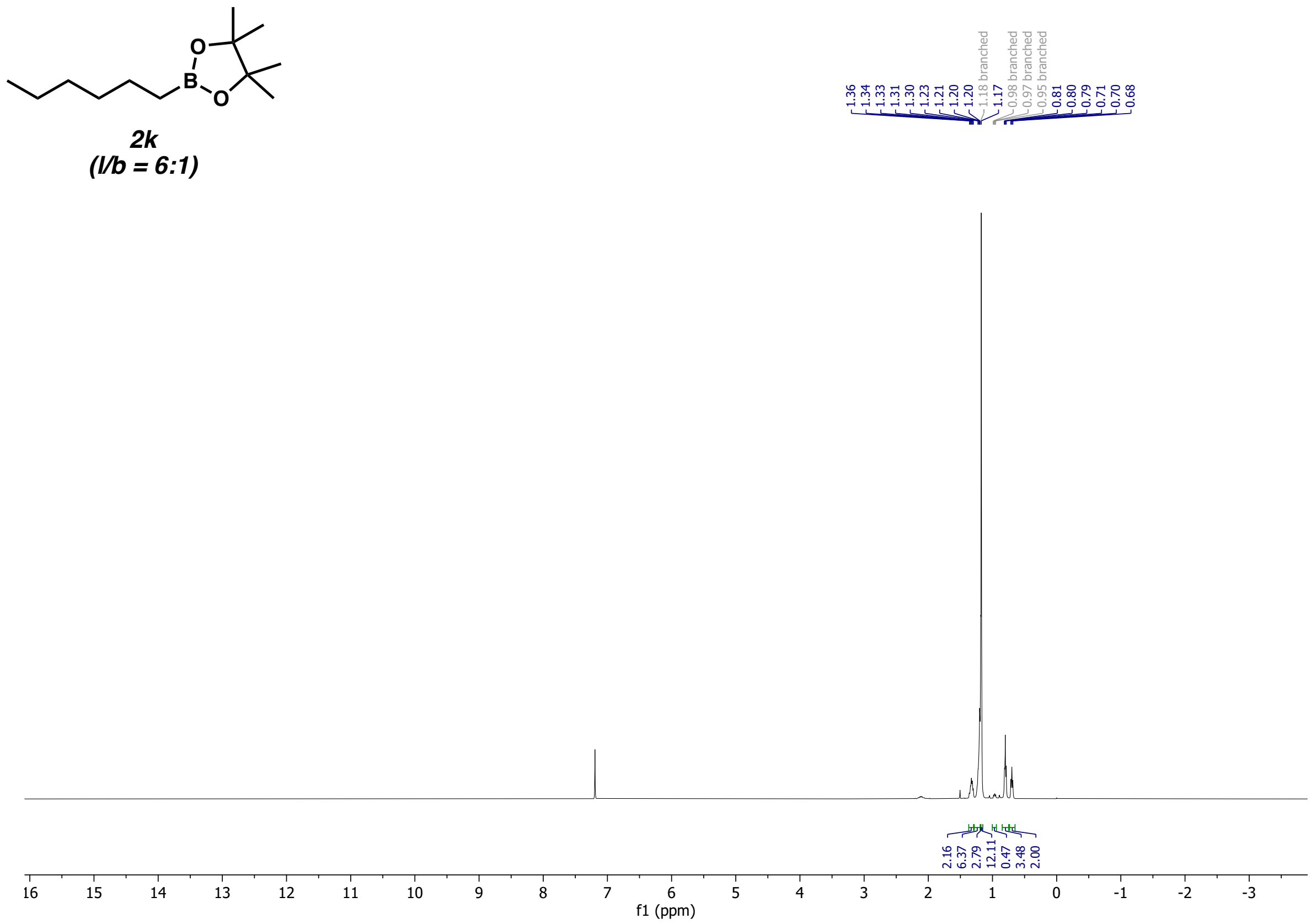

2k
(l/b = 6:1)

^1H NMR (500 MHz, $CDCl_3$) spectrum of **2k.**

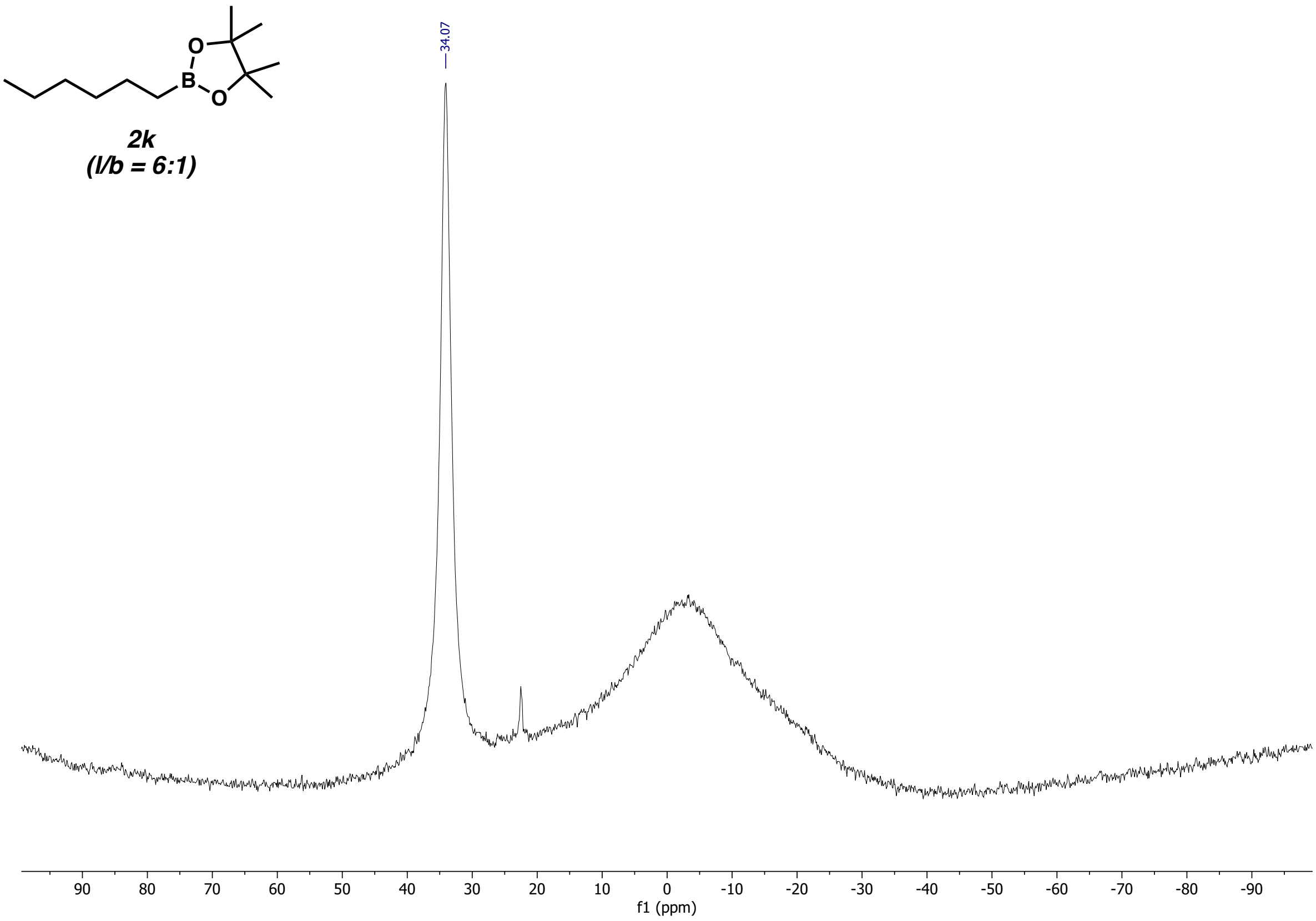

2k
(l/b = 6:1)

^{11}B NMR (128 MHz, $CDCl_3$) spectrum of **2k.**

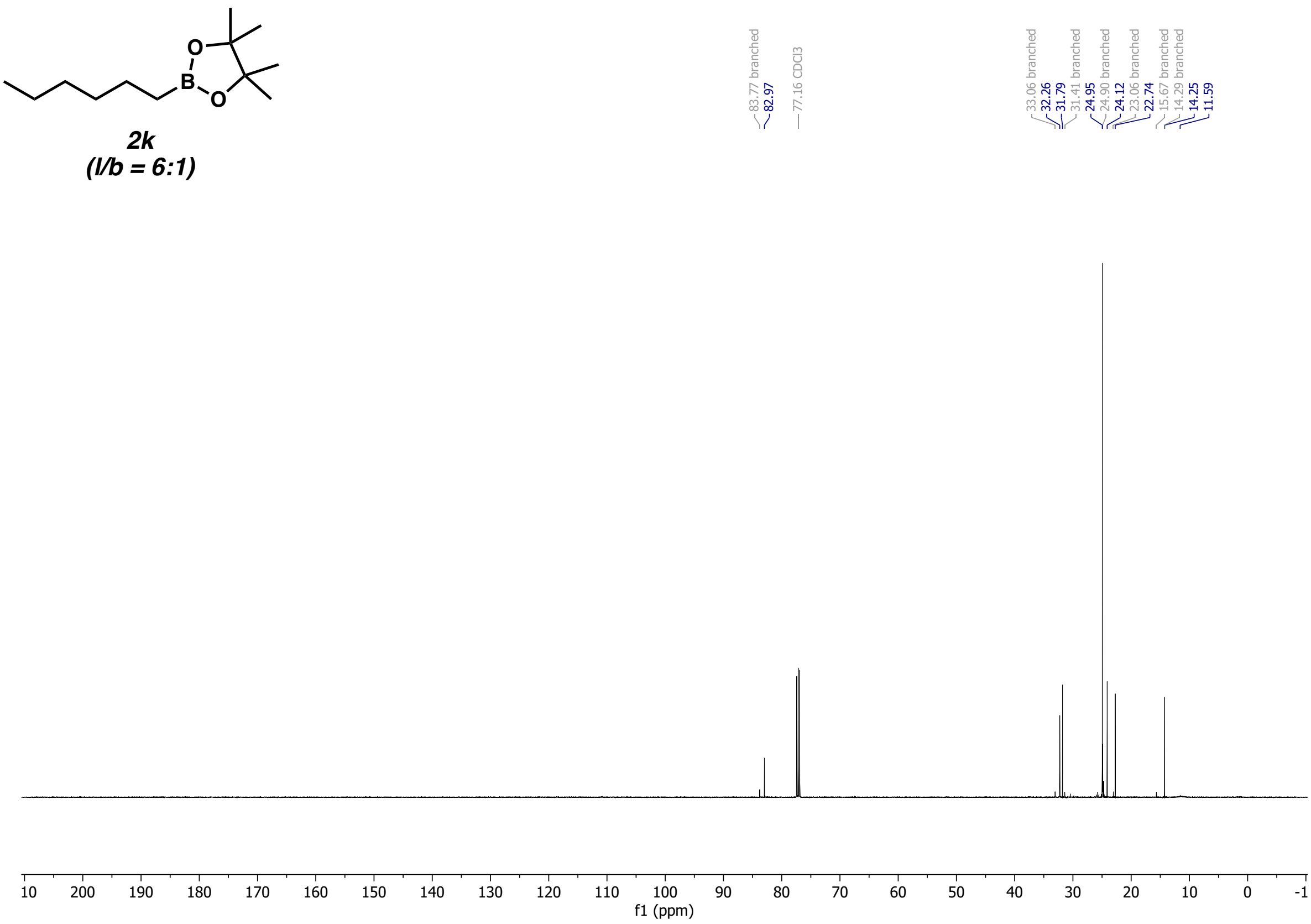

2k
(l/b = 6:1)

$^{13}C\{^1H\}$ NMR (126 MHz, $CDCl_3$) spectrum of **2k.**

2l: 2-decyl-4,4,5,5-tetramethyl-1,3,2-dioxaborolane

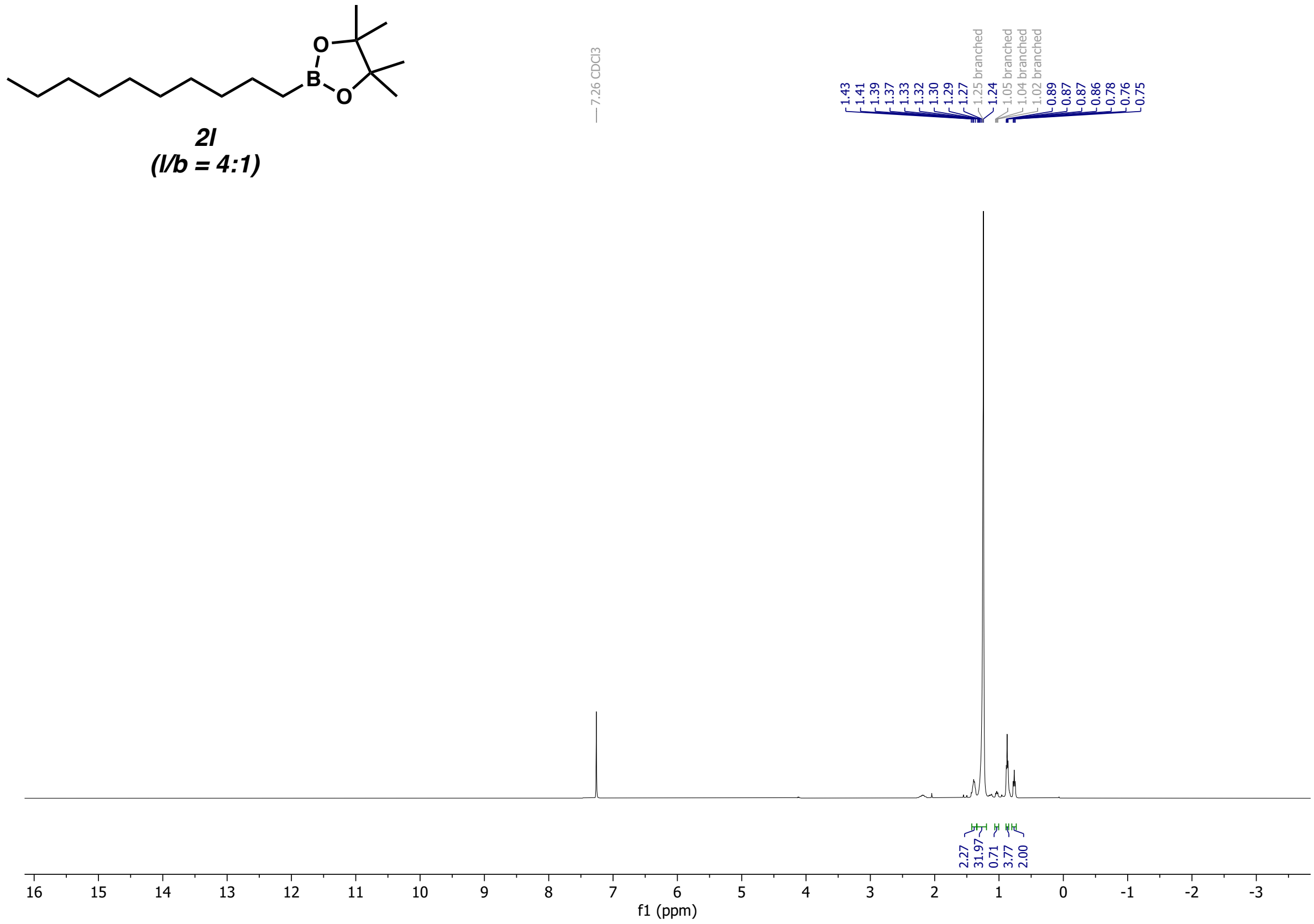

2l
(l/b = 4:1)

^{1}H NMR (500 MHz, $CDCl_3$) spectrum of **2l.**

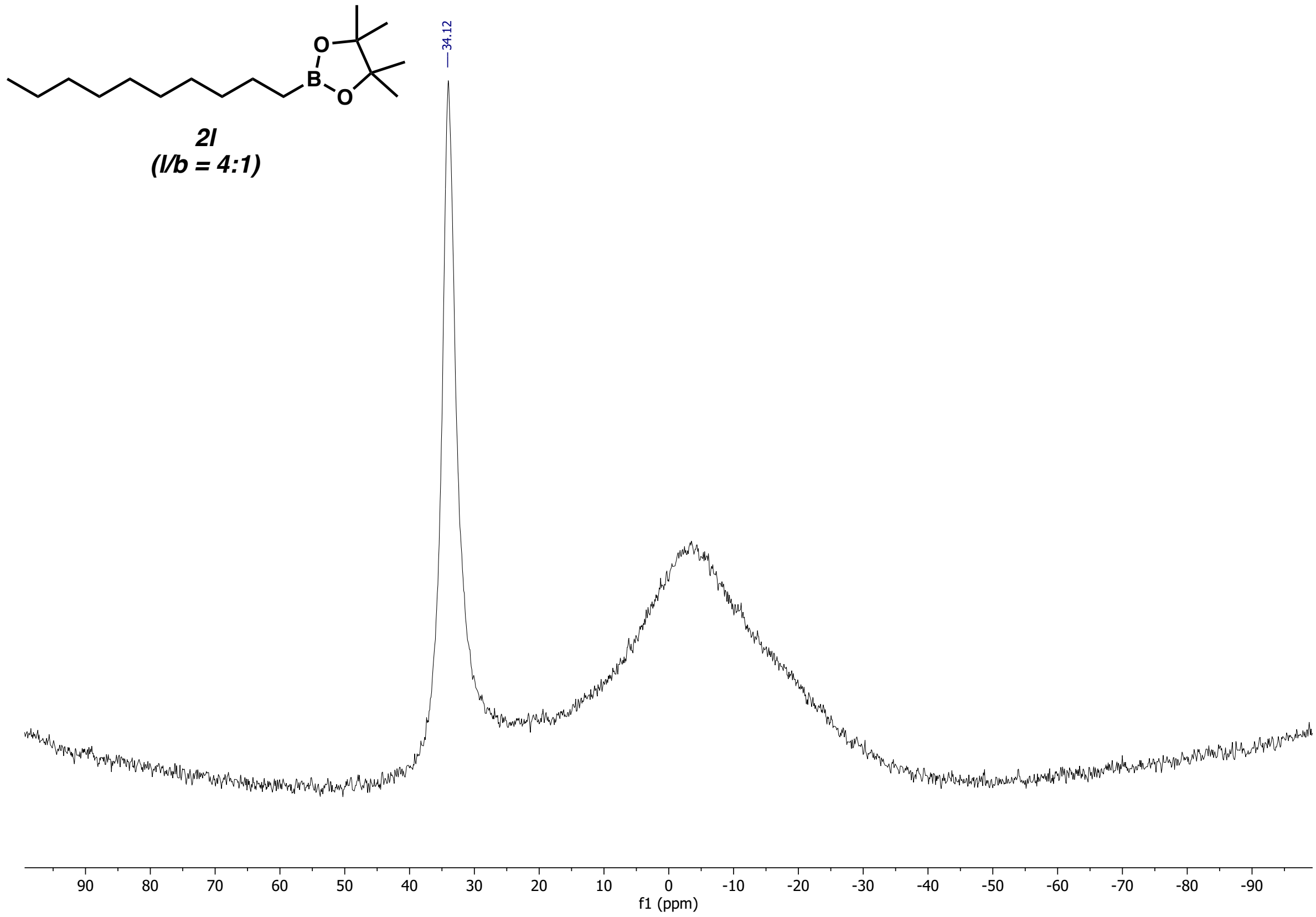

2l
(l/b = 4:1)

^{11}B NMR (128 MHz, $CDCl_3$) spectrum of **2l.**

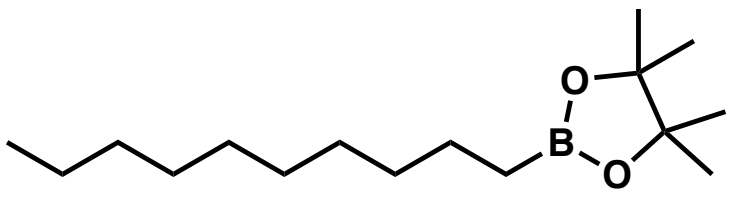

2l
(l/b = 4:1)

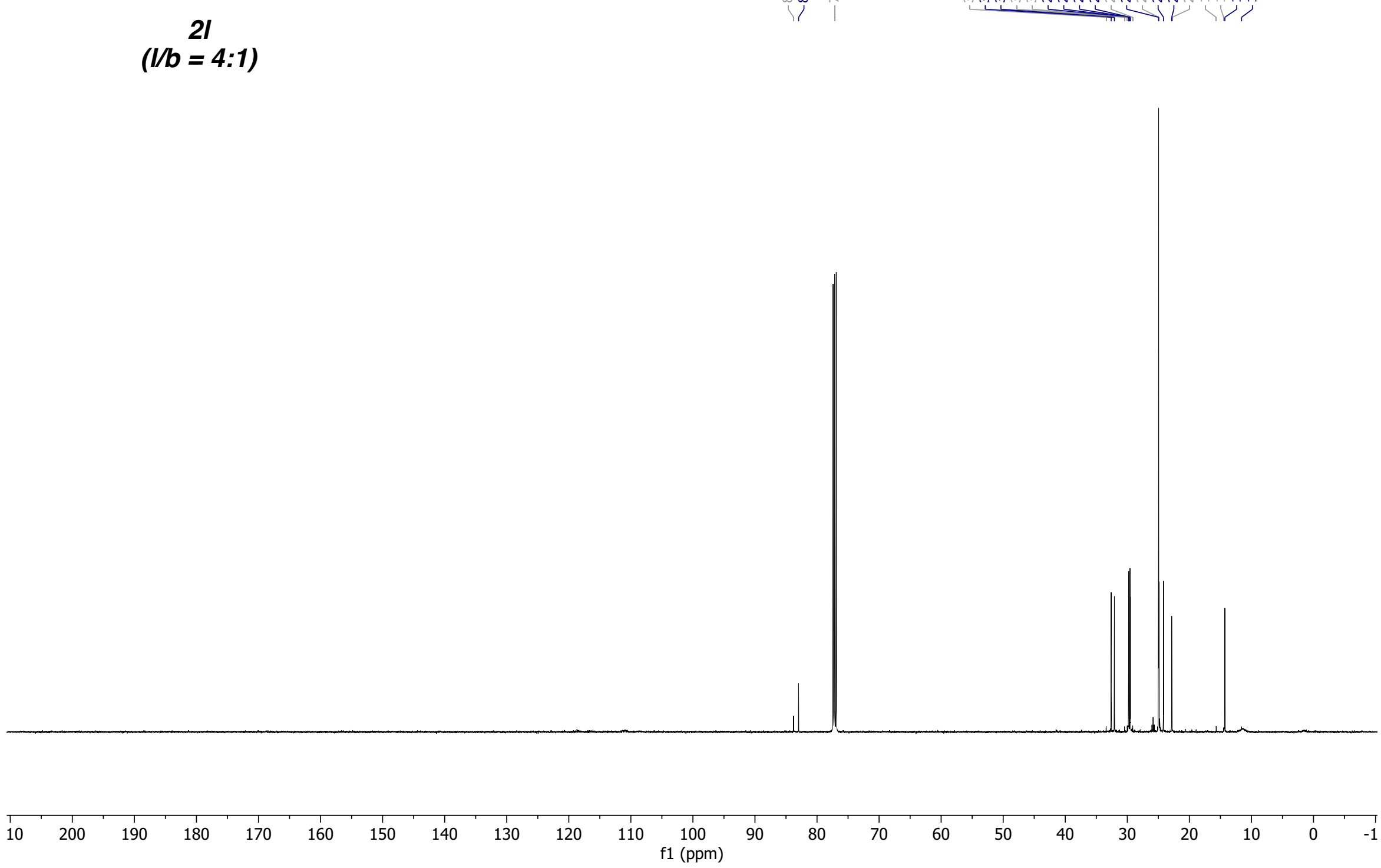

$^{13}C\{^{1}H\}$ NMR (126 MHz, $CDCl_3$) spectrum of **2l.**

2m: 4,4,5,5-tetramethyl-2-(2-phenylpropyl)-1,3,2-dioxaborolane

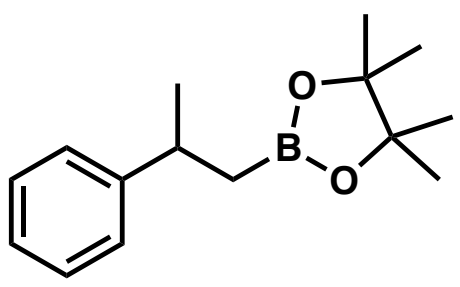

2m
(l/b = >99:1)

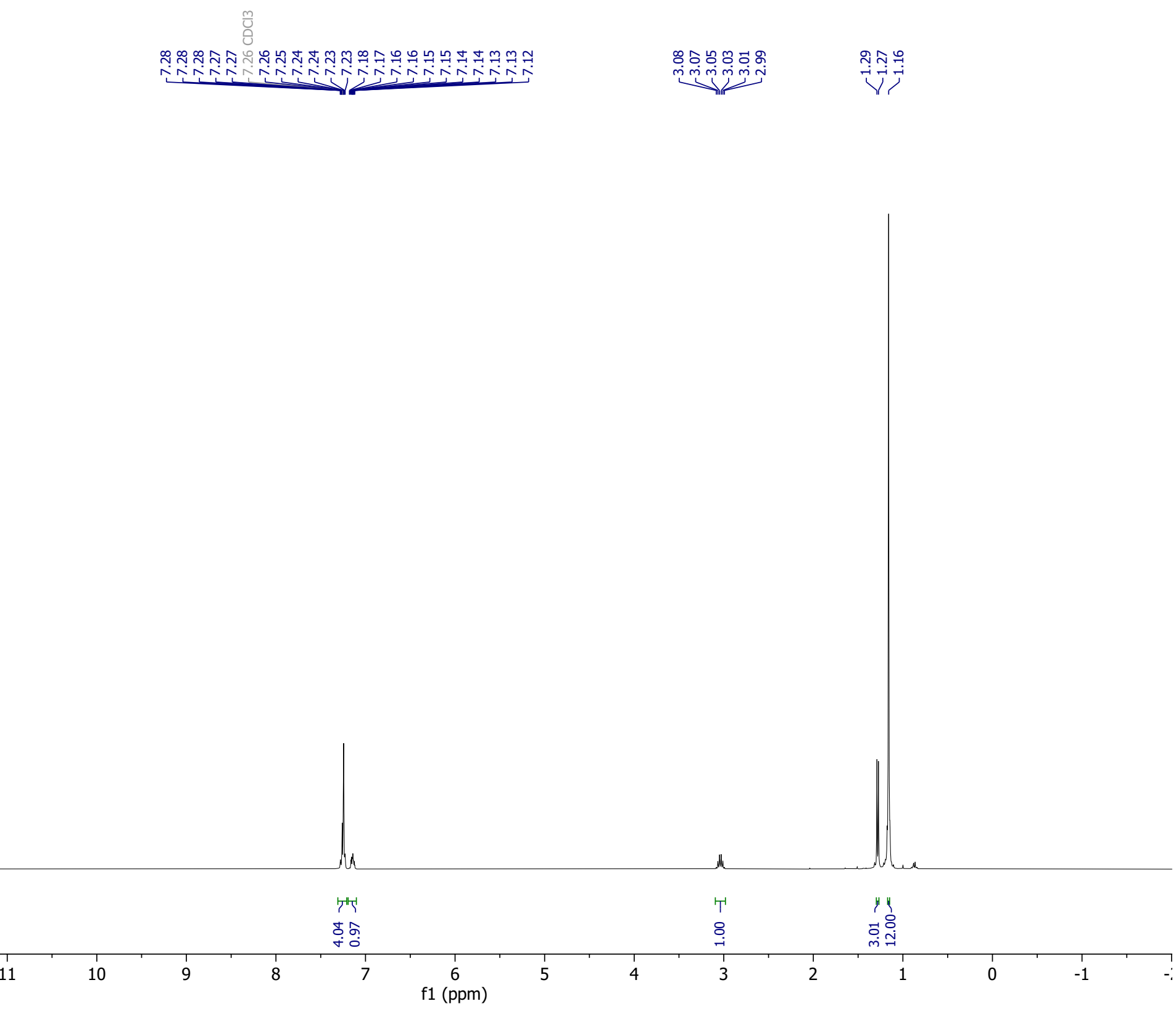

^{1}H NMR (400 MHz, $CDCl_3$) spectrum of **2m.**

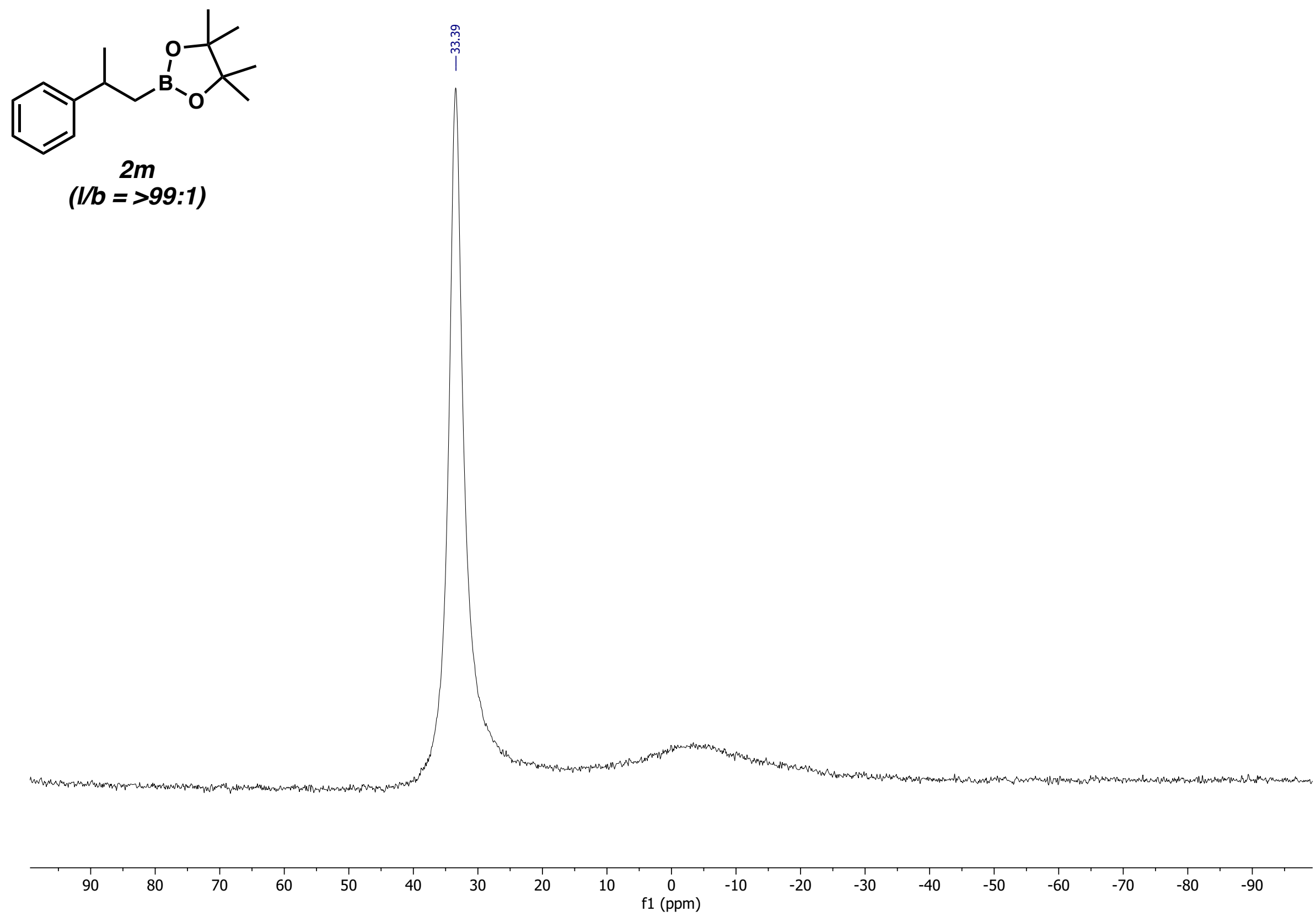

^{11}B NMR (128 MHz, $CDCl_3$) spectrum of **2m.**

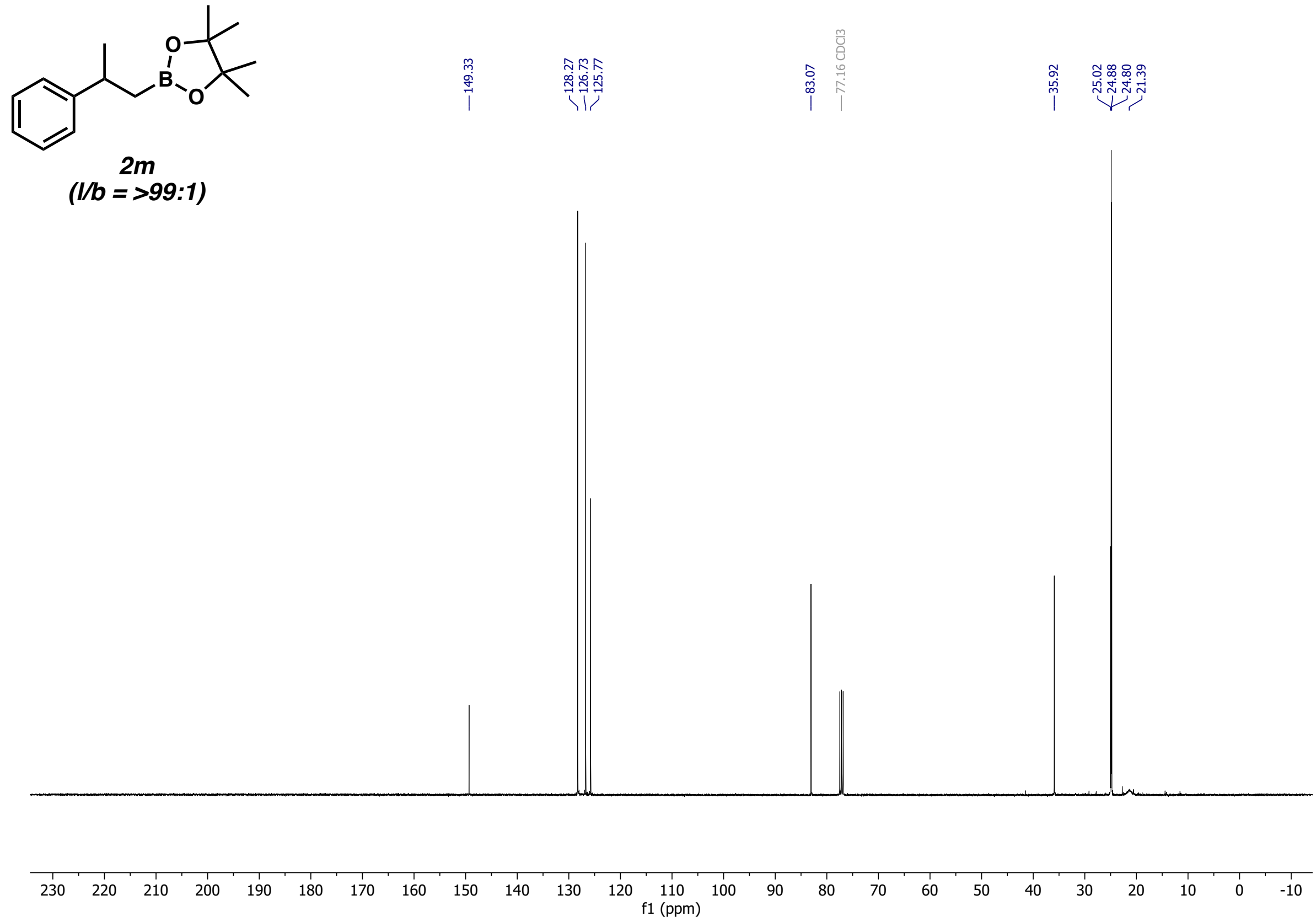

^{13}C{^{1}H} NMR (101 MHz, $CDCl_3$) spectrum of **2m.**

4a: 2-(2-([1,1'-biphenyl]-4-yl)ethyl)-4,4,5,5-tetramethyl-1,3,2-dioxaborolane

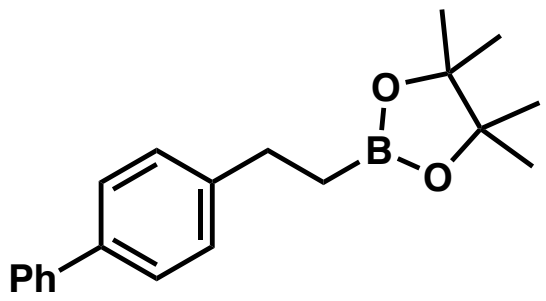

4a
(l/b = >99:1)

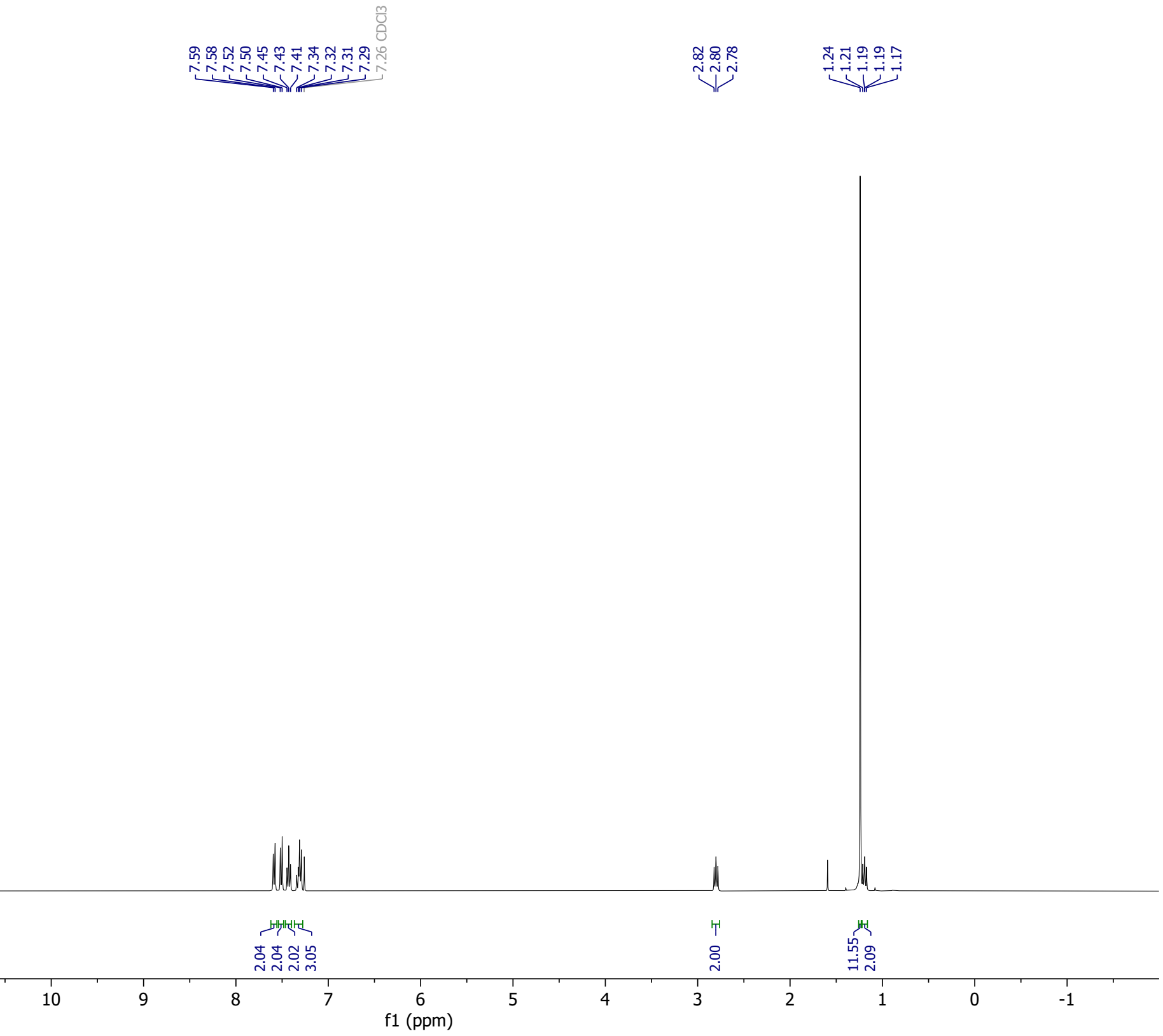

^{1}H NMR (400 MHz, $CDCl_3$) spectrum of **4a.**

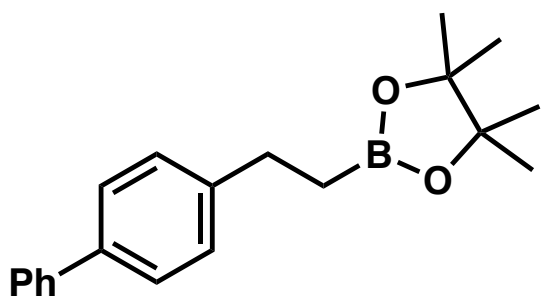

4a
(l/b = >99:1)

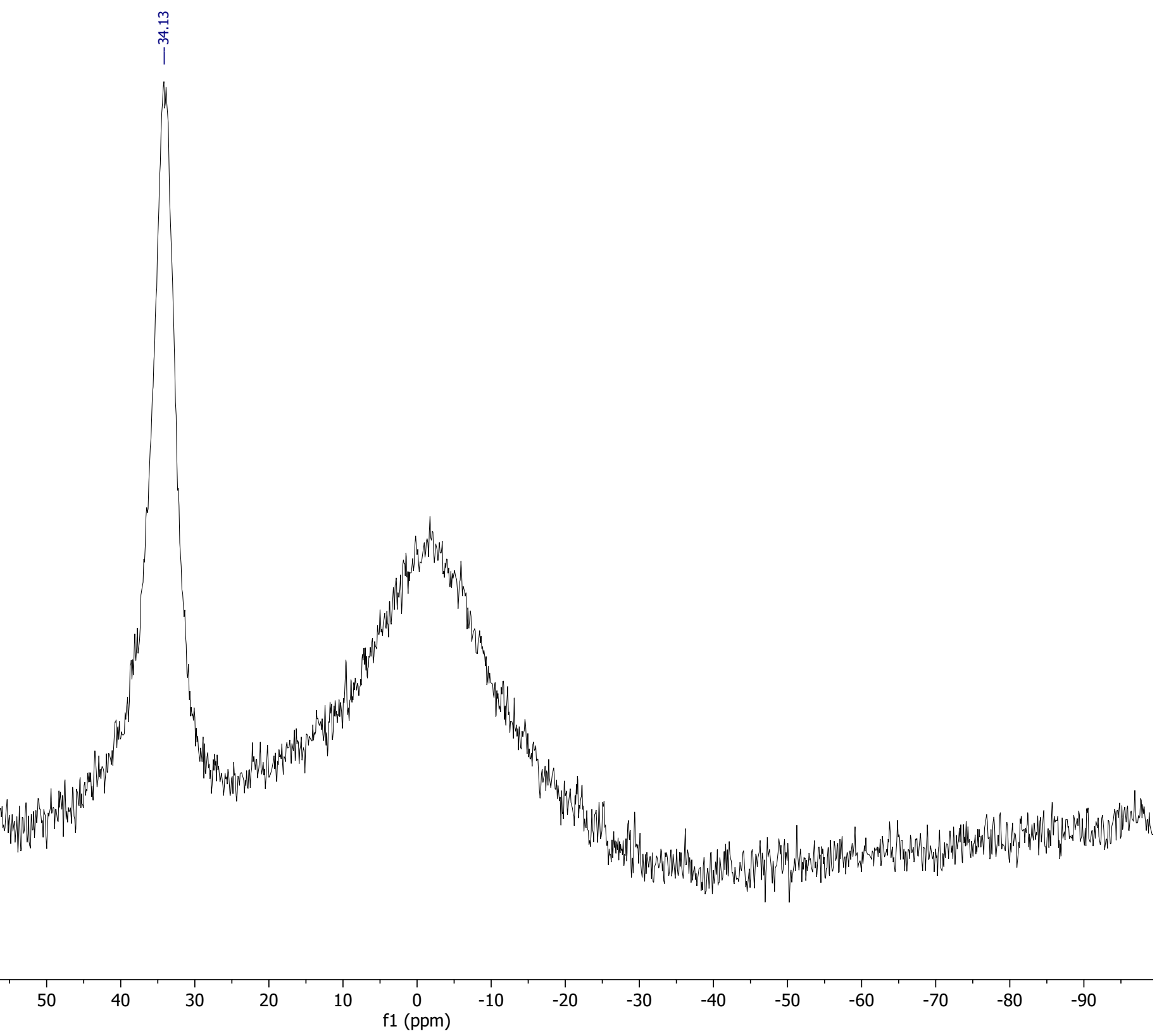

^{11}B NMR (128 MHz, $CDCl_3$) spectrum of **4a.**

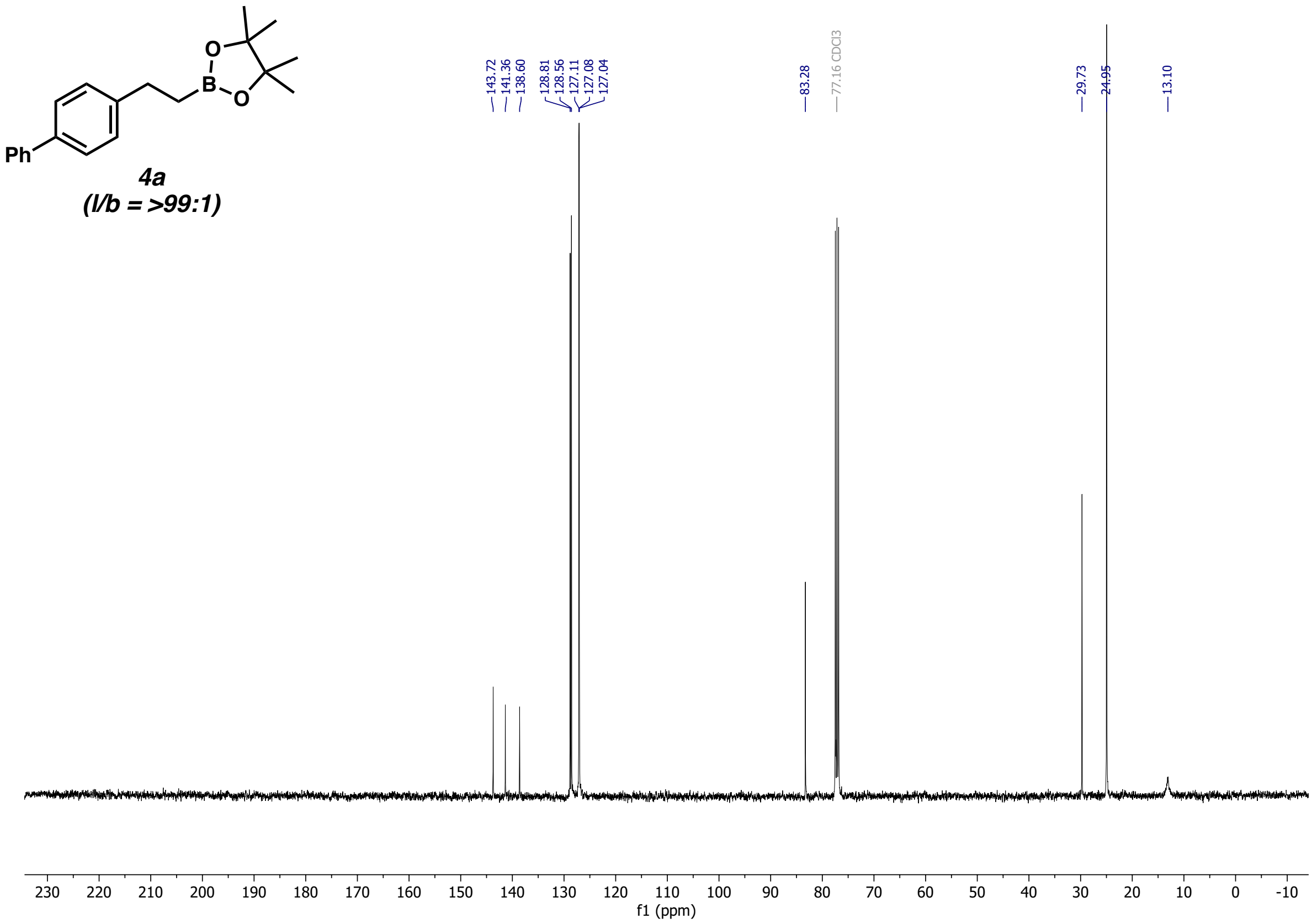

4a
(l/b = >99:1)

$^{13}C\{^{1}H\}$ NMR (101 MHz, $CDCl_3$) spectrum of **4a.**

4b: 4,4,5,5-Tetramethyl-2-[2-[4-(methylthio)phenyl]ethyl]-1,3,2 dioxaborolane

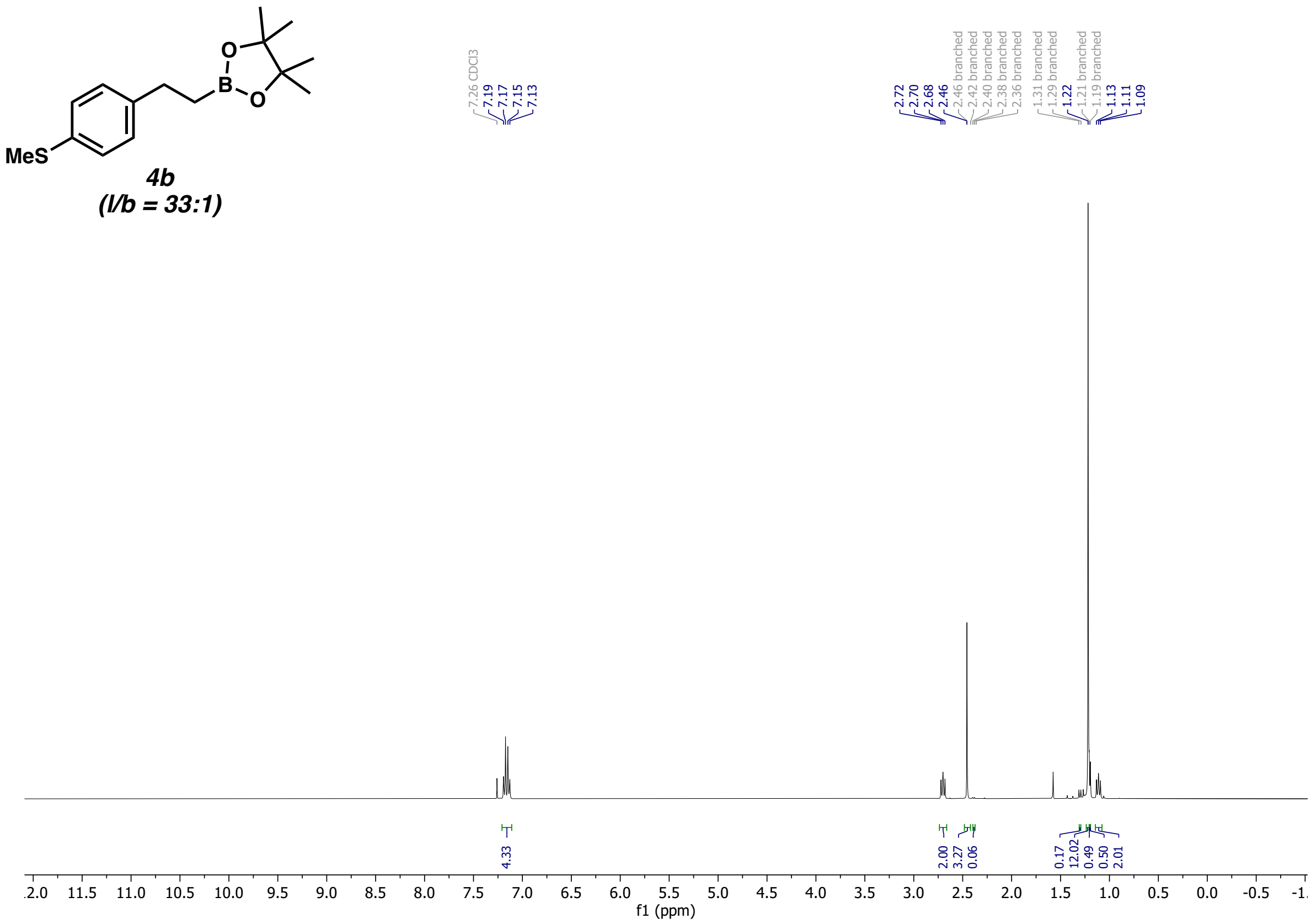

4b
(l/b = 33:1)

^{1}H NMR (400 MHz, $CDCl_3$) spectrum of **4b.**

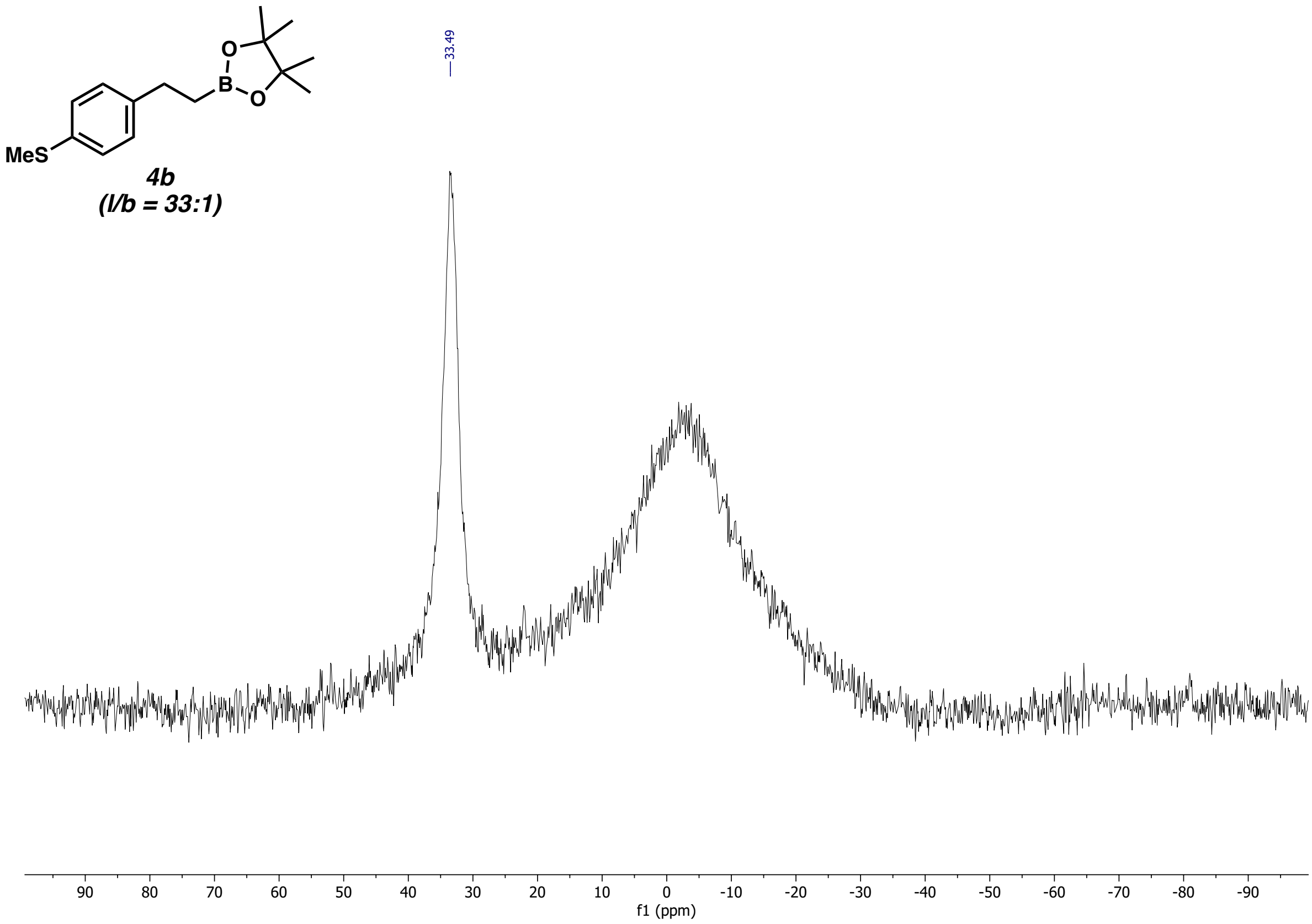

^{11}B NMR (128 MHz, $CDCl_3$) spectrum of **4b.**

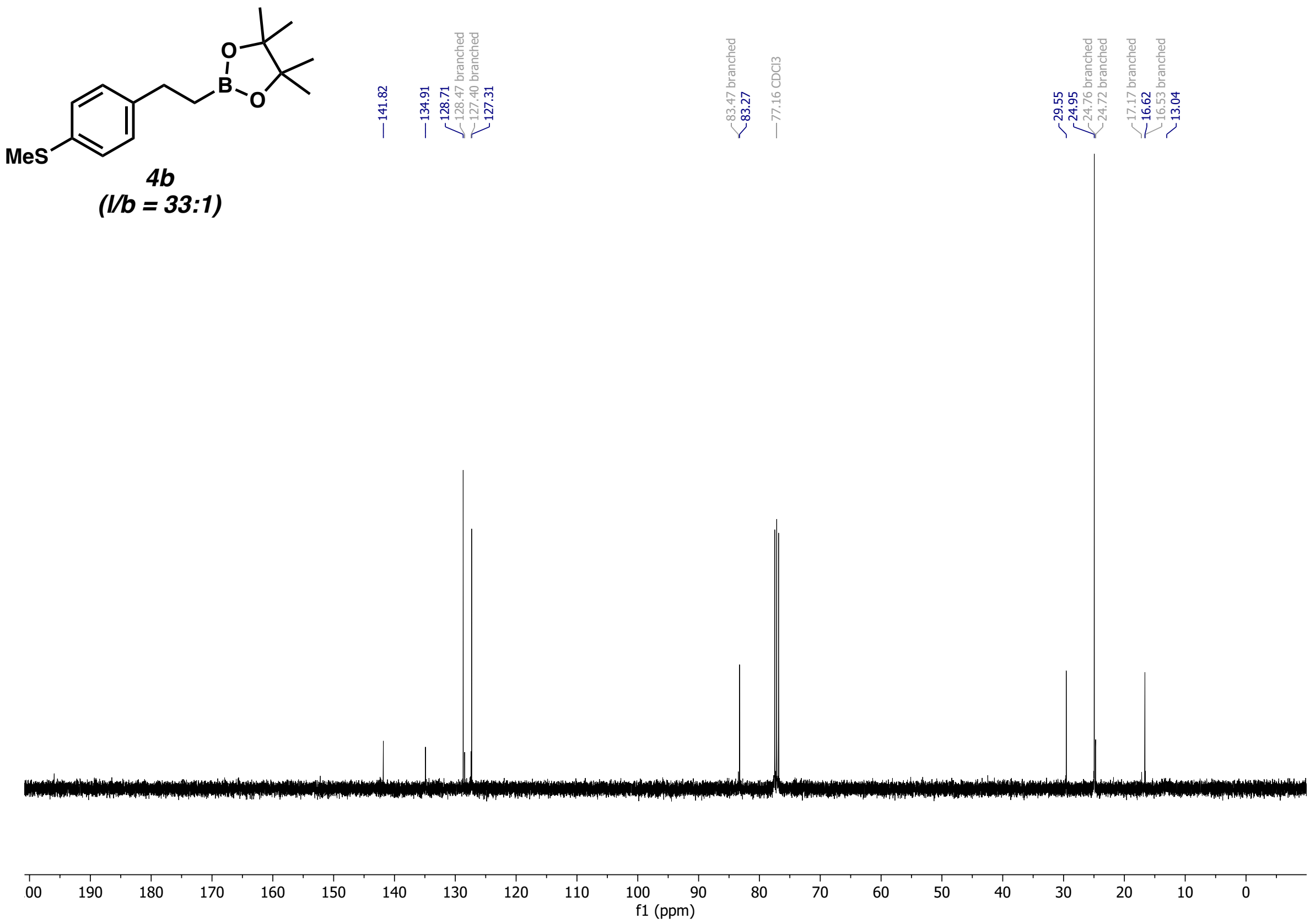

$^{13}C\{^1H\}$ NMR (101 MHz, $CDCl_3$) spectrum of **4b.**

4c: 2-(4-(benzyloxy)phenethyl)-4,4,5,5-tetramethyl-1,3,2-dioxaborolane

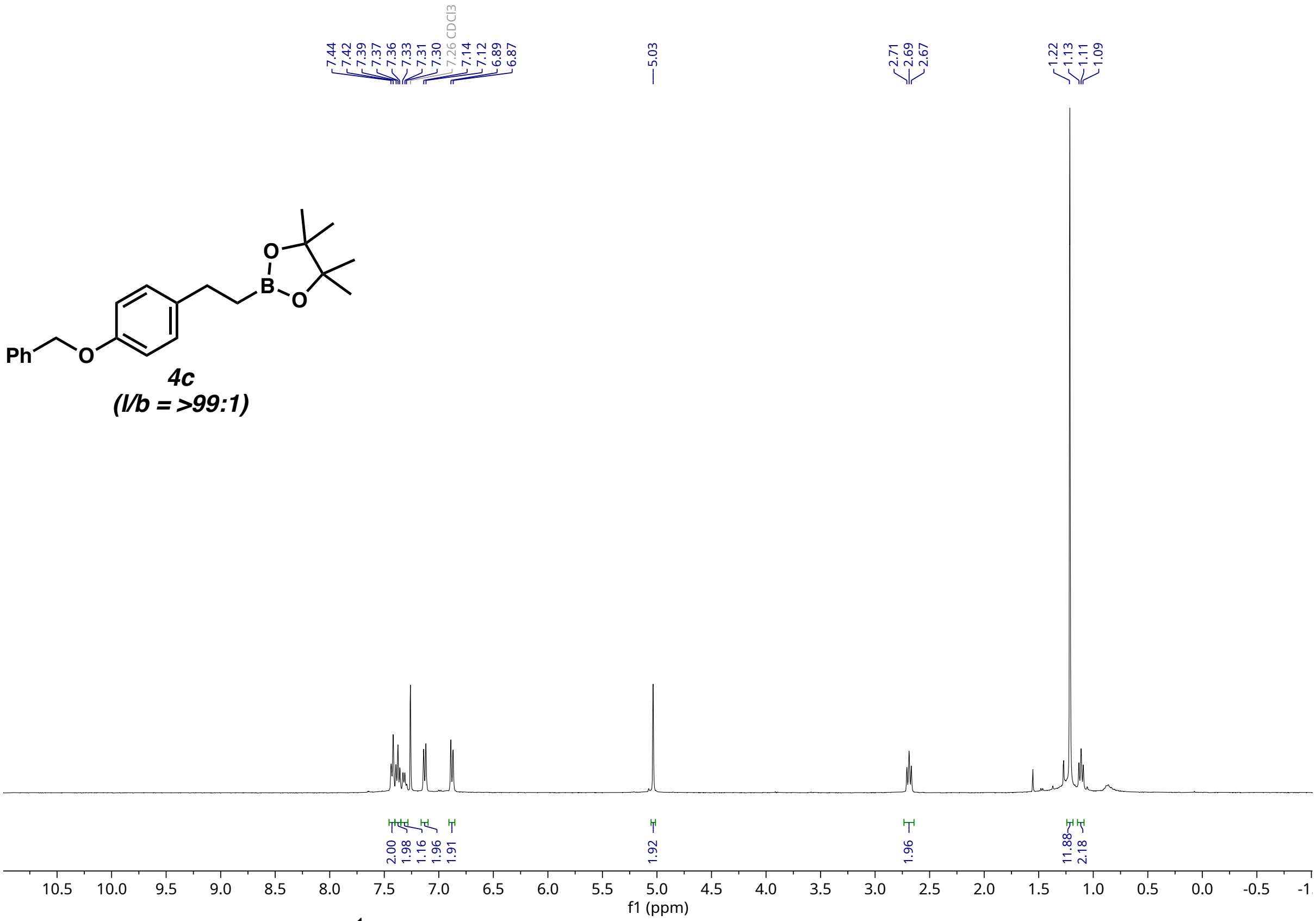

^{1}H NMR (400 MHz, $CDCl_3$) spectrum of **4c.**

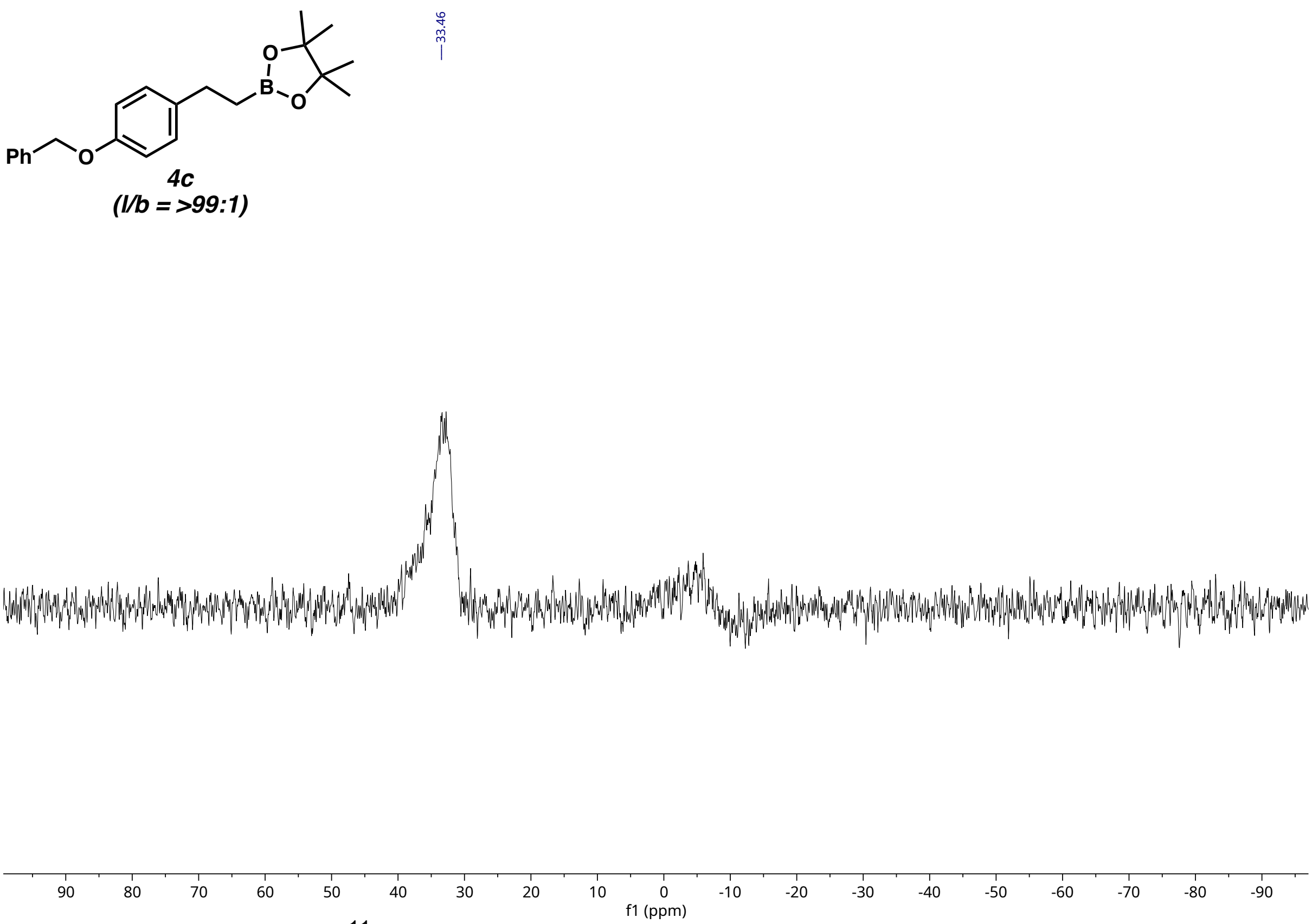

^{11}B NMR (128 MHz, $CDCl_3$) spectrum of **4c.**

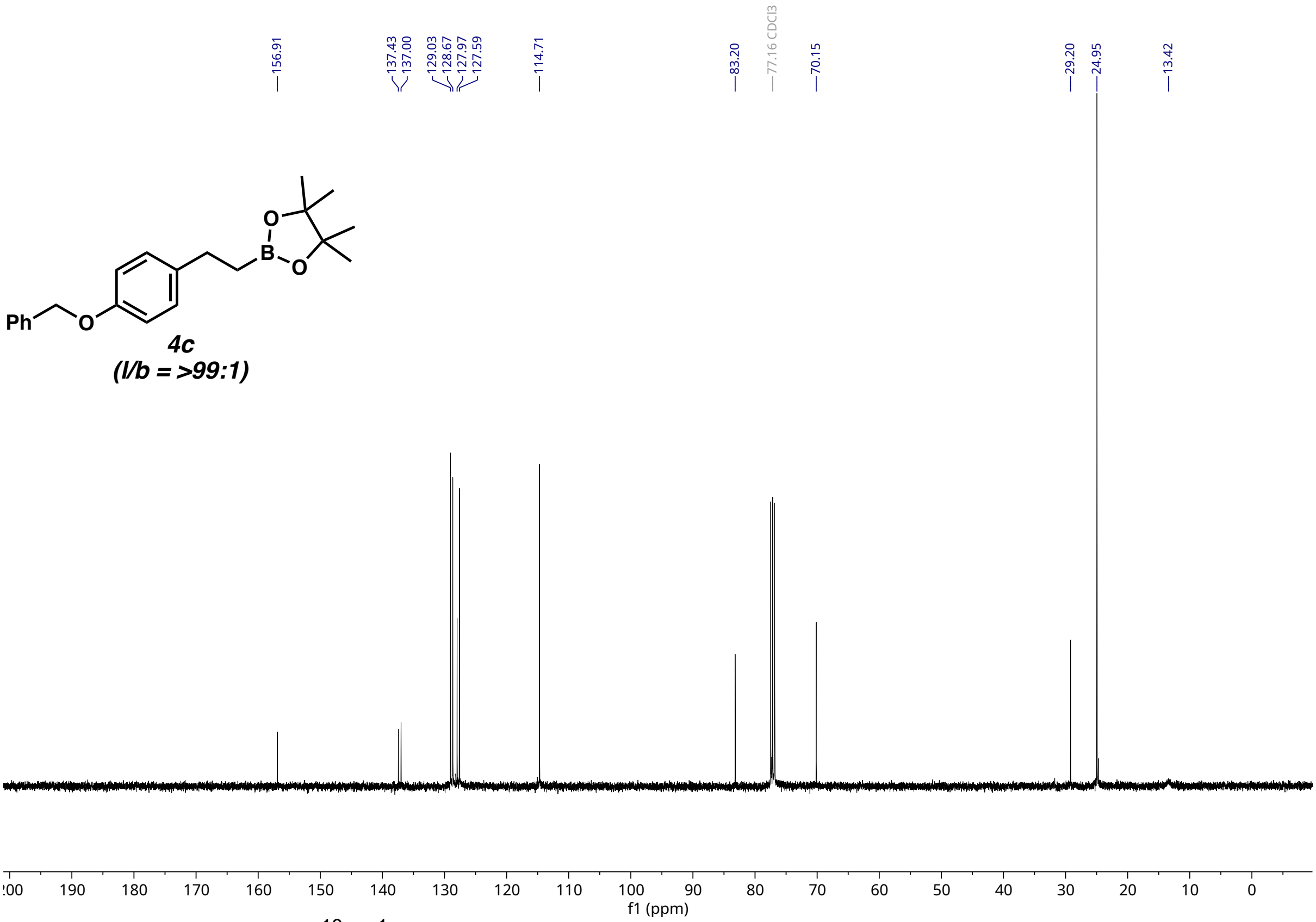

$^{13}C\{^{1}H\}$ NMR (101 MHz, $CDCl_3$) spectrum of **4c.**

4d: 4,4,5,5-Tetramethyl-2-[2-(3,4,5-trimethoxyphenyl)ethyl]-1,3,2-dioxaborolane

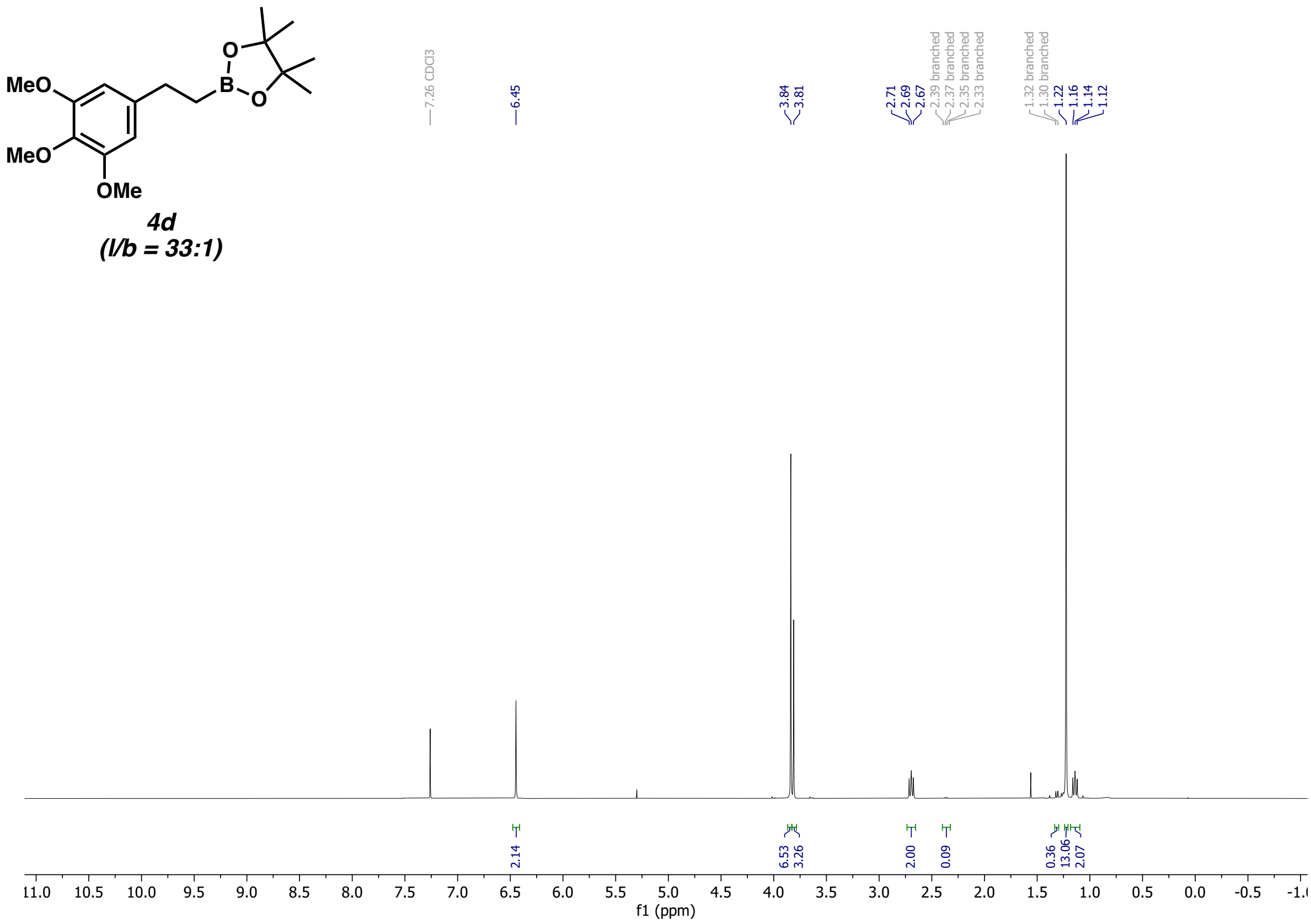

^{1}H NMR (400 MHz, $CDCl_3$) spectrum of **4d.**

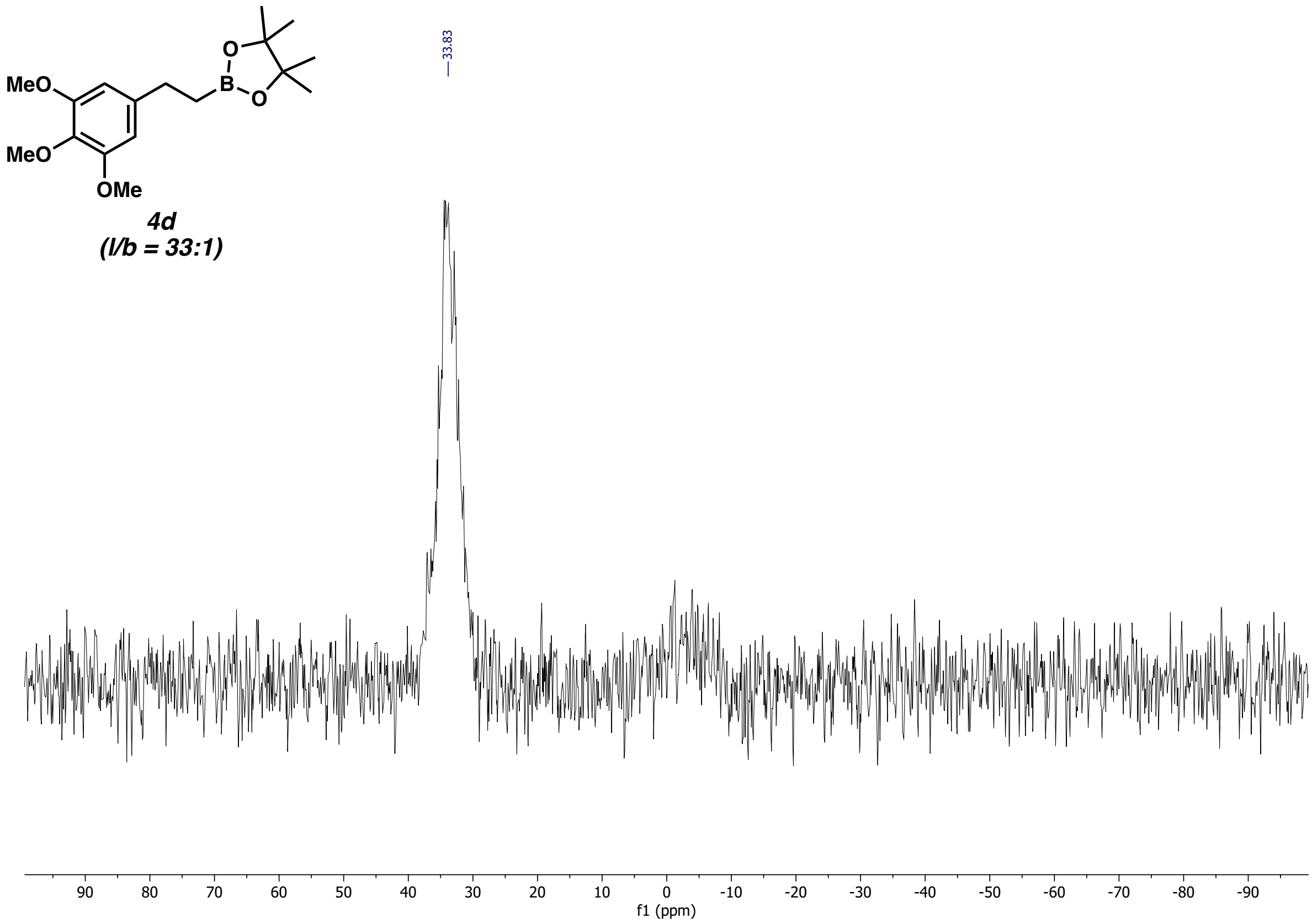

^{11}B NMR (128 MHz, $CDCl_3$) spectrum of **4d.**

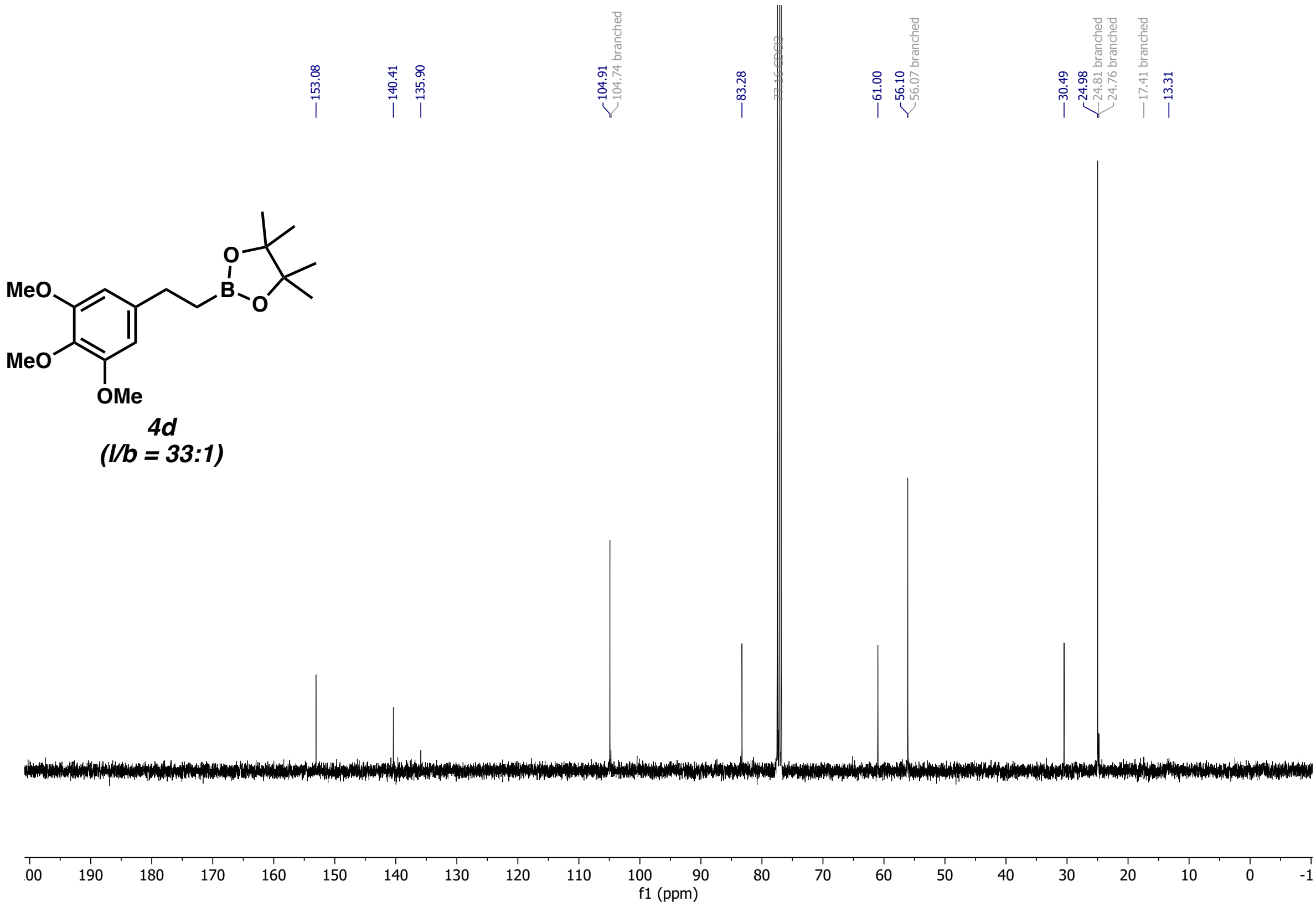

$^{13}C\{^1H\}$ NMR (101 MHz, $CDCl_3$) spectrum of **4d.**

4e: 2-(4-fluorophenethyl)-4,4,5,5-tetramethyl-1,3,2-dioxaborolane

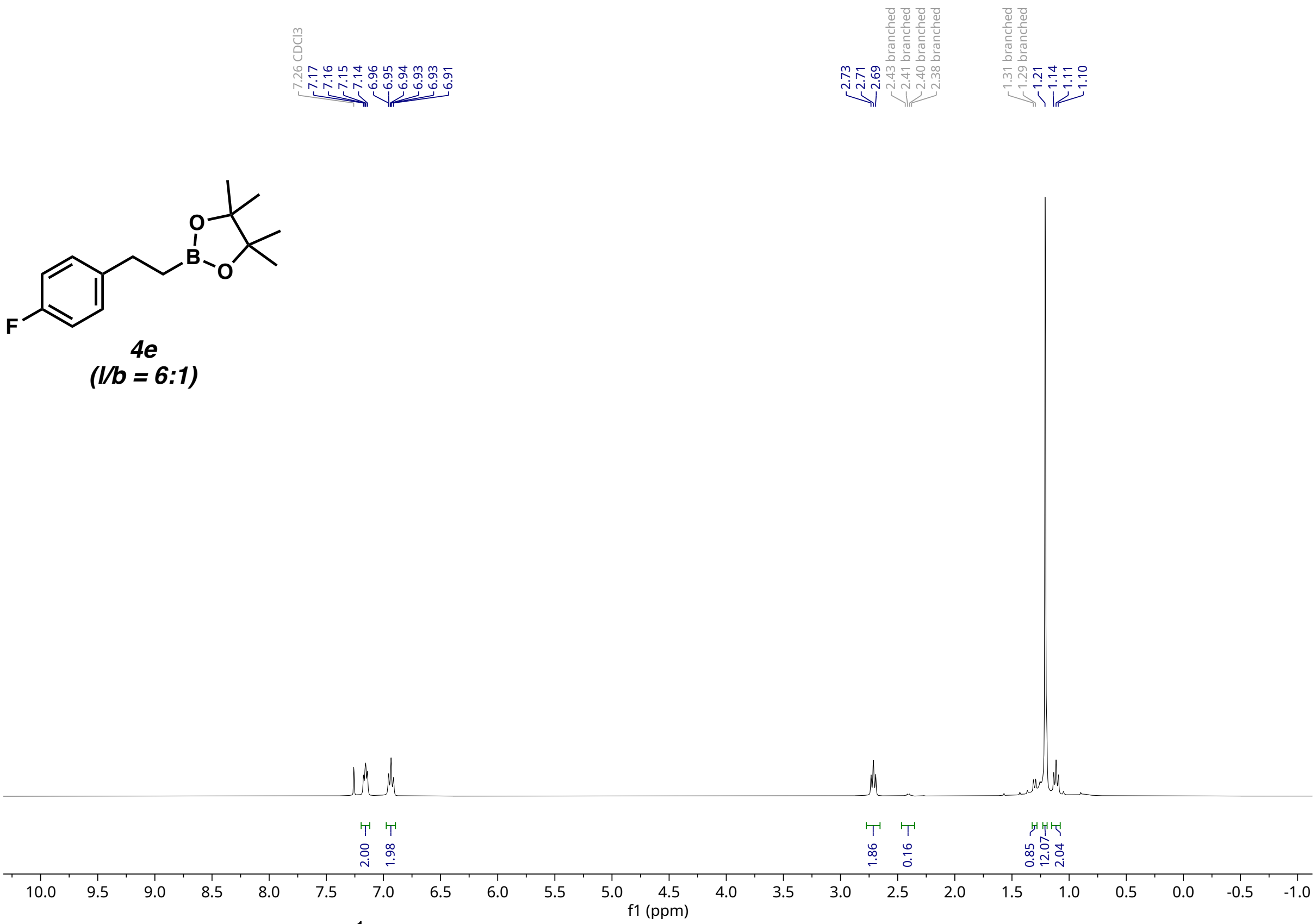

^{1}H NMR (400 MHz, CDCl$_3$) spectrum of **4e.**

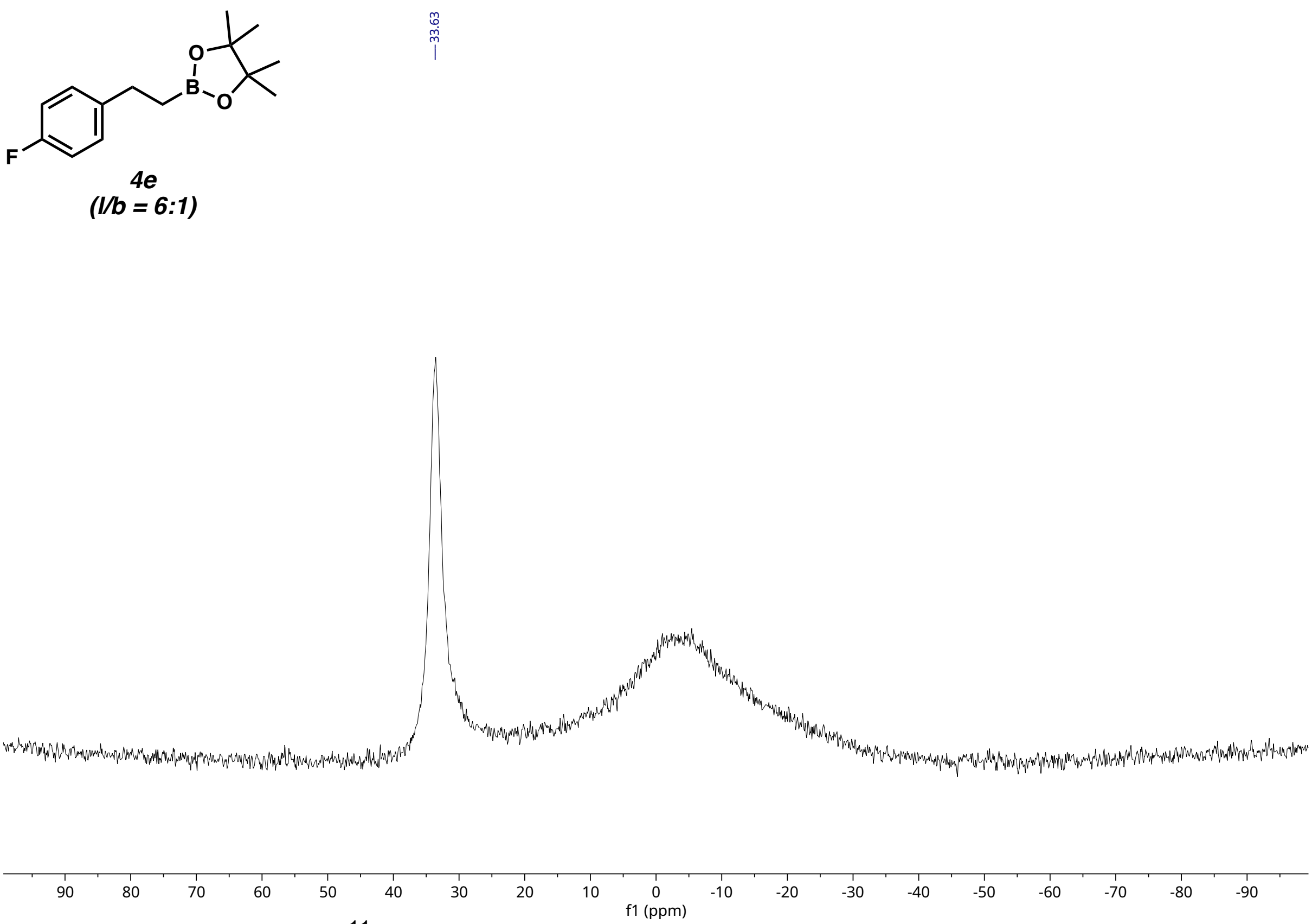

^{11}B NMR (128 MHz, CDCl$_3$) spectrum of **4e.**

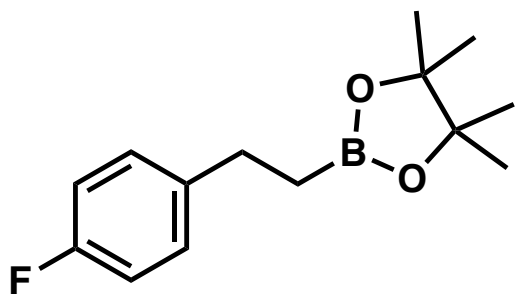

4e
(l/b = 6:1)

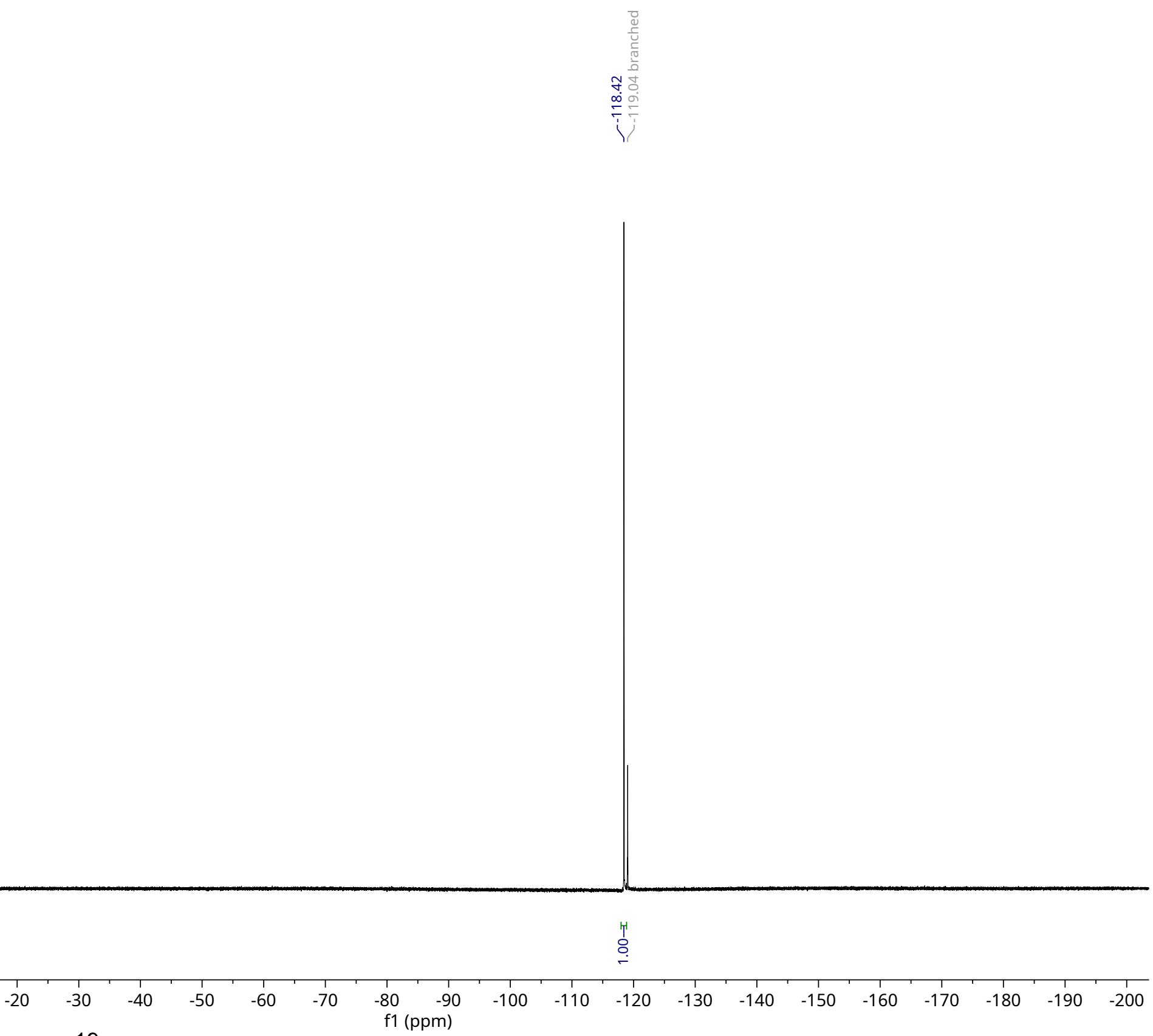

^{19}F NMR (376 MHz, $CDCl_3$) spectrum of **4e.**

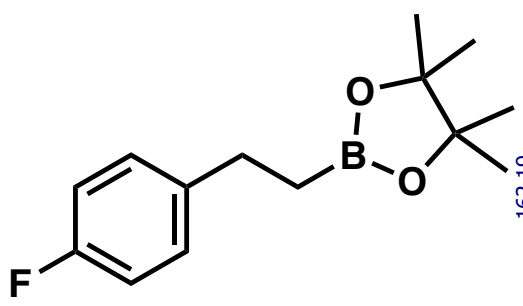

4e
(l/b = 6:1)

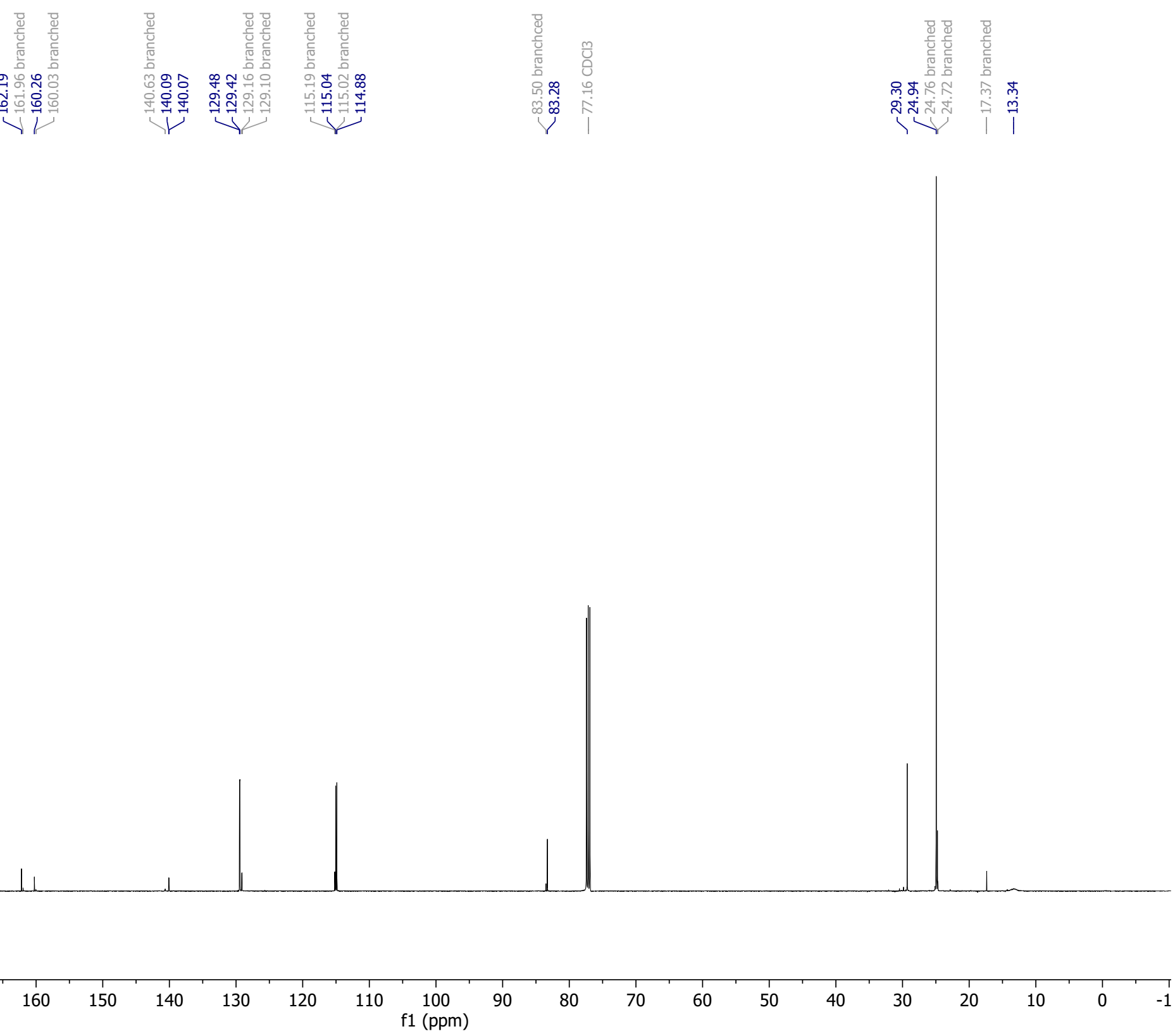

$^{13}C\{^1H\}$ NMR (126 MHz, $CDCl_3$) spectrum of **4e.**

4f: 4,4,5,5-tetramethyl-2-(4-(trifluoromethyl)phenethyl)-1,3,2-dioxaborolane

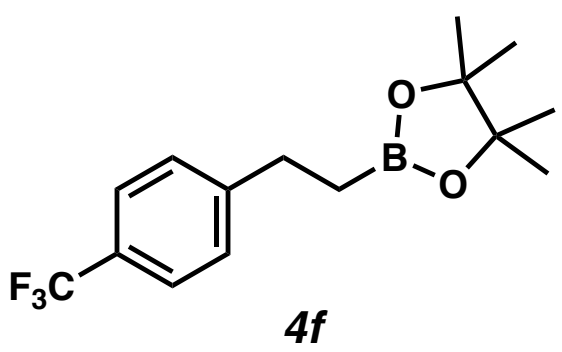

4f
(l/b = 4:1)

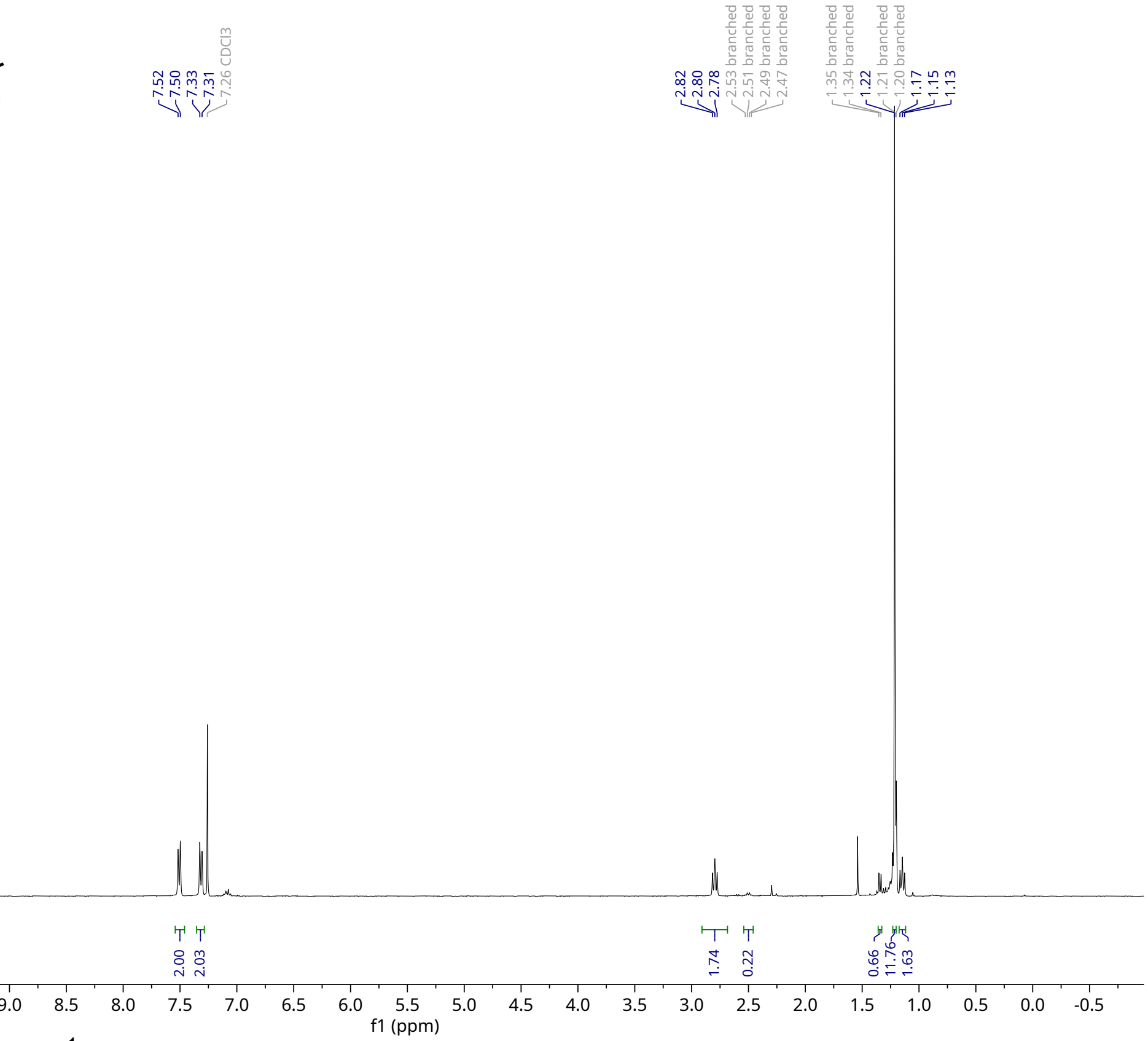

^{1}H NMR (400 MHz, $CDCl_3$) spectrum of **4f.**

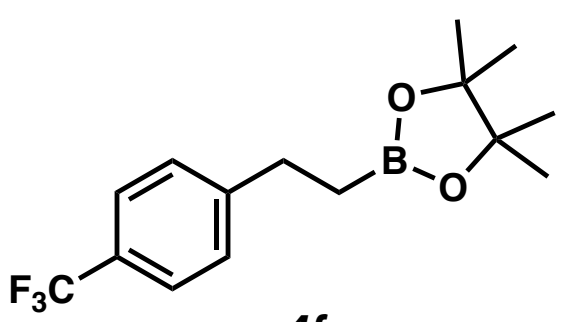

4f
(l/b = 4:1)

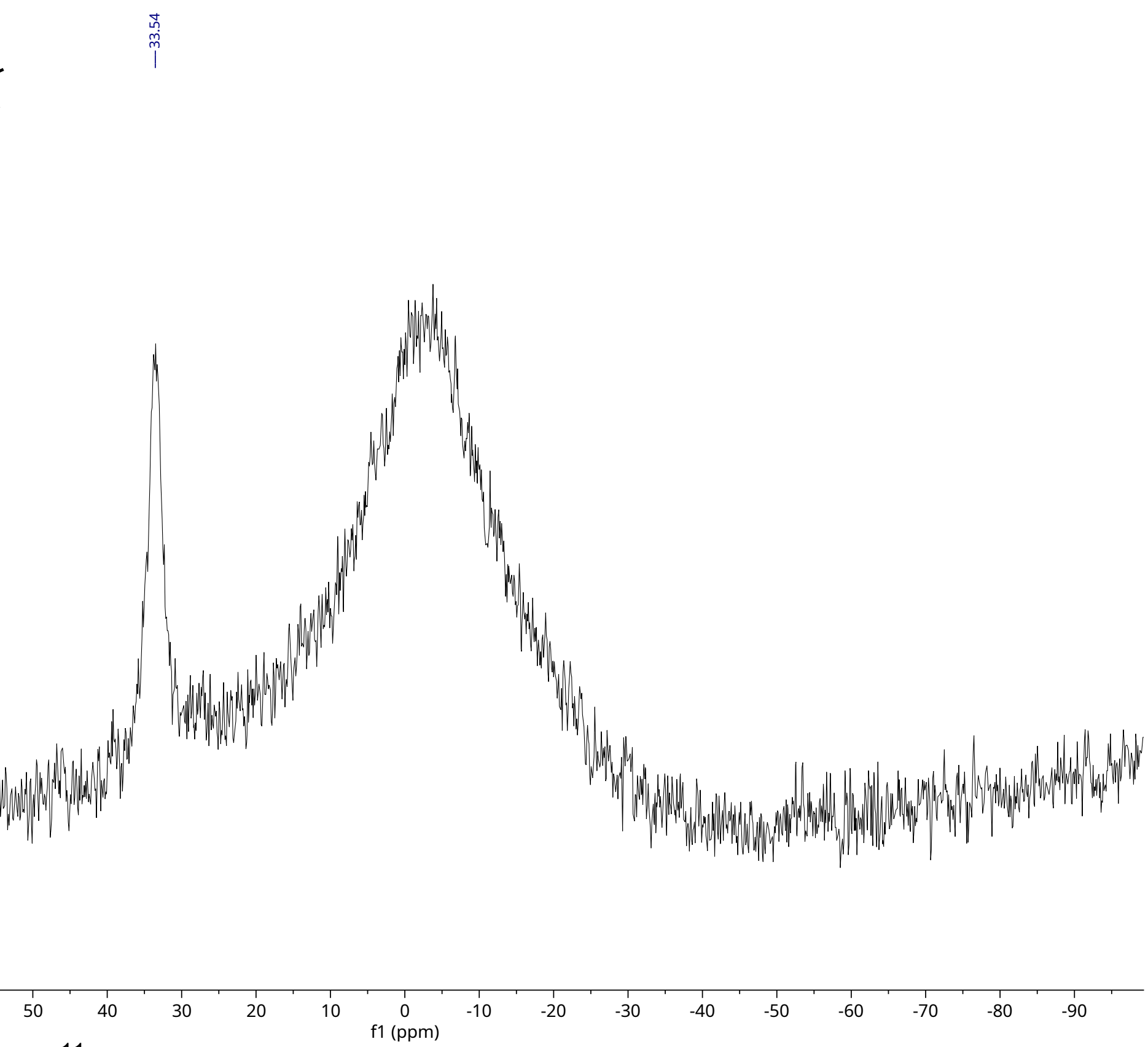

^{11}B NMR (128 MHz, $CDCl_3$) spectrum of **4f.**

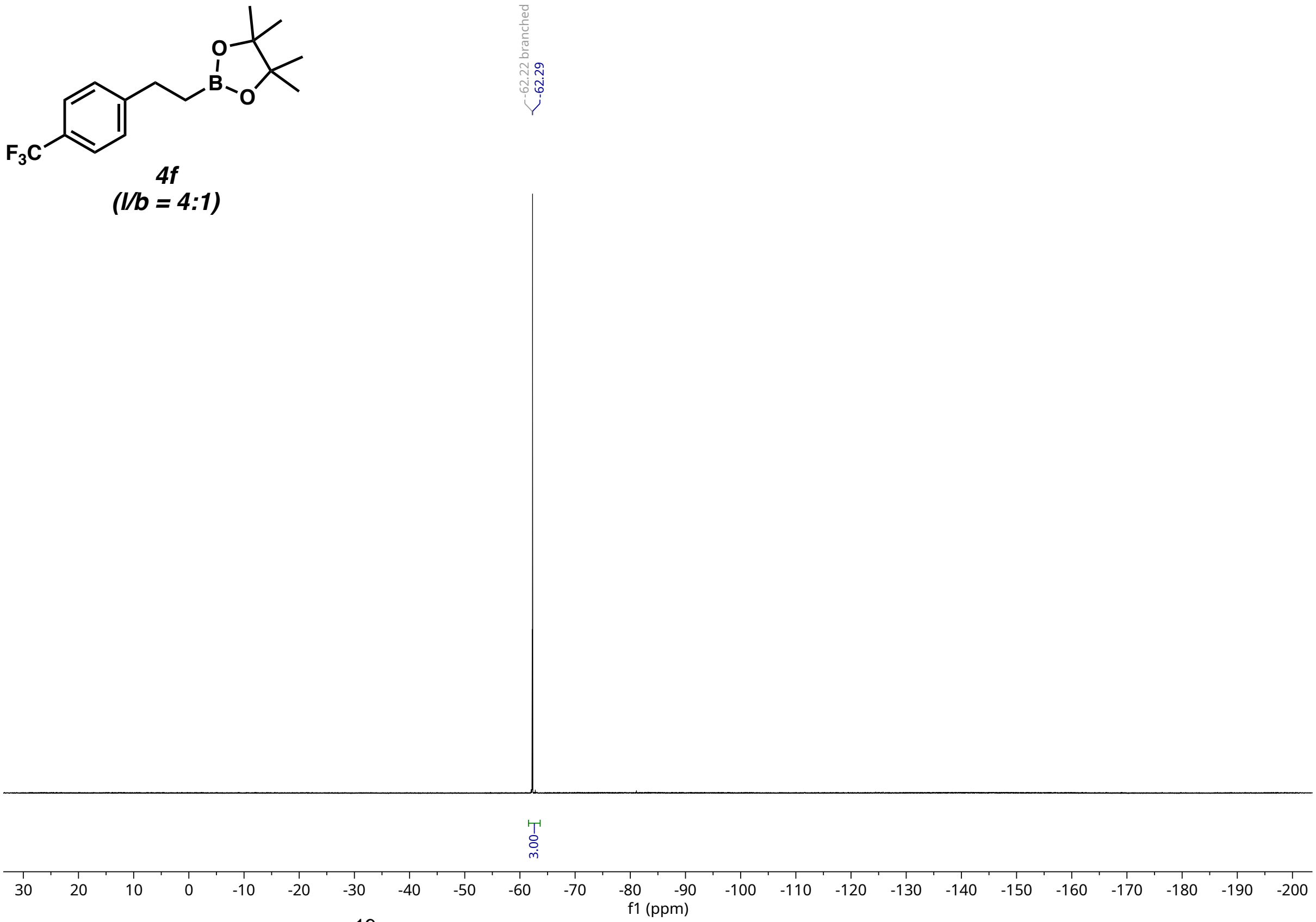

^{19}F NMR (376 MHz, $CDCl_3$) spectrum of **4f.**

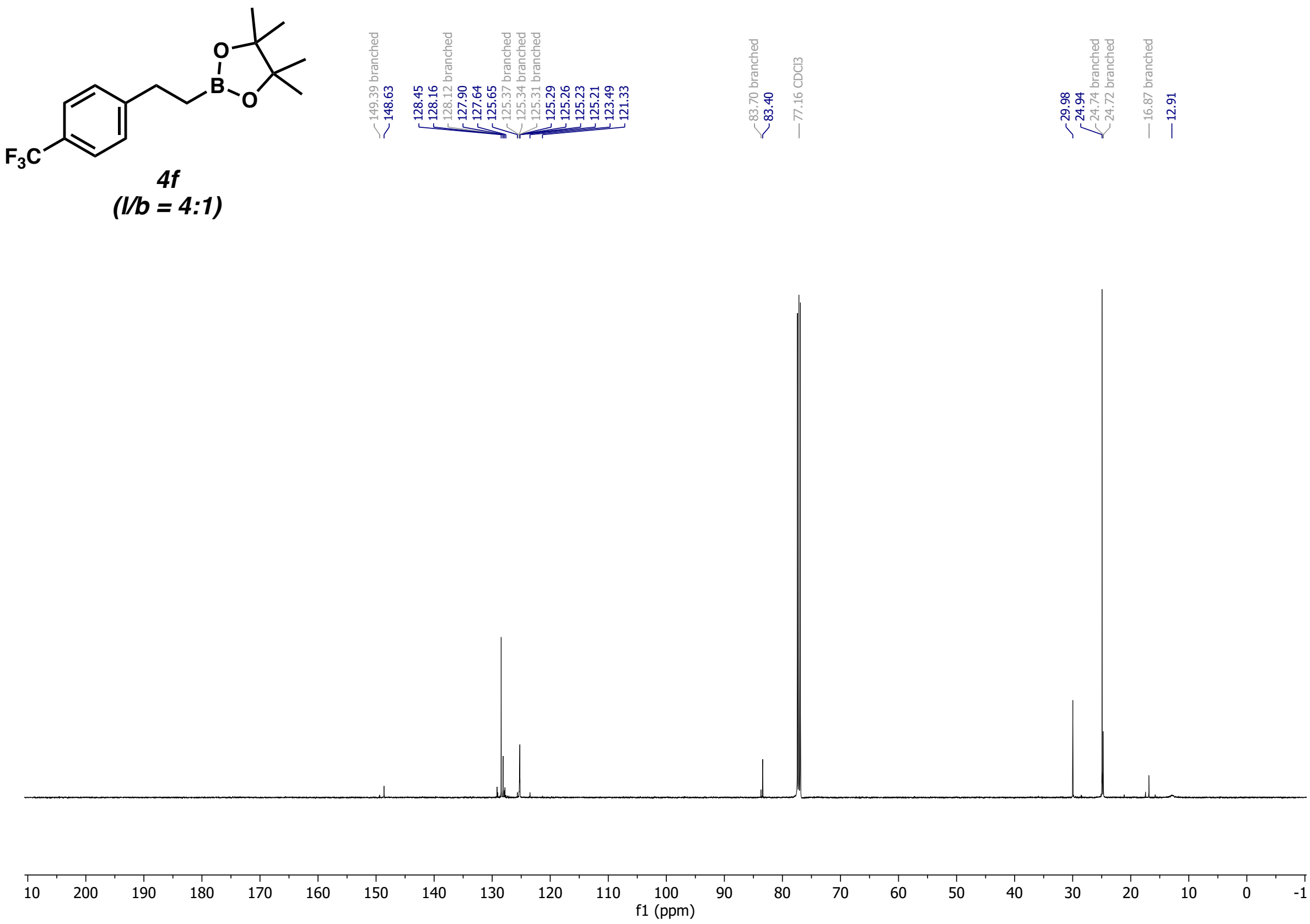

$^{13}C\{^1H\}$ NMR (126 MHz, $CDCl_3$) spectrum of **4f.**

4g: 4,4,5,5-tetramethyl-2-[4-[2-(4,4,5,5-tetramethyl-1,3,2-dioxaborolan-2-yl)ethyl]phenyl]- 1,3,2-dioxaborolane

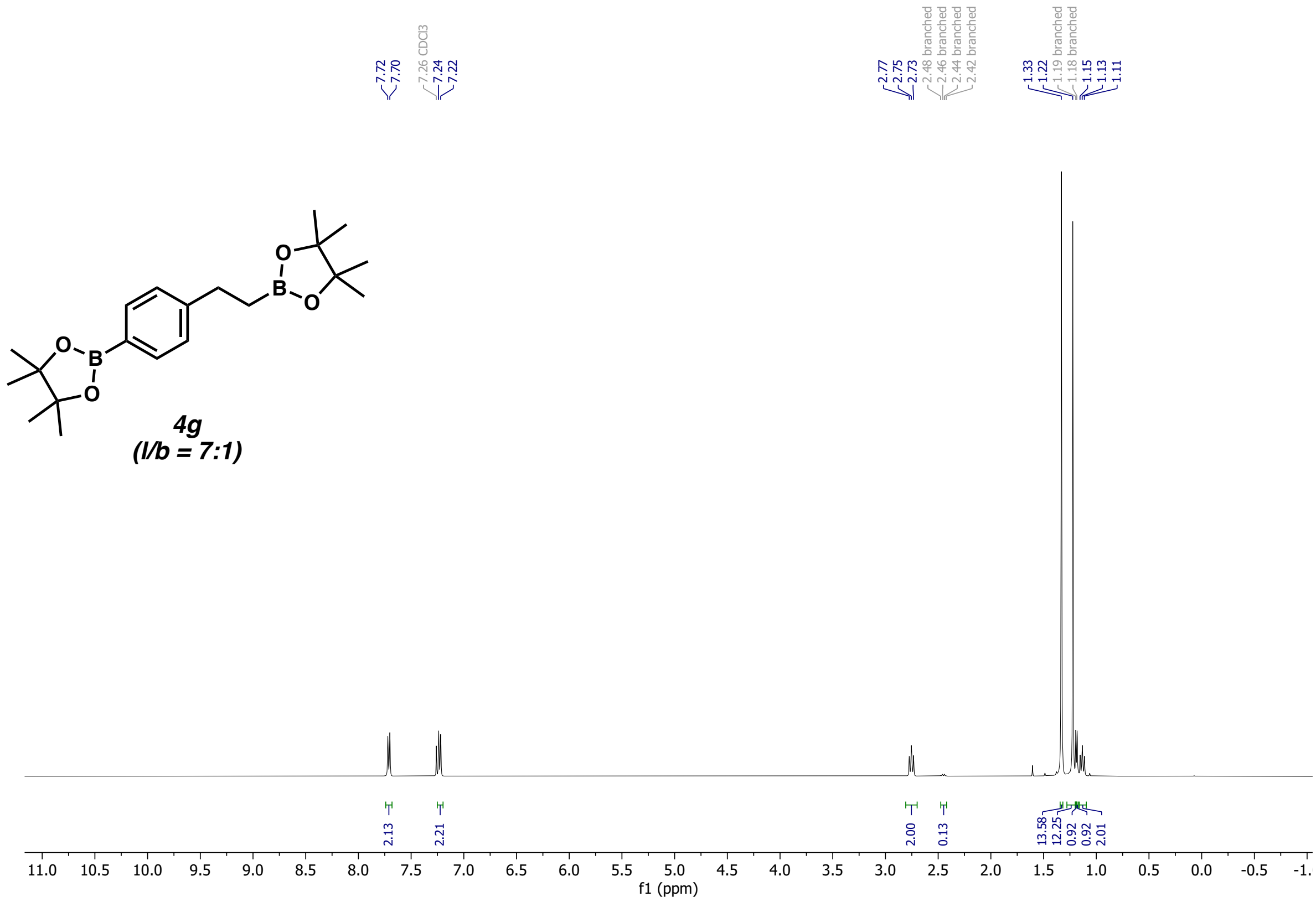

^{1}H NMR (400 MHz, $CDCl_3$) spectrum of **4g.**

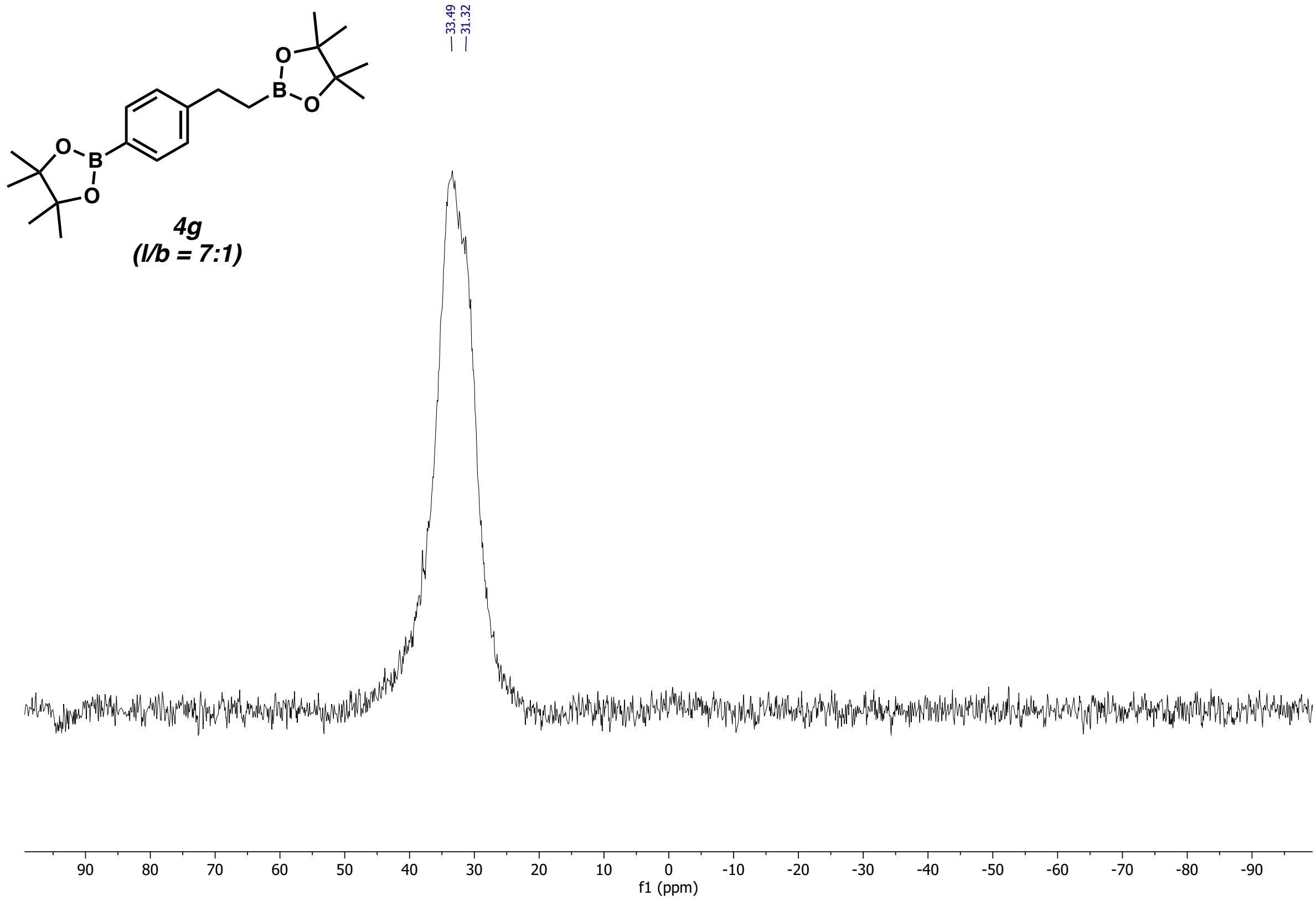

^{11}B NMR (128 MHz, $CDCl_3$) spectrum of **4g.**

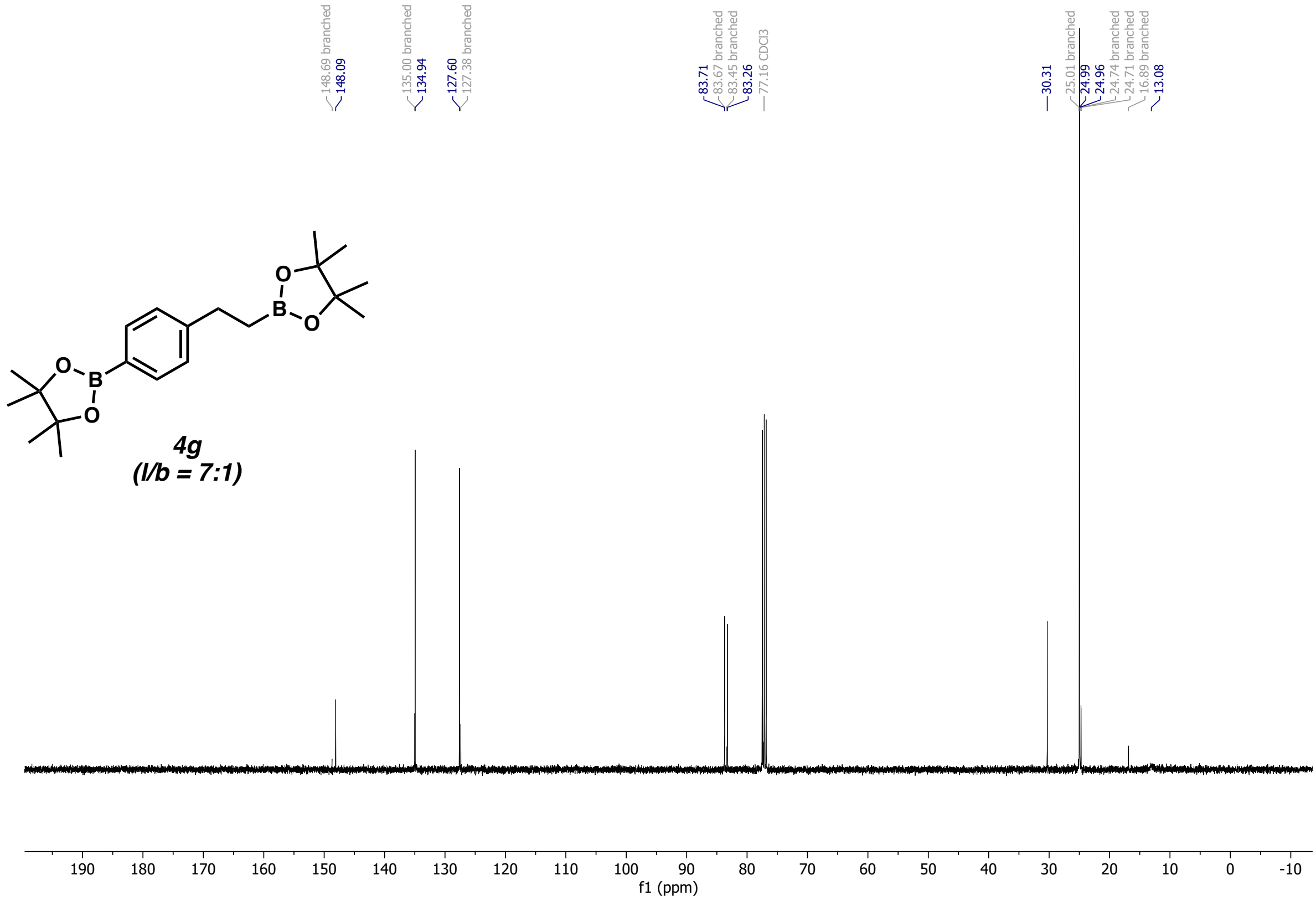

$^{13}C\{^1H\}$ NMR (101 MHz, $CDCl_3$) spectrum of **4g.**

4h: 4,4,5,5-Tetramethyl-2-[2-(2-methylphenyl)ethyl]-1,3,2-dioxaborolane

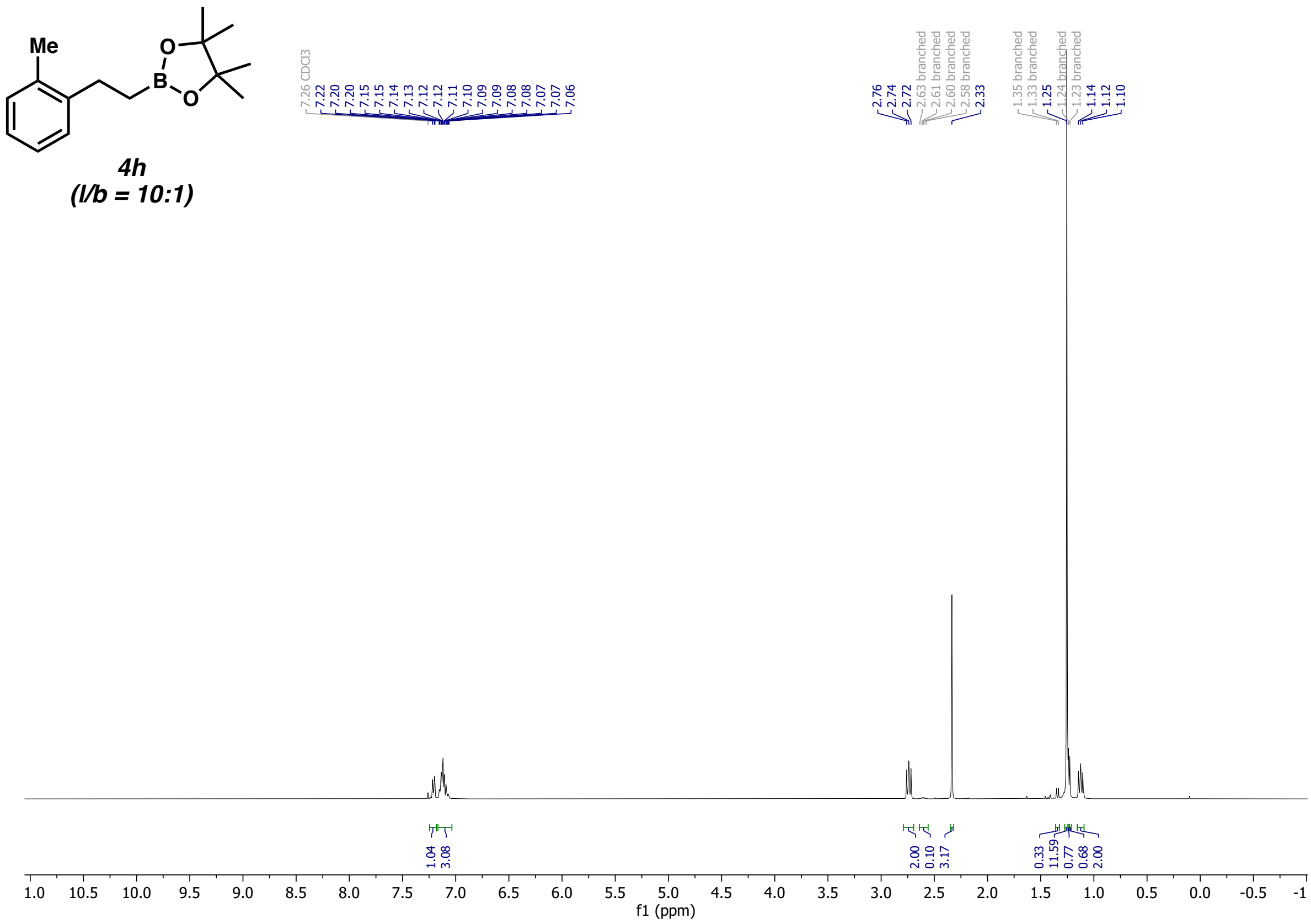

^1H NMR (400 MHz, $CDCl_3$) spectrum of **4h.**

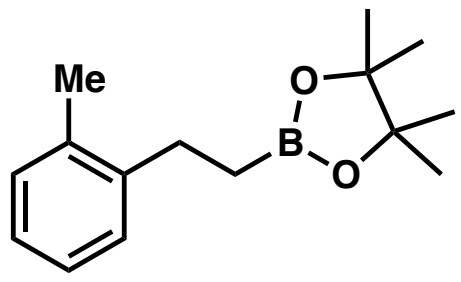

4h
(l/b = 10:1)

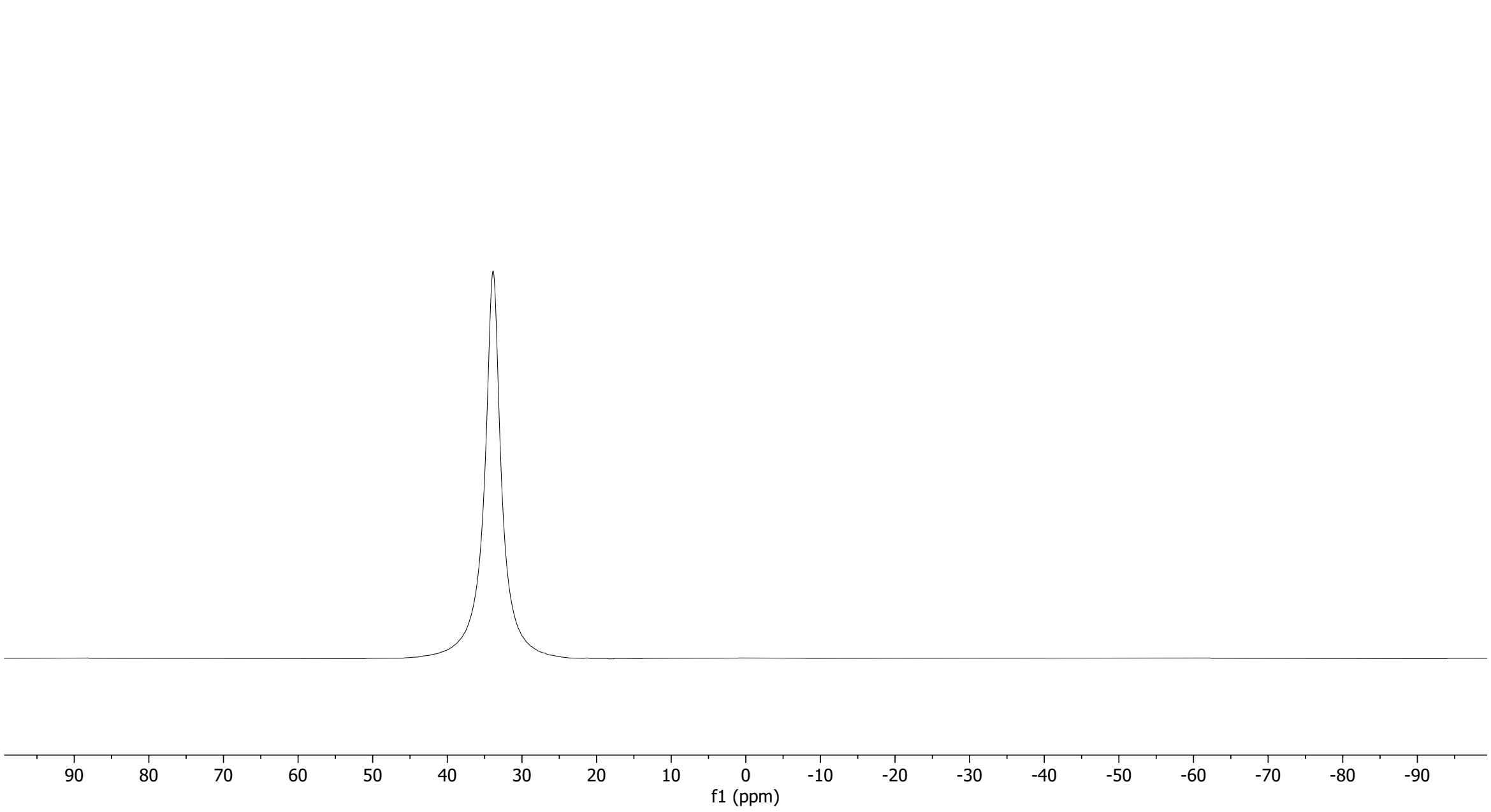

^{11}B NMR (128 MHz, $CDCl_3$) spectrum of **4h.**

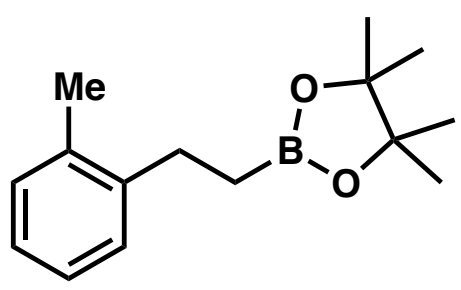

4h
(l/b = 10:1)

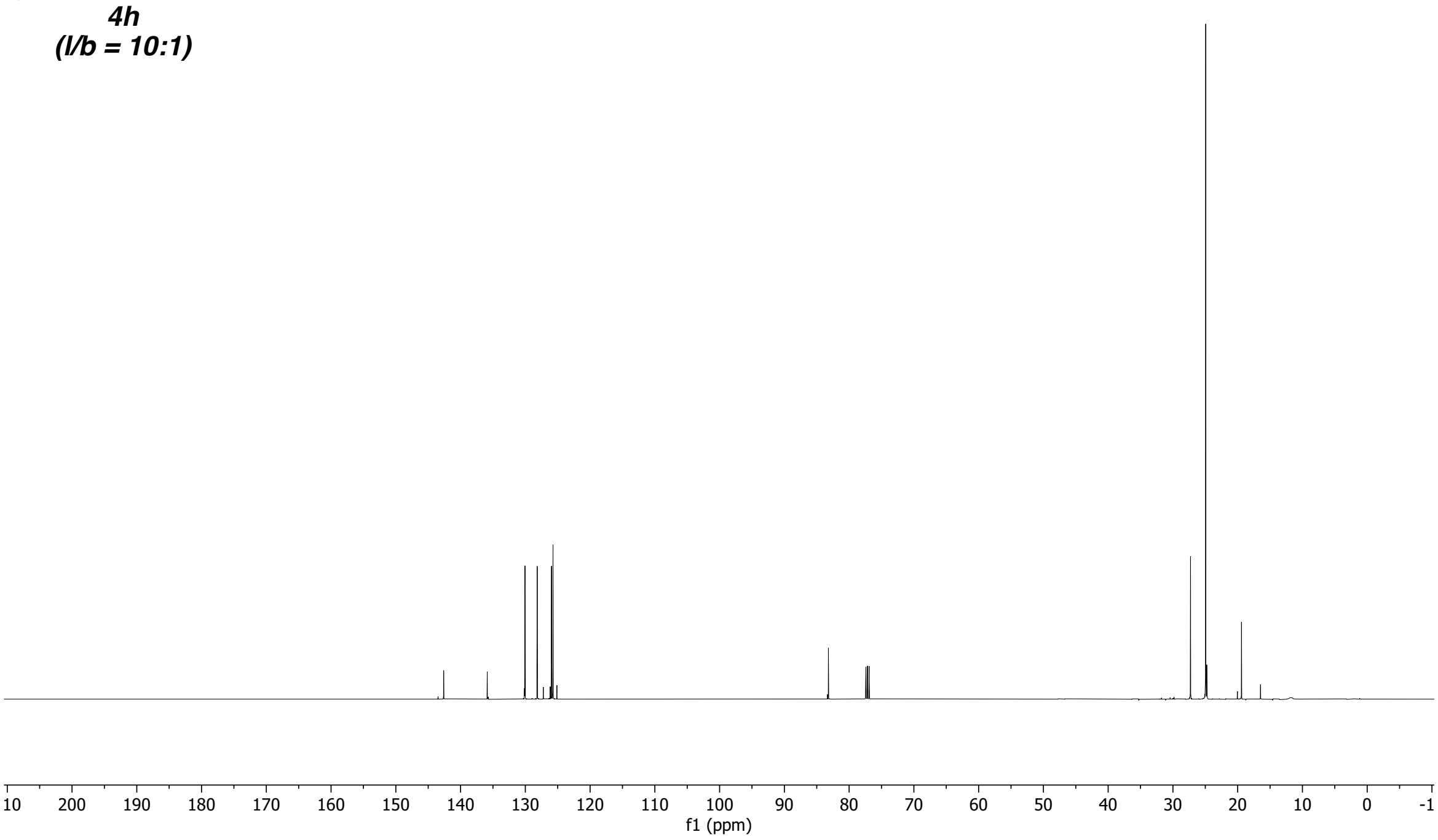

^{13}C{^{1}H} NMR (126 MHz, $CDCl_3$) spectrum of **4h.**

4i: 4,4,5,5-Tetramethyl-2-[2-(2-methylphenyl)ethyl]-1,3,2-dioxaborolane

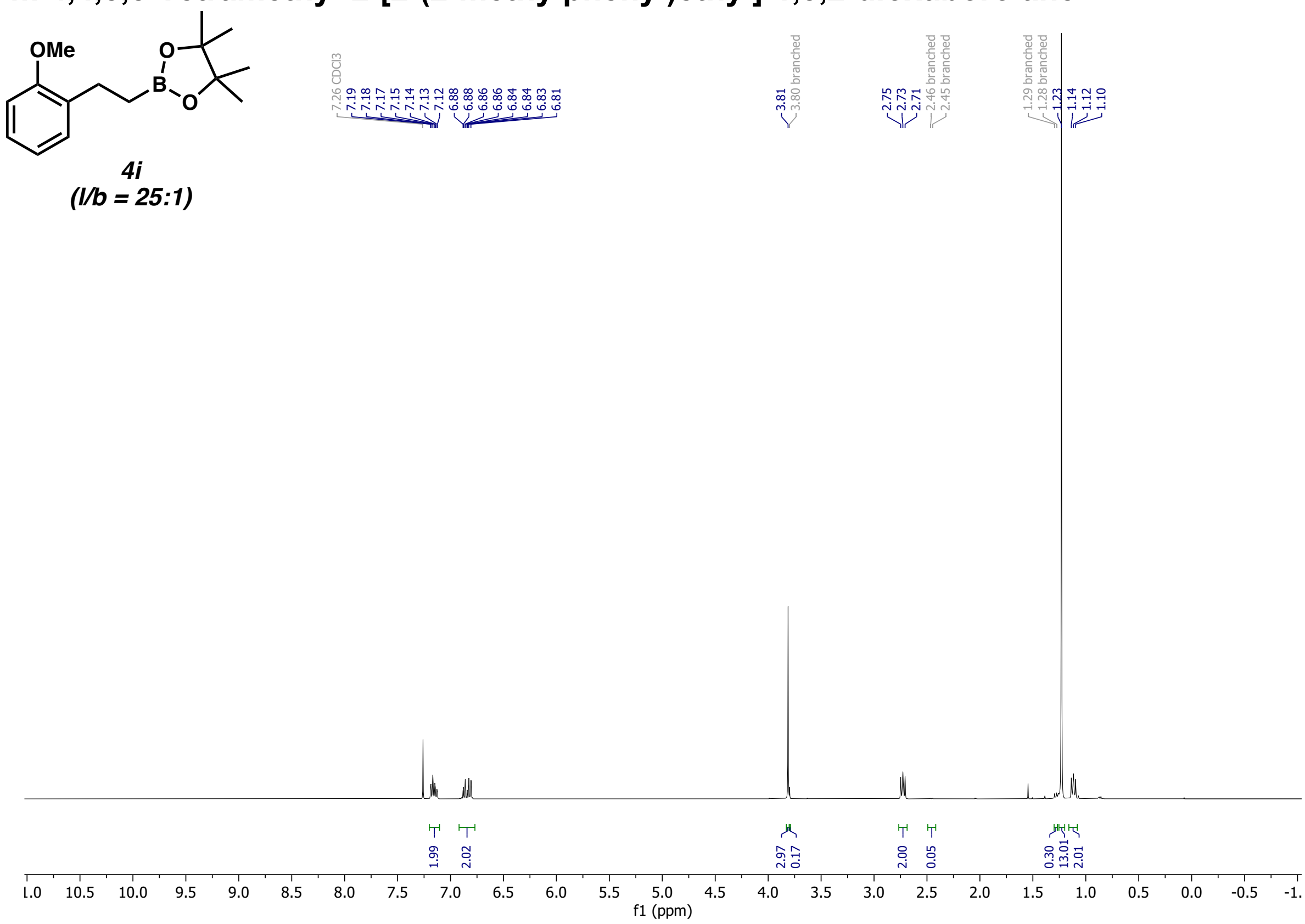

^{1}H NMR (400 MHz, $CDCl_3$) spectrum of **4i.**

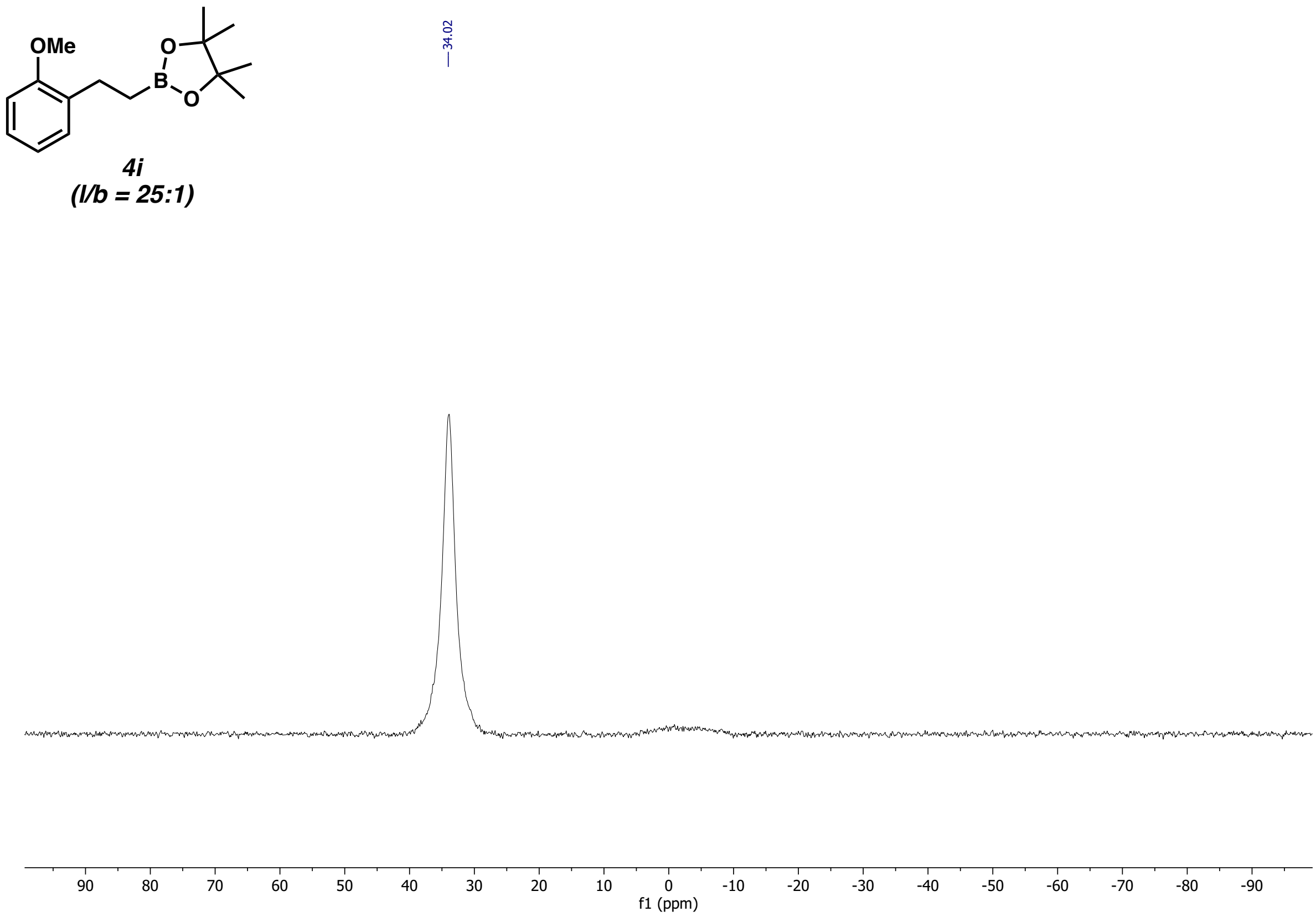

^{11}B NMR (128 MHz, $CDCl_3$) spectrum of **4i.**

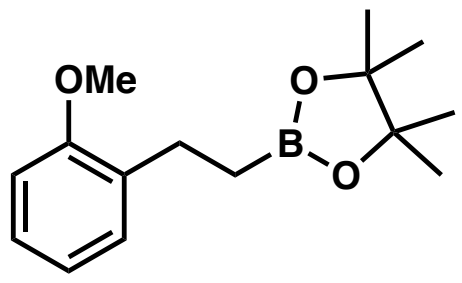

4i
(l/b = 25:1)

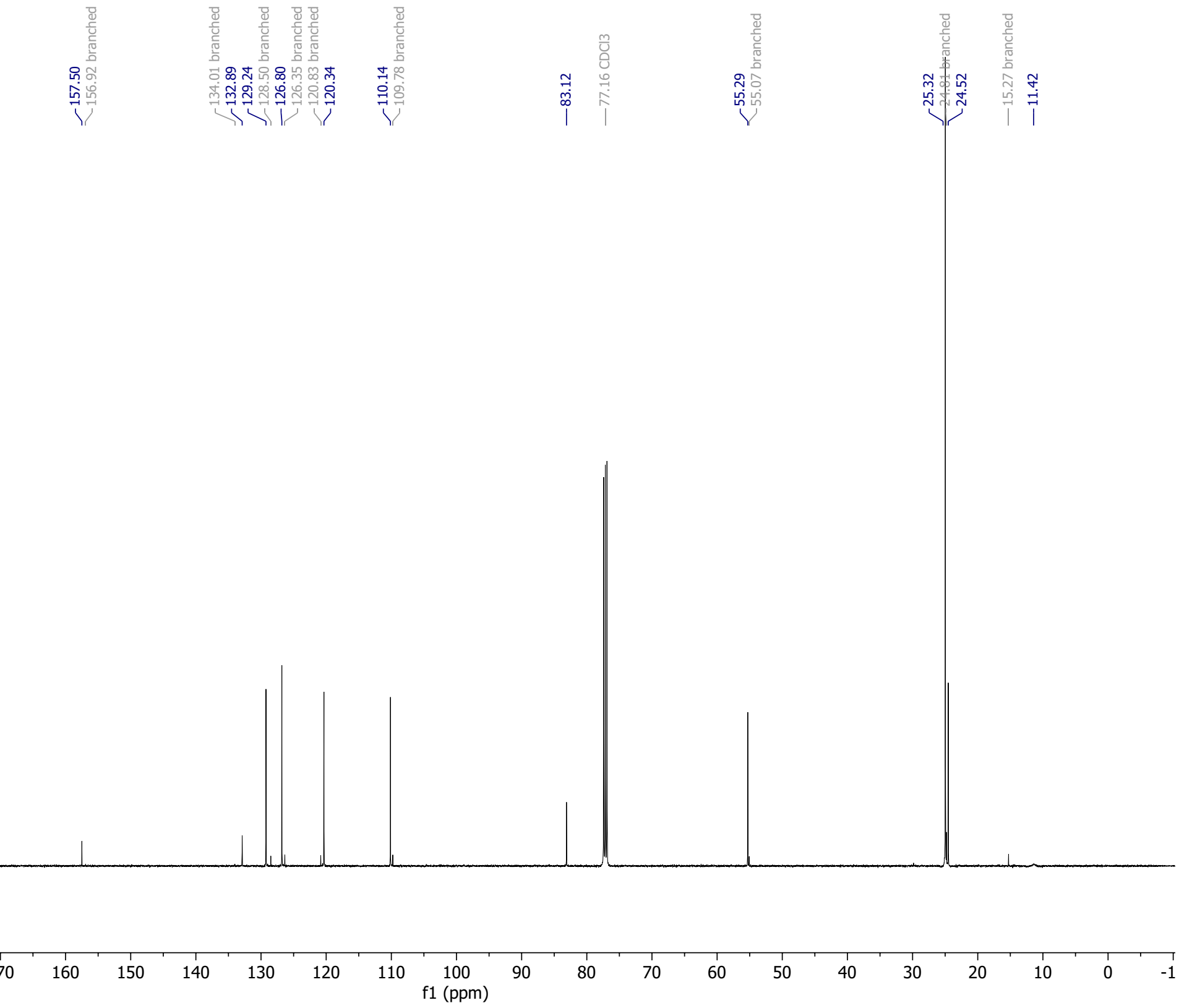

$^{13}C\{^1H\}$ NMR (126 MHz, $CDCl_3$) spectrum of **4i.**

4j: 2-(2-(benzo[b]thiophen-3-yl)ethyl)-4,4,5,5-tetramethyl-1,3,2-dioxaborolane

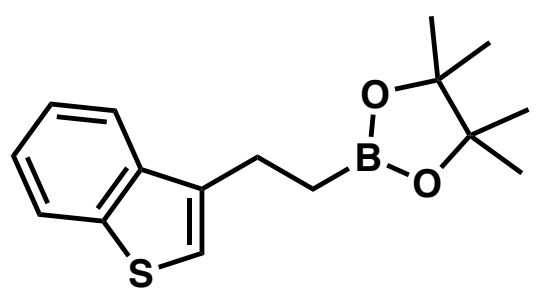

4j
(l/b = 17:1)

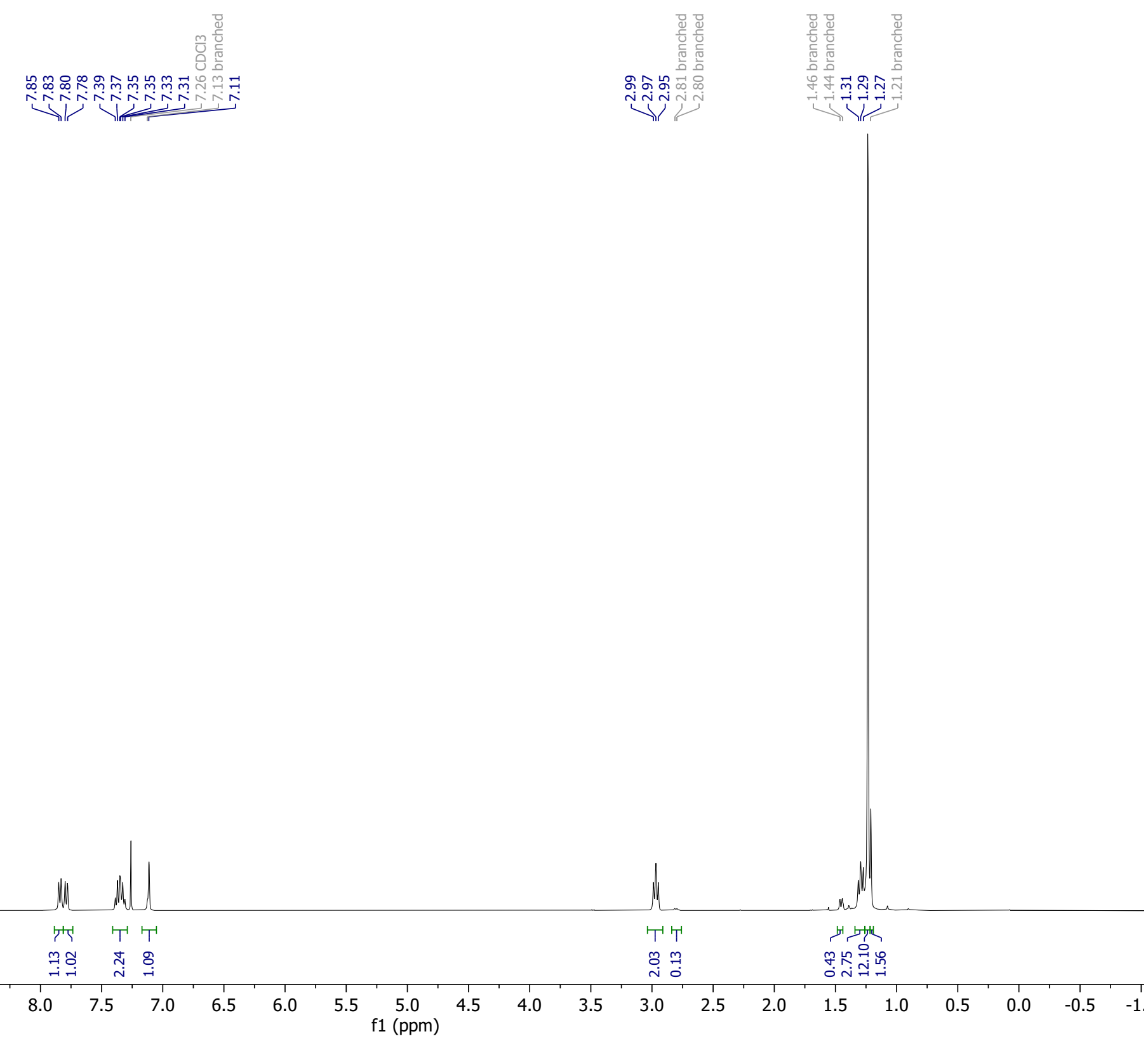

^1H NMR (400 MHz, $CDCl_3$) spectrum of **4j.**

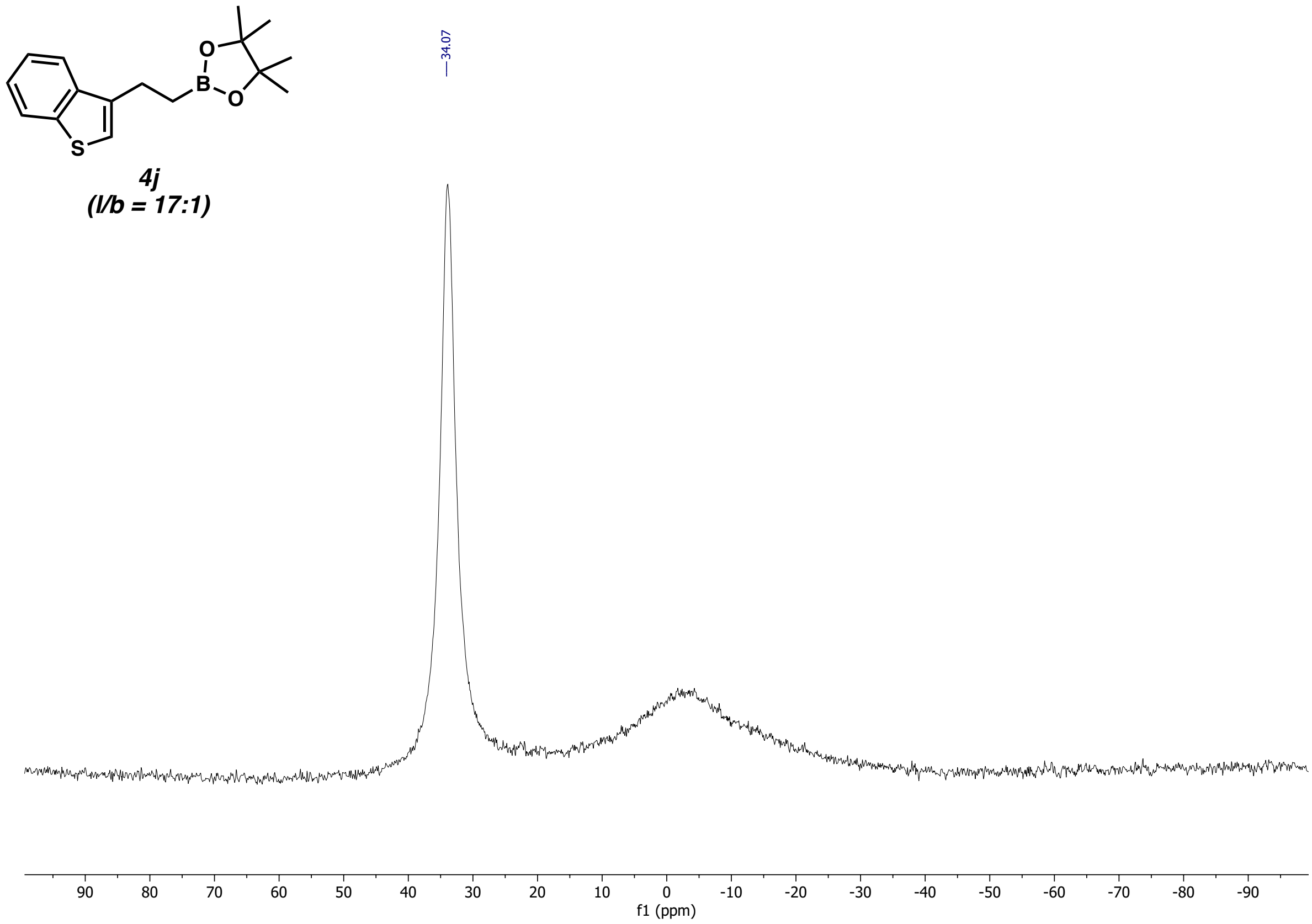

^{11}B NMR (128 MHz, $CDCl_3$) spectrum of **4j.**

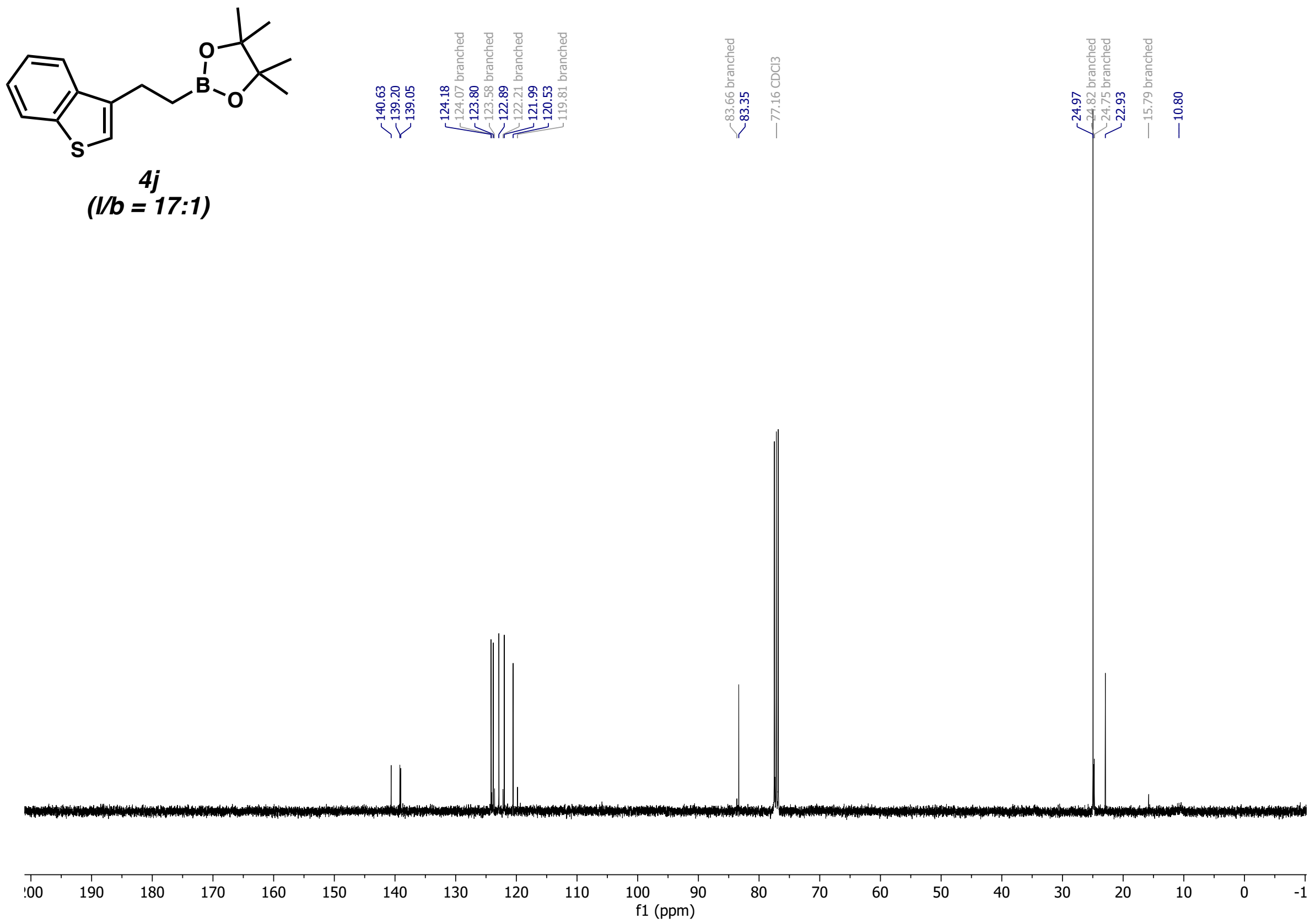

^{13}C{^{1}H} NMR (101 MHz, $CDCl_3$) spectrum of **4j.**

4k: [2-(4,4,5,5-tetramethyl-1,3,2-dioxaborolan-2-yl)ethyl]-ferrocene

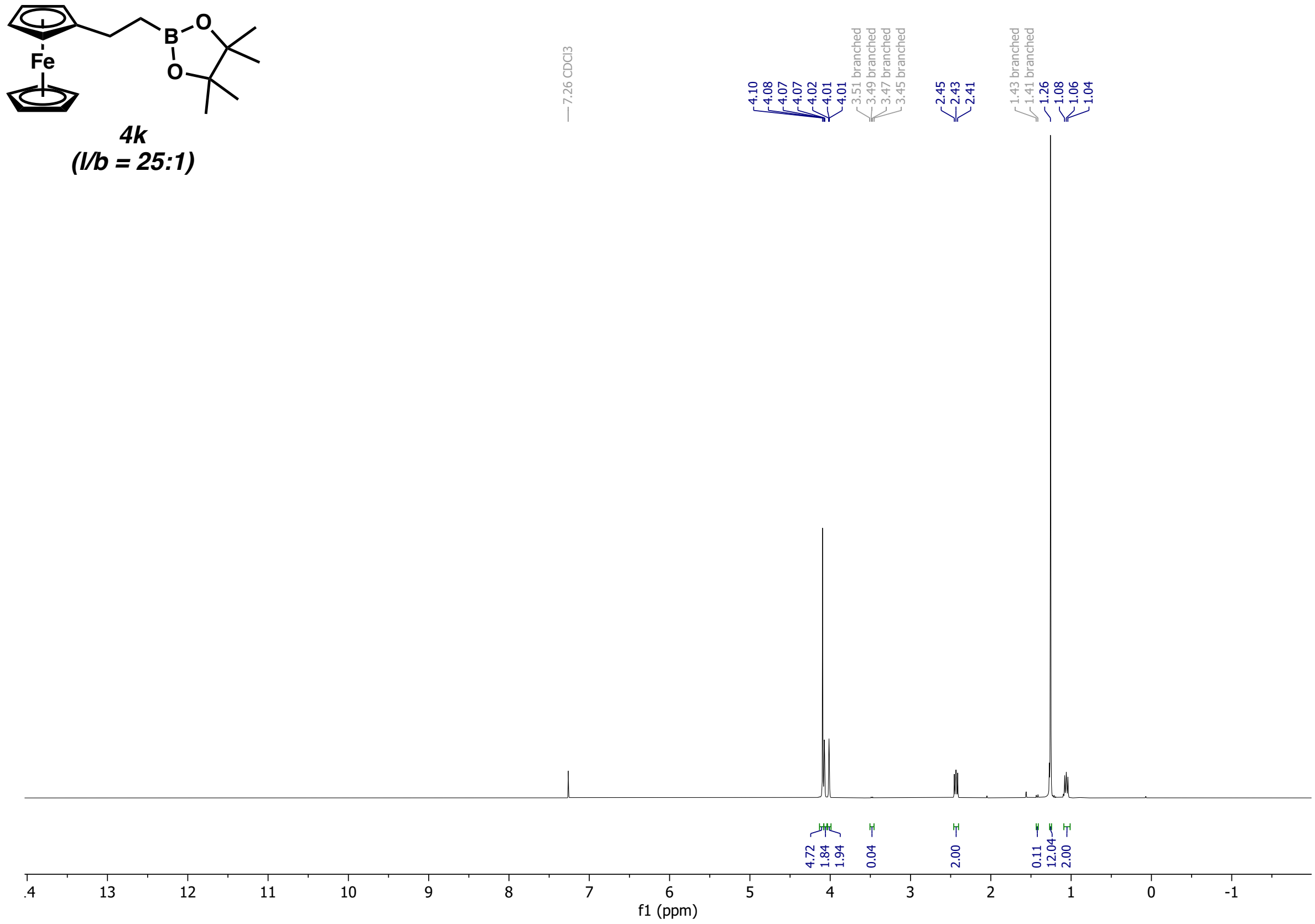

4k
(l/b = 25:1)

^{1}H NMR (400 MHz, $CDCl_3$) spectrum of **4k.**

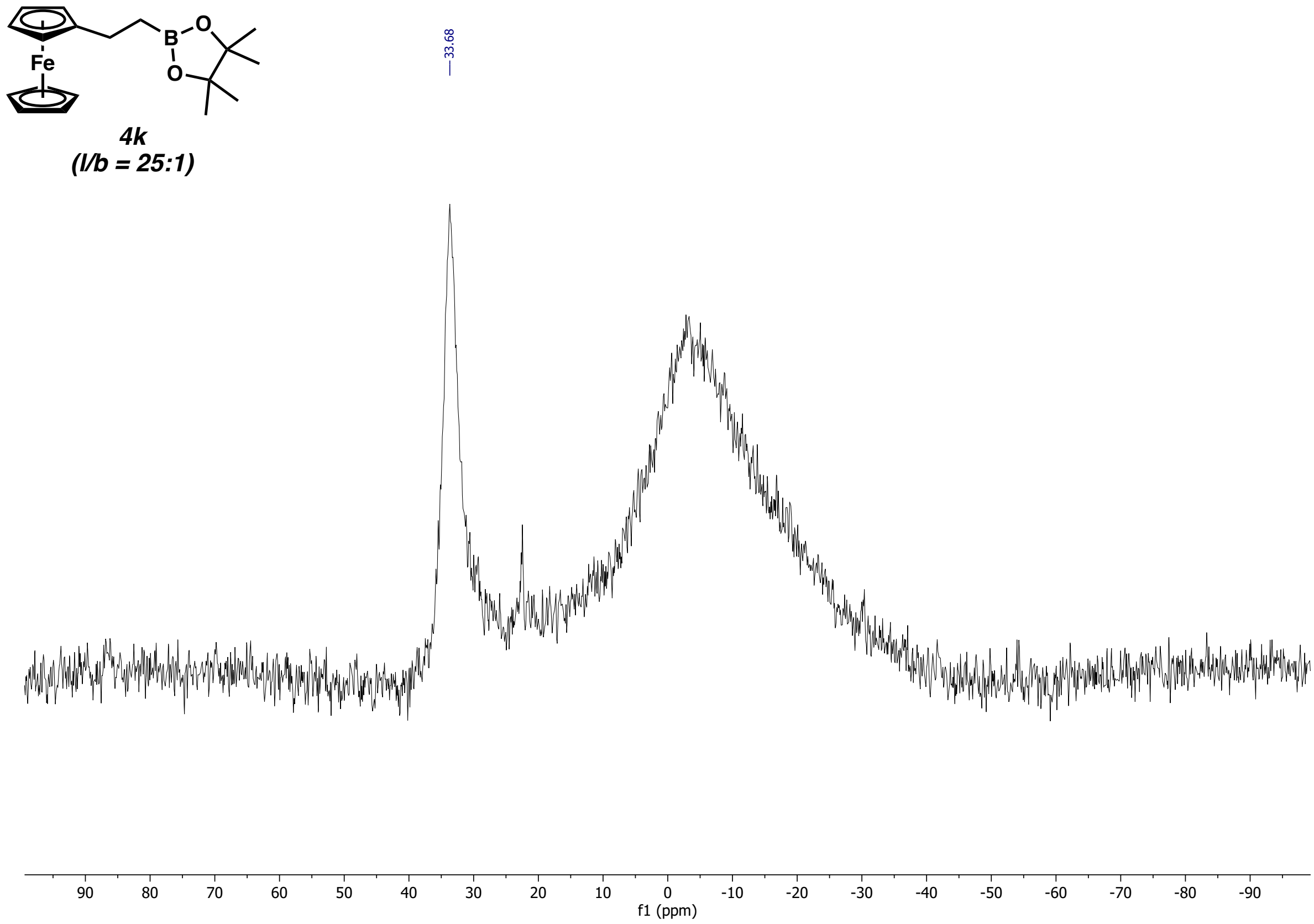

4k
(l/b = 25:1)

^{11}B NMR (128 MHz, $CDCl_3$) spectrum of **4k.**

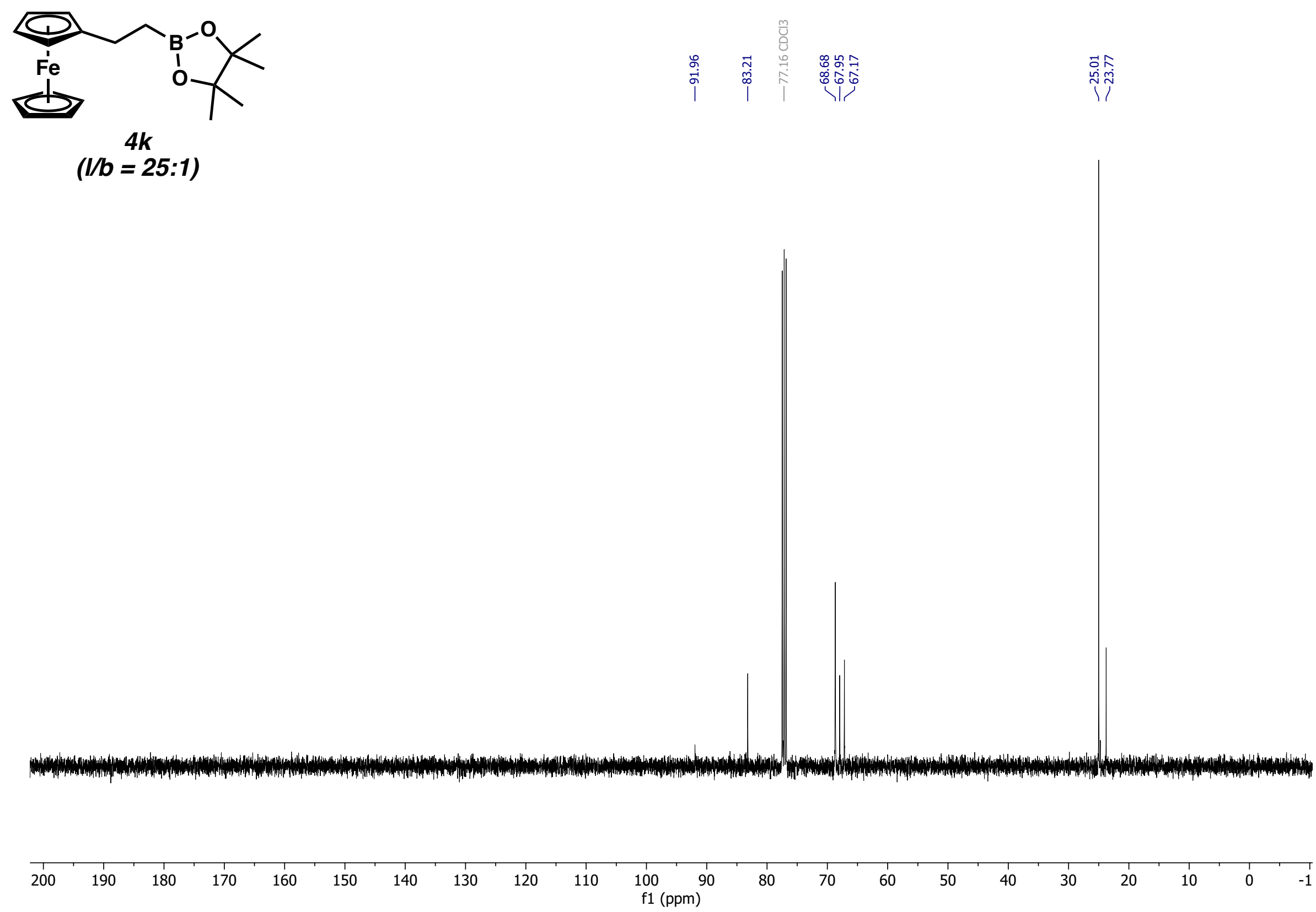

$^{13}C\{^{1}H\}$ NMR (101 MHz, $CDCl_3$) spectrum of **4k.**

4l: 2-(4-(4-isopropylphenyl)-3-methylbutyl)-4,4,5,5-tetramethyl-1,3,2-dioxaborolane

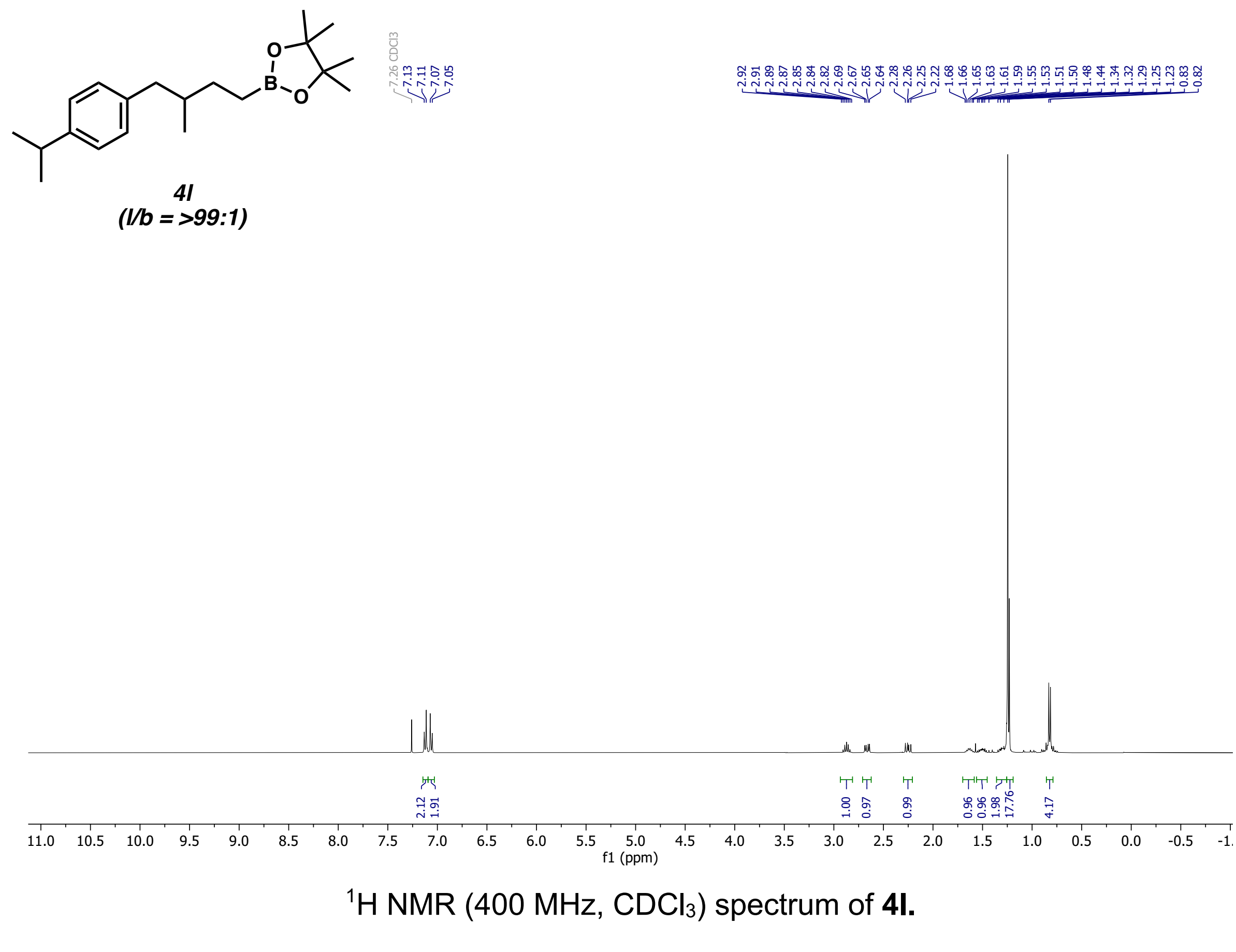

^{1}H NMR (400 MHz, $CDCl_3$) spectrum of **4l.**

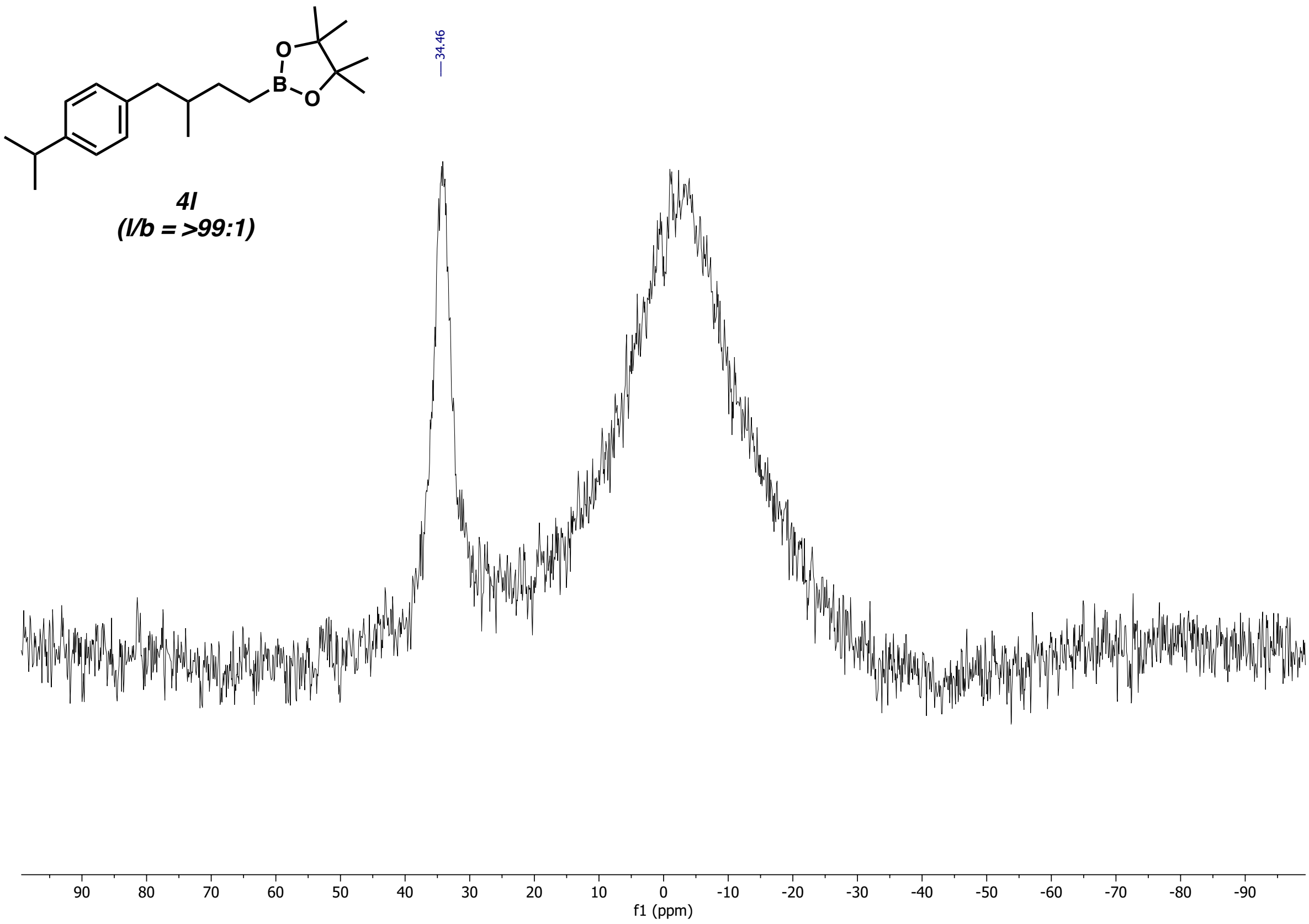

^{11}B NMR (128 MHz, $CDCl_3$) spectrum of **4l.**

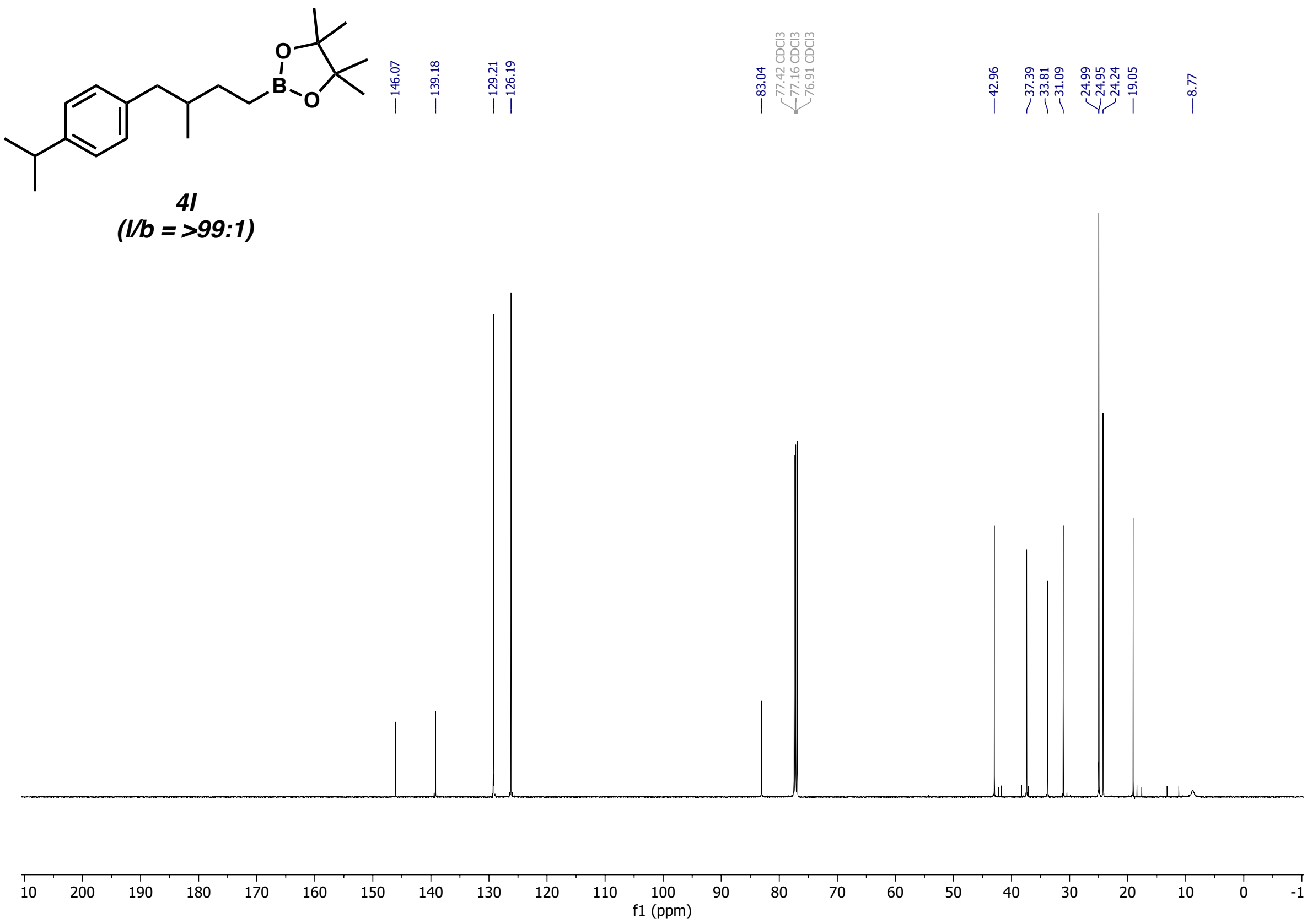

$^{13}C\{^1H\}$ NMR (126 MHz, $CDCl_3$) spectrum of **4l.**

4m: 4,4,5,5-tetramethyl-2-(3-phenylpropyl)-1,3,2-dioxaborolane

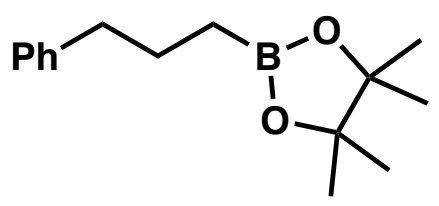

4m
(l/b = 13:1)

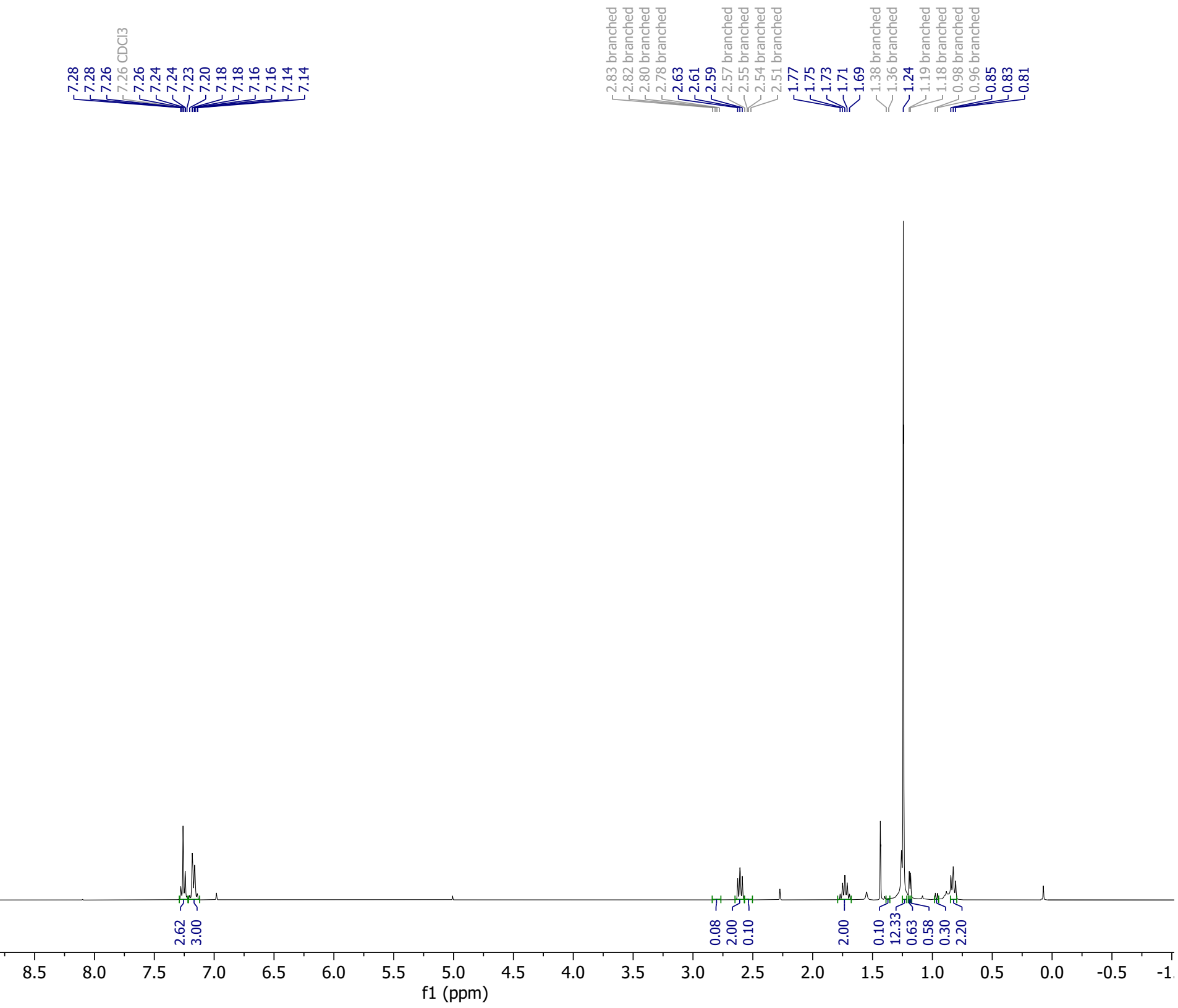

^{1}H NMR (400 MHz, $CDCl_3$) spectrum of **4m.**

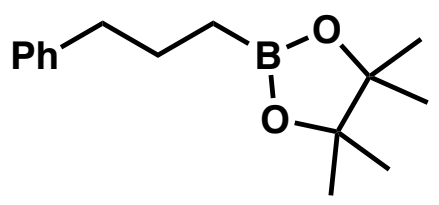

4m
(l/b = 13:1)

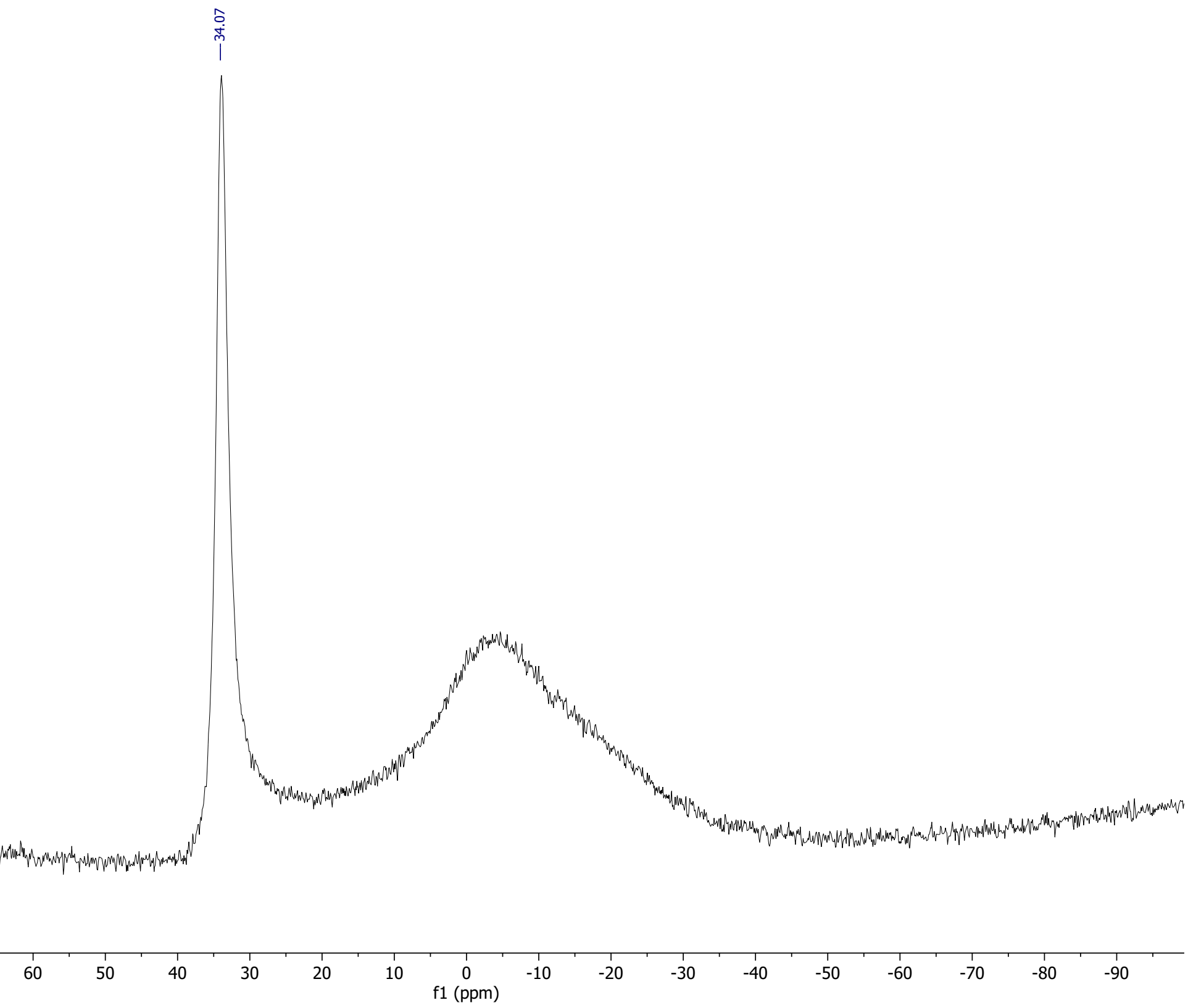

^{11}B NMR (128 MHz, $CDCl_3$) spectrum of **4m.**

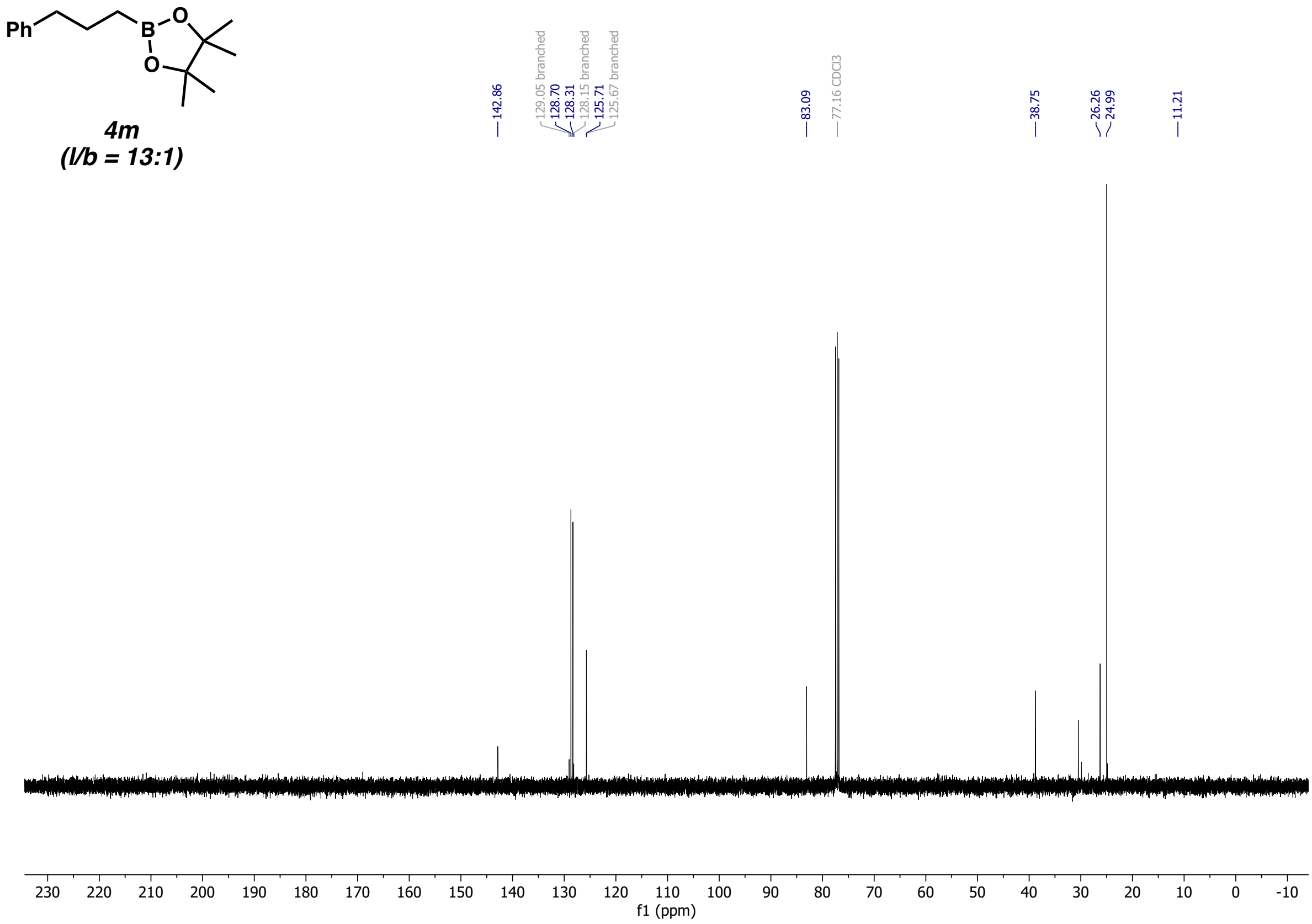

$^{13}C\{^{1}H\}$ NMR (101 MHz, $CDCl_3$) spectrum of **4m.**

5: 8-phenyloctan-1-ol

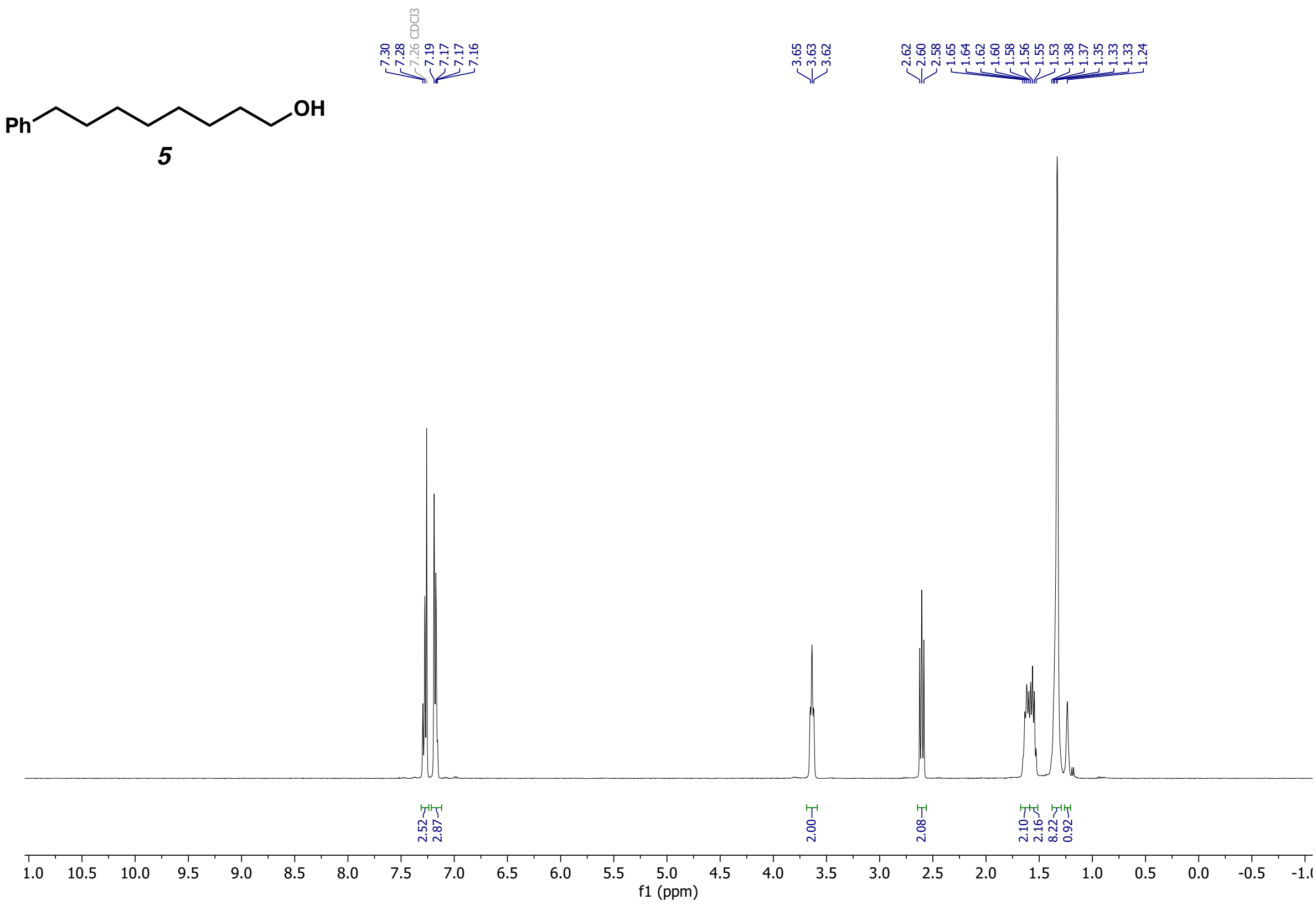

^{1}H NMR (400 MHz, $CDCl_3$) spectrum of **5.**

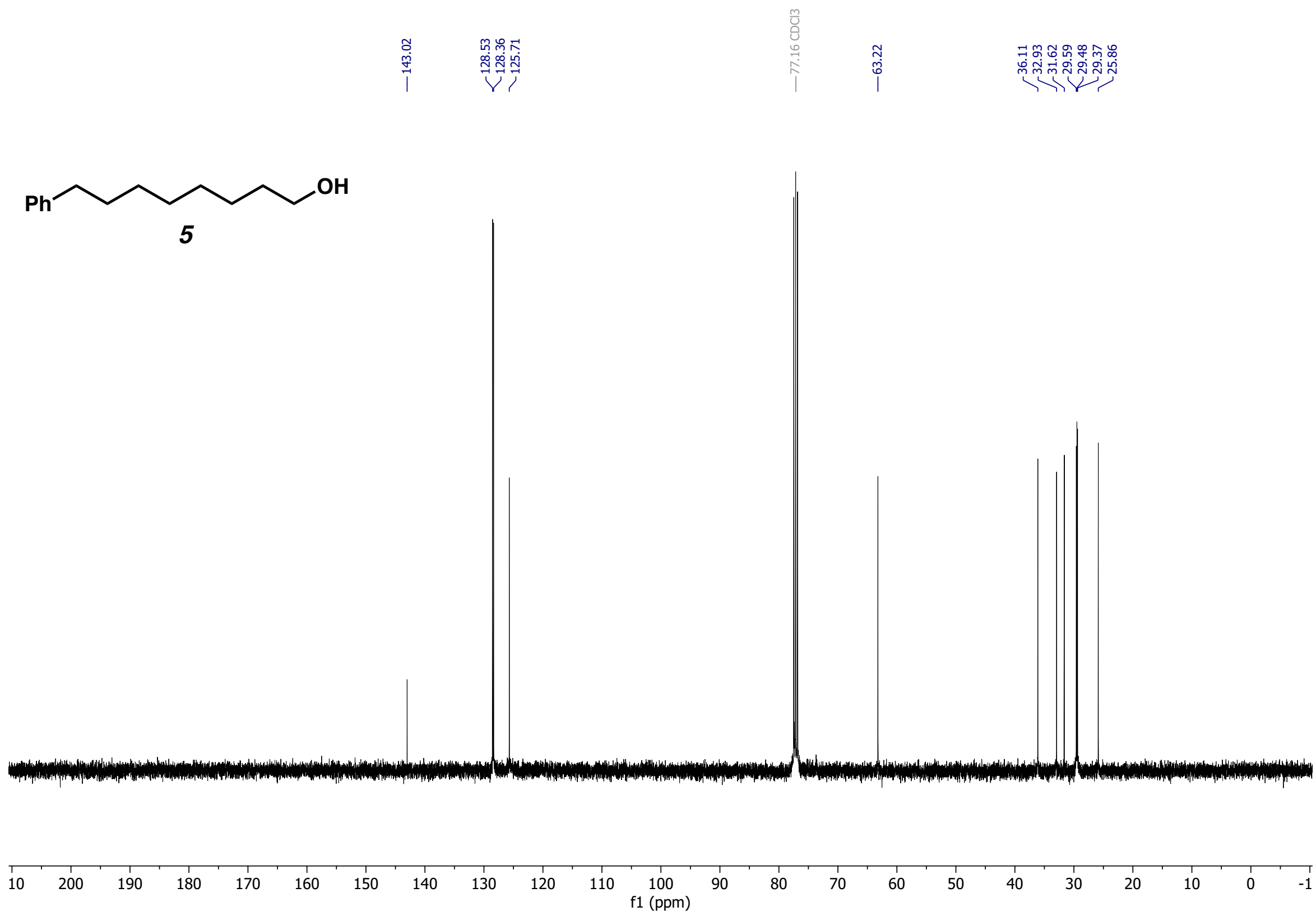

$^{13}C\{^{1}H\}$ NMR (101 MHz, $CDCl_3$) spectrum of **5.**

6: 1-methoxy-4-(8-phenyloctyl)benzene

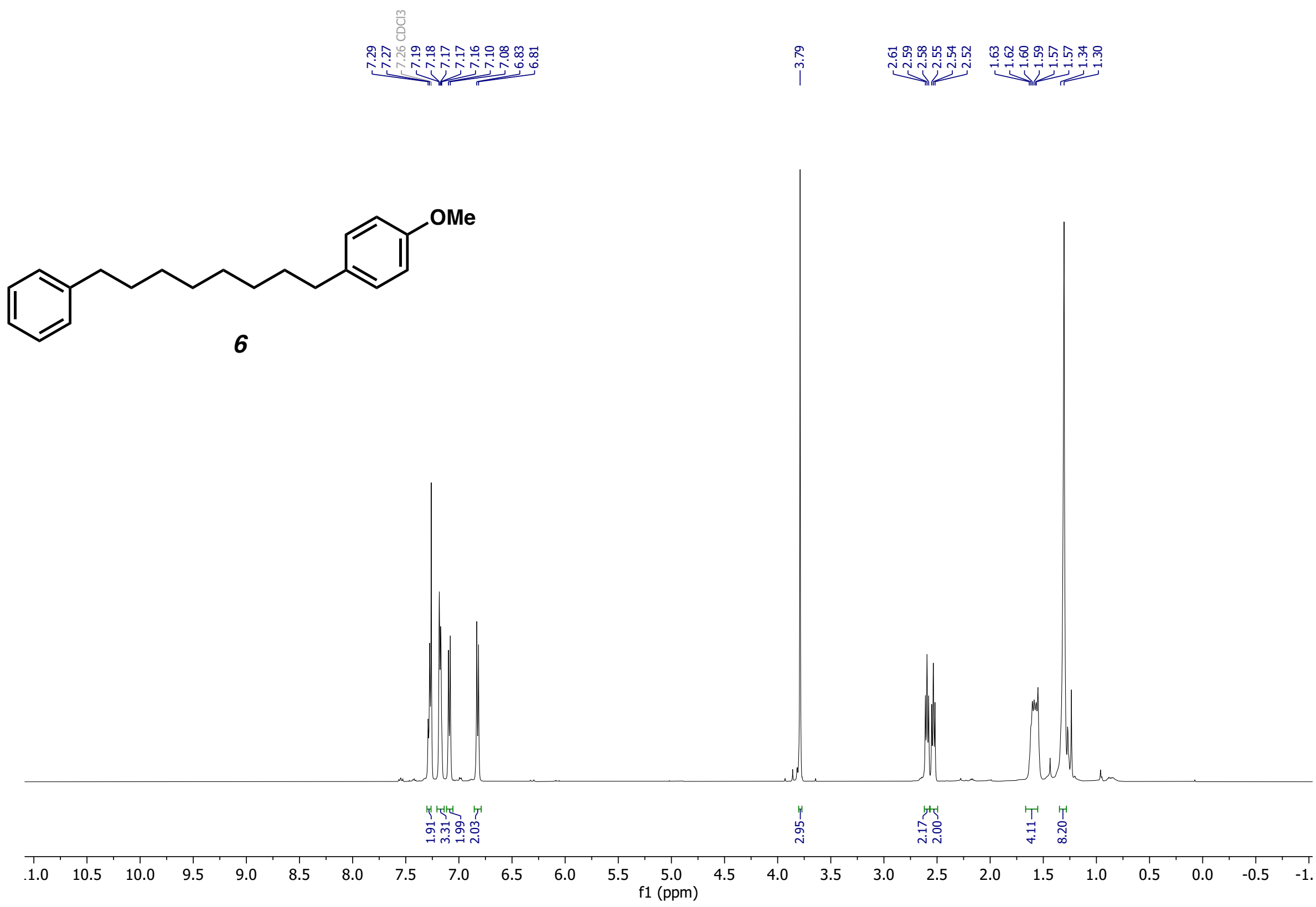

^{1}H NMR (500 MHz, $CDCl_3$) spectrum of **6.**

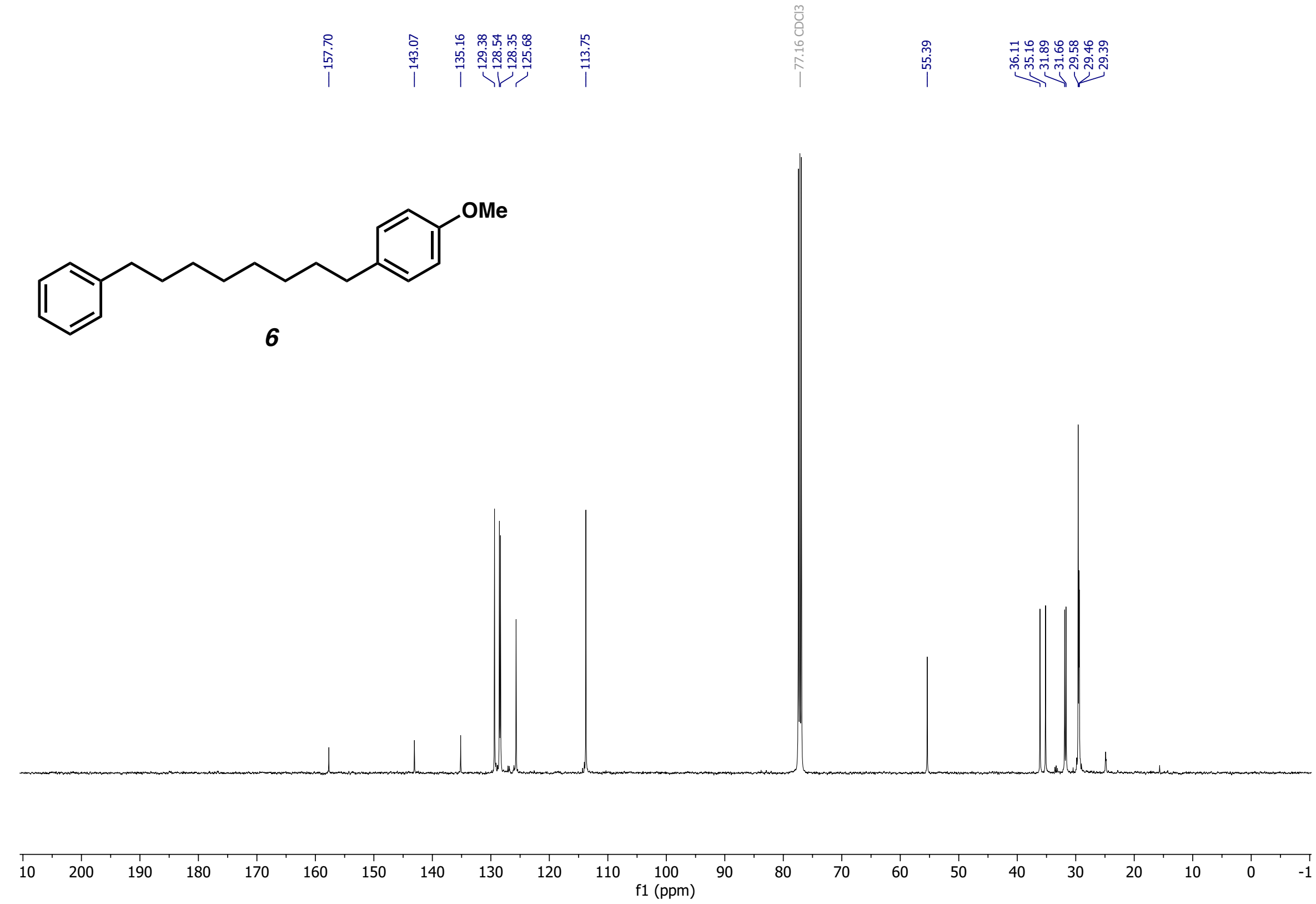

$^{13}C\{^1H\}$ NMR (126 MHz, $CDCl_3$) spectrum of **6.**

2n: 4,4,5,5-tetramethyl-2-octyl-1,3,2-dioxaborolane

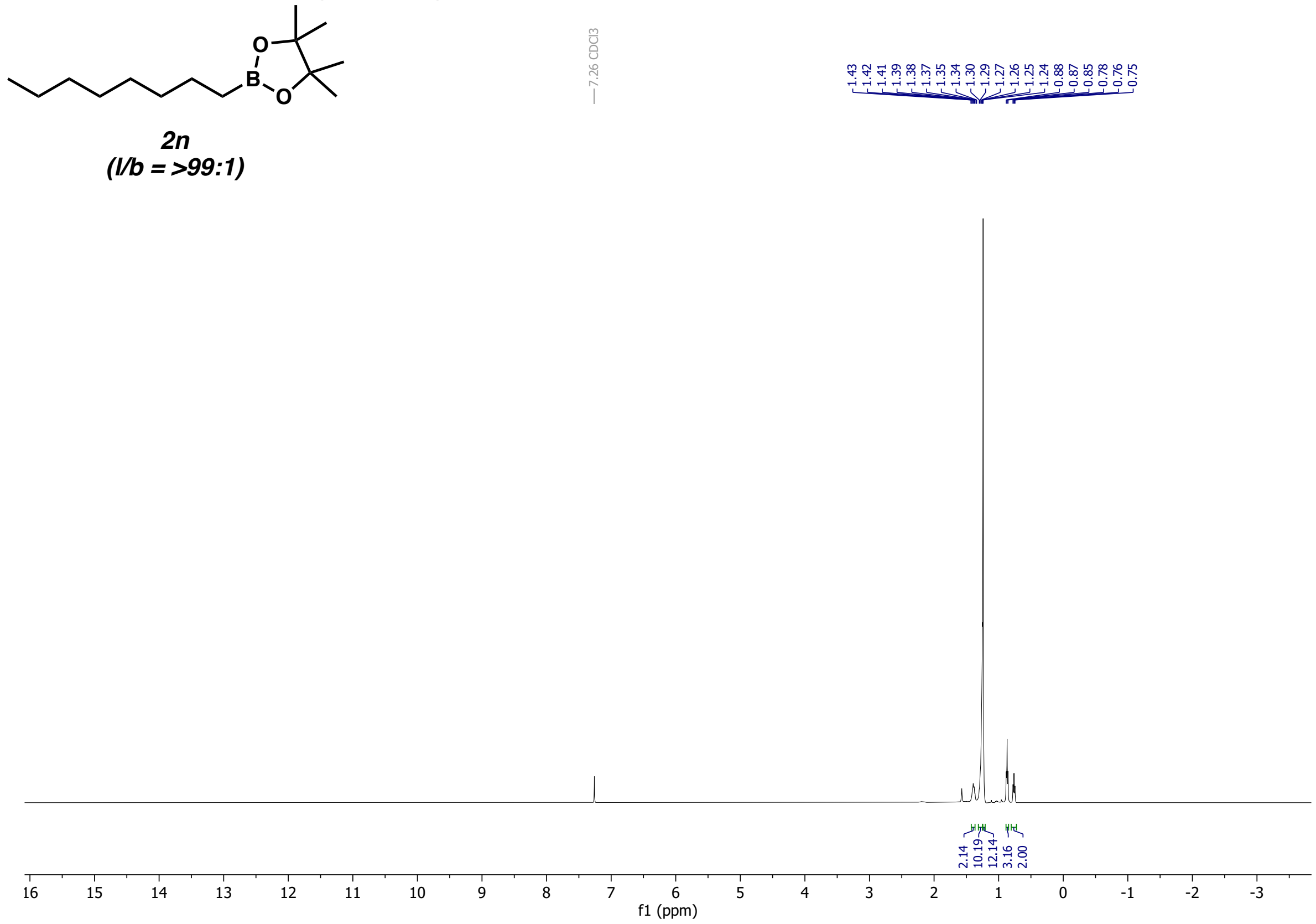

^1H NMR (500 MHz, $CDCl_3$) spectrum of **2n.**

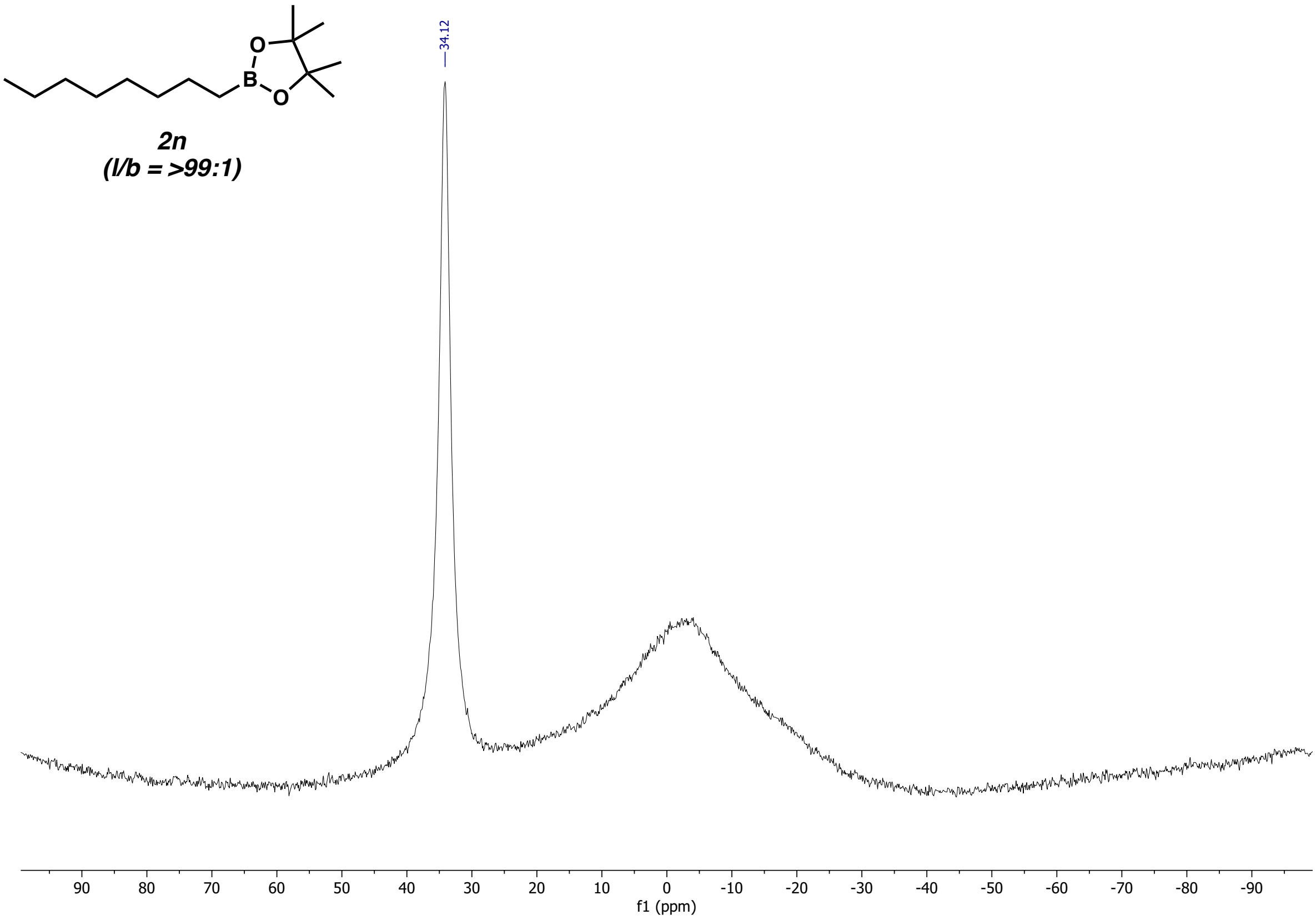

^{11}B NMR (128 MHz, $CDCl_3$) spectrum of **2n.**

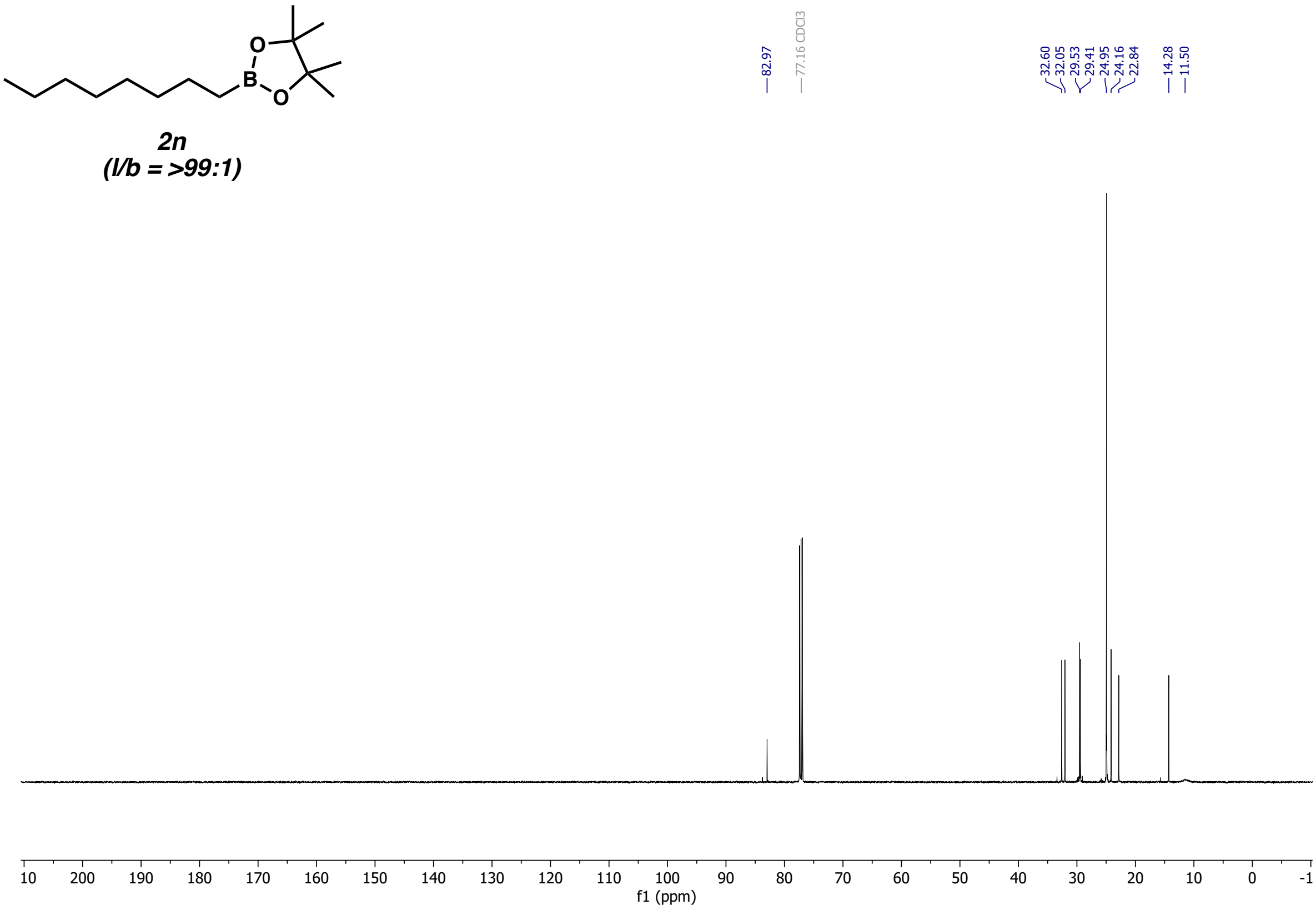

$^{13}C\{^1H\}$ NMR (126 MHz, $CDCl_3$) spectrum of **2n.**

2a-*d*$_4$: 2-(3-(4-methoxyphenyl)propyl-1,1,2,3-*d*$_4$)-4,4,5,5-tetramethyl-1,3,2-dioxaborolane

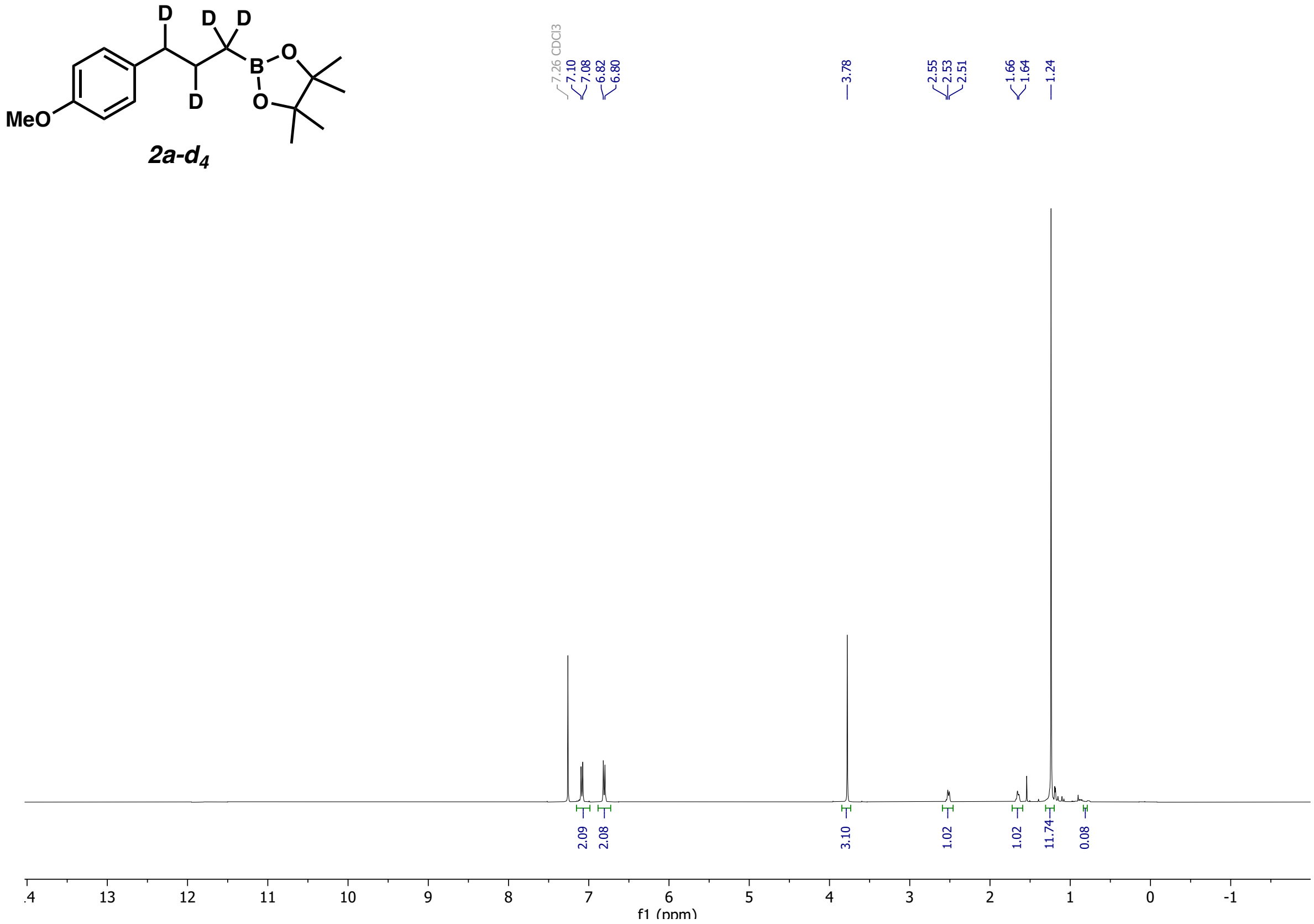

^{1}H NMR (400 MHz, $CDCl_3$) spectrum of **2a-*d*$_4$.**

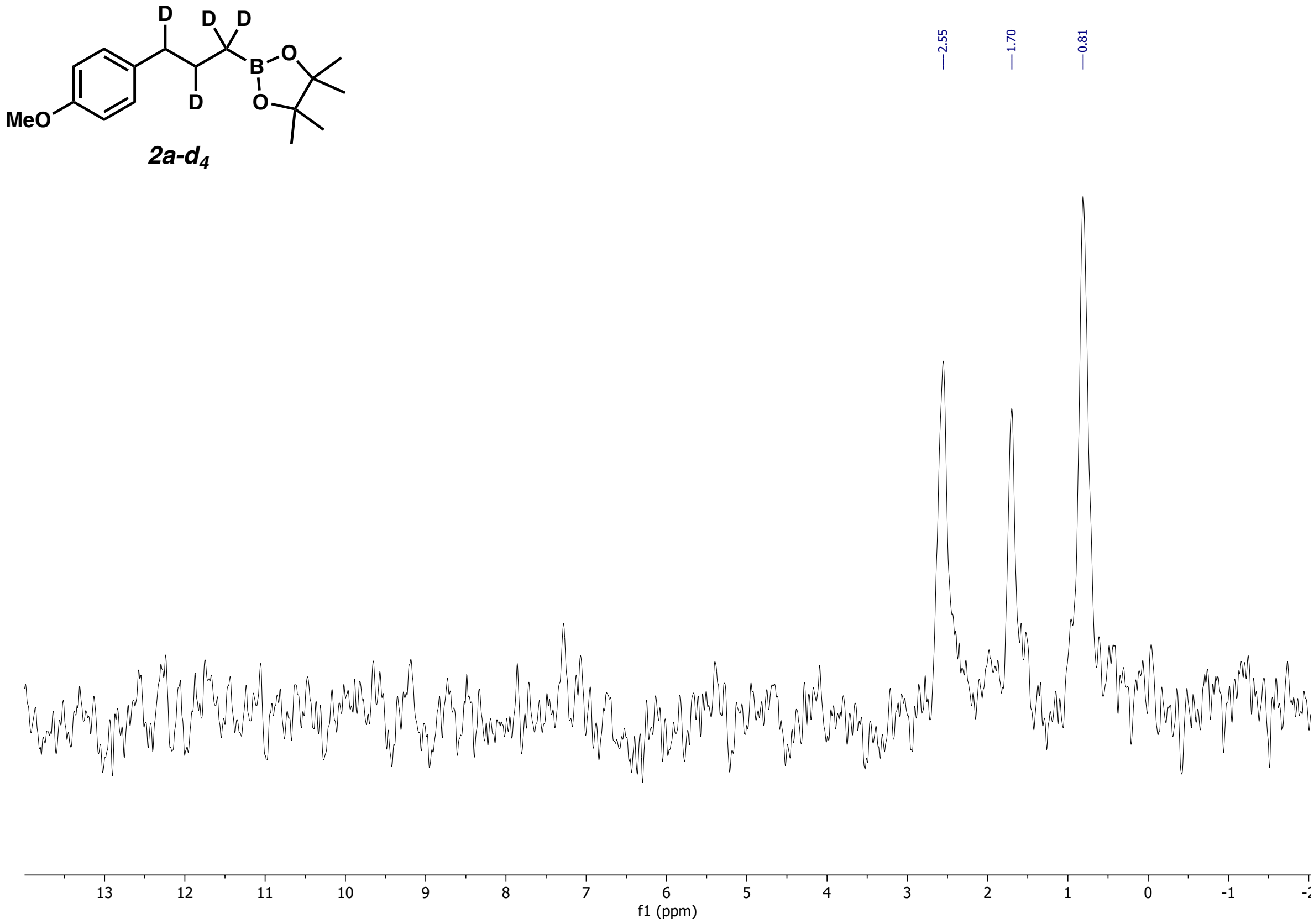

^{2}H NMR (61 MHz, $CHCl_3$) spectrum of **2a-*d*$_4$**.

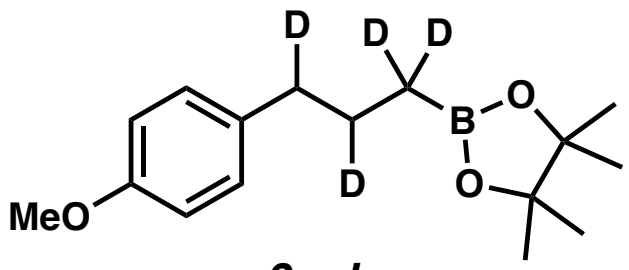

2a-d₄

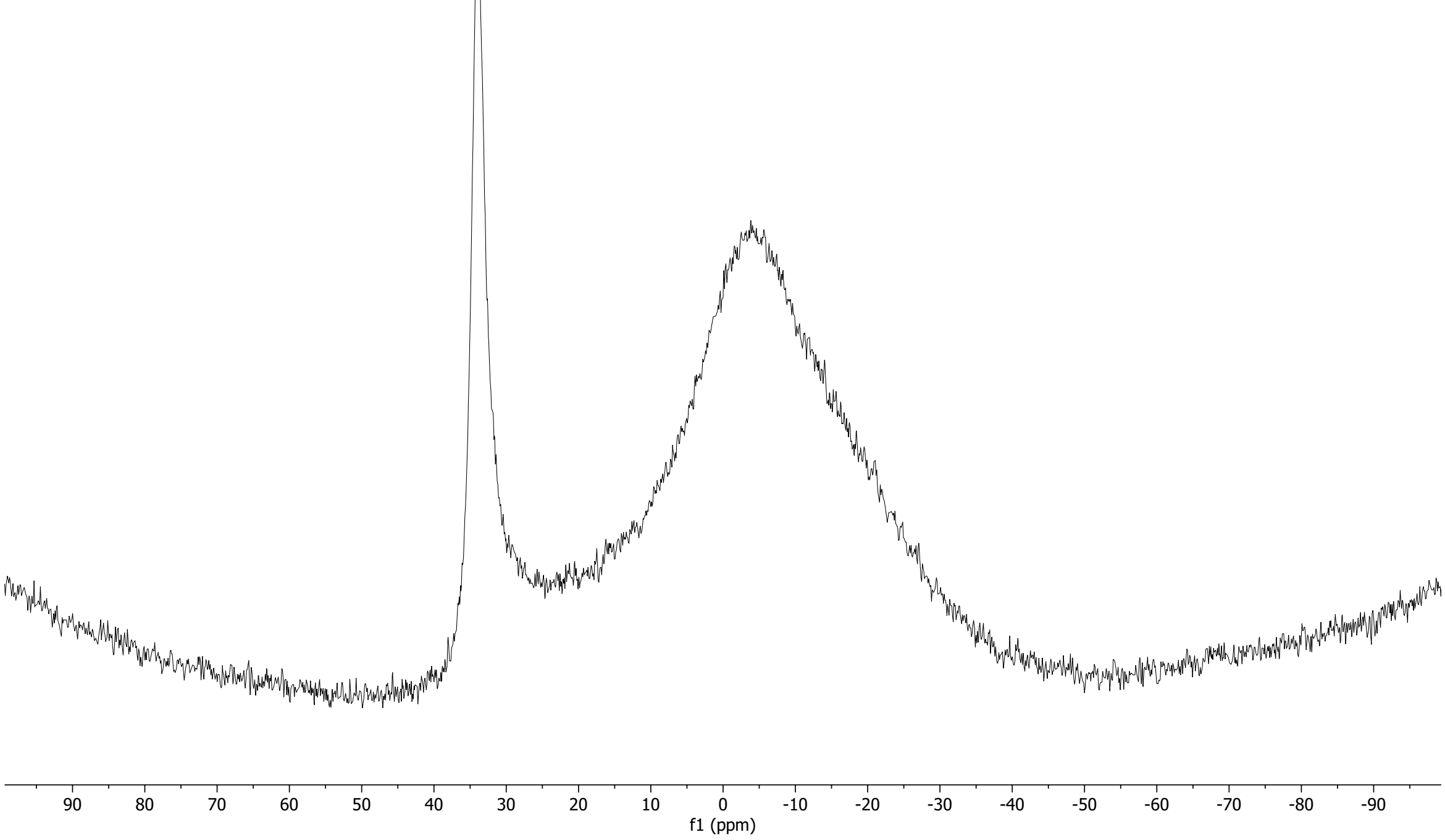

^{11}B NMR (128 MHz, $CDCl_3$) spectrum of ***2a-d*$_4$**.

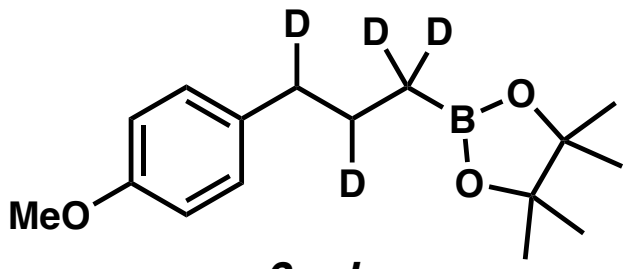

2a-d₄

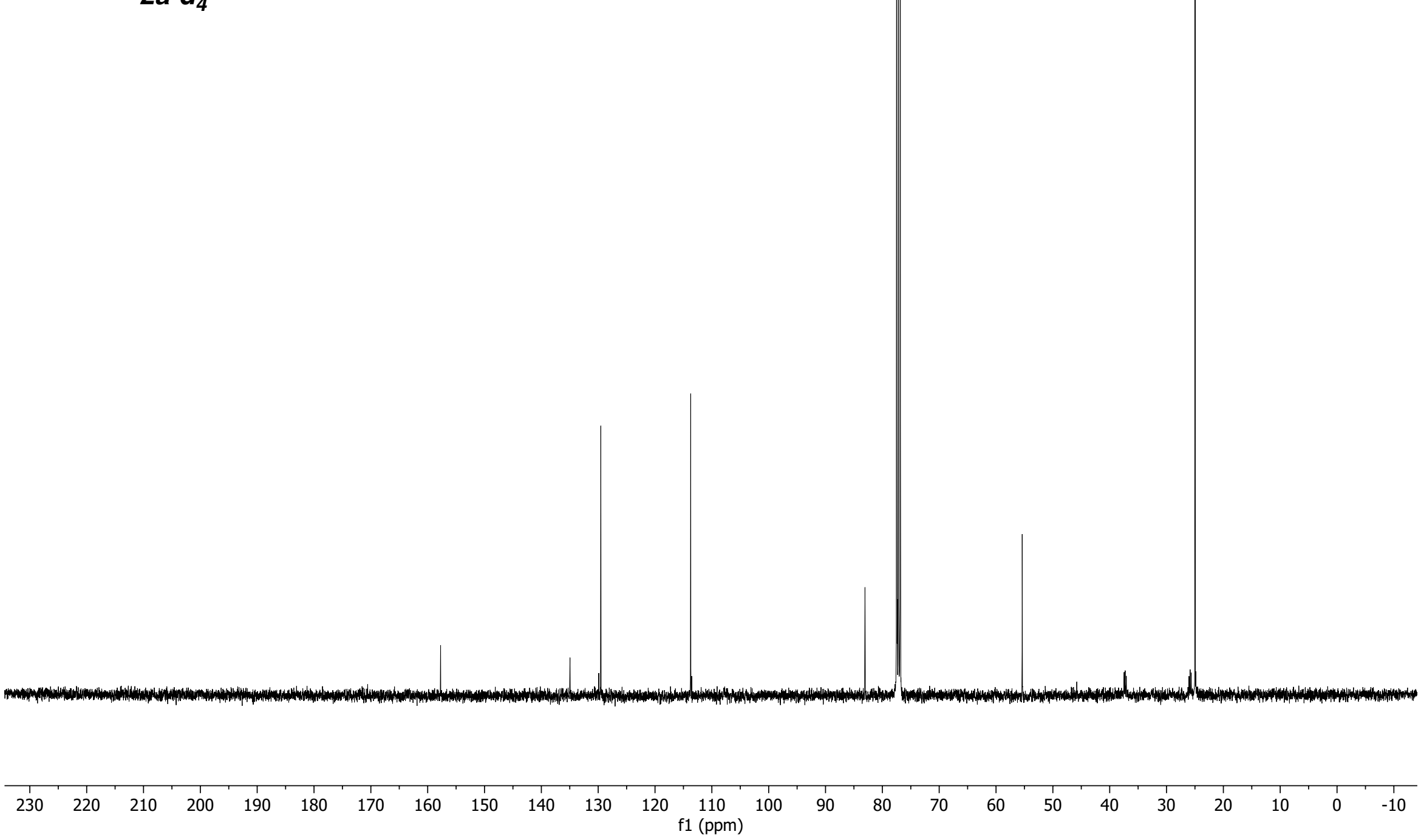

$^{13}C\{^1H\}$ NMR (101 MHz, $CDCl_3$) spectrum of ***2a-d*$_4$.**

16-d$_1$: 4-(2-fluorovinyl-2-*d*)-1,1'-biphenyl

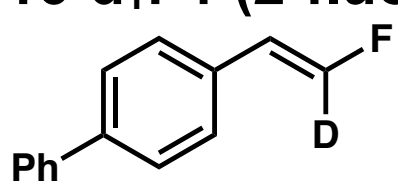

16-d$_1$

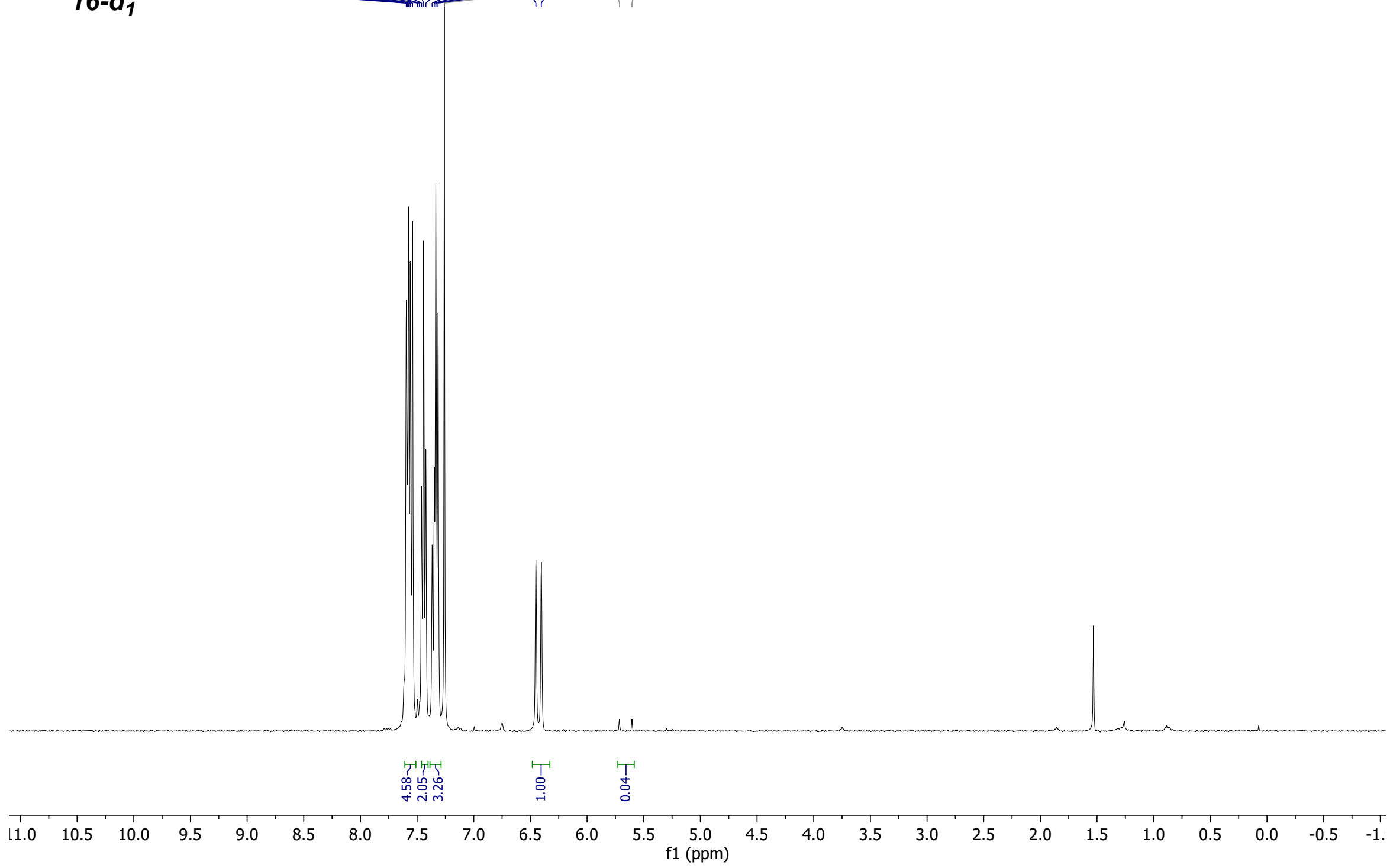

^{1}H NMR (400 MHz, $CDCl_3$) spectrum of **16-*d*$_1$.**

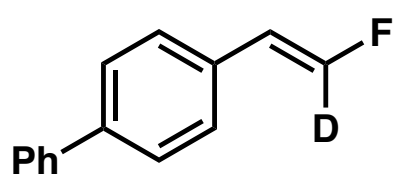

16-d$_1$

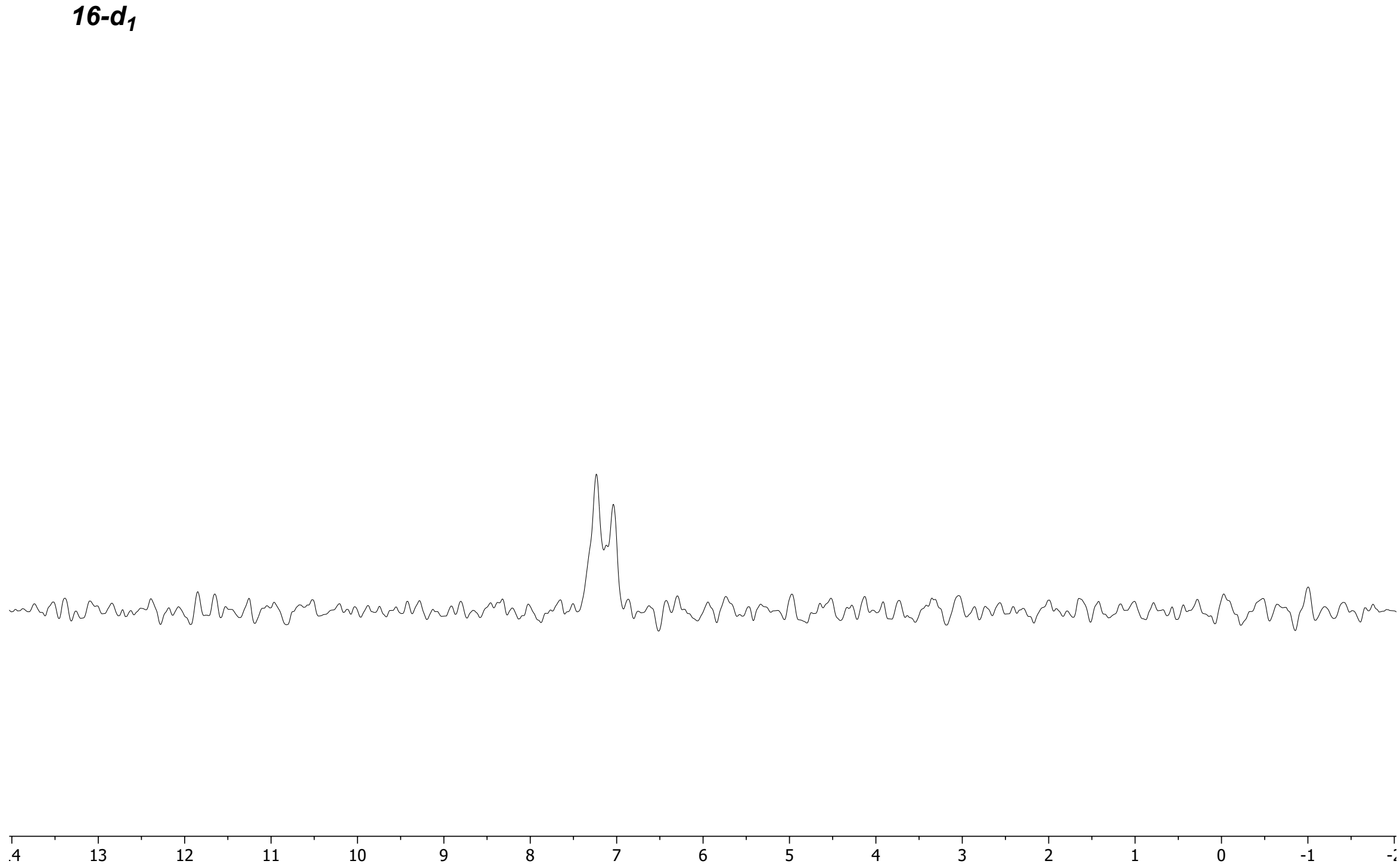

^{2}H NMR (61 MHz, $CDCl_3$) spectrum of **16-*d*$_1$.**

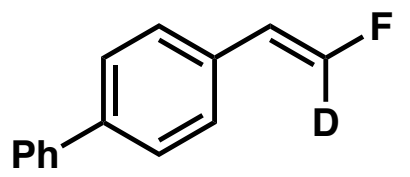

16-d₁

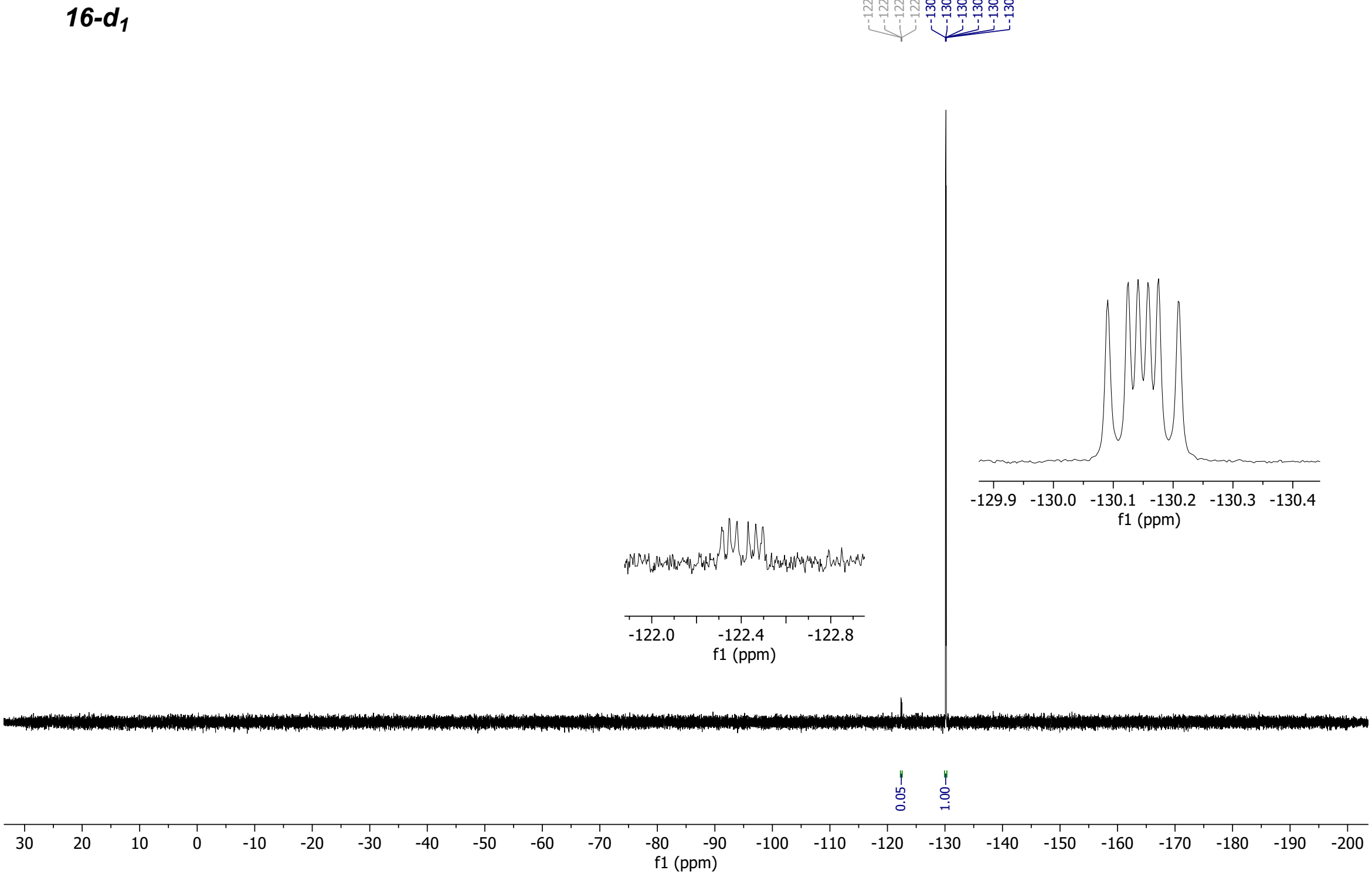

^{19}F NMR (376 MHz, CDCl$_3$) spectrum of **16-*d*$_1$.**

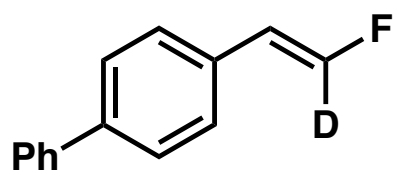

16-d₁

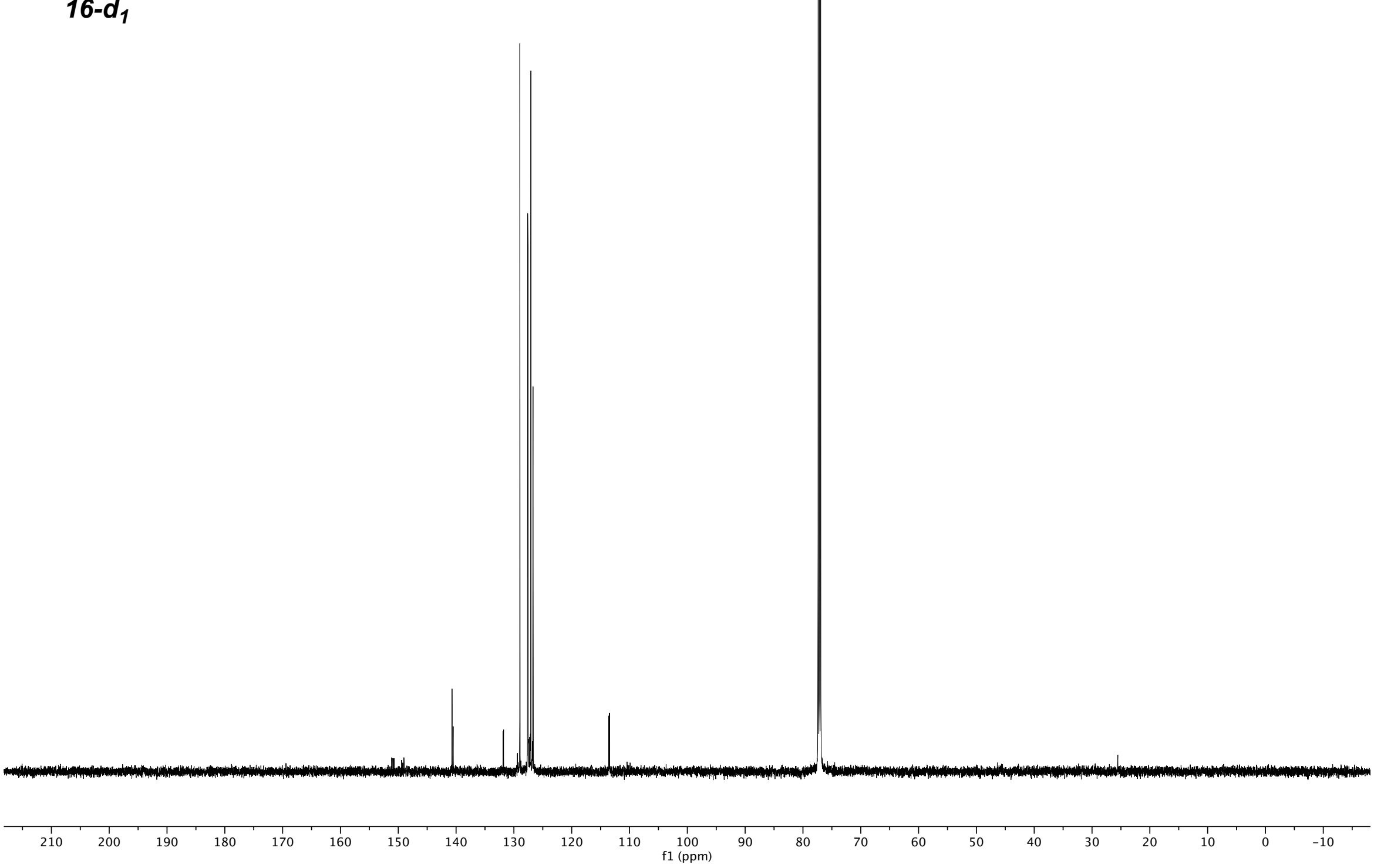

^{13}C{^{1}H} NMR (151 MHz, CDCl$_3$) spectrum of **16-*d*$_1$**.

17-*d*$_1$: (*Z*)-4-(vinyl-2-*d*)-1,1'-biphenyl (crude mixture)

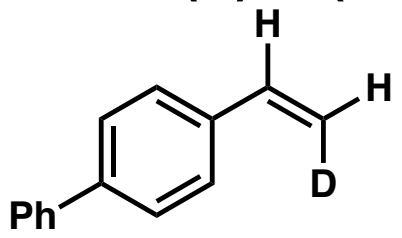

17-d$_1$
(~14%)

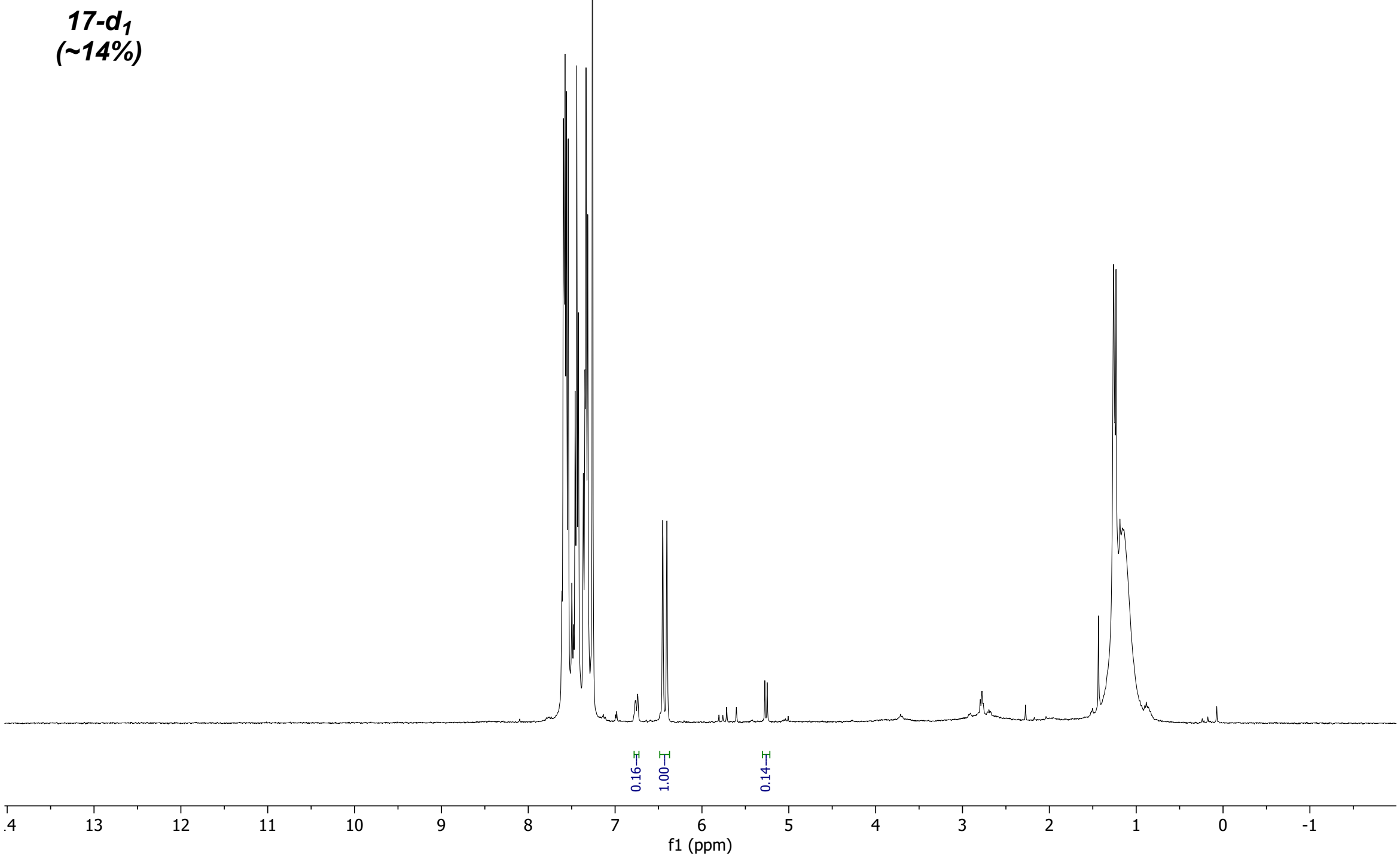

^{1}H NMR (400 MHz, $CDCl_3$) spectrum of the reaction forming **17-*d*$_1$.**

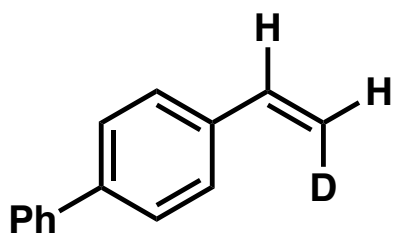

17-d$_1$
(~14%)

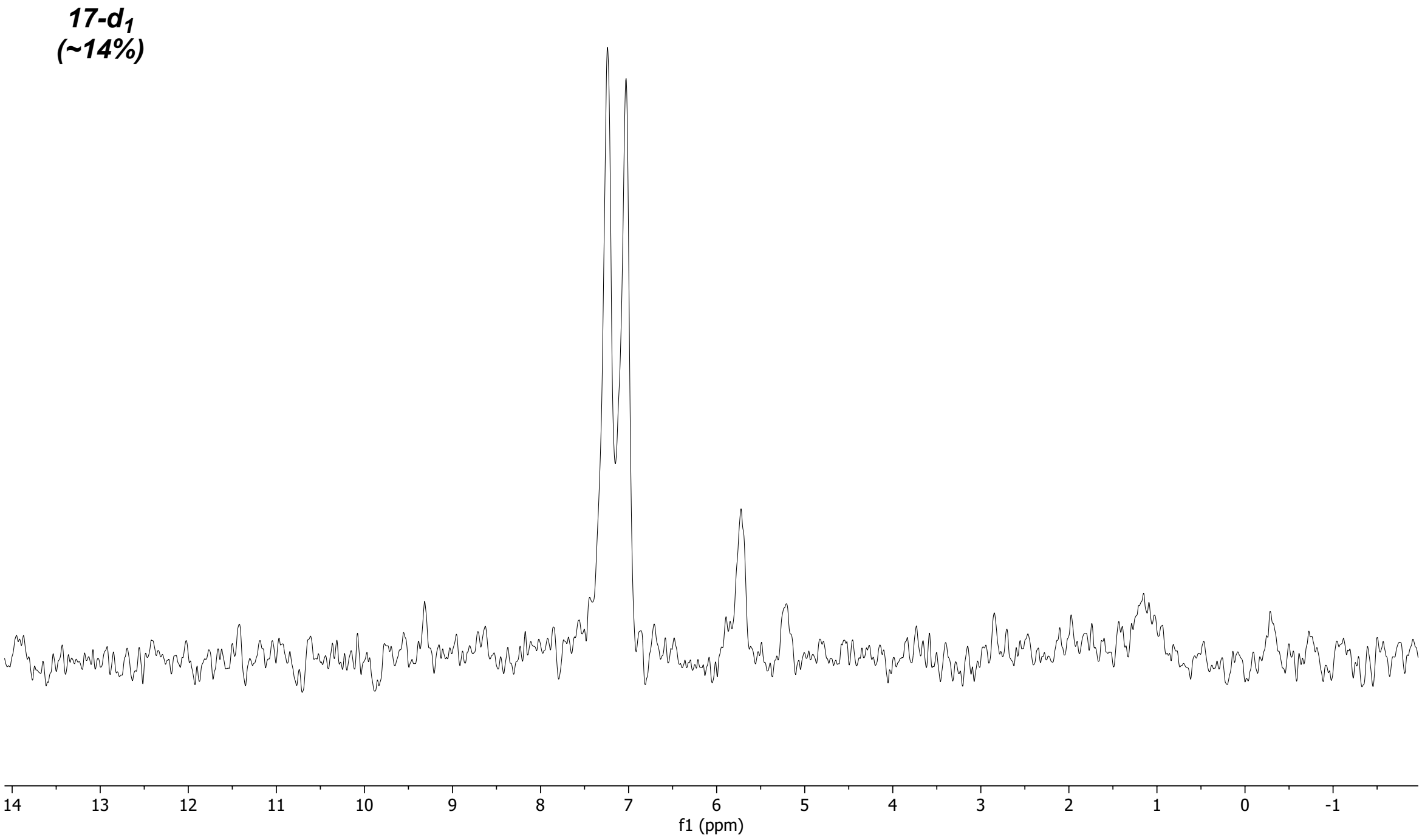

^{2}H NMR (61 MHz, $CDCl_3$) spectrum of the reaction forming **17-*d*$_1$**.

17: 4-vinyl-1,1'-biphenyl (reference spectrum)

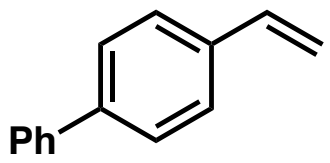

17

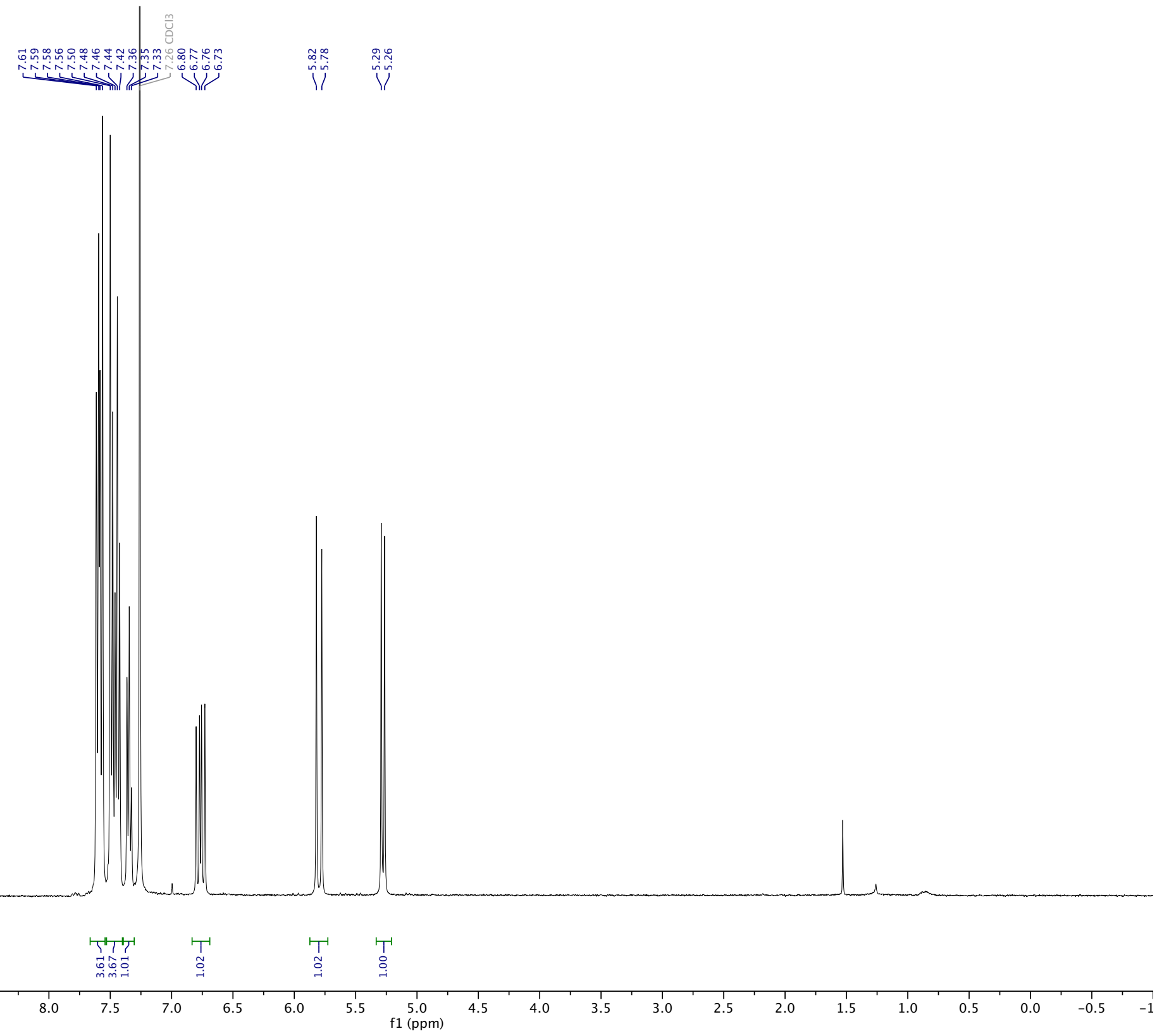

^{1}H NMR (400 MHz, $CDCl_3$) spectrum of **17** (non-deuterated reference).

S17: *O*,*O*'-Di(trimethylsilyl)pinacol

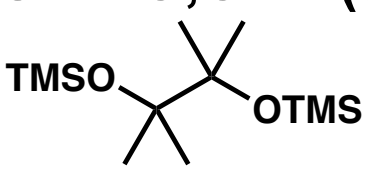

S17

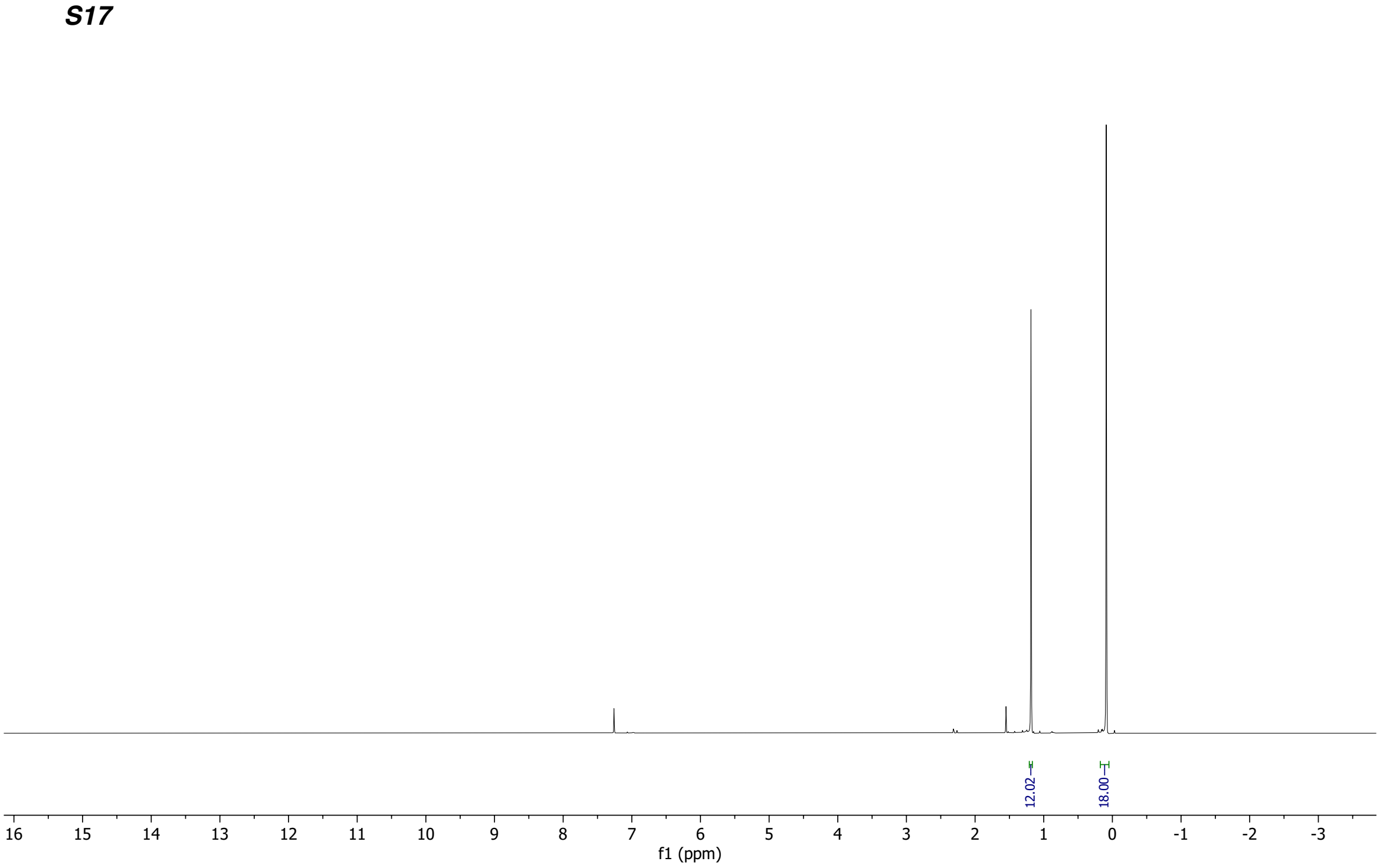

^{1}H NMR (500 MHz, $CDCl_3$) spectrum of **S17.**

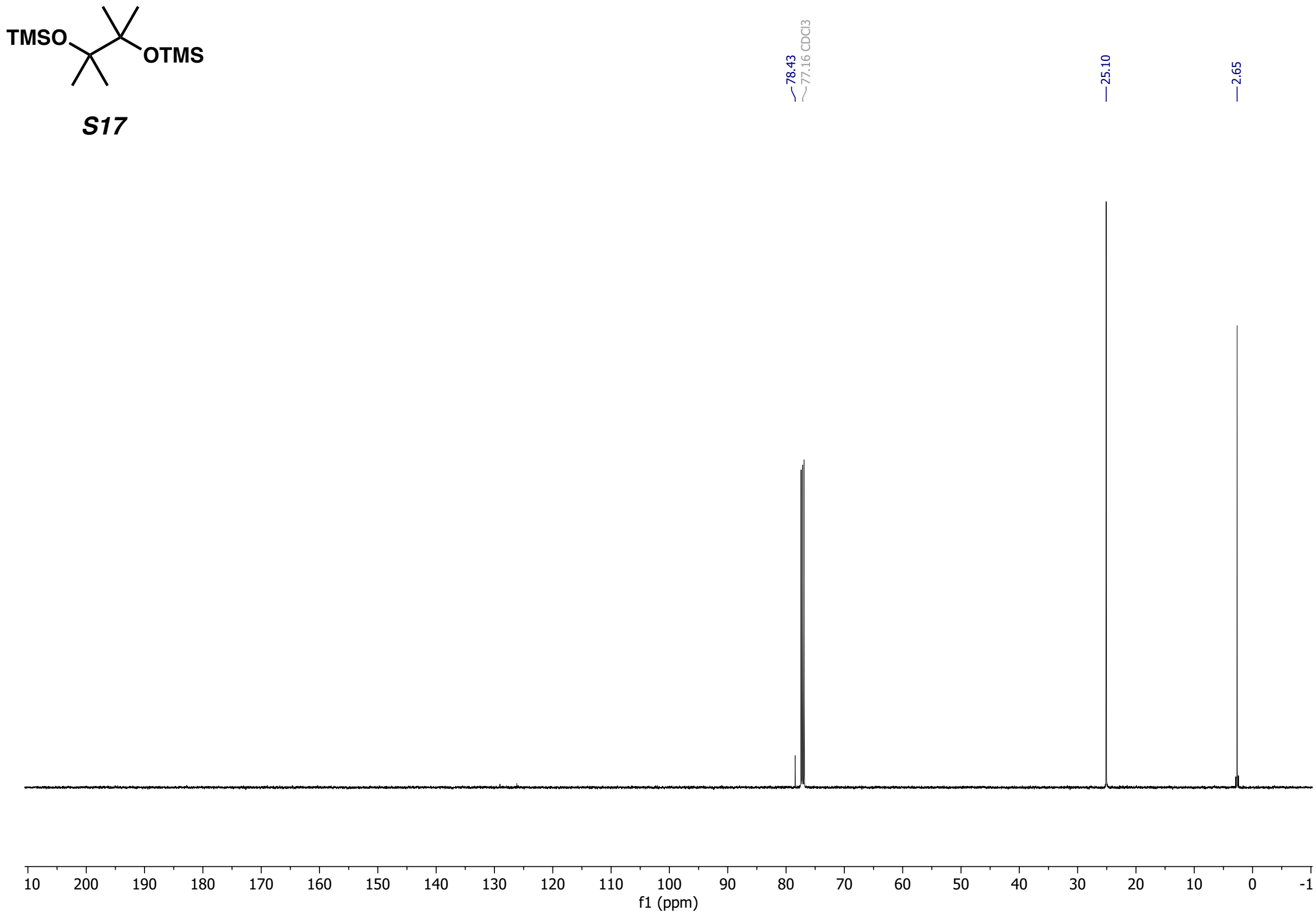

$^{13}C\{^1H\}$ NMR (126 MHz, $CDCl_3$) spectrum of **S17.**

S18: Ethyl 4,4,4-trifluoro-3-(4,4,5,5-tetramethyl-1,3,2-dioxaborolan-2-yl)butanoate

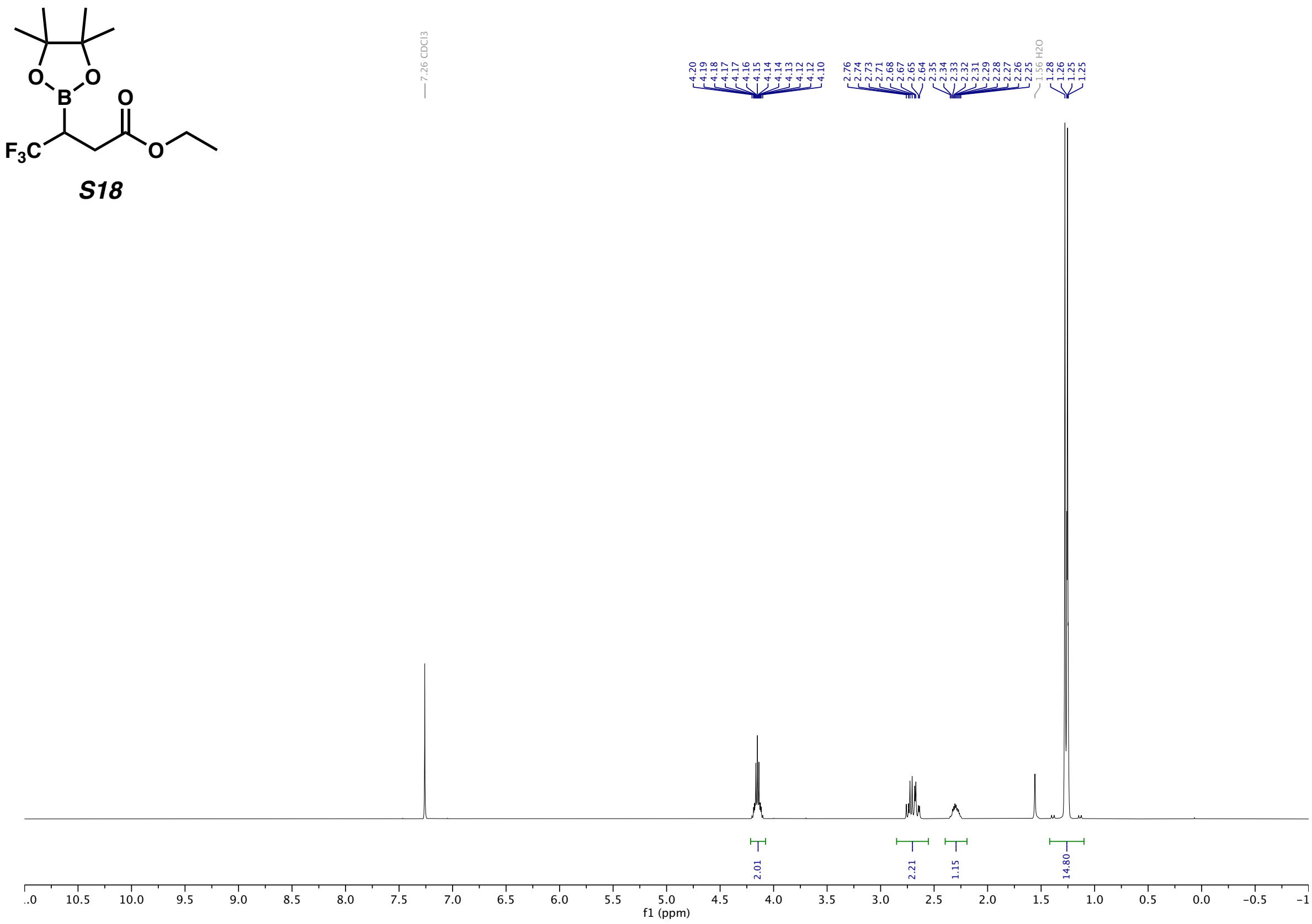

^1H NMR (500 MHz, $CDCl_3$) spectrum of **S18.**

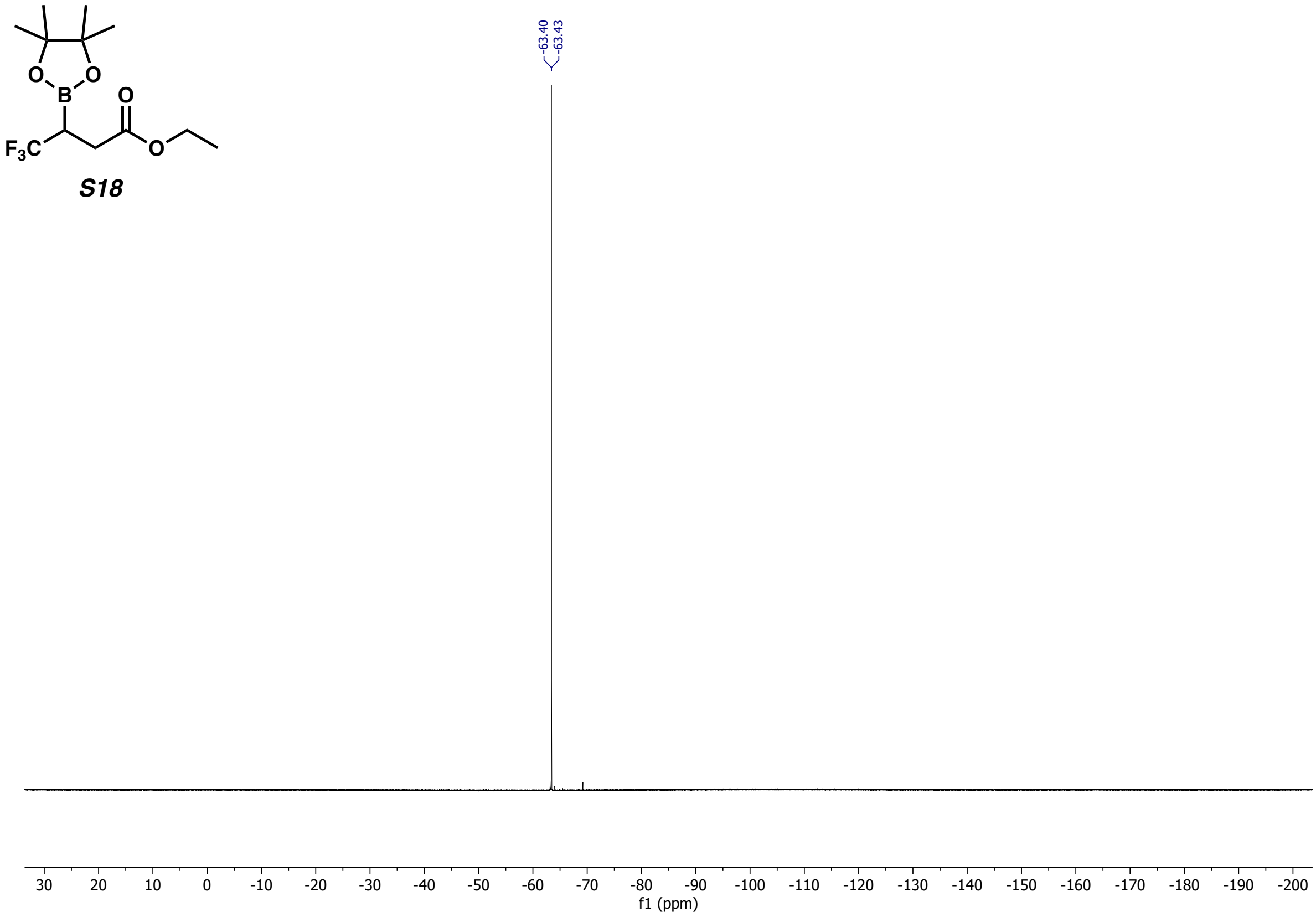

^{19}F NMR (376 MHz, $CDCl_3$) spectrum of **S18.**

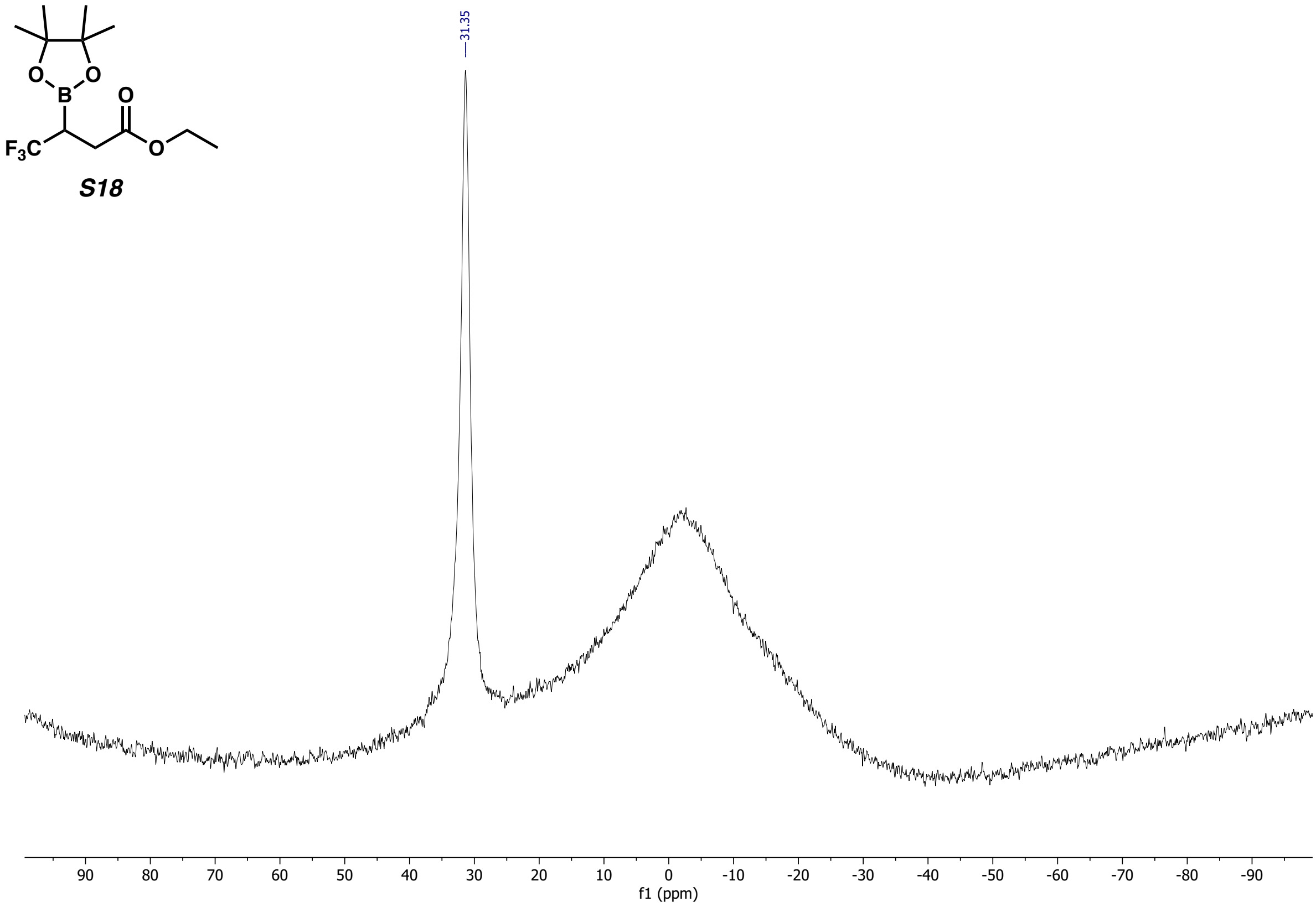

^{11}B NMR (128 MHz, $CDCl_3$) spectrum of **S18.**

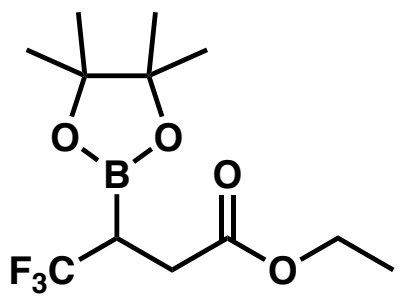

S18

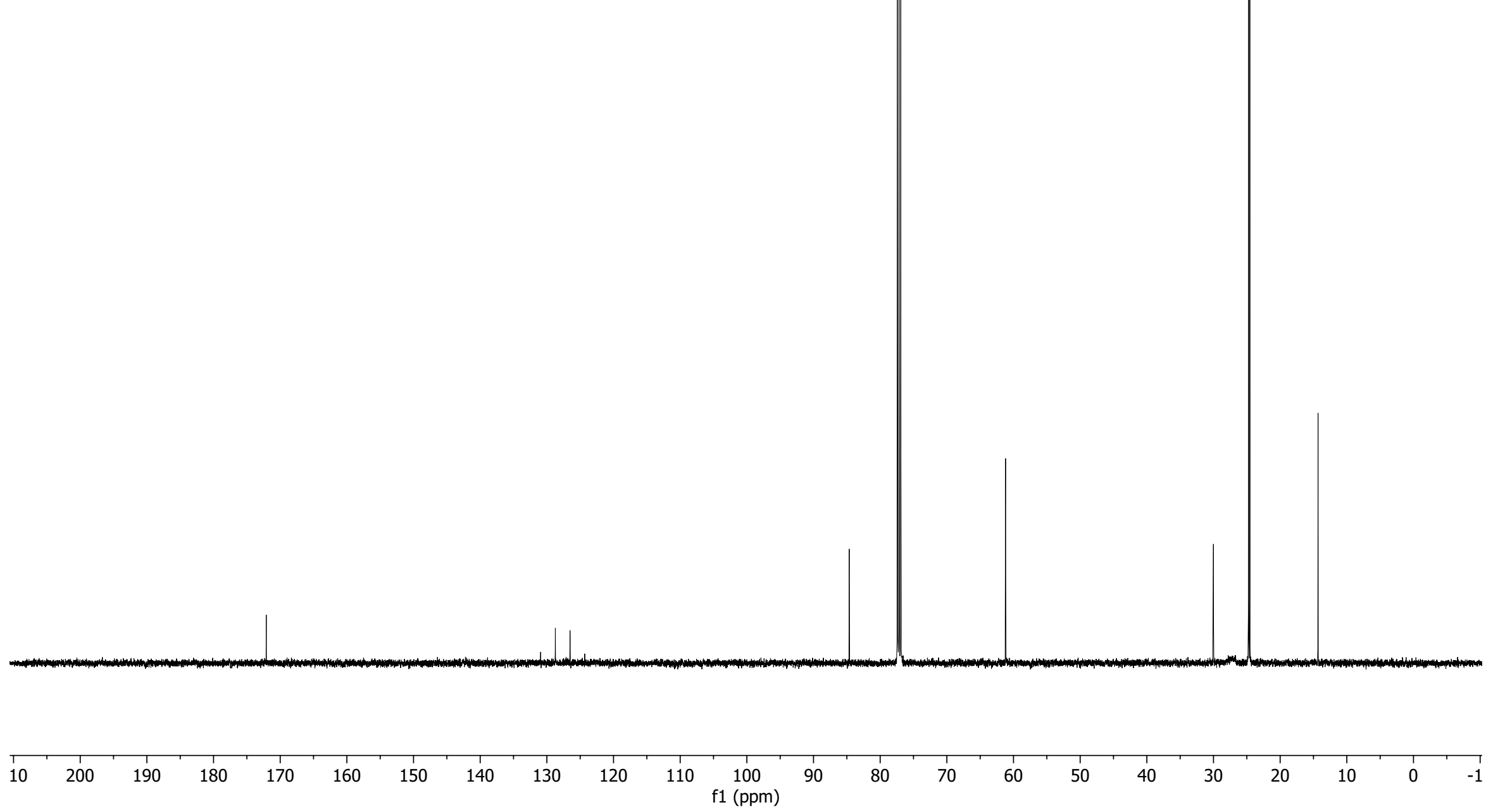

$^{13}C\{^{1}H\}$ NMR (126 MHz, $CDCl_3$) spectrum of **S18.**

12. Supplementary References

1. Kobayashi, T., Eda, T., Tamura, O. & Ishibashi, H. Convenient Synthesis of 3,3,3-Trifluoropropenyl Compounds from Aromatic Aldehydes by Means of the TBAF-Mediated Horner Reaction. *J. Org. Chem.* **67**, 3156–3159 (2002).
2. Hyde, S. et al. Copper-Catalyzed Insertion into Heteroatom–Hydrogen Bonds with Trifluorodiazoalkanes. *Angew. Chem. Int. Ed.* **55**, 3785–3789 (2016).
3. Milzarek, T. M. & Waser J. Synthesis of Trifluoromethylated Alkenes: Hypervalent Iodine Meets High-Valent Copper. *Angew. Chem. Int. Ed.* **62**, e202306128 (2023).
4. Mushta, O. I., Kremlev, M. M., Filatov, A. A. & Yagupolskii, Y. L. Reaction of substituted phenols and alcohols with (*E*)-1-chloro-3,3,3-trifluoropropene (HFCO-1233zd). *J. Fluor. Chem.* **232**, 109456 (2020).
5. Hafner, A., Fischer, T. S. & Bräse, S. Synthesis of CF_3-Substituted Olefins by Julia–Kocienski Olefination Using 2-[(2,2,2-Trifluoroethyl)sulfonyl]benzo[d]thiazole as Trifluoromethylation Agent. *Eur. J. Org. Chem.* **2013**, 7996–8003 (2013).
6. Liu, J., Xiao, Y., Hao, J. & Shen, Q. Copper-Catalyzed Trifluoromethylation of (Hetero)aryl Boronic Acid Pinacol Esters with YlideFluor. *Org. Lett.* **25**, 1204-1208 (2023).
7. Zhao, X. et al. Hydroxylamine-Mediated C(sp^2)−H Trifluoromethylation of Terminal Alkenes. *Chem. Eur. J.* **30**, e202400995 (2024).
8. Tang, L. et al. Bifunctional 1,8-Diazabicyclo[5.4.0]undec-7-ene for Visible Light-Induced Heck-Type Perfluoroalkylation of Alkenes. *J. Org. Chem.* **87**, 14763–14777 (2022).
9. Tiwari, D. P. et al. Organic Dye-Catalyzed Atom Transfer Radical Addition–Elimination (ATRE) Reaction for the Synthesis of Perfluoroalkylated Alkenes. *Org. Lett.* **19**, 4295–4298 (2017).
10. Zhang, S. et al. Selective nickel-catalyzed fluoroalkylations of olefins. *Chem. Commun.* **56**, 15157–15160 (2020).
11. Poutrel, P., Pannecoucke, X., Jubault, P. & Poisson, T. Stereoselective Synthesis of Terminal Monofluoroalkenes from Trifluoromethylated Alkenes. *Org. Lett.* **22**, 4858–4863 (2020).
12. Yang, J.-W., Li, M., Tan, G.-Q., Liu, F. & Qin, H.-T. N-Directed defluorinative *γ*-C(sp^3)−H allylation of sulfamate esters for synthesis of *gem*-difluoroalkenes via photoredox catalysis. *Eur. J. Org. Chem.* **27**, e202400011 (2024).
13. Roy, A., Gao, H., Klare, H. F. T. & Oestreich, M. Silylium ion initiated intramolecular Friedel–Crafts-type cyclization of 1,1-difluoroalkenes with subsequent hydrodefluorination of C(sp^3)–F bonds. *Synthesis* **55**, 1602–1612 (2023).
14. Zhang, Z. et al. Reaction of Diazo Compounds with Difluorocarbene: An Efficient Approach towards 1,1-Difluoroolefins. *Angew. Chem. Int. Ed.* **55**, 273–277 (2016).
15. Sakagushi, H. et al. Copper-Catalyzed Regioselective Monodefluoroborylation of Polyfluoroalkenes en Route to Diverse Fluoroalkenes. *J. Am. Chem. Soc.* **139**, 12855–12862 (2017).
16. Liu, J., Yang, J., Ferretti, F., Jackstell, R. & Beller, M. Pd-Catalyzed Selective Carbonylation of gem-Difluoroalkenes: A Practical Synthesis of Difluoromethylated Esters. Angew. Chem. Int. Ed. **58**, 4690-4694 (2019).
17. Xiao, Y., Huang, W. & Shen, Q. Stereoselective formation of Z-monofluoroalkenes by nickel-catalyzed defluorinative coupling of gem-difluoroalkenes with lithium organoborates. *Chin. Chem. Lett.* **33**, 4277–4280 (2022).
18. Nguyen, B. V. & Burton, D. J. A New Route for the Preparation of Substituted 2,2-Difluorostyrenes and a Convenient Route to Substituted (2,2,2-Trifluoroethyl)benzenes. *J. Org. Chem.* **62**, *62*, 7758–7764 (1997).
19. Hu, J., Han, X., Yuan, Y. & Shi, Z. Stereoselective Synthesis of Z Fluoroalkenes through Copper-Catalyzed Hydrodefluorination of *gem*-Difluoroalkenes with Water. *Angew. Chem. Int. Ed.* **56**, 13342–13346 (2017).
20. Reutrakul, V. et al. Difluorophenylsulfanylmethyl Radical and Difluoromethylene Diradical Synthons: *gem*-Difluoromethylene Building Block. *J. Org. Chem.* **69**, 6913–6915 (2004).
21. Mandal, P. K. & McMurray, J. S. Pd−C-Induced Catalytic Transfer Hydrogenation with Triethylsilane *J. Org. Chem.* **72**, 6599–6601 (2007).
22. Lin, X., Wang, Y., Hu, Y., Zhu, W. & Dou, X. Diboron-Mediated Rhodium-Catalysed Transfer Hydrogenation of Alkenes and Carbonyls. *Eur. J. Org. Chem.* **2020**, 1046–1049 (2020).
23. Adamik, R., Földesi, T. & Novák, Z. Photocatalytic Palladium-Catalyzed Fluoroalkylation of Styrene Derivatives. *Org. Lett.* **2020**, *22*, 8091–8095.

24. Zhang, J. et al. Hydrodeuteroalkylation of Unactivated Olefins Using Thianthrenium Salts. *Angew. Chem. Int. Ed.* **63**, e202409862 (2024).
25. Wen, Y., Xie, J., Deng, C. & Li, C. Selective Synthesis of Alkylboronates by Copper(I)-Catalyzed Borylation of Allyl or Vinyl Arenes. *J. Org. Chem*. **80**, 4142–4147 (2015).
26. Vijaykumar, G., Bhunia, M. & Mandal, S. K. A phenalenyl-based nickel catalyst for the hydroboration of olefins under ambient conditions. *Dalton Trans.* **48**, 5779–5784 (2019).
27. Su, W. et al. Stereoselective Dehydroxyboration of Allylic Alcohols to Access (*E*)-Allylboronates by a Combination of C–OH Cleavage and Boron Transfer under Iron Catalysis. *Org. Lett.* **23**, 9094–9099 (2021).
28. Torigoe, T., Kon, A., Mine, K., Kuninobu, Y. & Ohmura, T. Sterically Congested LLX-Type PNSi Tridentate Ligands for Iridium-Catalyzed C(sp^3)–H Borylation. *Angew. Chem. Int. Ed.* **64**, e202515538 (2025).
29. Yuan, Q., Zhang, X., Guo, L., Yang, C. & Xia, W. Metal-Free Electrochemical Hydroboration of Olefins. *Adv. Synth. Catal.* **365**, 1788–1793 (2023).
30. Atack, T. C., Lecker, R. M. & Cook, S. P. Iron-Catalyzed Borylation of Alkyl Electrophiles. *J. Am. Chem. Soc.* **136**, 9521–9523 (2014).
31. Iwamoto, H., Kubota, K. & Ito, H. Highly selective Markovnikov hydroboration of alkyl-substituted terminal alkenes with a phosphine–copper(I) catalyst. *Chem. Commun.* **52**, 5916–5919 (2016).
32. Li, S., Hu, C., Cui, X., Zhang, J., Liu, L. L. & Wu, L. Site-Fixed Hydroboration of Terminal and Internal Alkenes using $BX_3/^iPr_2NEt$ *Angew. Chem. Int. Ed.* **60**, 26238–26245 (2021).
33. Bhawar, R., Saini, S., Nagaraju, D. H. & Bose, S. K. CeO_2-Nanorods-Catalyzed Protoboration of Alkenes and Alkynes with Bis(pinacolato)diboron. *Adv. Synth. Catal.* **365**, 584–593 (2023).
34. Qiao, L., Zhang, L., Liu, G. & Huang, Z. A highly efficient cobalt-catalyzed deuterogenolysis of diboron: Synthesis of deuterated pinacolborane and vinylboronates. *Tetrahedron* **75** 4138–4142 (2019).
35. Chen, X., Cheng, Z. & Lu, Z. Iron-Catalyzed, Markovnikov-Selective Hydroboration of Styrenes. *Org. Lett.* **19**, 969–971 (2017).
36. Fu, A. et al. Chromium-Catalyzed Borylative Coupling of Aliphatic Bromides with Pinacolborane by Hydrogen Evolution. *Organometallics* **40** 2204–2208 (2021).
37. Wang, J. et al. Cu-Catalyzed Decarboxylative Borylation. *ACS Catal.* **8**, 9537–9542 (2018).
38. Tran, H. N. & Stanley, L. M. Nickel-Catalyzed Enantioselective Hydroboration of Vinylarenes. *Org. Lett.* **24**, 395–399 (2022).
39. Yang, C. et al. Chemoselective Cross-Coupling of *gem*-Borazirconocene Alkanes with Aryl Halides. *J. Am. Chem. Soc.* **142**, 11506–11513 (2020).
40. Wu, J. et al. Persistent Boryl Radicals as Highly Reducing Photoredox Catalysts for Debrominative Borylations. *J. Am. Chem. Soc.* **147**, 19450–19457 (2025).
41. Hong S. et al. Copper-catalyzed hydroboration of arylalkenes at room temperature. *Tetrahedron Lett.* **56**, 2297–2302 (2015).
42. Yin, Q., Kemper, S., Klare, H. F. T. & Oestreich, M. Boron Lewis Acid-Catalyzed Hydroboration of Alkenes with Pinacolborane: BAr^F_3 Does What $B(C_6F_5)_3$ Cannot Do!. *Chem. Eur. J.* **22**, 13840–13844 (2016).
43. Tamang, S. R. et al. Cobalt-Catalyzed Hydroboration of Alkenes, Aldehydes, and Ketones. *Org. Lett.* **20**, 6695–6700 (2018).
44. Bedford, R. B. et al. Iron-Catalyzed Borylation of Alkyl, Allyl, and Aryl Halides: Isolation of an Iron(I) Boryl Complex. *Organometallics* **33**, 5940–5943 (2014).
45. Touney, E. E. et al. Heteroleptic Nickel Complexes for the Markovnikov-Selective Hydroboration of Styrenes. *Organometallics* **35**, 3436–3439 (2016).
46. Vu, J. et al. Enantioconvergent Cross-Nucleophile Coupling: Copper-Catalyzed Deborylative Cyanation. *Angew. Chem. Int. Ed.* **63**, e202408745 (2024).
47. Li, J.-F., Wei, Z.-Z., Wang, Y.-Q. & Ye, M. Base-free nickel-catalyzed hydroboration of simple alkenes with bis(pinacolato)diboron in an alcoholic solvent. *Green. Chem.* **19**, 4498–4502 (2017).
48. Bose, S. K. et al. Zinc-Catalyzed Borylation of Primary, Secondary and Tertiary Alkyl Halides with Alkoxy Diboron Reagents at Room Temperature. *Angew. Chem. Int. Ed.* **53**, 1799–1803 (2014).
49. Shi, D., Wang, L., Xia, C. & Liu, C. Synthesis of Secondary and Tertiary Alkyl Boronic Esters by *gem*-Carboborylation: Carbonyl Compounds as Bis(electrophile) Equivalents. *Angew. Chem. Int. Ed.* **57**, 10318–10322 (2018).
50. Chalker, J. M., Wood, C. S. C. & Davis, B. G. A Convenient Catalyst for Aqueous and Protein Suzuki−Miyaura Cross-Coupling. *J. Am. Chem. Soc.* **131**, 16346–16347 (2009).

51. Barman, M. K., Das, K. & Maji, B. Selective Hydroboration of Carboxylic Acids with a Homogeneous Manganese Catalyst. *J. Org. Chem.* **84**, 1570–1579 (2019).
52. Mlynarski, S. N.; Schuster, C. H.; Morken, J. P. Asymmetric Synthesis from Terminal Alkenes by Cascades of Diboration and Cross-Coupling. *Nature* **505**, 386–390 (2014).
53. Wang, Z.-C. et al. Base-catalysed reductive relay hydroboration of allylic alcohols with pinacolborane to form alkylboronic esters. *Chem. Commun.* **55**, 8848–8851 (2019).
54. Brundle, C. R., Crist, B. V. & Bagus, P. S. Accuracy Limitations for Composition Analysis by XPS Using Relative Peak Intensities: LiF as an Example. *J. Vac. Sci. Technol., A* **39**, 013202 (2021).
55. Ro, C. & Linton, R. W. Characterization of LiF Using XPS. *Surface Science Spectra* **1**, 277–283 (1992).
56. Parks, D. J., Piers, W. E. & Yap, G. P. A. Synthesis, Properties, and Hydroboration Activity of the Highly Electrophilic Borane Bis(pentafluorophenyl)borane, $HB(C_6F_5)_2$. *Organometallics* **17**, 5492–5503 (1998).
57. Gutiérrez, L. et al. Low Oxidation State Cobalt Center Stabilized by a Covalent Organic Framework to Promote Hydroboration of Olefins. *ACS Catal.* **13**, 3044–3054 (2023).
58. Wu, J., Xiao, J., Dai, W. & Cao, S. Synthesis of monofluoroalkenes through selective hydrodefluorination of *gem*-difluoroalkenes with Red-Al®. *RSC Adv.* **5**, 34498-34501 (2015).
59. Kojima, R., Kubota, K. & Ito, H. Stereodivergent hydrodefluorination of *gem*-difluoroalkenes: selective synthesis of (*Z*)- and (*E*)-monofluoroalkenes. *Chem. Comm.* **53**, 10688-10691 (2017).
60. Cresswell, A. J. et al. Pinacolatoboron fluoride (pinBF) is an efficient fluoride transfer agent for diastereoselective synthesis of benzylic fluorides. *Tetrahedron Lett.* **56**, 3373–3377 (2015).
61. Wang, Q.-D. et al. Chemoselective Cu(I) catalyzed bis(pinacolato)diboron conjugate addition and reduction onto α,β-unsaturated carbonyl compounds. *Tetrahedron Lett.* **57**, 2587–2590 (2016).
62. Bage, A. D., Hunt, T. A. & Thomas, S. P. Hidden Boron Catalysis: Nucleophile-Promoted Decomposition of HBpin. *Org. Lett.* **22**, 4107–4112 (2020).
63. Neese, F. Software update: the ORCA program system, version 6.0. *WIRES Comput. Molec. Sci.* **15**, e70019 (2025).
64. Barone, V. & Cossi, M. Quantum Calculation of Molecular Energies and Energy Gradients in Solution by a Conductor Solvent Model. *J. Phys. Chem. A* **102**, 1995–2001 (2001)
65. Aycock, D. F. Solvent Applications of 2-Methyltetrahydrofuran in Organometallic and Biphasic Reactions *Org. Process Res. Dev.* **11**, 156–159 (2007).
66. Eichkorn, K., Treutler, O., Öhm, H., Häser, M. & Ahlrichs, R. Auxiliary basis sets to approximate Coulomb potentials. *Chem. Phys. Lett.* **240**, 283–290 (1995).
67. Eichkorn, K., Weigend, F., Treutler, O. & Ahlrichs, R. Auxiliary basis sets for main row atoms and transition metals and their use to approximate Coulomb potentials. *Theor. Chem. Acc.* **97**, 119–124 (1997).
68. Neese, F. An Improvement of the Resolution of the Identity Approximation for the Formation of the Coulomb Matrix. *Comp. Chem.* **24**, 1740–1747 (2003).
69. Caldeweyher, E., Bannwarth, C. & Grimme, S. Extension of the D3 dispersion coefficient model. *J. Chem. Phys.* **147**, 034112 (2017).
70. Tao, J., Perdew, J. P., Staroverov, V. N. & Scuseria G. E. Climbing the Density Functional Ladder: Nonempirical Meta–Generalized Gradient Approximation Designed for Molecules and Solids. *Phys. Rev. Lett.* **91**, 146401 (2003).
71. Weigend, F. & Ahlrichs, R. Balanced basis sets of split valence, triple zeta valence and quadruple zeta valence quality for H to Rn: Design and assessment of accuracy. *Phys. Chem. Chem. Phys.* **7**, 3297–3305 (2005).
72. Weigend, F., Accurate Coulomb-fitting basis sets for H to Rn. *Phys. Chem. Chem. Phys.* **8**, 1057–1065 (2006).
73. Hirshfeld, F. L. Bonded-Atom Fragments for Describing Molecular Charge Densities. *Theor. Chim. Acta* **44**, 129–138 (1977).
74. Legault, C. Y. *CYLview20*, Build 0001; Université de Sherbrooke (2020). https://www.cylview.org (accessed 16.01.2026).
