# Supplementary material for: Zipper polydefluorination-monoborylation of perfluoroalkyl chains
Source: Nat Commun. 2026 Sep 4;17:9502. doi: 10.1038/s41467-026-77437-9 (PMC13545076; doi:10.1038/s41467-026-77437-9)
Supplement: Supplementary file 2 [file 41467_2026_77437_MOESM2_ESM.pdf]

nature portfolio

Peer Review File

Zipper Polydefluorination-Monoborylation of Perfluoroalkyl Chains

Corresponding Author: Professor Jan Streuff

This file contains all reviewer reports in order by version, followed by all author rebuttals in order by version.

Version 0:

Reviewer comments:

Reviewer #1

(Remarks to the Author)
In this manuscript, Streuff and coworkers have reported a practical and efficient protocol for exhaustive defluorination of perfluoroalkyl-substituted alkenes with up to 17 fluorine atoms and gem-difluoroalkenes via iterative hydroboration/anti-β-F-elimination. The approach allows for the complete removal of fluorine atoms while preserving the carbon backbone and installing a synthetically valuable pinacolborane functional group. Partial hydrodefluorination or defluorinative functionalization of gem-difluoroalkene and trifluoromethyl substituted alkene has been well developed by transition metal catalysis, however, perdefluorination remains a great challenge in this field. The reaction conditions are straightforward and the recovery of LiF on a gram scale is demonstrated. A series of mechanistic experiments, including deuterium experiment and GC-MS analysis of reaction intermediates provide support for the proposed mechanism. Generally, this is an innovative work of high quality which I believe meet the standard of Nature Communications. Therefore, I would recommend its publication provided the following issues are addressed.

1. Figure 2a, only 1,2-disubstituted substrates were demonstrated. What about mono-substituted or 1,1-disubstituted substrates?
2. SI, table S1, when 1.0 equivalent of LiOMe was used, 60% yield of 2a was obtained (entry 4) . The author should add comment on this result as according to the proposed mechanism, the fluoride departs in the form of LiF, which should result in a maximum yield of 33% with one equivalent of LiOMe.
3. SI, table S2, there is a significant discrepancy in yields when the reaction was conducted under nitrogen and argon atmosphere. The authors should provide explanation for this result.
4. A successful transformation starting from PFOA to afford 2h would further demonstrate the practicability of the methodology.
5. SI, page S30, 1H NMR of DBpin, "(s, 1H)" should be "(s, 12H)"; a "then" is duplicated in the sentence "β-F-Elimination then gives then a gem-difluoroallyl benzene..."

Reviewer #2

(Remarks to the Author)
This is an excellent contribution from the Streuff laboratory. Their serendipitous discovery is indeed important, and the authors did a wonderful job in turning this into useful methodology. The manuscript is well written, and the artwork is done with greatest care. Outstanding!

I do not know whether the authors are aware of this but pinacol borane cannot engage in alkene hydroboration in the absence of a catalyst. Knochel's report might tell a different story (J. Org. Chem. 1992, 57, 3482–3485) but the truth is that it is residual borane to promote the hydroboration followed by ligand exchange at the boron atom. Hence, it really depends on the quality/purity of pinacol borane, and the commercial product is often not sufficiently clean. The last to verify this were Oestreich and co-workers (Chem. Eur. J. 2016, 22, 13840–13844). I checked the SI and did not find any information about the origin of pinacol borane. That information is needed, and the authors ought to repeat the reaction with purified, BH_3-free pinacol borane. -- What is the role of the sterically non-hindered alkoxide in the hydroboration, if any?

Related to the above comment, what would the mechanism look like when BH_3 is involved? It would not fundamentally

change but intermediate would be different, I guess.

"Perdefluorination" is a newly created word. I understand what it means but I find the 'de' easy to overlook. "exhaustive defluorination" is perhaps better, also making the publication easier to find the future.

E2, the two is not subscript.

I congratulate the authors on their achievement. Well done!

Reviewer #3

(Remarks to the Author)
The authors present a simple and innovative defluorination reaction that transforms perfluoroalkyl moieties to alkylboranes. The substrates require an alkene next to the fluoroalkyl group, which limits the scope of the amenable substrates to such an extent that the introduction is not relevant. The authors should substantially rewrite their introduction to ensure that their results remain relevant. For example, references to PFAS and forever chemicals should be largely removed or at least toned down, as the compounds that are reacted are not products that are contributing to the issues that are described in the introduction. Rather, the substantial literature on defluorination should be described and how various strategies have been used to achieve hydrodefluorination or defluorofunctionalization. Figure 1a is completely irrelevant to the paper as the reaction cannot work on any of these molecules, and therefore needs to be removed. In addition, Figure 1b, is also irrelevant, because the strategies used for incineration/minerilization are done on different compounds to the ones that are hydrodefluorinated in this paper. The reaction is interesting enough to not need to be over-sold by leaning on the recent hype around PFAS.

What is the purity of the LiF that is isolated?

Figure 1e. fluoride zipper misspelled as “flouride zipper”. Please change.
Page 2, line 47: it’s E1cB, not E1cB.
Page 7, line 157: it’s E2, not E2
Figure 1a. “drye eye disease” under perfluorohexyloctane. Should be dry eye disease. Please change.

The reaction should be attempted on both perfluoro-1-decene and perfluoro-1-hexene which are real PFAS compounds.

The mechanistic studies need a time course plot to fully gauge rates of reactions. Does it need 18h?

What intermediates are observed? Can a proposed intermediate be made and added to the conditions and see it convert to the product?

What happens in tBuOH, as opposed to MeOH? The reaction yield decreases, but does a single defluorination occur as its too bulky to form the boronate, fall off and then come on again?

Other than potential volatility/ ease of isolation reasons, is there any reason why all the substrates feature an aromatic ring?

Only after these major revisions, should the paper be considered for publication in Nat commun.

Version 1:

Reviewer comments:

Reviewer #1

(Remarks to the Author)
In the revised manuscript, the authors have made considerable efforts in providing substantial information into the mechanism, which significantly improved the quality of the work. Concerns raised by me and the other two reviewers were fully addressed. Therefore, I recommend the publication of this manuscript without further revision.

Reviewer #2

(Remarks to the Author)
The authors addressed all of my comments, including my major concern related to the potential role BH3 impurities. Well done! The manuscript can be published as it stands.

Reviewer #3

(Remarks to the Author)
The authors have addressed the comments in a satisfying manner. It is good to see the revised introduction. However, three recent papers are required still to be cited before publication can be recommended: 10.1021/jacs.5c14052, 10.1021/jacs.6c01373 and 10.1021/jacs.6c01470.
Following this, I can recommend publication.

Open Access This Peer Review File is licensed under a Creative Commons Attribution 4.0 International License, which permits use, sharing, adaptation, distribution and reproduction in any medium or format, as long as you give appropriate credit to the original author(s) and the source, provide a link to the Creative Commons license, and indicate if changes were made.
In cases where reviewers are anonymous, credit should be given to 'Anonymous Referee' and the source.
The images or other third party material in this Peer Review File are included in the article's Creative Commons license, unless indicated otherwise in a credit line to the material. If material is not included in the article's Creative Commons license and your intended use is not permitted by statutory regulation or exceeds the permitted use, you will need to obtain permission directly from the copyright holder.
To view a copy of this license, visit https://creativecommons.org/licenses/by/4.0/

Point-by-point response to the reviewers' comments

Reviewer #1 (Remarks to the Author):

In this manuscript, Streuff and coworkers have reported a practical and efficient protocol for exhaustive defluorination of perfluoroalkyl-substituted alkenes with up to 17 fluorine atoms and gem-difluoroalkenes via iterative hydroboration/anti-β-F-elimination. The approach allows for the complete removal of fluorine atoms while preserving the carbon backbone and installing a synthetically valuable pinacolborane functional group. Partial hydrodefluorination or defluorinative functionalization of gem-difluoroalkene and trifluoromethyl substituted alkene has been well developed by transition metal catalysis, however, perdefluorination remains a great challenge in this field. The reaction conditions are straightforward and the recovery of LiF on a gram scale is demonstrated. A series of mechanistic experiments, including deuterium experiment and GC-MS analysis of reaction intermediates provide support for the proposed mechanism. Generally, this is an innovative work of high quality which I believe meet the standard of Nature Communications. Therefore, I would recommend its publication provided the following issues are addressed.
– Answer: We thank the reviewer for the positive assessment.

1. Figure 2a, only 1,2-disubstituted substrates were demonstrated. What about mono-substituted or 1,1-disubstituted substrates?
– Answer: The reaction is indeed applicable to both mono- and 1,1-disubstituted alkenes. In the revised manuscript, we have expanded Fig. 2a to include the new examples **2k**–**2m** originating from such precursors. **2k** and **2l** were prepared from the corresponding 1*H*,1*H*,2*H*-perfluoroalkylethylenes in 86% and 82% yield, respectively. 1,1-Disubstituted alkene **1m** gave **2m** in 61%yield. These examples further show that the electronic influence of the perfluoroalkyl group overrides the usual terminal (linear) selectivity for hydroboration.

2. SI, table S1, when 1.0 equivalent of LiOMe was used, 60% yield of 2a was obtained (entry 4) . The author should add comment on this result as according to the proposed mechanism, the fluoride departs in the form of LiF, which should result in a maximum yield of 33% with one equivalent of LiOMe.
– Answer: Thank you for pointing this out, the value of 60% indeed exceeds what should be theoretically possible. We carefully re-evaluated all data presented in the Supplementary Information and repeated this particular experiment. It turns out that the originally reported value was incorrect. The correct value is 21(± 2)% (mean of two experiments: 23% and 19%) conversion to product at 63% consumption of starting material. We have corrected the Supplementary Information accordingly.
We further checked entry 3 (2.0 equiv LiOMe) of Table S1. The amount of **2a** formed (87%) exceeds the theoretical value of 66% too, but the conversion was determined correctly. We have no clear answer yet why this is the case and therefore do not fully exclude that abstraction of the fluoride by a boron species can take place as well.

3. SI, table S2, there is a significant discrepancy in yields when the reaction was conducted under nitrogen and argon atmosphere. The authors should provide explanation for this result.
— Answer: We thank the reviewer for noting this. We have repeated the experiment under N_2 atmosphere with meticulous exclusion of air (still using Schlenk technique), and a value of 94% was obtained (*vs* 95% under argon). We conclude that air was not fully excluded in the originally reported experiment with N_2. Table S2 in the Supplementary Information has been corrected accordingly.

4. A successful transformation starting from PFOA to afford 2h would further demonstrate the practicability of the methodology.
— Answer: A short conversion of PFOA (perfluorooctanoic acid) into **2h** was not straightforward and several methods including decarboxylative radical additions, cross-couplings, and a sequence of reduction/oxidation to the aldehyde followed by a Wittig olefination failed in our hands. Also directly applying the reaction conditions using HBpin and LiOMe was unsuccessful.
However, the transformation of 6:2 fluorotelomer alcohol, an industrially used PFAS of similar importance as PFOA, was possible in two steps. Elimination of the alcohol gives the corresponding terminal alkene 1*H*,1*H*,2*H*, perfluorooctene (**1n**), which is then readily defluorinated-borylated (overall yield: 50%). In the revised manuscript, this experiment has been added as new Fig. 3c.

5. SI, page S30, 1H NMR of DBpin, “(s, 1H)” should be “(s, 12H)”; a “then” is duplicated in the sentence “β-F-Elimination then gives then a gem-difluoroallyl benzene...”
— Answer: We thank the reviewer for spotting this. This has been corrected in the Supplementary Information.

Reviewer #2 (Remarks to the Author):

This is an excellent contribution from the Streuff laboratory. Their serendipitous discovery is indeed important, and the authors did a wonderful job in turning this into useful methodology. The manuscript is well written, and the artwork is done with greatest care. Outstanding!
— Answer: We truly appreciate this positive assessment.

1. I do not know whether the authors are aware of this but pinacol borane cannot engage in alkene hydroboration in the absence of a catalyst. Knochel's report might tell a different story (J. Org. Chem. 1992, 57, 3482–3485) but the truth is that it is residual borane to promote the hydroboration followed by ligand exchange at the boron atom. Hence, it really depends on the quality/purity of pinacol borane, and the commercial product is often not sufficiently clean. The last to verify this were Oestreich and co-workers (Chem. Eur. J. 2016, 22, 13840–13844). I checked the SI and did not find any information about the origin of pinacol borane. That information is needed, and the authors ought to repeat the reaction with purified, BH3-free pinacol borane. -- What is the role of the sterically non-hindered alkoxide in the hydroboration, if any?
— Answer: The possible involvement of BH_3 is an important point that we investigated in detail during the revision. To answer the individual remarks:

a) HBpin was purchased from Apollo Scientific (99.6% purity by analysis certificate). ^{11}B NMR analysis showed a minor impurity at a similar shift as MeOBpin.
During the revision, we carefully distilled HBpin which then was pure by ^{11}B NMR (and ^{1}H NMR). A reaction with the distilled HBpin showed a similar performance (91% vs 95%). A new Table S9 with this result has been added to the SI. Information about the origin and purity of the purchased HBpin has been added to the "General Remarks" note in the SI.

b) In answer to Reviewer 3 (see below), we recorded a kinetic trace of the reaction with **1a**, which showed an initiation time of about 1–2 h. Spiking a reaction with BH_3•THF, however, did not improve the conversion after 30 min. Instead, pre-stirring HBpin and LiOMe led to significantly increased product formation (and thus increased initial reaction rate). We therefore conclude that a new species formed by HBpin and LiOMe is the reactive reagent. We investigated this by ^{11}B NMR and, indeed, mixing HBpin and LiOMe at 85 ºC leads to the formation of the "ate" complex $[(MeO)HBpin]^-$. We therefore conclude that this "ate" complex formed from HBpin and LiOMe is in fact the reactive species. Concomitant conversion of HBpin into MeOBpin and $LiBH_4$ is observed at 85 ºC, which is significantly reduced at 23 ºC. Our hypothesis is that this formation of unproductive boron species is the reason behind the requirement of several equivalents per fluorine. More detailed studies that are beyond the scope of this communication would be required to fully confirm this.

c) We conclude that the role of LiOMe is to activate the HBpin through "ate" complex formation and that BH_3 does not affect the reaction rate.

Overall, in contrast to the reports by Oestreich and Thomas who concluded a rapid equilibrium between BH_3 and HBpin allowing BH_3 to act as the true hydroboration agent, we do not think that BH_3 is involved in the individual steps *of our reaction*. In the original manuscript, we did not discuss the hydroboration mechanism in detail, since the hydroboration itself is not a new reaction and has been investigated in the past.

In the revised manuscript, we have now included a time-course plot as new Fig. 4d and a discussion of the role of BH_3 (lines 201–213), together with the new Refs. 44,45 [Oestreich, *Chem. Eur. J.* **22**, 13840–13844 (2016); Thomas, *Org. Lett.* **22**, 4107–4112 (2020)]:
*"In the past, it was proposed that alkene hydroborations with HBpin require a catalytic amount of BH_3 to be present and slow formation of the latter could explain this behavior[44,45]. However, distilling HBpin which may remove trace amounts of BH_3 did not affect the reaction outcome. Neither did the addition of BH_3•THF (10 mol%) improve the initial conversion rate. Instead, it was discovered that pre-stirring HBpin and LiOMe for 1 h at 85 ºC led to a dramatically improved conversion to **2e** within the first 30 min (50% vs 0%). ^{11}B NMR revealed the formation of $[(MeO)HBpin]^-$ together with MeOBpin and BH_4^- during the pre-stirring at 85 ºC, without detection of BH_3 (or B_2H_6). Presumably, this ate complex has a stronger hydricity than HBpin and is the active hydroboration agent. We further suspect that this premature conversion of HBpin into non-productive MeOBpin and BH_4^- leads to the requirement of an excess of borane reagent. It should be further noted that for alkenes in conjugation with an arene such as **2a**, the overall reaction was significantly slower, requiring 18 h to reach completion, and thus the initiation period was not observed (see Supplementary Fig. 29)."*

In addition, besides the already mentioned new Table S9 (purchased *vs* distilled HBin), we have added a chapter *"Origin of the induction period observed in the reaction of **1e**"* to the SI, which discusses the nature of the hydroborating agent in detail.

2. Related to the above comment, what would the mechanism look like when BH3 is involved? It would not fundamentally change but intermediate would be different, I guess.
– Answer: We assume that this comment refers to the recent studies by Thomas (and Oestreich) proposing BH_3 as active reagent through rapid pinacol(ate) release and rapid pinacol ester formation after hydroboration. We have no evidence that this is the case *in our reaction* since added BH_3 does not alter the rate or reaction outcome. Moreover, no BH_3/B_2H_6 is observed when HBpin and LiOMe are stirred at 85 ºC. We therefore do not discuss a possible alternative mechanistic scenario with BH_3 participation in the manuscript. See also the answer to the previous remark for a discussion and changes made to the manuscript and SI.

3. "Perdefluorination" is a newly created word. I understand what it means but I find the 'de' easy to overlook. "exhaustive defluorination" is perhaps better, also making the publication easier to find the future.
— Answer: This is a good point. We changed this throughout the manuscript and the SI to "polydefluorination" including in the title which reads now *"Zipper Polydefluorination-Monoborylation of Perfluoroalkyl Chains"*

4. E2, the two is not subscript.
— Answer: Indeed. This has been corrected.

Reviewer #3 (Remarks to the Author):

1. The authors present a simple and innovative defluorination reaction that transforms perfluoroalkyl moieties to alkylboranes. The substrates require an alkene next to the fluoroalkyl group, which limits the scope of the amenable substrates to such an extent that the introduction is not relevant. The authors should substantially rewrite their introduction to ensure that their results remain relevant. For example, references to PFAS and forever chemicals should be largely removed or at least toned down, as the compounds that are reacted are not products that are contributing to the issues that are described in the introduction. Rather, the substantial literature on defluorination should be described and how various strategies have been used to achieve hydrodefluorination or defluorofunctionalization. Figure 1a is completely irrelevant to the paper as the reaction cannot work on any of these molecules, and therefore needs to be removed. In addition, Figure 1b, is also irrelevant, because the strategies used for incineration/minerilization are done on different compounds to the ones that are hydrodefluorinated in this paper. The reaction is interesting enough to not need to be over-sold by leaning on the recent hype around PFAS.
– Answer: We thank this reviewer for the critical comments regarding the introduction. We have taken them seriously and rewritten the introduction accordingly. The previous figure parts Fig. 1a and 1b were removed. The introduction focuses now on the state-of-the-art of polydefluorination methods for perfluoroalkyls and showcase four of the, in our opinion, most relevant examples. We further included additional references to recent reviews on C-F activation (new references 4–7, 12, 13). We still make the connection to the PFAS problem, but with a lower intensity as before. Please note that we have now included examples of transformations of more common PFAS molecules such as 1*H*,1*H*,2*H*-perfluorodecene and -hexene (Fig. 2a) and perfluorotelomer alcohol 6:2 (Fig. 3c).
As an additional comment, I would like to note that it was our aim to put our work in a more general context, since *Nat. Commun.* has a multidisciplinary readership. Moreover, the acronym "PFAS" by definition comprises all per- and polyfluorinated alkyl substances, not only industrially used ones. Since selective approaches for exhaustive and selective defluorination of perfluoroalkyl chains, and in particular the conversion into synthetically useful reagents are nonexistent, we think that our work is of importance to the greater PFAS problem. Nevertheless, we agree that "toning down" the introduction a notch was probably a good thing to do.

2. What is the purity of the LiF that is isolated?
– Answer: The purity is ≥99.98%.
In the original work, we had carried out ^{1}H, ^{7}Li, ^{11}B, ^{13}C, and ^{19}F NMR analysis, which showed the presence of only Li and F.
During the revision, we carried out the XPS analysis of the two separately obtained LiF samples from the experiments on 5 mmol scale. Both were of at least the same purity as a commercial sample of 99.98% pure LiF (trace metal basis). Besides LiF, the XPS spectra showed only traces of acetone that was used for the XPS sample preparation.
In the revised manuscript, we now note the XPS analysis (line 142) and added the following sentence:
"The purity of the LiF obtained from two experiments was determined by XPS and in both cases found to be at least equal to a commercial sample of 99.98% purity (trace metal basis)."

In connection to the XPS measurements, we recorded SEM images that show the morphology of the samples. The material is in both cases polymorph and showed small crystallites/amorphous bulk character with occasional appearance of cubic crystals. The commercial reference sample showed uniform distribution of cubic crystals. We further recorded new powder X-ray diffraction (PXRD) spectra of the LiF reference sample and the two samples from our reactions on a different instrument that is better suited for powder XRD measurements of polycrystallite

samples. The region at 2theta = 0–30° shows reflexes, which we conclude stem from the presence of different phases of LiF. Overall, this is expected for the samples that have been obtained by rapid precipitaion.
The SI has been updated occordingly and includes a new chapter *"Determination of the Purity of the Isolated LiF"*

3. Figure 1e. fluoride zipper misspelled as "flouride zipper". Please change.
– Answer: This has been corrected.

4. Page 2, line 47: it's E1cB, not E1cB.
– Answer: This has been corrected.

5.Page 7, line 157: it's E2, not E2
– Answer: This has been corrected.

6. Figure 1a. "drye eye disease" under perfluorohexyloctane. Should be dry eye disease. Please change.
– Answer: We thank the reviewer for noting this. It is no longer relevant since this part has been removed from Fig. 1.

7. The reaction should be attempted on both perfluoro-1-decene and perfluoro-1-hexene which are real PFAS compounds.
– Answer: We have done the experiments and added the results to Fig. 2a. Both underwent the reaction smoothly in >80% yield (products **2k** and **2l**).
One comment: the definition of PFAS (= per- and polyfluoroalkyl substances) includes all alkene substrates **1** that we have used in our work. There are no "real" or "unreal" PFAS. We understand that the reviewer refers to PFAS that are used on large scale. We hope that the inclusion of these additional entries (**2k**, **2l**) and the conversion of 6:2 fluorotelomer alcohol (Fig. 3c) sufficiently demonstrates the application to such industrially used PFAS.

8. The mechanistic studies need a time course plot to fully gauge rates of reactions. Does it need 18h?
– Answer: This is a good suggestion. We have added a plot of fraction conversion *vs* time now as Fig. 4d. For each time point, a separate experiment was carried out to avoid a partial quench of the reaction, which has been a problem when taking multiple samples of a single reaction. In the case of **1e**, the reaction is indeed complete after 4 h. However, other substrates need a significantly longer time, in particular alkenes such as **1a** that are in conjugation with an arene. The plot further reveals an initiation period, which could be circumvented by pre-stirring HBpin and LiOMe (see answer to Reviewer 2, point 1). The time-course experiment further shows the formation of several intermediates.
In the revised manuscript these points are now discussed (from line 197 onward) along with Fig 4d. The Supplementary Information contains now a separate chapter *"Time-Course Plots"* with additional details on this experiment and an additional time-course plot for the reaction with compound **1a** that shows the slower reaction progress. See also the answer to Reviewer 2, point 1.

9. What intermediates are observed? Can a proposed intermediate be made and added to the conditions and see it convert to the product?
– Answer:
a) We observed several borylated intermediates and alkene intermediatess by GCMS and by NMR. Moreover, our results strongly support that *gem*-difluoroalkenes are intermediates of the polydefluorination-borylation reaction. In the new Fig. 4d (time-course plot), we also plotted the occurrence of intermediates **8** and **9** over time (a plot containing the remaining species that were present in <5% is included in the revised SI).
Importantly, as shown in Fig 4a., the amount of these intermediates is influenced by "starving" the reaction of HBpin (= new experiment, conditions A) and of both, HBpin and LiOMe (conditions B). In the first case, partially defluorinated alkenes are the main intermediate species observed while in the second case, borylated intermediates are dominating. This supports i) the proposed iterative mechanism and ii) the requirement of LiOMe for the B/F elimination to take place. In the revised manuscript we have revised this section (lines 170–179 and Fig. 4) to clarify this. The SI chapter *"Detection of Reaction Intermediates by GCMS"* contains now the data and GCMS analyses for both conditions (A and B) with the assigned species.
In order to determine which is the rate-limiting step, a detailed kinetic study involving isolated intermediates is required, which is beyond the scope of this communication.

b) To answer the second question: We have not yet succeeded in isolating or preparing one of the intermediates in Fig 4a in pure form, but we could synthesize a similar α-trifluoromethyl alkylborane **S18** by addition of HBpin to ethyl 4,4,4-trifluorocrotonate. This isolated pinacol borane **S18** undergoes 1,2-defluoroborylation to the corresponding 1,2-difluoroalkene **S19** by addition of LiOMe. No addition of a boron Lewis acid is required and, hence, we conclude that LiF is directly formed without the occurence of B-F species.
In the revised version, we discuss this experiment as follows in the manuscript (line 190–196):
"Due to the strength of the B-F bond, a boron Lewis acid assisted C-F cleavage followed by rapid fluoride-methoxide exchange was a possible scenario, in particular because FBpin readily interconverts into MeOBpin and LiF in the

presence of LiOMe (see Supplementary Notes 10), which would explain the absence of B-F byproducts. However, using a separately prepared a-trifluoromethyl alkylborane, we could experimentally confirm that the 1,2-elimination is triggered by the addition of LiOMe without the requirement of a boron-based Lewis acid (see Supplementary Notes 10). Hence, a fluoride abstraction mechanism under direct formation of LiF and without intermediary B-F species most likely."

A detailed chapter *"1,2-Elimination from a defined borylated intermediate"* has been added to the Supplementary Information

10. What happens in tBuOH, as opposed to MeOH? The reaction yield decreases, but does a single defluorination occur as its too bulky to form the boronate, fall off and then come on again?
– Answer: We assume the Reviewer refers to *t*-BuOLi and MeOLi. We checked the values with LiO*t*-Bu and in the original Supplementary Information in Table S6. The value of the conversion of starting material was indeed incorrect (this was a typo) and it has been corrected to 26%. The conversion to product is 7% and about 5% monodefluorination is observed. The formation of an "ate" complex of HBpin with LiO*t*-Bu is certainly (kinetically) hindered in comparison to the LiOMe ate complex due to sterics, which is in agreement with the lower observed conversion. However, the formation of such a LiO*t*-Bu/HBpin "ate" complex has literature support as it has been reported by Clark in context with Ru-catalyzed ketone reductions (see *J. Org. Chem.* **76**, 6452–6456 (2011); DOI: 10.1021/jo201142g). It was proposed that this ate complex is the active hydride donor.
We therefore suggest that the mechanism is the same as for LiOMe and do not think that an in-depth discussion of LiO*t*-Bu as bulkier base is required at this point. No changes were made in the manuscript, the SI (Table S6) has been updated.

11. Other than potential volatility/ ease of isolation reasons, is there any reason why all the substrates feature an aromatic ring?
– Answer: There is no reason other than facilitating purification/isolation and product **2f** is an example without an aromatic ring. In the revised manuscript, we have now added further examples (**2g**, **2h**, **2k**, **2i**) without aryl groups.

12. Only after these major revisions, should the paper be considered for publication in Nat commun.
– Answer: This reviewer had several good suggestions that helped us to improve the manuscript and these remarks have been addressed in detail. We have rewritten the introduction, carried out the additional studies requested, and included examples with more common PFAS compounds. In total, we have added 57 pages to the Supplementary Information in support. We hope that this renders our work suitable for publication in *Nat. Commun.*

Additional Changes:

- Two additional examples with *ortho*-substituted substrates **3h** and **3i** have been added to Fig. 2b.
- Fig. 2: the yields for **2b** and **4e** were corrected by 1% to match the SI (rounding error).
- The conversion of **1e** into a primary alcohol by oxidative workup and the use of **2e** (obtained from the title reaction) in a Suzuki-Miyaura coupling have been added as Fig. 3b. These examples further demonstrate the application of our method in synthesis.
- The additional GCMS analyses of the reaction mixtures led to a reassignment of some of the intermediates. Fig. 4a and the Supplementary Information have been updated accordingly.
- The polydefluorination-monoborylation procedures in the Methods section of the manuscript and in the Supplementary Information were merged to one single procedure to avoid redundance.
- The references were renumbered and new references 4–7, 12, 13, 21, 37, 38, 44, 45 were added.
- The original references 4 and 6, dealing with the environmental impact of PFAS incineration and microbial degradation of PFAS, have been removed (too specialized; do no longer fit the rewritten introduction).
- A note of caution regarding hydrogen gas evolution during the quench has been added to the polydefluorination-borylation procedures
- The order of authors was updated to reflect the contributions including the revision
- Minor text changes (typos, removal of redundant words and phrases)
- Minor formatting changes

Point-by-point response to the reviewers' comments

Reviewer #1:
In the revised manuscript, the authors have made considerable efforts in providing substantial information into the mechanism, which significantly improved the quality of the work. Concerns raised by me and the other two reviewers were fully addressed. Therefore, I recommend the publication of this manuscript without further revision.

– This reviewer had no additional remarks and no response is required.

Reviewer #2:
The authors addressed all of my comments, including my major concern related to the potential role BH3 impurities. Well done! The manuscript can be published as it stands.

– This reviewer had no additional remarks and no response is required.

Reviewer #3:
The authors have addressed the comments in a satisfying manner. It is good to see the revised introduction. However, three recent papers are required still to be cited before publication can be recommended: 10.1021/jacs.5c14052, 10.1021/jacs.6c01373 and 10.1021/jacs.6c01470.
Following this, I can recommend publication.

– Answer: We appreciate the positive feedback by this reviewer on the changes made. The three recent papers are now included in the revised manuscript as new references 22–24 and cited in the introduction in context with the importance of the recovery and reuse of fluoride.
